# CPEB3 low-complexity motif regulates local protein synthesis via protein–protein interactions in neuronal ribonucleoprotein granules

**DOI:** 10.1073/pnas.2114747120

**Published:** 2023-01-30

**Authors:** Lenzie Ford, Arun Asok, Arielle D. Tripp, Cameron Parro, Michelle Fitzpatrick, Christopher A. de Solis, Po-Tao Y. Chen, Neeva Shafiian, Luana Fioriti, Rajesh K. Soni, Eric R. Kandel

**Affiliations:** ^a^Mortimer B. Zuckerman Mind Brain Behavior Institute, Columbia University, New York, NY 10027; ^b^Department of Neuroscience, Columbia University, New York, NY 10027; ^c^Dulbecco Telethon Institute, Istituto di Ricerche Farmacologiche Mario Negri, Milan 20156, Italy; ^d^Proteomics and Macromolecular Crystallography Shared Resource, Herbert Irving Comprehensive Cancer Center, Columbia University Irving Medical Center, New York, NY 10032; ^e^HHMI, Chevy Chase, MD 20815; ^f^Kavli Institute for Brain Science, Columbia University, New York, NY 10027

**Keywords:** biomolecular condensate, processing body, P body, RNA-binding protein, CPEB3

## Abstract

Biomolecular condensates, membraneless organelles found throughout the cell, play critical roles in many aspects of cellular function. Ribonucleoprotein granules (RNPs) are a type of biomolecular condensate necessary for local protein synthesis and are involved in synaptic plasticity and long-term memory. Most of the proteins in RNPs possess low-complexity motifs (LCM), allowing for increased promiscuity of protein–protein interactions. Here, we describe the importance of protein–protein interactions mediated by the LCM of RNA-binding protein cytoplasmic polyadenylation element binding protein 3 (CPEB3). CPEB3 is necessary for long-term synaptic plasticity and memory persistence, but the mechanisms involved are still not completely elucidated. We now present key mechanisms involved in its regulation of synaptic plasticity. We find that CPEB3-LCM plays a role in appropriate local protein synthesis of messenger ribonucleic acid (mRNA) targets, through crucial protein–protein interactions that drive localization to neuronal Decapping protein 1 (DCP1)-bodies. Translation-promoting CPEB3 and translation-inhibiting CPEB1 are packaged into neuronal RNP granules immediately after chemical long-term potentiation is induced, but only translation-promoting CPEB3 is repackaged to these organelles at later time points. This localization to neuronal RNP granules is critical for functional influence on translation as well as overall local protein synthesis (measured as α-amino-3-hydroxy-5-methyl-4-isoxazolepropionic acid (AMPA) insertion into the membrane and localization to the synapse). We therefore conclude that protein–protein interaction between the LCM of CPEB3 plays a critical role in local protein synthesis by utilizing neuronal RNP granules.

## Significance

Cytoplasmic polyadenylation element binding protein 3 (CPEB3) is necessary for long-term memory persistence, but the mechanisms behind this function are still unknown. Here, we describe the role of the low-complexity motif of CPEB3 in local protein synthesis of mRNA targets, through crucial protein–protein interactions that drive localization to neuronal ribonucleoprotein granules. This localization to neuronal ribonucleoprotein granules is critical for functional influence on translation as well as overall local protein synthesis.

Local protein synthesis is a critical component of long-term memory, allowing for the morphological changes observed in activated neurons, such as synapse enlarging, spine formation, and AMPA-receptor insertion (1). A key component of local protein synthesis near active synapses is the presence of ribosomes, mRNAs, and translation-associated proteins (1). Interestingly, these key components are often compartmentalized into membraneless organelles known as ribonucleoprotein granules (RNPs). RNPs contain a high concentration of mRNAs and proteins involved in translation and are shuttled from the soma to distal processes by motor protein movement along the cytoskeleton (2).

Over the past two decades, P bodies have been implicated in localized neuronal translation (3). Seminal work conducted in nonmammalian and somatic cells found that P bodies contain mRNA destined for degradation through the RNA-induced silencing complex (4). Paradoxically, P bodies are known to play a dual role in both degradation and translation within neurons. Indeed, a number of RNA-binding proteins involved in synaptic plasticity, such as Staufen, fragile X mental retardation protein (FMRP), zipcode binding protein 1, and La, have been found in neuronal P bodies (5–7). Some of the proteins within P bodies move to distal processes in order to promote translation in an N-methyl-D-aspartate (NMDA)-dependent manner (6), seemingly after leaving the P body (8). Little is known about how proteins move from a translation-inhibiting P body to a translation-promoting P body/neuronal granule. Here, we utilize the well-characterized RNA-binding protein cytoplasmic polyadenylation element binding protein 3 (CPEB3) to address this gap in knowledge.

**Fig. 1.**
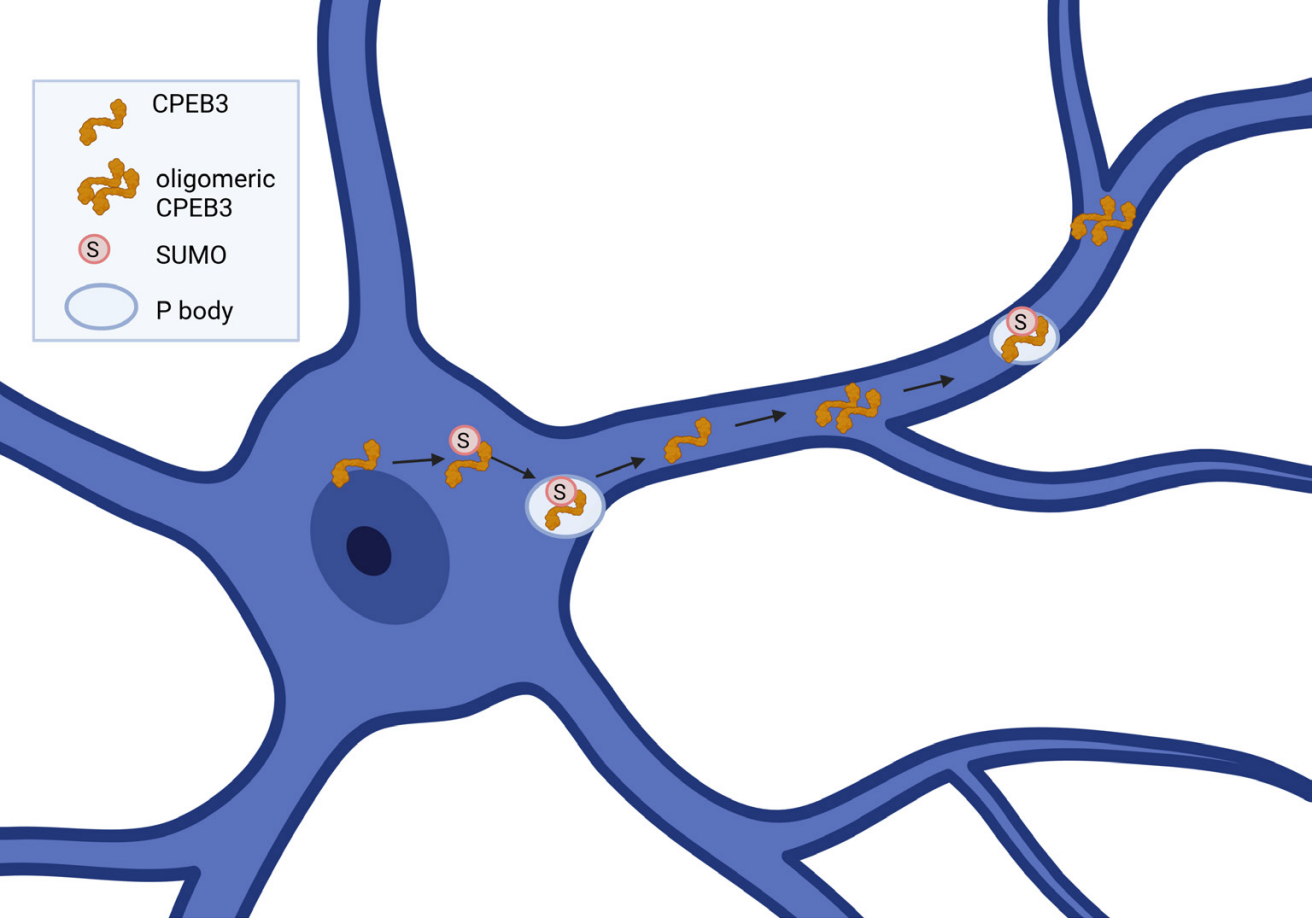
CPEB3 protein regulation in the neuron. A sequence diagram and spatiotemporal cellular diagram of CPEB3. CPEB3 exits the nucleus via sequences in the zinc-finger domain and Prion Domain 2. CPEB3 is SUMOylated (pink circle with S) and moves to the P body in the basal state and early time points of long-term potentiation (LTP). This is dependent on sequences in the RNA Recognition Motif 1. Thirty minutes post-LTP, CPEB3 is deSUMOylated and leaves the P body. By 1 h post-LTP, CPEB3 is located in the polysome, as well as again in P bodies. CPEB3 at the polysome is oligomerized, due to sequences in Prion Domain 1. Low-complexity motif (LCM).

CPEB3 is a multifaceted RNA-binding protein with prion-like properties that is regulated by what appears to be a complex relationship between the cellular environment and the protein’s structure. We have developed a working model of CPEB3 regulation in the neuron (Fig. 1): CPEB3 is soluble in the basal state and functions to inhibit translation of mRNA targets (9–12). Upon neuronal activation, CPEB3 undergoes a structural change that makes the protein semiinsoluble and switches CPEB3 to a state that promotes translation of mRNA targets (9, 13–15). This structure–function relationship is regulated in part by small ubiquitin-like modifier (SUMO)-ylation, RNA binding, and P body targeting (10, 16). In the basal state, CPEB3 is SUMOylated and localized in P bodies; however, 30 min following chemical long-term potentiation (chemLTP), CPEB3 is deSUMOylated (16) and leaves P bodies; by 1 h post-chemLTP, CPEB3 relocates to the polysome (16). Presumably, this is where oligomeric CPEB3 promotes translation of its mRNA targets (9, 11).

We do not yet fully understand the regulatory mechanisms behind CPEB3’s movement between translation-inhibiting and translation-promoting membraneless organelles. To address this, we performed a series of mutagenesis experiments and identified that the low-complexity motif (LCM), and more specifically critical residues (S240-S242), mediates CPEB3 movement and activity in the stimulated neuron. In this study, we find that CPEB3 localizes to neuronal RNP granules and identify a pattern of relocalization to the DCP1-containing neuronal RNP granules (hereon called a DCP1-body) after neuronal stimulation. Next, we identified the mechanisms behind CPEB3 localization to these membraneless organelles and the functional consequences of mutating these elements. We found that the LCM of CPEB3 is necessary for DCP1-body localization and, when disrupted, inhibits the translation-inhibiting function of basal state CPEB3. Using mass spectroscopy, we identify a disruption to protein–protein interactions and phosphorylation of LCM-mutated CPEB3. With the DCP1-body localization mechanism identified, we then investigated the impact of LCM-mutated CPEB3 on local protein synthesis by measuring AMPAfication of distal sites. While more GluA2 is produced in LCM-mutated CPEB3, the GluA2 is no longer inserted into the membrane in distal processes. Thus, we identify that cyclical relocalization of CPEB3 to a translation-promoting DCP1-containing-body is necessary for distal site local protein synthesis and that protein–protein interactions within the LCM of CPEB3 drive this required localization.

## Results

### CPEB3 Is Present in Neuronal RNP Granules.

**Fig. 2.**
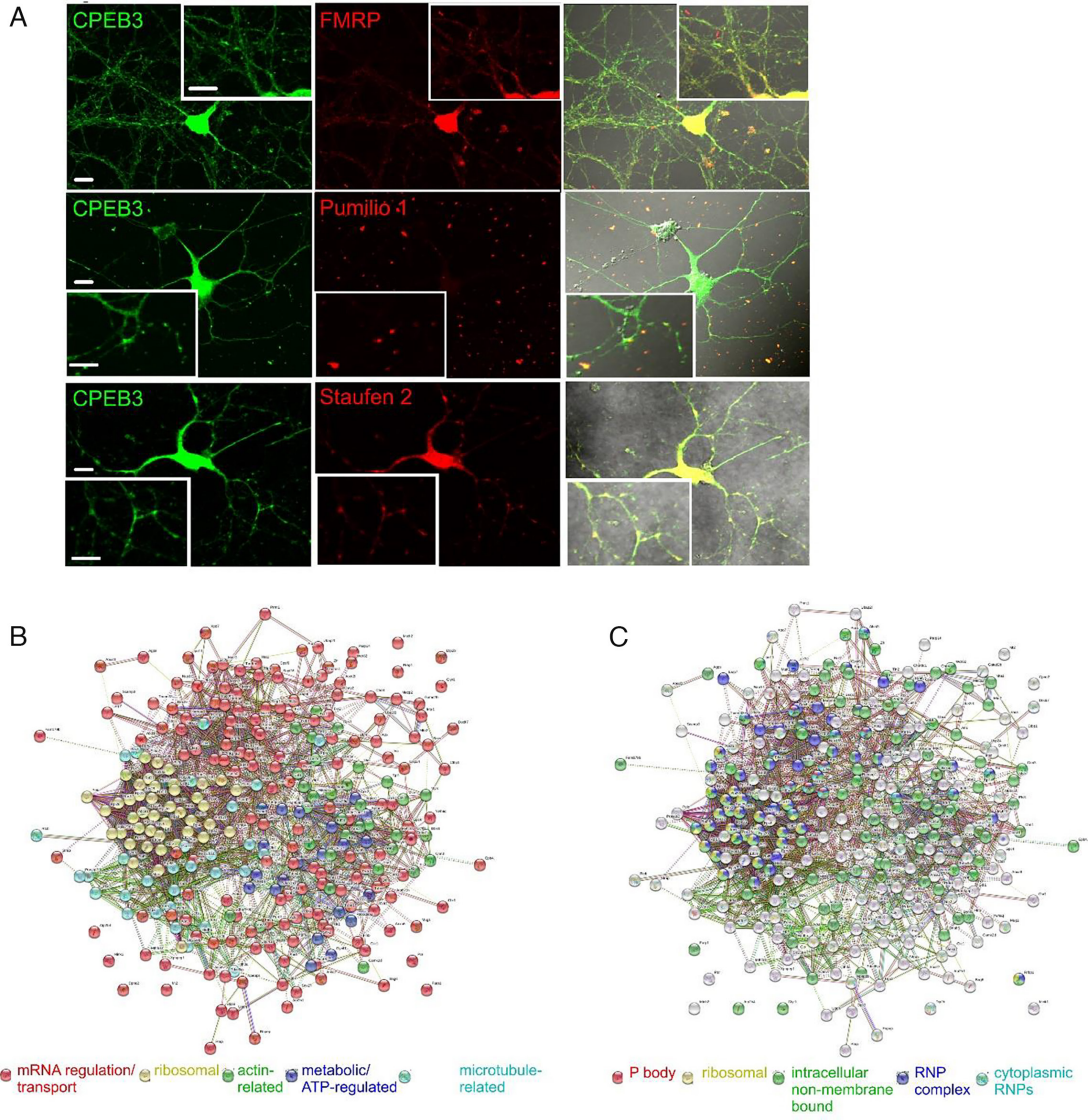
CPEB3 is present in neuronal RNP granules. (*A*) CPEB3 (green) and FMRP, Pumilio 1, or Staufen 2 (red) in mouse primary hippocampal neurons and colocalized (merge). Magnified inserts in each panel. (Scale bars, 10 mm.) (*B*) CPEB3 interactome data (interacting partners identified using mass spectrometry of CPEB3 pull-down) were clustered using k means with 5 clusters, which identified groups that were mRNA processing- or transport-related (red), ribosomal (yellow), actin-related (green), metabolic/ATP-regulated (blue), and microtubule-related (cyan). (*C*) CPEB3 interactome (interacting partners identified using mass spectrometry of CPEB3 pull-down) was clustered using Cellular Component Gene Ontology and found that CPEB3 interacts with proteins in various RNPs: cytoplasmic RNPs (cyan), intracellular non–membrane-bounded organelles (green), ribonucleoprotein complexes (blue), and P bodies (red). CPEB3 also interacts with ribosomal proteins (yellow).

In neurons, P bodies are believed to function similarly to Staufen- and FMRP-containing neuronal RNP granules (5). Therefore, we questioned whether CPEB3, which is localized to P bodies, is also localized to neuronal RNP granules. We observed CPEB3 and FMRP, Pumilio 1, and Staufen 2 colocalization in mouse primary neuronal cultures (Fig. 2*A*). To explore the functional relevance of CPEB3 localization within the neuronal RNP granule, we sought to define all neuronal RNP granules in which CPEB3 is present. First, we identified the CPEB3 interactome (Fig. 2*B*). To build an interactome, we expressed CPEB3-HA (Human influenza hemagglutinin) or CPEB3-GFP in HEK293T cells under a CMV promoter and immunoprecipitated via the respective tag. We used two different tags to exclude the possibility of nonspecific interaction due to the tag. We performed on-bead trypsin digestion and measured peptides using mass spectrometry. A total of 1,492 proteins were identified in both the CPEB3-HA and CPEB3-GFP samples (n = 5) (Dataset S1). CPEB3-HA was also expressed in mouse primary cortical neurons under a human synapsin promoter, immunoprecipitated via the HA tag, and digested on-bead with trypsin before being subjected to mass spectrometry. Across three samples, 2,294 proteins were identified (Dataset S2). To enrich for the most conserved interactors, we identified 293 proteins from the HEK293T and neuron data combined. We analyzed the data using Strings k means clustering (17) and identified groups that were mRNA processing or transport-related (red), ribosomal (yellow), actin-related (green), metabolic/ATP-regulated (cyan), and microtubule-related (blue) (Fig. 2*B* and *SI Appendix*, Table S1).

Gene ontology (GO) analysis using PantherGO (18, 19) revealed that CPEB3 interacts with proteins in various RNPs: cytoplasmic RNPs (cyan), intracellular non–membrane-bounded organelles (green), ribonucleoprotein complexes (blue), and P bodies (red). CPEB3 also interacts with ribosomal proteins (yellow) (Fig. 2*C* and *SI Appendix*, Table S1). Interestingly, P body-related proteins do not overlap with ribosomal proteins in the GO clustering. This is consistent with our hypothesis that CPEB3 is stored in P bodies in the basal state and leaves the P body by 30 min post-chemLTP, relocating to the polysome by 1 h post-chemLTP (16). Thus, we would not expect to see overlap of CPEB3 interactors in the P body and ribosome clusters, and our interactome data confirm this prediction.

From the clustering, it became apparent that CPEB3 interacts with well-defined complexes involved in neuronal mRNA trafficking. CPEB3 interacts with the exon junction complex (EJC) proteins Eukaryotic Translation Initiation Factor 4A3 (eIF4A3) and Mago Nashi (Fig. 2 *B* and *C*). EJC proteins bind to mRNAs during splicing and carry them to dendrites within RNP granules (20–22). Additionally, CPEB3 interacts with all three components of the long-term potentiation-relevant CYFIP1–eIF4e–FMR1 complex, which inhibits mRNA translation through the RNA-binding protein FMRP (23, 24) (Fig. 2 *B* and *C*).

### Neurons Concentrate Translation-Enhancing Proteins in Membraneless Organelles.

**Fig. 3.**
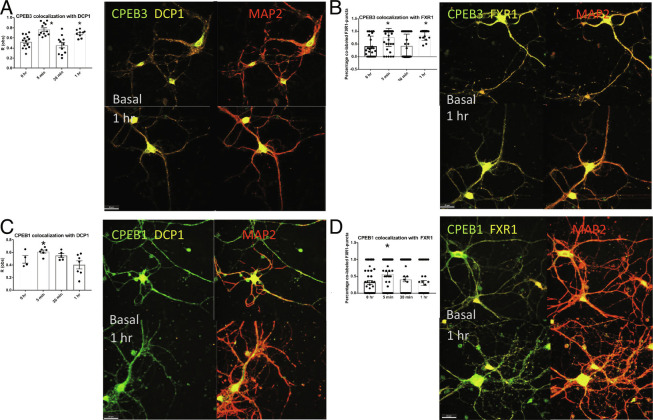
Neurons concentrate translation-enhancing proteins in membraneless organelles. (*A*) Graph of CPEB3 colocalization to DCP1 measured at 0 h, 5 min, 30 min, and 1 h; 5 min and 1 h are significantly increased from 0 h and 30 min (*P* < 0.001; n = 5). CPEB3 (green) and DCP1 (red) representative images at 0 min and 1 h. (*B*) Graph of CPEB3 colocalization to FXR1 measured at 0 h, 5 min, 30 min, and 1 h; 5 min and 1 h are significantly increased from 0 h and 30 min (*P* < 0.001; n = 5). CPEB3 (green) and FXR1 (red) representative images at 0 min and 1 h. (*C*) Graph of CPEB1 colocalization to DCP1 measured at 0 h, 5 min, 30 min, and 1 h; 5 min is significantly increased from 1 h (*P* = 0.025; n = 5). CPEB1 (green) and DCP1 (red) representative images at 0 min and 1 h. (*D*) Graph of CPEB1 colocalization to FXR1 measured at 0 h, 5 min, 30 min, and 1 h; 5 min is significantly increased from 0 h and 1 h (*P* = 0.0134; n = 5). CPEB (green) and FXR1 (red) representative images at 0 min and 1 h. One-way ANOVA and Tukey’s multiple comparisons; significance, *P < 0.05.

CPEB3 is concentrated into both P bodies and neuronal RNP granules and interacts with proteins involved in mRNA transport and translation (Fig. 2). We hypothesized that this localization promotes synaptic maintenance over time by providing necessary components of local protein synthesis. To investigate, we considered the translation-promoting CPEB3 and translation-inhibiting CPEB1. To avoid artifacts of overexpression, we measured the colocalization of endogenous CPEB3 with endogenous DCP1-containing or FMR1 Autosomal Homolog 1 (FXR1)-containing RNPs. Mouse primary neurons were subjected to chemLTP, and samples were collected at basal, 5 min, 30 min, and 1 h post-chemLTP. We found that the CPEB3 presence in DCP1-bodies increases immediately after stimulation, leaves the DCP1-body at 30 min, and is relocalized to DCP1-bodies by 1 h (Fig. 3*A*), confirming our previous finding (16). CPEB3 shows similar movement in FXR1-containing neuronal granules (Fig. 3*B*). This cyclical movement suggests that immediately after stimulation, CPEB3 is packaged into membraneless organelles for appropriate trafficking. By 30 min, it leaves these organelles, probably to perform its functional role. By 1 h, CPEB3 is repackaged into membraneless organelles at significantly increased levels from the basal state. This may provide a means of priming neurons for the site-specific local protein synthesis needed for the growth, maturation, and strengthening of synapses. Indeed, packaging proteins and transcripts into organelles could enhance the lifespan of these components at sites where they are most needed. We next tested how an inhibitory RNA-binding protein, CPEB1, is localized to membraneless organelles after chemLTP. CPEB1 has a well-characterized role in translation inhibition and is also localized to membraneless organelles(25). We found that CPEB1 moves within DCP1-bodies and FXR1-containing neuronal RNP granules distinctly. In both the DCP1-bodies and FXR1-containing neuronal RNP granules, CPEB1 has a significantly increased colocalization after stimulation, but the colocalization drops by 1 h post-chemLTP. This is intriguing as we know that CPEB1 forms mRNP complexes to inhibit mRNA translation (25). Upon stimulation, these complexes are localized to the postsynapse and recruit poly(A)-binding protein, initiating translation of the mRNA targets (25). Both CPEB3 and CPEB1 are inhibitory in the basal state and localize to membraneless organelles immediately after stimulation, likely for trafficking to sites where local protein synthesis will occur. However, CPEB3 is relocalized to these organelles by 1 h when CPEB1 is not, possibly to prime the neuron for synaptic strengthening.

### The LCM of CPEB3 Regulates the Ability to Inhibit Translation.

In order to understand how CPEB3 is localized to membraneless organelles, we created a minimally mutated CPEB3 that was no longer able to form puncta. First, we utilized an array of bioinformatic tools (*SI Appendix*, Fig. S1) and identified 16 mutations that could possibly lead to morphological differences based on the high likelihood for posttranslational modification, protein–protein interaction, formation of super secondary structure, and splice variation (*SI Appendix*, Fig. S2). S240-242A was the only mutant screened that had a significant difference in morphology compared to the full-length CPEB3 (*SI Appendix*, Fig. S2). Additionally, we measured the influence of the 16 mutant CPEB3s on translation using a luciferase-based reporter system. A SUMO-3′-UTR-Renilla plasmid was coexpressed alongside the mutant CPEB3, allowing translation of the SUMO-3′-UTR-Renilla to be measured as luminosity (10). The S240-242A produced an increase in translation, indicating that the mutant could not inhibit translation of the target. (*SI Appendix*, Fig. S2). Amino acids 240 to 242 are within the uncharacterized LCM (11), which has a high probability of protein–protein interaction (*SI Appendix*, Fig. S3). Residues 240 to 242 flank a unique primary sequence; amino acids 220 to 242 contain a stretch of hydrophobic alanines flanked by commonly posttranslationally modified residues: SKPSSSSAVAAAAAAAAASSASSS. We modified all serines within the 220 to 242 stretch to assess the most important amino acids for the observed functional changes in *SI Appendix*, Fig. S2. Of the 14 mutants screened, the serine to alanine mutation of amino acids 240, 241, and 242 (identified as S240-242A) was disruptive to function (*SI Appendix*, Fig. S4).

### The CPEB3 LCM Is Involved in P Body Localization.

**Fig. 4.**
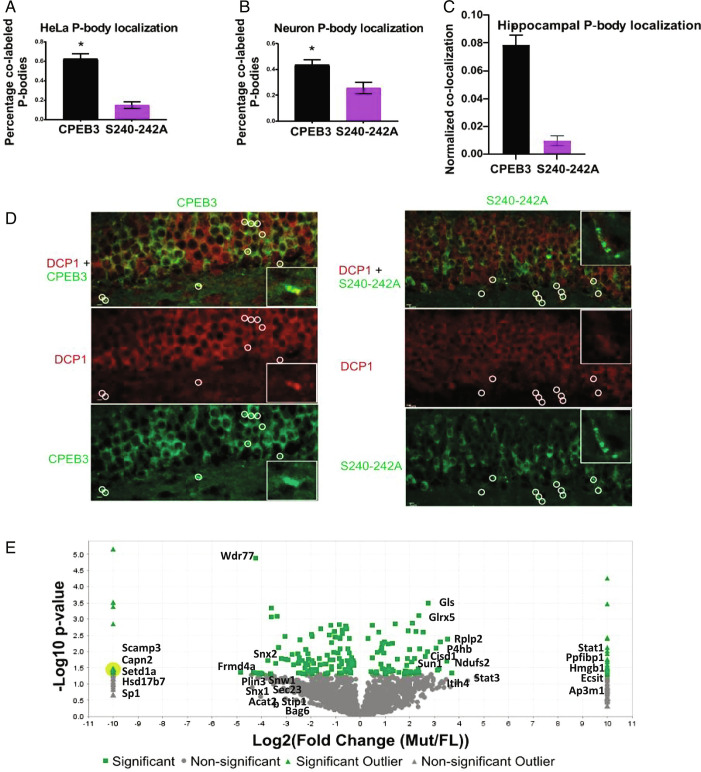
The LCM localizes CPEB3 to membraneless organelles. (*A*–*C*) Graphical representation of colabel of P body marker DCP1 and either CPEB3 or the S240-242A mutant in HeLa cells (*A*; *P* < 0.0001, n = 30), stimulated state neurons (*B*; *P* = 0.0072, n = 21), or mouse forebrain (*C*; *P* = 0.0021, n = 8; technical repeats = 5). Student's *t* test was used; significance, *P < 0.05. (*D*) Colocalization of DCP1 (red) and CPEB3 or S240-242A (green) mouse forebrain (scale bars, 5 µm). *Insets* offer a magnified region of interest. White circles indicate P body location to indicate the presence or lack of colocalization. Images were enhanced for the figure; images were not altered for data analysis. (*E*) Volcano plot of full-length CPEB3 and mutant S240-242A interactomes. Significance, *P* > 0.05, Fischer's *t* test.

Because the S240-242A region disrupts puncta morphology and is necessary for CPEB3 inhibitory function, we hypothesized that modification of the LCM would also disrupt CPEB3 localization to DCP1-bodies. To test this hypothesis, we measured the colocalization of the S240-242A mutant with DCP1-bodies using the Colocalization Test in Fiji (26). When the S240-242A mutant is expressed in HeLa cells, the mutant localizes to DCP1 significantly less than the full-length protein (Fig. 4*A*). When full-length CPEB3 or the S240-242A mutant was expressed in primary hippocampal cultures, the mutant again localized much less to DCP1 than the full length (Fig. 4*B*).

Although our findings provide strong support for the necessity of the S240-242A site in localizing CPEB3 to DCP1-bodies in vitro, we wanted to determine whether this was also true in vivo. We thus generated two viral constructs under the control of the alpha calcium calmodulin-dependent protein kinase 2 (aCamKII) promoter that contained either the mutated CPEB3 (AAV-DJ8-(0.4)aCamKII-S240-242A-HA-P2A-eGFP) or wild-type CPEB3. We injected these constructs bilaterally into the dorsal hippocampus of wild-type mice and sacrificed the animals 2 wk later. We sectioned and probed hippocampal slices for HA and DCP1. We measured colocalization of HA and DCP1 using the Spots function in Imaris. Consistent with our in vitro work, mice expressing full-length CPEB3 exhibited significantly more HA-DCP1 colocalization than mice injected with the S240-242A mutant (Fig. 4 *C* and *D*).

### Protein–Protein Interactions and CPEB3 Phosphorylation Are Dysregulated in the S240–242A Mutant.

To verify that the S240-242A mutant is excluded from P bodies and neuronal RNP granules, we compared the interactome of mouse neuroblastoma N2A-expressed CPEB3 and S240-242A. Using Fischer’s test, we identified 103 proteins that were significantly underrepresented in the S240-242A samples, including membraneless organelle and synapse components (Fig. 4*E* and Dataset S3). Of these 103 proteins, GO cellular component analysis found that protein components of intracellular non–membrane-bounded organelles, non–membrane-bounded organelles, and ribonucleoprotein complexes were significantly enriched in full-length CPEB3 compared to the S240-242A mutant (2.19-fold, *P* = 3.75^−3^; 2.23-fold, *P* = 1.42^−5^; 6.49-fold, 1.69^−8^, respectively). Thus, the S240-242A mutation lacks the ability to interact with several proteins often bound by full-length CPEB3 and significantly lacks interaction with membraneless organelle components. These proteins are Atrx, Bop1, Ckap2, Cmss1, Ctcf, Ddx46, Ehmt1, Fxr1, Fmr1, Ilk, Imp3, Kif4, Mark4, Mms19, Msh6, Nek9, Pard3, Phf8, Polr1e, Rsl24d1, Sec13, Sept6, Setd1b, Sp1, Srp14, Tsr1, Utp23, and Vapa. We were particularly interested to observe the decreased binding of the S240-242A mutant with neuronal RNP granule protein FMR1 and its paralog FXR1. It is this protein–protein interaction, and not a difference in RNA binding, that is important for the observed localization. We expressed CPEB3-GFP or the S240-242A-GFP mutant in HT22 cells and performed an RNA immunoprecipitation. RNA was extracted, sequenced, and analyzed for differentially bound RNA species. We found no difference in RNA pulled down with CPEB3 and the S240-242A mutant (*SI Appendix*, Fig. S5).

The S240-242A mutant disrupts appropriate function and protein–protein interaction, and there is a high likelihood that phosphorylation at 240, 241, or 242 regulates this functioning (*SI Appendix*, Fig. S1). Unfortunately, we could not analyze posttranslational modifications in the 240 to 242 amino acid sequence; no peptides containing this region of the protein could be recognized in the mass spectrometry analysis. Instead, we identified 2 phosphorylation sites that were present in CPEB3 samples but not in the S240-242A mutant samples (Dataset S4). Of note, 66% of CPEB3 samples were phosphorylated at amino acids S285 and S443, while 0% were phosphorylated in the S240-242A samples (Dataset S4). These phosphorylation sites were identified previously in CPEB3-expressing HEK cells (27). Further studies are needed to understand how the S240-242A mutant affects S285 and S443 phosphorylation, but it is interesting to note that basal state phosphorylation, as well as basal inhibitory function, is disrupted in the mutant.

### The LCM of CPEB3 Is Necessary for Local Protein Synthesis in Neurons.

**Fig. 5.**
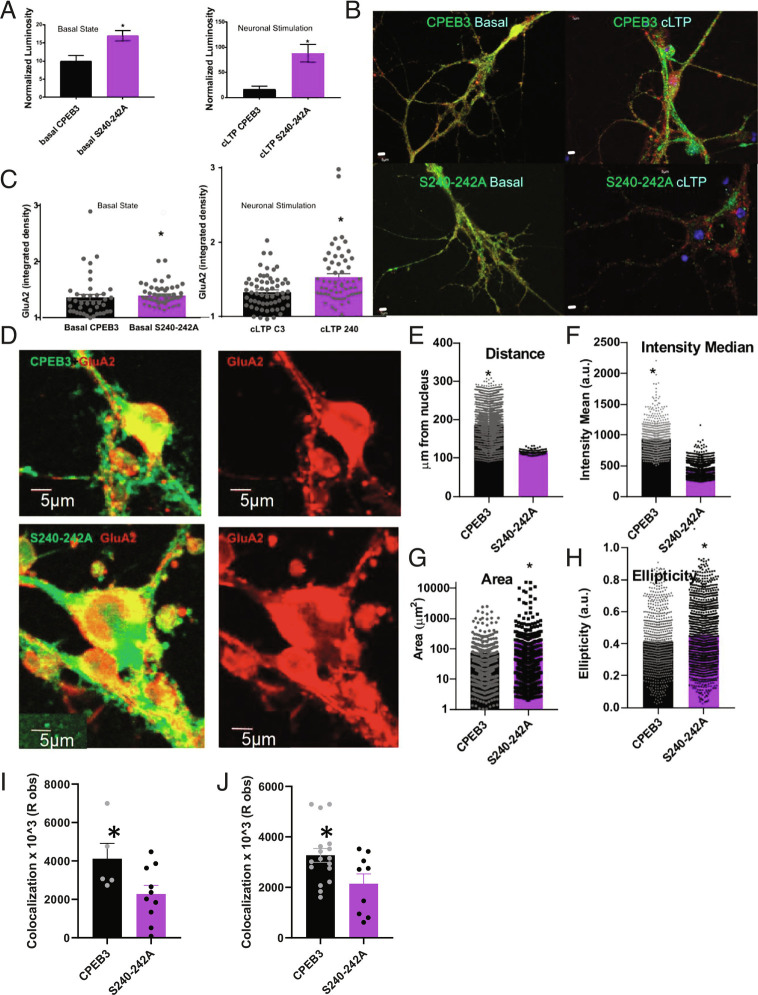
CPEB3 LCM plays a role in AMPAfication. (*A*) Full-length CPEB3 or the S240-242A mutants were expressed in primary neuronal cultures along with a SUMO2-3′UTR-Renilla reporter plasmid. Translation of the reporter was measured using a luciferase assay, and luminosity was measured as a readout of translation. The S240-242A mutant (purple) has significantly increased luminosity in a translational assay compared to full-length CPEB3 (black) in basal (*C*; *P* = 0.0042, n = 8) and stimulated (*D*; *P* = 0.0004, n = 9) conditions. (*B* and *C*) CPEB3 or S240-242A (green) were expressed in primary neuronal cultures, and GluA2 (red) was measured under basal and chemLTP stimulated conditions; DAPI in blue. Neurons expressing the S240-242A mutant have significantly increased GluA2 levels in distal sites compared to neurons expressing CPEB3 in the basal (*E*; *P* = 0.0006, n = 40) and stimulated (*F*; *P* = 0.0004, n = 27) condition. Student's *t* test was used to determine significance, *P < 0.05. (*D*) Membrane-bound GluA2 (red) expression in CPEB3 or S240-242A (green)-expressing neurons. (*E*) Distance of membrane-bound GluA2 from the nucleus (*P* < 0.005; n = 5; technical replicate = 5). (*F*) Area of membrane-bound GluA2 (*P* = 0.015; n = 5; technical replicate = 5). (*G*) Median intensity value of membrane-bound GluA2 (*P* < 0.005; n = 5; technical replicate = 5). (*H*) Ellipticity of membrane-bound GluA2 (*P* < 0.005; n = 5; technical replicate = 5). (*I*) Colocalization of extracellular AMPAR and bassoon (*P* = 0.05; n = 3; technical replicates = 2). (*J*) Colocalization of renilla and bassoon (*P* = 0.025; n = 5; technical replicates = 3). Student’s *t* test determined statistical significance. * indicates statistical significance if *P* < 0.05. (Scale bars, 5 µm.) Images were enhanced for the figure; images were not altered for data analysis.

We now understand that protein–protein interactions with the LCM of CPEB3 are critical for its localization to membraneless organelles. From our initial studies in HEK cells, the S240-242A LCM mutation also disrupted basal state function (*SI Appendix*, Figs. S2 and S3). Next, we tested the effect of the mutant on activity-dependent translation. Neurons were transfected with either wild-type CPEB3 or the S240-242A mutant and a SUMO 2 3′-UTR Renilla reporter (10), and 10 d later, they were stimulated using chemLTP. Lysate was collected immediately or at 1 h. We found that the S240-242A mutant displayed significantly increased translation compared to full-length CPEB3 (Fig. 5*A*). We verified these results by measuring levels of an endogenous target, GluA2, in CPEB3- or S240-242A-expressing neurons. In the basal and stimulated state, GluA2 expression was increased in S240-242A-expressing neurons compared to CPEB3-expressing neurons (Fig. 5 *B* and *C*)

The S240-242A mutant 1) has decreased localization to P bodies and neuronal RNP granules, 2) has decreased protein–protein interactions with RNP-related proteins and disrupted phosphorylation, and 3) no longer exhibits inhibitory function.

Local protein synthesis at the synapse is necessary for LTP and long-term memory, and so, an overall increase in translation may not fulfill the needs of the neuron if mislocalized. AMPAfication, or the insertion of AMPA receptors into the synaptic membrane, offers a well-established example of the need for localized protein synthesis in synaptic plasticity (for review, see refs. 1, 28, and 29). We hypothesized that disruption to CPEB3 function and localization would disrupt the appropriate AMPAfication of neurons. To test our hypothesis, we first compared the spatial expression of membrane-bound GluA2 in CPEB3- and S240-242A-expressing neurons (Fig. 5*D*). We analyzed data using the “spots” and “surface” functions in Imaris, with Matlab XTensions. We found that membrane-bound GluA2 in CPEB3-expressing neurons were located throughout the neuron, where distance was measured as GluA2 spot location from a nuclear surface (Fig. 5*E*). However, the S240-242A mutant-expressing neurons contained membrane-bound GluA2 localized much closer to the nuclear surface, suggesting that mutated CPEB3 disrupts appropriate GluA2 trafficking and/or insertion into the membrane at distal sites (Fig. 5 *D* and *E*). Overall, CPEB3 appears to influence membrane-bound GluA2 expression throughout the neurons, with more intense membrane-bound GluA2 expression than the mutant (Fig. 5*F*). The S240-242A mutant severely disrupted membrane-bound GluA2 expression: Expression was largely somal, with large elliptical “rafts” of membrane-bound GluA2 forming (Fig. 5 *G* and *H*). This decrease in membrane-bound GluA2 distal expression in the S240-242A mutant was validated by measuring the colocalization of extracellular GluA2 and the postsynaptic marker Bassoon (Fig. 5*I*). Interestingly, total GluA2 expression is increased in S240-242A-expressing neurons (Fig. 5 *B* and *C*), but more of the GluA2 appears to be membrane-bound in full-length CPEB3-expressing neurons than in the mutant-expressing neurons (Fig. 5 *D* and *E*). Furthermore, we measured the expression of a GluA2 3′-UTR-Renilla target and its colocalization with the postsynaptic marker Bassoon. The target had significantly diminished colocalization to the postsynapse in neurons expressing the S240-242A mutant compared to full-length CPEB3 (Fig. 5*J*). Because of the disruption in the expression pattern of distal, membrane-bound GluA2, we find that mutation of the LCM of CPEB3 disrupts localization and insertion of AMPA receptors after chemLTP.

## Discussion

Here, we investigate how neurons target components involved in synaptic plasticity using membraneless organelles. Using the RNA-binding protein CPEB3, we found that proteins can move from P bodies and neuronal RNP granules after chemLTP and that this is critical for local protein synthesis. We know that RNA binding and SUMOylation are necessary for CPEB3’s localization to P bodies (16); similar regulation is necessary for proteins La, heterogeneous nuclear ribonucleoprotein (hnRNP) M and C, and FMRP (2, 30–33). Additionally, CPEB3 requires protein–protein interaction through its LCM to target membraneless organelles in the neuron. The LCM has been implicated in the biophysical liquid–liquid phase separation observed in many proteins, including U1 small nuclear ribonucleoprotein 70 kDa (U1-70K) (34), Fused in sarcoma (FUS) (35, 36), hnRNP A (37), and FMRP (38). This regulation of CPEB3 draws parallels to the well-characterized FMRP, which is necessary for local protein synthesis, is located in P bodies and RNPs, inhibits translation of mRNA targets, exhibits a reciprocal function when SUMOylated in distal processes, and is required for long-term memory [for review, see refs. 1–2; 32, 33)]. Do universal regulatory mechanisms exist for RNA-binding proteins involved in synaptic plasticity? Of the examples presented above, it appears plausible. Furthermore, CPEB4, GRB10 interacting GYF protein 2 (GIGYF2), Caprin 1, and FMR1 Autosomal Homolog 2 (FXR2) are believed to be involved in RNP-mediated mRNA trafficking which promotes local protein synthesis, as well (38). It will be interesting to investigate whether the cyclical localization to membraneless organelles, as observed with CPEB3 in Fig. 3, is canonical for translation-promoting proteins.

In-depth understanding of CPEB3 neuronal regulation could provide insight into a universal mechanism for mRNA trafficking in synaptic plasticity. We know that soluble CPEB3 binds mRNA targets and inhibits translation (9). Soluble, inhibitory CPEB3 is found in P bodies (16). The P body-contained CPEB3 is SUMOylated, which is necessary for the inhibitory function of the protein (10). Upon chemLTP, CPEB3 is deSUMOylated, leaves the P body at a time point coinciding with peak SUMO-specific peptidase I activity (39), and localizes to the polysome (11, 16). This activated CPEB3 is monoubiquitinated and phosphorylated (27, 40), promotes translation of its mRNA targets, and is semiinsoluble and oligomeric (9). We have now found that CPEB3 requires an intact LCM to localize to P bodies and neuronal RNP granules. Mutation of the LCM results in a disruption to protein–protein interactions between CPEB3 and binding partners within P bodies and neuronal RNP granules.

What are the similarities and differences in regulation of RNA-binding proteins involved in synaptic plasticity? Subsets of neuronal RNP granules appear to be signaling cascade–dependent and have defined protein and mRNA contents (41–43). Our comparison of CPEB3 and CPEB1 movement from neuronal RNP granules confirmed this. CPEB3 and CPEB1 are inhibitory in the basal state and localize to membraneless organelles immediately after stimulation, likely for trafficking to sites where local protein synthesis will occur. However, CPEB3 is relocalized to these organelles by 1 h when CPEB1 is not, possibly to prime the neuron for synaptic strengthening. This possibility echoes the hypothesis of our lab that CPEB3 is primed at synapses in a degradation-resistant state as a tag for active neuronal connections (44). There must be an important determination for which proteins are localized to specific subsets of neuronal RNP granules, but this mechanism is unknown. We hypothesize that protein–protein interaction through intrinsically disordered regions, with neuronal RNP granule components, allows for specificity.

Some, but not all, neuronal RNP granule-bound proteins exit the neuronal RNP granule under appropriate neuronal stimulation (16, 38, 45, 46), such as CPEB3, and its LCM-interacting partner FMRP and Caprin 1 (Fig. 3). These proteins have been shown to phase-separate in vitro, in the presence of RNA and an appropriate PTM (16, 35, 37, 38). For both FMRP and Caprin 1, as well as FUS and hnRNP A, phosphorylation is the post-translational modification (PTM) that influences phase separation of the protein (35, 37, 38, 46). Furthermore, we know that CPEB3 is phosphorylated in its activated state (27) but do not yet understand how this phosphorylation influences phase separation or membraneless organelle localization. However, we do know that SUMOylation of CPEB3 is linked to its phase separation and membraneless organelle localization (16). PTMs of these proteins, and availability of these modifiable sites within the membraneless organelle, are critical.

Analysis of the regulatory mechanisms of CPEB3 suggests conserved mechanisms involved in the translation-enhancing RNP-mediated mRNA trafficking of synaptic plasticity. This has important implications in the neurobiology of learning and memory and our understanding of targeted local protein synthesis in neurons.

## Materials and Methods

Antibodies.

See Table 1.

**Table 1.** Antibodies used in these studies

	Immunocytochemistry	Immunohistochemistry
GFP (Abcam cat#ab13970)	1/1,000	1/1,000
HA (BioLegend cat#16B12)	1/500	1/1,000
G3BP (Abcam cat#ab56574)	1/1,000	×
DCP1a (Abcam cat#ab47811)	1/1,000	1/1,000
GluA2 (Abcam cat#ab20673)	1/750	×
FMRP (Abcam cat#ab17722)	1/1,000	×
Extracellular GluA2 (Alomone cat#AGC-005)	1/500	×

### Animals: Animal Maintenance.

Wild-type (C57BL/6J background) male mice were obtained from Jackson Laboratory (Bar Harbor, ME) at approximately 9 to 10 wk of age. Animals were housed in the Zuckerman Institute vivarium at Columbia University and maintained on a standard 12-h to 12-h light–dark cycle with ad libitum access to food and water. All animal procedures were conducted in accordance with the Institutional Animal Care and Use Committee at Columbia University.

### Animals: Viral Surgery.

For all surgeries, mice were first anesthetized with isoflurane and administered a weight-appropriate dose of Bupivacaine, a local anesthetic, subcutaneously at the surgical site and an intraperitoneal dose of Carprofen for pain. A bilateral craniotomy was made directly above the dorsal hippocampus. Next, a glass micropipette attached to a Nanoject III (Drummond Scientific, Bromall, PA) was lowered to a region just below the dorsal blade of CA1 in the dorsal hippocampus (AP = ±1.4, ML = −2.3, and DV = −2.1). Then, 500 nL of either AAV-DJ8-(0.4)aCamKII-CPEB3-HA-P2A-eGFP or AAV-DJ8-(0.4)aCamKII-S240-242A-HA-P2A-eGFP was injected at a rate of 100 nL/min for 5 min. Following surgery, mice were allowed to recover for 2 wk.

### Animals: Immunohistochemistry.

Animals were anesthetized with a mixture of ketamine and xylazine (200/20 mg/mL). Animals were then transcardially perfused with 10 mL 1× phosphate buffered saline (PBS) and 10 mL 4% paraformaldehyde. Brains were extracted and postfixed for 24 h in 4% paraformaldehyde. Brains were washed twice for 30 min in 1× PBS before being stored in 1× PBS + 0.02% NaN_3_.

Brains were sliced coronally at 40 μm on a vibratome (Leica VT1200s). Forebrain tissue was collected in 1× PBS.

Tissue was washed for 10 min in 300 μl 1× TBS (tris buffered saline) and then washed 3 times in 300 μL TBST (TBS + 0.05% Triton-x). Tissue was placed in 300 μl 1× Antigen Unmasking Solution (Citric Acid Based, H-3300, (Vector Laboratories)) and loaded into an oven at 70 °C for 1 h. Samples cooled to room temperature before being washed 3 times in 300 μL TBST. Tissue was placed in a blocking buffer consisting of 2.5% of Normal Goat Serum, 2.5% of Normal Donkey Serum, 1% BSA, and 94% TBST overnight. Tissue was stained with a combination of primary antibodies in blocking buffer, again, overnight. Finally, tissue was washed 4 times for 10 min with 300 μL TBST. Tissue was incubated in blocking buffer and secondary antibodies for 2 h at room temperature. Tissue was then washed 4 last times for 10 min in TBST. Tissue was rinsed briefly with 70% EtOH.

Tissue was mounted using Prolong Gold Antifade Reagent on Superplus Frost Slides and let dry in the dark for 24 h before being imaged.

### Cell Culture: Cell Lines.

HEK293T, HeLa, and N2A cells were cultured in Dulbecco’s modified Eagle’s medium with high glucose and L-glutamine (Gibco) with 10% fetal bovine serum (FBS) (Sigma) and 1% penicillin/streptomycin (Gibco) in a tissue-culture incubator at 37 °C and 5% CO_2_. Cells were plated in T25 or T75 flasks and grown to 40 to 60% confluency before transfection. Additionally, HEK293T were plated on 12-mm glass coverslips coated with poly-L-lysine (Sigma) in 24-well flat-bottom cell-culture plates.

HEK293T and HeLa cells were transfected with TransFast Transfection Reagent (Promega) –500 μg DNA per 24-well culture plate. N2A cells were transfected with 2 μL Lipofectamine 3000 (ThermoFisher) + 800 ng DNA per 24-well in Opti-minimal essential medium (MEM) (ThermoFisher). N2As were incubated in transfection media for 1 h and then changed to fresh complete DMEM (ThermoFisher). Cells expressed for 24 h before lysate was collected.

Cells were transfected with CPEB3-GFP (9), S240-242A-GFP [a mutation of CPEB3-GFP], CPEB3-HA (9), S240-242A-HA [a mutation of CPEB3-HA], Actin 3′UTR-Renilla (11), SUMO 2 3′UTR-Renilla (10), and mutant SUMO 2 3′UTR-Renilla (10).

### Cell Culture: Primary Neuronal Culture.

The primary neuron isolation and culture methods were adapted from Kaech and Banker, 2006 (47). Hippocampi and neocortices were dissected from P0-P2 mouse pups and isolated from meningeal tissue in an ice-cold dish of Hank’s Balanced Salt Solution. In a 15-mL Falcon tube prepared with warm papain dissociation media (Worthington Biochemical Corporation), large pieces of tissue were mechanically dissociated with forceps and then incubated in the media for 40 to 50 min at 37 °C and 5% CO_2_. The tubes were agitated every 15 min. Tissue was allowed to settle to the bottom of the tube and then transferred with a 1-mL pipette into another 15-mL tube prepared with ovomucoid protease inhibitor solution (Worthington) and incubated for 10 min at room temperature. Tissue was again allowed to settle and transferred to a third tube prepared with warm Dulbecco’s modified Eagle’s medium (Gibco) containing 10% horse serum (Gibco) and 1.6% glucose (Sigma). Tissue was gently triturated in this media with a Pasteur pipette 15 times and allowed to settle, and then, the supernatant was transferred to a final Falcon tube for dissociated cell collection. The trituration process was repeated three times by adding warm MEM to the remaining tissue, triturating, and transferring the supernatant into the collection tube. At the end of the process, dissociated cells in the collection tube were centrifuged at 1,000 g for 5 min, resuspended in warm MEM, and plated at 300 k cells/mL in 24- and 12-well cell-culture plates pretreated with poly-L-lysine (1 mg/mL, Sigma). For imaging samples, isolated neurons were plated on cleaned and poly-L-lysine–pretreated coverslips in multiwell cell-culture plates. Plated cells were incubated in the MEM for 4 h at 37 °C and 5% CO_2_. After this incubation, the MEM was carefully aspirated from each well and immediately replaced with warm Neurobasal media containing B27 supplement and GlutaMax (Gibco). One third of the media was removed and replaced with fresh Neurobasal media every 4 to 5 d. Neurons were cultured for up to 21 d at 37 °C and 5% CO_2_.

Cells were transfected at DIV 5 with Lipofectamine 3000 (ThermoFisher) and 1 μg DNA per 2-cm^2^ culture well. Or, cells were transduced with a lentivirus (modified from Malcolm Moore, Addgene plasmid #48687, RRID: Addgene_48687) and LentiX Accelerator (Takara Bio). Cells expressed for at least 24 h before being collected.

Cells were transfected with: CPEB3-GFP (9), S240-242A-GFP [a mutation of CPEB3-GFP], CPEB3-HA (9), S240-242A-HA [a mutation of CPEB3-HA], synapsin-CPEB3-HA-tdTomato, synapsin-S240-242A-HA-tdTomato, Actin 3′UTR-Renilla (11), SUMO 2 3′UTR-Renilla (10), and mutant SUMO 2 3′UTR-Renilla (10).

### Cell Culture: chemLTP.

DIV 15 neurons were placed in fresh Neurobasal media for 3 min and then moved to stimulation media for 5 min. The stimulation media contained 50 μM forskolin, 0.1 μM rolipram, and 50 μM picrotoxin in artificial cerebrospinal fluid (ACSF) without MgCl. Neurons were moved back to conditioned media.

### Cell Culture: Immunocytochemistry.

Cells were washed with cold PBS and fixed in-plate with 4% PFA in PBS (neuronal cultures additional 5% sucrose). Cells were permeabilized for 2 min with 0.1% Triton-X in PBS, blocked for 1 h at room temperature in 5% FBS in PBS, probed overnight at 4 °C with primary antibody in block, and probed for 1.5 h at room temperature with secondary antibody in block. Coverslips were mounted using Fluoroshield (Sigma).

### Mutagenesis.

A plasmid containing mCPEB3 in the pAcGFP1-N1 vector (9) was used as a template for site-directed mutagenesis using the Q5® Site-Directed Mutagenesis Kit (New England Biolabs). Complementary primers containing the desired deletion or substitution were annealed to the template plasmid, and the Q5 Hot Start High-Fidelity DNA Polymerase (NEB) replicated and incorporated the mutant primers into the template. The PCR mix was digested with Dpn1 to fragment the template DNA and then transformed into XL10-Gold Ultracompetent Cells (Agilent) to be grown overnight and have single colonies picked for DNA extraction using the QIAprep Spin Miniprep Kit. The correct DNA sequence of all mutant constructs was confirmed by DNA sequencing. Mutants were transformed into NEB® 5-alpha Competent E. coli (High Efficiency), and then, the DNA was extracted using the QIAGEN Plasmid Maxi Kit.

Splice C:

A​AAT​TTA​TTC​CCA​TGC​GAC​CTT​TCA​GAG​GCT​CAT​GGT​CGA​CCA​TGA​GCC​TCT​GAA​AGG​TCGCATGGGAATAAATTT

Splice B: C

TTCAAAGGGAAAGAGAGAAGAGGTGTTAAGTGCCATAATGTTA TAACATTATGGCACTTAACACCTCTTCTCTCTTTCCCTTTGAAG

S194A:

GCTGGCGGGCGCGCGGCGTTGCT AGCAACGCCGCGCGCCCGCCAGC

S197A:

CCTGGCTGGGGGCGGCGGGCGAGC GCTCGCCCGCCGCCCCCAGCCAGG

S237-238A:

GGACGAGGCCGCAGCGGCAGCGGCCG CGGCCGCTGCCGCTGCGGCCTCGTCC

S240-242A:

G​CTG​CCT​CTT​CGG​CCG​CGG​CCG​CCT​GGA​ACA​CGC​ACC​ATG​GTG​CGT​GTT​CCA​GGC​GGC​CGCGGCCGAAGAGGCAGC

S349A:

CATTAAGGAGTTCTCCAAGGCGTGCAAGTTAAAAGTGTCAT ATGACACTTTTAACTTGCACGCCTTGGAGAACTCCTTAATG

Del_N:

Published previously; see ref. 10.

Del_RBD:

GAACGAGTGGAACGCTACTCTCCGTACGTGCT AGCACGTACGGAGAGTAGCGTTCCACTCGTTC

S419-420A:

CAAAGGGAAAGAGAGCAGCCCGACCTCGTCTCCG. CGGAGACGAGGTCGGGCTGCTCTCTTTCCCTTTG

S444A:

AGGAGCAGCTAAGCC GGCTTAGCTGCTCCT

Y457A/S458A:

CCAACAAACACCTTTCTAGCGGCGCGTTCCACTCGTTCCCCA TGGGGAACGAGTGGAACGCGCCGCTAGAAAGGTGTTTGTTGG

K459A/R460A:

GGCCTCCAACAAACACCGCTGCAGAGTAGCGTTCCACTCGTTCCC GGGAACGAGTGGAACGCTACTCTGCAGCGGTGTTTGTTGGAGGCC

S222A:

GCCGAGGATGAGGCCGGCTTGCTGGTC GACCAGCAAGCCGGCCTCATCCTCGGC

S223A:

CCGCCGAGGATGCGGACGGCTTGCT AGCAAGCCGTCCGCATCCTCGGCGG

S224A:

CCAGCAAGCCGTCCTCAGCCTCGGCGG CCGCCGAGGCTGAGGACGGCTTGCTGG

S225A:

CGTCCTCATCCGCGGCGGTCGCG CGCGACCGCCGCGGATGAGGACG

S237A:

CGGCCGCTGCCGCTTCGGCCTCG CGAGGCCGAAGCGGCAGCGGCCG

S238A:

CGAGGCCGCAGAGGCAGCGGCCGC GCGGCCGCTGCCTCTGCGGCCTCG

S240A:

GCCTCTTCGGCCGCGTCCAGCTGGA TCCAGCTGGACGCGGCCGAAGAGGC

S242A:

GTGTTCCAGCTGGCCGAGGCCGAAGAG
CTCTTCGGCCTCGGCCAGCTGGAACAC

### Translational Assay.

Double-transfected cells (a CPEB3 and Renilla transfection) were washed in cold PBS, scraped, and lysed in the lysis buffer provided in the Dual-Glo Luciferase Assay System (Promega). Lysate luminosity was measured with a luminometer. Background luminosity was measured with a Luciferase substrate (Firefly; Promega). Experimental luminosity was measured with a Stop & Glo substrate (Renilla; Promega). Luminosity was normalized to protein concentration.

### Confocal Imaging and Analysis.

All images were acquired on an Olympus IX81 laser-scanning confocal microscope using the FluoView FV1000 Microscopy System. Cell culture images were quantified using SyNPanal (48) or ImageJ/Fiji integrated density measurements and Colocalization Test (26). Tissue images and spatial GluA2 images were quantified using the spots and surfaces functions and Matlab XTension “find spots near surface”, in Imaris. Each spot/surface selection was hand-verified. Access to Imaris software was provided by Cellular Imaging at Zuckerman Mind, Brain, Behavior Institute.

### Biochemistry: Sample Preparation and Immunoprecipitation.

Cells were washed with cold PBS and lysed in Radioimmunoprecipitation assay buffer (RIPA) + NEM (n-ethylmaleimide) buffer. Lysate was added to either 50 μL GFP-Trap magnetic bead (Chromotek) slurry or 50 μL HA-linked magnetic bead (Pierce) slurry. Lysate bound to magnetic beads overnight at 4 °C with end-over-end mixing. Beads were washed 3× with GFP-Trap Wash Buffer (Chromotek). Washed GFP-Trap beads were boiled in 2× Laemelli Buffer (Bio-Rad) and immediately loaded onto a 4 to 20% Tris-glycine SDS-PAGE gel (Bio-Rad). Washed HA beads were mixed with a 4:1 column volume HA peptide (Sigma Aldrich) for 15 min at 37 °C. Eluate was further processed for western blotting (addition of 4× Laemmeli [Bio-Rad] and boiling), additional immunoprecipitation, or RNAseq.

### Biochemistry: RNAseq.

RNA was extracted from the immunoprecipitate using TRIzol reagent. Then, 2 µg of total RNA was processed to enrich in polyA-containing mRNA and sequenced by Genewiz using Illumina Technology.

### Mass Spectrometry: Preparation of Samples.

Proteins bound to streptavidin beads were washed five times with 200 µL 50 mM ammonium bicarbonate and subjected to disulfide bond reduction with 5 mM TECP (room temperature, 30 min) and alkylation with 10 mM iodoacetamide (room temperature, 30 min in the dark). Excess iodoacetamide was quenched with 5 mM DTT (room temperature, 15 min). Proteins bound on beads were digested overnight at 37 °C with 1 µg of trypsin/LysC mix. The next day, digested peptides were collected in a new microfuge tube, and digestion was stopped by the addition of 1% TFA (final v/v) and centrifuged at 14,000 g for 10 min at room temperature. Cleared digested peptides were desalted on SDB-RP Stage-Tip and dried in a speed-vac. Peptides were dissolved in 3% acetonitrile/0.1% formic acid.

### Mass Spectrometry: LC-MS/MS Analysis.

Liquid Chromatography with tandem mass spectrometry (LC-MS/MS) was performed using a Waters NanoAcquity M-class system coupled to a Thermo Scientific Q Exactive HF mass spectrometer. Thermo Scientific EASY-Spray 50-cm × 75-µm ID length C18 columns were used to separate desalted peptides with a 5-30% acetonitrile gradient in 0.1% formic acid over 70 min at a flow rate of 300 nL/min. After each gradient, the column was washed with 90% buffer B (0.1% formic acid, 100% HPLC-grade acetonitrile) for 5 min and reequilibrated with 98% buffer A (0.1% formic acid, 100% HPLC-grade water) for 40 min.

MS data were acquired in data-dependent acquisition mode with an automatic switch between a full scan and 15 data-dependent MS/MS scans (TopN method). The target value for the full-scan MS spectra was 3 × 10^6^ ions in the 375 to 1,600 m/z range with a maximum injection time of 100 ms and resolution of 60,000 at 200 m/z with data collected in the profile mode. Precursors were selected using a 1.6-m/z isolation width. Precursors were fragmented by higher-energy C-trap dissociation with a normalized collision energy of 27 eV. MS/MS scans were acquired at a resolution of 15,000 at 200 m/z with an ion target value of 5 × 10^5^, maximum injection time of 50 ms, dynamic exclusion for 15 s, and data collected in the centroid mode.

### Mass Spectrometry: Data Analysis.

Raw mass spectrometric data were analyzed using MaxQuant (49) v.1.6.1.0 and employed Andromeda for database search 50 at default settings with a few modifications. The default was used for first search tolerance and main search tolerance: 20 ppm and 6 ppm, respectively. MaxQuant was set up to search the reference mouse proteome database downloaded from UniProt. MaxQuant performed the search trypsin digestion with up to 2 missed cleavages. Peptide, Site, and Protein FDR were all set to 1% with a minimum of 2 peptides needed for identification but 2 peptides needed to calculate a protein level ratio. The following modifications were used as fixed carbamidomethyl modification of cysteine, and oxidation of methionine, deamination for asparagine or glutamine (NQ) and acetylation on N-terminal of protein were used as variable modifications. MaxQuant combined folder was uploaded in scaffold for data visualization.

### Quantification and Statistical Analysis.

Specific statistical analyses are indicated in figure legends. Mass spectrometry data analysis is indicated in the appropriate methods section. Graphs represent mean +/− SEM. Statistical analyses were performed using GraphPad Prism 7. All data were analyzed using Student’s *t* test or one-way ANOVA, with Dunn’s multiple comparison tests. Statistical significance was designated as *P* < 0.05.

## Supplementary Material

Appendix 01 (PDF)

Dataset S01 (RTF)

Dataset S02 (RTF)

Dataset S03 (RTF)

Dataset S04 (XLSX)

## Data Availability

Materials are available with an appropriate material transfer agreement (MTA); please contact the Lead Contact. hSyn-tdTomato and aCaMKII-eGFP are not available, due to MTA limitations. The published article includes all datasets generated or analyzed during this study.
