# Supplementary material for: CPEB3 low-complexity motif regulates local protein synthesis via protein–protein interactions in neuronal ribonucleoprotein granules
Source: Proc Natl Acad Sci U S A. 2023 Jan 30;120(6):e2114747120. doi: 10.1073/pnas.2114747120 (PMC9964033; doi:10.1073/pnas.2114747120)
Supplement: Supplementary file 1 — Appendix 01 (PDF) [file pnas.2114747120.sapp.pdf]

Supplementary Figures

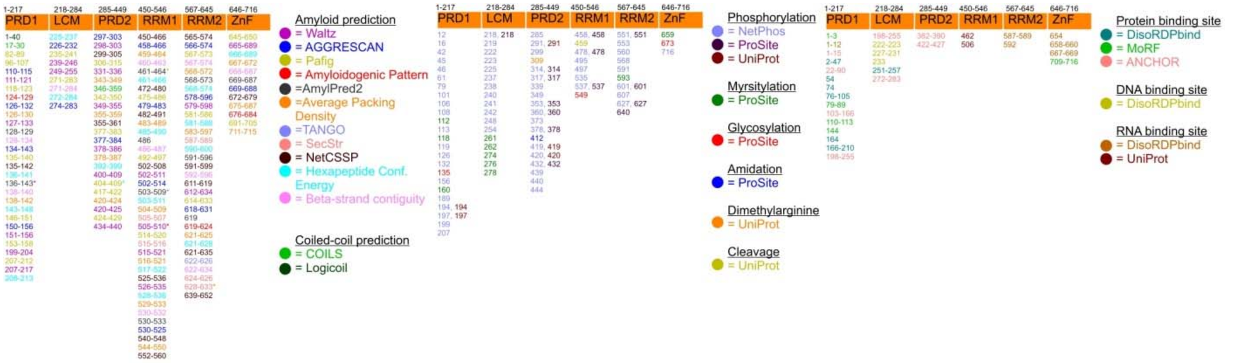

Supp Figure 1. Predictions of super-secondary structures, post-translational modifications, and binding domains of CPEB3. Amyloid prediction algorithms AmylPred2 (Tsolis et al., 2013) (which includes Aggrescan (Conchillo-Sole et al., 2007), AmyloidMutants (O'Donnell et al., 2011), Amyloidogenic Pattern (Lopez de la Paz and Serrano, 2004), Average Packing Density (Galzitskaya and Lobanov, 2006), Beta-strand contiguity (Zibaee et al., 2007), Hexapeptide Conformational Energy (Zhang et al., 2007), NetCSSP (Kim et al., 2009), Pafig (Tian et al., 2009), SecStr (Hamodrakas et al., 2007), Tango (Fernandez-Escamilla et al., 2004), and Watlz (Maurer-Stroh et al., 2010)); and coiled-coil prediction algorithms COILS (Lupas et al., 1991) and LogiCoil (Vincent et al., 2013) were utilized to pinpoint regions of aggregation to expand our focus from areas previously studied (Fiumara et al., 2010; Raveendra et al., 2013; and Stephan et al., 2015). We then included post-translational modifications to our search, which have been identified as important for RNA-binding protein self-association (Nielsen et al., 2015; Calabretta and Richard, 2015; Araki et al., 2015; Monahan et al., 2017; Larson et al., 2017; Ambadipudi et al., 2017). From NetPhos (Blom et al., 1999), UniProt (The UniProt Consortium, 2017), and ProSite (Sigrist et al., 2009), we collected a variety of potential post-translational modification sites with an emphasis on phosphorylation, again expanding our scope from previous work (Theis et al., 2003; Fiumara et al., 2015; Drisaldi et al., 2015; and Kaczmarczyk et al., 2016). Finally, we utilized DisoRDPbind (Peng and Kurgan, 2015), MoRF (Disfani et al., 2012), UniProt (The UniProt Consortium, 2017), and ANCHOR (Meszaros et al., 2009; Dosztanyi et al., 2009) to discriminate areas that bind protein, RNA, and DNA (Figure 1), all of which are believed to be critical components of conformation change and/or RNA-binding function (Zanchetta et al., 2008; Nielsen et al., 2015; Calabretta and Richard, 2015; Stephan et al., 2015; Chen et al., 2016; Hult et al., 2017; Watanabe et al., 2017).

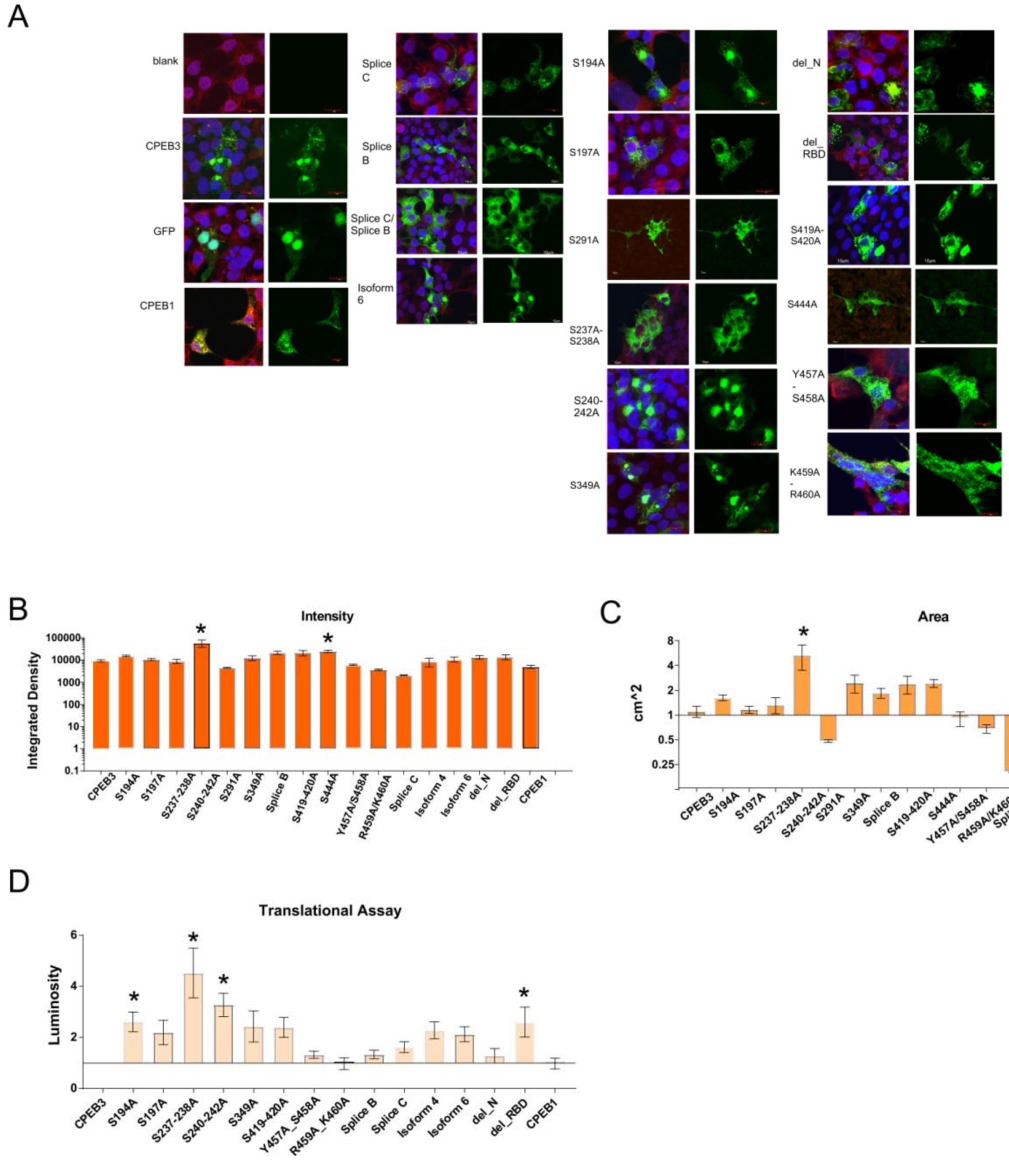

Supp Figure 2. CPEB3 mutants give rise to distinct morphologies and functions. (A) The 16 mutants were created using mutagenesis of wild-type CPEB3-GFP. S194A, S197A, S291A, and S444A were created to disrupt the ability of CPEB3 to be phosphorylated by mutating serine to alanine (Creixell et al., 2012). delN and delRBD were created to delete entire domains that had previously been identified as critical for structure and function (Fioriti et al., 2015; Stephan et al., 2015; Ford et al., 2019), as well as to remove mass spectroscopy-identified modification sites. S237-238A, S240-242A, S349A, S419/420A, Y457A/S458A, and R459A/K460A was created to disrupt sites that are critically situated in areas of predicated structure, protein-binding, and modification, as well as to disrupt S/T/Y repeat sequences, which exhibit high phosphorylation probability in RNA-binding proteins (Monahan et. al, 2017). Finally, isoforms 2b, 3c, 4d, and 6f were created to avoid bias towards overexpressing isoform 1a. Expressed protein (green), G3BP control (red), and DAPI. **(B and C)** Our 16 mutants and full length CPEB3 were expressed in confluent HEK cells and visualized via their GFP tag using fluorescent microscopy. We hypothesized that disrupting regions important for function and/or structure would result in distinct morphologies *in vivo*. To test this we compared both the punctae area of the mutants to full length CPEB3, as well as signal intensity of punctae. We also measured these characteristics on our method

control proteins: non-oligomerizing CPEB1 (Fioriti et al., 2015) and GFP. Critically, we included labeling of G3BP, a protein with high localization to stress granules (Kedersha and Anderson, 2007), to ensure that any CPEB3-GFP morphology wasn't an overexpression artifact. Co-localization of G3BP in red with CPEB3-GFP can be considered an artifact of overexpression, as CPEB3 does not naturally occur in stress granules (Kedersha and Anderson, 2007; Ford et al., 2019), and so all quantitation avoided overlapping fluorescence. The data were analyzed using punctae analysis software SyNPanal (Danielson et al., 2014). Two mutants had a significantly increased intensity of the punctae compared to control CPEB3, S240-242A and S444A. S240-242A was the only mutant to have significantly increased punctae area from control CPEB3. **(D)** Our 16 mutants and full length CPEB3 were expressed in confluent HEK cells along with an actin-3'UTR-Renilla reporter plasmid. Translation of the reporter was measured using a luciferase assay, and luminosity was measured as a readout of translation. S194, S237-238A, S240-242A, and del_RBD mutant were significantly different from CPEB3 levels. Scale bars represent 10 μm. *= p<0.05, as measured with One-Way ANOVA with Dunn's Multiple Comparison. n=10. Error bars = +-SEM.

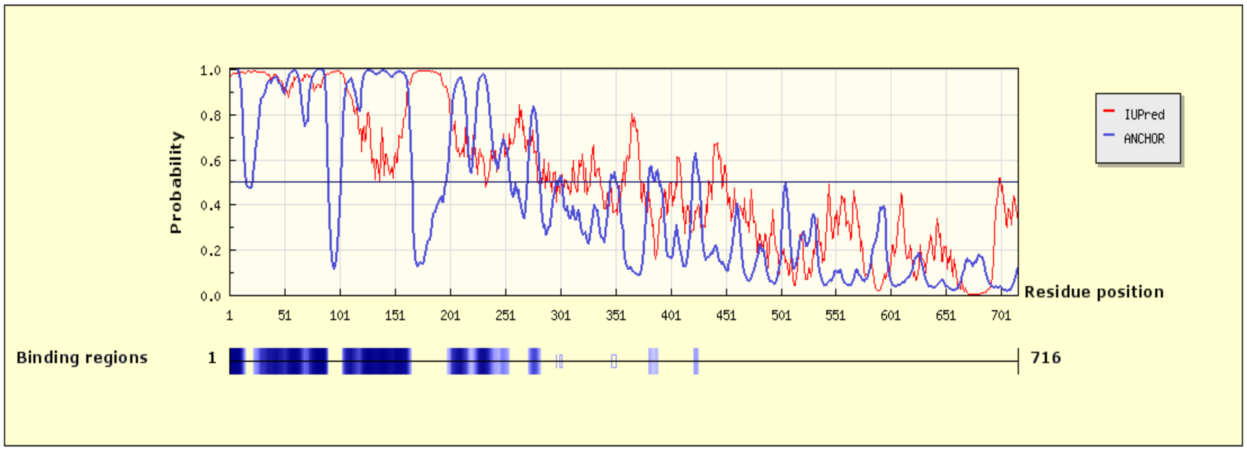

Supp Figure 3. ANCHOR protein-protein interaction analysis of CPEB3

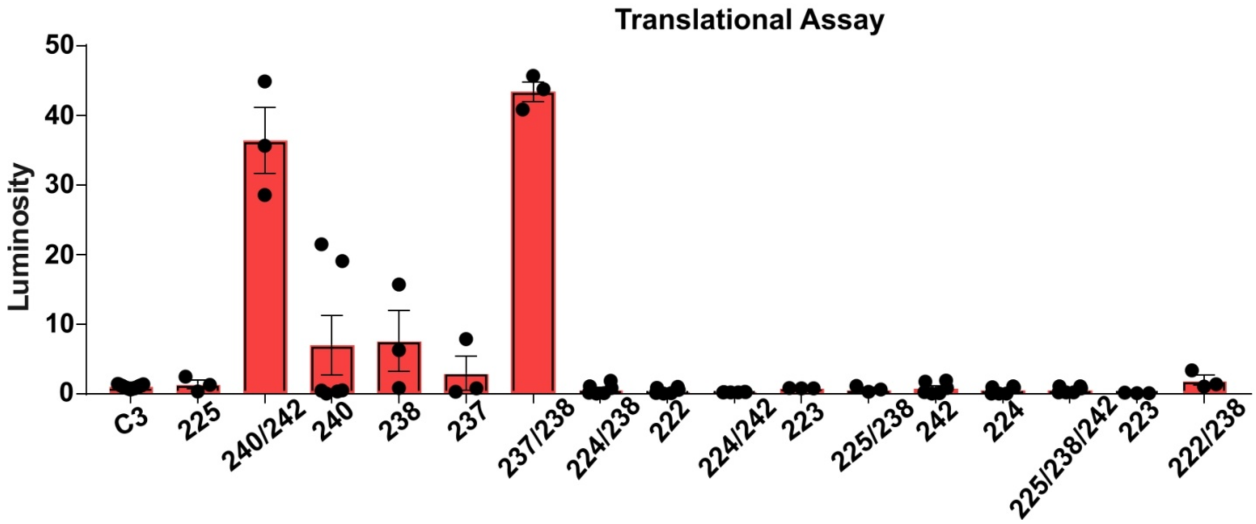

Supp Figure 4. CPEB3 low-complexity domain mutants disrupt inhibitory function. The 16 mutants were created using mutagenesis of wild-type CPEB3-GFP, targeting the serines within the low-complexity domain. Our 16 mutants and full length CPEB3 were expressed in confluent HEK cells along

with an actin-3'UTR-Renilla reporter plasmid. Translation of the reporter was measured using a luciferase assay, and luminosity was measured as a readout of translation. S240-242A, S240A, S238A, S237-238A are significantly different from CPEB3, $p<0.05$, as measured with One-Way ANOVA with Dunn's Multiple Comparison. n=3- 23. Error bars = +-SEM.

Supplementary Tables

Supp Table 1. Detailed Strings data.

Protein ID	B color code	C color code
Magohb	red	blue
Ktn1	red	
Bzw1	red	
Tardbp	red	
Clasp1	blue	green
Naa15	red	
Atp6v1h	red	
Cnn3	red	green
Fxr1	red	green, blue
Eif4e	yellow	cyan, green, blue, red
Ubap2l	red	
Ctbp1	red	
Lrp1	red	
Cyfip1	red	
Cpne2	red	
Dbn1	red	
Tmem33	cyan	
Etfb	red	
Atxn2l	red	cyan, blue, green
Tjp1	red	green
Rpl18a	yellow	yellow, green, blue
Tln2	red	green

Rps25	yellow	yellow, green, blue
Idh3a	cyan	
Chordc1	red	
Rps12	yellow	yellow, green, blue
Atp2b1	red	green
Psmd6	green	
Anxa7	red	
Actn1	red	green
Gatad2b	red	
Kpna2	red	cyan, blue, green
Sept9	red	green
Eif3m	yellow	
Srp68	yellow	green, blue
Rpn2	cyan	
Eif4a3	yellow	blue, green
Hsd17b10	red	green, blue
Agps	red	green
Nckap1	red	green
Rrbp1	red	yellow, green, blue
Dync1i2	blue	
Xpo5	red	green
Clasp2	blue	green
Ywhaq	red	
Acaca	red	green
Acly	yellow	

Actr1a	blue	green
Slc25a4	red	
Ago2	red	cyan, green, blue, red
Ina	red	green, blue, cyan
Rnpep	red	
Apob	red	
Aprt	cyan	
Araf	red	
Arf5	blue	
Arl1	red	
Atrx	red	green
Acadvl	red	green
Macf1	red	green
Lrrc47	cyan	
Prdx1	red	green
Mylk	red	green
Gmps	cyan	
Actb	red	green, blue, cyan
Parp14	red	
Strap	red	
Wdr82	red	green
Cnot1	red	green, blue, cyan, red
Cbr1	red	
Ddx3x	red	blue, cyan, green
Eprs	cyan	blue

Bag6	red	
Mthfd1l	red	
Chd4	red	green
C3	red	
Csk	red	
Cul1	red	
Cul3	red	green
Cul5	red	
Glyr1	red	green
Prdx6	red	
Hspd1	cyan	
Ctnnd1	red	
Dip2b	red	
Ddx6	green	cyan, green, blue, red
Dld	red	
Dpp3	red	
Drg2	red	
Dnah5	blue	green
Nae1	red	
Xpo7	red	
Usp24	red	
Gcc2	red	
Iars2	cyan	
Dock7	red	
Cul4b	red	blue

Npepps	red	
Camk2d	red	
Itsn1	red	
Sec22b	red	
Luc7l2	red	blue
Farp1	red	green
Atp2b4	red	
Scamp3	red	
Cad	red	
Mars	cyan	green
Rab3gap2	blue	
Dhx9	green	blue, cyan, green, yellow
Sacs	red	
Hadha	red	green
Echs1	red	
Eef1g	cyan	
Eef2	yellow	yellow, blue, reen
Etf1	yellow	
Gspt1	yellow	
Hnrnpd	green	blue
Ube2m	green	
Cfl1	red	green
Ddx46	green	green
Rps15a	yellow	yellow, green, blue
Fasn	red	

Flnb	red	green
Fxr2	red	green
Hk1	red	
Gps1	red	
Qrich1	red	
Sec23ip	red	
Supt16	red	green
Mink1	red	
Dnm2	red	green
Sf3b1	green	
Hnrnpl	green	blue, green
Gars	cyan	
Gdi1	red	
Gpc4	red	
Cpsf6	red	cyan, green
Cct8	yellow	green
Hat1	red	green
Hnrnpf	green	blue
Larp7	red	blue
Eif2s2	yellow	
Ilf2	red	green, blue
Ipo11	red	green
Ipo7	cyan	
Ckb	red	
Kif5b	blue	green

Mta1	red	green
Mecp2	red	green
Msn	red	green
Mt2	red	
Mta2	red	green
Mug1	red	
Myh9	red	green
Por	red	
Nsf	red	
rps14	yellow	yellow, green, blue
Pes1	cyan	green, blue
Pgm1	red	
Phb	red	
Pls3	red	green
Prep	red	
Psma4	green	green, blue, cyan, red
Ptpn11	red	
Adsl	cyan	
Atic	yellow	
Ssrp1	red	green
Uchl1	red	
Coro1c	red	green
Pgm3	red	
Pelo	yellow	
Eef1a1	yellow	green

Clic1	red	
Parp1	red	green
Gdi2	red	
Rtcb	cyan	
Vps35	red	
Ola1	cyan	green
Pafah1b1	blue	green
Tnpo1	green	
Map2k1	red	green
G6pdx	red	
Gnb1	red	green
Ap1b1	red	
Psmd2	green	
Dnajb1	red	green
Hspa4	green	green
Cct3	cyan	green
Ugp2	red	
Ppia	red	
Mcm6	red	
Ddost	red	
Sptbn2	blue	
Myh10	red	green, blue
Ugdh	red	
Mcm3	red	green
Stat3	red	green

Actn	red	
Tubb6	blue	green
Gna11	red	
Ruvbl2	red	green, blue
Eif2b1	red	
Rpl30	yellow	yellow, green, blue
Wars	cyan	
Arpc2	red	green
Rpl21	yellow	yellow, green, blue
Rpl22	yellow	yellow, green, blue
Tuba1c	blue	green
Kpna2	red	cyan
Alb	red	
Ube2l3	red	
Psmc4	green	
Hnrnpm	green	blue
Rpl7a	yellow	yellow, green, blue
Rpl5	yellow	yellow, green, blue
Matr3	red	
Rps6	yellow	cyan, yellow, green, blue
Rps16	yellow	yellow, green, blue
Ube3a	red	
Hnrnpa1	green	blue
Aldoa	red	green
Vim	red	green, blue

Rpl9	yellow	yellow, green, blue
Rpl28	yellow	yellow, cyan, blue, green
Rps8	yellow	yellow, green, blue
Abcf1	cyan	yellow, blue, green
Actr2	red	green
Ap2b1	red	
Prpsap2	cyan	yellow, green, blue
Rpl10a	yellow	yellow, green, blue
Copa	blue	
Eif3a	yellow	blue, green
Ap2m1	red	
Eif3f	yellow	
Abce1	yellow	
Snx1	red	
Rcc1	red	green
Rpl17	yellow	yellow, green, blue
Aars	cyan	
Iqgap1	red	green, blue, cyan
Hsp90ab1	blue	
Rangap1	blue	green
Prmt1	red	
Nup155	red	
Pdcd6ip	red	green
H2afy2	red	green
Nap1l1	red	

Vamp4	red	
Elavl1	green	green, cyan, blue
Eif4a2	yellow	green
Psmc2	green	red, green, blue, cyan
Kars	cyan	
Klc1	blue	green
Eif3b	yellow	
Qars	cyan	
Abcd3	red	
Snx2	blue	
Ddb1	red	
Anxa6	red	
Tubb4b	blue	green
Anxa2	red	green
Mtch2	red	
Arpc4	red	green
Dynll1	blue	green
Uqcrc1	red	
Rcc2	blue	green
Upf1	yellow	cyan, red, blue, green
Rpl11	yellow	yellow, green, blue
Rpl32	yellow	yellow, green, blue
Rpl4	yellow	yellow, green, blue
Rpn1	cyan	
Rps11	yellow	yellow, green, blue

Rps17	yellow	yellow, green, blue
Rps20	yellow	yellow, green, blue
Rps27	yellow	yellow, green, blue
Rps9	yellow	yellow, green, blue
Xpnpep1	red	
Ank2	blue	green
Sae1	cyan	
Sec31a	green	
Sdha	red	green
Sfpq	green	green
Snx27	red	
Ubr4	red	green
Gls	cyan	
Gcn1	red	blue
