# Supplementary material for: CPEB3 low-complexity motif regulates local protein synthesis via protein–protein interactions in neuronal ribonucleoprotein granules
Source: Proc Natl Acad Sci U S A. 2023 Jan 30;120(6):e2114747120. doi: 10.1073/pnas.2114747120 (PMC9964033; doi:10.1073/pnas.2114747120)
Supplement: Supplementary file 2 — Dataset S01 (RTF) [file pnas.2114747120.sd01.rtf]

Supplementary Data 1. Proteomics results, HEK
Q3TKA2_MOUSE	Uncharacterized protein OS=Mus musculus OX=10090 GN=Hsp90aa1 PE=2 SV=1	
NP_001277756.1 cytoplasmic polyadenylation element-binding protein 3 isoform a [Mus...	cytoplasmic polyadenylation element-binding protein 3 isoform a [Mus musculus]	
Q5SXR6_MOUSE	Clathrin heavy chain OS=Mus musculus OX=10090 GN=Cltc PE=1 SV=1	
Q5FWJ3_MOUSE	Vimentin OS=Mus musculus OX=10090 GN=Vim PE=1 SV=1	
EF2_MOUSE	Elongation factor 2 OS=Mus musculus OX=10090 GN=Eef2 PE=1 SV=2	
Q3TQ13_MOUSE	Uncharacterized protein OS=Mus musculus OX=10090 GN=Hspa8 PE=2 SV=1	
Q52L87_MOUSE	Tubulin alpha chain OS=Mus musculus OX=10090 GN=Tuba1c PE=1 SV=1	
sp|P52480|KPYM_MOUSE	Pyruvate kinase PKM OS=Mus musculus OX=10090 GN=Pkm PE=1 SV=4	
Q3TWN5_MOUSE	Uncharacterized protein OS=Mus musculus OX=10090 GN=Hnrnpu PE=2 SV=1	
Q3UH59_MOUSE	Myosin-10 OS=Mus musculus OX=10090 GN=Myh10 PE=1 SV=1	
sp|Q8BTM8|FLNA_MOUSE	Filamin-A OS=Mus musculus OX=10090 GN=Flna PE=1 SV=5	
TLN1_MOUSE	Talin-1 OS=Mus musculus OX=10090 GN=Tln1 PE=1 SV=2	
Q3TII3_MOUSE	Elongation factor 1-alpha OS=Mus musculus OX=10090 GN=Eef1a1 PE=2 SV=1	
ACLY_MOUSE	ATP-citrate synthase OS=Mus musculus OX=10090 GN=Acly PE=1 SV=1	
G5E866_MOUSE	Splicing factor 3B subunit 1 OS=Mus musculus OX=10090 GN=Sf3b1 PE=1 SV=1	
Q6A0B9_MOUSE	Eukaryotic translation initiation factor 3 subunit A (Fragment) OS=Mus musculus OX=10090 GN=Eif3a PE=2 SV=1	
Q3TFG3_MOUSE	Uncharacterized protein OS=Mus musculus OX=10090 GN=Eif4a1 PE=2 SV=1	
Q570Z0_MOUSE	MKIAA4193 protein (Fragment) OS=Mus musculus OX=10090 GN=Hnrnpm PE=2 SV=1	
Q8BNF8_MOUSE	Uncharacterized protein OS=Mus musculus OX=10090 GN=Vcp PE=2 SV=1	
XPO2_MOUSE	Exportin-2 OS=Mus musculus OX=10090 GN=Cse1l PE=1 SV=1	
Q63ZW9_MOUSE	Copa protein (Fragment) OS=Mus musculus OX=10090 GN=Copa PE=2 SV=1	
E9Q8K5_MOUSE	Titin OS=Mus musculus OX=10090 GN=Ttn PE=1 SV=1	
Q3TIV3_MOUSE	Poly [ADP-ribose] polymerase OS=Mus musculus OX=10090 GN=Parp1 PE=2 SV=1	
Q3U4U6_MOUSE	T-complex protein 1 subunit gamma OS=Mus musculus OX=10090 GN=Cct3 PE=1 SV=1	
tr|A1E2B8|A1E2B8_MOUSE Inducible heat shock protein 70 OS=Mus musculus OX=10090 PE=3...	Inducible heat shock protein 70 OS=Mus musculus OX=10090 PE=3 SV=1	
G3X956_MOUSE	Suppressor of Ty 16 OS=Mus musculus OX=10090 GN=Supt16 PE=1 SV=1	
CHD4_MOUSE	Chromodomain-helicase-DNA-binding protein 4 OS=Mus musculus OX=10090 GN=Chd4 PE=1 SV=1	
MYH9_MOUSE	Myosin-9 OS=Mus musculus OX=10090 GN=Myh9 PE=1 SV=4	
sp|Q7TMY8-3|HUWE1_MOUSE	Isoform 3 of E3 ubiquitin-protein ligase HUWE1 OS=Mus musculus OX=10090 GN=Huwe1	
Q5BL18_MOUSE	Matrin 3 OS=Mus musculus OX=10090 GN=Matr3 PE=1 SV=1	
RL4_MOUSE	60S ribosomal protein L4 OS=Mus musculus OX=10090 GN=Rpl4 PE=1 SV=3	
sp|P11983|TCPA_MOUSE	T-complex protein 1 subunit alpha OS=Mus musculus OX=10090 GN=Tcp1 PE=1 SV=3	
sp|P38647|GRP75_MOUSEStress-70 protein, mitochondrial OS=Mus musculus OX=10090 GN=Hspa9 PE=1 SV=3	sp|P38647|GRP75_MOUSE Stress-70 protein, mitochondrial OS=Mus musculus OX=10090 GN=Hspa9 PE=1 SV=3	
tr|Q80U36|Q80U36_MOUSE MKIAA0325 protein (Fragment) OS=Mus musculus OX=10090 GN=Dync1h1...	MKIAA0325 protein (Fragment) OS=Mus musculus OX=10090 GN=Dync1h1 PE=2 SV=1	
Q6ZQK2_MOUSE	MKIAA0051 protein (Fragment) OS=Mus musculus OX=10090 GN=Iqgap1 PE=2 SV=1	
tr|Q3TWF2|Q3TWF2_MOUSE Uncharacterized protein OS=Mus musculus OX=10090 GN=Hspa5...	Uncharacterized protein OS=Mus musculus OX=10090 GN=Hspa5 PE=2 SV=1	
H3BL49_MOUSE	T-complex protein 1 subunit theta OS=Mus musculus OX=10090 GN=Cct8 PE=1 SV=1	
sp|Q61656|DDX5_MOUSE Probable ATP-dependent RNA helicase DDX5 OS=Mus musculus OX=10090...	Probable ATP-dependent RNA helicase DDX5 OS=Mus musculus OX=10090 GN=Ddx5 PE=1 SV=2	
sp|Q9Z1X4-3|ILF3_MOUSE	Isoform 3 of Interleukin enhancer-binding factor 3 OS=Mus musculus OX=10090 GN=Ilf3	
PLST_MOUSE	Plastin-3 OS=Mus musculus OX=10090 GN=Pls3 PE=1 SV=3	
Q3TMY8_MOUSE	Uncharacterized protein OS=Mus musculus OX=10090 GN=Eftud2 PE=2 SV=1	
Q5DTS8_MOUSE	DNA helicase (Fragment) OS=Mus musculus OX=10090 GN=Mcm4 PE=2 SV=1	
tr|E9Q8K5|E9Q8K5_MOUSE-DECOY Titin OS=Mus musculus OX=10090 GN=Ttn PE=1 SV=1	tr|E9Q8K5|E9Q8K5_MOUSE-DECOY Titin OS=Mus musculus OX=10090 GN=Ttn PE=1 SV=1	
Q3THH8_MOUSE	Uncharacterized protein OS=Mus musculus OX=10090 GN=Cct7 PE=2 SV=1	
tr|Q3TFE5|Q3TFE5_MOUSE Uncharacterized protein OS=Mus musculus OX=10090 GN=Dhx15...	Uncharacterized protein OS=Mus musculus OX=10090 GN=Dhx15 PE=2 SV=1	
tr|Q3UAD6|Q3UAD6_MOUSE Heat shock protein 90kDa beta (Grp94), member 1 OS=Mus musculus...	Heat shock protein 90kDa beta (Grp94), member 1 OS=Mus musculus OX=10090 GN=Hsp90b1 PE=1 SV=1	
Q3UJN1_MOUSE	DNA helicase OS=Mus musculus OX=10090 GN=Mcm2 PE=2 SV=1	
Q3ULD6_MOUSE	DNA helicase OS=Mus musculus OX=10090 GN=Mcm3 PE=2 SV=1	
sp|Q03265|ATPA_MOUSE ATP synthase subunit alpha, mitochondrial OS=Mus musculus OX=10090...	ATP synthase subunit alpha, mitochondrial OS=Mus musculus OX=10090 GN=Atp5f1a PE=1 SV=1	
RENT1_MOUSE	Isoform 2 of Regulator of nonsense transcripts 1 OS=Mus musculus OX=10090 GN=Upf1	
G3X9G4_MOUSE	Dynamin-2 OS=Mus musculus OX=10090 GN=Dnm2 PE=1 SV=1	
Q3TIX8_MOUSE	Uncharacterized protein OS=Mus musculus OX=10090 GN=Cct6a PE=2 SV=1	
VINC_MOUSE	Vinculin OS=Mus musculus OX=10090 GN=Vcl PE=1 SV=4	
sp|E9PVA8|GCN1_MOUSE eIF-2-alpha kinase activator GCN1 OS=Mus musculus OX=10090 GN=Gcn1...	eIF-2-alpha kinase activator GCN1 OS=Mus musculus OX=10090 GN=Gcn1 PE=1 SV=1	
sp|P09411|PGK1_MOUSE Phosphoglycerate kinase 1 OS=Mus musculus OX=10090 GN=Pgk1 PE=1...	sp|P09411|PGK1_MOUSE Phosphoglycerate kinase 1 OS=Mus musculus OX=10090 GN=Pgk1 PE=1...	
Q3UB15_MOUSE	Uncharacterized protein OS=Mus musculus OX=10090 GN=Rpl3 PE=2 SV=1	
XPO1_MOUSE	Exportin-1 OS=Mus musculus OX=10090 GN=Xpo1 PE=1 SV=1	
sp|Q91WQ3|SYYC_MOUSE Tyrosine--tRNA ligase, cytoplasmic OS=Mus musculus OX=10090...	sp|Q91WQ3|SYYC_MOUSE Tyrosine--tRNA ligase, cytoplasmic OS=Mus musculus OX=10090...	
E9Q039_MOUSE	Aminopeptidase OS=Mus musculus OX=10090 GN=Npepps PE=1 SV=1	
Q3UGW8_MOUSE	Uncharacterized protein (Fragment) OS=Mus musculus OX=10090 GN=Aldh18a1 PE=2 SV=1	
tr|Q7TSZ6|Q7TSZ6_MOUSE Karyopherin (Importin) beta 1 OS=Mus musculus OX=10090 GN=Kpnb1...	tr|Q7TSZ6|Q7TSZ6_MOUSE Karyopherin (Importin) beta 1 OS=Mus musculus OX=10090 GN=Kpnb1...	
FLNB_MOUSE	Filamin-B OS=Mus musculus OX=10090 GN=Flnb PE=1 SV=3	
sp|Q6ZQ38|CAND1_MOUSECullin-associated NEDD8-dissociated protein 1 OS=Mus musculus OX=10090 GN=Cand1 PE=1 SV=2	sp|Q6ZQ38|CAND1_MOUSE Cullin-associated NEDD8-dissociated protein 1 OS=Mus musculus OX=10090 GN=Cand1 PE=1 SV=2	
Q3TFC2_MOUSE	Uncharacterized protein OS=Mus musculus OX=10090 GN=Nono PE=2 SV=1	
Q80Y09_MOUSE	Pdcd6ip protein OS=Mus musculus OX=10090 GN=Pdcd6ip PE=2 SV=1	
tr|Q3TJ38|Q3TJ38_MOUSE Uncharacterized protein OS=Mus musculus OX=10090 GN=Hnrnpk...	tr|Q3TJ38|Q3TJ38_MOUSE Uncharacterized protein OS=Mus musculus OX=10090 GN=Hnrnpk...	
tr|Q811H6|Q811H6_MOUSE Hsp90ab1 protein (Fragment) OS=Mus musculus OX=10090 GN=Hsp90ab1...	tr|Q811H6|Q811H6_MOUSE Hsp90ab1 protein (Fragment) OS=Mus musculus OX=10090 GN=Hsp90ab1...	
Q3TW51_MOUSE	Uncharacterized protein OS=Mus musculus OX=10090 GN=Snd1 PE=2 SV=1	
Q3TWL6_MOUSE	26S proteasome non-ATPase regulatory subunit 2 OS=Mus musculus OX=10090 GN=Psmd2 PE=2 SV=1	
tr|Q3V1M8|Q3V1M8_MOUSE Uncharacterized protein OS=Mus musculus OX=10090 GN=Hdlbp...	Uncharacterized protein OS=Mus musculus OX=10090 GN=Hdlbp PE=2 SV=1	
tr|Q542X7|Q542X7_MOUSE Chaperonin subunit 2 (Beta), isoform CRA_a OS=Mus musculus...	Chaperonin subunit 2 (Beta), isoform CRA_a OS=Mus musculus OX=10090 GN=Cct2 PE=1 SV=1	
A0A1S6GWH2_MOUSE	Uncharacterized protein OS=Mus musculus OX=10090 GN=Ddx39b PE=2 SV=1	
B7ZWF1_MOUSE	Ddx3x protein OS=Mus musculus OX=10090 GN=Ddx3x PE=2 SV=1	
Q3UC10_MOUSE	DNA helicase OS=Mus musculus OX=10090 GN=Mcm6 PE=2 SV=1	
Q52KC3_MOUSE	DNA helicase OS=Mus musculus OX=10090 GN=Mcm5 PE=1 SV=1	
sp|Q62261-2|SPTB2_MOUSE Isoform 2 of Spectrin beta chain, non-erythrocytic 1 OS=Mus...	Isoform 2 of Spectrin beta chain, non-erythrocytic 1 OS=Mus musculus OX=10090 GN=Sptbn1	
tr|Q3UIG0|Q3UIG0_MOUSE Eukaryotic translation initiation factor 3 subunit E OS=Mus...	tr|Q3UIG0|Q3UIG0_MOUSE Eukaryotic translation initiation factor 3 subunit E OS=Mus...	
tr|Q8C2Q7|Q8C2Q7_MOUSE Heterogeneous nuclear ribonucleoprotein H OS=Mus musculus...	Heterogeneous nuclear ribonucleoprotein H OS=Mus musculus OX=10090 GN=Hnrnph1 PE=1 SV=1	
E9QAI5_MOUSE	CAD protein OS=Mus musculus OX=10090 GN=Cad PE=1 SV=1	
NSF_MOUSE	Vesicle-fusing ATPase OS=Mus musculus OX=10090 GN=Nsf PE=1 SV=2	
tr|Q3UJN2|Q3UJN2_MOUSE RuvB-like helicase OS=Mus musculus OX=10090 GN=Ruvbl1 PE=2...	RuvB-like helicase OS=Mus musculus OX=10090 GN=Ruvbl1 PE=2 SV=1	
B9EIU1_MOUSE	Glutamyl-prolyl-tRNA synthetase OS=Mus musculus OX=10090 GN=Eprs PE=2 SV=1	
Q05DR5_MOUSE	FACT complex subunit SSRP1 (Fragment) OS=Mus musculus OX=10090 GN=Ssrp1 PE=2 SV=1	
SFPQ_MOUSE	Splicing factor, proline- and glutamine-rich OS=Mus musculus OX=10090 GN=Sfpq PE=1 SV=1	
sp|P16125|LDHB_MOUSE L-lactate dehydrogenase B chain OS=Mus musculus OX=10090 GN=Ldhb...	L-lactate dehydrogenase B chain OS=Mus musculus OX=10090 GN=Ldhb PE=1 SV=2	
Q32P04_MOUSE	Keratin 5 OS=Mus musculus OX=10090 GN=Krt5 PE=1 SV=2	
tr|Q3TXF9|Q3TXF9_MOUSE Sodium/potassium-transporting ATPase subunit alpha OS=Mus...	Sodium/potassium-transporting ATPase subunit alpha OS=Mus musculus OX=10090 GN=Atp1a1 PE=2 SV=1	
tr|Q3UZH3|Q3UZH3_MOUSE Uncharacterized protein (Fragment) OS=Mus musculus OX=10090...	Uncharacterized protein (Fragment) OS=Mus musculus OX=10090 GN=Mthfd1 PE=2 SV=1	
sp|Q8BKC5|IPO5_MOUSE	Importin-5 OS=Mus musculus OX=10090 GN=Ipo5 PE=1 SV=3	
Q545A2_MOUSE	MCG11560 OS=Mus musculus OX=10090 GN=Slc25a5 PE=1 SV=1	
sp|Q61753|SERA_MOUSE D-3-phosphoglycerate dehydrogenase OS=Mus musculus OX=10090...	sp|Q61753|SERA_MOUSE D-3-phosphoglycerate dehydrogenase OS=Mus musculus OX=10090...	
Q3U4D1_MOUSE	Adenosylhomocysteinase OS=Mus musculus OX=10090 PE=2 SV=1	
sp|Q8QZY1|EIF3L_MOUSEEukaryotic translation initiation factor 3 subunit L OS=Mus musculus OX=10090 GN=Eif3l PE=1 SV=1	sp|Q8QZY1|EIF3L_MOUSE Eukaryotic translation initiation factor 3 subunit L OS=Mus musculus OX=10090 GN=Eif3l PE=1 SV=1	
sp|Q8BFR5|EFTU_MOUSE Elongation factor Tu, mitochondrial OS=Mus musculus OX=10090...	sp|Q8BFR5|EFTU_MOUSE Elongation factor Tu, mitochondrial OS=Mus musculus OX=10090...	
FAS_MOUSE	Fatty acid synthase OS=Mus musculus OX=10090 GN=Fasn PE=1 SV=2	
Q5EBP8_MOUSE	Heterogeneous nuclear ribonucleoprotein A1 OS=Mus musculus OX=10090 GN=Hnrnpa1 PE=1 SV=1	
tr|B2RSV4|B2RSV4_MOUSE Splicing factor 3b, subunit 3 OS=Mus musculus OX=10090 GN=Sf3b3...	tr|B2RSV4|B2RSV4_MOUSE Splicing factor 3b, subunit 3 OS=Mus musculus OX=10090 GN=Sf3b3...	
CUL1_MOUSE	Cullin-1 OS=Mus musculus OX=10090 GN=Cul1 PE=1 SV=1	
Q5SWR1_MOUSE	AP complex subunit beta OS=Mus musculus OX=10090 GN=Ap2b1 PE=2 SV=1	
Q8BVQ9_MOUSE	26S proteasome regulatory subunit 7 OS=Mus musculus OX=10090 GN=Psmc2 PE=1 SV=1	
sp|Q8VHG2-2|AMOT_MOUSE	Isoform 2 of Angiomotin OS=Mus musculus OX=10090 GN=Amot	
tr|A0JLM6|A0JLM6_MOUSE Smc1a protein (Fragment) OS=Mus musculus OX=10090 GN=Smc1a...	tr|A0JLM6|A0JLM6_MOUSE Smc1a protein (Fragment) OS=Mus musculus OX=10090 GN=Smc1a...	
tr|Q3U8U8|Q3U8U8_MOUSE Polyadenylate-binding protein OS=Mus musculus OX=10090 GN=Pabpc1...	tr|Q3U8U8|Q3U8U8_MOUSE Polyadenylate-binding protein OS=Mus musculus OX=10090 GN=Pabpc1...	
tr|Q6PCP0|Q6PCP0_MOUSE DEAD (Asp-Glu-Ala-Asp) box polypeptide 21 OS=Mus musculus...	tr|Q6PCP0|Q6PCP0_MOUSE DEAD (Asp-Glu-Ala-Asp) box polypeptide 21 OS=Mus musculus...	
E9QNN1_MOUSE	ATP-dependent RNA helicase A OS=Mus musculus OX=10090 GN=Dhx9 PE=1 SV=1	
tr|Q3UXQ6|Q3UXQ6_MOUSE 40S ribosomal protein S4 OS=Mus musculus OX=10090 GN=Rps4x...	40S ribosomal protein S4 OS=Mus musculus OX=10090 GN=Rps4x PE=2 SV=1	
ILF2_MOUSE	Interleukin enhancer-binding factor 2 OS=Mus musculus OX=10090 GN=Ilf2 PE=1 SV=1	
Q58ET1_MOUSE	MCG11348 OS=Mus musculus OX=10090 GN=Rpl7a PE=1 SV=1	
B2RRX1_MOUSE	Actin, beta OS=Mus musculus OX=10090 GN=Actb PE=2 SV=1	
Q5BLK1_MOUSE	40S ribosomal protein S6 OS=Mus musculus OX=10090 GN=Rps6 PE=1 SV=1	
Q6NXX7_MOUSE	Abce1 protein (Fragment) OS=Mus musculus OX=10090 GN=Abce1 PE=2 SV=1	
sp|P14733|LMNB1_MOUSELamin-B1 OS=Mus musculus OX=10090 GN=Lmnb1 PE=1 SV=3	sp|P14733|LMNB1_MOUSE Lamin-B1 OS=Mus musculus OX=10090 GN=Lmnb1 PE=1 SV=3	
sp|Q61699-2|HS105_MOUSE Isoform HSP105-beta of Heat shock protein 105 kDa OS=Mus...	sp|Q61699-2|HS105_MOUSE Isoform HSP105-beta of Heat shock protein 105 kDa OS=Mus...	
sp|Q8BJ71|NUP93_MOUSENuclear pore complex protein Nup93 OS=Mus musculus OX=10090 GN=Nup93 PE=1 SV=1	sp|Q8BJ71|NUP93_MOUSE Nuclear pore complex protein Nup93 OS=Mus musculus OX=10090 GN=Nup93 PE=1 SV=1	
sp|Q8CAQ8-5|MIC60_MOUSE Isoform 5 of MICOS complex subunit Mic60 OS=Mus musculus...	sp|Q8CAQ8-5|MIC60_MOUSE Isoform 5 of MICOS complex subunit Mic60 OS=Mus musculus...	
sp|Q9ET01|PYGL_MOUSE Glycogen phosphorylase, liver form OS=Mus musculus OX=10090...	Glycogen phosphorylase, liver form OS=Mus musculus OX=10090 GN=Pygl PE=1 SV=4	
tr|A0A0H3XWX3|A0A0H3XWX3_MOUSE Insulin-like growth factor 2 mRNA binding protein...	Insulin-like growth factor 2 mRNA binding protein 1 dN CRDBP-2 OS=Mus musculus OX=10090 GN=Igf2bp1 PE=2 SV=1	
tr|Q3UAC2|Q3UAC2_MOUSE 40S ribosomal protein S3a OS=Mus musculus OX=10090 GN=Rps3a1...	40S ribosomal protein S3a OS=Mus musculus OX=10090 GN=Rps3a1 PE=2 SV=1	
Q3U4P3_MOUSE	Uncharacterized protein (Fragment) OS=Mus musculus OX=10090 GN=Dnm1l PE=2 SV=1	
sp|P62196|PRS8_MOUSE 26S proteasome regulatory subunit 8 OS=Mus musculus OX=10090...	26S proteasome regulatory subunit 8 OS=Mus musculus OX=10090 GN=Psmc5 PE=1 SV=1	
tr|B2RRH9|B2RRH9_MOUSE Guanine monphosphate synthetase OS=Mus musculus OX=10090 GN=Gmps...	Guanine monphosphate synthetase OS=Mus musculus OX=10090 GN=Gmps PE=1 SV=1	
Q9Z1R9_MOUSE	MCG124046 OS=Mus musculus OX=10090 GN=Prss1 PE=1 SV=1	
A0A0A6YW80_MOUSE	N-alpha-acetyltransferase 15, NatA auxiliary subunit OS=Mus musculus OX=10090 GN=Naa15 PE=1 SV=1	
sp|Q3U0V1|FUBP2_MOUSEFar upstream element-binding protein 2 OS=Mus musculus OX=10090 GN=Khsrp PE=1 SV=2	sp|Q3U0V1|FUBP2_MOUSE Far upstream element-binding protein 2 OS=Mus musculus OX=10090 GN=Khsrp PE=1 SV=2	
sp|Q8K224|NAT10_MOUSERNA cytidine acetyltransferase OS=Mus musculus OX=10090 GN=Nat10 PE=1 SV=1	sp|Q8K224|NAT10_MOUSE RNA cytidine acetyltransferase OS=Mus musculus OX=10090 GN=Nat10 PE=1 SV=1	
sp|Q8R1B4|EIF3C_MOUSEEukaryotic translation initiation factor 3 subunit C OS=Mus musculus OX=10090 GN=Eif3c PE=1 SV=1	sp|Q8R1B4|EIF3C_MOUSE Eukaryotic translation initiation factor 3 subunit C OS=Mus musculus OX=10090 GN=Eif3c PE=1 SV=1	
sp|Q9D0R2|SYTC_MOUSE Threonine--tRNA ligase, cytoplasmic OS=Mus musculus OX=10090...	Threonine--tRNA ligase, cytoplasmic OS=Mus musculus OX=10090 GN=Tars PE=1 SV=2	
sp|Q9DBR1|XRN2_MOUSE	5'-3' exoribonuclease 2 OS=Mus musculus OX=10090 GN=Xrn2 PE=1 SV=1	
sp|Q9JIF7|COPB_MOUSE Coatomer subunit beta OS=Mus musculus OX=10090 GN=Copb1 PE=1...	sp|Q9JIF7|COPB_MOUSE Coatomer subunit beta OS=Mus musculus OX=10090 GN=Copb1 PE=1...	
tr|E9Q3M3|E9Q3M3_MOUSE Dynactin subunit 1 OS=Mus musculus OX=10090 GN=Dctn1 PE=1...	Dynactin subunit 1 OS=Mus musculus OX=10090 GN=Dctn1 PE=1 SV=1	
E9QKZ2_MOUSE	Importin-9 OS=Mus musculus OX=10090 GN=Ipo9 PE=1 SV=1	
Q3TK29_MOUSE	Uncharacterized protein OS=Mus musculus OX=10090 GN=Trap1 PE=2 SV=1	
Q3U2G2_MOUSE	Heat shock 70 kDa protein 4 OS=Mus musculus OX=10090 GN=Hspa4 PE=1 SV=1	
Q3ULS2_MOUSE	Structural maintenance of chromosomes protein OS=Mus musculus OX=10090 GN=Smc2 PE=2 SV=1	
sp|P68040|RACK1_MOUSEReceptor of activated protein C kinase 1 OS=Mus musculus OX=10090 GN=Rack1 PE=1 SV=3	sp|P68040|RACK1_MOUSE Receptor of activated protein C kinase 1 OS=Mus musculus OX=10090 GN=Rack1 PE=1 SV=3	
sp|Q02053|UBA1_MOUSE Ubiquitin-like modifier-activating enzyme 1 OS=Mus musculus...	sp|Q02053|UBA1_MOUSE Ubiquitin-like modifier-activating enzyme 1 OS=Mus musculus...	
tr|B2M1R7|B2M1R7_MOUSE Poly(RC) binding protein 2 OS=Mus musculus OX=10090 GN=Pcbp2...	tr|B2M1R7|B2M1R7_MOUSE Poly(RC) binding protein 2 OS=Mus musculus OX=10090 GN=Pcbp2...	
tr|E9Q1S3|E9Q1S3_MOUSE Protein transport protein Sec23A OS=Mus musculus OX=10090...	Protein transport protein Sec23A OS=Mus musculus OX=10090 GN=Sec23a PE=1 SV=1	
tr|Q5YLW3|Q5YLW3_MOUSE Ribosomal protein S3 OS=Mus musculus OX=10090 GN=Rps3 PE=1...	tr|Q5YLW3|Q5YLW3_MOUSE Ribosomal protein S3 OS=Mus musculus OX=10090 GN=Rps3 PE=1...	
MTA2_MOUSE	Metastasis-associated protein MTA2 OS=Mus musculus OX=10090 GN=Mta2 PE=1 SV=1	
Q3U1S6_MOUSE	Uncharacterized protein OS=Mus musculus OX=10090 GN=Csde1 PE=1 SV=1	
Q8CIJ3_MOUSE	Eukaryotic translation initiation factor 3 subunit B OS=Mus musculus OX=10090 GN=Eif3b PE=2 SV=1	
TOP1_MOUSE	DNA topoisomerase 1 OS=Mus musculus OX=10090 GN=Top1 PE=1 SV=2	
tr|A1A4T2|A1A4T2_MOUSE Alpha glucosidase 2 alpha neutral subunit OS=Mus musculus...	tr|A1A4T2|A1A4T2_MOUSE Alpha glucosidase 2 alpha neutral subunit OS=Mus musculus...	
E9PV04_MOUSE	Predicted gene 8994 OS=Mus musculus OX=10090 GN=Gm8994 PE=3 SV=2	
MOES_MOUSE	Moesin OS=Mus musculus OX=10090 GN=Msn PE=1 SV=3	
Q3TH47_MOUSE	Uncharacterized protein OS=Mus musculus OX=10090 GN=Ubc PE=2 SV=1	
Q3TKG4_MOUSE	Uncharacterized protein (Fragment) OS=Mus musculus OX=10090 GN=Psmc3 PE=2 SV=1	
Q546G4_MOUSE	Albumin 1 OS=Mus musculus OX=10090 GN=Alb PE=1 SV=1	
Q58EU6_MOUSE	MCG13589 OS=Mus musculus OX=10090 GN=Rpl5 PE=1 SV=1	
SYRC_MOUSE	Arginine--tRNA ligase, cytoplasmic OS=Mus musculus OX=10090 GN=Rars PE=1 SV=2	
sp|Q76MZ3|2AAA_MOUSE Serine/threonine-protein phosphatase 2A 65 kDa regulatory subunit...	sp|Q76MZ3|2AAA_MOUSE Serine/threonine-protein phosphatase 2A 65 kDa regulatory subunit...	
tr|E9PVC5|E9PVC5_MOUSE Eukaryotic translation initiation factor 4 gamma 1 OS=Mus...	Eukaryotic translation initiation factor 4 gamma 1 OS=Mus musculus OX=10090 GN=Eif4g1 PE=1 SV=1	
E9PXY1_MOUSE	Cullin-4B OS=Mus musculus OX=10090 GN=Cul4b PE=1 SV=2	
EF1G_MOUSE	Elongation factor 1-gamma OS=Mus musculus OX=10090 GN=Eef1g PE=1 SV=3	
KINH_MOUSE	Kinesin-1 heavy chain OS=Mus musculus OX=10090 GN=Kif5b PE=1 SV=3	
Q5XJF6_MOUSE	Ribosomal protein OS=Mus musculus OX=10090 GN=Rpl10a PE=1 SV=1	
Q71LX8_MOUSE	Heat shock protein 84b OS=Mus musculus OX=10090 GN=Hsp90ab1 PE=1 SV=1	
sp|Q91YR7|PRP6_MOUSE	Pre-mRNA-processing factor 6 OS=Mus musculus OX=10090 GN=Prpf6 PE=1 SV=1	
sp|Q9CZN7-2|GLYM_MOUSE Isoform 2 of Serine hydroxymethyltransferase, mitochondrial...	sp|Q9CZN7-2|GLYM_MOUSE Isoform 2 of Serine hydroxymethyltransferase, mitochondrial...	
F8WHR6_MOUSE	Probable ATP-dependent RNA helicase DDX46 OS=Mus musculus OX=10090 GN=Ddx46 PE=1 SV=1	
G3UXL2_MOUSE	Phosphoribosyl pyrophosphate synthetase 1-like 3 OS=Mus musculus OX=10090 GN=Prps1l3 PE=3 SV=1	
IPO7_MOUSE	Importin-7 OS=Mus musculus OX=10090 GN=Ipo7 PE=1 SV=2	
Q3TIY6_MOUSE	Rab GDP dissociation inhibitor OS=Mus musculus OX=10090 GN=Gdi2 PE=2 SV=1	
Q9CZI7_MOUSE	Annexin OS=Mus musculus OX=10090 GN=Anxa2 PE=2 SV=1	
Q3UXP2_MOUSE	RuvB-like helicase OS=Mus musculus OX=10090 GN=Ruvbl2 PE=2 SV=1	
GFP	GFP	
PRP8_MOUSE	Pre-mRNA-processing-splicing factor 8 OS=Mus musculus OX=10090 GN=Prpf8 PE=1 SV=2	
ECM29_MOUSE	Isoform 2 of Proteasome adapter and scaffold protein ECM29 OS=Mus musculus OX=10090 GN=Ecpas	
Q99JX6_MOUSE	Annexin OS=Mus musculus OX=10090 GN=Anxa6 PE=2 SV=1	
sp|Q99KP6-2|PRP19_MOUSE Isoform 2 of Pre-mRNA-processing factor 19 OS=Mus musculus...	sp|Q99KP6-2|PRP19_MOUSE Isoform 2 of Pre-mRNA-processing factor 19 OS=Mus musculus...	
tr|Q8QZS9|Q8QZS9_MOUSE Psmc6 protein (Fragment) OS=Mus musculus OX=10090 GN=Psmc6...	Psmc6 protein (Fragment) OS=Mus musculus OX=10090 GN=Psmc6 PE=2 SV=1	
G5E924_MOUSE	Heterogeneous nuclear ribonucleoprotein L (Fragment) OS=Mus musculus OX=10090 GN=Hnrnpl PE=1 SV=1	
Q3TJG1_MOUSE	Platelet-activating factor acetylhydrolase IB subunit alpha OS=Mus musculus OX=10090 GN=Pafah1b1 PE=2 SV=1	
Q3UJS2_MOUSE	Uncharacterized protein OS=Mus musculus OX=10090 GN=Fbl PE=2 SV=1	
Q8BNS0_MOUSE	Uncharacterized protein OS=Mus musculus OX=10090 GN=Drg1 PE=2 SV=1	
sp|P70698|PYRG1_MOUSECTP synthase 1 OS=Mus musculus OX=10090 GN=Ctps1 PE=1 SV=2	sp|P70698|PYRG1_MOUSE CTP synthase 1 OS=Mus musculus OX=10090 GN=Ctps1 PE=1 SV=2	
DESP_MOUSE	Desmoplakin OS=Mus musculus OX=10090 GN=Dsp PE=1 SV=1	
GARS_MOUSE	Glycine--tRNA ligase OS=Mus musculus OX=10090 GN=Gars PE=1 SV=1	
KCRB_MOUSE	Creatine kinase B-type OS=Mus musculus OX=10090 GN=Ckb PE=1 SV=1	
Q569X4_MOUSE	Proteasome (Prosome, macropain) 26S subunit, ATPase, 4 OS=Mus musculus OX=10090 GN=Psmc4 PE=2 SV=1	
Q5CZY9_MOUSE	Rps16 protein OS=Mus musculus OX=10090 GN=Rps16 PE=2 SV=1	
sp|O70194|EIF3D_MOUSEEukaryotic translation initiation factor 3 subunit D OS=Mus musculus OX=10090 GN=Eif3d PE=1 SV=2	sp|O70194|EIF3D_MOUSE Eukaryotic translation initiation factor 3 subunit D OS=Mus musculus OX=10090 GN=Eif3d PE=1 SV=2	
sp|Q925I1-2|ATAD3_MOUSE Isoform 2 of ATPase family AAA domain-containing protein...	Isoform 2 of ATPase family AAA domain-containing protein 3 OS=Mus musculus OX=10090 GN=Atad3	
sp|Q9ESZ8-2|GTF2I_MOUSE Isoform 2 of General transcription factor II-I OS=Mus musculus...	Isoform 2 of General transcription factor II-I OS=Mus musculus OX=10090 GN=Gtf2i	
tr|D3YZ35|D3YZ35_MOUSE Neurofilament medium polypeptide OS=Mus musculus OX=10090...	Neurofilament medium polypeptide OS=Mus musculus OX=10090 GN=Nefm PE=1 SV=1	
tr|Q69ZW4|Q69ZW4_MOUSE MKIAA0899 protein (Fragment) OS=Mus musculus OX=10090 GN=Ap2a2...	MKIAA0899 protein (Fragment) OS=Mus musculus OX=10090 GN=Ap2a2 PE=2 SV=1	
TCPE_MOUSE	T-complex protein 1 subunit epsilon OS=Mus musculus OX=10090 GN=Cct5 PE=1 SV=1	
B2RXR3_MOUSE	Cnot1 protein OS=Mus musculus OX=10090 GN=Cnot1 PE=2 SV=1	
Q80WV6_MOUSE	Prmt1 protein OS=Mus musculus OX=10090 GN=Prmt1 PE=2 SV=1	
sp|Q64737|PUR2_MOUSE Trifunctional purine biosynthetic protein adenosine-3 OS=Mus...	sp|Q64737|PUR2_MOUSE Trifunctional purine biosynthetic protein adenosine-3 OS=Mus...	
sp|Q6PGB8-2|SMCA1_MOUSE Isoform 2 of Probable global transcription activator SNF2L1...	Isoform 2 of Probable global transcription activator SNF2L1 OS=Mus musculus OX=10090 GN=Smarca1	
sp|Q9JKR6|HYOU1_MOUSEHypoxia up-regulated protein 1 OS=Mus musculus OX=10090 GN=Hyou1 PE=1 SV=1	sp|Q9JKR6|HYOU1_MOUSE Hypoxia up-regulated protein 1 OS=Mus musculus OX=10090 GN=Hyou1 PE=1 SV=1	
sp|Q9QXS1-3|PLEC_MOUSE	Isoform PLEC-1A of Plectin OS=Mus musculus OX=10090 GN=Plec	
tr|B1AUX2|B1AUX2_MOUSE Host cell factor 1 OS=Mus musculus OX=10090 GN=Hcfc1 PE=1...	Host cell factor 1 OS=Mus musculus OX=10090 GN=Hcfc1 PE=1 SV=1	
tr|Q3UDI8|Q3UDI8_MOUSE DNA replication licensing factor MCM7 OS=Mus musculus OX=10090...	DNA replication licensing factor MCM7 OS=Mus musculus OX=10090 GN=Mcm7 PE=2 SV=1	
tr|Q3UYQ4|Q3UYQ4_MOUSE Uncharacterized protein OS=Mus musculus OX=10090 GN=Api5 PE=2...	Uncharacterized protein OS=Mus musculus OX=10090 GN=Api5 PE=2 SV=1	
A2A513_MOUSE	Keratin, type I cytoskeletal 10 OS=Mus musculus OX=10090 GN=Krt10 PE=1 SV=1	
B2RXM2_MOUSE	EG627828 protein OS=Mus musculus OX=10090 GN=Gm6793 PE=1 SV=1	
G3UVV4_MOUSE	Hexokinase 1, isoform CRA_f OS=Mus musculus OX=10090 GN=Hk1 PE=1 SV=1	
PGM1_MOUSE	Phosphoglucomutase-1 OS=Mus musculus OX=10090 GN=Pgm1 PE=1 SV=4	
PUR9_MOUSE	Bifunctional purine biosynthesis protein PURH OS=Mus musculus OX=10090 GN=Atic PE=1 SV=2	
Q3TLE5_MOUSE	Uncharacterized protein OS=Mus musculus OX=10090 GN=Rps2 PE=2 SV=1	
Q3UBI6_MOUSE	Uncharacterized protein OS=Mus musculus OX=10090 GN=Rpl7 PE=2 SV=1	
Q91VZ1_MOUSE	Snx2 protein OS=Mus musculus OX=10090 GN=Snx2 PE=2 SV=1	
sp|Q810A7-2|DDX42_MOUSE Isoform 2 of ATP-dependent RNA helicase DDX42 OS=Mus musculus...	sp|Q810A7-2|DDX42_MOUSE Isoform 2 of ATP-dependent RNA helicase DDX42 OS=Mus musculus...	
sp|Q91ZW3|SMCA5_MOUSESWI/SNF-related matrix-associated actin-dependent regulator of chromatin subfamily A member 5 OS=Mus musculus OX=10090 GN=Smarca5 PE=1 SV=1	sp|Q91ZW3|SMCA5_MOUSE SWI/SNF-related matrix-associated actin-dependent regulator of chromatin subfamily A member 5 OS=Mus musculus OX=10090 GN=Smarca5 PE=1 SV=1	
sp|Q9Z1Z0|USO1_MOUSE	General vesicular transport factor p115 OS=Mus musculus OX=10090 GN=Uso1 PE=1 SV=2	
sp|O55029|COPB2_MOUSECoatomer subunit beta' OS=Mus musculus OX=10090 GN=Copb2 PE=1 SV=2	sp|O55029|COPB2_MOUSE Coatomer subunit beta' OS=Mus musculus OX=10090 GN=Copb2 PE=1 SV=2	
SYIC_MOUSE	Isoleucine--tRNA ligase, cytoplasmic OS=Mus musculus OX=10090 GN=Iars PE=1 SV=2	
E9Q2X6_MOUSE	Structural maintenance of chromosomes protein OS=Mus musculus OX=10090 GN=Smc4 PE=1 SV=1	
Q3THJ6_MOUSE	Uncharacterized protein OS=Mus musculus OX=10090 PE=2 SV=1	
Q3TIA9_MOUSE	Uncharacterized protein OS=Mus musculus OX=10090 GN=Asns PE=2 SV=1	
Q3TJ43_MOUSE	Vacuolar protein sorting-associated protein 35 OS=Mus musculus OX=10090 GN=Vps35 PE=2 SV=1	
sp|P62830|RL23_MOUSE 60S ribosomal protein L23 OS=Mus musculus OX=10090 GN=Rpl23...	sp|P62830|RL23_MOUSE 60S ribosomal protein L23 OS=Mus musculus OX=10090 GN=Rpl23...	
tr|E9Q3D6|E9Q3D6_MOUSE Heat shock protein HSP 90-beta (Fragment) OS=Mus musculus...	tr|E9Q3D6|E9Q3D6_MOUSE Heat shock protein HSP 90-beta (Fragment) OS=Mus musculus...	
tr|Q8BU34|Q8BU34_MOUSE Uncharacterized protein (Fragment) OS=Mus musculus OX=10090...	Uncharacterized protein (Fragment) OS=Mus musculus OX=10090 GN=Psmd1 PE=2 SV=2	
Q8BZY3_MOUSE	DEAD (Asp-Glu-Ala-Asp) box polypeptide 19b OS=Mus musculus OX=10090 GN=Ddx19b PE=1 SV=1	
PHB_MOUSE	Prohibitin OS=Mus musculus OX=10090 GN=Phb PE=1 SV=1	
Q3ULT2_MOUSE	Actinin alpha 4 OS=Mus musculus OX=10090 GN=Actn4 PE=1 SV=1	
Q5M9L9_MOUSE	40S ribosomal protein S8 OS=Mus musculus OX=10090 GN=Rps8 PE=2 SV=1	
sp|P06745|G6PI_MOUSE Glucose-6-phosphate isomerase OS=Mus musculus OX=10090 GN=Gpi...	sp|P06745|G6PI_MOUSE Glucose-6-phosphate isomerase OS=Mus musculus OX=10090 GN=Gpi...	
sp|P70388-2|RAD50_MOUSE	Isoform 2 of DNA repair protein RAD50 OS=Mus musculus OX=10090 GN=Rad50	
tr|Q3UIQ2|Q3UIQ2_MOUSE Uncharacterized protein OS=Mus musculus OX=10090 GN=Ndufs1...	Uncharacterized protein OS=Mus musculus OX=10090 GN=Ndufs1 PE=2 SV=1	
tr|Q3UJS0|Q3UJS0_MOUSE Uncharacterized protein OS=Mus musculus OX=10090 GN=Rpl8 PE=2...	tr|Q3UJS0|Q3UJS0_MOUSE Uncharacterized protein OS=Mus musculus OX=10090 GN=Rpl8 PE=2...	
tr|Q5M8R8|Q5M8R8_MOUSE 60S acidic ribosomal protein P0 OS=Mus musculus OX=10090 GN=Rplp0...	60S acidic ribosomal protein P0 OS=Mus musculus OX=10090 GN=Rplp0 PE=2 SV=1	
tr|Q91ZH2|Q91ZH2_MOUSE Proliferating cell nuclear antigen OS=Mus musculus OX=10090...	tr|Q91ZH2|Q91ZH2_MOUSE Proliferating cell nuclear antigen OS=Mus musculus OX=10090...	
Q08EK4_MOUSE	Keratin 77 OS=Mus musculus OX=10090 GN=Krt77 PE=2 SV=1	
Q3TWJ4_MOUSE	Uncharacterized protein OS=Mus musculus OX=10090 GN=Actr3 PE=2 SV=1	
Q3UJZ7_MOUSE	Uncharacterized protein OS=Mus musculus OX=10090 GN=Nop56 PE=2 SV=1	
Q8BK73_MOUSE	Uncharacterized protein OS=Mus musculus OX=10090 GN=Psmd11 PE=2 SV=1	
sp|P54071|IDHP_MOUSE Isocitrate dehydrogenase [NADP], mitochondrial OS=Mus musculus...	Isocitrate dehydrogenase [NADP], mitochondrial OS=Mus musculus OX=10090 GN=Idh2 PE=1 SV=3	
sp|P60335|PCBP1_MOUSEPoly(rC)-binding protein 1 OS=Mus musculus OX=10090 GN=Pcbp1 PE=1 SV=1	sp|P60335|PCBP1_MOUSE Poly(rC)-binding protein 1 OS=Mus musculus OX=10090 GN=Pcbp1 PE=1 SV=1	
sp|Q8C7X2-2|EMC1_MOUSE Isoform 2 of ER membrane protein complex subunit 1 OS=Mus...	Isoform 2 of ER membrane protein complex subunit 1 OS=Mus musculus OX=10090 GN=Emc1	
sp|Q99MR6|SRRT_MOUSE	Serrate RNA effector molecule homolog OS=Mus musculus OX=10090 GN=Srrt PE=1 SV=1	
sp|Q99PU8-2|DHX30_MOUSE Isoform 2 of Putative ATP-dependent RNA helicase DHX30 OS=Mus...	Isoform 2 of Putative ATP-dependent RNA helicase DHX30 OS=Mus musculus OX=10090 GN=Dhx30	
sp|Q9QXX4|CMC2_MOUSE Calcium-binding mitochondrial carrier protein Aralar2 OS=Mus...	sp|Q9QXX4|CMC2_MOUSE Calcium-binding mitochondrial carrier protein Aralar2 OS=Mus...	
tr|A0A0R4J008|A0A0R4J008_MOUSE Histone deacetylase OS=Mus musculus OX=10090 GN=Hdac2...	tr|A0A0R4J008|A0A0R4J008_MOUSE Histone deacetylase OS=Mus musculus OX=10090 GN=Hdac2...	
tr|A0A0R4J0K2|A0A0R4J0K2_MOUSE Cytoskeleton-associated protein 5 OS=Mus musculus...	tr|A0A0R4J0K2|A0A0R4J0K2_MOUSE Cytoskeleton-associated protein 5 OS=Mus musculus...	
tr|A0JNY7|A0JNY7_MOUSE Eukaryotic translation initiation factor 4, gamma 2 OS=Mus...	Eukaryotic translation initiation factor 4, gamma 2 OS=Mus musculus OX=10090 GN=Eif4g2 PE=2 SV=1	
tr|Q3UKX8|Q3UKX8_MOUSE Uncharacterized protein OS=Mus musculus OX=10090 GN=Ap1g1...	Uncharacterized protein OS=Mus musculus OX=10090 GN=Ap1g1 PE=2 SV=1	
tr|Q8BTX6|Q8BTX6_MOUSE Uncharacterized protein (Fragment) OS=Mus musculus OX=10090...	tr|Q8BTX6|Q8BTX6_MOUSE Uncharacterized protein (Fragment) OS=Mus musculus OX=10090...	
Q3UVI9_MOUSE	Uncharacterized protein OS=Mus musculus OX=10090 GN=Abcf2 PE=2 SV=1	
sp|Q9CU62|SMC1A_MOUSEStructural maintenance of chromosomes protein 1A OS=Mus musculus OX=10090 GN=Smc1a PE=1 SV=4	sp|Q9CU62|SMC1A_MOUSE Structural maintenance of chromosomes protein 1A OS=Mus musculus OX=10090 GN=Smc1a PE=1 SV=4	
Q3U5M8_MOUSE	Uncharacterized protein OS=Mus musculus OX=10090 GN=Psmd3 PE=2 SV=1	
Q5SW83_MOUSE	ARP2 actin-related protein 2 homolog (Yeast) OS=Mus musculus OX=10090 GN=Actr2 PE=1 SV=1	
Q80V79_MOUSE	DNA mismatch repair protein OS=Mus musculus OX=10090 GN=Msh2 PE=2 SV=1	
RPN1_MOUSE	Dolichyl-diphosphooligosaccharide--protein glycosyltransferase subunit 1 OS=Mus musculus OX=10090 GN=Rpn1 PE=1 SV=1	
sp|P47857-3|PFKAM_MOUSE	Isoform 3 of ATP-dependent 6-phosphofructokinase, muscle type OS=Mus musculus OX=10090 GN=Pfkm	
sp|Q5XJY5|COPD_MOUSE Coatomer subunit delta OS=Mus musculus OX=10090 GN=Arcn1 PE=1...	sp|Q5XJY5|COPD_MOUSE Coatomer subunit delta OS=Mus musculus OX=10090 GN=Arcn1 PE=1...	
tr|A2A547|A2A547_MOUSE Ribosomal protein L19 OS=Mus musculus OX=10090 GN=Rpl19 PE=1...	Ribosomal protein L19 OS=Mus musculus OX=10090 GN=Rpl19 PE=1 SV=1	
A0A0R4J119_MOUSE	Cytoplasmic FMR1-interacting protein 1 OS=Mus musculus OX=10090 GN=Cyfip1 PE=1 SV=1	
IF2H_MOUSE	Eukaryotic translation initiation factor 2 subunit 3, Y-linked OS=Mus musculus OX=10090 GN=Eif2s3y PE=1 SV=2	
Q3TGQ3_MOUSE	Uncharacterized protein OS=Mus musculus OX=10090 GN=Ctnna1 PE=2 SV=1	
Q80X48_MOUSE	Nucleoporin 155 OS=Mus musculus OX=10090 GN=Nup155 PE=2 SV=1	
RS11_MOUSE	40S ribosomal protein S11 OS=Mus musculus OX=10090 GN=Rps11 PE=1 SV=3	
sp|Q8BMJ2|SYLC_MOUSE Leucine--tRNA ligase, cytoplasmic OS=Mus musculus OX=10090 GN=Lars...	sp|Q8BMJ2|SYLC_MOUSE Leucine--tRNA ligase, cytoplasmic OS=Mus musculus OX=10090 GN=Lars...	
tr|A0A0A0MQ76|A0A0A0MQ76_MOUSE Nucleolar protein 58 OS=Mus musculus OX=10090 GN=Nop58...	tr|A0A0A0MQ76|A0A0A0MQ76_MOUSE Nucleolar protein 58 OS=Mus musculus OX=10090 GN=Nop58...	
tr|A2AJ72|A2AJ72_MOUSE Far upstream element (FUSE)-binding protein 3 OS=Mus musculus...	Far upstream element (FUSE)-binding protein 3 OS=Mus musculus OX=10090 GN=Fubp3 PE=1 SV=1	
tr|Q3UAG2|Q3UAG2_MOUSE 6-phosphogluconate dehydrogenase, decarboxylating OS=Mus musculus...	6-phosphogluconate dehydrogenase, decarboxylating OS=Mus musculus OX=10090 GN=Pgd PE=2 SV=1	
tr|Q3ULW0|Q3ULW0_MOUSE GTP-binding nuclear protein Ran OS=Mus musculus OX=10090 GN=Ran...	GTP-binding nuclear protein Ran OS=Mus musculus OX=10090 GN=Ran PE=2 SV=1	
tr|Q3UZT7|Q3UZT7_MOUSE Uncharacterized protein OS=Mus musculus OX=10090 GN=Ctnnb1...	tr|Q3UZT7|Q3UZT7_MOUSE Uncharacterized protein OS=Mus musculus OX=10090 GN=Ctnnb1...	
tr|Q5FW97|Q5FW97_MOUSE Enolase 1, alpha non-neuron OS=Mus musculus OX=10090 GN=EG433182...	tr|Q5FW97|Q5FW97_MOUSE Enolase 1, alpha non-neuron OS=Mus musculus OX=10090 GN=EG433182...	
A3KML3_MOUSE	MCG126220 OS=Mus musculus OX=10090 GN=Ywhaq PE=2 SV=1	
Q3TG12_MOUSE	Uncharacterized protein (Fragment) OS=Mus musculus OX=10090 GN=Farsb PE=2 SV=1	
Q3TMJ8_MOUSE	Mitogen activated protein kinase kinase 1 OS=Mus musculus OX=10090 GN=Map2k1 PE=1 SV=1	
Q3U292_MOUSE	Uncharacterized protein OS=Mus musculus OX=10090 GN=Hist1h1d PE=2 SV=1	
Q3U8F5_MOUSE	Uncharacterized protein OS=Mus musculus OX=10090 GN=Psmd12 PE=2 SV=1	
Q497D7_MOUSE	Rpl30 protein OS=Mus musculus OX=10090 GN=Rpl30 PE=2 SV=1	
sp|Q3UKJ7|SMU1_MOUSE WD40 repeat-containing protein SMU1 OS=Mus musculus OX=10090...	sp|Q3UKJ7|SMU1_MOUSE WD40 repeat-containing protein SMU1 OS=Mus musculus OX=10090...	
sp|Q8CGK3|LONM_MOUSE Lon protease homolog, mitochondrial OS=Mus musculus OX=10090...	sp|Q8CGK3|LONM_MOUSE Lon protease homolog, mitochondrial OS=Mus musculus OX=10090...	
sp|Q9ESE1|LRBA_MOUSE	Lipopolysaccharide-responsive and beige-like anchor protein OS=Mus musculus OX=10090 GN=Lrba PE=1 SV=1	
tr|A0A1D5RLM8|A0A1D5RLM8_MOUSE Predicted gene 11639 OS=Mus musculus OX=10090 GN=Gm11639...	Predicted gene 11639 OS=Mus musculus OX=10090 GN=Gm11639 PE=1 SV=1	
tr|G3UX26|G3UX26_MOUSE Voltage-dependent anion-selective channel protein 2 OS=Mus...	tr|G3UX26|G3UX26_MOUSE Voltage-dependent anion-selective channel protein 2 OS=Mus...	
tr|Q3TWH2|Q3TWH2_MOUSE Uncharacterized protein OS=Mus musculus OX=10090 GN=Nnt PE=2...	Uncharacterized protein OS=Mus musculus OX=10090 GN=Nnt PE=2 SV=1	
tr|Q3UAT9|Q3UAT9_MOUSE Inosine-5'-monophosphate dehydrogenase OS=Mus musculus OX=10090...	tr|Q3UAT9|Q3UAT9_MOUSE Inosine-5'-monophosphate dehydrogenase OS=Mus musculus OX=10090...	
tr|Q8BK18|Q8BK18_MOUSE Uncharacterized protein OS=Mus musculus OX=10090 GN=Dars PE=2...	tr|Q8BK18|Q8BK18_MOUSE Uncharacterized protein OS=Mus musculus OX=10090 GN=Dars PE=2...	
tr|Q91V55|Q91V55_MOUSE 40S ribosomal protein S5 OS=Mus musculus OX=10090 GN=Rps5...	tr|Q91V55|Q91V55_MOUSE 40S ribosomal protein S5 OS=Mus musculus OX=10090 GN=Rps5...	
A0A1B0GRU8_MOUSE	Pre-mRNA-processing factor 40 homolog A OS=Mus musculus OX=10090 GN=Prpf40a PE=1 SV=1	
H2B1P_MOUSE	Isoform 2 of Histone H2B type 1-P OS=Mus musculus OX=10090 GN=Hist1h2bp	
H31_MOUSE	Histone H3.1 OS=Mus musculus OX=10090 GN=Hist1h3a PE=1 SV=2	
A0A286YDW8_MOUSE	26S proteasome non-ATPase regulatory subunit 6 (Fragment) OS=Mus musculus OX=10090 GN=Psmd6 PE=1 SV=1	
B1AT82_MOUSE	MCG6846, isoform CRA_c OS=Mus musculus OX=10090 GN=Prpsap1 PE=1 SV=1	
B2RUC7_MOUSE	Serine/threonine kinase receptor associated protein OS=Mus musculus OX=10090 GN=Strap PE=1 SV=1	
D3Z2H2_MOUSE	Catenin delta-1 OS=Mus musculus OX=10090 GN=Ctnnd1 PE=1 SV=1	
Q3TJ01_MOUSE	tRNA-splicing ligase RtcB homolog OS=Mus musculus OX=10090 GN=Rtcb PE=2 SV=1	
Q3ULI4_MOUSE	Signal transducer and activator of transcription OS=Mus musculus OX=10090 GN=Stat3 PE=2 SV=1	
Q99KA2_MOUSE	Tubulin alpha chain (Fragment) OS=Mus musculus OX=10090 GN=Tuba1b PE=2 SV=1	
Q99PB4_MOUSE	Mage-d2 OS=Mus musculus OX=10090 GN=Maged2 PE=2 SV=1	
sp|O55201-2|SPT5H_MOUSE Isoform 2 of Transcription elongation factor SPT5 OS=Mus...	sp|O55201-2|SPT5H_MOUSE Isoform 2 of Transcription elongation factor SPT5 OS=Mus...	
tr|D3YWT1|D3YWT1_MOUSE Heterogeneous nuclear ribonucleoprotein H3 OS=Mus musculus...	tr|D3YWT1|D3YWT1_MOUSE Heterogeneous nuclear ribonucleoprotein H3 OS=Mus musculus...	
tr|D3Z0M9|D3Z0M9_MOUSE DEAD (Asp-Glu-Ala-Asp) box polypeptide 23 OS=Mus musculus...	DEAD (Asp-Glu-Ala-Asp) box polypeptide 23 OS=Mus musculus OX=10090 GN=Ddx23 PE=1 SV=1	
tr|Q3TII4|Q3TII4_MOUSE Uncharacterized protein (Fragment) OS=Mus musculus OX=10090...	Uncharacterized protein (Fragment) OS=Mus musculus OX=10090 GN=Vars PE=2 SV=1	
tr|Q3TR62|Q3TR62_MOUSE DNA topoisomerase 2 (Fragment) OS=Mus musculus OX=10090 GN=Top2b...	tr|Q3TR62|Q3TR62_MOUSE DNA topoisomerase 2 (Fragment) OS=Mus musculus OX=10090 GN=Top2b...	
tr|Q5M8Q0|Q5M8Q0_MOUSE Ribosomal protein L15 OS=Mus musculus OX=10090 GN=Rpl15 PE=2...	Ribosomal protein L15 OS=Mus musculus OX=10090 GN=Rpl15 PE=2 SV=1	
A0A1W2P7Z8_MOUSE	Predicted gene 9045 OS=Mus musculus OX=10090 GN=Gm9045 PE=4 SV=1	
A0A0H2UH17_MOUSE	Ubiquitin associated protein 2-like, isoform CRA_b OS=Mus musculus OX=10090 GN=Ubap2l PE=1 SV=1	
A0A1D5RM85_MOUSE	60S ribosomal protein L18a (Fragment) OS=Mus musculus OX=10090 GN=Rpl18a PE=1 SV=1	
A2AAN2_MOUSE	Signal recognition particle subunit SRP68 OS=Mus musculus OX=10090 GN=Srp68 PE=1 SV=1	
A2AVJ7_MOUSE	Ribosome-binding protein 1 OS=Mus musculus OX=10090 GN=Rrbp1 PE=1 SV=1	
B1AXN9_MOUSE	Non-specific serine/threonine protein kinase OS=Mus musculus OX=10090 GN=Rps6ka3 PE=1 SV=1	
B2RX05_MOUSE	Slit2 protein OS=Mus musculus OX=10090 GN=Slit2 PE=2 SV=1	
F8WGL3_MOUSE	Cofilin-1 OS=Mus musculus OX=10090 GN=Cfl1 PE=1 SV=1	
O70569_MOUSE	Ribosomal protein S14 OS=Mus musculus OX=10090 GN=rps14 PE=3 SV=1	
Q3TQW3_MOUSE	Uncharacterized protein OS=Mus musculus OX=10090 GN=Ptbp1 PE=2 SV=1	
Q3U2V0_MOUSE	Uncharacterized protein OS=Mus musculus OX=10090 GN=Sympk PE=2 SV=1	
Q3UAJ1_MOUSE	Peptidyl-prolyl cis-trans isomerase OS=Mus musculus OX=10090 GN=Ppia PE=2 SV=1	
Q3UXT6_MOUSE	Uncharacterized protein (Fragment) OS=Mus musculus OX=10090 GN=Rbm39 PE=2 SV=1	
sp|P00493|HPRT_MOUSE Hypoxanthine-guanine phosphoribosyltransferase OS=Mus musculus...	sp|P00493|HPRT_MOUSE Hypoxanthine-guanine phosphoribosyltransferase OS=Mus musculus...	
sp|P48722-2|HS74L_MOUSE Isoform 2 of Heat shock 70 kDa protein 4L OS=Mus musculus...	Isoform 2 of Heat shock 70 kDa protein 4L OS=Mus musculus OX=10090 GN=Hspa4l	
sp|P97807|FUMH_MOUSE	Fumarate hydratase, mitochondrial OS=Mus musculus OX=10090 GN=Fh PE=1 SV=3	
sp|Q4VA53-3|PDS5B_MOUSE	Isoform 3 of Sister chromatid cohesion protein PDS5 homolog B OS=Mus musculus OX=10090 GN=Pds5b	
sp|Q6ZWR6-4|SYNE1_MOUSE	Isoform 4 of Nesprin-1 OS=Mus musculus OX=10090 GN=Syne1	
sp|Q8BTI8-2|SRRM2_MOUSE Isoform 2 of Serine/arginine repetitive matrix protein 2...	sp|Q8BTI8-2|SRRM2_MOUSE Isoform 2 of Serine/arginine repetitive matrix protein 2...	
sp|Q99KI0|ACON_MOUSE Aconitate hydratase, mitochondrial OS=Mus musculus OX=10090...	sp|Q99KI0|ACON_MOUSE Aconitate hydratase, mitochondrial OS=Mus musculus OX=10090...	
sp|Q9WVA3|BUB3_MOUSE Mitotic checkpoint protein BUB3 OS=Mus musculus OX=10090 GN=Bub3...	Mitotic checkpoint protein BUB3 OS=Mus musculus OX=10090 GN=Bub3 PE=1 SV=2	
tr|B2CY77|B2CY77_MOUSE Laminin receptor (Fragment) OS=Mus musculus OX=10090 GN=Rpsa...	tr|B2CY77|B2CY77_MOUSE Laminin receptor (Fragment) OS=Mus musculus OX=10090 GN=Rpsa...	
tr|Q3U6A2|Q3U6A2_MOUSE Uncharacterized protein OS=Mus musculus OX=10090 GN=Rbm14...	Uncharacterized protein OS=Mus musculus OX=10090 GN=Rbm14 PE=2 SV=1	
tr|Q8C605|Q8C605_MOUSE ATP-dependent 6-phosphofructokinase OS=Mus musculus OX=10090...	tr|Q8C605|Q8C605_MOUSE ATP-dependent 6-phosphofructokinase OS=Mus musculus OX=10090...	
Q6XMP4_MOUSE	Alanyl-tRNA synthase OS=Mus musculus OX=10090 GN=Aars PE=2 SV=1	
A0A1S6GWG6_MOUSE	Uncharacterized protein OS=Mus musculus OX=10090 GN=Atp6v1b2 PE=2 SV=1	
ACTZ_MOUSE	Alpha-centractin OS=Mus musculus OX=10090 GN=Actr1a PE=1 SV=1	
AMPB_MOUSE	Aminopeptidase B OS=Mus musculus OX=10090 GN=Rnpep PE=1 SV=2	
B1AXW6_MOUSE	Peroxiredoxin-1 (Fragment) OS=Mus musculus OX=10090 GN=Prdx1 PE=1 SV=1	
B2RRI2_MOUSE	Protein strawberry notch homolog 1 OS=Mus musculus OX=10090 GN=Sbno1 PE=1 SV=1	
CSK_MOUSE	Tyrosine-protein kinase CSK OS=Mus musculus OX=10090 GN=Csk PE=1 SV=2	
E9PUW7_MOUSE	Exportin-7 OS=Mus musculus OX=10090 GN=Xpo7 PE=1 SV=1	
Q3TIQ2_MOUSE	Uncharacterized protein OS=Mus musculus OX=10090 GN=Rpl12 PE=2 SV=1	
Q3TVM2_MOUSE	Uncharacterized protein OS=Mus musculus OX=10090 GN=Aldh2 PE=2 SV=1	
Q3TVN4_MOUSE	AP complex subunit beta OS=Mus musculus OX=10090 GN=Ap1b1 PE=2 SV=1	
Q3UDQ7_MOUSE	Uncharacterized protein OS=Mus musculus OX=10090 GN=Afg3l2 PE=2 SV=1	
Q3UIX8_MOUSE	Uncharacterized protein OS=Mus musculus OX=10090 GN=Ctbp2 PE=2 SV=1	
Q3V141_MOUSE	Uncharacterized protein (Fragment) OS=Mus musculus OX=10090 GN=Papola PE=2 SV=1	
Q5BKP5_MOUSE	DEAD (Asp-Glu-Ala-Asp) box polypeptide 50 OS=Mus musculus OX=10090 GN=Ddx50 PE=2 SV=1	
Q5SWZ0_MOUSE	Prpsap2 protein OS=Mus musculus OX=10090 GN=Prpsap2 PE=1 SV=1	
Q6IRT4_MOUSE	Eukaryotic translation initiation factor 3 subunit F OS=Mus musculus OX=10090 GN=Eif3f PE=2 SV=1	
Q80YG4_MOUSE	SEC63 OS=Mus musculus OX=10090 GN=Sec63 PE=2 SV=1	
Q8C2N0_MOUSE	DNA polymerase OS=Mus musculus OX=10090 GN=Pold1 PE=2 SV=1	
Q8CCE5_MOUSE	Uncharacterized protein (Fragment) OS=Mus musculus OX=10090 GN=Top2a PE=2 SV=1	
RS20_MOUSE	40S ribosomal protein S20 OS=Mus musculus OX=10090 GN=Rps20 PE=1 SV=1	
Z4YLI8_MOUSE	Clustered mitochondria protein homolog OS=Mus musculus OX=10090 GN=Cluh PE=1 SV=1	
sp|O35593|PSDE_MOUSE 26S proteasome non-ATPase regulatory subunit 14 OS=Mus musculus...	sp|O35593|PSDE_MOUSE 26S proteasome non-ATPase regulatory subunit 14 OS=Mus musculus...	
sp|P47757-2|CAPZB_MOUSE	Isoform 2 of F-actin-capping protein subunit beta OS=Mus musculus OX=10090 GN=Capzb	
sp|Q6NZN0-4|RBM26_MOUSE	Isoform 4 of RNA-binding protein 26 OS=Mus musculus OX=10090 GN=Rbm26	
sp|Q8CIG8|ANM5_MOUSE Protein arginine N-methyltransferase 5 OS=Mus musculus OX=10090...	sp|Q8CIG8|ANM5_MOUSE Protein arginine N-methyltransferase 5 OS=Mus musculus OX=10090...	
sp|Q9Z204-4|HNRPC_MOUSE	Isoform 4 of Heterogeneous nuclear ribonucleoproteins C1/C2 OS=Mus musculus OX=10090 GN=Hnrnpc	
tr|E9Q1W3|E9Q1W3_MOUSE-DECOY Nebulin OS=Mus musculus OX=10090 GN=Neb PE=1 SV=1	tr|E9Q1W3|E9Q1W3_MOUSE-DECOY Nebulin OS=Mus musculus OX=10090 GN=Neb PE=1 SV=1	
Q4FZG5_MOUSE	Arp2/3 complex 34 kDa subunit (Fragment) OS=Mus musculus OX=10090 GN=Arpc2 PE=2 SV=1	
sp|Q9QZE5|COPG1_MOUSECoatomer subunit gamma-1 OS=Mus musculus OX=10090 GN=Copg1 PE=1 SV=1	sp|Q9QZE5|COPG1_MOUSE Coatomer subunit gamma-1 OS=Mus musculus OX=10090 GN=Copg1 PE=1 SV=1	
Q91YC8_MOUSE	Damaged-DNA recognition protein 1 OS=Mus musculus OX=10090 GN=Ddb1 PE=2 SV=1	
A0A087WR97_MOUSE	TAR DNA-binding protein 43 (Fragment) OS=Mus musculus OX=10090 GN=Tardbp PE=1 SV=1	
A0A2I3BRL0_MOUSE	5'-nucleotidase domain containing 2 OS=Mus musculus OX=10090 GN=Nt5dc2 PE=1 SV=1	
A2ANY6_MOUSE	Midasin OS=Mus musculus OX=10090 GN=Mdn1 PE=1 SV=1	
B2RTP7_MOUSE	Krt2 protein OS=Mus musculus OX=10090 GN=Krt2 PE=2 SV=1	
E9QP46_MOUSE	Nesprin-2 OS=Mus musculus OX=10090 GN=Syne2 PE=1 SV=1	
Q3THH1_MOUSE	Uncharacterized protein OS=Mus musculus OX=10090 GN=Pdia6 PE=2 SV=1	
Q3TIJ1_MOUSE	Uncharacterized protein OS=Mus musculus OX=10090 GN=Cops4 PE=2 SV=1	
Q3UKP5_MOUSE	Uncharacterized protein OS=Mus musculus OX=10090 GN=Xab2 PE=2 SV=1	
Q9QWU1_MOUSE	Hsc70t (Fragment) OS=Mus musculus OX=10090 PE=3 SV=1	
RS9_MOUSE	40S ribosomal protein S9 OS=Mus musculus OX=10090 GN=Rps9 PE=1 SV=3	
sp|P28740-2|KIF2A_MOUSE Isoform 2 of Kinesin-like protein KIF2A OS=Mus musculus OX=10090...	Isoform 2 of Kinesin-like protein KIF2A OS=Mus musculus OX=10090 GN=Kif2a	
tr|E9Q616|E9Q616_MOUSE AHNAK nucleoprotein (desmoyokin) OS=Mus musculus OX=10090...	tr|E9Q616|E9Q616_MOUSE AHNAK nucleoprotein (desmoyokin) OS=Mus musculus OX=10090...	
tr|Q3TLE2|Q3TLE2_MOUSE Pleckstrin homology domain containing, family C (With FERM...	tr|Q3TLE2|Q3TLE2_MOUSE Pleckstrin homology domain containing, family C (With FERM...	
tr|Q99J57|Q99J57_MOUSE S-adenosylmethionine synthase OS=Mus musculus OX=10090 GN=Mat2a...	S-adenosylmethionine synthase OS=Mus musculus OX=10090 GN=Mat2a PE=1 SV=1	
B1ARU4_MOUSE	Microtubule-actin cross-linking factor 1 OS=Mus musculus OX=10090 GN=Macf1 PE=1 SV=1	
sp|Q62318-2|TIF1B_MOUSE Isoform 2 of Transcription intermediary factor 1-beta OS=Mus...	Isoform 2 of Transcription intermediary factor 1-beta OS=Mus musculus OX=10090 GN=Trim28	
Q3TEU8_MOUSE	Coronin OS=Mus musculus OX=10090 GN=Coro1c PE=2 SV=1	
A2A841_MOUSE	Protein 4.1 OS=Mus musculus OX=10090 GN=Epb41 PE=1 SV=1	
sp|P97313|PRKDC_MOUSEDNA-dependent protein kinase catalytic subunit OS=Mus musculus OX=10090 GN=Prkdc PE=1 SV=3	sp|P97313|PRKDC_MOUSE DNA-dependent protein kinase catalytic subunit OS=Mus musculus OX=10090 GN=Prkdc PE=1 SV=3	
sp|Q1HFZ0-2|NSUN2_MOUSE Isoform 2 of tRNA (cytosine(34)-C(5))-methyltransferase OS=Mus...	sp|Q1HFZ0-2|NSUN2_MOUSE Isoform 2 of tRNA (cytosine(34)-C(5))-methyltransferase OS=Mus...	
B2RUG7_MOUSE	Zinc finger RNA binding protein OS=Mus musculus OX=10090 GN=Zfr PE=2 SV=1	
E9Q1W0_MOUSE	Calcium/calmodulin-dependent protein kinase type II subunit delta OS=Mus musculus OX=10090 GN=Camk2d PE=1 SV=2	
ESYT1_MOUSE	Isoform 2 of Extended synaptotagmin-1 OS=Mus musculus OX=10090 GN=Esyt1	
H4_MOUSE	Histone H4 OS=Mus musculus OX=10090 GN=Hist1h4a PE=1 SV=2	
Q3TIP8_MOUSE	Chloride intracellular channel protein OS=Mus musculus OX=10090 GN=Clic1 PE=2 SV=1	
Q6PHZ1_MOUSE	Rpl17 protein OS=Mus musculus OX=10090 GN=Rpl17 PE=2 SV=1	
Q8BP16_MOUSE	Core histone macro-H2A OS=Mus musculus OX=10090 GN=H2afy2 PE=2 SV=1	
V9GX76_MOUSE	Unconventional myosin-VI OS=Mus musculus OX=10090 GN=Myo6 PE=1 SV=1	
sp|P62192|PRS4_MOUSE 26S proteasome regulatory subunit 4 OS=Mus musculus OX=10090...	sp|P62192|PRS4_MOUSE 26S proteasome regulatory subunit 4 OS=Mus musculus OX=10090...	
sp|Q8VHX6-2|FLNC_MOUSE	Isoform 2 of Filamin-C OS=Mus musculus OX=10090 GN=Flnc	
sp|Q91V12|BACH_MOUSE Cytosolic acyl coenzyme A thioester hydrolase OS=Mus musculus...	Cytosolic acyl coenzyme A thioester hydrolase OS=Mus musculus OX=10090 GN=Acot7 PE=1 SV=2	
sp|Q9QYB1|CLIC4_MOUSEChloride intracellular channel protein 4 OS=Mus musculus OX=10090 GN=Clic4 PE=1 SV=3	sp|Q9QYB1|CLIC4_MOUSE Chloride intracellular channel protein 4 OS=Mus musculus OX=10090 GN=Clic4 PE=1 SV=3	
tr|D3YTQ3|D3YTQ3_MOUSE Heterogeneous nuclear ribonucleoprotein D-like OS=Mus musculus...	tr|D3YTQ3|D3YTQ3_MOUSE Heterogeneous nuclear ribonucleoprotein D-like OS=Mus musculus...	
tr|Q20BD0|Q20BD0_MOUSE Heterogeneous nuclear ribonucleoprotein A/B OS=Mus musculus...	tr|Q20BD0|Q20BD0_MOUSE Heterogeneous nuclear ribonucleoprotein A/B OS=Mus musculus...	
tr|Q9CTC7|Q9CTC7_MOUSE Uncharacterized protein (Fragment) OS=Mus musculus OX=10090...	tr|Q9CTC7|Q9CTC7_MOUSE Uncharacterized protein (Fragment) OS=Mus musculus OX=10090...	
QCR1_MOUSE	Cytochrome b-c1 complex subunit 1, mitochondrial OS=Mus musculus OX=10090 GN=Uqcrc1 PE=1 SV=2	
B9EKK3_MOUSE	IQ motif containing GTPase activating protein 2 OS=Mus musculus OX=10090 GN=Iqgap2 PE=2 SV=1	
sp|P50516|VATA_MOUSE V-type proton ATPase catalytic subunit A OS=Mus musculus OX=10090...	sp|P50516|VATA_MOUSE V-type proton ATPase catalytic subunit A OS=Mus musculus OX=10090...	
sp|P24527|LKHA4_MOUSELeukotriene A-4 hydrolase OS=Mus musculus OX=10090 GN=Lta4h PE=1 SV=4	sp|P24527|LKHA4_MOUSE Leukotriene A-4 hydrolase OS=Mus musculus OX=10090 GN=Lta4h PE=1 SV=4	
Q4VA28_MOUSE	Ribosomal protein L21 OS=Mus musculus OX=10090 GN=Rpl21 PE=2 SV=1	
tr|Q3U4W8|Q3U4W8_MOUSE Ubiquitinyl hydrolase 1 OS=Mus musculus OX=10090 GN=Usp5 PE=1...	Ubiquitinyl hydrolase 1 OS=Mus musculus OX=10090 GN=Usp5 PE=1 SV=1	
Q5FWB7_MOUSE	Fructose-bisphosphate aldolase OS=Mus musculus OX=10090 GN=Aldoa PE=1 SV=1	
sp|Q9JLN9|MTOR_MOUSE	Serine/threonine-protein kinase mTOR OS=Mus musculus OX=10090 GN=Mtor PE=1 SV=2	
sp|B2RY56|RBM25_MOUSERNA-binding protein 25 OS=Mus musculus OX=10090 GN=Rbm25 PE=1 SV=2	sp|B2RY56|RBM25_MOUSE RNA-binding protein 25 OS=Mus musculus OX=10090 GN=Rbm25 PE=1 SV=2	
sp|O88477|IF2B1_MOUSEInsulin-like growth factor 2 mRNA-binding protein 1 OS=Mus musculus OX=10090 GN=Igf2bp1 PE=1 SV=1	sp|O88477|IF2B1_MOUSE Insulin-like growth factor 2 mRNA-binding protein 1 OS=Mus musculus OX=10090 GN=Igf2bp1 PE=1 SV=1	
A0A0R4J0I9_MOUSE	Low density lipoprotein receptor-related protein 1 OS=Mus musculus OX=10090 GN=Lrp1 PE=1 SV=1	
A1L3S7_MOUSE	Gatad2b protein OS=Mus musculus OX=10090 GN=Gatad2b PE=1 SV=1	
D6RE33_MOUSE	Enhancer of mRNA-decapping protein 4 OS=Mus musculus OX=10090 GN=Edc4 PE=1 SV=1	
E9Q6Q8_MOUSE	TBC1 domain family member 4 OS=Mus musculus OX=10090 GN=Tbc1d4 PE=1 SV=1	
E9QB02_MOUSE	Methionine--tRNA ligase, cytoplasmic OS=Mus musculus OX=10090 GN=Mars PE=1 SV=1	
Q05DU8_MOUSE	Ribonucleoside-diphosphate reductase (Fragment) OS=Mus musculus OX=10090 GN=Rrm1 PE=2 SV=1	
Q3TE06_MOUSE	Uncharacterized protein (Fragment) OS=Mus musculus OX=10090 GN=Wdr1 PE=2 SV=1	
Q3TKD0_MOUSE	Transportin-1 (Fragment) OS=Mus musculus OX=10090 GN=Tnpo1 PE=1 SV=1	
Q3TLX1_MOUSE	Uncharacterized protein OS=Mus musculus OX=10090 GN=Nampt PE=2 SV=1	
Q3UMM1_MOUSE	Tubulin beta chain OS=Mus musculus OX=10090 GN=Tubb6 PE=1 SV=1	
Q3UNG1_MOUSE	Uncharacterized protein (Fragment) OS=Mus musculus OX=10090 GN=Prpf8 PE=2 SV=1	
sp|P62267|RS23_MOUSE 40S ribosomal protein S23 OS=Mus musculus OX=10090 GN=Rps23...	sp|P62267|RS23_MOUSE 40S ribosomal protein S23 OS=Mus musculus OX=10090 GN=Rps23...	
sp|Q91VR5|DDX1_MOUSE ATP-dependent RNA helicase DDX1 OS=Mus musculus OX=10090 GN=Ddx1...	sp|Q91VR5|DDX1_MOUSE ATP-dependent RNA helicase DDX1 OS=Mus musculus OX=10090 GN=Ddx1...	
sp|Q9DBE9|SPB1_MOUSE pre-rRNA processing protein FTSJ3 OS=Mus musculus OX=10090 GN=Ftsj3...	sp|Q9DBE9|SPB1_MOUSE pre-rRNA processing protein FTSJ3 OS=Mus musculus OX=10090 GN=Ftsj3...	
tr|Q4FJZ2|Q4FJZ2_MOUSE Importin subunit alpha OS=Mus musculus OX=10090 GN=Kpna6 PE=1...	tr|Q4FJZ2|Q4FJZ2_MOUSE Importin subunit alpha OS=Mus musculus OX=10090 GN=Kpna6 PE=1...	
E9Q828_MOUSE	Calcium-transporting ATPase OS=Mus musculus OX=10090 GN=Atp2b4 PE=1 SV=1	
Q3TTV6_MOUSE	Tuftelin-interacting protein 11 OS=Mus musculus OX=10090 GN=Tfip11 PE=1 SV=1	
sp|O88569|ROA2_MOUSE	Heterogeneous nuclear ribonucleoproteins A2/B1 OS=Mus musculus OX=10090 GN=Hnrnpa2b1 PE=1 SV=2	
sp|P11103|PARP1_MOUSEPoly [ADP-ribose] polymerase 1 OS=Mus musculus OX=10090 GN=Parp1 PE=1 SV=3	sp|P11103|PARP1_MOUSE Poly [ADP-ribose] polymerase 1 OS=Mus musculus OX=10090 GN=Parp1 PE=1 SV=3	
E9Q0F0_MOUSE	Keratin 78 OS=Mus musculus OX=10090 GN=Krt78 PE=1 SV=1	
F8WJ41_MOUSE	40S ribosomal protein S15a (Fragment) OS=Mus musculus OX=10090 GN=Rps15a PE=1 SV=1	
Q3UKV5_MOUSE	Uncharacterized protein (Fragment) OS=Mus musculus OX=10090 GN=Hnrnpr PE=2 SV=1	
E9Q715_MOUSE	Putative RNA-binding protein Luc7-like 2 OS=Mus musculus OX=10090 GN=Luc7l2 PE=1 SV=1	
Q3TJF2_MOUSE	Obg-like ATPase 1 OS=Mus musculus OX=10090 GN=Ola1 PE=2 SV=1	
CUL3_MOUSE	Cullin-3 OS=Mus musculus OX=10090 GN=Cul3 PE=1 SV=1	
Z4YL78_MOUSE	Cytoskeleton-associated protein 5 OS=Mus musculus OX=10090 GN=Ckap5 PE=1 SV=1	
CISY_MOUSE	Citrate synthase, mitochondrial OS=Mus musculus OX=10090 GN=Cs PE=1 SV=1	
F6ZV59_MOUSE	Heterogeneous nuclear ribonucleoprotein D0 (Fragment) OS=Mus musculus OX=10090 GN=Hnrnpd PE=1 SV=1	
GDIA_MOUSE	Rab GDP dissociation inhibitor alpha OS=Mus musculus OX=10090 GN=Gdi1 PE=1 SV=3	
H3BJW3_MOUSE	Cleavage and polyadenylation-specificity factor subunit 6 OS=Mus musculus OX=10090 GN=Cpsf6 PE=1 SV=1	
Q3THL5_MOUSE	Protein pelota homolog OS=Mus musculus OX=10090 GN=Pelo PE=2 SV=1	
Q3TNL1_MOUSE	Glucose-6-phosphate 1-dehydrogenase OS=Mus musculus OX=10090 GN=G6pdx PE=2 SV=1	
Q6NWW1_MOUSE	Ubiquitination factor E4B, UFD2 homolog (S. cerevisiae) OS=Mus musculus OX=10090 GN=Ube4b PE=2 SV=1	
Q7TMW1_MOUSE	Rangap1 protein OS=Mus musculus OX=10090 GN=Rangap1 PE=2 SV=1	
Q80X98_MOUSE	DEAH (Asp-Glu-Ala-His) box polypeptide 38 OS=Mus musculus OX=10090 GN=Dhx38 PE=1 SV=1	
Q9CVR0_MOUSE	Tubulin beta chain (Fragment) OS=Mus musculus OX=10090 GN=Tubb4b PE=2 SV=2	
RS17_MOUSE	40S ribosomal protein S17 OS=Mus musculus OX=10090 GN=Rps17 PE=1 SV=2	
RS27_MOUSE	40S ribosomal protein S27 OS=Mus musculus OX=10090 GN=Rps27 PE=1 SV=3	
sp|P04104|K2C1_MOUSE Keratin, type II cytoskeletal 1 OS=Mus musculus OX=10090 GN=Krt1...	Keratin, type II cytoskeletal 1 OS=Mus musculus OX=10090 GN=Krt1 PE=1 SV=4	
sp|Q60902-4|EP15R_MOUSE	Isoform 4 of Epidermal growth factor receptor substrate 15-like 1 OS=Mus musculus OX=10090 GN=Eps15l1	
sp|Q6ZWX6|IF2A_MOUSE Eukaryotic translation initiation factor 2 subunit 1 OS=Mus...	Eukaryotic translation initiation factor 2 subunit 1 OS=Mus musculus OX=10090 GN=Eif2s1 PE=1 SV=3	
sp|Q7M6Y3-2|PICAL_MOUSE	Isoform 2 of Phosphatidylinositol-binding clathrin assembly protein OS=Mus musculus OX=10090 GN=Picalm	
sp|Q8CGY8|OGT1_MOUSE	UDP-N-acetylglucosamine--peptide N-acetylglucosaminyltransferase 110 kDa subunit OS=Mus musculus OX=10090 GN=Ogt PE=1 SV=2	
sp|Q9Z1F9|SAE2_MOUSE SUMO-activating enzyme subunit 2 OS=Mus musculus OX=10090 GN=Uba2...	sp|Q9Z1F9|SAE2_MOUSE SUMO-activating enzyme subunit 2 OS=Mus musculus OX=10090 GN=Uba2...	
tr|A0A0N4SVT1|A0A0N4SVT1_MOUSE Probable ATP-dependent RNA helicase DDX47 (Fragment)...	tr|A0A0N4SVT1|A0A0N4SVT1_MOUSE Probable ATP-dependent RNA helicase DDX47 (Fragment)...	
tr|E9QN31|E9QN31_MOUSE Probable 28S rRNA (cytosine-C(5))-methyltransferase OS=Mus...	tr|E9QN31|E9QN31_MOUSE Probable 28S rRNA (cytosine-C(5))-methyltransferase OS=Mus...	
tr|Q3TJK3|Q3TJK3_MOUSE Uncharacterized protein OS=Mus musculus OX=10090 GN=Serpinh1...	tr|Q3TJK3|Q3TJK3_MOUSE Uncharacterized protein OS=Mus musculus OX=10090 GN=Serpinh1...	
tr|Q3UJK5|Q3UJK5_MOUSE Uncharacterized protein OS=Mus musculus OX=10090 GN=Oat PE=2...	Uncharacterized protein OS=Mus musculus OX=10090 GN=Oat PE=2 SV=1	
S4R1I3_MOUSE	Xaa-Pro aminopeptidase 1 OS=Mus musculus OX=10090 GN=Xpnpep1 PE=1 SV=1	
G3X8R5_MOUSE	Glutamine-rich protein 1 OS=Mus musculus OX=10090 GN=Qrich1 PE=1 SV=1	
sp|A2AN08-5|UBR4_MOUSE	Isoform 5 of E3 ubiquitin-protein ligase UBR4 OS=Mus musculus OX=10090 GN=Ubr4	
ERF1_MOUSE	Eukaryotic peptide chain release factor subunit 1 OS=Mus musculus OX=10090 GN=Etf1 PE=1 SV=4	
HNRPF_MOUSE	Isoform 2 of Heterogeneous nuclear ribonucleoprotein F OS=Mus musculus OX=10090 GN=Hnrnpf	
sp|Q9QXK3-3|COPG2_MOUSE Isoform 3 of Coatomer subunit gamma-2 OS=Mus musculus OX=10090...	Isoform 3 of Coatomer subunit gamma-2 OS=Mus musculus OX=10090 GN=Copg2	
H7BX05_MOUSE	Obscurin OS=Mus musculus OX=10090 GN=Obscn PE=1 SV=2	
tr|Q3UIF3|Q3UIF3_MOUSE Uncharacterized protein OS=Mus musculus OX=10090 GN=Hsp90aa1...	tr|Q3UIF3|Q3UIF3_MOUSE Uncharacterized protein OS=Mus musculus OX=10090 GN=Hsp90aa1...	
sp|Q6P5E4|UGGG1_MOUSEUDP-glucose:glycoprotein glucosyltransferase 1 OS=Mus musculus OX=10090 GN=Uggt1 PE=1 SV=4	sp|Q6P5E4|UGGG1_MOUSE UDP-glucose:glycoprotein glucosyltransferase 1 OS=Mus musculus OX=10090 GN=Uggt1 PE=1 SV=4	
sp|Q8K363|DDX18_MOUSEATP-dependent RNA helicase DDX18 OS=Mus musculus OX=10090 GN=Ddx18 PE=1 SV=1	sp|Q8K363|DDX18_MOUSE ATP-dependent RNA helicase DDX18 OS=Mus musculus OX=10090 GN=Ddx18 PE=1 SV=1	
sp|Q8K2Z4|CND1_MOUSE	Condensin complex subunit 1 OS=Mus musculus OX=10090 GN=Ncapd2 PE=1 SV=2	
sp|Q00PI9|HNRL2_MOUSEHeterogeneous nuclear ribonucleoprotein U-like protein 2 OS=Mus musculus OX=10090 GN=Hnrnpul2 PE=1 SV=2	sp|Q00PI9|HNRL2_MOUSE Heterogeneous nuclear ribonucleoprotein U-like protein 2 OS=Mus musculus OX=10090 GN=Hnrnpul2 PE=1 SV=2	
sp|Q64511|TOP2B_MOUSEDNA topoisomerase 2-beta OS=Mus musculus OX=10090 GN=Top2b PE=1 SV=2	sp|Q64511|TOP2B_MOUSE DNA topoisomerase 2-beta OS=Mus musculus OX=10090 GN=Top2b PE=1 SV=2	
C1TM_MOUSE	Monofunctional C1-tetrahydrofolate synthase, mitochondrial OS=Mus musculus OX=10090 GN=Mthfd1l PE=1 SV=2	
CUL5_MOUSE	Cullin-5 OS=Mus musculus OX=10090 GN=Cul5 PE=1 SV=3	
E9Q5B5_MOUSE	Hexokinase-2 OS=Mus musculus OX=10090 GN=Hk2 PE=1 SV=1	
G3X928_MOUSE	SEC23-interacting protein OS=Mus musculus OX=10090 GN=Sec23ip PE=1 SV=1	
M1VHG6_MOUSE	Metastasis-associated protein MTA1 isoform 11 OS=Mus musculus OX=10090 GN=Mta1 PE=2 SV=1	
Q3TQ70_MOUSE	Beta1 subnuit of GTP-binding protein OS=Mus musculus OX=10090 GN=Gnb1 PE=1 SV=1	
Q80W12_MOUSE	TROVE domain family, member 2 OS=Mus musculus OX=10090 GN=Trove2 PE=2 SV=1	
Q80ZX0_MOUSE	Sec24-related gene family, member B (S. cerevisiae) OS=Mus musculus OX=10090 GN=Sec24b PE=1 SV=1	
Q8VDC3_MOUSE	Cytoplasmic aconitase OS=Mus musculus OX=10090 GN=aco1 PE=3 SV=1	
sp|P28481-5|CO2A1_MOUSE	Isoform 5 of Collagen alpha-1(II) chain OS=Mus musculus OX=10090 GN=Col2a1	
sp|P61205|ARF3_MOUSE ADP-ribosylation factor 3 OS=Mus musculus OX=10090 GN=Arf3 PE=2...	ADP-ribosylation factor 3 OS=Mus musculus OX=10090 GN=Arf3 PE=2 SV=2	
sp|P62141|PP1B_MOUSE Serine/threonine-protein phosphatase PP1-beta catalytic subunit...	sp|P62141|PP1B_MOUSE Serine/threonine-protein phosphatase PP1-beta catalytic subunit...	
sp|Q3UA06|PCH2_MOUSE Pachytene checkpoint protein 2 homolog OS=Mus musculus OX=10090...	sp|Q3UA06|PCH2_MOUSE Pachytene checkpoint protein 2 homolog OS=Mus musculus OX=10090...	
sp|Q61781|K1C14_MOUSEKeratin, type I cytoskeletal 14 OS=Mus musculus OX=10090 GN=Krt14 PE=1 SV=2	sp|Q61781|K1C14_MOUSE Keratin, type I cytoskeletal 14 OS=Mus musculus OX=10090 GN=Krt14 PE=1 SV=2	
sp|Q6NS46|RRP5_MOUSE Protein RRP5 homolog OS=Mus musculus OX=10090 GN=Pdcd11 PE=1...	sp|Q6NS46|RRP5_MOUSE Protein RRP5 homolog OS=Mus musculus OX=10090 GN=Pdcd11 PE=1...	
sp|Q8BRF7-3|SCFD1_MOUSE Isoform 3 of Sec1 family domain-containing protein 1 OS=Mus...	sp|Q8BRF7-3|SCFD1_MOUSE Isoform 3 of Sec1 family domain-containing protein 1 OS=Mus...	
tr|A0A0R4J187|A0A0R4J187_MOUSE X-ray repair cross-complementing protein 6 OS=Mus...	X-ray repair cross-complementing protein 6 OS=Mus musculus OX=10090 GN=Xrcc6 PE=1 SV=1	
tr|A3KG93|A3KG93_MOUSE Lysine-specific histone demethylase 1A OS=Mus musculus OX=10090...	tr|A3KG93|A3KG93_MOUSE Lysine-specific histone demethylase 1A OS=Mus musculus OX=10090...	
tr|E9QP46|E9QP46_MOUSE-DECOY Nesprin-2 OS=Mus musculus OX=10090 GN=Syne2 PE=1 SV=1	tr|E9QP46|E9QP46_MOUSE-DECOY Nesprin-2 OS=Mus musculus OX=10090 GN=Syne2 PE=1 SV=1	
tr|Q3TC91|Q3TC91_MOUSE Uncharacterized protein OS=Mus musculus OX=10090 GN=Cpsf3...	tr|Q3TC91|Q3TC91_MOUSE Uncharacterized protein OS=Mus musculus OX=10090 GN=Cpsf3...	
tr|Q8CD14|Q8CD14_MOUSE Uncharacterized protein OS=Mus musculus OX=10090 GN=Ddx54...	Uncharacterized protein OS=Mus musculus OX=10090 GN=Ddx54 PE=2 SV=1	
sp|P54276|MSH6_MOUSE DNA mismatch repair protein Msh6 OS=Mus musculus OX=10090 GN=Msh6...	sp|P54276|MSH6_MOUSE DNA mismatch repair protein Msh6 OS=Mus musculus OX=10090 GN=Msh6...	
tr|Q3UB63|Q3UB63_MOUSE Uncharacterized protein OS=Mus musculus OX=10090 GN=Slc25a3...	Uncharacterized protein OS=Mus musculus OX=10090 GN=Slc25a3 PE=2 SV=1	
A0A0G2JEP0_MOUSE	Fragile X mental retardation syndrome-related protein 1 OS=Mus musculus OX=10090 GN=Fxr1 PE=1 SV=1	
A0A0R4J0W6_MOUSE	Leucine rich repeat containing 40, isoform CRA_a OS=Mus musculus OX=10090 GN=Lrrc40 PE=1 SV=1	
IF2B2_MOUSE	Isoform 2 of Insulin-like growth factor 2 mRNA-binding protein 2 OS=Mus musculus OX=10090 GN=Igf2bp2	
tr|Q3UR54|Q3UR54_MOUSE Uncharacterized protein (Fragment) OS=Mus musculus OX=10090...	tr|Q3UR54|Q3UR54_MOUSE Uncharacterized protein (Fragment) OS=Mus musculus OX=10090...	
A0A087WP14_MOUSE	Kinectin OS=Mus musculus OX=10090 GN=Ktn1 PE=1 SV=1	
A0A0R4J098_MOUSE	Zinc finger protein 326 OS=Mus musculus OX=10090 GN=Zfp326 PE=1 SV=1	
sp|Q8K3H0|DP13A_MOUSEDCC-interacting protein 13-alpha OS=Mus musculus OX=10090 GN=Appl1 PE=1 SV=1	sp|Q8K3H0|DP13A_MOUSE DCC-interacting protein 13-alpha OS=Mus musculus OX=10090 GN=Appl1 PE=1 SV=1	
sp|Q8VDS4|RPR1A_MOUSERegulation of nuclear pre-mRNA domain-containing protein 1A OS=Mus musculus OX=10090 GN=Rprd1a PE=1 SV=1	sp|Q8VDS4|RPR1A_MOUSE Regulation of nuclear pre-mRNA domain-containing protein 1A OS=Mus musculus OX=10090 GN=Rprd1a PE=1 SV=1	
sp|P14115|RL27A_MOUSE60S ribosomal protein L27a OS=Mus musculus OX=10090 GN=Rpl27a PE=1 SV=5	sp|P14115|RL27A_MOUSE 60S ribosomal protein L27a OS=Mus musculus OX=10090 GN=Rpl27a PE=1 SV=5	
CHD5_MOUSE	Chromodomain-helicase-DNA-binding protein 5 OS=Mus musculus OX=10090 GN=Chd5 PE=1 SV=1	
sp|P48036|ANXA5_MOUSEAnnexin A5 OS=Mus musculus OX=10090 GN=Anxa5 PE=1 SV=1	sp|P48036|ANXA5_MOUSE Annexin A5 OS=Mus musculus OX=10090 GN=Anxa5 PE=1 SV=1	
A2RRR3_MOUSE	Clasp2 protein OS=Mus musculus OX=10090 GN=Clasp2 PE=2 SV=1	
RCC2_MOUSE	Protein RCC2 OS=Mus musculus OX=10090 GN=Rcc2 PE=1 SV=1	
sp|Q9CPN8|IF2B3_MOUSEInsulin-like growth factor 2 mRNA-binding protein 3 OS=Mus musculus OX=10090 GN=Igf2bp3 PE=1 SV=1	sp|Q9CPN8|IF2B3_MOUSE Insulin-like growth factor 2 mRNA-binding protein 3 OS=Mus musculus OX=10090 GN=Igf2bp3 PE=1 SV=1	
Q3U547_MOUSE	Uncharacterized protein OS=Mus musculus OX=10090 GN=Stt3a PE=2 SV=1	
E9Q481_MOUSE	Serine/threonine-protein phosphatase 4 regulatory subunit 3A OS=Mus musculus OX=10090 GN=Ppp4r3a PE=1 SV=1	
E9Q804_MOUSE	Ankyrin repeat domain-containing protein 17 OS=Mus musculus OX=10090 GN=Ankrd17 PE=1 SV=3	
K3W4Q5_MOUSE	Family with sequence similarity 186, member A OS=Mus musculus OX=10090 GN=Fam186a PE=4 SV=1	
Q8R1V9_MOUSE	Qars protein OS=Mus musculus OX=10090 GN=Qars PE=1 SV=1	
sp|P47963|RL13_MOUSE 60S ribosomal protein L13 OS=Mus musculus OX=10090 GN=Rpl13...	sp|P47963|RL13_MOUSE 60S ribosomal protein L13 OS=Mus musculus OX=10090 GN=Rpl13...	
sp|P55200-2|KMT2A_MOUSE-DECOY Isoform 2 of Histone-lysine N-methyltransferase 2A...	sp|P55200-2|KMT2A_MOUSE-DECOY Isoform 2 of Histone-lysine N-methyltransferase 2A...	
sp|Q70FJ1-2|AKAP9_MOUSE Isoform 2 of A-kinase anchor protein 9 OS=Mus musculus OX=10090...	Isoform 2 of A-kinase anchor protein 9 OS=Mus musculus OX=10090 GN=Akap9	
sp|Q8BL97-3|SRSF7_MOUSE Isoform 3 of Serine/arginine-rich splicing factor 7 OS=Mus...	sp|Q8BL97-3|SRSF7_MOUSE Isoform 3 of Serine/arginine-rich splicing factor 7 OS=Mus...	
sp|Q8K0D5|EFGM_MOUSE Elongation factor G, mitochondrial OS=Mus musculus OX=10090...	sp|Q8K0D5|EFGM_MOUSE Elongation factor G, mitochondrial OS=Mus musculus OX=10090...	
sp|Q99JX4|EIF3M_MOUSEEukaryotic translation initiation factor 3 subunit M OS=Mus musculus OX=10090 GN=Eif3m PE=1 SV=1	sp|Q99JX4|EIF3M_MOUSE Eukaryotic translation initiation factor 3 subunit M OS=Mus musculus OX=10090 GN=Eif3m PE=1 SV=1	
tr|A0A1W2P6U8|A0A1W2P6U8_MOUSE-DECOY RIKEN cDNA 4932415D10 gene OS=Mus musculus OX=10090...	tr|A0A1W2P6U8|A0A1W2P6U8_MOUSE-DECOY RIKEN cDNA 4932415D10 gene OS=Mus musculus OX=10090...	
tr|A3KFU8|A3KFU8_MOUSE Polyadenylate-binding protein OS=Mus musculus OX=10090 GN=Pabpc4...	Polyadenylate-binding protein OS=Mus musculus OX=10090 GN=Pabpc4 PE=1 SV=1	
tr|Q3UFJ3|Q3UFJ3_MOUSE Pyruvate dehydrogenase E1 component subunit alpha OS=Mus musculus...	tr|Q3UFJ3|Q3UFJ3_MOUSE Pyruvate dehydrogenase E1 component subunit alpha OS=Mus musculus...	
A0A0R4J1E3_MOUSE	Drebrin OS=Mus musculus OX=10090 GN=Dbn1 PE=1 SV=1	
D3YYI8_MOUSE	Histone deacetylase OS=Mus musculus OX=10090 GN=Gm10093 PE=3 SV=1	
H9KV00_MOUSE	Protein SON OS=Mus musculus OX=10090 GN=Son PE=1 SV=1	
sp|O88738-2|BIRC6_MOUSE	Isoform 2 of Baculoviral IAP repeat-containing protein 6 OS=Mus musculus OX=10090 GN=Birc6	
sp|Q99ME9|NOG1_MOUSE Nucleolar GTP-binding protein 1 OS=Mus musculus OX=10090 GN=Gtpbp4...	sp|Q99ME9|NOG1_MOUSE Nucleolar GTP-binding protein 1 OS=Mus musculus OX=10090 GN=Gtpbp4...	
Q4VAG4_MOUSE	MCG12304 OS=Mus musculus OX=10090 GN=Rpl22 PE=1 SV=1	
tr|Q3U7B3|Q3U7B3_MOUSE Uncharacterized protein OS=Mus musculus OX=10090 GN=Tmpo PE=2...	Uncharacterized protein OS=Mus musculus OX=10090 GN=Tmpo PE=2 SV=1	
A0A2I3BQS6_MOUSE	E3 ubiquitin-protein ligase UBR5 OS=Mus musculus OX=10090 GN=Ubr5 PE=1 SV=1	
sp|Q60598|SRC8_MOUSE Src substrate cortactin OS=Mus musculus OX=10090 GN=Cttn PE=1...	sp|Q60598|SRC8_MOUSE Src substrate cortactin OS=Mus musculus OX=10090 GN=Cttn PE=1...	
Q6PFF0_MOUSE	SR-related CTD-associated factor 4 OS=Mus musculus OX=10090 GN=Scaf4 PE=1 SV=1	
B1AX98_MOUSE	Leucine rich repeat containing 47 OS=Mus musculus OX=10090 GN=Lrrc47 PE=1 SV=1	
sp|Q8CCF0|PRP31_MOUSEU4/U6 small nuclear ribonucleoprotein Prp31 OS=Mus musculus OX=10090 GN=Prpf31 PE=1 SV=3	sp|Q8CCF0|PRP31_MOUSE U4/U6 small nuclear ribonucleoprotein Prp31 OS=Mus musculus OX=10090 GN=Prpf31 PE=1 SV=3	
A0A1B0GSX7_MOUSE	Nuclear pore complex protein Nup98-Nup96 OS=Mus musculus OX=10090 GN=Nup98 PE=1 SV=1	
Q99JZ4_MOUSE	GTP-binding protein SAR1a OS=Mus musculus OX=10090 GN=Sar1a PE=1 SV=1	
B2RQS6_MOUSE	Dhx36 protein OS=Mus musculus OX=10090 GN=Dhx36 PE=2 SV=1	
E9PZF0_MOUSE	Nucleoside diphosphate kinase OS=Mus musculus OX=10090 GN=Gm20390 PE=3 SV=1	
sp|P47911|RL6_MOUSE 60S ribosomal protein L6 OS=Mus musculus OX=10090 GN=Rpl6 PE=1...	sp|P47911|RL6_MOUSE 60S ribosomal protein L6 OS=Mus musculus OX=10090 GN=Rpl6 PE=1...	
sp|Q8BGH2|SAM50_MOUSESorting and assembly machinery component 50 homolog OS=Mus musculus OX=10090 GN=Samm50 PE=1 SV=1	sp|Q8BGH2|SAM50_MOUSE Sorting and assembly machinery component 50 homolog OS=Mus musculus OX=10090 GN=Samm50 PE=1 SV=1	
Q3UJI2_MOUSE	Uncharacterized protein OS=Mus musculus OX=10090 GN=Rbbp7 PE=2 SV=1	
sp|Q8BWW9|PKN2_MOUSE	Serine/threonine-protein kinase N2 OS=Mus musculus OX=10090 GN=Pkn2 PE=1 SV=3	
A0A1L1STE6_MOUSE	Isocitrate dehydrogenase [NAD] subunit, mitochondrial OS=Mus musculus OX=10090 GN=Idh3a PE=1 SV=1	
tr|Q059T9|Q059T9_MOUSE PRP4 pre-mRNA processing factor 4 homolog (Yeast) OS=Mus musculus...	tr|Q059T9|Q059T9_MOUSE PRP4 pre-mRNA processing factor 4 homolog (Yeast) OS=Mus musculus...	
ATRX_MOUSE	Transcriptional regulator ATRX OS=Mus musculus OX=10090 GN=Atrx PE=1 SV=3	
ERF3A_MOUSE	Isoform 2 of Eukaryotic peptide chain release factor GTP-binding subunit ERF3A OS=Mus musculus OX=10090 GN=Gspt1	
A0A0R4J036_MOUSE	Neurofilament 3, medium OS=Mus musculus OX=10090 GN=Nefm PE=1 SV=1	
A0A0G2JDV8_MOUSE	Calponin OS=Mus musculus OX=10090 GN=Cnn3 PE=1 SV=1	
A1L366_MOUSE	RAD21 homolog (S. pombe) OS=Mus musculus OX=10090 GN=Rad21 PE=2 SV=1	
A2RRJ4_MOUSE	Exportin 5 OS=Mus musculus OX=10090 GN=Xpo5 PE=2 SV=1	
A8DUL0_MOUSE	Beta-globin OS=Mus musculus OX=10090 GN=Hbbt1 PE=3 SV=1	
BAG6_MOUSE	Large proline-rich protein BAG6 OS=Mus musculus OX=10090 GN=Bag6 PE=1 SV=1	
Q3U548_MOUSE	UTP--glucose-1-phosphate uridylyltransferase OS=Mus musculus OX=10090 GN=Ugp2 PE=2 SV=1	
Q3UDK3_MOUSE	Uncharacterized protein OS=Mus musculus OX=10090 GN=Rfc5 PE=2 SV=1	
Q3UIZ1_MOUSE	UDP-glucose 6-dehydrogenase OS=Mus musculus OX=10090 GN=Ugdh PE=2 SV=1	
Q5M9J8_MOUSE	MCG13936 OS=Mus musculus OX=10090 GN=Rpl28 PE=1 SV=1	
sp|Q6ZWR6-4|SYNE1_MOUSE-DECOY Isoform 4 of Nesprin-1 OS=Mus musculus OX=10090 GN=Syne1	sp|Q6ZWR6-4|SYNE1_MOUSE-DECOY Isoform 4 of Nesprin-1 OS=Mus musculus OX=10090 GN=Syne1	
sp|Q8BTI8-2|SRRM2_MOUSE-DECOY Isoform 2 of Serine/arginine repetitive matrix protein...	sp|Q8BTI8-2|SRRM2_MOUSE-DECOY Isoform 2 of Serine/arginine repetitive matrix protein...	
sp|Q9D051|ODPB_MOUSE Pyruvate dehydrogenase E1 component subunit beta, mitochondrial...	sp|Q9D051|ODPB_MOUSE Pyruvate dehydrogenase E1 component subunit beta, mitochondrial...	
sp|Q9EP69|SAC1_MOUSE Phosphatidylinositide phosphatase SAC1 OS=Mus musculus OX=10090...	sp|Q9EP69|SAC1_MOUSE Phosphatidylinositide phosphatase SAC1 OS=Mus musculus OX=10090...	
sp|Q9JJ28|FLII_MOUSE Protein flightless-1 homolog OS=Mus musculus OX=10090 GN=Flii...	Protein flightless-1 homolog OS=Mus musculus OX=10090 GN=Flii PE=1 SV=1	
sp|Q9Z0E0|NCDN_MOUSE	Neurochondrin OS=Mus musculus OX=10090 GN=Ncdn PE=1 SV=1	
tr|D3YWF6|D3YWF6_MOUSE Ubiquitin thioesterase OTUB1 OS=Mus musculus OX=10090 GN=Otub1...	tr|D3YWF6|D3YWF6_MOUSE Ubiquitin thioesterase OTUB1 OS=Mus musculus OX=10090 GN=Otub1...	
tr|E9PUC2|E9PUC2_MOUSE Long-chain-fatty-acid--CoA ligase 3 OS=Mus musculus OX=10090...	tr|E9PUC2|E9PUC2_MOUSE Long-chain-fatty-acid--CoA ligase 3 OS=Mus musculus OX=10090...	
tr|E9Q5I9|E9Q5I9_MOUSE 26S proteasome non-ATPase regulatory subunit 13 OS=Mus musculus...	tr|E9Q5I9|E9Q5I9_MOUSE 26S proteasome non-ATPase regulatory subunit 13 OS=Mus musculus...	
tr|E9Q616|E9Q616_MOUSE-DECOY AHNAK nucleoprotein (desmoyokin) OS=Mus musculus OX=10090...	tr|E9Q616|E9Q616_MOUSE-DECOY AHNAK nucleoprotein (desmoyokin) OS=Mus musculus OX=10090...	
tr|Q497N1|Q497N1_MOUSE 40S ribosomal protein S26 OS=Mus musculus OX=10090 GN=Rps26...	40S ribosomal protein S26 OS=Mus musculus OX=10090 GN=Rps26 PE=1 SV=1	
tr|Q4FJL0|Q4FJL0_MOUSE RAB10, member RAS oncogene family OS=Mus musculus OX=10090...	tr|Q4FJL0|Q4FJL0_MOUSE RAB10, member RAS oncogene family OS=Mus musculus OX=10090...	
tr|Q80U83|Q80U83_MOUSE MKIAA0079 protein (Fragment) OS=Mus musculus OX=10090 GN=Sec24c...	tr|Q80U83|Q80U83_MOUSE MKIAA0079 protein (Fragment) OS=Mus musculus OX=10090 GN=Sec24c...	
tr|Q3TL52|Q3TL52_MOUSE Uncharacterized protein OS=Mus musculus OX=10090 GN=Ncl PE=2...	tr|Q3TL52|Q3TL52_MOUSE Uncharacterized protein OS=Mus musculus OX=10090 GN=Ncl PE=2...	
Q545V8_MOUSE	Uncharacterized protein OS=Mus musculus OX=10090 GN=Csnk2a2 PE=1 SV=1	
sp|P27773|PDIA3_MOUSEProtein disulfide-isomerase A3 OS=Mus musculus OX=10090 GN=Pdia3 PE=1 SV=2	sp|P27773|PDIA3_MOUSE Protein disulfide-isomerase A3 OS=Mus musculus OX=10090 GN=Pdia3 PE=1 SV=2	
tr|E9Q1Y3|E9Q1Y3_MOUSE-DECOY Apolipoprotein B-100 (Fragment) OS=Mus musculus OX=10090...	tr|E9Q1Y3|E9Q1Y3_MOUSE-DECOY Apolipoprotein B-100 (Fragment) OS=Mus musculus OX=10090...	
Q3TSX8_MOUSE	Uncharacterized protein OS=Mus musculus OX=10090 GN=Tomm70a PE=2 SV=1	
Q3TVT4_MOUSE	Uncharacterized protein (Fragment) OS=Mus musculus OX=10090 GN=Dync1li1 PE=2 SV=1	
A0A0U1RPW2_MOUSE	Tight junction protein ZO-1 OS=Mus musculus OX=10090 GN=Tjp1 PE=1 SV=1	
sp|P27546|MAP4_MOUSE	Microtubule-associated protein 4 OS=Mus musculus OX=10090 GN=Map4 PE=1 SV=3	
sp|Q9JLI8-2|SART3_MOUSE Isoform 2 of Squamous cell carcinoma antigen recognized by...	sp|Q9JLI8-2|SART3_MOUSE Isoform 2 of Squamous cell carcinoma antigen recognized by...	
A0A0R4J1G5_MOUSE	Erlin-1 OS=Mus musculus OX=10090 GN=Erlin1 PE=1 SV=1	
B1AT03_MOUSE	DNA ligase OS=Mus musculus OX=10090 GN=Lig3 PE=1 SV=1	
tr|Q9D0K3|Q9D0K3_MOUSE Uncharacterized protein OS=Mus musculus OX=10090 GN=Rfc2 PE=2...	Uncharacterized protein OS=Mus musculus OX=10090 GN=Rfc2 PE=2 SV=1	
APOB_MOUSE	Apolipoprotein B-100 OS=Mus musculus OX=10090 GN=Apob PE=1 SV=1	
sp|Q9DBG7|SRPRA_MOUSESignal recognition particle receptor subunit alpha OS=Mus musculus OX=10090 GN=Srpra PE=1 SV=1	sp|Q9DBG7|SRPRA_MOUSE Signal recognition particle receptor subunit alpha OS=Mus musculus OX=10090 GN=Srpra PE=1 SV=1	
A2A4N9_MOUSE	ATP-dependent RNA helicase DHX8 (Fragment) OS=Mus musculus OX=10090 GN=Dhx8 PE=1 SV=8	
E9Q1G8_MOUSE	Septin-7 OS=Mus musculus OX=10090 GN=Sept7 PE=1 SV=2	
sp|Q6A4J8-2|UBP7_MOUSE	Isoform 2 of Ubiquitin carboxyl-terminal hydrolase 7 OS=Mus musculus OX=10090 GN=Usp7	
sp|Q6NV83-2|SR140_MOUSE Isoform 2 of U2 snRNP-associated SURP motif-containing protein...	sp|Q6NV83-2|SR140_MOUSE Isoform 2 of U2 snRNP-associated SURP motif-containing protein...	
sp|E9PVX6-2|KI67_MOUSE-DECOY Isoform 2 of Proliferation marker protein Ki-67 OS=Mus...	sp|E9PVX6-2|KI67_MOUSE-DECOY Isoform 2 of Proliferation marker protein Ki-67 OS=Mus...	
Q8BTU6_MOUSE	Eukaryotic initiation factor 4A-II OS=Mus musculus OX=10090 GN=Eif4a2 PE=1 SV=1	
G3UXW9_MOUSE	COP9 signalosome complex subunit 1 OS=Mus musculus OX=10090 GN=Gps1 PE=1 SV=1	
sp|Q6P9L6|KIF15_MOUSEKinesin-like protein KIF15 OS=Mus musculus OX=10090 GN=Kif15 PE=1 SV=1	sp|Q6P9L6|KIF15_MOUSE Kinesin-like protein KIF15 OS=Mus musculus OX=10090 GN=Kif15 PE=1 SV=1	
PRAX_MOUSE	Periaxin OS=Mus musculus OX=10090 GN=Prx PE=1 SV=1	
sp|Q6PB66|LPPRC_MOUSELeucine-rich PPR motif-containing protein, mitochondrial OS=Mus musculus OX=10090 GN=Lrpprc PE=1 SV=2	sp|Q6PB66|LPPRC_MOUSE Leucine-rich PPR motif-containing protein, mitochondrial OS=Mus musculus OX=10090 GN=Lrpprc PE=1 SV=2	
Q5SUH6_MOUSE	Clathrin interactor 1 OS=Mus musculus OX=10090 GN=Clint1 PE=1 SV=1	
Q8BPH1_MOUSE	Uncharacterized protein OS=Mus musculus OX=10090 GN=Ywhae PE=2 SV=1	
Q8BTQ1_MOUSE	ELAV-like protein (Fragment) OS=Mus musculus OX=10090 GN=Elavl1 PE=2 SV=1	
Q0VDR7_MOUSE	Krt6b protein OS=Mus musculus OX=10090 GN=Krt6b PE=2 SV=1	
A0A0R4J195_MOUSE	Regulation of nuclear pre-mRNA domain-containing protein 1B OS=Mus musculus OX=10090 GN=Rprd1b PE=1 SV=1	
A0A2C9F2D2_MOUSE	Annexin OS=Mus musculus OX=10090 GN=Anxa7 PE=1 SV=1	
A2BFF8_MOUSE	Cytoplasmic dynein 1 intermediate chain 2 OS=Mus musculus OX=10090 GN=Dync1i2 PE=1 SV=1	
B1AUY7_MOUSE	N-alpha-acetyltransferase 10 OS=Mus musculus OX=10090 GN=Naa10 PE=1 SV=1	
CDK2_MOUSE	Cyclin-dependent kinase 2 OS=Mus musculus OX=10090 GN=Cdk2 PE=1 SV=2	
DYH5_MOUSE	Dynein heavy chain 5, axonemal OS=Mus musculus OX=10090 GN=Dnah5 PE=1 SV=2	
Q8BSZ8_MOUSE	Uncharacterized protein OS=Mus musculus OX=10090 GN=Srm PE=2 SV=1	
S4R2F3_MOUSE	Ankyrin-2 OS=Mus musculus OX=10090 GN=Ank2 PE=1 SV=3	
sp|A2AAE1-6|K1109_MOUSE-DECOY Isoform 6 of Uncharacterized protein KIAA1109 OS=Mus...	sp|A2AAE1-6|K1109_MOUSE-DECOY Isoform 6 of Uncharacterized protein KIAA1109 OS=Mus...	
sp|P26443|DHE3_MOUSE Glutamate dehydrogenase 1, mitochondrial OS=Mus musculus OX=10090...	sp|P26443|DHE3_MOUSE Glutamate dehydrogenase 1, mitochondrial OS=Mus musculus OX=10090...	
sp|P55200-2|KMT2A_MOUSE Isoform 2 of Histone-lysine N-methyltransferase 2A OS=Mus...	sp|P55200-2|KMT2A_MOUSE Isoform 2 of Histone-lysine N-methyltransferase 2A OS=Mus...	
sp|Q6R0H7-4|GNAS1_MOUSE Isoform XLas-4 of Guanine nucleotide-binding protein G(s)...	sp|Q6R0H7-4|GNAS1_MOUSE Isoform XLas-4 of Guanine nucleotide-binding protein G(s)...	
sp|Q80U72-2|SCRIB_MOUSE Isoform 2 of Protein scribble homolog OS=Mus musculus OX=10090...	sp|Q80U72-2|SCRIB_MOUSE Isoform 2 of Protein scribble homolog OS=Mus musculus OX=10090...	
sp|Q922J9-3|FACR1_MOUSE	Isoform 3 of Fatty acyl-CoA reductase 1 OS=Mus musculus OX=10090 GN=Far1	
sp|Q9JHS4|CLPX_MOUSE ATP-dependent Clp protease ATP-binding subunit clpX-like, mitochondrial...	sp|Q9JHS4|CLPX_MOUSE ATP-dependent Clp protease ATP-binding subunit clpX-like, mitochondrial...	
sp|Q9QZQ8-2|H2AY_MOUSE Isoform 1 of Core histone macro-H2A.1 OS=Mus musculus OX=10090...	sp|Q9QZQ8-2|H2AY_MOUSE Isoform 1 of Core histone macro-H2A.1 OS=Mus musculus OX=10090...	
tr|H7BX05|H7BX05_MOUSE-DECOY Obscurin OS=Mus musculus OX=10090 GN=Obscn PE=1 SV=2	tr|H7BX05|H7BX05_MOUSE-DECOY Obscurin OS=Mus musculus OX=10090 GN=Obscn PE=1 SV=2	
tr|Q3TQ33|Q3TQ33_MOUSE Uncharacterized protein (Fragment) OS=Mus musculus OX=10090...	tr|Q3TQ33|Q3TQ33_MOUSE Uncharacterized protein (Fragment) OS=Mus musculus OX=10090...	
Q3UE11_MOUSE	Uncharacterized protein OS=Mus musculus OX=10090 PE=2 SV=1	
sp|Q8BTW9|PAK4_MOUSE Serine/threonine-protein kinase PAK 4 OS=Mus musculus OX=10090...	Serine/threonine-protein kinase PAK 4 OS=Mus musculus OX=10090 GN=Pak4 PE=1 SV=1	
tr|A2AD84|A2AD84_MOUSE Serine/threonine-protein kinase 26 OS=Mus musculus OX=10090...	Serine/threonine-protein kinase 26 OS=Mus musculus OX=10090 GN=Stk26 PE=1 SV=1	
tr|F8VQ05|F8VQ05_MOUSE-DECOY FRY-like transcription coactivator OS=Mus musculus OX=10090...	tr|F8VQ05|F8VQ05_MOUSE-DECOY FRY-like transcription coactivator OS=Mus musculus OX=10090...	
tr|Q99K20|Q99K20_MOUSE L-lactate dehydrogenase (Fragment) OS=Mus musculus OX=10090...	L-lactate dehydrogenase (Fragment) OS=Mus musculus OX=10090 GN=Ldha PE=2 SV=1	
A0A286YDJ6_MOUSE	Lysine-specific demethylase 3B OS=Mus musculus OX=10090 GN=Kdm3b PE=1 SV=1	
sp|A2AWL7|MGAP_MOUSE	MAX gene-associated protein OS=Mus musculus OX=10090 GN=Mga PE=2 SV=1	
E9PWV3_MOUSE	Ribosomal protein S6 kinase OS=Mus musculus OX=10090 GN=Rps6ka1 PE=1 SV=1	
sp|P97412|LYST_MOUSE	Lysosomal-trafficking regulator OS=Mus musculus OX=10090 GN=Lyst PE=1 SV=1	
Q6PFB2_MOUSE	Rcc1 protein OS=Mus musculus OX=10090 GN=Rcc1 PE=1 SV=1	
Q4FJZ4_MOUSE	Wars protein OS=Mus musculus OX=10090 GN=Wars PE=2 SV=1	
tr|D3Z7E5|D3Z7E5_MOUSE Glycogen synthase kinase-3 alpha OS=Mus musculus OX=10090...	tr|D3Z7E5|D3Z7E5_MOUSE Glycogen synthase kinase-3 alpha OS=Mus musculus OX=10090...	
DLDH_MOUSE	Dihydrolipoyl dehydrogenase, mitochondrial OS=Mus musculus OX=10090 GN=Dld PE=1 SV=2	
Q5XJZ3_MOUSE	Grwd1 protein (Fragment) OS=Mus musculus OX=10090 GN=Grwd1 PE=2 SV=1	
B2RXQ8_MOUSE	MCG19514, isoform CRA_a OS=Mus musculus OX=10090 GN=Wdr82 PE=1 SV=1	
tr|E9QPD7|E9QPD7_MOUSE Pyruvate carboxylase OS=Mus musculus OX=10090 GN=Pcx PE=1...	tr|E9QPD7|E9QPD7_MOUSE Pyruvate carboxylase OS=Mus musculus OX=10090 GN=Pcx PE=1...	
VP26A_MOUSE	Isoform 2 of Vacuolar protein sorting-associated protein 26A OS=Mus musculus OX=10090 GN=Vps26a	
E9Q9M0_MOUSE	Centrosomal protein of 290 kDa OS=Mus musculus OX=10090 GN=Cep290 PE=1 SV=1	
tr|O08821|O08821_MOUSE Cytoplasmic dynein heavy chain (Fragment) OS=Mus musculus...	tr|O08821|O08821_MOUSE Cytoplasmic dynein heavy chain (Fragment) OS=Mus musculus...	
tr|Q3U6K8|Q3U6K8_MOUSE Uncharacterized protein OS=Mus musculus OX=10090 GN=Vdac1...	tr|Q3U6K8|Q3U6K8_MOUSE Uncharacterized protein OS=Mus musculus OX=10090 GN=Vdac1...	
A0A0U1RPL0_MOUSE	Ataxin-2-like protein OS=Mus musculus OX=10090 GN=Atxn2l PE=1 SV=1	
sp|Q61553|FSCN1_MOUSEFascin OS=Mus musculus OX=10090 GN=Fscn1 PE=1 SV=4	sp|Q61553|FSCN1_MOUSE Fascin OS=Mus musculus OX=10090 GN=Fscn1 PE=1 SV=4	
tr|Q9DCR1|Q9DCR1_MOUSE Uncharacterized protein (Fragment) OS=Mus musculus OX=10090...	Uncharacterized protein (Fragment) OS=Mus musculus OX=10090 GN=Tubb4b PE=2 SV=2	
sp|P05132-2|KAPCA_MOUSE Isoform 2 of cAMP-dependent protein kinase catalytic subunit...	sp|P05132-2|KAPCA_MOUSE Isoform 2 of cAMP-dependent protein kinase catalytic subunit...	
FAT3_MOUSE	Protocadherin Fat 3 OS=Mus musculus OX=10090 GN=Fat3 PE=1 SV=2	
SYAM_MOUSE	Alanine--tRNA ligase, mitochondrial OS=Mus musculus OX=10090 GN=Aars2 PE=1 SV=1	
G3X9B1_MOUSE	HEAT repeat-containing 1 OS=Mus musculus OX=10090 GN=Heatr1 PE=1 SV=1	
PLAK_MOUSE	Junction plakoglobin OS=Mus musculus OX=10090 GN=Jup PE=1 SV=3	
Q3TCI8_MOUSE	Glucosamine OS=Mus musculus OX=10090 GN=Gne PE=1 SV=1	
tr|Q3UL32|Q3UL32_MOUSE Uncharacterized protein OS=Mus musculus OX=10090 GN=Dnajc7...	Uncharacterized protein OS=Mus musculus OX=10090 GN=Dnajc7 PE=2 SV=1	
sp|Q9D024-2|CCD47_MOUSE	Isoform 2 of Coiled-coil domain-containing protein 47 OS=Mus musculus OX=10090 GN=Ccdc47	
TBG1_MOUSE	Tubulin gamma-1 chain OS=Mus musculus OX=10090 GN=Tubg1 PE=1 SV=1	
sp|Q921N6|DDX27_MOUSEProbable ATP-dependent RNA helicase DDX27 OS=Mus musculus OX=10090 GN=Ddx27 PE=1 SV=3	sp|Q921N6|DDX27_MOUSE Probable ATP-dependent RNA helicase DDX27 OS=Mus musculus OX=10090 GN=Ddx27 PE=1 SV=3	
HERC2_MOUSE	Isoform 2 of E3 ubiquitin-protein ligase HERC2 OS=Mus musculus OX=10090 GN=Herc2	
D3YUP1_MOUSE	Histone-arginine methyltransferase CARM1 OS=Mus musculus OX=10090 GN=Carm1 PE=1 SV=1	
E9Q6R3_MOUSE	Vesicle-trafficking protein SEC22b OS=Mus musculus OX=10090 GN=Sec22b PE=1 SV=1	
Q3ZAW8_MOUSE	Keratin 16 OS=Mus musculus OX=10090 GN=Krt16 PE=2 SV=1	
Q8BZZ0_MOUSE	Gamma-tubulin complex component OS=Mus musculus OX=10090 GN=Tubgcp2 PE=2 SV=1	
sp|E9Q8T7|DYH1_MOUSE Dynein heavy chain 1, axonemal OS=Mus musculus OX=10090 GN=Dnah1...	Dynein heavy chain 1, axonemal OS=Mus musculus OX=10090 GN=Dnah1 PE=1 SV=1	
sp|Q62009-4|POSTN_MOUSE	Isoform 4 of Periostin OS=Mus musculus OX=10090 GN=Postn	
tr|A2AUY4|A2AUY4_MOUSE-DECOY Bromodomain adjacent to zinc finger domain, 2B OS=Mus...	tr|A2AUY4|A2AUY4_MOUSE-DECOY Bromodomain adjacent to zinc finger domain, 2B OS=Mus...	
tr|B1ARU1|B1ARU1_MOUSE-DECOY Microtubule-actin cross-linking factor 1 OS=Mus musculus...	tr|B1ARU1|B1ARU1_MOUSE-DECOY Microtubule-actin cross-linking factor 1 OS=Mus musculus...	
tr|B9EKJ3|B9EKJ3_MOUSE-DECOY Mon2 protein OS=Mus musculus OX=10090 GN=Mon2 PE=1 SV=1	tr|B9EKJ3|B9EKJ3_MOUSE-DECOY Mon2 protein OS=Mus musculus OX=10090 GN=Mon2 PE=1 SV=1	
tr|E9Q740|E9Q740_MOUSE Signal recognition particle subunit SRP72 OS=Mus musculus...	Signal recognition particle subunit SRP72 OS=Mus musculus OX=10090 GN=Srp72 PE=1 SV=1	
tr|Q99J49|Q99J49_MOUSE Tubb2a protein (Fragment) OS=Mus musculus OX=10090 GN=Tubb2a...	Tubb2a protein (Fragment) OS=Mus musculus OX=10090 GN=Tubb2a PE=2 SV=1	
tr|W6PPR4|W6PPR4_MOUSE-DECOY 480-kDa ankyrinG OS=Mus musculus OX=10090 GN=Ank3 PE=4...	tr|W6PPR4|W6PPR4_MOUSE-DECOY 480-kDa ankyrinG OS=Mus musculus OX=10090 GN=Ank3 PE=4...	
A0A0R4J0S4_MOUSE	Lethal(2) giant larvae protein homolog 1 OS=Mus musculus OX=10090 GN=Llgl1 PE=1 SV=1	
sp|P49615|CDK5_MOUSE Cyclin-dependent-like kinase 5 OS=Mus musculus OX=10090 GN=Cdk5...	Cyclin-dependent-like kinase 5 OS=Mus musculus OX=10090 GN=Cdk5 PE=1 SV=1	
tr|A0A087WNX7|A0A087WNX7_MOUSE Snf2-related CREBBP activator protein OS=Mus musculus...	tr|A0A087WNX7|A0A087WNX7_MOUSE Snf2-related CREBBP activator protein OS=Mus musculus...	
tr|A0A0A6YWA9|A0A0A6YWA9_MOUSE Guanine nucleotide-binding protein G(i) subunit alpha-2...	tr|A0A0A6YWA9|A0A0A6YWA9_MOUSE Guanine nucleotide-binding protein G(i) subunit alpha-2...	
tr|Q3TUQ7|Q3TUQ7_MOUSE Non-specific serine/threonine protein kinase OS=Mus musculus...	Non-specific serine/threonine protein kinase OS=Mus musculus OX=10090 GN=Prkaa1 PE=1 SV=1	
E9Q1T3_MOUSE	Mitogen-activated protein kinase kinase kinase kinase OS=Mus musculus OX=10090 GN=Map4k5 PE=1 SV=1	
RL11_MOUSE	60S ribosomal protein L11 OS=Mus musculus OX=10090 GN=Rpl11 PE=1 SV=4	
tr|A2ANY6|A2ANY6_MOUSE-DECOY Midasin OS=Mus musculus OX=10090 GN=Mdn1 PE=1 SV=1	tr|A2ANY6|A2ANY6_MOUSE-DECOY Midasin OS=Mus musculus OX=10090 GN=Mdn1 PE=1 SV=1	
B2RXY7_MOUSE	Carbonyl reductase 1 OS=Mus musculus OX=10090 GN=Cbr1 PE=1 SV=1	
sp|D3Z7P3-2|GLSK_MOUSE	Isoform 2 of Glutaminase kidney isoform, mitochondrial OS=Mus musculus OX=10090 GN=Gls	
tr|A0A0G2JDI9|A0A0G2JDI9_MOUSE ATP-binding cassette sub-family D member 3 OS=Mus...	tr|A0A0G2JDI9|A0A0G2JDI9_MOUSE ATP-binding cassette sub-family D member 3 OS=Mus...	
sp|Q9JLB0-2|MPP6_MOUSE Isoform Alpha of MAGUK p55 subfamily member 6 OS=Mus musculus...	Isoform Alpha of MAGUK p55 subfamily member 6 OS=Mus musculus OX=10090 GN=Mpp6	
sp|Q8VHE6|DYH5_MOUSE-DECOY Dynein heavy chain 5, axonemal OS=Mus musculus OX=10090...	sp|Q8VHE6|DYH5_MOUSE-DECOY Dynein heavy chain 5, axonemal OS=Mus musculus OX=10090...	
tr|A0A087WNZ7|A0A087WNZ7_MOUSE E3 ubiquitin-protein ligase TRIP12 OS=Mus musculus...	E3 ubiquitin-protein ligase TRIP12 OS=Mus musculus OX=10090 GN=Trip12 PE=1 SV=1	
A0A0N4SV80_MOUSE	Zinc finger protein 638 OS=Mus musculus OX=10090 GN=Zfp638 PE=1 SV=1	
AINX_MOUSE	Alpha-internexin OS=Mus musculus OX=10090 GN=Ina PE=1 SV=3	
E9QNH6_MOUSE	Unconventional myosin-Ib OS=Mus musculus OX=10090 GN=Myo1b PE=1 SV=1	
sp|Q9JHR7|IDE_MOUSE Insulin-degrading enzyme OS=Mus musculus OX=10090 GN=Ide PE=1...	Insulin-degrading enzyme OS=Mus musculus OX=10090 GN=Ide PE=1 SV=1	
HAT1_MOUSE	Histone acetyltransferase type B catalytic subunit OS=Mus musculus OX=10090 GN=Hat1 PE=1 SV=1	
sp|Q62407|SPEG_MOUSE-DECOY Striated muscle-specific serine/threonine-protein kinase...	sp|Q62407|SPEG_MOUSE-DECOY Striated muscle-specific serine/threonine-protein kinase...	
Q3TW11_MOUSE	Signal transducer and activator of transcription OS=Mus musculus OX=10090 GN=Stat1 PE=2 SV=1	
sp|Q05512-2|MARK2_MOUSE Isoform 2 of Serine/threonine-protein kinase MARK2 OS=Mus...	sp|Q05512-2|MARK2_MOUSE Isoform 2 of Serine/threonine-protein kinase MARK2 OS=Mus...	
B2RY05_MOUSE	Pik3r4 protein OS=Mus musculus OX=10090 GN=Pik3r4 PE=2 SV=1	
PUM3_MOUSE	Pumilio homolog 3 OS=Mus musculus OX=10090 GN=Pum3 PE=1 SV=2	
Q3UXN3_MOUSE	Uncharacterized protein OS=Mus musculus OX=10090 GN=Cars PE=2 SV=1	
tr|J3QMX2|J3QMX2_MOUSE Transportin-1 (Fragment) OS=Mus musculus OX=10090 GN=Tnpo1...	Transportin-1 (Fragment) OS=Mus musculus OX=10090 GN=Tnpo1 PE=1 SV=1	
F7CDT0_MOUSE	NEDD8-conjugating enzyme Ubc12 (Fragment) OS=Mus musculus OX=10090 GN=Ube2m PE=1 SV=1	
A2A9P6_MOUSE	Cyclin-dependent kinase 11B OS=Mus musculus OX=10090 GN=Cdk11b PE=1 SV=1	
tr|Q3UHW2|Q3UHW2_MOUSE Uncharacterized protein OS=Mus musculus OX=10090 GN=Hsd17b4...	Uncharacterized protein OS=Mus musculus OX=10090 GN=Hsd17b4 PE=2 SV=1	
A0A140T8W1_MOUSE	Collagen alpha-5(VI) chain OS=Mus musculus OX=10090 GN=Col6a5 PE=1 SV=1	
sp|Q7TMY8-3|HUWE1_MOUSE-DECOY Isoform 3 of E3 ubiquitin-protein ligase HUWE1 OS=Mus...	sp|Q7TMY8-3|HUWE1_MOUSE-DECOY Isoform 3 of E3 ubiquitin-protein ligase HUWE1 OS=Mus...	
sp|Q7TPH6-2|MYCB2_MOUSE Isoform 2 of E3 ubiquitin-protein ligase MYCBP2 OS=Mus musculus...	Isoform 2 of E3 ubiquitin-protein ligase MYCBP2 OS=Mus musculus OX=10090 GN=Mycbp2	
sp|Q5SUR0|PUR4_MOUSE Phosphoribosylformylglycinamidine synthase OS=Mus musculus OX=10090...	Phosphoribosylformylglycinamidine synthase OS=Mus musculus OX=10090 GN=Pfas PE=1 SV=1	
Q561N4_MOUSE	MCG1032217 OS=Mus musculus OX=10090 GN=Ube2l3 PE=1 SV=1	
sp|Q4U2R1-2|HERC2_MOUSE-DECOY Isoform 2 of E3 ubiquitin-protein ligase HERC2 OS=Mus...	sp|Q4U2R1-2|HERC2_MOUSE-DECOY Isoform 2 of E3 ubiquitin-protein ligase HERC2 OS=Mus...	
BYST_MOUSE	Bystin OS=Mus musculus OX=10090 GN=Bysl PE=1 SV=3	
sp|Q61245-2|COBA1_MOUSE-DECOY Isoform Short of Collagen alpha-1(XI) chain OS=Mus...	sp|Q61245-2|COBA1_MOUSE-DECOY Isoform Short of Collagen alpha-1(XI) chain OS=Mus...	
sp|Q80U78-2|PUM1_MOUSE	Isoform 2 of Pumilio homolog 1 OS=Mus musculus OX=10090 GN=Pum1	
Q8C952_MOUSE	Flap endonuclease 1 OS=Mus musculus OX=10090 GN=Fen1 PE=2 SV=1	
FXR2_MOUSE	Fragile X mental retardation syndrome-related protein 2 OS=Mus musculus OX=10090 GN=Fxr2 PE=1 SV=1	
Q3TA68_MOUSE	WD repeat domain 36 OS=Mus musculus OX=10090 GN=Wdr36 PE=1 SV=1	
A1BN54_MOUSE	Alpha actinin 1a OS=Mus musculus OX=10090 GN=Actn1 PE=1 SV=1	
Q91XW2_MOUSE	Putative ubiquitin-specific protease OS=Mus musculus OX=10090 GN=Usp9y PE=2 SV=1	
PESC_MOUSE	Pescadillo homolog OS=Mus musculus OX=10090 GN=Pes1 PE=1 SV=1	
sp|P70303-2|PYRG2_MOUSE	Isoform 2 of CTP synthase 2 OS=Mus musculus OX=10090 GN=Ctps2	
sp|Q09XV5|CHD8_MOUSE Chromodomain-helicase-DNA-binding protein 8 OS=Mus musculus...	sp|Q09XV5|CHD8_MOUSE Chromodomain-helicase-DNA-binding protein 8 OS=Mus musculus...	
sp|O35218|CPSF2_MOUSECleavage and polyadenylation specificity factor subunit 2 OS=Mus musculus OX=10090 GN=Cpsf2 PE=1 SV=1	sp|O35218|CPSF2_MOUSE Cleavage and polyadenylation specificity factor subunit 2 OS=Mus musculus OX=10090 GN=Cpsf2 PE=1 SV=1	
tr|Q3TL79|Q3TL79_MOUSE Uncharacterized protein OS=Mus musculus OX=10090 GN=Ahsa1...	tr|Q3TL79|Q3TL79_MOUSE Uncharacterized protein OS=Mus musculus OX=10090 GN=Ahsa1...	
SNX27_MOUSE	Isoform 2 of Sorting nexin-27 OS=Mus musculus OX=10090 GN=Snx27	
A0A1C9IC75_MOUSE	ABCC4-N1 OS=Mus musculus OX=10090 GN=Abcc4 PE=2 SV=1	
tr|Q69ZD1|Q69ZD1_MOUSE MKIAA1699 protein (Fragment) OS=Mus musculus OX=10090 GN=Exoc4...	MKIAA1699 protein (Fragment) OS=Mus musculus OX=10090 GN=Exoc4 PE=2 SV=1	
sp|O54990-3|PROM1_MOUSE	Isoform 3 of Prominin-1 OS=Mus musculus OX=10090 GN=Prom1	
Q3TJS0_MOUSE	Uncharacterized protein OS=Mus musculus OX=10090 GN=Flot1 PE=2 SV=1	
B2RQ80_MOUSE	Tnik protein OS=Mus musculus OX=10090 GN=Tnik PE=1 SV=1	
sp|O08582|GTPB1_MOUSEGTP-binding protein 1 OS=Mus musculus OX=10090 GN=Gtpbp1 PE=1 SV=2	sp|O08582|GTPB1_MOUSE GTP-binding protein 1 OS=Mus musculus OX=10090 GN=Gtpbp1 PE=1 SV=2	
A2AQA9_MOUSE	Nebulin OS=Mus musculus OX=10090 GN=Neb PE=1 SV=1	
NAV3_MOUSE	Neuron navigator 3 OS=Mus musculus OX=10090 GN=Nav3 PE=1 SV=2	
B2RQA0_MOUSE	EG331392 protein OS=Mus musculus OX=10090 GN=Gm5124 PE=2 SV=1	
F7BGR7_MOUSE	Predicted gene 21992 OS=Mus musculus OX=10090 GN=Gm21992 PE=1 SV=1	
tr|A0A1B0GR11|A0A1B0GR11_MOUSE Transaldolase OS=Mus musculus OX=10090 GN=Taldo1 PE=1...	Transaldolase OS=Mus musculus OX=10090 GN=Taldo1 PE=1 SV=1	
tr|Q8CD98|Q8CD98_MOUSE Uncharacterized protein OS=Mus musculus OX=10090 GN=Pfkl PE=2...	tr|Q8CD98|Q8CD98_MOUSE Uncharacterized protein OS=Mus musculus OX=10090 GN=Pfkl PE=2...	
sp|Q8VDP4|CCAR2_MOUSECell cycle and apoptosis regulator protein 2 OS=Mus musculus OX=10090 GN=Ccar2 PE=1 SV=2	sp|Q8VDP4|CCAR2_MOUSE Cell cycle and apoptosis regulator protein 2 OS=Mus musculus OX=10090 GN=Ccar2 PE=1 SV=2	
sp|P01786|HVM17_MOUSEIg heavy chain V region MOPC 47A OS=Mus musculus OX=10090 PE=1 SV=1	sp|P01786|HVM17_MOUSE Ig heavy chain V region MOPC 47A OS=Mus musculus OX=10090 PE=1 SV=1	
sp|B2RR83|YTDC2_MOUSEProbable ATP-dependent RNA helicase YTHDC2 OS=Mus musculus OX=10090 GN=Ythdc2 PE=1 SV=1	sp|B2RR83|YTDC2_MOUSE Probable ATP-dependent RNA helicase YTHDC2 OS=Mus musculus OX=10090 GN=Ythdc2 PE=1 SV=1	
A0A1L1SS82_MOUSE	Coiled-coil domain-containing 7A OS=Mus musculus OX=10090 GN=Ccdc7a PE=4 SV=1	
sp|Q9Z1T6-1|FYV1_MOUSE	Isoform 2 of 1-phosphatidylinositol 3-phosphate 5-kinase OS=Mus musculus OX=10090 GN=Pikfyve	
B2RRH9_MOUSE	Guanine monphosphate synthetase OS=Mus musculus OX=10090 GN=Gmps PE=1 SV=1	
Q155P7_MOUSE	LEK1 OS=Mus musculus OX=10090 GN=Cenpf PE=1 SV=1	
B2RSI3_MOUSE	Poly [ADP-ribose] polymerase OS=Mus musculus OX=10090 GN=Parp14 PE=2 SV=1	
Q3TRH1_MOUSE	Uncharacterized protein (Fragment) OS=Mus musculus OX=10090 GN=Eif3a PE=2 SV=1	
A2AFQ2_MOUSE	3-hydroxyacyl-CoA dehydrogenase type-2 OS=Mus musculus OX=10090 GN=Hsd17b10 PE=1 SV=1	
sp|O35344|IMA4_MOUSE Importin subunit alpha-4 OS=Mus musculus OX=10090 GN=Kpna3 PE=1...	sp|O35344|IMA4_MOUSE Importin subunit alpha-4 OS=Mus musculus OX=10090 GN=Kpna3 PE=1...	
sp|O70546-2|KDM6A_MOUSE-DECOY Isoform 2 of Lysine-specific demethylase 6A OS=Mus...	sp|O70546-2|KDM6A_MOUSE-DECOY Isoform 2 of Lysine-specific demethylase 6A OS=Mus...	
sp|Q9QYX7-2|PCLO_MOUSE	Isoform 2 of Protein piccolo OS=Mus musculus OX=10090 GN=Pclo	
tr|Q3TXG1|Q3TXG1_MOUSE Mitogen-activated protein kinase kinase kinase 7 OS=Mus musculus...	tr|Q3TXG1|Q3TXG1_MOUSE Mitogen-activated protein kinase kinase kinase 7 OS=Mus musculus...	
sp|Q9Z1K7|APCL_MOUSE-DECOY Adenomatous polyposis coli protein 2 OS=Mus musculus OX=10090...	sp|Q9Z1K7|APCL_MOUSE-DECOY Adenomatous polyposis coli protein 2 OS=Mus musculus OX=10090...	
F8WI37_MOUSE	Histone-lysine N-methyltransferase 2C OS=Mus musculus OX=10090 GN=Kmt2c PE=1 SV=1	
Q8CD76_MOUSE	Kinesin light chain 1 OS=Mus musculus OX=10090 GN=Klc1 PE=1 SV=1	
tr|D3YYQ8|D3YYQ8_MOUSE Dynein, axonemal, heavy chain 10 OS=Mus musculus OX=10090...	tr|D3YYQ8|D3YYQ8_MOUSE Dynein, axonemal, heavy chain 10 OS=Mus musculus OX=10090...	
tr|Z4YJT3|Z4YJT3_MOUSE La-related protein 1 OS=Mus musculus OX=10090 GN=Larp1 PE=1...	tr|Z4YJT3|Z4YJT3_MOUSE La-related protein 1 OS=Mus musculus OX=10090 GN=Larp1 PE=1...	
sp|Q8BW94-2|DYH3_MOUSE-DECOY Isoform 2 of Dynein heavy chain 3, axonemal OS=Mus musculus...	sp|Q8BW94-2|DYH3_MOUSE-DECOY Isoform 2 of Dynein heavy chain 3, axonemal OS=Mus musculus...	
tr|B9EK93|B9EK93_MOUSE Insulin receptor substrate 4 OS=Mus musculus OX=10090 GN=Irs4...	tr|B9EK93|B9EK93_MOUSE Insulin receptor substrate 4 OS=Mus musculus OX=10090 GN=Irs4...	
tr|E9QAS5|E9QAS5_MOUSE-DECOY Chromodomain-helicase-DNA-binding protein 4 OS=Mus musculus...	tr|E9QAS5|E9QAS5_MOUSE-DECOY Chromodomain-helicase-DNA-binding protein 4 OS=Mus musculus...	
tr|Q8BK66|Q8BK66_MOUSE Uncharacterized protein OS=Mus musculus OX=10090 GN=Ddx56...	tr|Q8BK66|Q8BK66_MOUSE Uncharacterized protein OS=Mus musculus OX=10090 GN=Ddx56...	
RYR2_MOUSE	Ryanodine receptor 2 OS=Mus musculus OX=10090 GN=Ryr2 PE=1 SV=1	
PUR8_MOUSE	Adenylosuccinate lyase OS=Mus musculus OX=10090 GN=Adsl PE=1 SV=2	
E9PX48_MOUSE	Dedicator of cytokinesis protein 7 OS=Mus musculus OX=10090 GN=Dock7 PE=1 SV=2	
SC31A_MOUSE	Isoform 2 of Protein transport protein Sec31A OS=Mus musculus OX=10090 GN=Sec31a	
Q3UGZ4_MOUSE	Spectrin beta chain OS=Mus musculus OX=10090 GN=Sptbn2 PE=2 SV=1	
IPO11_MOUSE	Isoform 2 of Importin-11 OS=Mus musculus OX=10090 GN=Ipo11	
sp|Q7TPH6-2|MYCB2_MOUSE-DECOY Isoform 2 of E3 ubiquitin-protein ligase MYCBP2 OS=Mus...	sp|Q7TPH6-2|MYCB2_MOUSE-DECOY Isoform 2 of E3 ubiquitin-protein ligase MYCBP2 OS=Mus...	
A0A1L1SQ51_MOUSE	Talin-2 OS=Mus musculus OX=10090 GN=Tln2 PE=1 SV=1	
tr|Q3TQW0|Q3TQW0_MOUSE Uncharacterized protein (Fragment) OS=Mus musculus OX=10090...	tr|Q3TQW0|Q3TQW0_MOUSE Uncharacterized protein (Fragment) OS=Mus musculus OX=10090...	
E0CZE0_MOUSE	NEDD8-activating enzyme E1 regulatory subunit OS=Mus musculus OX=10090 GN=Nae1 PE=1 SV=1	
O08847_MOUSE	DNA-directed RNA polymerase subunit OS=Mus musculus OX=10090 GN=Polr2a PE=2 SV=1	
Q5DTH1_MOUSE	MKIAA4216 protein (Fragment) OS=Mus musculus OX=10090 GN=Ube3a PE=2 SV=1	
Q7TN20_MOUSE	Ythdf3 protein (Fragment) OS=Mus musculus OX=10090 GN=Ythdf3 PE=2 SV=1	
Q80UE3_MOUSE	Protein 4.1G OS=Mus musculus OX=10090 GN=Epb41l2 PE=2 SV=1	
sp|P11440|CDK1_MOUSE Cyclin-dependent kinase 1 OS=Mus musculus OX=10090 GN=Cdk1 PE=1...	sp|P11440|CDK1_MOUSE Cyclin-dependent kinase 1 OS=Mus musculus OX=10090 GN=Cdk1 PE=1...	
sp|Q8C8R3-2|ANK2_MOUSE-DECOY Isoform 2 of Ankyrin-2 OS=Mus musculus OX=10090 GN=Ank2	sp|Q8C8R3-2|ANK2_MOUSE-DECOY Isoform 2 of Ankyrin-2 OS=Mus musculus OX=10090 GN=Ank2	
tr|A0A0G2JF52|A0A0G2JF52_MOUSE Actin related protein 2/3 complex, subunit 1A, isoform...	tr|A0A0G2JF52|A0A0G2JF52_MOUSE Actin related protein 2/3 complex, subunit 1A, isoform...	
tr|A0A0G2JGX4|A0A0G2JGX4_MOUSE Sodium/potassium-transporting ATPase subunit alpha...	tr|A0A0G2JGX4|A0A0G2JGX4_MOUSE Sodium/potassium-transporting ATPase subunit alpha...	
tr|D3YU58|D3YU58_MOUSE Anaphase-promoting complex subunit 7 OS=Mus musculus OX=10090...	Anaphase-promoting complex subunit 7 OS=Mus musculus OX=10090 GN=Anapc7 PE=1 SV=1	
tr|E9Q3P4|E9Q3P4_MOUSE-DECOY Centromere protein F OS=Mus musculus OX=10090 GN=Cenpf...	tr|E9Q3P4|E9Q3P4_MOUSE-DECOY Centromere protein F OS=Mus musculus OX=10090 GN=Cenpf...	
tr|G3UWR2|G3UWR2_MOUSE GRB10-interacting GYF protein 2 OS=Mus musculus OX=10090 GN=Gigyf2...	tr|G3UWR2|G3UWR2_MOUSE GRB10-interacting GYF protein 2 OS=Mus musculus OX=10090 GN=Gigyf2...	
tr|Q6NZK2|Q6NZK2_MOUSE-DECOY NCK-associated protein 5 OS=Mus musculus OX=10090 GN=Nckap5...	tr|Q6NZK2|Q6NZK2_MOUSE-DECOY NCK-associated protein 5 OS=Mus musculus OX=10090 GN=Nckap5...	
A2AMU9_MOUSE	Ribosome assembly factor mrt4 OS=Mus musculus OX=10090 GN=Mrto4 PE=1 SV=1	
E9PV41_MOUSE	Protein diaphanous homolog 1 OS=Mus musculus OX=10090 GN=Diaph1 PE=1 SV=1	
E9Q043_MOUSE	Fibronectin type III domain-containing 1 OS=Mus musculus OX=10090 GN=Fndc1 PE=1 SV=1	
tr|Q571A1|Q571A1_MOUSE MKIAA0765 splice variant 1 (Fragment) OS=Mus musculus OX=10090...	tr|Q571A1|Q571A1_MOUSE MKIAA0765 splice variant 1 (Fragment) OS=Mus musculus OX=10090...	
A0A1W2P7Q6_MOUSE	Exportin-T OS=Mus musculus OX=10090 GN=Xpot PE=1 SV=1	
A7TU71_MOUSE	Protein Shroom2 OS=Mus musculus OX=10090 GN=Shroom2 PE=1 SV=1	
tr|A2A520|A2A520_MOUSE-DECOY Dynein heavy chain 17, axonemal OS=Mus musculus OX=10090...	tr|A2A520|A2A520_MOUSE-DECOY Dynein heavy chain 17, axonemal OS=Mus musculus OX=10090...	
tr|E9Q1M6|E9Q1M6_MOUSE Ankyrin repeat and KH domain-containing 1 OS=Mus musculus...	tr|E9Q1M6|E9Q1M6_MOUSE Ankyrin repeat and KH domain-containing 1 OS=Mus musculus...	
E9Q6A7_MOUSE	Bromodomain PHD finger transcription factor OS=Mus musculus OX=10090 GN=Bptf PE=1 SV=1	
Q9CWR5_MOUSE	Serine hydroxymethyltransferase OS=Mus musculus OX=10090 GN=Shmt1 PE=2 SV=1	
sp|Q78DX7|ROS1_MOUSE Proto-oncogene tyrosine-protein kinase ROS OS=Mus musculus OX=10090...	sp|Q78DX7|ROS1_MOUSE Proto-oncogene tyrosine-protein kinase ROS OS=Mus musculus OX=10090...	
D3Z667_MOUSE	Dynein heavy chain 2, axonemal OS=Mus musculus OX=10090 GN=Dnah2 PE=1 SV=1	
tr|Q5SUC3|Q5SUC3_MOUSE Calnexin, isoform CRA_a OS=Mus musculus OX=10090 GN=Canx PE=1...	tr|Q5SUC3|Q5SUC3_MOUSE Calnexin, isoform CRA_a OS=Mus musculus OX=10090 GN=Canx PE=1...	
sp|Q8C0C7|SYFA_MOUSE Phenylalanine--tRNA ligase alpha subunit OS=Mus musculus OX=10090...	sp|Q8C0C7|SYFA_MOUSE Phenylalanine--tRNA ligase alpha subunit OS=Mus musculus OX=10090...	
sp|Q8BP67|RL24_MOUSE 60S ribosomal protein L24 OS=Mus musculus OX=10090 GN=Rpl24...	sp|Q8BP67|RL24_MOUSE 60S ribosomal protein L24 OS=Mus musculus OX=10090 GN=Rpl24...	
A0A087WSE3_MOUSE	Striated muscle-specific serine/threonine-protein kinase (Fragment) OS=Mus musculus OX=10090 GN=Speg PE=1 SV=1	
sp|E9Q401|RYR2_MOUSE-DECOY Ryanodine receptor 2 OS=Mus musculus OX=10090 GN=Ryr2...	sp|E9Q401|RYR2_MOUSE-DECOY Ryanodine receptor 2 OS=Mus musculus OX=10090 GN=Ryr2...	
tr|D0VYV7|D0VYV7_MOUSE Erythrocyte protein band 4.1-like 3 isoform C OS=Mus musculus...	Erythrocyte protein band 4.1-like 3 isoform C OS=Mus musculus OX=10090 GN=Epb41l3 PE=2 SV=1	
tr|Q8BTJ1|Q8BTJ1_MOUSE Phosphoserine aminotransferase OS=Mus musculus OX=10090 GN=Psat1...	Phosphoserine aminotransferase OS=Mus musculus OX=10090 GN=Psat1 PE=2 SV=1	
Q3UDN8_MOUSE	Uncharacterized protein (Fragment) OS=Mus musculus OX=10090 GN=Trim28 PE=2 SV=1	
ATRN_MOUSE	Attractin OS=Mus musculus OX=10090 GN=Atrn PE=1 SV=3	
sp|Q9WVA4|TAGL2_MOUSETransgelin-2 OS=Mus musculus OX=10090 GN=Tagln2 PE=1 SV=4	sp|Q9WVA4|TAGL2_MOUSE Transgelin-2 OS=Mus musculus OX=10090 GN=Tagln2 PE=1 SV=4	
Q9D6F6_MOUSE	Dynein light chain OS=Mus musculus OX=10090 GN=Dynll1 PE=2 SV=1	
sp|Q60974-2|NCOR1_MOUSE-DECOY Isoform 2 of Nuclear receptor corepressor 1 OS=Mus...	sp|Q60974-2|NCOR1_MOUSE-DECOY Isoform 2 of Nuclear receptor corepressor 1 OS=Mus...	
NCPR_MOUSE	NADPH--cytochrome P450 reductase OS=Mus musculus OX=10090 GN=Por PE=1 SV=2	
DPP3_MOUSE	Dipeptidyl peptidase 3 OS=Mus musculus OX=10090 GN=Dpp3 PE=1 SV=2	
A0A0A6YX18_MOUSE	V-type proton ATPase subunit H OS=Mus musculus OX=10090 GN=Atp6v1h PE=1 SV=1	
tr|G5E8B1|G5E8B1_MOUSE-DECOY Protein tyrosine phosphatase, non-receptor type 13 OS=Mus...	tr|G5E8B1|G5E8B1_MOUSE-DECOY Protein tyrosine phosphatase, non-receptor type 13 OS=Mus...	
E9QK83_MOUSE	Small subunit processome component 20 homolog OS=Mus musculus OX=10090 GN=Utp20 PE=1 SV=1	
tr|B2RX08|B2RX08_MOUSE-DECOY Spectrin beta chain OS=Mus musculus OX=10090 GN=Sptb...	tr|B2RX08|B2RX08_MOUSE-DECOY Spectrin beta chain OS=Mus musculus OX=10090 GN=Sptb...	
A0A076N9U7_MOUSE	Celsr3 OS=Mus musculus OX=10090 GN=Celsr3 PE=2 SV=1	
A2A6U3_MOUSE	Septin-9 OS=Mus musculus OX=10090 GN=Sept9 PE=1 SV=1	
sp|E9PVX6-2|KI67_MOUSE Isoform 2 of Proliferation marker protein Ki-67 OS=Mus musculus...	Isoform 2 of Proliferation marker protein Ki-67 OS=Mus musculus OX=10090 GN=Mki67	
APCL_MOUSE	Adenomatous polyposis coli protein 2 OS=Mus musculus OX=10090 GN=Apc2 PE=1 SV=1	
sp|P46664|PURA2_MOUSEAdenylosuccinate synthetase isozyme 2 OS=Mus musculus OX=10090 GN=Adss PE=1 SV=2	sp|P46664|PURA2_MOUSE Adenylosuccinate synthetase isozyme 2 OS=Mus musculus OX=10090 GN=Adss PE=1 SV=2	
sp|Q9Z2U1|PSA5_MOUSE Proteasome subunit alpha type-5 OS=Mus musculus OX=10090 GN=Psma5...	sp|Q9Z2U1|PSA5_MOUSE Proteasome subunit alpha type-5 OS=Mus musculus OX=10090 GN=Psma5...	
VP13A_MOUSE	Isoform 2 of Vacuolar protein sorting-associated protein 13A OS=Mus musculus OX=10090 GN=Vps13a	
E9QM38_MOUSE	Solute carrier family 12 member 2 OS=Mus musculus OX=10090 GN=Slc12a2 PE=1 SV=1	
sp|Q9CSH3|RRP44_MOUSEExosome complex exonuclease RRP44 OS=Mus musculus OX=10090 GN=Dis3 PE=1 SV=4	sp|Q9CSH3|RRP44_MOUSE Exosome complex exonuclease RRP44 OS=Mus musculus OX=10090 GN=Dis3 PE=1 SV=4	
A0A1W2P6U8_MOUSE	RIKEN cDNA 4932415D10 gene OS=Mus musculus OX=10090 GN=4932415D10Rik PE=4 SV=1	
CAP1_MOUSE	Adenylyl cyclase-associated protein 1 OS=Mus musculus OX=10090 GN=Cap1 PE=1 SV=4	
sp|Q9CZS1|AL1B1_MOUSEAldehyde dehydrogenase X, mitochondrial OS=Mus musculus OX=10090 GN=Aldh1b1 PE=1 SV=1	sp|Q9CZS1|AL1B1_MOUSE Aldehyde dehydrogenase X, mitochondrial OS=Mus musculus OX=10090 GN=Aldh1b1 PE=1 SV=1	
Q3U1K2_MOUSE	Uncharacterized protein (Fragment) OS=Mus musculus OX=10090 GN=Atp2a3 PE=2 SV=1	
sp|P27641|XRCC5_MOUSEX-ray repair cross-complementing protein 5 OS=Mus musculus OX=10090 GN=Xrcc5 PE=1 SV=4	sp|P27641|XRCC5_MOUSE X-ray repair cross-complementing protein 5 OS=Mus musculus OX=10090 GN=Xrcc5 PE=1 SV=4	
tr|E9Q0B6|E9Q0B6_MOUSE-DECOY Dynein, axonemal, heavy chain 6 OS=Mus musculus OX=10090...	tr|E9Q0B6|E9Q0B6_MOUSE-DECOY Dynein, axonemal, heavy chain 6 OS=Mus musculus OX=10090...	
ARF5_MOUSE	ADP-ribosylation factor 5 OS=Mus musculus OX=10090 GN=Arf5 PE=1 SV=2	
NXN_MOUSE	Nucleoredoxin OS=Mus musculus OX=10090 GN=Nxn PE=1 SV=1	
sp|P48678|LMNA_MOUSE	Prelamin-A/C OS=Mus musculus OX=10090 GN=Lmna PE=1 SV=2	
sp|P97313-2|PRKDC_MOUSE Isoform 2 of DNA-dependent protein kinase catalytic subunit...	sp|P97313-2|PRKDC_MOUSE Isoform 2 of DNA-dependent protein kinase catalytic subunit...	
sp|Q6P2B1-2|TNPO3_MOUSE	Isoform 2 of Transportin-3 OS=Mus musculus OX=10090 GN=Tnpo3	
tr|E9Q8T1|E9Q8T1_MOUSE Transforming acidic coiled-coil-containing protein 2 OS=Mus...	tr|E9Q8T1|E9Q8T1_MOUSE Transforming acidic coiled-coil-containing protein 2 OS=Mus...	
tr|A2BH46|A2BH46_MOUSE RNA cytidine acetyltransferase (Fragment) OS=Mus musculus...	tr|A2BH46|A2BH46_MOUSE RNA cytidine acetyltransferase (Fragment) OS=Mus musculus...	
sp|P70333|HNRH2_MOUSEHeterogeneous nuclear ribonucleoprotein H2 OS=Mus musculus OX=10090 GN=Hnrnph2 PE=1 SV=1	sp|P70333|HNRH2_MOUSE Heterogeneous nuclear ribonucleoprotein H2 OS=Mus musculus OX=10090 GN=Hnrnph2 PE=1 SV=1	
VPP1_MOUSE	V-type proton ATPase 116 kDa subunit a isoform 1 OS=Mus musculus OX=10090 GN=Atp6v0a1 PE=1 SV=3	
B2RX66_MOUSE	MCG124812 OS=Mus musculus OX=10090 GN=Taok1 PE=1 SV=1	
ECHA_MOUSE	Trifunctional enzyme subunit alpha, mitochondrial OS=Mus musculus OX=10090 GN=Hadha PE=1 SV=1	
A0A1B0GR10_MOUSE	Predicted gene, 40460 OS=Mus musculus OX=10090 GN=Gm40460 PE=4 SV=1	
tr|E9PWM3|E9PWM3_MOUSE-DECOY Armadillo repeat-containing, X-linked 4 OS=Mus musculus...	tr|E9PWM3|E9PWM3_MOUSE-DECOY Armadillo repeat-containing, X-linked 4 OS=Mus musculus...	
tr|A0A1B0GS68|A0A1B0GS68_MOUSE Predicted gene 45713 OS=Mus musculus OX=10090 GN=Gm45713...	tr|A0A1B0GS68|A0A1B0GS68_MOUSE Predicted gene 45713 OS=Mus musculus OX=10090 GN=Gm45713...	
Q8BYP9_MOUSE	Uncharacterized protein OS=Mus musculus OX=10090 GN=Atl2 PE=2 SV=1	
E9Q9C6_MOUSE	Fc fragment of IgG-binding protein OS=Mus musculus OX=10090 GN=Fcgbp PE=1 SV=1	
sp|Q01320|TOP2A_MOUSEDNA topoisomerase 2-alpha OS=Mus musculus OX=10090 GN=Top2a PE=1 SV=2	sp|Q01320|TOP2A_MOUSE DNA topoisomerase 2-alpha OS=Mus musculus OX=10090 GN=Top2a PE=1 SV=2	
E9QJU8_MOUSE	Transformation-related protein 53-binding protein 2 OS=Mus musculus OX=10090 GN=Trp53bp2 PE=1 SV=1	
A0A1B0GT92_MOUSE	Glycogen [starch] synthase OS=Mus musculus OX=10090 GN=Gys1 PE=1 SV=1	
S4R2J9_MOUSE	Protein PRRC2C OS=Mus musculus OX=10090 GN=Prrc2c PE=1 SV=1	
ELP1_MOUSE	Elongator complex protein 1 OS=Mus musculus OX=10090 GN=Elp1 PE=1 SV=2	
A0A087WQS2_MOUSE	Basic leucine zipper and W2 domain-containing protein 1 OS=Mus musculus OX=10090 GN=Bzw1 PE=1 SV=1	
E9QPE7_MOUSE	Myosin-11 OS=Mus musculus OX=10090 GN=Myh11 PE=1 SV=1	
ACACA_MOUSE	Isoform 2 of Acetyl-CoA carboxylase 1 OS=Mus musculus OX=10090 GN=Acaca	
Q6PFV8_MOUSE	Cacna1g protein OS=Mus musculus OX=10090 GN=Cacna1g PE=2 SV=1	
sp|P97384|ANX11_MOUSEAnnexin A11 OS=Mus musculus OX=10090 GN=Anxa11 PE=1 SV=2	sp|P97384|ANX11_MOUSE Annexin A11 OS=Mus musculus OX=10090 GN=Anxa11 PE=1 SV=2	
C7G3P2_MOUSE	MKIAA0536 protein (Fragment) OS=Mus musculus OX=10090 GN=Prpf4b PE=2 SV=1	
Q3UAY3_MOUSE	Uncharacterized protein OS=Mus musculus OX=10090 GN=Gtf3c4 PE=2 SV=1	
sp|P56480|ATPB_MOUSE ATP synthase subunit beta, mitochondrial OS=Mus musculus OX=10090...	ATP synthase subunit beta, mitochondrial OS=Mus musculus OX=10090 GN=Atp5f1b PE=1 SV=2	
sp|A2AWL7-2|MGAP_MOUSE-DECOY Isoform 2 of MAX gene-associated protein OS=Mus musculus...	sp|A2AWL7-2|MGAP_MOUSE-DECOY Isoform 2 of MAX gene-associated protein OS=Mus musculus...	
sp|Q8CGC7|SYEP_MOUSE-DECOY Bifunctional glutamate/proline--tRNA ligase OS=Mus musculus...	sp|Q8CGC7|SYEP_MOUSE-DECOY Bifunctional glutamate/proline--tRNA ligase OS=Mus musculus...	
E9QAE4_MOUSE	Histone-lysine N-methyltransferase, H3 lysine-36 and H4 lysine-20-specific OS=Mus musculus OX=10090 GN=Nsd1 PE=1 SV=1	
sp|Q91XQ0-2|DYH8_MOUSE Isoform 2 of Dynein heavy chain 8, axonemal OS=Mus musculus...	sp|Q91XQ0-2|DYH8_MOUSE Isoform 2 of Dynein heavy chain 8, axonemal OS=Mus musculus...	
tr|E9Q526|E9Q526_MOUSE-DECOY C2 domain-containing protein 3 OS=Mus musculus OX=10090...	tr|E9Q526|E9Q526_MOUSE-DECOY C2 domain-containing protein 3 OS=Mus musculus OX=10090...	
tr|K3W4Q5|K3W4Q5_MOUSE-DECOY Family with sequence similarity 186, member A OS=Mus...	tr|K3W4Q5|K3W4Q5_MOUSE-DECOY Family with sequence similarity 186, member A OS=Mus...	
tr|Q69Z43|Q69Z43_MOUSE MKIAA1995 protein (Fragment) OS=Mus musculus OX=10090 GN=Nek9...	tr|Q69Z43|Q69Z43_MOUSE MKIAA1995 protein (Fragment) OS=Mus musculus OX=10090 GN=Nek9...	
sp|O54988|SLK_MOUSE STE20-like serine/threonine-protein kinase OS=Mus musculus OX=10090...	sp|O54988|SLK_MOUSE STE20-like serine/threonine-protein kinase OS=Mus musculus OX=10090...	
sp|P68404|KPCB_MOUSE	Protein kinase C beta type OS=Mus musculus OX=10090 GN=Prkcb PE=1 SV=4	
sp|Q6PR54-3|RIF1_MOUSE-DECOY Isoform 3 of Telomere-associated protein RIF1 OS=Mus...	sp|Q6PR54-3|RIF1_MOUSE-DECOY Isoform 3 of Telomere-associated protein RIF1 OS=Mus...	
A0A2I3BR78_MOUSE	Exosome complex exonuclease RRP44 OS=Mus musculus OX=10090 GN=Dis3 PE=1 SV=1	
A2AL50_MOUSE	Alkylglycerone-phosphate synthase OS=Mus musculus OX=10090 GN=Agps PE=1 SV=1	
E9Q855_MOUSE	Secretory carrier-associated membrane protein OS=Mus musculus OX=10090 GN=Scamp3 PE=1 SV=1	
GLE1_MOUSE	Nucleoporin GLE1 OS=Mus musculus OX=10090 GN=Gle1 PE=1 SV=2	
sp|A2AAE1-6|K1109_MOUSE	Isoform 6 of Uncharacterized protein KIAA1109 OS=Mus musculus OX=10090 GN=Kiaa1109	
sp|O35654|DPOD2_MOUSEDNA polymerase delta subunit 2 OS=Mus musculus OX=10090 GN=Pold2 PE=1 SV=2	sp|O35654|DPOD2_MOUSE DNA polymerase delta subunit 2 OS=Mus musculus OX=10090 GN=Pold2 PE=1 SV=2	
sp|Q3TDN2-2|FAF2_MOUSE Isoform 2 of FAS-associated factor 2 OS=Mus musculus OX=10090...	sp|Q3TDN2-2|FAF2_MOUSE Isoform 2 of FAS-associated factor 2 OS=Mus musculus OX=10090...	
sp|Q61315|APC_MOUSE-DECOY Adenomatous polyposis coli protein OS=Mus musculus OX=10090...	sp|Q61315|APC_MOUSE-DECOY Adenomatous polyposis coli protein OS=Mus musculus OX=10090...	
sp|Q62440-2|TLE1_MOUSE Isoform 2 of Transducin-like enhancer protein 1 OS=Mus musculus...	sp|Q62440-2|TLE1_MOUSE Isoform 2 of Transducin-like enhancer protein 1 OS=Mus musculus...	
sp|Q64521|GPDM_MOUSE Glycerol-3-phosphate dehydrogenase, mitochondrial OS=Mus musculus...	sp|Q64521|GPDM_MOUSE Glycerol-3-phosphate dehydrogenase, mitochondrial OS=Mus musculus...	
sp|Q99J95-2|CDK9_MOUSE Isoform 2 of Cyclin-dependent kinase 9 OS=Mus musculus OX=10090...	Isoform 2 of Cyclin-dependent kinase 9 OS=Mus musculus OX=10090 GN=Cdk9	
tr|A0A0N4SVC2|A0A0N4SVC2_MOUSE Transformer-2 protein homolog alpha OS=Mus musculus...	tr|A0A0N4SVC2|A0A0N4SVC2_MOUSE Transformer-2 protein homolog alpha OS=Mus musculus...	
tr|A0A1L1SQ51|A0A1L1SQ51_MOUSE-DECOY Talin-2 OS=Mus musculus OX=10090 GN=Tln2 PE=1...	tr|A0A1L1SQ51|A0A1L1SQ51_MOUSE-DECOY Talin-2 OS=Mus musculus OX=10090 GN=Tln2 PE=1...	
tr|Q3UNM2|Q3UNM2_MOUSE Uncharacterized protein (Fragment) OS=Mus musculus OX=10090...	tr|Q3UNM2|Q3UNM2_MOUSE Uncharacterized protein (Fragment) OS=Mus musculus OX=10090...	
tr|Q80ZU1|Q80ZU1_MOUSE WD repeat domain 68 OS=Mus musculus OX=10090 GN=Dcaf7 PE=2...	WD repeat domain 68 OS=Mus musculus OX=10090 GN=Dcaf7 PE=2 SV=1	
sp|Q921N6-2|DDX27_MOUSE Isoform 2 of Probable ATP-dependent RNA helicase DDX27 OS=Mus...	sp|Q921N6-2|DDX27_MOUSE Isoform 2 of Probable ATP-dependent RNA helicase DDX27 OS=Mus...	
tr|F6TN03|F6TN03_MOUSE Ribosome maturation protein SBDS (Fragment) OS=Mus musculus...	Ribosome maturation protein SBDS (Fragment) OS=Mus musculus OX=10090 GN=Sbds PE=1 SV=1	
COG5_MOUSE	Conserved oligomeric Golgi complex subunit 5 OS=Mus musculus OX=10090 GN=Cog5 PE=1 SV=3	
sp|E9Q5F9-2|SETD2_MOUSE Isoform 2 of Histone-lysine N-methyltransferase SETD2 OS=Mus...	Isoform 2 of Histone-lysine N-methyltransferase SETD2 OS=Mus musculus OX=10090 GN=Setd2	
tr|Q8BL36|Q8BL36_MOUSE Uncharacterized protein OS=Mus musculus OX=10090 GN=Fam98a...	tr|Q8BL36|Q8BL36_MOUSE Uncharacterized protein OS=Mus musculus OX=10090 GN=Fam98a...	
tr|Q7TNG4|Q7TNG4_MOUSE Smc1a protein (Fragment) OS=Mus musculus OX=10090 GN=Smc1a...	tr|Q7TNG4|Q7TNG4_MOUSE Smc1a protein (Fragment) OS=Mus musculus OX=10090 GN=Smc1a...	
Q3TI21_MOUSE	Uncharacterized protein OS=Mus musculus OX=10090 GN=Ddx41 PE=2 SV=1	
sp|P27612|PLAP_MOUSE Phospholipase A-2-activating protein OS=Mus musculus OX=10090...	sp|P27612|PLAP_MOUSE Phospholipase A-2-activating protein OS=Mus musculus OX=10090...	
Q3UI30_MOUSE	Uncharacterized protein OS=Mus musculus OX=10090 GN=Ewsr1 PE=2 SV=1	
sp|Q8VEK3-2|HNRPU_MOUSE Isoform 2 of Heterogeneous nuclear ribonucleoprotein U OS=Mus...	Isoform 2 of Heterogeneous nuclear ribonucleoprotein U OS=Mus musculus OX=10090 GN=Hnrnpu	
tr|D3YWC5|D3YWC5_MOUSE Cullin-associated NEDD8-dissociated protein 1 (Fragment) OS=Mus...	Cullin-associated NEDD8-dissociated protein 1 (Fragment) OS=Mus musculus OX=10090 GN=Cand1 PE=1 SV=1	
EP400_MOUSE	Isoform 4 of E1A-binding protein p400 OS=Mus musculus OX=10090 GN=Ep400	
tr|Q9CX79|Q9CX79_MOUSE Uncharacterized protein OS=Mus musculus OX=10090 GN=Ntpcr...	tr|Q9CX79|Q9CX79_MOUSE Uncharacterized protein OS=Mus musculus OX=10090 GN=Ntpcr...	
tr|Q6SLK2|Q6SLK2_MOUSE Protein kinase lysine deficient 1 OS=Mus musculus OX=10090...	tr|Q6SLK2|Q6SLK2_MOUSE Protein kinase lysine deficient 1 OS=Mus musculus OX=10090...	
A8DUV3_MOUSE	Alpha-globin OS=Mus musculus OX=10090 GN=Hbat1 PE=3 SV=1	
sp|Q61687|ATRX_MOUSE-DECOY Transcriptional regulator ATRX OS=Mus musculus OX=10090...	sp|Q61687|ATRX_MOUSE-DECOY Transcriptional regulator ATRX OS=Mus musculus OX=10090...	
tr|F8WGX5|F8WGX5_MOUSE-DECOY DmX-like protein 1 OS=Mus musculus OX=10090 GN=Dmxl1...	tr|F8WGX5|F8WGX5_MOUSE-DECOY DmX-like protein 1 OS=Mus musculus OX=10090 GN=Dmxl1...	
A0A0R4J1Z3_MOUSE	Transmembrane protein 33 OS=Mus musculus OX=10090 GN=Tmem33 PE=1 SV=1	
tr|Q8BWM7|Q8BWM7_MOUSE Uncharacterized protein (Fragment) OS=Mus musculus OX=10090...	tr|Q8BWM7|Q8BWM7_MOUSE Uncharacterized protein (Fragment) OS=Mus musculus OX=10090...	
tr|E9Q1M6|E9Q1M6_MOUSE-DECOY Ankyrin repeat and KH domain-containing 1 OS=Mus musculus...	tr|E9Q1M6|E9Q1M6_MOUSE-DECOY Ankyrin repeat and KH domain-containing 1 OS=Mus musculus...	
tr|A0A1Y7VLA4|A0A1Y7VLA4_MOUSE-DECOY Protein FAM208B (Fragment) OS=Mus musculus OX=10090...	tr|A0A1Y7VLA4|A0A1Y7VLA4_MOUSE-DECOY Protein FAM208B (Fragment) OS=Mus musculus OX=10090...	
sp|Q6IFX2|K1C42_MOUSEKeratin, type I cytoskeletal 42 OS=Mus musculus OX=10090 GN=Krt42 PE=1 SV=1	sp|Q6IFX2|K1C42_MOUSE Keratin, type I cytoskeletal 42 OS=Mus musculus OX=10090 GN=Krt42 PE=1 SV=1	
tr|E9QPI5|E9QPI5_MOUSE Sister chromatid cohesion protein PDS5 homolog A OS=Mus musculus...	Sister chromatid cohesion protein PDS5 homolog A OS=Mus musculus OX=10090 GN=Pds5a PE=1 SV=1	
Q3TLF6_MOUSE	Uncharacterized protein OS=Mus musculus OX=10090 GN=Mybbp1a PE=2 SV=1	
tr|E9PZP8|E9PZP8_MOUSE-DECOY HECT and RLD domain-containing E3 ubiquitin protein...	tr|E9PZP8|E9PZP8_MOUSE-DECOY HECT and RLD domain-containing E3 ubiquitin protein...	
E9PWN2_MOUSE	Isoleucine--tRNA ligase, mitochondrial OS=Mus musculus OX=10090 GN=Iars2 PE=1 SV=1	
A0A0R4J1D0_MOUSE	Copine-2 OS=Mus musculus OX=10090 GN=Cpne2 PE=1 SV=1	
Q3TKX4_MOUSE	Uncharacterized protein (Fragment) OS=Mus musculus OX=10090 GN=Ddx52 PE=2 SV=1	
Q05CX1_MOUSE	Mgea5 protein (Fragment) OS=Mus musculus OX=10090 GN=Mgea5 PE=2 SV=1	
A2ACG7_MOUSE	Dolichyl-diphosphooligosaccharide--protein glycosyltransferase subunit 2 OS=Mus musculus OX=10090 GN=Rpn2 PE=1 SV=1	
TXTP_MOUSE	Tricarboxylate transport protein, mitochondrial OS=Mus musculus OX=10090 GN=Slc25a1 PE=1 SV=1	
A1A596_MOUSE	Pwp2 protein OS=Mus musculus OX=10090 GN=Pwp2 PE=2 SV=1	
Q9CS46_MOUSE	Uncharacterized protein (Fragment) OS=Mus musculus OX=10090 GN=Ilkap PE=2 SV=1	
Q3TJD0_MOUSE	Uncharacterized protein OS=Mus musculus OX=10090 GN=Sec61a1 PE=2 SV=1	
NOL8_MOUSE	Nucleolar protein 8 OS=Mus musculus OX=10090 GN=Nol8 PE=1 SV=2	
tr|A2AFF6|A2AFF6_MOUSE Cohesin subunit SA-2 OS=Mus musculus OX=10090 GN=Stag2 PE=1...	Cohesin subunit SA-2 OS=Mus musculus OX=10090 GN=Stag2 PE=1 SV=1	
Q3TFH8_MOUSE	Phosphoacetylglucosamine mutase OS=Mus musculus OX=10090 GN=Pgm3 PE=2 SV=1	
tr|B1AQ77|B1AQ77_MOUSE Keratin 15, isoform CRA_a OS=Mus musculus OX=10090 GN=Krt15...	Keratin 15, isoform CRA_a OS=Mus musculus OX=10090 GN=Krt15 PE=1 SV=1	
sp|Q9ESW4|AGK_MOUSE Acylglycerol kinase, mitochondrial OS=Mus musculus OX=10090 GN=Agk...	sp|Q9ESW4|AGK_MOUSE Acylglycerol kinase, mitochondrial OS=Mus musculus OX=10090 GN=Agk...	
tr|F8VPZ5|F8VPZ5_MOUSE-DECOY Excision repair cross-complementing rodent repair deficiency,...	tr|F8VPZ5|F8VPZ5_MOUSE-DECOY Excision repair cross-complementing rodent repair deficiency,...	
sp|Q3TIX9|SNUT2_MOUSEU4/U6.U5 tri-snRNP-associated protein 2 OS=Mus musculus OX=10090 GN=Usp39 PE=1 SV=2	sp|Q3TIX9|SNUT2_MOUSE U4/U6.U5 tri-snRNP-associated protein 2 OS=Mus musculus OX=10090 GN=Usp39 PE=1 SV=2	
tr|A0A0R4J0B7|A0A0R4J0B7_MOUSE Protein SDA1 homolog OS=Mus musculus OX=10090 GN=Sdad1...	Protein SDA1 homolog OS=Mus musculus OX=10090 GN=Sdad1 PE=1 SV=1	
Q3TEB0_MOUSE	Uncharacterized protein OS=Mus musculus OX=10090 GN=Pspc1 PE=2 SV=1	
tr|D3Z2X2|D3Z2X2_MOUSE Dynein heavy chain domain 1 OS=Mus musculus OX=10090 GN=Dnhd1...	tr|D3Z2X2|D3Z2X2_MOUSE Dynein heavy chain domain 1 OS=Mus musculus OX=10090 GN=Dnhd1...	
tr|E9Q264|E9Q264_MOUSE-DECOY Myosin, heavy chain 15 OS=Mus musculus OX=10090 GN=Myh15...	tr|E9Q264|E9Q264_MOUSE-DECOY Myosin, heavy chain 15 OS=Mus musculus OX=10090 GN=Myh15...	
tr|D6RIN1|D6RIN1_MOUSE Protein unc-45 homolog A OS=Mus musculus OX=10090 GN=Unc45a...	tr|D6RIN1|D6RIN1_MOUSE Protein unc-45 homolog A OS=Mus musculus OX=10090 GN=Unc45a...	
tr|A0A0G2JEA5|A0A0G2JEA5_MOUSE Predicted gene 43738 OS=Mus musculus OX=10090 GN=Gm43738...	tr|A0A0G2JEA5|A0A0G2JEA5_MOUSE Predicted gene 43738 OS=Mus musculus OX=10090 GN=Gm43738...	
RICTR_MOUSE	Isoform 2 of Rapamycin-insensitive companion of mTOR OS=Mus musculus OX=10090 GN=Rictor	
tr|Q8C872|Q8C872_MOUSE Transferrin receptor protein 1 OS=Mus musculus OX=10090 GN=Tfrc...	Transferrin receptor protein 1 OS=Mus musculus OX=10090 GN=Tfrc PE=1 SV=1	
tr|H9KV04|H9KV04_MOUSE Mini-chromosome maintenance complex-binding protein OS=Mus...	tr|H9KV04|H9KV04_MOUSE Mini-chromosome maintenance complex-binding protein OS=Mus...	
A0A087WRK0_MOUSE	CLIP-associating protein 1 OS=Mus musculus OX=10090 GN=Clasp1 PE=1 SV=2	
SIN3A_MOUSE	Isoform 2 of Paired amphipathic helix protein Sin3a OS=Mus musculus OX=10090 GN=Sin3a	
sp|Q3UYV9|NCBP1_MOUSENuclear cap-binding protein subunit 1 OS=Mus musculus OX=10090 GN=Ncbp1 PE=1 SV=2	sp|Q3UYV9|NCBP1_MOUSE Nuclear cap-binding protein subunit 1 OS=Mus musculus OX=10090 GN=Ncbp1 PE=1 SV=2	
sp|A2A6Q5-3|CDC27_MOUSE	Isoform 3 of Cell division cycle protein 27 homolog OS=Mus musculus OX=10090 GN=Cdc27	
sp|Q9D6F9|TBB4A_MOUSETubulin beta-4A chain OS=Mus musculus OX=10090 GN=Tubb4a PE=1 SV=3	sp|Q9D6F9|TBB4A_MOUSE Tubulin beta-4A chain OS=Mus musculus OX=10090 GN=Tubb4a PE=1 SV=3	
tr|A0A0R4J0K2|A0A0R4J0K2_MOUSE-DECOY Cytoskeleton-associated protein 5 OS=Mus musculus...	tr|A0A0R4J0K2|A0A0R4J0K2_MOUSE-DECOY Cytoskeleton-associated protein 5 OS=Mus musculus...	
tr|B7ZP20|B7ZP20_MOUSE Ankyrin repeat and FYVE domain containing 1 OS=Mus musculus...	tr|B7ZP20|B7ZP20_MOUSE Ankyrin repeat and FYVE domain containing 1 OS=Mus musculus...	
sp|P68510|1433F_MOUSE14-3-3 protein eta OS=Mus musculus OX=10090 GN=Ywhah PE=1 SV=2	sp|P68510|1433F_MOUSE 14-3-3 protein eta OS=Mus musculus OX=10090 GN=Ywhah PE=1 SV=2	
sp|Q9EQF6|DPYL5_MOUSEDihydropyrimidinase-related protein 5 OS=Mus musculus OX=10090 GN=Dpysl5 PE=1 SV=1	sp|Q9EQF6|DPYL5_MOUSE Dihydropyrimidinase-related protein 5 OS=Mus musculus OX=10090 GN=Dpysl5 PE=1 SV=1	
sp|Q80TN7|NAV3_MOUSE-DECOY Neuron navigator 3 OS=Mus musculus OX=10090 GN=Nav3 PE=1...	sp|Q80TN7|NAV3_MOUSE-DECOY Neuron navigator 3 OS=Mus musculus OX=10090 GN=Nav3 PE=1...	
Q8BP60_MOUSE	Uncharacterized protein OS=Mus musculus OX=10090 PE=2 SV=1	
tr|A0A1B0GT70|A0A1B0GT70_MOUSE Erlin-2 (Fragment) OS=Mus musculus OX=10090 GN=Erlin2...	tr|A0A1B0GT70|A0A1B0GT70_MOUSE Erlin-2 (Fragment) OS=Mus musculus OX=10090 GN=Erlin2...	
D4AFX7_MOUSE	DnaJ heat shock protein family (Hsp40) member C13 OS=Mus musculus OX=10090 GN=Dnajc13 PE=1 SV=1	
sp|Q5SUF2-2|LC7L3_MOUSE Isoform 2 of Luc7-like protein 3 OS=Mus musculus OX=10090...	Isoform 2 of Luc7-like protein 3 OS=Mus musculus OX=10090 GN=Luc7l3	
sp|Q8K4Z5|SF3A1_MOUSESplicing factor 3A subunit 1 OS=Mus musculus OX=10090 GN=Sf3a1 PE=1 SV=1	sp|Q8K4Z5|SF3A1_MOUSE Splicing factor 3A subunit 1 OS=Mus musculus OX=10090 GN=Sf3a1 PE=1 SV=1	
sp|Q9JIX8-4|ACINU_MOUSE Isoform 4 of Apoptotic chromatin condensation inducer in...	Isoform 4 of Apoptotic chromatin condensation inducer in the nucleus OS=Mus musculus OX=10090 GN=Acin1	
tr|F8VPN4|F8VPN4_MOUSE Amylo-1,6-glucosidase, 4-alpha-glucanotransferase OS=Mus musculus...	tr|F8VPN4|F8VPN4_MOUSE Amylo-1,6-glucosidase, 4-alpha-glucanotransferase OS=Mus musculus...	
APT_MOUSE	Adenine phosphoribosyltransferase OS=Mus musculus OX=10090 GN=Aprt PE=1 SV=2	
tr|Q14C24|Q14C24_MOUSE MCG14259, isoform CRA_a OS=Mus musculus OX=10090 GN=U2af1...	tr|Q14C24|Q14C24_MOUSE MCG14259, isoform CRA_a OS=Mus musculus OX=10090 GN=U2af1...	
sp|P33609|DPOLA_MOUSEDNA polymerase alpha catalytic subunit OS=Mus musculus OX=10090 GN=Pola1 PE=1 SV=2	sp|P33609|DPOLA_MOUSE DNA polymerase alpha catalytic subunit OS=Mus musculus OX=10090 GN=Pola1 PE=1 SV=2	
E9Q1Z0_MOUSE	Keratin 90 OS=Mus musculus OX=10090 GN=Krt90 PE=1 SV=1	
I2BP1_MOUSE	Isoform 2 of Interferon regulatory factor 2-binding protein 1 OS=Mus musculus OX=10090 GN=Irf2bp1	
Q5RL55_MOUSE	ATP-binding cassette, sub-family F (GCN20), member 1 OS=Mus musculus OX=10090 GN=Abcf1 PE=2 SV=1	
sp|Q6ZPU9|KBP_MOUSE KIF1-binding protein OS=Mus musculus OX=10090 GN=Kif1bp PE=1...	KIF1-binding protein OS=Mus musculus OX=10090 GN=Kif1bp PE=1 SV=2	
sp|Q8BH59|CMC1_MOUSE Calcium-binding mitochondrial carrier protein Aralar1 OS=Mus...	sp|Q8BH59|CMC1_MOUSE Calcium-binding mitochondrial carrier protein Aralar1 OS=Mus...	
B1AYL0_MOUSE	Sodium channel protein OS=Mus musculus OX=10090 GN=Scn9a PE=1 SV=1	
tr|Q61782|Q61782_MOUSE Type I epidermal keratin mRNA, 3'end (Fragment) OS=Mus musculus...	tr|Q61782|Q61782_MOUSE Type I epidermal keratin mRNA, 3'end (Fragment) OS=Mus musculus...	
sp|Q8CGC6|RBM28_MOUSERNA-binding protein 28 OS=Mus musculus OX=10090 GN=Rbm28 PE=1 SV=4	sp|Q8CGC6|RBM28_MOUSE RNA-binding protein 28 OS=Mus musculus OX=10090 GN=Rbm28 PE=1 SV=4	
Q91YS7_MOUSE	Dual-specificity mitogen-activated protein kinase kinase 2 OS=Mus musculus OX=10090 GN=Map2k2 PE=1 SV=1	
Q0VGN4_MOUSE	Stx16 protein (Fragment) OS=Mus musculus OX=10090 GN=Stx16 PE=2 SV=1	
sp|P62334|PRS10_MOUSE26S proteasome regulatory subunit 10B OS=Mus musculus OX=10090 GN=Psmc6 PE=1 SV=1	sp|P62334|PRS10_MOUSE 26S proteasome regulatory subunit 10B OS=Mus musculus OX=10090 GN=Psmc6 PE=1 SV=1	
tr|A0A1B0GSA8|A0A1B0GSA8_MOUSE 60S ribosomal protein L18 (Fragment) OS=Mus musculus...	tr|A0A1B0GSA8|A0A1B0GSA8_MOUSE 60S ribosomal protein L18 (Fragment) OS=Mus musculus...	
B9EKB5_MOUSE	Baz2b protein OS=Mus musculus OX=10090 GN=Baz2b PE=2 SV=1	
Q8CIH9_MOUSE	Amidophosphoribosyltransferase OS=Mus musculus OX=10090 GN=Ppat PE=1 SV=1	
Q3UJX2_MOUSE	Uncharacterized protein OS=Mus musculus OX=10090 GN=Ctnnbl1 PE=2 SV=1	
tr|E9Q8V6|E9Q8V6_MOUSE-DECOY DENN/MADD domain-containing 4A OS=Mus musculus OX=10090...	tr|E9Q8V6|E9Q8V6_MOUSE-DECOY DENN/MADD domain-containing 4A OS=Mus musculus OX=10090...	
sp|Q91WT9|CBS_MOUSE Cystathionine beta-synthase OS=Mus musculus OX=10090 GN=Cbs PE=1...	sp|Q91WT9|CBS_MOUSE Cystathionine beta-synthase OS=Mus musculus OX=10090 GN=Cbs PE=1...	
sp|Q3ULD5|MCCB_MOUSE Methylcrotonoyl-CoA carboxylase beta chain, mitochondrial OS=Mus...	Methylcrotonoyl-CoA carboxylase beta chain, mitochondrial OS=Mus musculus OX=10090 GN=Mccc2 PE=1 SV=1	
STX4_MOUSE	Syntaxin-4 OS=Mus musculus OX=10090 GN=Stx4 PE=1 SV=1	
tr|A0A1D5RMI8|A0A1D5RMI8_MOUSE Alstrom syndrome protein 1 homolog OS=Mus musculus...	tr|A0A1D5RMI8|A0A1D5RMI8_MOUSE Alstrom syndrome protein 1 homolog OS=Mus musculus...	
E9QNY8_MOUSE	Sacsin OS=Mus musculus OX=10090 GN=Sacs PE=1 SV=1	
F8VPZ3_MOUSE	Ubiquitin-specific peptidase 32 OS=Mus musculus OX=10090 GN=Usp32 PE=1 SV=1	
sp|Q99JI4|PSMD6_MOUSE26S proteasome non-ATPase regulatory subunit 6 OS=Mus musculus OX=10090 GN=Psmd6 PE=1 SV=1	sp|Q99JI4|PSMD6_MOUSE 26S proteasome non-ATPase regulatory subunit 6 OS=Mus musculus OX=10090 GN=Psmd6 PE=1 SV=1	
Q6P9Q2_MOUSE	DNA-directed RNA polymerase subunit beta OS=Mus musculus OX=10090 GN=Polr1b PE=2 SV=1	
E0CZ42_MOUSE	Fer-1-like 6 (C. elegans) OS=Mus musculus OX=10090 GN=Fer1l6 PE=1 SV=1	
E9Q1R5_MOUSE	Voltage-dependent P/Q-type calcium channel subunit alpha OS=Mus musculus OX=10090 GN=Cacna1a PE=1 SV=1	
Q6AXF8_MOUSE	Hmgxb3 protein OS=Mus musculus OX=10090 GN=Hmgxb3 PE=2 SV=1	
sp|Q3V300|KIF22_MOUSEKinesin-like protein KIF22 OS=Mus musculus OX=10090 GN=Kif22 PE=2 SV=2	sp|Q3V300|KIF22_MOUSE Kinesin-like protein KIF22 OS=Mus musculus OX=10090 GN=Kif22 PE=2 SV=2	
Q3TGQ4_MOUSE	Uncharacterized protein OS=Mus musculus OX=10090 GN=Secisbp2 PE=2 SV=1	
MT2_MOUSE	Metallothionein-2 OS=Mus musculus OX=10090 GN=Mt2 PE=1 SV=2	
Q6PDG0_MOUSE	Nup205 protein (Fragment) OS=Mus musculus OX=10090 GN=Nup205 PE=2 SV=1	
Q5M9J5_MOUSE	Ribosomal protein L9 OS=Mus musculus OX=10090 GN=Rpl9 PE=2 SV=1	
H3BKE1_MOUSE	Phosphoinositide phospholipase C (Fragment) OS=Mus musculus OX=10090 GN=Plch1 PE=1 SV=8	
Q91VT3_MOUSE	ATP-binding cassette, sub-family D (ALD), member 3 OS=Mus musculus OX=10090 GN=Abcd3 PE=2 SV=1	
JARD2_MOUSE	Isoform 2 of Protein Jumonji OS=Mus musculus OX=10090 GN=Jarid2	
CFA54_MOUSE	Isoform 2 of Cilia- and flagella-associated protein 54 OS=Mus musculus OX=10090 GN=Cfap54	
E9PV45_MOUSE	Ubiquitin carboxyl-terminal hydrolase 24 OS=Mus musculus OX=10090 GN=Usp24 PE=1 SV=1	
sp|Q9D4H1|EXOC2_MOUSEExocyst complex component 2 OS=Mus musculus OX=10090 GN=Exoc2 PE=1 SV=1	sp|Q9D4H1|EXOC2_MOUSE Exocyst complex component 2 OS=Mus musculus OX=10090 GN=Exoc2 PE=1 SV=1	
sp|O54824|IL16_MOUSE	Pro-interleukin-16 OS=Mus musculus OX=10090 GN=Il16 PE=1 SV=3	
A0A1L1SQA8_MOUSE	40S ribosomal protein S25 OS=Mus musculus OX=10090 GN=Rps25 PE=1 SV=1	
A0A1L1SU22_MOUSE	Cysteine and histidine-rich domain-containing protein 1 (Fragment) OS=Mus musculus OX=10090 GN=Chordc1 PE=1 SV=1	
Q921V3_MOUSE	Nitrogen fixation gene 1 (S. cerevisiae) OS=Mus musculus OX=10090 GN=Nfs1 PE=2 SV=1	
sp|Q61301-3|CTNA2_MOUSE	Isoform 3 of Catenin alpha-2 OS=Mus musculus OX=10090 GN=Ctnna2	
A0A0R4J0G0_MOUSE	Phosphoenolpyruvate carboxykinase [GTP], mitochondrial OS=Mus musculus OX=10090 GN=Pck2 PE=1 SV=1	
Q3T9L0_MOUSE	Uncharacterized protein OS=Mus musculus OX=10090 GN=Ddx39 PE=2 SV=1	
tr|E9Q3N0|E9Q3N0_MOUSE ATP-dependent DNA helicase OS=Mus musculus OX=10090 GN=Recql...	tr|E9Q3N0|E9Q3N0_MOUSE ATP-dependent DNA helicase OS=Mus musculus OX=10090 GN=Recql...	
tr|F6Y3V0|F6Y3V0_MOUSE Receptor-type tyrosine-protein phosphatase F (Fragment) OS=Mus...	tr|F6Y3V0|F6Y3V0_MOUSE Receptor-type tyrosine-protein phosphatase F (Fragment) OS=Mus...	
tr|F8VQE9|F8VQE9_MOUSE Arf-GAP with GTPase, ANK repeat and PH domain-containing protein...	Arf-GAP with GTPase, ANK repeat and PH domain-containing protein 3 OS=Mus musculus OX=10090 GN=Agap3 PE=1 SV=1	
tr|Q3T9Y9|Q3T9Y9_MOUSE Uncharacterized protein (Fragment) OS=Mus musculus OX=10090...	tr|Q3T9Y9|Q3T9Y9_MOUSE Uncharacterized protein (Fragment) OS=Mus musculus OX=10090...	
AGO2_MOUSE	Protein argonaute-2 OS=Mus musculus OX=10090 GN=Ago2 PE=1 SV=3	
tr|B1AR51|B1AR51_MOUSE Dynein, axonemal, heavy chain 9 OS=Mus musculus OX=10090 GN=Dnah9...	tr|B1AR51|B1AR51_MOUSE Dynein, axonemal, heavy chain 9 OS=Mus musculus OX=10090 GN=Dnah9...	
tr|Q3TU36|Q3TU36_MOUSE RAP1, GTP-GDP dissociation stimulator 1 OS=Mus musculus OX=10090...	RAP1, GTP-GDP dissociation stimulator 1 OS=Mus musculus OX=10090 GN=Rap1gds1 PE=1 SV=1	
sp|E9Q6J5-3|BD1L1_MOUSE Isoform New of Biorientation of chromosomes in cell division...	Isoform New of Biorientation of chromosomes in cell division protein 1-like 1 OS=Mus musculus OX=10090 GN=Bod1l	
D3YYT1_MOUSE	Putative oxidoreductase GLYR1 OS=Mus musculus OX=10090 GN=Glyr1 PE=1 SV=1	
tr|A0A1B0GRY3|A0A1B0GRY3_MOUSE Predicted gene 45861 OS=Mus musculus OX=10090 GN=Gm45861...	tr|A0A1B0GRY3|A0A1B0GRY3_MOUSE Predicted gene 45861 OS=Mus musculus OX=10090 GN=Gm45861...	
tr|A0A140LIJ4|A0A140LIJ4_MOUSE-DECOY Dynein, axonemal, heavy chain 14 OS=Mus musculus...	tr|A0A140LIJ4|A0A140LIJ4_MOUSE-DECOY Dynein, axonemal, heavy chain 14 OS=Mus musculus...	
Q571M5_MOUSE	MKIAA4011 protein (Fragment) OS=Mus musculus OX=10090 GN=Itch PE=2 SV=1	
J3QN19_MOUSE	DNA primase OS=Mus musculus OX=10090 GN=Prim1 PE=1 SV=1	
sp|Q9CY64|BIEA_MOUSE Biliverdin reductase A OS=Mus musculus OX=10090 GN=Blvra PE=1...	sp|Q9CY64|BIEA_MOUSE Biliverdin reductase A OS=Mus musculus OX=10090 GN=Blvra PE=1...	
sp|Q62388|ATM_MOUSE Serine-protein kinase ATM OS=Mus musculus OX=10090 GN=Atm PE=1...	sp|Q62388|ATM_MOUSE Serine-protein kinase ATM OS=Mus musculus OX=10090 GN=Atm PE=1...	
sp|Q922H2|PDK3_MOUSE [Pyruvate dehydrogenase (acetyl-transferring)] kinase isozyme...	sp|Q922H2|PDK3_MOUSE [Pyruvate dehydrogenase (acetyl-transferring)] kinase isozyme...	
Q3UGS1_MOUSE	Uncharacterized protein OS=Mus musculus OX=10090 GN=Nipsnap1 PE=2 SV=1	
tr|V9GX23|V9GX23_MOUSE Vacuolar protein sorting 13D (Fragment) OS=Mus musculus OX=10090...	Vacuolar protein sorting 13D (Fragment) OS=Mus musculus OX=10090 GN=Vps13d PE=1 SV=1	
sp|Q9QZQ1-2|AFAD_MOUSE-DECOY Isoform 1 of Afadin OS=Mus musculus OX=10090 GN=Afdn	sp|Q9QZQ1-2|AFAD_MOUSE-DECOY Isoform 1 of Afadin OS=Mus musculus OX=10090 GN=Afdn	
sp|P01027|CO3_MOUSE-DECOY Complement C3 OS=Mus musculus OX=10090 GN=C3 PE=1 SV=3	sp|P01027|CO3_MOUSE-DECOY Complement C3 OS=Mus musculus OX=10090 GN=C3 PE=1 SV=3	
sp|Q9WTI7-2|MYO1C_MOUSE	Isoform 2 of Unconventional myosin-Ic OS=Mus musculus OX=10090 GN=Myo1c	
sp|Q8C1B1-2|CAMP2_MOUSE Isoform 2 of Calmodulin-regulated spectrin-associated protein...	sp|Q8C1B1-2|CAMP2_MOUSE Isoform 2 of Calmodulin-regulated spectrin-associated protein...	
sp|Q3TEA8-2|HP1B3_MOUSE	Isoform 2 of Heterochromatin protein 1-binding protein 3 OS=Mus musculus OX=10090 GN=Hp1bp3	
tr|B9EKC3|B9EKC3_MOUSE-DECOY Rho GTPase activating protein 5 OS=Mus musculus OX=10090...	tr|B9EKC3|B9EKC3_MOUSE-DECOY Rho GTPase activating protein 5 OS=Mus musculus OX=10090...	
tr|A0A0G2JG52|A0A0G2JG52_MOUSE Crystallin beta-gamma domain-containing 1 OS=Mus musculus...	tr|A0A0G2JG52|A0A0G2JG52_MOUSE Crystallin beta-gamma domain-containing 1 OS=Mus musculus...	
sp|Q11011|PSA_MOUSE Puromycin-sensitive aminopeptidase OS=Mus musculus OX=10090 GN=Npepps...	Puromycin-sensitive aminopeptidase OS=Mus musculus OX=10090 GN=Npepps PE=1 SV=2	
sp|Q6NXI6-2|RPRD2_MOUSE Isoform 2 of Regulation of nuclear pre-mRNA domain-containing...	sp|Q6NXI6-2|RPRD2_MOUSE Isoform 2 of Regulation of nuclear pre-mRNA domain-containing...	
tr|Q8K205|Q8K205_MOUSE Processing of 1, ribonuclease P/MRP family, (S. cerevisiae)...	Processing of 1, ribonuclease P/MRP family, (S. cerevisiae) OS=Mus musculus OX=10090 GN=Pop1 PE=1 SV=1	
sp|A2CG49-7|KALRN_MOUSE-DECOY Isoform 7 of Kalirin OS=Mus musculus OX=10090 GN=Kalrn	sp|A2CG49-7|KALRN_MOUSE-DECOY Isoform 7 of Kalirin OS=Mus musculus OX=10090 GN=Kalrn	
sp|Q9JLC8-3|SACS_MOUSE-DECOY Isoform 3 of Sacsin OS=Mus musculus OX=10090 GN=Sacs	sp|Q9JLC8-3|SACS_MOUSE-DECOY Isoform 3 of Sacsin OS=Mus musculus OX=10090 GN=Sacs	
tr|A0A1D5RLL4|A0A1D5RLL4_MOUSE-DECOY Transformation/transcription domain-associated...	tr|A0A1D5RLL4|A0A1D5RLL4_MOUSE-DECOY Transformation/transcription domain-associated...	
NLE1_MOUSE	Notchless protein homolog 1 OS=Mus musculus OX=10090 GN=Nle1 PE=1 SV=4	
tr|Q3TKP3|Q3TKP3_MOUSE Uncharacterized protein OS=Mus musculus OX=10090 GN=Atxn10...	tr|Q3TKP3|Q3TKP3_MOUSE Uncharacterized protein OS=Mus musculus OX=10090 GN=Atxn10...	
D3Z487_MOUSE	Translation initiation factor eIF-2B subunit beta (Fragment) OS=Mus musculus OX=10090 GN=Eif2b2 PE=1 SV=1	
tr|A0A1S6GWH6|A0A1S6GWH6_MOUSE-DECOY Uncharacterized protein OS=Mus musculus OX=10090...	tr|A0A1S6GWH6|A0A1S6GWH6_MOUSE-DECOY Uncharacterized protein OS=Mus musculus OX=10090...	
A2AK42_MOUSE	Predicted gene 13691 OS=Mus musculus OX=10090 GN=Gm13697 PE=4 SV=1	
sp|Q3UHX0|NOL8_MOUSE-DECOY Nucleolar protein 8 OS=Mus musculus OX=10090 GN=Nol8 PE=1...	sp|Q3UHX0|NOL8_MOUSE-DECOY Nucleolar protein 8 OS=Mus musculus OX=10090 GN=Nol8 PE=1...	
sp|Q4VA53-3|PDS5B_MOUSE-DECOY Isoform 3 of Sister chromatid cohesion protein PDS5...	sp|Q4VA53-3|PDS5B_MOUSE-DECOY Isoform 3 of Sister chromatid cohesion protein PDS5...	
tr|A0A140LJJ5|A0A140LJJ5_MOUSE-DECOY A-kinase anchor protein 13 OS=Mus musculus OX=10090...	tr|A0A140LJJ5|A0A140LJJ5_MOUSE-DECOY A-kinase anchor protein 13 OS=Mus musculus OX=10090...	
sp|P70298|CUX2_MOUSE-DECOY Homeobox protein cut-like 2 OS=Mus musculus OX=10090 GN=Cux2...	sp|P70298|CUX2_MOUSE-DECOY Homeobox protein cut-like 2 OS=Mus musculus OX=10090 GN=Cux2...	
tr|A7TU71|A7TU71_MOUSE-DECOY Protein Shroom2 OS=Mus musculus OX=10090 GN=Shroom2...	tr|A7TU71|A7TU71_MOUSE-DECOY Protein Shroom2 OS=Mus musculus OX=10090 GN=Shroom2...	
tr|D3Z5K8|D3Z5K8_MOUSE SH3 and multiple ankyrin repeat domains protein 2 OS=Mus musculus...	SH3 and multiple ankyrin repeat domains protein 2 OS=Mus musculus OX=10090 GN=Shank2 PE=1 SV=1	
tr|E9PWT1|E9PWT1_MOUSE Transformation/transcription domain-associated protein OS=Mus...	Transformation/transcription domain-associated protein OS=Mus musculus OX=10090 GN=Trrap PE=1 SV=1	
tr|H7BX26|H7BX26_MOUSE Centrosomal protein of 170 kDa OS=Mus musculus OX=10090 GN=Cep170...	tr|H7BX26|H7BX26_MOUSE Centrosomal protein of 170 kDa OS=Mus musculus OX=10090 GN=Cep170...	
tr|Q3UYH9|Q3UYH9_MOUSE Alpha-1,4 glucan phosphorylase OS=Mus musculus OX=10090 GN=Pygb...	Alpha-1,4 glucan phosphorylase OS=Mus musculus OX=10090 GN=Pygb PE=2 SV=1	
sp|Q8QZS1|HIBCH_MOUSE3-hydroxyisobutyryl-CoA hydrolase, mitochondrial OS=Mus musculus OX=10090 GN=Hibch PE=1 SV=1	sp|Q8QZS1|HIBCH_MOUSE 3-hydroxyisobutyryl-CoA hydrolase, mitochondrial OS=Mus musculus OX=10090 GN=Hibch PE=1 SV=1	
B1AUN3_MOUSE	Eukaryotic translation initiation factor 2B, subunit 3 OS=Mus musculus OX=10090 GN=Eif2b3 PE=1 SV=1	
RFC4_MOUSE	Replication factor C subunit 4 OS=Mus musculus OX=10090 GN=Rfc4 PE=1 SV=1	
tr|F8WIE5|F8WIE5_MOUSE E3 ubiquitin-protein ligase HECTD1 OS=Mus musculus OX=10090...	tr|F8WIE5|F8WIE5_MOUSE E3 ubiquitin-protein ligase HECTD1 OS=Mus musculus OX=10090...	
tr|A0A0R4J0D3|A0A0R4J0D3_MOUSE Dolichyl-diphosphooligosaccharide--protein glycosyltransferase...	tr|A0A0R4J0D3|A0A0R4J0D3_MOUSE Dolichyl-diphosphooligosaccharide--protein glycosyltransferase...	
IF2B_MOUSE	Eukaryotic translation initiation factor 2 subunit 2 OS=Mus musculus OX=10090 GN=Eif2s2 PE=1 SV=1	
WRIP1_MOUSE	Isoform 2 of ATPase WRNIP1 OS=Mus musculus OX=10090 GN=Wrnip1	
K7Q751_MOUSE	Focal ashension kinase 1 OS=Mus musculus OX=10090 GN=Ptk2 PE=2 SV=1	
G3UZA0_MOUSE	KRR1 small subunit processome component OS=Mus musculus OX=10090 GN=Krr1 PE=1 SV=1	
A2AS98_MOUSE	Nck-associated protein 1 OS=Mus musculus OX=10090 GN=Nckap1 PE=1 SV=1	
SUCA_MOUSE	Succinate--CoA ligase [ADP/GDP-forming] subunit alpha, mitochondrial OS=Mus musculus OX=10090 GN=Suclg1 PE=1 SV=4	
tr|Q3U0F8|Q3U0F8_MOUSE Uncharacterized protein (Fragment) OS=Mus musculus OX=10090...	tr|Q3U0F8|Q3U0F8_MOUSE Uncharacterized protein (Fragment) OS=Mus musculus OX=10090...	
A0A0G2JFB4_MOUSE	Eukaryotic translation initiation factor 4E OS=Mus musculus OX=10090 GN=Eif4e PE=1 SV=1	
UCHL5_MOUSE	Isoform 2 of Ubiquitin carboxyl-terminal hydrolase isozyme L5 OS=Mus musculus OX=10090 GN=Uchl5	
G3X9F7_MOUSE	DEAD (Asp-Glu-Ala-Asp) box polypeptide 60 OS=Mus musculus OX=10090 GN=Ddx60 PE=1 SV=1	
B2RY79_MOUSE	Dock9 protein OS=Mus musculus OX=10090 GN=Dock9 PE=2 SV=1	
Q68FC6_MOUSE	Wrd43 protein (Fragment) OS=Mus musculus OX=10090 GN=Wdr43 PE=2 SV=1	
tr|Q922G9|Q922G9_MOUSE Copg2 protein (Fragment) OS=Mus musculus OX=10090 GN=Copg2...	tr|Q922G9|Q922G9_MOUSE Copg2 protein (Fragment) OS=Mus musculus OX=10090 GN=Copg2...	
A0A1W2P7C7_MOUSE	Plasma membrane calcium-transporting ATPase 1 (Fragment) OS=Mus musculus OX=10090 GN=Atp2b1 PE=1 SV=1	
sp|P26516|PSMD7_MOUSE26S proteasome non-ATPase regulatory subunit 7 OS=Mus musculus OX=10090 GN=Psmd7 PE=1 SV=2	sp|P26516|PSMD7_MOUSE 26S proteasome non-ATPase regulatory subunit 7 OS=Mus musculus OX=10090 GN=Psmd7 PE=1 SV=2	
Q3UXM3_MOUSE	Uncharacterized protein OS=Mus musculus OX=10090 GN=Maged1 PE=2 SV=1	
Q6NZD2_MOUSE	Sorting nexin-1 OS=Mus musculus OX=10090 GN=Snx1 PE=1 SV=1	
Q31143_MOUSE	Histocompatibility 2, O region beta locus OS=Mus musculus OX=10090 GN=H2-Ob PE=3 SV=1	
sp|P04117|FABP4_MOUSEFatty acid-binding protein, adipocyte OS=Mus musculus OX=10090 GN=Fabp4 PE=1 SV=3	sp|P04117|FABP4_MOUSE Fatty acid-binding protein, adipocyte OS=Mus musculus OX=10090 GN=Fabp4 PE=1 SV=3	
tr|A0A2I3BQ03|A0A2I3BQ03_MOUSE 14-3-3 protein zeta/delta (Fragment) OS=Mus musculus...	tr|A0A2I3BQ03|A0A2I3BQ03_MOUSE 14-3-3 protein zeta/delta (Fragment) OS=Mus musculus...	
Q3TT06_MOUSE	Uncharacterized protein OS=Mus musculus OX=10090 GN=Nup107 PE=2 SV=1	
FRY_MOUSE	Protein furry homolog OS=Mus musculus OX=10090 GN=Fry PE=1 SV=1	
ARMC3_MOUSE	Isoform 2 of Armadillo repeat-containing protein 3 OS=Mus musculus OX=10090 GN=Armc3	
Q149V4_MOUSE	Histone H2A OS=Mus musculus OX=10090 GN=Hist2h2ac PE=2 SV=1	
A0A0U1RNK9_MOUSE	Electron transfer flavoprotein subunit beta (Fragment) OS=Mus musculus OX=10090 GN=Etfb PE=1 SV=2	
A0A1S6GWJ0_MOUSE	Oxysterol-binding protein OS=Mus musculus OX=10090 GN=Osbpl9 PE=2 SV=1	
A2ALR8_MOUSE	Ubiquitin carboxyl-terminal hydrolase 48 (Fragment) OS=Mus musculus OX=10090 GN=Usp48 PE=1 SV=1	
NU160_MOUSE	Isoform 2 of Nuclear pore complex protein Nup160 OS=Mus musculus OX=10090 GN=Nup160	
Q7TNL5_MOUSE	Serine/threonine-protein phosphatase 2A 56 kDa regulatory subunit OS=Mus musculus OX=10090 GN=Ppp2r5d PE=2 SV=1	
TSN_MOUSE	Translin OS=Mus musculus OX=10090 GN=Tsn PE=1 SV=1	
sp|E9PVA8|GCN1_MOUSE-DECOY eIF-2-alpha kinase activator GCN1 OS=Mus musculus OX=10090...	sp|E9PVA8|GCN1_MOUSE-DECOY eIF-2-alpha kinase activator GCN1 OS=Mus musculus OX=10090...	
sp|P14152|MDHC_MOUSE Malate dehydrogenase, cytoplasmic OS=Mus musculus OX=10090 GN=Mdh1...	sp|P14152|MDHC_MOUSE Malate dehydrogenase, cytoplasmic OS=Mus musculus OX=10090 GN=Mdh1...	
sp|Q8C196|CPSM_MOUSE Carbamoyl-phosphate synthase [ammonia], mitochondrial OS=Mus...	sp|Q8C196|CPSM_MOUSE Carbamoyl-phosphate synthase [ammonia], mitochondrial OS=Mus...	
sp|Q8K0C9|GMDS_MOUSE GDP-mannose 4,6 dehydratase OS=Mus musculus OX=10090 GN=Gmds...	GDP-mannose 4,6 dehydratase OS=Mus musculus OX=10090 GN=Gmds PE=1 SV=1	
sp|Q9JJV2-2|PROF2_MOUSE	Isoform 2 of Profilin-2 OS=Mus musculus OX=10090 GN=Pfn2	
tr|A0A0A0MQM0|A0A0A0MQM0_MOUSE Eukaryotic translation initiation factor 5A (Fragment)...	tr|A0A0A0MQM0|A0A0A0MQM0_MOUSE Eukaryotic translation initiation factor 5A (Fragment)...	
tr|A0A0R4J1L9|A0A0R4J1L9_MOUSE Microtubule-associated tumor suppressor 1 homolog...	tr|A0A0R4J1L9|A0A0R4J1L9_MOUSE Microtubule-associated tumor suppressor 1 homolog...	
tr|A2ARP8|A2ARP8_MOUSE Microtubule-associated protein 1A OS=Mus musculus OX=10090...	Microtubule-associated protein 1A OS=Mus musculus OX=10090 GN=Map1a PE=1 SV=1	
tr|B2RQQ5|B2RQQ5_MOUSE Microtubule-associated protein 1B OS=Mus musculus OX=10090...	tr|B2RQQ5|B2RQQ5_MOUSE Microtubule-associated protein 1B OS=Mus musculus OX=10090...	
tr|D6RHT6|D6RHT6_MOUSE E3 ubiquitin-protein ligase TTC3 (Fragment) OS=Mus musculus...	E3 ubiquitin-protein ligase TTC3 (Fragment) OS=Mus musculus OX=10090 GN=Ttc3 PE=1 SV=1	
tr|E9Q0N0|E9Q0N0_MOUSE-DECOY Intersectin-1 OS=Mus musculus OX=10090 GN=Itsn1 PE=1...	tr|E9Q0N0|E9Q0N0_MOUSE-DECOY Intersectin-1 OS=Mus musculus OX=10090 GN=Itsn1 PE=1...	
sp|Q5SVQ0-4|KAT7_MOUSE	Isoform 4 of Histone acetyltransferase KAT7 OS=Mus musculus OX=10090 GN=Kat7	
sp|Q9D2G2|ODO2_MOUSE Dihydrolipoyllysine-residue succinyltransferase component of...	sp|Q9D2G2|ODO2_MOUSE Dihydrolipoyllysine-residue succinyltransferase component of...	
sp|Q9ERD7|TBB3_MOUSE Tubulin beta-3 chain OS=Mus musculus OX=10090 GN=Tubb3 PE=1...	Tubulin beta-3 chain OS=Mus musculus OX=10090 GN=Tubb3 PE=1 SV=1	
sp|Q9CZX0-2|ELP3_MOUSE Isoform 2 of Elongator complex protein 3 OS=Mus musculus OX=10090...	Isoform 2 of Elongator complex protein 3 OS=Mus musculus OX=10090 GN=Elp3	
I3PQW3_MOUSE	LARP7 OS=Mus musculus OX=10090 GN=Larp7 PE=4 SV=1	
W6PPR4_MOUSE	480-kDa ankyrinG OS=Mus musculus OX=10090 GN=Ank3 PE=4 SV=1	
sp|O88492-2|PLIN4_MOUSE-DECOY Isoform 2 of Perilipin-4 OS=Mus musculus OX=10090 GN=Plin4	sp|O88492-2|PLIN4_MOUSE-DECOY Isoform 2 of Perilipin-4 OS=Mus musculus OX=10090 GN=Plin4	
sp|Q8BKX6|SMG1_MOUSE Serine/threonine-protein kinase SMG1 OS=Mus musculus OX=10090...	Serine/threonine-protein kinase SMG1 OS=Mus musculus OX=10090 GN=Smg1 PE=1 SV=3	
sp|Q99LH1|NOG2_MOUSE Nucleolar GTP-binding protein 2 OS=Mus musculus OX=10090 GN=Gnl2...	sp|Q99LH1|NOG2_MOUSE Nucleolar GTP-binding protein 2 OS=Mus musculus OX=10090 GN=Gnl2...	
tr|A0A0A0MQ73|A0A0A0MQ73_MOUSE-DECOY Histone-lysine N-methyltransferase 2D OS=Mus...	tr|A0A0A0MQ73|A0A0A0MQ73_MOUSE-DECOY Histone-lysine N-methyltransferase 2D OS=Mus...	
tr|H3BLK8|H3BLK8_MOUSE-DECOY Zinc finger homeobox protein 4 OS=Mus musculus OX=10090...	tr|H3BLK8|H3BLK8_MOUSE-DECOY Zinc finger homeobox protein 4 OS=Mus musculus OX=10090...	
tr|Q8BVX3|Q8BVX3_MOUSE Uncharacterized protein (Fragment) OS=Mus musculus OX=10090...	tr|Q8BVX3|Q8BVX3_MOUSE Uncharacterized protein (Fragment) OS=Mus musculus OX=10090...	
sp|Q80Y81|RNZ2_MOUSE	Zinc phosphodiesterase ELAC protein 2 OS=Mus musculus OX=10090 GN=Elac2 PE=1 SV=1	
A0A068BFS4_MOUSE	Ring finger protein 1 OS=Mus musculus OX=10090 GN=Ring1 PE=2 SV=1	
O70349_MOUSE	MCG15924, isoform CRA_a OS=Mus musculus OX=10090 GN=SKI PE=4 SV=1	
Q8BSH9_MOUSE	Nucleosome assembly protein 1-like 1 OS=Mus musculus OX=10090 GN=Nap1l1 PE=1 SV=1	
Q3TST1_MOUSE	Gremlin OS=Mus musculus OX=10090 GN=Grem2 PE=2 SV=1	
NIPBL_MOUSE	Isoform 2 of Nipped-B-like protein OS=Mus musculus OX=10090 GN=Nipbl	
tr|G3UW82|G3UW82_MOUSE MCG140437, isoform CRA_d OS=Mus musculus OX=10090 GN=Myh2...	MCG140437, isoform CRA_d OS=Mus musculus OX=10090 GN=Myh2 PE=1 SV=1	
sp|Q45VK7-2|DYHC2_MOUSE-DECOY Isoform 2 of Cytoplasmic dynein 2 heavy chain 1 OS=Mus...	sp|Q45VK7-2|DYHC2_MOUSE-DECOY Isoform 2 of Cytoplasmic dynein 2 heavy chain 1 OS=Mus...	
sp|Q8VBZ3|CLPT1_MOUSECleft lip and palate transmembrane protein 1 homolog OS=Mus musculus OX=10090 GN=Clptm1 PE=1 SV=1	sp|Q8VBZ3|CLPT1_MOUSE Cleft lip and palate transmembrane protein 1 homolog OS=Mus musculus OX=10090 GN=Clptm1 PE=1 SV=1	
IMA5_MOUSE	Importin subunit alpha-5 OS=Mus musculus OX=10090 GN=Kpna1 PE=1 SV=2	
sp|Q9QY06-2|MYO9B_MOUSE-DECOY Isoform 2 of Unconventional myosin-IXb OS=Mus musculus...	sp|Q9QY06-2|MYO9B_MOUSE-DECOY Isoform 2 of Unconventional myosin-IXb OS=Mus musculus...	
Q3THX4_MOUSE	Uncharacterized protein OS=Mus musculus OX=10090 GN=Vps45 PE=2 SV=1	
E9Q8K8_MOUSE	Zinc finger CCCH domain-containing protein 4 OS=Mus musculus OX=10090 GN=Zc3h4 PE=1 SV=1	
SORL_MOUSE	Sortilin-related receptor OS=Mus musculus OX=10090 GN=Sorl1 PE=1 SV=3	
sp|Q9Z0P5-2|TWF2_MOUSE	Isoform 2 of Twinfilin-2 OS=Mus musculus OX=10090 GN=Twf2	
sp|Q62315-2|JARD2_MOUSE-DECOY Isoform 2 of Protein Jumonji OS=Mus musculus OX=10090...	sp|Q62315-2|JARD2_MOUSE-DECOY Isoform 2 of Protein Jumonji OS=Mus musculus OX=10090...	
Q66X24_MOUSE	NALP-beta (Fragment) OS=Mus musculus OX=10090 GN=Nlrp4d PE=2 SV=1	
Q3TCH2_MOUSE	Ubiquitin carboxyl-terminal hydrolase (Fragment) OS=Mus musculus OX=10090 GN=Uchl1 PE=2 SV=1	
tr|A0A1W2P7W3|A0A1W2P7W3_MOUSE-DECOY Protein tyrosine phosphatase, receptor type,...	tr|A0A1W2P7W3|A0A1W2P7W3_MOUSE-DECOY Protein tyrosine phosphatase, receptor type,...	
sp|Q64028-2|PHC1_MOUSE-DECOY Isoform 2 of Polyhomeotic-like protein 1 OS=Mus musculus...	sp|Q64028-2|PHC1_MOUSE-DECOY Isoform 2 of Polyhomeotic-like protein 1 OS=Mus musculus...	
A0A1W2P7A1_MOUSE	40S ribosomal protein S12 OS=Mus musculus OX=10090 GN=Rps12 PE=1 SV=1	
A0A1B0GT06_MOUSE	Predicted gene, 29735 OS=Mus musculus OX=10090 GN=Gm29735 PE=4 SV=1	
sp|Q6PE01|SNR40_MOUSEU5 small nuclear ribonucleoprotein 40 kDa protein OS=Mus musculus OX=10090 GN=Snrnp40 PE=1 SV=1	sp|Q6PE01|SNR40_MOUSE U5 small nuclear ribonucleoprotein 40 kDa protein OS=Mus musculus OX=10090 GN=Snrnp40 PE=1 SV=1	
SRSF3_MOUSE	Isoform Short of Serine/arginine-rich splicing factor 3 OS=Mus musculus OX=10090 GN=Srsf3	
PGM2_MOUSE	Phosphoglucomutase-2 OS=Mus musculus OX=10090 GN=Pgm2 PE=1 SV=1	
sp|Q8BKX6|SMG1_MOUSE-DECOY Serine/threonine-protein kinase SMG1 OS=Mus musculus OX=10090...	sp|Q8BKX6|SMG1_MOUSE-DECOY Serine/threonine-protein kinase SMG1 OS=Mus musculus OX=10090...	
ARAF_MOUSE	Serine/threonine-protein kinase A-Raf OS=Mus musculus OX=10090 GN=Araf PE=1 SV=2	
Q3UG68_MOUSE	Dolichyl-diphosphooligosaccharide--protein glycosyltransferase 48 kDa subunit OS=Mus musculus OX=10090 GN=Ddost PE=2 SV=1	
Q3TX38_MOUSE	Uncharacterized protein OS=Mus musculus OX=10090 GN=Vdac3 PE=1 SV=1	
tr|Q3TIV2|Q3TIV2_MOUSE Uncharacterized protein OS=Mus musculus OX=10090 GN=Zc3h11a...	Uncharacterized protein OS=Mus musculus OX=10090 GN=Zc3h11a PE=2 SV=1	
Q3UZR8_MOUSE	Eukaryotic translation initiation factor 2B, subunit 1 (Alpha) OS=Mus musculus OX=10090 GN=Eif2b1 PE=1 SV=1	
B2RY11_MOUSE	Abca12 protein OS=Mus musculus OX=10090 GN=Abca12 PE=2 SV=1	
G3X9F2_MOUSE	Histone demethylase UTY OS=Mus musculus OX=10090 GN=Uty PE=1 SV=1	
A0A1B0GSA0_MOUSE	Predicted gene 45618 OS=Mus musculus OX=10090 GN=Gm45618 PE=4 SV=1	
A0A0R4J150_MOUSE	Oxysterol-binding protein OS=Mus musculus OX=10090 GN=Osbpl8 PE=1 SV=1	
PLSI_MOUSE	Plastin-1 OS=Mus musculus OX=10090 GN=Pls1 PE=1 SV=1	
Q6A0C9_MOUSE	MKIAA0109 protein (Fragment) OS=Mus musculus OX=10090 GN=Ap2m1 PE=2 SV=1	
sp|A2AKX3-2|SETX_MOUSE	Isoform 2 of Probable helicase senataxin OS=Mus musculus OX=10090 GN=Setx	
sp|Q641K1-2|CBPC1_MOUSE	Isoform 2 of Cytosolic carboxypeptidase 1 OS=Mus musculus OX=10090 GN=Agtpbp1	
MUG1_MOUSE	Murinoglobulin-1 OS=Mus musculus OX=10090 GN=Mug1 PE=1 SV=3	
E9Q3I4_MOUSE	Intersectin-1 OS=Mus musculus OX=10090 GN=Itsn1 PE=1 SV=1	
A0A0R4J198_MOUSE	Succinyl-CoA:3-ketoacid-coenzyme A transferase OS=Mus musculus OX=10090 GN=Oxct2b PE=3 SV=1	
tr|Q3TI71|Q3TI71_MOUSE Uncharacterized protein OS=Mus musculus OX=10090 GN=Prpf8...	tr|Q3TI71|Q3TI71_MOUSE Uncharacterized protein OS=Mus musculus OX=10090 GN=Prpf8...	
sp|Q3B7Z2|OSBP1_MOUSEOxysterol-binding protein 1 OS=Mus musculus OX=10090 GN=Osbp PE=1 SV=3	sp|Q3B7Z2|OSBP1_MOUSE Oxysterol-binding protein 1 OS=Mus musculus OX=10090 GN=Osbp PE=1 SV=3	
sp|Q3UDE2|TTL12_MOUSETubulin--tyrosine ligase-like protein 12 OS=Mus musculus OX=10090 GN=Ttll12 PE=1 SV=1	sp|Q3UDE2|TTL12_MOUSE Tubulin--tyrosine ligase-like protein 12 OS=Mus musculus OX=10090 GN=Ttll12 PE=1 SV=1	
Q8C7E6_MOUSE	Uncharacterized protein OS=Mus musculus OX=10090 PE=2 SV=1	
B1AR28_MOUSE	Very long-chain-specific acyl-CoA dehydrogenase, mitochondrial OS=Mus musculus OX=10090 GN=Acadvl PE=1 SV=1	
CO3_MOUSE	Complement C3 OS=Mus musculus OX=10090 GN=C3 PE=1 SV=3	
Q3TNP4_MOUSE	Uncharacterized protein OS=Mus musculus OX=10090 GN=Fam208b PE=2 SV=1	
GRM3_MOUSE	Metabotropic glutamate receptor 3 OS=Mus musculus OX=10090 GN=Grm3 PE=1 SV=1	
D3Z5G8_MOUSE	Disco-interacting protein 2 homolog B OS=Mus musculus OX=10090 GN=Dip2b PE=1 SV=1	
E9Q7H6_MOUSE	Threonine--tRNA ligase, mitochondrial OS=Mus musculus OX=10090 GN=Tars2 PE=1 SV=1	
R4H4Y7_MOUSE	SCY1-like 1 splice variant 13 OS=Mus musculus OX=10090 GN=Scyl1 PE=2 SV=1	
B7ZMT5_MOUSE	Mmrn1 protein OS=Mus musculus OX=10090 GN=Mmrn1 PE=2 SV=1	
sp|Q8K411-2|PREP_MOUSE	Isoform 2 of Presequence protease, mitochondrial OS=Mus musculus OX=10090 GN=Pitrm1	
EDC3_MOUSE	Enhancer of mRNA-decapping protein 3 OS=Mus musculus OX=10090 GN=Edc3 PE=1 SV=1	
sp|Q9Z2I0|LETM1_MOUSEMitochondrial proton/calcium exchanger protein OS=Mus musculus OX=10090 GN=Letm1 PE=1 SV=1	sp|Q9Z2I0|LETM1_MOUSE Mitochondrial proton/calcium exchanger protein OS=Mus musculus OX=10090 GN=Letm1 PE=1 SV=1	
PSA4_MOUSE	Proteasome subunit alpha type-4 OS=Mus musculus OX=10090 GN=Psma4 PE=1 SV=1	
Q52L97_MOUSE	Importin subunit alpha OS=Mus musculus OX=10090 GN=Kpna2 PE=1 SV=1	
G3X9H5_MOUSE	Huntingtin OS=Mus musculus OX=10090 GN=Htt PE=1 SV=1	
Q3UD86_MOUSE	Uncharacterized protein OS=Mus musculus OX=10090 GN=Mov10 PE=2 SV=1	
HELLS_MOUSE	Isoform 2 of Lymphocyte-specific helicase OS=Mus musculus OX=10090 GN=Hells	
A0A0J9YU62_MOUSE	C-terminal-binding protein 1 OS=Mus musculus OX=10090 GN=Ctbp1 PE=1 SV=1	
Q3TF25_MOUSE	Uncharacterized protein OS=Mus musculus OX=10090 GN=Atp5o PE=2 SV=1	
sp|Q9JK48-2|SHLB1_MOUSE	Isoform 2 of Endophilin-B1 OS=Mus musculus OX=10090 GN=Sh3glb1	
tr|A2AAN2|A2AAN2_MOUSE-DECOY Signal recognition particle subunit SRP68 OS=Mus musculus...	tr|A2AAN2|A2AAN2_MOUSE-DECOY Signal recognition particle subunit SRP68 OS=Mus musculus...	
tr|A8IP69|A8IP69_MOUSE 14-3-3 protein gamma subtype OS=Mus musculus OX=10090 GN=Ywhag...	tr|A8IP69|A8IP69_MOUSE 14-3-3 protein gamma subtype OS=Mus musculus OX=10090 GN=Ywhag...	
tr|B2RWU2|B2RWU2_MOUSE-DECOY Immunodeficiency virus type I enhancer binding protein...	tr|B2RWU2|B2RWU2_MOUSE-DECOY Immunodeficiency virus type I enhancer binding protein...	
tr|Q3TXN6|Q3TXN6_MOUSE Uncharacterized protein (Fragment) OS=Mus musculus OX=10090...	Uncharacterized protein (Fragment) OS=Mus musculus OX=10090 GN=Cstf3 PE=2 SV=1	
tr|Q3U9P7|Q3U9P7_MOUSE Succinyl-CoA:3-ketoacid-coenzyme A transferase OS=Mus musculus...	Succinyl-CoA:3-ketoacid-coenzyme A transferase OS=Mus musculus OX=10090 GN=Oxct1 PE=2 SV=1	
tr|A0A1B0GS20|A0A1B0GS20_MOUSE-DECOY Predicted gene 45861 OS=Mus musculus OX=10090...	tr|A0A1B0GS20|A0A1B0GS20_MOUSE-DECOY Predicted gene 45861 OS=Mus musculus OX=10090...	
tr|Q3TVV7|Q3TVV7_MOUSE Uncharacterized protein OS=Mus musculus OX=10090 GN=Umps PE=2...	Uncharacterized protein OS=Mus musculus OX=10090 GN=Umps PE=2 SV=1	
sp|P35922-2|FMR1_MOUSE-DECOY Isoform 2 of Synaptic functional regulator FMR1 OS=Mus...	sp|P35922-2|FMR1_MOUSE-DECOY Isoform 2 of Synaptic functional regulator FMR1 OS=Mus...	
tr|A0A087WQ94|A0A087WQ94_MOUSE-DECOY Tensin 1 (Fragment) OS=Mus musculus OX=10090...	tr|A0A087WQ94|A0A087WQ94_MOUSE-DECOY Tensin 1 (Fragment) OS=Mus musculus OX=10090...	
tr|E9Q1M1|E9Q1M1_MOUSE-DECOY PDZ domain-containing 2 OS=Mus musculus OX=10090 GN=Pdzd2...	tr|E9Q1M1|E9Q1M1_MOUSE-DECOY PDZ domain-containing 2 OS=Mus musculus OX=10090 GN=Pdzd2...	
tr|Q9CPN9|Q9CPN9_MOUSE RIKEN cDNA 2210010C04 gene OS=Mus musculus OX=10090 GN=2210010C04Rik...	tr|Q9CPN9|Q9CPN9_MOUSE RIKEN cDNA 2210010C04 gene OS=Mus musculus OX=10090 GN=2210010C04Rik...	
sp|Q8CI03-2|FWCH1_MOUSE-DECOY Isoform 2 of FLYWCH-type zinc finger-containing protein...	sp|Q8CI03-2|FWCH1_MOUSE-DECOY Isoform 2 of FLYWCH-type zinc finger-containing protein...	
sp|Q9QXS1-6|PLEC_MOUSE-DECOY Isoform PLEC-0,1C of Plectin OS=Mus musculus OX=10090...	sp|Q9QXS1-6|PLEC_MOUSE-DECOY Isoform PLEC-0,1C of Plectin OS=Mus musculus OX=10090...	
sp|O88196-2|TTC3_MOUSE-DECOY Isoform 2 of E3 ubiquitin-protein ligase TTC3 OS=Mus...	sp|O88196-2|TTC3_MOUSE-DECOY Isoform 2 of E3 ubiquitin-protein ligase TTC3 OS=Mus...	
sp|Q8BI29-2|SARG_MOUSE Isoform 2 of Specifically androgen-regulated gene protein...	sp|Q8BI29-2|SARG_MOUSE Isoform 2 of Specifically androgen-regulated gene protein...	
tr|D3Z794|D3Z794_MOUSE Small ubiquitin-related modifier 2 OS=Mus musculus OX=10090...	tr|D3Z794|D3Z794_MOUSE Small ubiquitin-related modifier 2 OS=Mus musculus OX=10090...	
tr|D3YYK9|D3YYK9_MOUSE-DECOY Brefeldin A-inhibited guanine nucleotide-exchange protein...	tr|D3YYK9|D3YYK9_MOUSE-DECOY Brefeldin A-inhibited guanine nucleotide-exchange protein...	
tr|A0A158SIT8|A0A158SIT8_MOUSE Proline, glutamic acid and leucine rich protein 1...	tr|A0A158SIT8|A0A158SIT8_MOUSE Proline, glutamic acid and leucine rich protein 1...	
tr|B2RUJ4|B2RUJ4_MOUSE-DECOY Atad5 protein OS=Mus musculus OX=10090 GN=Atad5 PE=2...	tr|B2RUJ4|B2RUJ4_MOUSE-DECOY Atad5 protein OS=Mus musculus OX=10090 GN=Atad5 PE=2...	
tr|F8VPK0|F8VPK0_MOUSE Tetratricopeptide repeat domain 37 OS=Mus musculus OX=10090...	tr|F8VPK0|F8VPK0_MOUSE Tetratricopeptide repeat domain 37 OS=Mus musculus OX=10090...	
sp|P53349|M3K1_MOUSE-DECOY Mitogen-activated protein kinase kinase kinase 1 OS=Mus...	sp|P53349|M3K1_MOUSE-DECOY Mitogen-activated protein kinase kinase kinase 1 OS=Mus...	
Q8VED4_MOUSE	Serine/threonine-protein phosphatase OS=Mus musculus OX=10090 GN=Ppp2cb PE=2 SV=1	
sp|Q05860-3|FMN1_MOUSE-DECOY Isoform 3 of Formin-1 OS=Mus musculus OX=10090 GN=Fmn1	sp|Q05860-3|FMN1_MOUSE-DECOY Isoform 3 of Formin-1 OS=Mus musculus OX=10090 GN=Fmn1	
tr|B2RX10|B2RX10_MOUSE-DECOY Fanconi anemia, complementation group M OS=Mus musculus...	tr|B2RX10|B2RX10_MOUSE-DECOY Fanconi anemia, complementation group M OS=Mus musculus...	
sp|Q69ZA1-2|CDK13_MOUSE Isoform 2 of Cyclin-dependent kinase 13 OS=Mus musculus OX=10090...	Isoform 2 of Cyclin-dependent kinase 13 OS=Mus musculus OX=10090 GN=Cdk13	
tr|A0A087WSS1|A0A087WSS1_MOUSE RIKEN cDNA 2310035C23 gene OS=Mus musculus OX=10090...	tr|A0A087WSS1|A0A087WSS1_MOUSE RIKEN cDNA 2310035C23 gene OS=Mus musculus OX=10090...	
tr|Q3TEK2|Q3TEK2_MOUSE-DECOY Uncharacterized protein OS=Mus musculus OX=10090 GN=Hspa8...	tr|Q3TEK2|Q3TEK2_MOUSE-DECOY Uncharacterized protein OS=Mus musculus OX=10090 GN=Hspa8...	
sp|O88491|NSD1_MOUSE-DECOY Histone-lysine N-methyltransferase, H3 lysine-36 and H4...	sp|O88491|NSD1_MOUSE-DECOY Histone-lysine N-methyltransferase, H3 lysine-36 and H4...	
sp|A0JP43-3|EFCB5_MOUSE-DECOY Isoform 3 of EF-hand calcium-binding domain-containing...	sp|A0JP43-3|EFCB5_MOUSE-DECOY Isoform 3 of EF-hand calcium-binding domain-containing...	
sp|Q9DB77|QCR2_MOUSE Cytochrome b-c1 complex subunit 2, mitochondrial OS=Mus musculus...	sp|Q9DB77|QCR2_MOUSE Cytochrome b-c1 complex subunit 2, mitochondrial OS=Mus musculus...	
sp|Q9D0T1|NH2L1_MOUSENHP2-like protein 1 OS=Mus musculus OX=10090 GN=Snu13 PE=1 SV=4	sp|Q9D0T1|NH2L1_MOUSE NHP2-like protein 1 OS=Mus musculus OX=10090 GN=Snu13 PE=1 SV=4	
tr|Q3UG63|Q3UG63_MOUSE Uncharacterized protein OS=Mus musculus OX=10090 GN=Ascc2...	tr|Q3UG63|Q3UG63_MOUSE Uncharacterized protein OS=Mus musculus OX=10090 GN=Ascc2...	
tr|Q545N7|Q545N7_MOUSE Creatine kinase, mitochondrial 1, ubiquitous, isoform CRA_a...	tr|Q545N7|Q545N7_MOUSE Creatine kinase, mitochondrial 1, ubiquitous, isoform CRA_a...	
sp|Q05512-2|MARK2_MOUSE-DECOY Isoform 2 of Serine/threonine-protein kinase MARK2...	sp|Q05512-2|MARK2_MOUSE-DECOY Isoform 2 of Serine/threonine-protein kinase MARK2...	
sp|Q99N50-2|SYTL2_MOUSE Isoform 2 of Synaptotagmin-like protein 2 OS=Mus musculus...	sp|Q99N50-2|SYTL2_MOUSE Isoform 2 of Synaptotagmin-like protein 2 OS=Mus musculus...	
tr|Q3U0X6|Q3U0X6_MOUSE-DECOY Zinc finger protein 217 OS=Mus musculus OX=10090 GN=Zfp217...	tr|Q3U0X6|Q3U0X6_MOUSE-DECOY Zinc finger protein 217 OS=Mus musculus OX=10090 GN=Zfp217...	
A2AKU9_MOUSE	ATP synthase subunit gamma OS=Mus musculus OX=10090 GN=Atp5c1 PE=1 SV=1	
Q8K366_MOUSE	Exosc10 protein OS=Mus musculus OX=10090 GN=Exosc10 PE=1 SV=1	
ENOB_MOUSE	Beta-enolase OS=Mus musculus OX=10090 GN=Eno3 PE=1 SV=3	
sp|Q9CXF7-2|CHD1L_MOUSE Isoform 2 of Chromodomain-helicase-DNA-binding protein 1-like...	Isoform 2 of Chromodomain-helicase-DNA-binding protein 1-like OS=Mus musculus OX=10090 GN=Chd1l	
sp|Q99K01-2|PDXD1_MOUSE Isoform 2 of Pyridoxal-dependent decarboxylase domain-containing...	sp|Q99K01-2|PDXD1_MOUSE Isoform 2 of Pyridoxal-dependent decarboxylase domain-containing...	
tr|Q3TTI6|Q3TTI6_MOUSE Uncharacterized protein (Fragment) OS=Mus musculus OX=10090...	tr|Q3TTI6|Q3TTI6_MOUSE Uncharacterized protein (Fragment) OS=Mus musculus OX=10090...	
Q3U2B4_MOUSE	Uncharacterized protein OS=Mus musculus OX=10090 GN=Xrcc1 PE=2 SV=1	
E9Q4D0_MOUSE	Proprotein convertase subtilisin/kexin type 6 OS=Mus musculus OX=10090 GN=Pcsk6 PE=1 SV=2	
sp|Q3TZZ7|ESYT2_MOUSEExtended synaptotagmin-2 OS=Mus musculus OX=10090 GN=Esyt2 PE=1 SV=1	sp|Q3TZZ7|ESYT2_MOUSE Extended synaptotagmin-2 OS=Mus musculus OX=10090 GN=Esyt2 PE=1 SV=1	
sp|Q61037-2|TSC2_MOUSE	Isoform A of Tuberin OS=Mus musculus OX=10090 GN=Tsc2	
tr|A3KGJ7|A3KGJ7_MOUSE-DECOY Centrosome-associated protein CEP250 OS=Mus musculus...	tr|A3KGJ7|A3KGJ7_MOUSE-DECOY Centrosome-associated protein CEP250 OS=Mus musculus...	
A2AFK7_MOUSE	Eukaryotic initiation factor 4A-III (Fragment) OS=Mus musculus OX=10090 GN=Eif4a3 PE=1 SV=1	
E0CXV8_MOUSE	Polyhomeotic-like protein 1 OS=Mus musculus OX=10090 GN=Phc1 PE=1 SV=1	
Q3UDM0_MOUSE	MOB kinase activator 1B OS=Mus musculus OX=10090 GN=Mob1b PE=1 SV=1	
sp|P30416|FKBP4_MOUSEPeptidyl-prolyl cis-trans isomerase FKBP4 OS=Mus musculus OX=10090 GN=Fkbp4 PE=1 SV=5	sp|P30416|FKBP4_MOUSE Peptidyl-prolyl cis-trans isomerase FKBP4 OS=Mus musculus OX=10090 GN=Fkbp4 PE=1 SV=5	
A2A4N7_MOUSE	Next to BRCA1 gene 1 protein OS=Mus musculus OX=10090 GN=Nbr1 PE=1 SV=1	
tr|B1AUX2|B1AUX2_MOUSE-DECOY Host cell factor 1 OS=Mus musculus OX=10090 GN=Hcfc1...	tr|B1AUX2|B1AUX2_MOUSE-DECOY Host cell factor 1 OS=Mus musculus OX=10090 GN=Hcfc1...	
tr|F8WIE5|F8WIE5_MOUSE-DECOY E3 ubiquitin-protein ligase HECTD1 OS=Mus musculus OX=10090...	tr|F8WIE5|F8WIE5_MOUSE-DECOY E3 ubiquitin-protein ligase HECTD1 OS=Mus musculus OX=10090...	
E9Q805_MOUSE	FERM, ARHGEF and pleckstrin domain-containing protein 1 (Fragment) OS=Mus musculus OX=10090 GN=Farp1 PE=1 SV=1	
sp|Q8CH77-2|NAV1_MOUSE	Isoform 2 of Neuron navigator 1 OS=Mus musculus OX=10090 GN=Nav1	
tr|E9QPI5|E9QPI5_MOUSE-DECOY Sister chromatid cohesion protein PDS5 homolog A OS=Mus...	tr|E9QPI5|E9QPI5_MOUSE-DECOY Sister chromatid cohesion protein PDS5 homolog A OS=Mus...	
sp|E9Q8I9|FRY_MOUSE-DECOY Protein furry homolog OS=Mus musculus OX=10090 GN=Fry PE=1...	sp|E9Q8I9|FRY_MOUSE-DECOY Protein furry homolog OS=Mus musculus OX=10090 GN=Fry PE=1...	
tr|F8WJD4|F8WJD4_MOUSE-DECOY Symplekin OS=Mus musculus OX=10090 GN=Sympk PE=1 SV=1	tr|F8WJD4|F8WJD4_MOUSE-DECOY Symplekin OS=Mus musculus OX=10090 GN=Sympk PE=1 SV=1	
Q8C1V4_MOUSE	Lysine--tRNA ligase OS=Mus musculus OX=10090 GN=Kars PE=2 SV=1	
tr|B2RXX9|B2RXX9_MOUSE-DECOY Myosin, heavy polypeptide 7, cardiac muscle, beta OS=Mus...	tr|B2RXX9|B2RXX9_MOUSE-DECOY Myosin, heavy polypeptide 7, cardiac muscle, beta OS=Mus...	
sp|Q5SSL4|ABR_MOUSE Active breakpoint cluster region-related protein OS=Mus musculus...	sp|Q5SSL4|ABR_MOUSE Active breakpoint cluster region-related protein OS=Mus musculus...	
Q3U2K1_MOUSE	Uncharacterized protein (Fragment) OS=Mus musculus OX=10090 GN=Prmt3 PE=2 SV=1	
sp|Q91WJ8-2|FUBP1_MOUSE Isoform 2 of Far upstream element-binding protein 1 OS=Mus...	Isoform 2 of Far upstream element-binding protein 1 OS=Mus musculus OX=10090 GN=Fubp1	
A2A601_MOUSE	Importin subunit alpha-1 (Fragment) OS=Mus musculus OX=10090 GN=Kpna2 PE=1 SV=1	
DPP9_MOUSE	Dipeptidyl peptidase 9 OS=Mus musculus OX=10090 GN=Dpp9 PE=1 SV=2	
UFL1_MOUSE	E3 UFM1-protein ligase 1 OS=Mus musculus OX=10090 GN=Ufl1 PE=1 SV=2	
sp|Q4JK59|TET2_MOUSE-DECOY Methylcytosine dioxygenase TET2 OS=Mus musculus OX=10090...	sp|Q4JK59|TET2_MOUSE-DECOY Methylcytosine dioxygenase TET2 OS=Mus musculus OX=10090...	
sp|Q6PH08-3|ERC2_MOUSE	Isoform 3 of ERC protein 2 OS=Mus musculus OX=10090 GN=Erc2	
sp|Q9CXT8|MPPB_MOUSE Mitochondrial-processing peptidase subunit beta OS=Mus musculus...	Mitochondrial-processing peptidase subunit beta OS=Mus musculus OX=10090 GN=Pmpcb PE=1 SV=1	
tr|A0A0U1RPD8|A0A0U1RPD8_MOUSE General transcription factor 3C polypeptide 1 OS=Mus...	tr|A0A0U1RPD8|A0A0U1RPD8_MOUSE General transcription factor 3C polypeptide 1 OS=Mus...	
tr|A0A1D5RMI8|A0A1D5RMI8_MOUSE-DECOY Alstrom syndrome protein 1 homolog OS=Mus musculus...	tr|A0A1D5RMI8|A0A1D5RMI8_MOUSE-DECOY Alstrom syndrome protein 1 homolog OS=Mus musculus...	
tr|Q3TM70|Q3TM70_MOUSE EH-domain containing 4 OS=Mus musculus OX=10090 GN=Ehd4 PE=1...	EH-domain containing 4 OS=Mus musculus OX=10090 GN=Ehd4 PE=1 SV=1	
tr|Q8BL63|Q8BL63_MOUSE GPI-anchor transamidase OS=Mus musculus OX=10090 GN=Pigk PE=1...	tr|Q8BL63|Q8BL63_MOUSE GPI-anchor transamidase OS=Mus musculus OX=10090 GN=Pigk PE=1...	
MECP2_MOUSE	Isoform B of Methyl-CpG-binding protein 2 OS=Mus musculus OX=10090 GN=Mecp2	
sp|Q91XQ0-2|DYH8_MOUSE-DECOY Isoform 2 of Dynein heavy chain 8, axonemal OS=Mus musculus...	sp|Q91XQ0-2|DYH8_MOUSE-DECOY Isoform 2 of Dynein heavy chain 8, axonemal OS=Mus musculus...	
tr|E9Q718|E9Q718_MOUSE Procollagen-lysine,2-oxoglutarate 5-dioxygenase 2 OS=Mus musculus...	Procollagen-lysine,2-oxoglutarate 5-dioxygenase 2 OS=Mus musculus OX=10090 GN=Plod2 PE=1 SV=1	
tr|F6WI02|F6WI02_MOUSE Stomatin-like protein 2, mitochondrial (Fragment) OS=Mus musculus...	Stomatin-like protein 2, mitochondrial (Fragment) OS=Mus musculus OX=10090 GN=Stoml2 PE=1 SV=1	
tr|Q3TPN9|Q3TPN9_MOUSE Uncharacterized protein OS=Mus musculus OX=10090 GN=Chuk PE=2...	tr|Q3TPN9|Q3TPN9_MOUSE Uncharacterized protein OS=Mus musculus OX=10090 GN=Chuk PE=2...	
tr|E9Q526|E9Q526_MOUSE C2 domain-containing protein 3 OS=Mus musculus OX=10090 GN=C2cd3...	tr|E9Q526|E9Q526_MOUSE C2 domain-containing protein 3 OS=Mus musculus OX=10090 GN=C2cd3...	
E9Q0R3_MOUSE	Gamma-tubulin complex component OS=Mus musculus OX=10090 GN=Tubgcp3 PE=1 SV=1	
GPC4_MOUSE	Glypican-4 OS=Mus musculus OX=10090 GN=Gpc4 PE=1 SV=2	
sp|Q99JY0|ECHB_MOUSE Trifunctional enzyme subunit beta, mitochondrial OS=Mus musculus...	sp|Q99JY0|ECHB_MOUSE Trifunctional enzyme subunit beta, mitochondrial OS=Mus musculus...	
Q80Y25_MOUSE	Capn1 protein OS=Mus musculus OX=10090 GN=Capn1 PE=2 SV=1	
Q8CGI3_MOUSE	Sfrs11 protein (Fragment) OS=Mus musculus OX=10090 GN=Srsf11 PE=2 SV=1	
Q9CXZ9_MOUSE	Uncharacterized protein OS=Mus musculus OX=10090 GN=Galk1 PE=2 SV=1	
RL32_MOUSE	60S ribosomal protein L32 OS=Mus musculus OX=10090 GN=Rpl32 PE=1 SV=2	
sp|Q9D4H8|CUL2_MOUSE	Cullin-2 OS=Mus musculus OX=10090 GN=Cul2 PE=1 SV=2	
sp|Q9Z110-2|P5CS_MOUSE Isoform Short of Delta-1-pyrroline-5-carboxylate synthase...	Isoform Short of Delta-1-pyrroline-5-carboxylate synthase OS=Mus musculus OX=10090 GN=Aldh18a1	
RT34_MOUSE	28S ribosomal protein S34, mitochondrial OS=Mus musculus OX=10090 GN=Mrps34 PE=1 SV=1	
tr|E9Q1M1|E9Q1M1_MOUSE PDZ domain-containing 2 OS=Mus musculus OX=10090 GN=Pdzd2...	tr|E9Q1M1|E9Q1M1_MOUSE PDZ domain-containing 2 OS=Mus musculus OX=10090 GN=Pdzd2...	
sp|Q059U7|INTU_MOUSE	Protein inturned OS=Mus musculus OX=10090 GN=Intu PE=1 SV=1	
tr|A0A2I3BQ39|A0A2I3BQ39_MOUSE Ubiquitin carboxyl-terminal hydrolase (Fragment) OS=Mus...	tr|A0A2I3BQ39|A0A2I3BQ39_MOUSE Ubiquitin carboxyl-terminal hydrolase (Fragment) OS=Mus...	
tr|Q3UFZ4|Q3UFZ4_MOUSE Uncharacterized protein OS=Mus musculus OX=10090 GN=Nol10...	tr|Q3UFZ4|Q3UFZ4_MOUSE Uncharacterized protein OS=Mus musculus OX=10090 GN=Nol10...	
tr|Q3U7E6|Q3U7E6_MOUSE Uncharacterized protein OS=Mus musculus OX=10090 GN=Surf4...	tr|Q3U7E6|Q3U7E6_MOUSE Uncharacterized protein OS=Mus musculus OX=10090 GN=Surf4...	
G3X9G2_MOUSE	Misshapen-like kinase 1 OS=Mus musculus OX=10090 GN=Mink1 PE=1 SV=1	
G3X981_MOUSE	Peripherin OS=Mus musculus OX=10090 GN=Prph PE=1 SV=1	
tr|B2RT41|B2RT41_MOUSE-DECOY Zinc finger, C3H1-type-containing OS=Mus musculus OX=10090...	tr|B2RT41|B2RT41_MOUSE-DECOY Zinc finger, C3H1-type-containing OS=Mus musculus OX=10090...	
sp|Q8BTM8|FLNA_MOUSE-DECOY Filamin-A OS=Mus musculus OX=10090 GN=Flna PE=1 SV=5	sp|Q8BTM8|FLNA_MOUSE-DECOY Filamin-A OS=Mus musculus OX=10090 GN=Flna PE=1 SV=5	
sp|Q80X41-2|VRK1_MOUSE Isoform 2 of Serine/threonine-protein kinase VRK1 OS=Mus musculus...	Isoform 2 of Serine/threonine-protein kinase VRK1 OS=Mus musculus OX=10090 GN=Vrk1	
A2AA71_MOUSE	Protein transport protein Sec24A OS=Mus musculus OX=10090 GN=Sec24a PE=1 SV=1	
ARL1_MOUSE	ADP-ribosylation factor-like protein 1 OS=Mus musculus OX=10090 GN=Arl1 PE=1 SV=1	
tr|Q14DP3|Q14DP3_MOUSE Leucyl-tRNA synthetase, mitochondrial OS=Mus musculus OX=10090...	Leucyl-tRNA synthetase, mitochondrial OS=Mus musculus OX=10090 GN=Lars2 PE=2 SV=1	
B2RXS1_MOUSE	Nbeal2 protein OS=Mus musculus OX=10090 GN=Nbeal2 PE=2 SV=1	
sp|A2AN08-3|UBR4_MOUSE-DECOY Isoform 3 of E3 ubiquitin-protein ligase UBR4 OS=Mus...	sp|A2AN08-3|UBR4_MOUSE-DECOY Isoform 3 of E3 ubiquitin-protein ligase UBR4 OS=Mus...	
tr|Q3TJX6|Q3TJX6_MOUSE Uncharacterized protein OS=Mus musculus OX=10090 GN=Utp18...	tr|Q3TJX6|Q3TJX6_MOUSE Uncharacterized protein OS=Mus musculus OX=10090 GN=Utp18...	
tr|Q3UQJ9|Q3UQJ9_MOUSE Uncharacterized protein OS=Mus musculus OX=10090 GN=Trps1...	tr|Q3UQJ9|Q3UQJ9_MOUSE Uncharacterized protein OS=Mus musculus OX=10090 GN=Trps1...	
tr|A0A286YDA2|A0A286YDA2_MOUSE Nucleolar and coiled-body phosphoprotein 1 OS=Mus...	tr|A0A286YDA2|A0A286YDA2_MOUSE Nucleolar and coiled-body phosphoprotein 1 OS=Mus...	
sp|P08551|NFL_MOUSE Neurofilament light polypeptide OS=Mus musculus OX=10090 GN=Nefl...	Neurofilament light polypeptide OS=Mus musculus OX=10090 GN=Nefl PE=1 SV=5	
sp|Q8BH58|TIPRL_MOUSETIP41-like protein OS=Mus musculus OX=10090 GN=Tiprl PE=1 SV=1	sp|Q8BH58|TIPRL_MOUSE TIP41-like protein OS=Mus musculus OX=10090 GN=Tiprl PE=1 SV=1	
tr|A0A0R4J1W7|A0A0R4J1W7_MOUSE CDC23 (Cell division cycle 23, yeast, homolog), isoform...	tr|A0A0R4J1W7|A0A0R4J1W7_MOUSE CDC23 (Cell division cycle 23, yeast, homolog), isoform...	
tr|Q6PET9|Q6PET9_MOUSE DNA topoisomerase 2 (Fragment) OS=Mus musculus OX=10090 GN=Top2a...	tr|Q6PET9|Q6PET9_MOUSE DNA topoisomerase 2 (Fragment) OS=Mus musculus OX=10090 GN=Top2a...	
Q5U4B1_MOUSE	Replication factor C subunit 1 OS=Mus musculus OX=10090 GN=Rfc1 PE=1 SV=1	
Q3US09_MOUSE	Uncharacterized protein (Fragment) OS=Mus musculus OX=10090 GN=Thoc2 PE=2 SV=1	
tr|Q3UGZ4|Q3UGZ4_MOUSE-DECOY Spectrin beta chain OS=Mus musculus OX=10090 GN=Sptbn2...	tr|Q3UGZ4|Q3UGZ4_MOUSE-DECOY Spectrin beta chain OS=Mus musculus OX=10090 GN=Sptbn2...	
B2RUG9_MOUSE	Adenomatosis polyposis coli OS=Mus musculus OX=10090 GN=Apc PE=1 SV=1	
sp|Q9Z0H8-2|CLIP2_MOUSE Isoform 2 of CAP-Gly domain-containing linker protein 2 OS=Mus...	sp|Q9Z0H8-2|CLIP2_MOUSE Isoform 2 of CAP-Gly domain-containing linker protein 2 OS=Mus...	
sp|Q8C1B7-3|SEP11_MOUSE	Isoform 3 of Septin-11 OS=Mus musculus OX=10090 GN=Sept11	
sp|F8VPN2-2|TEX15_MOUSE Isoform 2 of Testis-expressed protein 15 OS=Mus musculus...	Isoform 2 of Testis-expressed protein 15 OS=Mus musculus OX=10090 GN=Tex15	
tr|Q4FE56|Q4FE56_MOUSE Probable ubiquitin carboxyl-terminal hydrolase FAF-X OS=Mus...	tr|Q4FE56|Q4FE56_MOUSE Probable ubiquitin carboxyl-terminal hydrolase FAF-X OS=Mus...	
tr|A0A1D5RLM8|A0A1D5RLM8_MOUSE-DECOY Predicted gene 11639 OS=Mus musculus OX=10090...	tr|A0A1D5RLM8|A0A1D5RLM8_MOUSE-DECOY Predicted gene 11639 OS=Mus musculus OX=10090...	
tr|F6RGI3|F6RGI3_MOUSE-DECOY Predicted gene 340 OS=Mus musculus OX=10090 GN=Gm340...	tr|F6RGI3|F6RGI3_MOUSE-DECOY Predicted gene 340 OS=Mus musculus OX=10090 GN=Gm340...	
sp|Q60749|KHDR1_MOUSEKH domain-containing, RNA-binding, signal transduction-associated protein 1 OS=Mus musculus OX=10090 GN=Khdrbs1 PE=1 SV=2	sp|Q60749|KHDR1_MOUSE KH domain-containing, RNA-binding, signal transduction-associated protein 1 OS=Mus musculus OX=10090 GN=Khdrbs1 PE=1 SV=2	
tr|A2A9H6|A2A9H6_MOUSE-DECOY Predicted gene 884 OS=Mus musculus OX=10090 GN=Gm884...	tr|A2A9H6|A2A9H6_MOUSE-DECOY Predicted gene 884 OS=Mus musculus OX=10090 GN=Gm884...	
tr|E9Q4K7|E9Q4K7_MOUSE Kinesin family member 13B OS=Mus musculus OX=10090 GN=Kif13b...	tr|E9Q4K7|E9Q4K7_MOUSE Kinesin family member 13B OS=Mus musculus OX=10090 GN=Kif13b...	
tr|A0A087WS83|A0A087WS83_MOUSE Voltage-dependent R-type calcium channel subunit alpha...	tr|A0A087WS83|A0A087WS83_MOUSE Voltage-dependent R-type calcium channel subunit alpha...	
sp|P27048|RSMB_MOUSE Small nuclear ribonucleoprotein-associated protein B OS=Mus...	sp|P27048|RSMB_MOUSE Small nuclear ribonucleoprotein-associated protein B OS=Mus...	
sp|Q8K2Q7|BROX_MOUSE BRO1 domain-containing protein BROX OS=Mus musculus OX=10090...	sp|Q8K2Q7|BROX_MOUSE BRO1 domain-containing protein BROX OS=Mus musculus OX=10090...	
tr|B9EKS2|B9EKS2_MOUSE-DECOY Jumonji domain containing 1B OS=Mus musculus OX=10090...	tr|B9EKS2|B9EKS2_MOUSE-DECOY Jumonji domain containing 1B OS=Mus musculus OX=10090...	
Q9D3C4_MOUSE	Actin-related protein 2/3 complex subunit 4 OS=Mus musculus OX=10090 GN=Arpc4 PE=2 SV=1	
sp|Q0KL02-3|TRIO_MOUSE	Isoform 3 of Triple functional domain protein OS=Mus musculus OX=10090 GN=Trio	
Q3KR45_MOUSE	DNA (cytosine-5)-methyltransferase 3B OS=Mus musculus OX=10090 GN=Dnmt3b PE=1 SV=1	
sp|Q8R5H1-5|UBP15_MOUSE Isoform 5 of Ubiquitin carboxyl-terminal hydrolase 15 OS=Mus...	Isoform 5 of Ubiquitin carboxyl-terminal hydrolase 15 OS=Mus musculus OX=10090 GN=Usp15	
A9JR55_MOUSE	Mybpc3 protein OS=Mus musculus OX=10090 GN=Mybpc3 PE=2 SV=1	
BIG2_MOUSE	Brefeldin A-inhibited guanine nucleotide-exchange protein 2 OS=Mus musculus OX=10090 GN=Arfgef2 PE=1 SV=1	
A0A1B0GRJ4_MOUSE	Predicted gene 7579 OS=Mus musculus OX=10090 GN=Gm7579 PE=4 SV=1	
Q3TZU7_MOUSE	Sorting nexin OS=Mus musculus OX=10090 GN=Snx9 PE=2 SV=1	
sp|Q9WTU3-3|SCN8A_MOUSE-DECOY Isoform 3 of Sodium channel protein type 8 subunit...	sp|Q9WTU3-3|SCN8A_MOUSE-DECOY Isoform 3 of Sodium channel protein type 8 subunit...	
Q8CGG9_MOUSE	Fes protein (Fragment) OS=Mus musculus OX=10090 GN=Fes PE=2 SV=1	
B1ARD8_MOUSE	Schlafen 8 OS=Mus musculus OX=10090 GN=Slfn8 PE=1 SV=1	
Q7TPY0_MOUSE	Gtf2h1 protein OS=Mus musculus OX=10090 GN=Gtf2h1 PE=2 SV=1	
KC1E_MOUSE	Casein kinase I isoform epsilon OS=Mus musculus OX=10090 GN=Csnk1e PE=1 SV=2	
B7ZMR9_MOUSE	MCG12507, isoform CRA_a OS=Mus musculus OX=10090 GN=Zfp800 PE=1 SV=1	
sp|Q6P5B0|RRP12_MOUSERRP12-like protein OS=Mus musculus OX=10090 GN=Rrp12 PE=1 SV=1	sp|Q6P5B0|RRP12_MOUSE RRP12-like protein OS=Mus musculus OX=10090 GN=Rrp12 PE=1 SV=1	
DVL2_MOUSE	Segment polarity protein dishevelled homolog DVL-2 OS=Mus musculus OX=10090 GN=Dvl2 PE=1 SV=2	
ADT1_MOUSE	ADP/ATP translocase 1 OS=Mus musculus OX=10090 GN=Slc25a4 PE=1 SV=4	
sp|Q9R269|PEPL_MOUSE-DECOY Periplakin OS=Mus musculus OX=10090 GN=Ppl PE=1 SV=1	sp|Q9R269|PEPL_MOUSE-DECOY Periplakin OS=Mus musculus OX=10090 GN=Ppl PE=1 SV=1	
sp|Q61102|ABCB7_MOUSEATP-binding cassette sub-family B member 7, mitochondrial OS=Mus musculus OX=10090 GN=Abcb7 PE=1 SV=3	sp|Q61102|ABCB7_MOUSE ATP-binding cassette sub-family B member 7, mitochondrial OS=Mus musculus OX=10090 GN=Abcb7 PE=1 SV=3	
Q3TL71_MOUSE	Uncharacterized protein OS=Mus musculus OX=10090 GN=Hnrnpk PE=2 SV=1	
A0A286YD72_MOUSE	Voltage-dependent L-type calcium channel subunit alpha OS=Mus musculus OX=10090 GN=Cacna1d PE=1 SV=1	
B1B1A8_MOUSE	Myosin light chain kinase, smooth muscle OS=Mus musculus OX=10090 GN=Mylk PE=1 SV=1	
Q7TQR2_MOUSE	Olfactory receptor OS=Mus musculus OX=10090 GN=Olfr1465 PE=3 SV=1	
sp|Q3UYI5|RGL3_MOUSE	Ral guanine nucleotide dissociation stimulator-like 3 OS=Mus musculus OX=10090 GN=Rgl3 PE=1 SV=2	
Q6A049_MOUSE	MKIAA0546 protein (Fragment) OS=Mus musculus OX=10090 GN=Zfc3h1 PE=2 SV=1	
Q3TXH6_MOUSE	Uncharacterized protein OS=Mus musculus OX=10090 GN=Smarcd2 PE=2 SV=1	
A2A9H6_MOUSE	Predicted gene 884 OS=Mus musculus OX=10090 GN=Gm884 PE=1 SV=1	
Q9D122_MOUSE	Uncharacterized protein OS=Mus musculus OX=10090 GN=Krtap4-2 PE=2 SV=1	
Q9JJD8_MOUSE	Brain cDNA, clone MNCb-1272, similar to Mus musculus chaperonin subunit 2 (beta) (Cct2), mRNA OS=Mus musculus OX=10090 GN=Cct2 PE=2 SV=1	
SMRCD_MOUSE	Isoform 2 of SWI/SNF-related matrix-associated actin-dependent regulator of chromatin subfamily A containing DEAD/H box 1 OS=Mus musculus OX=10090 GN=Smarcad1	
E9PWI6_MOUSE	GRIP and coiled-coil domain-containing protein 2 OS=Mus musculus OX=10090 GN=Gcc2 PE=1 SV=1	
O88348_MOUSE	Deubiquitinating enzyme (Fragment) OS=Mus musculus OX=10090 GN=Usp12 PE=2 SV=1	
S4R1W5_MOUSE	RNA-binding motif protein 6 OS=Mus musculus OX=10090 GN=Rbm6 PE=1 SV=1	
sp|P97480-2|EYA3_MOUSE	Isoform 2 of Eyes absent homolog 3 OS=Mus musculus OX=10090 GN=Eya3	
E9QP54_MOUSE	Jouberin OS=Mus musculus OX=10090 GN=Ahi1 PE=1 SV=1	
B2KFD2_MOUSE	2810474O19Rik protein OS=Mus musculus OX=10090 GN=2810474O19Rik PE=2 SV=1	
sp|Q8BW94-2|DYH3_MOUSE	Isoform 2 of Dynein heavy chain 3, axonemal OS=Mus musculus OX=10090 GN=Dnah3	
E9QKE4_MOUSE	Rab3 GTPase-activating protein non-catalytic subunit OS=Mus musculus OX=10090 GN=Rab3gap2 PE=1 SV=1	
DDX6_MOUSE	Probable ATP-dependent RNA helicase DDX6 OS=Mus musculus OX=10090 GN=Ddx6 PE=1 SV=1	
MSH6_MOUSE	DNA mismatch repair protein Msh6 OS=Mus musculus OX=10090 GN=Msh6 PE=1 SV=3	
A0A0R4J1Y3_MOUSE	Probable tRNA N6-adenosine threonylcarbamoyltransferase OS=Mus musculus OX=10090 GN=Osgep PE=1 SV=1	
D3YZF6_MOUSE	Family with sequence similarity 205, member A1 OS=Mus musculus OX=10090 GN=Fam205a1 PE=4 SV=1	
Q3UPA1_MOUSE	Guanine nucleotide binding protein, alpha 11 OS=Mus musculus OX=10090 GN=Gna11 PE=1 SV=1	
sp|P70451|FER_MOUSE Tyrosine-protein kinase Fer OS=Mus musculus OX=10090 GN=Fer PE=1...	sp|P70451|FER_MOUSE Tyrosine-protein kinase Fer OS=Mus musculus OX=10090 GN=Fer PE=1...	
sp|Q3UH68-3|LIMC1_MOUSE Isoform 3 of LIM and calponin homology domains-containing...	sp|Q3UH68-3|LIMC1_MOUSE Isoform 3 of LIM and calponin homology domains-containing...	
sp|Q8CF89|TAB1_MOUSE TGF-beta-activated kinase 1 and MAP3K7-binding protein 1 OS=Mus...	sp|Q8CF89|TAB1_MOUSE TGF-beta-activated kinase 1 and MAP3K7-binding protein 1 OS=Mus...	
sp|Q8R2N2|UTP4_MOUSE U3 small nucleolar RNA-associated protein 4 homolog OS=Mus musculus...	U3 small nucleolar RNA-associated protein 4 homolog OS=Mus musculus OX=10090 GN=Utp4 PE=2 SV=3	
sp|Q8VI56-2|LRP4_MOUSE-DECOY Isoform 2 of Low-density lipoprotein receptor-related...	sp|Q8VI56-2|LRP4_MOUSE-DECOY Isoform 2 of Low-density lipoprotein receptor-related...	
sp|Q9Z0R6-2|ITSN2_MOUSE-DECOY Isoform 2 of Intersectin-2 OS=Mus musculus OX=10090...	sp|Q9Z0R6-2|ITSN2_MOUSE-DECOY Isoform 2 of Intersectin-2 OS=Mus musculus OX=10090...	
tr|B2RY21|B2RY21_MOUSE-DECOY Abca12 protein OS=Mus musculus OX=10090 GN=Abca12 PE=2...	tr|B2RY21|B2RY21_MOUSE-DECOY Abca12 protein OS=Mus musculus OX=10090 GN=Abca12 PE=2...	
tr|F6VUT6|F6VUT6_MOUSE-DECOY Predicted gene 8251 OS=Mus musculus OX=10090 GN=Gm8251...	tr|F6VUT6|F6VUT6_MOUSE-DECOY Predicted gene 8251 OS=Mus musculus OX=10090 GN=Gm8251...	
tr|G5E8B1|G5E8B1_MOUSE Protein tyrosine phosphatase, non-receptor type 13 OS=Mus...	tr|G5E8B1|G5E8B1_MOUSE Protein tyrosine phosphatase, non-receptor type 13 OS=Mus...	
tr|Q6NV50|Q6NV50_MOUSE-DECOY Catenin (Cadherin associated protein), alpha 1 OS=Mus...	tr|Q6NV50|Q6NV50_MOUSE-DECOY Catenin (Cadherin associated protein), alpha 1 OS=Mus...	
tr|Q8C1T2|Q8C1T2_MOUSE Uncharacterized protein OS=Mus musculus OX=10090 GN=Psmd6...	tr|Q8C1T2|Q8C1T2_MOUSE Uncharacterized protein OS=Mus musculus OX=10090 GN=Psmd6...	
tr|Q9CTM9|Q9CTM9_MOUSE Uncharacterized protein (Fragment) OS=Mus musculus OX=10090...	tr|Q9CTM9|Q9CTM9_MOUSE Uncharacterized protein (Fragment) OS=Mus musculus OX=10090...	
tr|Q9D3V6|Q9D3V6_MOUSE Core histone macro-H2A OS=Mus musculus OX=10090 GN=H2afy3...	Core histone macro-H2A OS=Mus musculus OX=10090 GN=H2afy3 PE=2 SV=1	
Q3UYK8_MOUSE	Uncharacterized protein OS=Mus musculus OX=10090 GN=Rab3gap1 PE=2 SV=1	
tr|A2ALL9|A2ALL9_MOUSE Calcium-transporting ATPase OS=Mus musculus OX=10090 GN=Atp2b3...	Calcium-transporting ATPase OS=Mus musculus OX=10090 GN=Atp2b3 PE=1 SV=1	
E9QAQ7_MOUSE	AT-rich interactive domain-containing protein 1A OS=Mus musculus OX=10090 GN=Arid1a PE=1 SV=1	
ATLA3_MOUSE	Isoform 2 of Atlastin-3 OS=Mus musculus OX=10090 GN=Atl3	
tr|B2RRE0|B2RRE0_MOUSE-DECOY A kinase (PRKA) anchor protein (Gravin) 12 OS=Mus musculus...	tr|B2RRE0|B2RRE0_MOUSE-DECOY A kinase (PRKA) anchor protein (Gravin) 12 OS=Mus musculus...	
tr|Q6DFZ1|Q6DFZ1_MOUSE-DECOY Golgi-specific brefeldin A-resistance factor 1 OS=Mus...	tr|Q6DFZ1|Q6DFZ1_MOUSE-DECOY Golgi-specific brefeldin A-resistance factor 1 OS=Mus...	
B2RY30_MOUSE	Exosc9 protein OS=Mus musculus OX=10090 GN=Exosc9 PE=2 SV=1	
sp|Q8BGQ7|SYAC_MOUSE-DECOY Alanine--tRNA ligase, cytoplasmic OS=Mus musculus OX=10090...	sp|Q8BGQ7|SYAC_MOUSE-DECOY Alanine--tRNA ligase, cytoplasmic OS=Mus musculus OX=10090...	
tr|A0A140T8T2|A0A140T8T2_MOUSE-DECOY Slit homolog 2 protein OS=Mus musculus OX=10090...	tr|A0A140T8T2|A0A140T8T2_MOUSE-DECOY Slit homolog 2 protein OS=Mus musculus OX=10090...	
sp|Q9QY15-2|DDX25_MOUSE Isoform 2 of ATP-dependent RNA helicase DDX25 OS=Mus musculus...	sp|Q9QY15-2|DDX25_MOUSE Isoform 2 of ATP-dependent RNA helicase DDX25 OS=Mus musculus...	
sp|Q8BQM9-2|MD12L_MOUSE-DECOY Isoform 2 of Mediator of RNA polymerase II transcription...	sp|Q8BQM9-2|MD12L_MOUSE-DECOY Isoform 2 of Mediator of RNA polymerase II transcription...	
sp|Q80V86|INT8_MOUSE Integrator complex subunit 8 OS=Mus musculus OX=10090 GN=Ints8...	sp|Q80V86|INT8_MOUSE Integrator complex subunit 8 OS=Mus musculus OX=10090 GN=Ints8...	
tr|A0A140LIZ7|A0A140LIZ7_MOUSE-DECOY NHS-like protein 1 OS=Mus musculus OX=10090...	tr|A0A140LIZ7|A0A140LIZ7_MOUSE-DECOY NHS-like protein 1 OS=Mus musculus OX=10090...	
sp|Q9Z1K5|ARI1_MOUSE E3 ubiquitin-protein ligase ARIH1 OS=Mus musculus OX=10090 GN=Arih1...	sp|Q9Z1K5|ARI1_MOUSE E3 ubiquitin-protein ligase ARIH1 OS=Mus musculus OX=10090 GN=Arih1...	
tr|A0A087WPT7|A0A087WPT7_MOUSE Phosphatidylinositol 3,4,5-trisphosphate 5-phosphatase...	tr|A0A087WPT7|A0A087WPT7_MOUSE Phosphatidylinositol 3,4,5-trisphosphate 5-phosphatase...	
sp|Q8BYA0|TBCD_MOUSE Tubulin-specific chaperone D OS=Mus musculus OX=10090 GN=Tbcd...	Tubulin-specific chaperone D OS=Mus musculus OX=10090 GN=Tbcd PE=1 SV=1	
D3Z0Y2_MOUSE	Peroxiredoxin-6 OS=Mus musculus OX=10090 GN=Prdx6 PE=1 SV=1	
tr|Q3UH59|Q3UH59_MOUSE-DECOY Myosin-10 OS=Mus musculus OX=10090 GN=Myh10 PE=1 SV=1	tr|Q3UH59|Q3UH59_MOUSE-DECOY Myosin-10 OS=Mus musculus OX=10090 GN=Myh10 PE=1 SV=1	
PPCE_MOUSE	Prolyl endopeptidase OS=Mus musculus OX=10090 GN=Prep PE=1 SV=1	
sp|Q9D4H8-2|CUL2_MOUSE-DECOY Isoform 2 of Cullin-2 OS=Mus musculus OX=10090 GN=Cul2	sp|Q9D4H8-2|CUL2_MOUSE-DECOY Isoform 2 of Cullin-2 OS=Mus musculus OX=10090 GN=Cul2	
tr|Q3U048|Q3U048_MOUSE-DECOY Uncharacterized protein (Fragment) OS=Mus musculus OX=10090...	tr|Q3U048|Q3U048_MOUSE-DECOY Uncharacterized protein (Fragment) OS=Mus musculus OX=10090...	
Q8C1L7_MOUSE	40S ribosomal protein S21 OS=Mus musculus OX=10090 GN=mCG_6739 PE=2 SV=1	
sp|Q04859|MAK_MOUSE Serine/threonine-protein kinase MAK OS=Mus musculus OX=10090...	sp|Q04859|MAK_MOUSE Serine/threonine-protein kinase MAK OS=Mus musculus OX=10090...	
tr|Q3T9Y8|Q3T9Y8_MOUSE Eukaryotic translation initiation factor 3 subunit I OS=Mus...	tr|Q3T9Y8|Q3T9Y8_MOUSE Eukaryotic translation initiation factor 3 subunit I OS=Mus...	
tr|F6T9C3|F6T9C3_MOUSE Translation initiation factor eIF-2B subunit epsilon (Fragment)...	tr|F6T9C3|F6T9C3_MOUSE Translation initiation factor eIF-2B subunit epsilon (Fragment)...	
sp|Q924W5|SMC6_MOUSE Structural maintenance of chromosomes protein 6 OS=Mus musculus...	sp|Q924W5|SMC6_MOUSE Structural maintenance of chromosomes protein 6 OS=Mus musculus...	
Q3UGX2_MOUSE	Spectrin beta chain OS=Mus musculus OX=10090 GN=Sptb PE=1 SV=1	
A2A702_MOUSE	Eukaryotic translation initiation factor 3 subunit M OS=Mus musculus OX=10090 GN=Eif3m PE=1 SV=1	
PURA_MOUSE	Transcriptional activator protein Pur-alpha OS=Mus musculus OX=10090 GN=Pura PE=1 SV=1	
tr|E0CZ42|E0CZ42_MOUSE-DECOY Fer-1-like 6 (C. elegans) OS=Mus musculus OX=10090 GN=Fer1l6...	tr|E0CZ42|E0CZ42_MOUSE-DECOY Fer-1-like 6 (C. elegans) OS=Mus musculus OX=10090 GN=Fer1l6...	
B2RT32_MOUSE	Predicted gene, OTTMUSG00000010105 OS=Mus musculus OX=10090 GN=Gm13034 PE=2 SV=1	
sp|Q8BI72|CARF_MOUSE CDKN2A-interacting protein OS=Mus musculus OX=10090 GN=Cdkn2aip...	CDKN2A-interacting protein OS=Mus musculus OX=10090 GN=Cdkn2aip PE=1 SV=1	
sp|Q8CJ27|ASPM_MOUSE-DECOY Abnormal spindle-like microcephaly-associated protein...	sp|Q8CJ27|ASPM_MOUSE-DECOY Abnormal spindle-like microcephaly-associated protein...	
tr|Q3TWH2|Q3TWH2_MOUSE-DECOY Uncharacterized protein OS=Mus musculus OX=10090 GN=Nnt...	tr|Q3TWH2|Q3TWH2_MOUSE-DECOY Uncharacterized protein OS=Mus musculus OX=10090 GN=Nnt...	
tr|Q3UX80|Q3UX80_MOUSE Uncharacterized protein OS=Mus musculus OX=10090 GN=Snrpf...	tr|Q3UX80|Q3UX80_MOUSE Uncharacterized protein OS=Mus musculus OX=10090 GN=Snrpf...	
tr|B7ZCC3|B7ZCC3_MOUSE-DECOY Ataxin 7-like 2 OS=Mus musculus OX=10090 GN=Atxn7l2...	tr|B7ZCC3|B7ZCC3_MOUSE-DECOY Ataxin 7-like 2 OS=Mus musculus OX=10090 GN=Atxn7l2...	
A0A0B4J1L8_MOUSE	Immunoglobulin superfamily member 2 OS=Mus musculus OX=10090 GN=Cd101 PE=1 SV=1	
tr|Q3T9H2|Q3T9H2_MOUSE-DECOY Uncharacterized protein (Fragment) OS=Mus musculus OX=10090...	tr|Q3T9H2|Q3T9H2_MOUSE-DECOY Uncharacterized protein (Fragment) OS=Mus musculus OX=10090...	
sp|Q9WUU9|GANP_MOUSE-DECOY Germinal-center associated nuclear protein OS=Mus musculus...	sp|Q9WUU9|GANP_MOUSE-DECOY Germinal-center associated nuclear protein OS=Mus musculus...	
NKTR_MOUSE	NK-tumor recognition protein OS=Mus musculus OX=10090 GN=Nktr PE=1 SV=4	
tr|E9Q9A3|E9Q9A3_MOUSE-DECOY Clathrin coat assembly protein AP180 OS=Mus musculus...	tr|E9Q9A3|E9Q9A3_MOUSE-DECOY Clathrin coat assembly protein AP180 OS=Mus musculus...	
D3Z0B3_MOUSE	Acetyl-CoA carboxylase 2 (Fragment) OS=Mus musculus OX=10090 GN=Acacb PE=1 SV=1	
A0A0R4J0U8_MOUSE	RIKEN cDNA 1190005F20, isoform CRA_a OS=Mus musculus OX=10090 GN=Trmt1l PE=1 SV=1	
sp|Q9D2I5-5|ARMC9_MOUSE	Isoform 5 of LisH domain-containing protein ARMC9 OS=Mus musculus OX=10090 GN=Armc9	
ECHM_MOUSE	Enoyl-CoA hydratase, mitochondrial OS=Mus musculus OX=10090 GN=Echs1 PE=1 SV=1	
tr|A0A0G2JDZ9|A0A0G2JDZ9_MOUSE-DECOY Ankyrin repeat domain-containing protein 17...	tr|A0A0G2JDZ9|A0A0G2JDZ9_MOUSE-DECOY Ankyrin repeat domain-containing protein 17...	
Q3UIJ0_MOUSE	Uncharacterized protein OS=Mus musculus OX=10090 GN=Cct7 PE=2 SV=1	
D3Z131_MOUSE	THO complex subunit 6 homolog OS=Mus musculus OX=10090 GN=Thoc6 PE=1 SV=1	
tr|B4YB44|B4YB44_MOUSE Retrotransposon-like 1 OS=Mus musculus OX=10090 GN=Rtl1 PE=2...	tr|B4YB44|B4YB44_MOUSE Retrotransposon-like 1 OS=Mus musculus OX=10090 GN=Rtl1 PE=2...	
A0A140LJ59_MOUSE	Eukaryotic translation initiation factor 3 subunit K OS=Mus musculus OX=10090 GN=Eif3k PE=1 SV=1	
tr|A0A096P6K7|A0A096P6K7_MOUSE Transcription termination factor 1b, mitochondrial...	tr|A0A096P6K7|A0A096P6K7_MOUSE Transcription termination factor 1b, mitochondrial...	
tr|Q8K314|Q8K314_MOUSE Atp2b1 protein (Fragment) OS=Mus musculus OX=10090 GN=Atp2b1...	tr|Q8K314|Q8K314_MOUSE Atp2b1 protein (Fragment) OS=Mus musculus OX=10090 GN=Atp2b1...	
PTN11_MOUSE	Isoform 2 of Tyrosine-protein phosphatase non-receptor type 11 OS=Mus musculus OX=10090 GN=Ptpn11	
Q8BQD9_MOUSE	Uncharacterized protein OS=Mus musculus OX=10090 PE=2 SV=1	
tr|Q5SXR4|Q5SXR4_MOUSE Probable ATP-dependent RNA helicase DHX40 (Fragment) OS=Mus...	tr|Q5SXR4|Q5SXR4_MOUSE Probable ATP-dependent RNA helicase DHX40 (Fragment) OS=Mus...	
D3YZ98_MOUSE	Phosphatidylinositol 3-kinase catalytic subunit type 3 OS=Mus musculus OX=10090 GN=Pik3c3 PE=1 SV=1	
tr|Q05BH3|Q05BH3_MOUSE DEAH (Asp-Glu-Ala-His) box polypeptide 16 OS=Mus musculus...	tr|Q05BH3|Q05BH3_MOUSE DEAH (Asp-Glu-Ala-His) box polypeptide 16 OS=Mus musculus...	
sp|A6H603|NWD1_MOUSE-DECOY NACHT domain- and WD repeat-containing protein 1 OS=Mus...	sp|A6H603|NWD1_MOUSE-DECOY NACHT domain- and WD repeat-containing protein 1 OS=Mus...	
sp|O54990-2|PROM1_MOUSE-DECOY Isoform 2 of Prominin-1 OS=Mus musculus OX=10090 GN=Prom1	sp|O54990-2|PROM1_MOUSE-DECOY Isoform 2 of Prominin-1 OS=Mus musculus OX=10090 GN=Prom1	
SDHA_MOUSE	Succinate dehydrogenase [ubiquinone] flavoprotein subunit, mitochondrial OS=Mus musculus OX=10090 GN=Sdha PE=1 SV=1	
sp|Q08122-1|TLE3_MOUSE-DECOY Isoform 1 of Transducin-like enhancer protein 3 OS=Mus...	sp|Q08122-1|TLE3_MOUSE-DECOY Isoform 1 of Transducin-like enhancer protein 3 OS=Mus...	
Q9D050_MOUSE	Mitochondrial carrier homolog 2 OS=Mus musculus OX=10090 GN=Mtch2 PE=1 SV=1	
tr|D3Z7E6|D3Z7E6_MOUSE Platelet-activating factor acetylhydrolase IB subunit gamma...	tr|D3Z7E6|D3Z7E6_MOUSE Platelet-activating factor acetylhydrolase IB subunit gamma...	
IPO13_MOUSE	Isoform 2 of Importin-13 OS=Mus musculus OX=10090 GN=Ipo13	
sp|P11881-2|ITPR1_MOUSE-DECOY Isoform 2 of Inositol 1,4,5-trisphosphate receptor...	sp|P11881-2|ITPR1_MOUSE-DECOY Isoform 2 of Inositol 1,4,5-trisphosphate receptor...	
tr|F8VQ05|F8VQ05_MOUSE FRY-like transcription coactivator OS=Mus musculus OX=10090...	tr|F8VQ05|F8VQ05_MOUSE FRY-like transcription coactivator OS=Mus musculus OX=10090...	
sp|P35569|IRS1_MOUSE-DECOY Insulin receptor substrate 1 OS=Mus musculus OX=10090...	sp|P35569|IRS1_MOUSE-DECOY Insulin receptor substrate 1 OS=Mus musculus OX=10090...	
sp|Q8CH18-3|CCAR1_MOUSE-DECOY Isoform 3 of Cell division cycle and apoptosis regulator...	sp|Q8CH18-3|CCAR1_MOUSE-DECOY Isoform 3 of Cell division cycle and apoptosis regulator...	
Q9DBU8_MOUSE	Uncharacterized protein OS=Mus musculus OX=10090 GN=Ap3m1 PE=2 SV=1	
sp|Q99LI9|CLP1_MOUSE Polyribonucleotide 5'-hydroxyl-kinase Clp1 OS=Mus musculus OX=10090...	sp|Q99LI9|CLP1_MOUSE Polyribonucleotide 5'-hydroxyl-kinase Clp1 OS=Mus musculus OX=10090...	
tr|Q9CRE9|Q9CRE9_MOUSE Uncharacterized protein (Fragment) OS=Mus musculus OX=10090...	Uncharacterized protein (Fragment) OS=Mus musculus OX=10090 GN=Eif2s3x PE=2 SV=1	
tr|A0A087WPJ1|A0A087WPJ1_MOUSE-DECOY RIKEN cDNA 1700017N19 gene OS=Mus musculus OX=10090...	tr|A0A087WPJ1|A0A087WPJ1_MOUSE-DECOY RIKEN cDNA 1700017N19 gene OS=Mus musculus OX=10090...	
sp|O54692|ZW10_MOUSE Centromere/kinetochore protein zw10 homolog OS=Mus musculus...	sp|O54692|ZW10_MOUSE Centromere/kinetochore protein zw10 homolog OS=Mus musculus...	
NFH_MOUSE	Neurofilament heavy polypeptide OS=Mus musculus OX=10090 GN=Nefh PE=1 SV=3	
SAE1_MOUSE	SUMO-activating enzyme subunit 1 OS=Mus musculus OX=10090 GN=Sae1 PE=1 SV=1	
sp|Q6PHU5-2|SORT_MOUSE	Isoform 2 of Sortilin OS=Mus musculus OX=10090 GN=Sort1	
tr|E9PZP8|E9PZP8_MOUSE HECT and RLD domain-containing E3 ubiquitin protein ligase...	HECT and RLD domain-containing E3 ubiquitin protein ligase family member 1 OS=Mus musculus OX=10090 GN=Herc1 PE=1 SV=1	
sp|Q8CG48|SMC2_MOUSE-DECOY Structural maintenance of chromosomes protein 2 OS=Mus...	sp|Q8CG48|SMC2_MOUSE-DECOY Structural maintenance of chromosomes protein 2 OS=Mus...	
sp|Q7TMB8-2|CYFP1_MOUSE-DECOY Isoform 2 of Cytoplasmic FMR1-interacting protein 1...	sp|Q7TMB8-2|CYFP1_MOUSE-DECOY Isoform 2 of Cytoplasmic FMR1-interacting protein 1...	
tr|Q3TYX0|Q3TYX0_MOUSE-DECOY Uncharacterized protein OS=Mus musculus OX=10090 GN=St5...	tr|Q3TYX0|Q3TYX0_MOUSE-DECOY Uncharacterized protein OS=Mus musculus OX=10090 GN=St5...	
tr|Q8BR43|Q8BR43_MOUSE-DECOY Uncharacterized protein OS=Mus musculus OX=10090 GN=Jph3...	tr|Q8BR43|Q8BR43_MOUSE-DECOY Uncharacterized protein OS=Mus musculus OX=10090 GN=Jph3...	
tr|Q80TX5|Q80TX5_MOUSE MKIAA0572 protein (Fragment) OS=Mus musculus OX=10090 GN=Mcm3ap...	tr|Q80TX5|Q80TX5_MOUSE MKIAA0572 protein (Fragment) OS=Mus musculus OX=10090 GN=Mcm3ap...	
sp|Q8BJE2-2|BTNL9_MOUSE-DECOY Isoform 2 of Butyrophilin-like protein 9 OS=Mus musculus...	sp|Q8BJE2-2|BTNL9_MOUSE-DECOY Isoform 2 of Butyrophilin-like protein 9 OS=Mus musculus...	
sp|O88307|SORL_MOUSE-DECOY Sortilin-related receptor OS=Mus musculus OX=10090 GN=Sorl1...	sp|O88307|SORL_MOUSE-DECOY Sortilin-related receptor OS=Mus musculus OX=10090 GN=Sorl1...	
tr|E9Q7R9|E9Q7R9_MOUSE Cilia and flagella-associated protein 43 OS=Mus musculus OX=10090...	tr|E9Q7R9|E9Q7R9_MOUSE Cilia and flagella-associated protein 43 OS=Mus musculus OX=10090...	
tr|A0A1D5RMD1|A0A1D5RMD1_MOUSE-DECOY Predicted gene 16486 OS=Mus musculus OX=10090...	tr|A0A1D5RMD1|A0A1D5RMD1_MOUSE-DECOY Predicted gene 16486 OS=Mus musculus OX=10090...	
sp|P62274|RS29_MOUSE 40S ribosomal protein S29 OS=Mus musculus OX=10090 GN=Rps29...	sp|P62274|RS29_MOUSE 40S ribosomal protein S29 OS=Mus musculus OX=10090 GN=Rps29...	
sp|P16092-6|FGFR1_MOUSE	Isoform 6 of Fibroblast growth factor receptor 1 OS=Mus musculus OX=10090 GN=Fgfr1	
tr|A0A1B0GRL1|A0A1B0GRL1_MOUSE-DECOY Predicted gene 10153 OS=Mus musculus OX=10090...	tr|A0A1B0GRL1|A0A1B0GRL1_MOUSE-DECOY Predicted gene 10153 OS=Mus musculus OX=10090...	
B2RX77_MOUSE	MCG14870 OS=Mus musculus OX=10090 GN=Polr3c PE=2 SV=1	
TTK_MOUSE	Dual specificity protein kinase TTK OS=Mus musculus OX=10090 GN=Ttk PE=1 SV=1	
sp|P52480-2|KPYM_MOUSE-DECOY Isoform M1 of Pyruvate kinase PKM OS=Mus musculus OX=10090...	sp|P52480-2|KPYM_MOUSE-DECOY Isoform M1 of Pyruvate kinase PKM OS=Mus musculus OX=10090...	
tr|B2RY79|B2RY79_MOUSE-DECOY Dock9 protein OS=Mus musculus OX=10090 GN=Dock9 PE=2...	tr|B2RY79|B2RY79_MOUSE-DECOY Dock9 protein OS=Mus musculus OX=10090 GN=Dock9 PE=2...	
sp|Q8R5H1-2|UBP15_MOUSE-DECOY Isoform 2 of Ubiquitin carboxyl-terminal hydrolase...	sp|Q8R5H1-2|UBP15_MOUSE-DECOY Isoform 2 of Ubiquitin carboxyl-terminal hydrolase...	
sp|Q6QI06-2|RICTR_MOUSE-DECOY Isoform 2 of Rapamycin-insensitive companion of mTOR...	sp|Q6QI06-2|RICTR_MOUSE-DECOY Isoform 2 of Rapamycin-insensitive companion of mTOR...	
tr|A2AAU3|A2AAU3_MOUSE Probable helicase with zinc finger domain OS=Mus musculus...	tr|A2AAU3|A2AAU3_MOUSE Probable helicase with zinc finger domain OS=Mus musculus...	
tr|Q8R562|Q8R562_MOUSE-DECOY Ribonucleoprotein OS=Mus musculus OX=10090 GN=Trove2...	tr|Q8R562|Q8R562_MOUSE-DECOY Ribonucleoprotein OS=Mus musculus OX=10090 GN=Trove2...	
sp|Q8CC88|VWA8_MOUSE-DECOY von Willebrand factor A domain-containing protein 8 OS=Mus...	sp|Q8CC88|VWA8_MOUSE-DECOY von Willebrand factor A domain-containing protein 8 OS=Mus...	
sp|A2A8L1|CHD5_MOUSE-DECOY Chromodomain-helicase-DNA-binding protein 5 OS=Mus musculus...	sp|A2A8L1|CHD5_MOUSE-DECOY Chromodomain-helicase-DNA-binding protein 5 OS=Mus musculus...	
tr|Q3TZI5|Q3TZI5_MOUSE-DECOY DNA mismatch repair protein OS=Mus musculus OX=10090...	tr|Q3TZI5|Q3TZI5_MOUSE-DECOY DNA mismatch repair protein OS=Mus musculus OX=10090...	
tr|Q6PCP0|Q6PCP0_MOUSE-DECOY DEAD (Asp-Glu-Ala-Asp) box polypeptide 21 OS=Mus musculus...	tr|Q6PCP0|Q6PCP0_MOUSE-DECOY DEAD (Asp-Glu-Ala-Asp) box polypeptide 21 OS=Mus musculus...	
E9Q419_MOUSE	Beta-adrenergic receptor kinase 1 OS=Mus musculus OX=10090 GN=Grk2 PE=1 SV=1	
sp|Q8R5K2-2|UBP33_MOUSE-DECOY Isoform 2 of Ubiquitin carboxyl-terminal hydrolase...	sp|Q8R5K2-2|UBP33_MOUSE-DECOY Isoform 2 of Ubiquitin carboxyl-terminal hydrolase...	
tr|B2RQL1|B2RQL1_MOUSE-DECOY 4932413O14Rik protein OS=Mus musculus OX=10090 GN=Ccdc158...	tr|B2RQL1|B2RQL1_MOUSE-DECOY 4932413O14Rik protein OS=Mus musculus OX=10090 GN=Ccdc158...	
sp|Q9D4H4-2|AMOL1_MOUSE-DECOY Isoform 2 of Angiomotin-like protein 1 OS=Mus musculus...	sp|Q9D4H4-2|AMOL1_MOUSE-DECOY Isoform 2 of Angiomotin-like protein 1 OS=Mus musculus...	
tr|A0A1L1SQF0|A0A1L1SQF0_MOUSE Uncharacterized protein (Fragment) OS=Mus musculus...	Uncharacterized protein (Fragment) OS=Mus musculus OX=10090 PE=4 SV=1	
sp|E9Q6J5-3|BD1L1_MOUSE-DECOY Isoform New of Biorientation of chromosomes in cell...	sp|E9Q6J5-3|BD1L1_MOUSE-DECOY Isoform New of Biorientation of chromosomes in cell...	
sp|Q8CG72-2|ARHL2_MOUSE Isoform 2 of Poly(ADP-ribose) glycohydrolase ARH3 OS=Mus...	sp|Q8CG72-2|ARHL2_MOUSE Isoform 2 of Poly(ADP-ribose) glycohydrolase ARH3 OS=Mus...	
sp|Q9QYX7-2|PCLO_MOUSE-DECOY Isoform 2 of Protein piccolo OS=Mus musculus OX=10090...	sp|Q9QYX7-2|PCLO_MOUSE-DECOY Isoform 2 of Protein piccolo OS=Mus musculus OX=10090...	
sp|E9Q286|ICE1_MOUSE-DECOY Little elongation complex subunit 1 OS=Mus musculus OX=10090...	sp|E9Q286|ICE1_MOUSE-DECOY Little elongation complex subunit 1 OS=Mus musculus OX=10090...	
sp|Q9ESZ8-2|GTF2I_MOUSE-DECOY Isoform 2 of General transcription factor II-I OS=Mus...	sp|Q9ESZ8-2|GTF2I_MOUSE-DECOY Isoform 2 of General transcription factor II-I OS=Mus...	
sp|Q8CIN4|PAK2_MOUSE Serine/threonine-protein kinase PAK 2 OS=Mus musculus OX=10090...	sp|Q8CIN4|PAK2_MOUSE Serine/threonine-protein kinase PAK 2 OS=Mus musculus OX=10090...	
sp|A2A5R2|BIG2_MOUSE-DECOY Brefeldin A-inhibited guanine nucleotide-exchange protein...	sp|A2A5R2|BIG2_MOUSE-DECOY Brefeldin A-inhibited guanine nucleotide-exchange protein...	
Q8BKH5_MOUSE	RIKEN cDNA C330023M02, isoform CRA_b OS=Mus musculus OX=10090 GN=Naa25 PE=2 SV=1	
GSH1_MOUSE	Glutamate--cysteine ligase catalytic subunit OS=Mus musculus OX=10090 GN=Gclc PE=1 SV=4	
Q8BSN6_MOUSE	Vesicle-associated membrane protein 4 OS=Mus musculus OX=10090 GN=Vamp4 PE=1 SV=1	
sp|Q8CIE6|COPA_MOUSE Coatomer subunit alpha OS=Mus musculus OX=10090 GN=Copa PE=1...	sp|Q8CIE6|COPA_MOUSE Coatomer subunit alpha OS=Mus musculus OX=10090 GN=Copa PE=1...	
sp|P53349|M3K1_MOUSE Mitogen-activated protein kinase kinase kinase 1 OS=Mus musculus...	sp|P53349|M3K1_MOUSE Mitogen-activated protein kinase kinase kinase 1 OS=Mus musculus...	
sp|Q64735|CR1L_MOUSE	Complement component receptor 1-like protein OS=Mus musculus OX=10090 GN=Cr1l PE=1 SV=1	
sp|Q9QXS6-2|DREB_MOUSE-DECOY Isoform A2 of Drebrin OS=Mus musculus OX=10090 GN=Dbn1	sp|Q9QXS6-2|DREB_MOUSE-DECOY Isoform A2 of Drebrin OS=Mus musculus OX=10090 GN=Dbn1	
D3YX61_MOUSE	Rho-related GTP-binding protein RhoJ OS=Mus musculus OX=10090 GN=Rhoj PE=1 SV=1	
Q91YQ6_MOUSE	Acot5 protein OS=Mus musculus OX=10090 GN=Acot5 PE=2 SV=1	
Q9D3H4_MOUSE	Keratin-associated protein 2-4 OS=Mus musculus OX=10090 GN=Gm11938 PE=2 SV=1	
sp|Q8R326|PSPC1_MOUSEParaspeckle component 1 OS=Mus musculus OX=10090 GN=Pspc1 PE=1 SV=1	sp|Q8R326|PSPC1_MOUSE Paraspeckle component 1 OS=Mus musculus OX=10090 GN=Pspc1 PE=1 SV=1	
sp|Q7M6Z4-2|KIF27_MOUSE Isoform 2 of Kinesin-like protein KIF27 OS=Mus musculus OX=10090...	Isoform 2 of Kinesin-like protein KIF27 OS=Mus musculus OX=10090 GN=Kif27	
A0A0A6YW33_MOUSE	MCG18090 OS=Mus musculus OX=10090 GN=Gm6525 PE=4 SV=1	
K3W4M7_MOUSE	Putative ATP-dependent RNA helicase DHX33 (Fragment) OS=Mus musculus OX=10090 GN=Dhx33 PE=1 SV=1	
B2RQR1_MOUSE	Notch gene homolog 4 (Drosophila) OS=Mus musculus OX=10090 GN=Notch4 PE=2 SV=1	
A2AP88_MOUSE	Zinc finger CCCH domain-containing protein 6 OS=Mus musculus OX=10090 GN=Zc3h6 PE=1 SV=1	
Q3UGI2_MOUSE	Uncharacterized protein OS=Mus musculus OX=10090 GN=Gpatch2l PE=2 SV=1	
E9PV91_MOUSE	A disintegrin and metalloproteinase with thrombospondin motifs 4 OS=Mus musculus OX=10090 GN=Adamts4 PE=1 SV=1	
D3Z2F2_MOUSE	60 kDa heat shock protein, mitochondrial (Fragment) OS=Mus musculus OX=10090 GN=Hspd1 PE=1 SV=1	
tr|Q3TUG2|Q3TUG2_MOUSE DNA-directed RNA polymerase subunit (Fragment) OS=Mus musculus...	DNA-directed RNA polymerase subunit (Fragment) OS=Mus musculus OX=10090 GN=Polr1a PE=2 SV=1	
Q8CDE0_MOUSE	Uncharacterized protein OS=Mus musculus OX=10090 GN=Flot2 PE=2 SV=1	
Q8R268_MOUSE	Vomeronasal type-1 receptor OS=Mus musculus OX=10090 GN=Vmn1r212 PE=2 SV=1	
Q8BM06_MOUSE	Uncharacterized protein OS=Mus musculus OX=10090 GN=Nell2 PE=2 SV=1	
sp|E9Q8T7|DYH1_MOUSE-DECOY Dynein heavy chain 1, axonemal OS=Mus musculus OX=10090...	sp|E9Q8T7|DYH1_MOUSE-DECOY Dynein heavy chain 1, axonemal OS=Mus musculus OX=10090...	
DRG2_MOUSE	Developmentally-regulated GTP-binding protein 2 OS=Mus musculus OX=10090 GN=Drg2 PE=1 SV=1	
A0A286YE31_MOUSE	Ubiquitin-conjugating enzyme E2 E1 (Fragment) OS=Mus musculus OX=10090 GN=Ube2e1 PE=1 SV=1	
D6RG95_MOUSE	YTH domain-containing protein 1 OS=Mus musculus OX=10090 GN=Ythdc1 PE=1 SV=1	
B7ZNS2_MOUSE	Disks large-associated protein 4 OS=Mus musculus OX=10090 GN=Dlgap4 PE=1 SV=1	
tr|Q8BP21|Q8BP21_MOUSE Uncharacterized protein OS=Mus musculus OX=10090 GN=Cdk4 PE=2...	tr|Q8BP21|Q8BP21_MOUSE Uncharacterized protein OS=Mus musculus OX=10090 GN=Cdk4 PE=2...	
B2RWZ7_MOUSE	Phospholipase A2 OS=Mus musculus OX=10090 GN=Pla2g4f PE=2 SV=1	
sp|Q9QYF9|NDRG3_MOUSEProtein NDRG3 OS=Mus musculus OX=10090 GN=Ndrg3 PE=1 SV=1	sp|Q9QYF9|NDRG3_MOUSE Protein NDRG3 OS=Mus musculus OX=10090 GN=Ndrg3 PE=1 SV=1	
E9QK04_MOUSE	Neogenin OS=Mus musculus OX=10090 GN=Neo1 PE=1 SV=1	
Q3TYL7_MOUSE	DnaJ homolog subfamily B member 1 OS=Mus musculus OX=10090 GN=Dnajb1 PE=1 SV=1	
sp|Q80TL7-2|MON2_MOUSE	Isoform 2 of Protein MON2 homolog OS=Mus musculus OX=10090 GN=Mon2	
A0A0U1RP06_MOUSE	DNA repair protein-complementing XP-C cells homolog (Fragment) OS=Mus musculus OX=10090 GN=Xpc PE=1 SV=1	
B9EKC3_MOUSE	Rho GTPase activating protein 5 OS=Mus musculus OX=10090 GN=Arhgap5 PE=2 SV=1	
A0A1Y7VLS3_MOUSE	TOG array regulator of axonemal microtubules protein 1 OS=Mus musculus OX=10090 GN=Togaram1 PE=1 SV=1	
TWF1_MOUSE	Twinfilin-1 OS=Mus musculus OX=10090 GN=Twf1 PE=1 SV=2	
G3X8V0_MOUSE	Leucine-rich repeat-containing protein 63 OS=Mus musculus OX=10090 GN=Lrrc63 PE=4 SV=1	
tr|Q3V117|Q3V117_MOUSE ATP-citrate synthase OS=Mus musculus OX=10090 GN=Acly PE=1...	ATP-citrate synthase OS=Mus musculus OX=10090 GN=Acly PE=1 SV=1	
LSHR_MOUSE	Lutropin-choriogonadotropic hormone receptor OS=Mus musculus OX=10090 GN=Lhcgr PE=2 SV=1	
K0BWC3_MOUSE	Palladin isoform OS=Mus musculus OX=10090 GN=Palld PE=2 SV=1	
Q3U261_MOUSE	Uncharacterized protein (Fragment) OS=Mus musculus OX=10090 GN=Tacstd2 PE=2 SV=1	
BROMI_MOUSE	Isoform 2 of Protein broad-minded OS=Mus musculus OX=10090 GN=Tbc1d32	
Q3U2X1_MOUSE	Uncharacterized protein OS=Mus musculus OX=10090 GN=Nup62 PE=2 SV=1	
tr|E9Q6A7|E9Q6A7_MOUSE-DECOY Bromodomain PHD finger transcription factor OS=Mus musculus...	tr|E9Q6A7|E9Q6A7_MOUSE-DECOY Bromodomain PHD finger transcription factor OS=Mus musculus...	
E9Q3A0_MOUSE	E3 ubiquitin-protein ligase Trim36 OS=Mus musculus OX=10090 GN=Trim36 PE=1 SV=1	
D3Z4I0_MOUSE	Phosphatidylinositol glycan anchor biosynthesis, class G OS=Mus musculus OX=10090 GN=Pigg PE=1 SV=1	
sp|P08101-3|FCGR2_MOUSE	Isoform IIB1' of Low affinity immunoglobulin gamma Fc region receptor II OS=Mus musculus OX=10090 GN=Fcgr2	
sp|Q6PCM2-2|INT6_MOUSE	Isoform 2 of Integrator complex subunit 6 OS=Mus musculus OX=10090 GN=Ints6	
tr|A0A1Y7VM39|A0A1Y7VM39_MOUSE Extended synaptotagmin-2 OS=Mus musculus OX=10090...	tr|A0A1Y7VM39|A0A1Y7VM39_MOUSE Extended synaptotagmin-2 OS=Mus musculus OX=10090...	
sp|A2AFR3-2|FRPD4_MOUSE-DECOY Isoform 2 of FERM and PDZ domain-containing protein...	sp|A2AFR3-2|FRPD4_MOUSE-DECOY Isoform 2 of FERM and PDZ domain-containing protein...	
sp|Q8BGF7-4|PAN2_MOUSE	Isoform 4 of PAN2-PAN3 deadenylation complex catalytic subunit Pan2 OS=Mus musculus OX=10090 GN=Pan2	
D3YU89_MOUSE	Citron Rho-interacting kinase OS=Mus musculus OX=10090 GN=Cit PE=1 SV=1	
A0A023T778_MOUSE	Mago nashi protein OS=Mus musculus OX=10090 GN=Magohb PE=1 SV=1	
A0A1W2P7W3_MOUSE	Protein tyrosine phosphatase, receptor type, B, isoform CRA_b OS=Mus musculus OX=10090 GN=Ptprb PE=1 SV=1	
A0A2I3BRX9_MOUSE	Ral GTPase-activating protein subunit alpha-1 OS=Mus musculus OX=10090 GN=Ralgapa1 PE=1 SV=1	
sp|P53564-3|CUX1_MOUSE	Isoform 3 of Homeobox protein cut-like 1 OS=Mus musculus OX=10090 GN=Cux1	
ZO2_MOUSE	Tight junction protein ZO-2 OS=Mus musculus OX=10090 GN=Tjp2 PE=1 SV=2	
sp|Q9D4J7|PHF6_MOUSE	PHD finger protein 6 OS=Mus musculus OX=10090 GN=Phf6 PE=1 SV=1	
