# Supplementary material for: CPEB3 low-complexity motif regulates local protein synthesis via protein–protein interactions in neuronal ribonucleoprotein granules
Source: Proc Natl Acad Sci U S A. 2023 Jan 30;120(6):e2114747120. doi: 10.1073/pnas.2114747120 (PMC9964033; doi:10.1073/pnas.2114747120)
Supplement: Supplementary file 3 — Dataset S02 (RTF) [file pnas.2114747120.sd02.rtf]

Supplementary Data 2. Proteomics results, neurons.
ACTG_BOVIN	
Actin, cytoplasmic 2 OS=Bos taurus OX=9913 GN=ACTG1 PE=1 SV=1	
TBB4B_MOUSE	Tubulin beta-4B chain OS=Mus musculus OX=10090 GN=Tubb4b PE=1 SV=1	
SPTN1_MOUSE	Spectrin alpha chain, non-erythrocytic 1 OS=Mus musculus OX=10090 GN=Sptan1 PE=1 SV=4	
AT1A3_MOUSE	Sodium/potassium-transporting ATPase subunit alpha-3 OS=Mus musculus OX=10090 GN=Atp1a3 PE=1 SV=1	
SPTB2_MOUSE	Spectrin beta chain, non-erythrocytic 1 OS=Mus musculus OX=10090 GN=Sptbn1 PE=1 SV=2	
HS90B_MOUSE	Heat shock protein HSP 90-beta OS=Mus musculus OX=10090 GN=Hsp90ab1 PE=1 SV=3	
MYH10_MOUSE	Myosin-10 OS=Mus musculus OX=10090 GN=Myh10 PE=1 SV=2	
ATPB_MOUSE	ATP synthase subunit beta, mitochondrial OS=Mus musculus OX=10090 GN=Atp5f1b PE=1 SV=2	
HSP7C_MOUSE	Heat shock cognate 71 kDa protein OS=Mus musculus OX=10090 GN=Hspa8 PE=1 SV=1	
G3P_MOUSE	Glyceraldehyde-3-phosphate dehydrogenase OS=Mus musculus OX=10090 GN=Gapdh PE=1 SV=2	
EF1A1_MOUSE	Elongation factor 1-alpha 1 OS=Mus musculus OX=10090 GN=Eef1a1 PE=1 SV=3	
UBA1_MOUSE	Ubiquitin-like modifier-activating enzyme 1 OS=Mus musculus OX=10090 GN=Uba1 PE=1 SV=1	
MAP1B_MOUSE	Microtubule-associated protein 1B OS=Mus musculus OX=10090 GN=Map1b PE=1 SV=2	
TERA_HUMAN	Transitional endoplasmic reticulum ATPase OS=Homo sapiens OX=9606 GN=VCP PE=1 SV=4	
EF2_MOUSE	Elongation factor 2 OS=Mus musculus OX=10090 GN=Eef2 PE=1 SV=2	
ATPA_MOUSE	ATP synthase subunit alpha, mitochondrial OS=Mus musculus OX=10090 GN=Atp5f1a PE=1 SV=1	
FAS_MOUSE	Fatty acid synthase OS=Mus musculus OX=10090 GN=Fasn PE=1 SV=2	
CH60_MOUSE	60 kDa heat shock protein, mitochondrial OS=Mus musculus OX=10090 GN=Hspd1 PE=1 SV=1	
DPYL2_MOUSE	Dihydropyrimidinase-related protein 2 OS=Mus musculus OX=10090 GN=Dpysl2 PE=1 SV=2	
VIME_MOUSE	Vimentin OS=Mus musculus OX=10090 GN=Vim PE=1 SV=3	
MYH9_MOUSE	Myosin-9 OS=Mus musculus OX=10090 GN=Myh9 PE=1 SV=4	
KPYM_MOUSE	Pyruvate kinase PKM OS=Mus musculus OX=10090 GN=Pkm PE=1 SV=4	
MTAP2_MOUSE	Microtubule-associated protein 2 OS=Mus musculus OX=10090 GN=Map2 PE=1 SV=2	
KCRB_MOUSE	Creatine kinase B-type OS=Mus musculus OX=10090 GN=Ckb PE=1 SV=1	
ACON_MOUSE	Aconitate hydratase, mitochondrial OS=Mus musculus OX=10090 GN=Aco2 PE=1 SV=1	
PZP_MOUSE	Pregnancy zone protein OS=Mus musculus OX=10090 GN=Pzp PE=1 SV=3	
HS90A_MOUSE	Heat shock protein HSP 90-alpha OS=Mus musculus OX=10090 GN=Hsp90aa1 PE=1 SV=4	
DPYL1_MOUSE	Dihydropyrimidinase-related protein 1 OS=Mus musculus OX=10090 GN=Crmp1 PE=1 SV=1	
ENPL_MOUSE	Endoplasmin OS=Mus musculus OX=10090 GN=Hsp90b1 PE=1 SV=2	
GRP75_MOUSE	Stress-70 protein, mitochondrial OS=Mus musculus OX=10090 GN=Hspa9 PE=1 SV=3	
HSP74_MOUSE	Heat shock 70 kDa protein 4 OS=Mus musculus OX=10090 GN=Hspa4 PE=1 SV=1	
STXB1_MOUSE	Syntaxin-binding protein 1 OS=Mus musculus OX=10090 GN=Stxbp1 PE=1 SV=2	
NCAM1_MOUSE	Neural cell adhesion molecule 1 OS=Mus musculus OX=10090 GN=Ncam1 PE=1 SV=3	
FLNA_MOUSE	Filamin-A OS=Mus musculus OX=10090 GN=Flna PE=1 SV=5	
CO3_MOUSE	Complement C3 OS=Mus musculus OX=10090 GN=C3 PE=1 SV=3	
VATA_MOUSE	V-type proton ATPase catalytic subunit A OS=Mus musculus OX=10090 GN=Atp6v1a PE=1 SV=2	
GCAM_MOUSE	Ig gamma-2A chain C region, membrane-bound form OS=Mus musculus OX=10090 GN=Igh-1a PE=1 SV=3	
NSF_MOUSE	Vesicle-fusing ATPase OS=Mus musculus OX=10090 GN=Nsf PE=1 SV=2	
HXK1_MOUSE	Hexokinase-1 OS=Mus musculus OX=10090 GN=Hk1 PE=1 SV=3	
H2B1B_MOUSE	Histone H2B type 1-B OS=Mus musculus OX=10090 GN=Hist1h2bb PE=1 SV=3	
K2C1_HUMAN	Keratin, type II cytoskeletal 1 OS=Homo sapiens OX=9606 GN=KRT1 PE=1 SV=6	
DHX9_MOUSE	ATP-dependent RNA helicase A OS=Mus musculus OX=10090 GN=Dhx9 PE=1 SV=2	
ALDOA_MOUSE	Fructose-bisphosphate aldolase A OS=Mus musculus OX=10090 GN=Aldoa PE=1 SV=2	
AP2B1_MOUSE	AP-2 complex subunit beta OS=Mus musculus OX=10090 GN=Ap2b1 PE=1 SV=1	
MATR3_MOUSE	Matrin-3 OS=Mus musculus OX=10090 GN=Matr3 PE=1 SV=1	
HNRPU_MOUSE	Heterogeneous nuclear ribonucleoprotein U OS=Mus musculus OX=10090 GN=Hnrnpu PE=1 SV=1	
1433E_MOUSE	14-3-3 protein epsilon OS=Mus musculus OX=10090 GN=Ywhae PE=1 SV=1	
HNRPK_MOUSE	Heterogeneous nuclear ribonucleoprotein K OS=Mus musculus OX=10090 GN=Hnrnpk PE=1 SV=1	
BIP_MOUSE	Endoplasmic reticulum chaperone BiP OS=Mus musculus OX=10090 GN=Hspa5 PE=1 SV=3	
ENOA_MOUSE	Alpha-enolase OS=Mus musculus OX=10090 GN=Eno1 PE=1 SV=3	
DPYL5_MOUSE	Dihydropyrimidinase-related protein 5 OS=Mus musculus OX=10090 GN=Dpysl5 PE=1 SV=1	
CALX_MOUSE	Calnexin OS=Mus musculus OX=10090 GN=Canx PE=1 SV=1	
2AAA_MOUSE	Serine/threonine-protein phosphatase 2A 65 kDa regulatory subunit A alpha isoform OS=Mus musculus OX=10090 GN=Ppp2r1a PE=1 SV=3	
SCOT1_MOUSE	Succinyl-CoA:3-ketoacid coenzyme A transferase 1, mitochondrial OS=Mus musculus OX=10090 GN=Oxct1 PE=1 SV=1	
AP2A1_MOUSE	AP-2 complex subunit alpha-1 OS=Mus musculus OX=10090 GN=Ap2a1 PE=1 SV=1	
1433Z_MOUSE	14-3-3 protein zeta/delta OS=Mus musculus OX=10090 GN=Ywhaz PE=1 SV=1	
LRP1_MOUSE	Prolow-density lipoprotein receptor-related protein 1 OS=Mus musculus OX=10090 GN=Lrp1 PE=1 SV=1	
GDIB_MOUSE	Rab GDP dissociation inhibitor beta OS=Mus musculus OX=10090 GN=Gdi2 PE=1 SV=1	
PGK1_MOUSE	Phosphoglycerate kinase 1 OS=Mus musculus OX=10090 GN=Pgk1 PE=1 SV=4	
ACLY_MOUSE	ATP-citrate synthase OS=Mus musculus OX=10090 GN=Acly PE=1 SV=1	
DPYL3_MOUSE	Dihydropyrimidinase-related protein 3 OS=Mus musculus OX=10090 GN=Dpysl3 PE=1 SV=1	
HNRPL_MOUSE	Heterogeneous nuclear ribonucleoprotein L OS=Mus musculus OX=10090 GN=Hnrnpl PE=1 SV=2	
PDIA3_MOUSE	Protein disulfide-isomerase A3 OS=Mus musculus OX=10090 GN=Pdia3 PE=1 SV=2	
ADT1_MOUSE	ADP/ATP translocase 1 OS=Mus musculus OX=10090 GN=Slc25a4 PE=1 SV=4	
LPPRC_MOUSE	Leucine-rich PPR motif-containing protein, mitochondrial OS=Mus musculus OX=10090 GN=Lrpprc PE=1 SV=2	
MIC60_MOUSE	MICOS complex subunit Mic60 OS=Mus musculus OX=10090 GN=Immt PE=1 SV=1	
TCPB_MOUSE	T-complex protein 1 subunit beta OS=Mus musculus OX=10090 GN=Cct2 PE=1 SV=4	
TCPQ_MOUSE	T-complex protein 1 subunit theta OS=Mus musculus OX=10090 GN=Cct8 PE=1 SV=3	
TKT_MOUSE	Transketolase OS=Mus musculus OX=10090 GN=Tkt PE=1 SV=1	
KCC2B_MOUSE	Calcium/calmodulin-dependent protein kinase type II subunit beta OS=Mus musculus OX=10090 GN=Camk2b PE=1 SV=2	
GNAO_MOUSE	Guanine nucleotide-binding protein G(o) subunit alpha OS=Mus musculus OX=10090 GN=Gnao1 PE=1 SV=3	
AL1L1_MOUSE	Cytosolic 10-formyltetrahydrofolate dehydrogenase OS=Mus musculus OX=10090 GN=Aldh1l1 PE=1 SV=1	
AT1A1_MOUSE	Sodium/potassium-transporting ATPase subunit alpha-1 OS=Mus musculus OX=10090 GN=Atp1a1 PE=1 SV=1	
AT2B1_MOUSE	Plasma membrane calcium-transporting ATPase 1 OS=Mus musculus OX=10090 GN=Atp2b1 PE=1 SV=1	
DHE3_MOUSE	Glutamate dehydrogenase 1, mitochondrial OS=Mus musculus OX=10090 GN=Glud1 PE=1 SV=1	
DNM1L_MOUSE	Dynamin-1-like protein OS=Mus musculus OX=10090 GN=Dnm1l PE=1 SV=2	
GABT_MOUSE	4-aminobutyrate aminotransferase, mitochondrial OS=Mus musculus OX=10090 GN=Abat PE=1 SV=1	
LMNB1_MOUSE	Lamin-B1 OS=Mus musculus OX=10090 GN=Lmnb1 PE=1 SV=3	
VATB2_MOUSE	V-type proton ATPase subunit B, brain isoform OS=Mus musculus OX=10090 GN=Atp6v1b2 PE=1 SV=1	
AATM_MOUSE	Aspartate aminotransferase, mitochondrial OS=Mus musculus OX=10090 GN=Got2 PE=1 SV=1	
CON__P13645	CON__P13645	
MAP1A_MOUSE	Microtubule-associated protein 1A OS=Mus musculus OX=10090 GN=Map1a PE=1 SV=2	
MDHM_MOUSE	Malate dehydrogenase, mitochondrial OS=Mus musculus OX=10090 GN=Mdh2 PE=1 SV=3	
FSCN1_MOUSE	Fascin OS=Mus musculus OX=10090 GN=Fscn1 PE=1 SV=4	
MARCS_MOUSE	Myristoylated alanine-rich C-kinase substrate OS=Mus musculus OX=10090 GN=Marcks PE=1 SV=2	
PPIA_MOUSE	Peptidyl-prolyl cis-trans isomerase A OS=Mus musculus OX=10090 GN=Ppia PE=1 SV=2	
ROA2_MOUSE	Heterogeneous nuclear ribonucleoproteins A2/B1 OS=Mus musculus OX=10090 GN=Hnrnpa2b1 PE=1 SV=2	
SFPQ_MOUSE	Splicing factor, proline- and glutamine-rich OS=Mus musculus OX=10090 GN=Sfpq PE=1 SV=1	
VPP1_MOUSE	V-type proton ATPase 116 kDa subunit a isoform 1 OS=Mus musculus OX=10090 GN=Atp6v0a1 PE=1 SV=3	
HNRPM_MOUSE	Heterogeneous nuclear ribonucleoprotein M OS=Mus musculus OX=10090 GN=Hnrnpm PE=1 SV=3	
SYN1_MOUSE	Synapsin-1 OS=Mus musculus OX=10090 GN=Syn1 PE=1 SV=2	
TBB2A_MOUSE	Tubulin beta-2A chain OS=Mus musculus OX=10090 GN=Tubb2a PE=1 SV=1	
UBP5_MOUSE	Ubiquitin carboxyl-terminal hydrolase 5 OS=Mus musculus OX=10090 GN=Usp5 PE=1 SV=1	
VPS35_MOUSE	Vacuolar protein sorting-associated protein 35 OS=Mus musculus OX=10090 GN=Vps35 PE=1 SV=1	
GDIA_MOUSE	Rab GDP dissociation inhibitor alpha OS=Mus musculus OX=10090 GN=Gdi1 PE=1 SV=3	
TBB3_MOUSE	Tubulin beta-3 chain OS=Mus musculus OX=10090 GN=Tubb3 PE=1 SV=1	
VDAC1_MOUSE	Voltage-dependent anion-selective channel protein 1 OS=Mus musculus OX=10090 GN=Vdac1 PE=1 SV=3	
MYO5A_MOUSE	Unconventional myosin-Va OS=Mus musculus OX=10090 GN=Myo5a PE=1 SV=2	
ODO1_MOUSE	2-oxoglutarate dehydrogenase, mitochondrial OS=Mus musculus OX=10090 GN=Ogdh PE=1 SV=3	
HNRH1_MOUSE	Heterogeneous nuclear ribonucleoprotein H OS=Mus musculus OX=10090 GN=Hnrnph1 PE=1 SV=3	
IF4A1_MOUSE	Eukaryotic initiation factor 4A-I OS=Mus musculus OX=10090 GN=Eif4a1 PE=1 SV=1	
TCPA_MOUSE	T-complex protein 1 subunit alpha OS=Mus musculus OX=10090 GN=Tcp1 PE=1 SV=3	
ACTN4_MOUSE	Alpha-actinin-4 OS=Mus musculus OX=10090 GN=Actn4 PE=1 SV=1	
CMC1_MOUSE	Calcium-binding mitochondrial carrier protein Aralar1 OS=Mus musculus OX=10090 GN=Slc25a12 PE=1 SV=1	
ROA1_BOVIN	Heterogeneous nuclear ribonucleoprotein A1 OS=Bos taurus OX=9913 GN=HNRNPA1 PE=1 SV=2	
CALR_MOUSE	Calreticulin OS=Mus musculus OX=10090 GN=Calr PE=1 SV=1	
COF1_MOUSE	Cofilin-1 OS=Mus musculus OX=10090 GN=Cfl1 PE=1 SV=3	
H4_HUMAN	Histone H4 OS=Homo sapiens OX=9606 GN=H4C1 PE=1 SV=2	
LDHB_MOUSE	L-lactate dehydrogenase B chain OS=Mus musculus OX=10090 GN=Ldhb PE=1 SV=2	
SYAC_MOUSE	Alanine--tRNA ligase, cytoplasmic OS=Mus musculus OX=10090 GN=Aars PE=1 SV=1	
MACF1_MOUSE	Microtubule-actin cross-linking factor 1 OS=Mus musculus OX=10090 GN=Macf1 PE=1 SV=2	
MPCP_MOUSE	Phosphate carrier protein, mitochondrial OS=Mus musculus OX=10090 GN=Slc25a3 PE=1 SV=1	
NUCL_MOUSE	Nucleolin OS=Mus musculus OX=10090 GN=Ncl PE=1 SV=2	
QCR2_MOUSE	Cytochrome b-c1 complex subunit 2, mitochondrial OS=Mus musculus OX=10090 GN=Uqcrc2 PE=1 SV=1	
RTN4_MOUSE	Reticulon-4 OS=Mus musculus OX=10090 GN=Rtn4 PE=1 SV=2	
TOP2B_MOUSE	DNA topoisomerase 2-beta OS=Mus musculus OX=10090 GN=Top2b PE=1 SV=2	
DREB_MOUSE	Drebrin OS=Mus musculus OX=10090 GN=Dbn1 PE=1 SV=4	
ANXA6_MOUSE	Annexin A6 OS=Mus musculus OX=10090 GN=Anxa6 PE=1 SV=3	
IMB1_MOUSE	Importin subunit beta-1 OS=Mus musculus OX=10090 GN=Kpnb1 PE=1 SV=2	
RPN2_MOUSE	Dolichyl-diphosphooligosaccharide--protein glycosyltransferase subunit 2 OS=Mus musculus OX=10090 GN=Rpn2 PE=1 SV=1	
CISY_MOUSE	Citrate synthase, mitochondrial OS=Mus musculus OX=10090 GN=Cs PE=1 SV=1	
G6PI_MOUSE	Glucose-6-phosphate isomerase OS=Mus musculus OX=10090 GN=Gpi PE=1 SV=4	
GUAD_MOUSE	Guanine deaminase OS=Mus musculus OX=10090 GN=Gda PE=1 SV=1	
MAP6_MOUSE	Microtubule-associated protein 6 OS=Mus musculus OX=10090 GN=Map6 PE=1 SV=2	
QCR1_MOUSE	Cytochrome b-c1 complex subunit 1, mitochondrial OS=Mus musculus OX=10090 GN=Uqcrc1 PE=1 SV=2	
TCPD_MOUSE	T-complex protein 1 subunit delta OS=Mus musculus OX=10090 GN=Cct4 PE=1 SV=3	
CAND1_MOUSE	Cullin-associated NEDD8-dissociated protein 1 OS=Mus musculus OX=10090 GN=Cand1 PE=1 SV=2	
DDX3X_MOUSE	ATP-dependent RNA helicase DDX3X OS=Mus musculus OX=10090 GN=Ddx3x PE=1 SV=3	
GBB1_MOUSE	Guanine nucleotide-binding protein G(I)/G(S)/G(T) subunit beta-1 OS=Mus musculus OX=10090 GN=Gnb1 PE=1 SV=3	
NDUS1_MOUSE	NADH-ubiquinone oxidoreductase 75 kDa subunit, mitochondrial OS=Mus musculus OX=10090 GN=Ndufs1 PE=1 SV=2	
PYC_MOUSE	Pyruvate carboxylase, mitochondrial OS=Mus musculus OX=10090 GN=Pc PE=1 SV=1	
SYT1_MOUSE	Synaptotagmin-1 OS=Mus musculus OX=10090 GN=Syt1 PE=1 SV=1	
TCPG_MOUSE	T-complex protein 1 subunit gamma OS=Mus musculus OX=10090 GN=Cct3 PE=1 SV=1	
ALBU_MOUSE	Serum albumin OS=Mus musculus OX=10090 GN=Alb PE=1 SV=3	
CALM1_MOUSE	Calmodulin-1 OS=Mus musculus OX=10090 GN=Calm1 PE=1 SV=1	
KAP3_MOUSE	cAMP-dependent protein kinase type II-beta regulatory subunit OS=Mus musculus OX=10090 GN=Prkar2b PE=1 SV=3	
CAP1_MOUSE	Adenylyl cyclase-associated protein 1 OS=Mus musculus OX=10090 GN=Cap1 PE=1 SV=4	
SDHA_MOUSE	Succinate dehydrogenase [ubiquinone] flavoprotein subunit, mitochondrial OS=Mus musculus OX=10090 GN=Sdha PE=1 SV=1	
DYN1_MOUSE	Dynamin-1 OS=Mus musculus OX=10090 GN=Dnm1 PE=1 SV=2	
PRP8_MOUSE	Pre-mRNA-processing-splicing factor 8 OS=Mus musculus OX=10090 GN=Prpf8 PE=1 SV=2	
STIP1_MOUSE	Stress-induced-phosphoprotein 1 OS=Mus musculus OX=10090 GN=Stip1 PE=1 SV=1	
RPN1_MOUSE	Dolichyl-diphosphooligosaccharide--protein glycosyltransferase subunit 1 OS=Mus musculus OX=10090 GN=Rpn1 PE=1 SV=1	
PGAM1_MOUSE	Phosphoglycerate mutase 1 OS=Mus musculus OX=10090 GN=Pgam1 PE=1 SV=3	
PSA_MOUSE	Puromycin-sensitive aminopeptidase OS=Mus musculus OX=10090 GN=Npepps PE=1 SV=2	
PSMD2_MOUSE	26S proteasome non-ATPase regulatory subunit 2 OS=Mus musculus OX=10090 GN=Psmd2 PE=1 SV=1	
TCPE_MOUSE	T-complex protein 1 subunit epsilon OS=Mus musculus OX=10090 GN=Cct5 PE=1 SV=1	
WDR1_MOUSE	WD repeat-containing protein 1 OS=Mus musculus OX=10090 GN=Wdr1 PE=1 SV=3	
CON__P35908	CON__P35908	
TBB5_MOUSE	Tubulin beta-5 chain OS=Mus musculus OX=10090 GN=Tubb5 PE=1 SV=1	
CAPS1_MOUSE	Calcium-dependent secretion activator 1 OS=Mus musculus OX=10090 GN=Cadps PE=1 SV=3	
GANAB_MOUSE	Neutral alpha-glucosidase AB OS=Mus musculus OX=10090 GN=Ganab PE=1 SV=1	
IDHC_MOUSE	Isocitrate dehydrogenase [NADP] cytoplasmic OS=Mus musculus OX=10090 GN=Idh1 PE=1 SV=2	
KIF5C_MOUSE	Kinesin heavy chain isoform 5C OS=Mus musculus OX=10090 GN=Kif5c PE=1 SV=3	
THIL_MOUSE	Acetyl-CoA acetyltransferase, mitochondrial OS=Mus musculus OX=10090 GN=Acat1 PE=1 SV=1	
SYEP_MOUSE	Bifunctional glutamate/proline--tRNA ligase OS=Mus musculus OX=10090 GN=Eprs PE=1 SV=4	
IGG2B_MOUSE	Ig gamma-2B chain C region OS=Mus musculus OX=10090 GN=Igh-3 PE=1 SV=3	
ARP3_MOUSE	Actin-related protein 3 OS=Mus musculus OX=10090 GN=Actr3 PE=1 SV=3	
HYOU1_MOUSE	Hypoxia up-regulated protein 1 OS=Mus musculus OX=10090 GN=Hyou1 PE=1 SV=1	
XPO2_MOUSE	Exportin-2 OS=Mus musculus OX=10090 GN=Cse1l PE=1 SV=1	
CYFP2_MOUSE	Cytoplasmic FMR1-interacting protein 2 OS=Mus musculus OX=10090 GN=Cyfip2 PE=1 SV=2	
EF1G_MOUSE	Elongation factor 1-gamma OS=Mus musculus OX=10090 GN=Eef1g PE=1 SV=3	
FLNB_MOUSE	Filamin-B OS=Mus musculus OX=10090 GN=Flnb PE=1 SV=3	
H2A1C_HUMAN	Histone H2A type 1-C OS=Homo sapiens OX=9606 GN=HIST1H2AC PE=1 SV=3	
CATA_MOUSE	Catalase OS=Mus musculus OX=10090 GN=Cat PE=1 SV=4	
GPDM_MOUSE	Glycerol-3-phosphate dehydrogenase, mitochondrial OS=Mus musculus OX=10090 GN=Gpd2 PE=1 SV=2	
NCDN_MOUSE	Neurochondrin OS=Mus musculus OX=10090 GN=Ncdn PE=1 SV=1	
ROA3_MOUSE	Heterogeneous nuclear ribonucleoprotein A3 OS=Mus musculus OX=10090 GN=Hnrnpa3 PE=1 SV=1	
USP9X_MOUSE	Probable ubiquitin carboxyl-terminal hydrolase FAF-X OS=Mus musculus OX=10090 GN=Usp9x PE=1 SV=2	
DDX5_MOUSE	Probable ATP-dependent RNA helicase DDX5 OS=Mus musculus OX=10090 GN=Ddx5 PE=1 SV=2	
PDIA1_MOUSE	Protein disulfide-isomerase OS=Mus musculus OX=10090 GN=P4hb PE=1 SV=2	
PHB2_MOUSE	Prohibitin-2 OS=Mus musculus OX=10090 GN=Phb2 PE=1 SV=1	
SYYC_MOUSE	Tyrosine--tRNA ligase, cytoplasmic OS=Mus musculus OX=10090 GN=Yars PE=1 SV=3	
ECHA_MOUSE	Trifunctional enzyme subunit alpha, mitochondrial OS=Mus musculus OX=10090 GN=Hadha PE=1 SV=1	
MOES_MOUSE	Moesin OS=Mus musculus OX=10090 GN=Msn PE=1 SV=3	
RACK1_MOUSE	Receptor of activated protein C kinase 1 OS=Mus musculus OX=10090 GN=Rack1 PE=1 SV=3	
1433G_MOUSE	14-3-3 protein gamma OS=Mus musculus OX=10090 GN=Ywhag PE=1 SV=2	
AP2A2_MOUSE	AP-2 complex subunit alpha-2 OS=Mus musculus OX=10090 GN=Ap2a2 PE=1 SV=2	
DX39B_MOUSE	Spliceosome RNA helicase Ddx39b OS=Mus musculus OX=10090 GN=Ddx39b PE=1 SV=1	
PSMD1_MOUSE	26S proteasome non-ATPase regulatory subunit 1 OS=Mus musculus OX=10090 GN=Psmd1 PE=1 SV=1	
TCPH_MOUSE	T-complex protein 1 subunit eta OS=Mus musculus OX=10090 GN=Cct7 PE=1 SV=1	
AT2A2_MOUSE	Sarcoplasmic/endoplasmic reticulum calcium ATPase 2 OS=Mus musculus OX=10090 GN=Atp2a2 PE=1 SV=2	
PYGB_MOUSE	Glycogen phosphorylase, brain form OS=Mus musculus OX=10090 GN=Pygb PE=1 SV=3	
TCPZ_MOUSE	T-complex protein 1 subunit zeta OS=Mus musculus OX=10090 GN=Cct6a PE=1 SV=3	
IDHP_MOUSE	Isocitrate dehydrogenase [NADP], mitochondrial OS=Mus musculus OX=10090 GN=Idh2 PE=1 SV=3	
PP2AA_MOUSE	Serine/threonine-protein phosphatase 2A catalytic subunit alpha isoform OS=Mus musculus OX=10090 GN=Ppp2ca PE=1 SV=1	
ODPB_MOUSE	Pyruvate dehydrogenase E1 component subunit beta, mitochondrial OS=Mus musculus OX=10090 GN=Pdhb PE=1 SV=1	
CNTN1_MOUSE	Contactin-1 OS=Mus musculus OX=10090 GN=Cntn1 PE=1 SV=1	
PABP1_MOUSE	Polyadenylate-binding protein 1 OS=Mus musculus OX=10090 GN=Pabpc1 PE=1 SV=2	
RS4X_MOUSE	40S ribosomal protein S4, X isoform OS=Mus musculus OX=10090 GN=Rps4x PE=1 SV=2	
ANXA2_MOUSE	Annexin A2 OS=Mus musculus OX=10090 GN=Anxa2 PE=1 SV=2	
CKAP4_MOUSE	Cytoskeleton-associated protein 4 OS=Mus musculus OX=10090 GN=Ckap4 PE=1 SV=2	
ANK2_MOUSE	Ankyrin-2 OS=Mus musculus OX=10090 GN=Ank2 PE=1 SV=2	
AT1B1_MOUSE	Sodium/potassium-transporting ATPase subunit beta-1 OS=Mus musculus OX=10090 GN=Atp1b1 PE=1 SV=1	
PFKAM_MOUSE	ATP-dependent 6-phosphofructokinase, muscle type OS=Mus musculus OX=10090 GN=Pfkm PE=1 SV=3	
HS105_MOUSE	Heat shock protein 105 kDa OS=Mus musculus OX=10090 GN=Hsph1 PE=1 SV=2	
LDHA_MOUSE	L-lactate dehydrogenase A chain OS=Mus musculus OX=10090 GN=Ldha PE=1 SV=3	
4F2_MOUSE	4F2 cell-surface antigen heavy chain OS=Mus musculus OX=10090 GN=Slc3a2 PE=1 SV=1	
RS3_MOUSE	40S ribosomal protein S3 OS=Mus musculus OX=10090 GN=Rps3 PE=1 SV=1	
ANXA5_MOUSE	Annexin A5 OS=Mus musculus OX=10090 GN=Anxa5 PE=1 SV=1	
AP2M1_MOUSE	AP-2 complex subunit mu OS=Mus musculus OX=10090 GN=Ap2m1 PE=1 SV=1	
C1QA_MOUSE	Complement C1q subcomponent subunit A OS=Mus musculus OX=10090 GN=C1qa PE=1 SV=2	
DCTN1_MOUSE	Dynactin subunit 1 OS=Mus musculus OX=10090 GN=Dctn1 PE=1 SV=3	
FKBP4_MOUSE	Peptidyl-prolyl cis-trans isomerase FKBP4 OS=Mus musculus OX=10090 GN=Fkbp4 PE=1 SV=5	
GARS_MOUSE	Glycine--tRNA ligase OS=Mus musculus OX=10090 GN=Gars PE=1 SV=1	
RL4_MOUSE	60S ribosomal protein L4 OS=Mus musculus OX=10090 GN=Rpl4 PE=1 SV=3	
ODPA_MOUSE	Pyruvate dehydrogenase E1 component subunit alpha, somatic form, mitochondrial OS=Mus musculus OX=10090 GN=Pdha1 PE=1 SV=1	
SUCB1_MOUSE	Succinate--CoA ligase [ADP-forming] subunit beta, mitochondrial OS=Mus musculus OX=10090 GN=Sucla2 PE=1 SV=2	
U520_MOUSE	U5 small nuclear ribonucleoprotein 200 kDa helicase OS=Mus musculus OX=10090 GN=Snrnp200 PE=1 SV=1	
ACTA_MOUSE	Actin, aortic smooth muscle OS=Mus musculus OX=10090 GN=Acta2 PE=1 SV=1	
SERPH_MOUSE	Serpin H1 OS=Mus musculus OX=10090 GN=Serpinh1 PE=1 SV=3	
APOE_MOUSE	Apolipoprotein E OS=Mus musculus OX=10090 GN=Apoe PE=1 SV=2	
C1QB_MOUSE	Complement C1q subcomponent subunit B OS=Mus musculus OX=10090 GN=C1qb PE=1 SV=2	
PCBP2_MOUSE	Poly(rC)-binding protein 2 OS=Mus musculus OX=10090 GN=Pcbp2 PE=1 SV=1	
PRDX6_MOUSE	Peroxiredoxin-6 OS=Mus musculus OX=10090 GN=Prdx6 PE=1 SV=3	
UCHL1_MOUSE	Ubiquitin carboxyl-terminal hydrolase isozyme L1 OS=Mus musculus OX=10090 GN=Uchl1 PE=1 SV=1	
XPO1_MOUSE	Exportin-1 OS=Mus musculus OX=10090 GN=Xpo1 PE=1 SV=1	
BASP1_MOUSE	Brain acid soluble protein 1 OS=Mus musculus OX=10090 GN=Basp1 PE=1 SV=3	
DHB4_MOUSE	Peroxisomal multifunctional enzyme type 2 OS=Mus musculus OX=10090 GN=Hsd17b4 PE=1 SV=3	
PLCB1_MOUSE	1-phosphatidylinositol 4,5-bisphosphate phosphodiesterase beta-1 OS=Mus musculus OX=10090 GN=Plcb1 PE=1 SV=2	
AP180_MOUSE	Clathrin coat assembly protein AP180 OS=Mus musculus OX=10090 GN=Snap91 PE=1 SV=1	
EIF3A_MOUSE	Eukaryotic translation initiation factor 3 subunit A OS=Mus musculus OX=10090 GN=Eif3a PE=1 SV=5	
MDHC_MOUSE	Malate dehydrogenase, cytoplasmic OS=Mus musculus OX=10090 GN=Mdh1 PE=1 SV=3	
FABP7_MOUSE	Fatty acid-binding protein, brain OS=Mus musculus OX=10090 GN=Fabp7 PE=1 SV=2	
GNAI2_MOUSE	Guanine nucleotide-binding protein G(i) subunit alpha-2 OS=Mus musculus OX=10090 GN=Gnai2 PE=1 SV=5	
GSTM1_MOUSE	Glutathione S-transferase Mu 1 OS=Mus musculus OX=10090 GN=Gstm1 PE=1 SV=2	
PHB_MOUSE	Prohibitin OS=Mus musculus OX=10090 GN=Phb PE=1 SV=1	
AKA12_MOUSE	A-kinase anchor protein 12 OS=Mus musculus OX=10090 GN=Akap12 PE=1 SV=1	
DCLK1_MOUSE	Serine/threonine-protein kinase DCLK1 OS=Mus musculus OX=10090 GN=Dclk1 PE=1 SV=1	
DDX1_MOUSE	ATP-dependent RNA helicase DDX1 OS=Mus musculus OX=10090 GN=Ddx1 PE=1 SV=1	
EFTU_MOUSE	Elongation factor Tu, mitochondrial OS=Mus musculus OX=10090 GN=Tufm PE=1 SV=1	
PGBM_MOUSE	Basement membrane-specific heparan sulfate proteoglycan core protein OS=Mus musculus OX=10090 GN=Hspg2 PE=1 SV=1	
TAU_MOUSE	Microtubule-associated protein tau OS=Mus musculus OX=10090 GN=Mapt PE=1 SV=3	
DLDH_MOUSE	Dihydrolipoyl dehydrogenase, mitochondrial OS=Mus musculus OX=10090 GN=Dld PE=1 SV=2	
HNRL2_MOUSE	Heterogeneous nuclear ribonucleoprotein U-like protein 2 OS=Mus musculus OX=10090 GN=Hnrnpul2 PE=1 SV=2	
NONO_MOUSE	Non-POU domain-containing octamer-binding protein OS=Mus musculus OX=10090 GN=Nono PE=1 SV=3	
ODP2_MOUSE	Dihydrolipoyllysine-residue acetyltransferase component of pyruvate dehydrogenase complex, mitochondrial OS=Mus musculus OX=10090 GN=Dlat PE=1 SV=2	
EIF3B_MOUSE	Eukaryotic translation initiation factor 3 subunit B OS=Mus musculus OX=10090 GN=Eif3b PE=1 SV=1	
H2AY_MOUSE	Core histone macro-H2A.1 OS=Mus musculus OX=10090 GN=H2afy PE=1 SV=3	
IF4G1_MOUSE	Eukaryotic translation initiation factor 4 gamma 1 OS=Mus musculus OX=10090 GN=Eif4g1 PE=1 SV=1	
TPIS_MOUSE	Triosephosphate isomerase OS=Mus musculus OX=10090 GN=Tpi1 PE=1 SV=4	
C1TC_MOUSE	C-1-tetrahydrofolate synthase, cytoplasmic OS=Mus musculus OX=10090 GN=Mthfd1 PE=1 SV=4	
HNRPQ_MOUSE	Heterogeneous nuclear ribonucleoprotein Q OS=Mus musculus OX=10090 GN=Syncrip PE=1 SV=2	
LIS1_MOUSE	Platelet-activating factor acetylhydrolase IB subunit alpha OS=Mus musculus OX=10090 GN=Pafah1b1 PE=1 SV=2	
NEST_MOUSE	Nestin OS=Mus musculus OX=10090 GN=Nes PE=1 SV=1	
RAB14_MOUSE	Ras-related protein Rab-14 OS=Mus musculus OX=10090 GN=Rab14 PE=1 SV=3	
SAHH_MOUSE	Adenosylhomocysteinase OS=Mus musculus OX=10090 GN=Ahcy PE=1 SV=3	
ATPG_MOUSE	ATP synthase subunit gamma, mitochondrial OS=Mus musculus OX=10090 GN=Atp5f1c PE=1 SV=1	
ATX10_MOUSE	Ataxin-10 OS=Mus musculus OX=10090 GN=Atxn10 PE=1 SV=2	
DEST_MOUSE	Destrin OS=Mus musculus OX=10090 GN=Dstn PE=1 SV=3	
IGHG3_MOUSE	Ig gamma-3 chain C region OS=Mus musculus OX=10090 PE=1 SV=2	
MK01_MOUSE	Mitogen-activated protein kinase 1 OS=Mus musculus OX=10090 GN=Mapk1 PE=1 SV=3	
TFR1_MOUSE	Transferrin receptor protein 1 OS=Mus musculus OX=10090 GN=Tfrc PE=1 SV=1	
GHC1_MOUSE	Mitochondrial glutamate carrier 1 OS=Mus musculus OX=10090 GN=Slc25a22 PE=1 SV=1	
RAB2A_MOUSE	Ras-related protein Rab-2A OS=Mus musculus OX=10090 GN=Rab2a PE=1 SV=1	
RL3_MOUSE	60S ribosomal protein L3 OS=Mus musculus OX=10090 GN=Rpl3 PE=1 SV=3	
RL6_MOUSE	60S ribosomal protein L6 OS=Mus musculus OX=10090 GN=Rpl6 PE=1 SV=3	
DCTN2_MOUSE	Dynactin subunit 2 OS=Mus musculus OX=10090 GN=Dctn2 PE=1 SV=3	
M2OM_MOUSE	Mitochondrial 2-oxoglutarate/malate carrier protein OS=Mus musculus OX=10090 GN=Slc25a11 PE=1 SV=3	
PEBP1_MOUSE	Phosphatidylethanolamine-binding protein 1 OS=Mus musculus OX=10090 GN=Pebp1 PE=1 SV=3	
PRS7_MOUSE	26S proteasome regulatory subunit 7 OS=Mus musculus OX=10090 GN=Psmc2 PE=1 SV=5	
1433T_MOUSE	14-3-3 protein theta OS=Mus musculus OX=10090 GN=Ywhaq PE=1 SV=1	
CAMKV_MOUSE	CaM kinase-like vesicle-associated protein OS=Mus musculus OX=10090 GN=Camkv PE=1 SV=2	
COPA_MOUSE	Coatomer subunit alpha OS=Mus musculus OX=10090 GN=Copa PE=1 SV=2	
CTNA2_MOUSE	Catenin alpha-2 OS=Mus musculus OX=10090 GN=Ctnna2 PE=1 SV=3	
PGAP1_MOUSE	GPI inositol-deacylase OS=Mus musculus OX=10090 GN=Pgap1 PE=1 SV=3	
SYNJ1_MOUSE	Synaptojanin-1 OS=Mus musculus OX=10090 GN=Synj1 PE=1 SV=3	
BACH_MOUSE	Cytosolic acyl coenzyme A thioester hydrolase OS=Mus musculus OX=10090 GN=Acot7 PE=1 SV=2	
DPYL4_MOUSE	Dihydropyrimidinase-related protein 4 OS=Mus musculus OX=10090 GN=Dpysl4 PE=1 SV=1	
E41L3_MOUSE	Band 4.1-like protein 3 OS=Mus musculus OX=10090 GN=Epb41l3 PE=1 SV=1	
SYNC_MOUSE	Asparagine--tRNA ligase, cytoplasmic OS=Mus musculus OX=10090 GN=Nars PE=1 SV=2	
VATH_MOUSE	V-type proton ATPase subunit H OS=Mus musculus OX=10090 GN=Atp6v1h PE=1 SV=1	
E41L1_MOUSE	Band 4.1-like protein 1 OS=Mus musculus OX=10090 GN=Epb41l1 PE=1 SV=2	
IPO5_MOUSE	Importin-5 OS=Mus musculus OX=10090 GN=Ipo5 PE=1 SV=3	
SEPT7_MOUSE	Septin-7 OS=Mus musculus OX=10090 GN=Septin7 PE=1 SV=1	
SND1_MOUSE	Staphylococcal nuclease domain-containing protein 1 OS=Mus musculus OX=10090 GN=Snd1 PE=1 SV=1	
TOM70_MOUSE	Mitochondrial import receptor subunit TOM70 OS=Mus musculus OX=10090 GN=Tomm70 PE=1 SV=2	
PP1B_MOUSE	Serine/threonine-protein phosphatase PP1-beta catalytic subunit OS=Mus musculus OX=10090 GN=Ppp1cb PE=1 SV=3	
PRDX1_MOUSE	Peroxiredoxin-1 OS=Mus musculus OX=10090 GN=Prdx1 PE=1 SV=1	
VA0D1_MOUSE	V-type proton ATPase subunit d 1 OS=Mus musculus OX=10090 GN=Atp6v0d1 PE=1 SV=2	
ALDH2_MOUSE	Aldehyde dehydrogenase, mitochondrial OS=Mus musculus OX=10090 GN=Aldh2 PE=1 SV=1	
IDH3A_MOUSE	Isocitrate dehydrogenase [NAD] subunit alpha, mitochondrial OS=Mus musculus OX=10090 GN=Idh3a PE=1 SV=1	
PSMD3_MOUSE	26S proteasome non-ATPase regulatory subunit 3 OS=Mus musculus OX=10090 GN=Psmd3 PE=1 SV=3	
RL7_MOUSE	60S ribosomal protein L7 OS=Mus musculus OX=10090 GN=Rpl7 PE=1 SV=2	
VATC1_MOUSE	V-type proton ATPase subunit C 1 OS=Mus musculus OX=10090 GN=Atp6v1c1 PE=1 SV=4	
CLAP2_MOUSE	CLIP-associating protein 2 OS=Mus musculus OX=10090 GN=Clasp2 PE=1 SV=1	
ENOG_MOUSE	Gamma-enolase OS=Mus musculus OX=10090 GN=Eno2 PE=1 SV=2	
MYEF2_MOUSE	Myelin expression factor 2 OS=Mus musculus OX=10090 GN=Myef2 PE=1 SV=1	
OAT_MOUSE	Ornithine aminotransferase, mitochondrial OS=Mus musculus OX=10090 GN=Oat PE=1 SV=1	
PRDX2_MOUSE	Peroxiredoxin-2 OS=Mus musculus OX=10090 GN=Prdx2 PE=1 SV=3	
PROF1_MOUSE	Profilin-1 OS=Mus musculus OX=10090 GN=Pfn1 PE=1 SV=2	
STX1B_MOUSE	Syntaxin-1B OS=Mus musculus OX=10090 GN=Stx1b PE=1 SV=1	
AP3B2_MOUSE	AP-3 complex subunit beta-2 OS=Mus musculus OX=10090 GN=Ap3b2 PE=1 SV=2	
ARF1_MOUSE	ADP-ribosylation factor 1 OS=Mus musculus OX=10090 GN=Arf1 PE=1 SV=2	
CTNB1_HUMAN	Catenin beta-1 OS=Homo sapiens OX=9606 GN=CTNNB1 PE=1 SV=1	
NCKP1_MOUSE	Nck-associated protein 1 OS=Mus musculus OX=10090 GN=Nckap1 PE=1 SV=2	
NEUM_MOUSE	Neuromodulin OS=Mus musculus OX=10090 GN=Gap43 PE=1 SV=1	
RS3A_MOUSE	40S ribosomal protein S3a OS=Mus musculus OX=10090 GN=Rps3a PE=1 SV=3	
RTN1_MOUSE	Reticulon-1 OS=Mus musculus OX=10090 GN=Rtn1 PE=1 SV=1	
TIF1B_MOUSE	Transcription intermediary factor 1-beta OS=Mus musculus OX=10090 GN=Trim28 PE=1 SV=3	
1433B_MOUSE	14-3-3 protein beta/alpha OS=Mus musculus OX=10090 GN=Ywhab PE=1 SV=3	
AT5F1_MOUSE	ATP synthase F(0) complex subunit B1, mitochondrial OS=Mus musculus OX=10090 GN=Atp5pb PE=1 SV=1	
CAPZB_MOUSE	F-actin-capping protein subunit beta OS=Mus musculus OX=10090 GN=Capzb PE=1 SV=3	
HMCS1_MOUSE	Hydroxymethylglutaryl-CoA synthase, cytoplasmic OS=Mus musculus OX=10090 GN=Hmgcs1 PE=1 SV=1	
PRS8_MOUSE	26S proteasome regulatory subunit 8 OS=Mus musculus OX=10090 GN=Psmc5 PE=1 SV=1	
RL7A_MOUSE	60S ribosomal protein L7a OS=Mus musculus OX=10090 GN=Rpl7a PE=1 SV=2	
ACTZ_MOUSE	Alpha-centractin OS=Mus musculus OX=10090 GN=Actr1a PE=1 SV=1	
MTCH2_MOUSE	Mitochondrial carrier homolog 2 OS=Mus musculus OX=10090 GN=Mtch2 PE=1 SV=1	
PSA1_MOUSE	Proteasome subunit alpha type-1 OS=Mus musculus OX=10090 GN=Psma1 PE=1 SV=1	
SRSF1_MOUSE	Serine/arginine-rich splicing factor 1 OS=Mus musculus OX=10090 GN=Srsf1 PE=1 SV=3	
SYDC_MOUSE	Aspartate--tRNA ligase, cytoplasmic OS=Mus musculus OX=10090 GN=Dars PE=1 SV=2	
AMPH_MOUSE	Amphiphysin OS=Mus musculus OX=10090 GN=Amph PE=1 SV=1	
ATPO_MOUSE	ATP synthase subunit O, mitochondrial OS=Mus musculus OX=10090 GN=Atp5po PE=1 SV=1	
PP2BA_MOUSE	Serine/threonine-protein phosphatase 2B catalytic subunit alpha isoform OS=Mus musculus OX=10090 GN=Ppp3ca PE=1 SV=1	
RTCB_MOUSE	tRNA-splicing ligase RtcB homolog OS=Mus musculus OX=10090 GN=Rtcb PE=1 SV=1	
SCRN1_MOUSE	Secernin-1 OS=Mus musculus OX=10090 GN=Scrn1 PE=1 SV=1	
ADDA_MOUSE	Alpha-adducin OS=Mus musculus OX=10090 GN=Add1 PE=1 SV=2	
BDH_MOUSE	D-beta-hydroxybutyrate dehydrogenase, mitochondrial OS=Mus musculus OX=10090 GN=Bdh1 PE=1 SV=2	
GFAP_MOUSE	Glial fibrillary acidic protein OS=Mus musculus OX=10090 GN=Gfap PE=1 SV=4	
RS8_MOUSE	40S ribosomal protein S8 OS=Mus musculus OX=10090 GN=Rps8 PE=1 SV=2	
SNAB_MOUSE	Beta-soluble NSF attachment protein OS=Mus musculus OX=10090 GN=Napb PE=1 SV=2	
NB5R3_MOUSE	NADH-cytochrome b5 reductase 3 OS=Mus musculus OX=10090 GN=Cyb5r3 PE=1 SV=3	
RS2_MOUSE	40S ribosomal protein S2 OS=Mus musculus OX=10090 GN=Rps2 PE=1 SV=3	
ATP5H_MOUSE	ATP synthase subunit d, mitochondrial OS=Mus musculus OX=10090 GN=Atp5pd PE=1 SV=3	
IF5A1_MOUSE	Eukaryotic translation initiation factor 5A-1 OS=Mus musculus OX=10090 GN=Eif5a PE=1 SV=2	
PSIP1_MOUSE	PC4 and SFRS1-interacting protein OS=Mus musculus OX=10090 GN=Psip1 PE=1 SV=1	
SERC_MOUSE	Phosphoserine aminotransferase OS=Mus musculus OX=10090 GN=Psat1 PE=1 SV=1	
TAGL_MOUSE	Transgelin OS=Mus musculus OX=10090 GN=Tagln PE=1 SV=3	
1433F_MOUSE	14-3-3 protein eta OS=Mus musculus OX=10090 GN=Ywhah PE=1 SV=2	
AATC_MOUSE	Aspartate aminotransferase, cytoplasmic OS=Mus musculus OX=10090 GN=Got1 PE=1 SV=3	
CON__P15497	CON__P15497	
EAA1_MOUSE	Excitatory amino acid transporter 1 OS=Mus musculus OX=10090 GN=Slc1a3 PE=1 SV=2	
PURB_MOUSE	Transcriptional activator protein Pur-beta OS=Mus musculus OX=10090 GN=Purb PE=1 SV=3	
SSDH_MOUSE	Succinate-semialdehyde dehydrogenase, mitochondrial OS=Mus musculus OX=10090 GN=Aldh5a1 PE=1 SV=1	
VATE1_MOUSE	V-type proton ATPase subunit E 1 OS=Mus musculus OX=10090 GN=Atp6v1e1 PE=1 SV=2	
ANXA3_MOUSE	Annexin A3 OS=Mus musculus OX=10090 GN=Anxa3 PE=1 SV=4	
ETFA_MOUSE	Electron transfer flavoprotein subunit alpha, mitochondrial OS=Mus musculus OX=10090 GN=Etfa PE=1 SV=2	
OST48_MOUSE	Dolichyl-diphosphooligosaccharide--protein glycosyltransferase 48 kDa subunit OS=Mus musculus OX=10090 GN=Ddost PE=1 SV=2	
OTUB1_MOUSE	Ubiquitin thioesterase OTUB1 OS=Mus musculus OX=10090 GN=Otub1 PE=1 SV=2	
PSD12_MOUSE	26S proteasome non-ATPase regulatory subunit 12 OS=Mus musculus OX=10090 GN=Psmd12 PE=1 SV=4	
UCRI_MOUSE	Cytochrome b-c1 complex subunit Rieske, mitochondrial OS=Mus musculus OX=10090 GN=Uqcrfs1 PE=1 SV=1	
CCAR2_MOUSE	Cell cycle and apoptosis regulator protein 2 OS=Mus musculus OX=10090 GN=Ccar2 PE=1 SV=2	
CNN3_MOUSE	Calponin-3 OS=Mus musculus OX=10090 GN=Cnn3 PE=1 SV=1	
RSSA_MOUSE	40S ribosomal protein SA OS=Mus musculus OX=10090 GN=Rpsa PE=1 SV=4	
SFXN3_MOUSE	Sideroflexin-3 OS=Mus musculus OX=10090 GN=Sfxn3 PE=1 SV=1	
TOP1_MOUSE	DNA topoisomerase 1 OS=Mus musculus OX=10090 GN=Top1 PE=1 SV=2	
DHX15_MOUSE	Pre-mRNA-splicing factor ATP-dependent RNA helicase DHX15 OS=Mus musculus OX=10090 GN=Dhx15 PE=1 SV=2	
GNAQ_MOUSE	Guanine nucleotide-binding protein G(q) subunit alpha OS=Mus musculus OX=10090 GN=Gnaq PE=1 SV=4	
GPC4_MOUSE	Glypican-4 OS=Mus musculus OX=10090 GN=Gpc4 PE=1 SV=2	
RAB7A_MOUSE	Ras-related protein Rab-7a OS=Mus musculus OX=10090 GN=Rab7a PE=1 SV=2	
RL5_MOUSE	60S ribosomal protein L5 OS=Mus musculus OX=10090 GN=Rpl5 PE=1 SV=3	
SV2A_MOUSE	Synaptic vesicle glycoprotein 2A OS=Mus musculus OX=10090 GN=Sv2a PE=1 SV=1	
SYSC_MOUSE	Serine--tRNA ligase, cytoplasmic OS=Mus musculus OX=10090 GN=Sars PE=1 SV=3	
SYVC_MOUSE	Valine--tRNA ligase OS=Mus musculus OX=10090 GN=Vars PE=1 SV=1	
AT2B2_MOUSE	Plasma membrane calcium-transporting ATPase 2 OS=Mus musculus OX=10090 GN=Atp2b2 PE=1 SV=2	
IGHM_MOUSE	Immunoglobulin heavy constant mu OS=Mus musculus OX=10090 GN=Ighm PE=1 SV=2	
IPYR_MOUSE	Inorganic pyrophosphatase OS=Mus musculus OX=10090 GN=Ppa1 PE=1 SV=1	
LA_MOUSE	Lupus La protein homolog OS=Mus musculus OX=10090 GN=Ssb PE=1 SV=1	
SEP11_MOUSE	Septin-11 OS=Mus musculus OX=10090 GN=Septin11 PE=1 SV=4	
SYN2_MOUSE	Synapsin-2 OS=Mus musculus OX=10090 GN=Syn2 PE=1 SV=2	
U5S1_MOUSE	116 kDa U5 small nuclear ribonucleoprotein component OS=Mus musculus OX=10090 GN=Eftud2 PE=1 SV=1	
CBPE_MOUSE	Carboxypeptidase E OS=Mus musculus OX=10090 GN=Cpe PE=1 SV=2	
FUBP2_MOUSE	Far upstream element-binding protein 2 OS=Mus musculus OX=10090 GN=Khsrp PE=1 SV=2	
FUMH_MOUSE	Fumarate hydratase, mitochondrial OS=Mus musculus OX=10090 GN=Fh PE=1 SV=3	
HYEP_MOUSE	Epoxide hydrolase 1 OS=Mus musculus OX=10090 GN=Ephx1 PE=1 SV=2	
KLC1_MOUSE	Kinesin light chain 1 OS=Mus musculus OX=10090 GN=Klc1 PE=1 SV=3	
NDKA_MOUSE	Nucleoside diphosphate kinase A OS=Mus musculus OX=10090 GN=Nme1 PE=1 SV=1	
PDIA4_MOUSE	Protein disulfide-isomerase A4 OS=Mus musculus OX=10090 GN=Pdia4 PE=1 SV=3	
ADT2_MOUSE	ADP/ATP translocase 2 OS=Mus musculus OX=10090 GN=Slc25a5 PE=1 SV=3	
IF4A3_MOUSE	Eukaryotic initiation factor 4A-III OS=Mus musculus OX=10090 GN=Eif4a3 PE=1 SV=3	
MFGM_MOUSE	Lactadherin OS=Mus musculus OX=10090 GN=Mfge8 PE=1 SV=3	
PDIA6_MOUSE	Protein disulfide-isomerase A6 OS=Mus musculus OX=10090 GN=Pdia6 PE=1 SV=3	
RAB6A_MOUSE	Ras-related protein Rab-6A OS=Mus musculus OX=10090 GN=Rab6a PE=1 SV=4	
SYLC_MOUSE	Leucine--tRNA ligase, cytoplasmic OS=Mus musculus OX=10090 GN=Lars PE=1 SV=2	
VDAC2_MOUSE	Voltage-dependent anion-selective channel protein 2 OS=Mus musculus OX=10090 GN=Vdac2 PE=1 SV=2	
HVM02_MOUSE	Ig heavy chain V region 93G7 OS=Mus musculus OX=10090 PE=2 SV=1	
ACAD9_MOUSE	Complex I assembly factor ACAD9, mitochondrial OS=Mus musculus OX=10090 GN=Acad9 PE=1 SV=2	
DDAH1_MOUSE	N(G),N(G)-dimethylarginine dimethylaminohydrolase 1 OS=Mus musculus OX=10090 GN=Ddah1 PE=1 SV=3	
H2AW_MOUSE	Core histone macro-H2A.2 OS=Mus musculus OX=10090 GN=H2afy2 PE=1 SV=3	
IF2G_MOUSE	Eukaryotic translation initiation factor 2 subunit 3, X-linked OS=Mus musculus OX=10090 GN=Eif2s3x PE=1 SV=2	
SODM_MOUSE	Superoxide dismutase [Mn], mitochondrial OS=Mus musculus OX=10090 GN=Sod2 PE=1 SV=3	
TICN2_MOUSE	Testican-2 OS=Mus musculus OX=10090 GN=Spock2 PE=1 SV=1	
AT1A2_MOUSE	Sodium/potassium-transporting ATPase subunit alpha-2 OS=Mus musculus OX=10090 GN=Atp1a2 PE=1 SV=1	
FABP5_MOUSE	Fatty acid-binding protein 5 OS=Mus musculus OX=10090 GN=Fabp5 PE=1 SV=3	
KINH_MOUSE	Kinesin-1 heavy chain OS=Mus musculus OX=10090 GN=Kif5b PE=1 SV=3	
OPA1_MOUSE	Dynamin-like 120 kDa protein, mitochondrial OS=Mus musculus OX=10090 GN=Opa1 PE=1 SV=1	
PSD13_MOUSE	26S proteasome non-ATPase regulatory subunit 13 OS=Mus musculus OX=10090 GN=Psmd13 PE=1 SV=1	
RUVB2_MOUSE	RuvB-like 2 OS=Mus musculus OX=10090 GN=Ruvbl2 PE=1 SV=3	
SNP25_MOUSE	Synaptosomal-associated protein 25 OS=Mus musculus OX=10090 GN=Snap25 PE=1 SV=1	
TADBP_MOUSE	TAR DNA-binding protein 43 OS=Mus musculus OX=10090 GN=Tardbp PE=1 SV=1	
ALDR_MOUSE	Aldo-keto reductase family 1 member B1 OS=Mus musculus OX=10090 GN=Akr1b1 PE=1 SV=3	
ARC1A_MOUSE	Actin-related protein 2/3 complex subunit 1A OS=Mus musculus OX=10090 GN=Arpc1a PE=1 SV=1	
C1QC_MOUSE	Complement C1q subcomponent subunit C OS=Mus musculus OX=10090 GN=C1qc PE=1 SV=2	
COPG1_MOUSE	Coatomer subunit gamma-1 OS=Mus musculus OX=10090 GN=Copg1 PE=1 SV=1	
PRDX3_MOUSE	Thioredoxin-dependent peroxide reductase, mitochondrial OS=Mus musculus OX=10090 GN=Prdx3 PE=1 SV=1	
PUR6_MOUSE	Multifunctional protein ADE2 OS=Mus musculus OX=10090 GN=Paics PE=1 SV=4	
RAB3C_MOUSE	Ras-related protein Rab-3C OS=Mus musculus OX=10090 GN=Rab3c PE=1 SV=1	
RMXL1_MOUSE	RNA binding motif protein, X-linked-like-1 OS=Mus musculus OX=10090 GN=Rbmxl1 PE=2 SV=1	
S12A5_MOUSE	Solute carrier family 12 member 5 OS=Mus musculus OX=10090 GN=Slc12a5 PE=1 SV=2	
YBOX1_MOUSE	Y-box-binding protein 1 OS=Mus musculus OX=10090 GN=Ybx1 PE=1 SV=3	
ETFB_MOUSE	Electron transfer flavoprotein subunit beta OS=Mus musculus OX=10090 GN=Etfb PE=1 SV=3	
GBB2_MOUSE	Guanine nucleotide-binding protein G(I)/G(S)/G(T) subunit beta-2 OS=Mus musculus OX=10090 GN=Gnb2 PE=1 SV=3	
HP1B3_MOUSE	Heterochromatin protein 1-binding protein 3 OS=Mus musculus OX=10090 GN=Hp1bp3 PE=1 SV=1	
ILF3_MOUSE	Interleukin enhancer-binding factor 3 OS=Mus musculus OX=10090 GN=Ilf3 PE=1 SV=2	
PA2G4_MOUSE	Proliferation-associated protein 2G4 OS=Mus musculus OX=10090 GN=Pa2g4 PE=1 SV=3	
VDAC3_MOUSE	Voltage-dependent anion-selective channel protein 3 OS=Mus musculus OX=10090 GN=Vdac3 PE=1 SV=1	
6PGD_MOUSE	6-phosphogluconate dehydrogenase, decarboxylating OS=Mus musculus OX=10090 GN=Pgd PE=1 SV=3	
CY1_MOUSE	Cytochrome c1, heme protein, mitochondrial OS=Mus musculus OX=10090 GN=Cyc1 PE=1 SV=1	
PRS6A_MOUSE	26S proteasome regulatory subunit 6A OS=Mus musculus OX=10090 GN=Psmc3 PE=1 SV=2	
RAB5C_MOUSE	Ras-related protein Rab-5C OS=Mus musculus OX=10090 GN=Rab5c PE=1 SV=2	
RS27A_MOUSE	Ubiquitin-40S ribosomal protein S27a OS=Mus musculus OX=10090 GN=Rps27a PE=1 SV=2	
VISL1_MOUSE	Visinin-like protein 1 OS=Mus musculus OX=10090 GN=Vsnl1 PE=1 SV=2	
AGRIN_MOUSE	Agrin OS=Mus musculus OX=10090 GN=Agrn PE=1 SV=1	
EIF3C_MOUSE	Eukaryotic translation initiation factor 3 subunit C OS=Mus musculus OX=10090 GN=Eif3c PE=1 SV=1	
ESTD_MOUSE	S-formylglutathione hydrolase OS=Mus musculus OX=10090 GN=Esd PE=1 SV=1	
KAD1_MOUSE	Adenylate kinase isoenzyme 1 OS=Mus musculus OX=10090 GN=Ak1 PE=1 SV=1	
PPCE_MOUSE	Prolyl endopeptidase OS=Mus musculus OX=10090 GN=Prep PE=1 SV=1	
PRS10_MOUSE	26S proteasome regulatory subunit 10B OS=Mus musculus OX=10090 GN=Psmc6 PE=1 SV=1	
ARP2_MOUSE	Actin-related protein 2 OS=Mus musculus OX=10090 GN=Actr2 PE=1 SV=1	
ARPC2_MOUSE	Actin-related protein 2/3 complex subunit 2 OS=Mus musculus OX=10090 GN=Arpc2 PE=1 SV=3	
COPB_MOUSE	Coatomer subunit beta OS=Mus musculus OX=10090 GN=Copb1 PE=1 SV=1	
ECHM_MOUSE	Enoyl-CoA hydratase, mitochondrial OS=Mus musculus OX=10090 GN=Echs1 PE=1 SV=1	
GDIR1_MOUSE	Rho GDP-dissociation inhibitor 1 OS=Mus musculus OX=10090 GN=Arhgdia PE=1 SV=3	
GRIN1_MOUSE	G protein-regulated inducer of neurite outgrowth 1 OS=Mus musculus OX=10090 GN=Gprin1 PE=1 SV=2	
HS12A_MOUSE	Heat shock 70 kDa protein 12A OS=Mus musculus OX=10090 GN=Hspa12a PE=1 SV=1	
P5CS_MOUSE	Delta-1-pyrroline-5-carboxylate synthase OS=Mus musculus OX=10090 GN=Aldh18a1 PE=1 SV=2	
PRS6B_MOUSE	26S proteasome regulatory subunit 6B OS=Mus musculus OX=10090 GN=Psmc4 PE=1 SV=2	
RLA0_MOUSE	60S acidic ribosomal protein P0 OS=Mus musculus OX=10090 GN=Rplp0 PE=1 SV=3	
RUFY3_MOUSE	Protein RUFY3 OS=Mus musculus OX=10090 GN=Rufy3 PE=1 SV=1	
SERA_MOUSE	D-3-phosphoglycerate dehydrogenase OS=Mus musculus OX=10090 GN=Phgdh PE=1 SV=3	
AP3D1_MOUSE	AP-3 complex subunit delta-1 OS=Mus musculus OX=10090 GN=Ap3d1 PE=1 SV=1	
CBR1_MOUSE	Carbonyl reductase [NADPH] 1 OS=Mus musculus OX=10090 GN=Cbr1 PE=1 SV=3	
CTBP1_MOUSE	C-terminal-binding protein 1 OS=Mus musculus OX=10090 GN=Ctbp1 PE=1 SV=2	
DCX_MOUSE	Neuronal migration protein doublecortin OS=Mus musculus OX=10090 GN=Dcx PE=1 SV=1	
DHB12_MOUSE	Very-long-chain 3-oxoacyl-CoA reductase OS=Mus musculus OX=10090 GN=Hsd17b12 PE=1 SV=1	
FPPS_MOUSE	Farnesyl pyrophosphate synthase OS=Mus musculus OX=10090 GN=Fdps PE=1 SV=1	
HNRPC_MOUSE	Heterogeneous nuclear ribonucleoproteins C1/C2 OS=Mus musculus OX=10090 GN=Hnrnpc PE=1 SV=1	
MRP_MOUSE	MARCKS-related protein OS=Mus musculus OX=10090 GN=Marcksl1 PE=1 SV=2	
NDUA9_MOUSE	NADH dehydrogenase [ubiquinone] 1 alpha subcomplex subunit 9, mitochondrial OS=Mus musculus OX=10090 GN=Ndufa9 PE=1 SV=2	
TPP2_MOUSE	Tripeptidyl-peptidase 2 OS=Mus musculus OX=10090 GN=Tpp2 PE=1 SV=3	
MAP4_MOUSE	Microtubule-associated protein 4 OS=Mus musculus OX=10090 GN=Map4 PE=1 SV=3	
PGRC1_MOUSE	Membrane-associated progesterone receptor component 1 OS=Mus musculus OX=10090 GN=Pgrmc1 PE=1 SV=4	
PHIPL_MOUSE	Phytanoyl-CoA hydroxylase-interacting protein-like OS=Mus musculus OX=10090 GN=Phyhipl PE=1 SV=1	
PSB2_MOUSE	Proteasome subunit beta type-2 OS=Mus musculus OX=10090 GN=Psmb2 PE=1 SV=1	
RL12_MOUSE	60S ribosomal protein L12 OS=Mus musculus OX=10090 GN=Rpl12 PE=1 SV=2	
TRAP1_MOUSE	Heat shock protein 75 kDa, mitochondrial OS=Mus musculus OX=10090 GN=Trap1 PE=1 SV=1	
CAZA2_MOUSE	F-actin-capping protein subunit alpha-2 OS=Mus musculus OX=10090 GN=Capza2 PE=1 SV=3	
CLUS_MOUSE	Clusterin OS=Mus musculus OX=10090 GN=Clu PE=1 SV=1	
COPB2_MOUSE	Coatomer subunit beta' OS=Mus musculus OX=10090 GN=Copb2 PE=1 SV=2	
IGG2A_RAT	Ig gamma-2A chain C region OS=Rattus norvegicus OX=10116 GN=Igg-2a PE=1 SV=1	
IPO7_HUMAN	Importin-7 OS=Homo sapiens OX=9606 GN=IPO7 PE=1 SV=1	
LMNA_MOUSE	Prelamin-A/C OS=Mus musculus OX=10090 GN=Lmna PE=1 SV=2	
RAN_MOUSE	GTP-binding nuclear protein Ran OS=Mus musculus OX=10090 GN=Ran PE=1 SV=3	
ANM1_MOUSE	Protein arginine N-methyltransferase 1 OS=Mus musculus OX=10090 GN=Prmt1 PE=1 SV=1	
COX41_MOUSE	Cytochrome c oxidase subunit 4 isoform 1, mitochondrial OS=Mus musculus OX=10090 GN=Cox4i1 PE=1 SV=2	
GSTP1_MOUSE	Glutathione S-transferase P 1 OS=Mus musculus OX=10090 GN=Gstp1 PE=1 SV=2	
KAPCB_MOUSE	cAMP-dependent protein kinase catalytic subunit beta OS=Mus musculus OX=10090 GN=Prkacb PE=1 SV=2	
NDRG3_MOUSE	Protein NDRG3 OS=Mus musculus OX=10090 GN=Ndrg3 PE=1 SV=1	
NDUS2_MOUSE	NADH dehydrogenase [ubiquinone] iron-sulfur protein 2, mitochondrial OS=Mus musculus OX=10090 GN=Ndufs2 PE=1 SV=1	
NPM_MOUSE	Nucleophosmin OS=Mus musculus OX=10090 GN=Npm1 PE=1 SV=1	
PACN1_MOUSE	Protein kinase C and casein kinase substrate in neurons protein 1 OS=Mus musculus OX=10090 GN=Pacsin1 PE=1 SV=1	
SYRC_MOUSE	Arginine--tRNA ligase, cytoplasmic OS=Mus musculus OX=10090 GN=Rars PE=1 SV=2	
ALDOC_MOUSE	Fructose-bisphosphate aldolase C OS=Mus musculus OX=10090 GN=Aldoc PE=1 SV=4	
HMGB1_MOUSE	High mobility group protein B1 OS=Mus musculus OX=10090 GN=Hmgb1 PE=1 SV=2	
HNRH2_MOUSE	Heterogeneous nuclear ribonucleoprotein H2 OS=Mus musculus OX=10090 GN=Hnrnph2 PE=1 SV=1	
KCY_MOUSE	UMP-CMP kinase OS=Mus musculus OX=10090 GN=Cmpk1 PE=1 SV=1	
NCALD_MOUSE	Neurocalcin-delta OS=Mus musculus OX=10090 GN=Ncald PE=1 SV=4	
RS18_MOUSE	40S ribosomal protein S18 OS=Mus musculus OX=10090 GN=Rps18 PE=1 SV=3	
SF3B1_MOUSE	Splicing factor 3B subunit 1 OS=Mus musculus OX=10090 GN=Sf3b1 PE=1 SV=1	
THOP1_MOUSE	Thimet oligopeptidase OS=Mus musculus OX=10090 GN=Thop1 PE=1 SV=1	
TOM22_MOUSE	Mitochondrial import receptor subunit TOM22 homolog OS=Mus musculus OX=10090 GN=Tomm22 PE=1 SV=3	
AKAP5_MOUSE	A-kinase anchor protein 5 OS=Mus musculus OX=10090 GN=Akap5 PE=1 SV=2	
ASNS_MOUSE	Asparagine synthetase [glutamine-hydrolyzing] OS=Mus musculus OX=10090 GN=Asns PE=1 SV=3	
AT8A1_MOUSE	Phospholipid-transporting ATPase IA OS=Mus musculus OX=10090 GN=Atp8a1 PE=1 SV=2	
CBX3_MOUSE	Chromobox protein homolog 3 OS=Mus musculus OX=10090 GN=Cbx3 PE=1 SV=2	
CDC42_CHICK	Cell division control protein 42 homolog OS=Gallus gallus OX=9031 GN=CDC42 PE=2 SV=1	
DDX6_MOUSE	Probable ATP-dependent RNA helicase DDX6 OS=Mus musculus OX=10090 GN=Ddx6 PE=1 SV=1	
DPP6_MOUSE	Dipeptidyl aminopeptidase-like protein 6 OS=Mus musculus OX=10090 GN=Dpp6 PE=1 SV=1	
HCD2_MOUSE	3-hydroxyacyl-CoA dehydrogenase type-2 OS=Mus musculus OX=10090 GN=Hsd17b10 PE=1 SV=4	
HNRPD_MOUSE	Heterogeneous nuclear ribonucleoprotein D0 OS=Mus musculus OX=10090 GN=Hnrnpd PE=1 SV=2	
KCC2D_MOUSE	Calcium/calmodulin-dependent protein kinase type II subunit delta OS=Mus musculus OX=10090 GN=Camk2d PE=1 SV=1	
NOP56_MOUSE	Nucleolar protein 56 OS=Mus musculus OX=10090 GN=Nop56 PE=1 SV=2	
PGM1_MOUSE	Phosphoglucomutase-1 OS=Mus musculus OX=10090 GN=Pgm1 PE=1 SV=4	
PSB5_MOUSE	Proteasome subunit beta type-5 OS=Mus musculus OX=10090 GN=Psmb5 PE=1 SV=3	
RAB10_MOUSE	Ras-related protein Rab-10 OS=Mus musculus OX=10090 GN=Rab10 PE=1 SV=1	
RL9_MOUSE	60S ribosomal protein L9 OS=Mus musculus OX=10090 GN=Rpl9 PE=2 SV=2	
VGF_MOUSE	Neurosecretory protein VGF OS=Mus musculus OX=10090 GN=Vgf PE=1 SV=1	
ACADV_MOUSE	Very long-chain specific acyl-CoA dehydrogenase, mitochondrial OS=Mus musculus OX=10090 GN=Acadvl PE=1 SV=3	
DDX17_MOUSE	Probable ATP-dependent RNA helicase DDX17 OS=Mus musculus OX=10090 GN=Ddx17 PE=1 SV=1	
EF1A2_MOUSE	Elongation factor 1-alpha 2 OS=Mus musculus OX=10090 GN=Eef1a2 PE=1 SV=1	
EIF3E_HUMAN	Eukaryotic translation initiation factor 3 subunit E OS=Homo sapiens OX=9606 GN=EIF3E PE=1 SV=1	
MARE1_MOUSE	Microtubule-associated protein RP/EB family member 1 OS=Mus musculus OX=10090 GN=Mapre1 PE=1 SV=3	
MMSA_MOUSE	Methylmalonate-semialdehyde dehydrogenase [acylating], mitochondrial OS=Mus musculus OX=10090 GN=Aldh6a1 PE=1 SV=1	
NDUV1_MOUSE	NADH dehydrogenase [ubiquinone] flavoprotein 1, mitochondrial OS=Mus musculus OX=10090 GN=Ndufv1 PE=1 SV=1	
NOMO1_MOUSE	Nodal modulator 1 OS=Mus musculus OX=10090 GN=Nomo1 PE=1 SV=1	
PEA15_MOUSE	Astrocytic phosphoprotein PEA-15 OS=Mus musculus OX=10090 GN=Pea15 PE=1 SV=1	
PRS4_MOUSE	26S proteasome regulatory subunit 4 OS=Mus musculus OX=10090 GN=Psmc1 PE=1 SV=1	
PSMD6_MOUSE	26S proteasome non-ATPase regulatory subunit 6 OS=Mus musculus OX=10090 GN=Psmd6 PE=1 SV=1	
RB11B_MOUSE	Ras-related protein Rab-11B OS=Mus musculus OX=10090 GN=Rab11b PE=1 SV=3	
RL15_MOUSE	60S ribosomal protein L15 OS=Mus musculus OX=10090 GN=Rpl15 PE=1 SV=4	
RS7_MOUSE	40S ribosomal protein S7 OS=Mus musculus OX=10090 GN=Rps7 PE=2 SV=1	
THIC_MOUSE	Acetyl-CoA acetyltransferase, cytosolic OS=Mus musculus OX=10090 GN=Acat2 PE=1 SV=2	
VAT1_MOUSE	Synaptic vesicle membrane protein VAT-1 homolog OS=Mus musculus OX=10090 GN=Vat1 PE=1 SV=3	
MUG1_MOUSE	Murinoglobulin-1 OS=Mus musculus OX=10090 GN=Mug1 PE=1 SV=3	
NBEA_MOUSE	Neurobeachin OS=Mus musculus OX=10090 GN=Nbea PE=1 SV=1	
ACTN1_MOUSE	Alpha-actinin-1 OS=Mus musculus OX=10090 GN=Actn1 PE=1 SV=1	
AN32A_MOUSE	Acidic leucine-rich nuclear phosphoprotein 32 family member A OS=Mus musculus OX=10090 GN=Anp32a PE=1 SV=1	
CERU_MOUSE	Ceruloplasmin OS=Mus musculus OX=10090 GN=Cp PE=1 SV=2	
COR1C_MOUSE	Coronin-1C OS=Mus musculus OX=10090 GN=Coro1c PE=1 SV=2	
ERLN2_MOUSE	Erlin-2 OS=Mus musculus OX=10090 GN=Erlin2 PE=1 SV=1	
FUS_MOUSE	RNA-binding protein FUS OS=Mus musculus OX=10090 GN=Fus PE=1 SV=1	
GUAA_MOUSE	GMP synthase [glutamine-hydrolyzing] OS=Mus musculus OX=10090 GN=Gmps PE=1 SV=2	
HS74L_MOUSE	Heat shock 70 kDa protein 4L OS=Mus musculus OX=10090 GN=Hspa4l PE=1 SV=2	
NOP58_MOUSE	Nucleolar protein 58 OS=Mus musculus OX=10090 GN=Nop58 PE=1 SV=1	
PRP19_MOUSE	Pre-mRNA-processing factor 19 OS=Mus musculus OX=10090 GN=Prpf19 PE=1 SV=1	
PSA6_MOUSE	Proteasome subunit alpha type-6 OS=Mus musculus OX=10090 GN=Psma6 PE=1 SV=1	
RS11_HUMAN	40S ribosomal protein S11 OS=Homo sapiens OX=9606 GN=RPS11 PE=1 SV=3	
SF3B3_MOUSE	Splicing factor 3B subunit 3 OS=Mus musculus OX=10090 GN=Sf3b3 PE=1 SV=1	
CAPR1_MOUSE	Caprin-1 OS=Mus musculus OX=10090 GN=Caprin1 PE=1 SV=2	
KHDR1_MOUSE	KH domain-containing, RNA-binding, signal transduction-associated protein 1 OS=Mus musculus OX=10090 GN=Khdrbs1 PE=1 SV=2	
NDUAA_MOUSE	NADH dehydrogenase [ubiquinone] 1 alpha subcomplex subunit 10, mitochondrial OS=Mus musculus OX=10090 GN=Ndufa10 PE=1 SV=1	
PDXK_MOUSE	Pyridoxal kinase OS=Mus musculus OX=10090 GN=Pdxk PE=1 SV=1	
SC23A_MOUSE	Protein transport protein Sec23A OS=Mus musculus OX=10090 GN=Sec23a PE=1 SV=2	
GCN1_MOUSE	eIF-2-alpha kinase activator GCN1 OS=Mus musculus OX=10090 GN=Gcn1 PE=1 SV=1	
DC1L1_MOUSE	Cytoplasmic dynein 1 light intermediate chain 1 OS=Mus musculus OX=10090 GN=Dync1li1 PE=1 SV=1	
DNJA1_MOUSE	DnaJ homolog subfamily A member 1 OS=Mus musculus OX=10090 GN=Dnaja1 PE=1 SV=1	
FINC_MOUSE	Fibronectin OS=Mus musculus OX=10090 GN=Fn1 PE=1 SV=4	
H15_MOUSE	Histone H1.5 OS=Mus musculus OX=10090 GN=Hist1h1b PE=1 SV=2	
PSD11_MOUSE	26S proteasome non-ATPase regulatory subunit 11 OS=Mus musculus OX=10090 GN=Psmd11 PE=1 SV=3	
RUVB1_MOUSE	RuvB-like 1 OS=Mus musculus OX=10090 GN=Ruvbl1 PE=1 SV=1	
SAHH2_MOUSE	S-adenosylhomocysteine hydrolase-like protein 1 OS=Mus musculus OX=10090 GN=Ahcyl1 PE=1 SV=1	
SPEE_MOUSE	Spermidine synthase OS=Mus musculus OX=10090 GN=Srm PE=1 SV=1	
UBP14_MOUSE	Ubiquitin carboxyl-terminal hydrolase 14 OS=Mus musculus OX=10090 GN=Usp14 PE=1 SV=3	
C1QBP_RAT	Complement component 1 Q subcomponent-binding protein, mitochondrial OS=Rattus norvegicus OX=10116 GN=C1qbp PE=1 SV=2	
COR1A_MOUSE	Coronin-1A OS=Mus musculus OX=10090 GN=Coro1a PE=1 SV=5	
GLYM_MOUSE	Serine hydroxymethyltransferase, mitochondrial OS=Mus musculus OX=10090 GN=Shmt2 PE=1 SV=1	
ILF2_MOUSE	Interleukin enhancer-binding factor 2 OS=Mus musculus OX=10090 GN=Ilf2 PE=1 SV=1	
PRPS1_MOUSE	Ribose-phosphate pyrophosphokinase 1 OS=Mus musculus OX=10090 GN=Prps1 PE=1 SV=4	
RAB1B_MOUSE	Ras-related protein Rab-1B OS=Mus musculus OX=10090 GN=Rab1b PE=1 SV=1	
RAB3A_MOUSE	Ras-related protein Rab-3A OS=Mus musculus OX=10090 GN=Rab3a PE=1 SV=1	
RL30_MOUSE	60S ribosomal protein L30 OS=Mus musculus OX=10090 GN=Rpl30 PE=1 SV=2	
ALG2_MOUSE	Alpha-1,3/1,6-mannosyltransferase ALG2 OS=Mus musculus OX=10090 GN=Alg2 PE=1 SV=2	
COTL1_MOUSE	Coactosin-like protein OS=Mus musculus OX=10090 GN=Cotl1 PE=1 SV=3	
DBNL_MOUSE	Drebrin-like protein OS=Mus musculus OX=10090 GN=Dbnl PE=1 SV=2	
DCE1_MOUSE	Glutamate decarboxylase 1 OS=Mus musculus OX=10090 GN=Gad1 PE=1 SV=2	
IF2A_MOUSE	Eukaryotic translation initiation factor 2 subunit 1 OS=Mus musculus OX=10090 GN=Eif2s1 PE=1 SV=3	
ODPX_MOUSE	Pyruvate dehydrogenase protein X component, mitochondrial OS=Mus musculus OX=10090 GN=Pdhx PE=1 SV=1	
PCBP1_MOUSE	Poly(rC)-binding protein 1 OS=Mus musculus OX=10090 GN=Pcbp1 PE=1 SV=1	
PFKAP_MOUSE	ATP-dependent 6-phosphofructokinase, platelet type OS=Mus musculus OX=10090 GN=Pfkp PE=1 SV=1	
PSA7_MOUSE	Proteasome subunit alpha type-7 OS=Mus musculus OX=10090 GN=Psma7 PE=1 SV=1	
TBB4A_MOUSE	Tubulin beta-4A chain OS=Mus musculus OX=10090 GN=Tubb4a PE=1 SV=3	
VAPA_MOUSE	Vesicle-associated membrane protein-associated protein A OS=Mus musculus OX=10090 GN=Vapa PE=1 SV=2	
VIAAT_MOUSE	Vesicular inhibitory amino acid transporter OS=Mus musculus OX=10090 GN=Slc32a1 PE=1 SV=3	
PUR2_MOUSE	Trifunctional purine biosynthetic protein adenosine-3 OS=Mus musculus OX=10090 GN=Gart PE=1 SV=3	
PPID_MOUSE	Peptidyl-prolyl cis-trans isomerase D OS=Mus musculus OX=10090 GN=Ppid PE=1 SV=3	
ARL8B_MOUSE	ADP-ribosylation factor-like protein 8B OS=Mus musculus OX=10090 GN=Arl8b PE=1 SV=1	
DDB1_MOUSE	DNA damage-binding protein 1 OS=Mus musculus OX=10090 GN=Ddb1 PE=1 SV=2	
EF1D_MOUSE	Elongation factor 1-delta OS=Mus musculus OX=10090 GN=Eef1d PE=1 SV=3	
GLSK_MOUSE	Glutaminase kidney isoform, mitochondrial OS=Mus musculus OX=10090 GN=Gls PE=1 SV=1	
GNAS1_MOUSE	Guanine nucleotide-binding protein G(s) subunit alpha isoforms XLas OS=Mus musculus OX=10090 GN=Gnas PE=1 SV=1	
KIF2A_MOUSE	Kinesin-like protein KIF2A OS=Mus musculus OX=10090 GN=Kif2a PE=1 SV=2	
RHOA_MOUSE	Transforming protein RhoA OS=Mus musculus OX=10090 GN=Rhoa PE=1 SV=1	
SET_MOUSE	Protein SET OS=Mus musculus OX=10090 GN=Set PE=1 SV=1	
LAC1_MOUSE	Ig lambda-1 chain C region OS=Mus musculus OX=10090 PE=1 SV=1	
VINC_MOUSE	Vinculin OS=Mus musculus OX=10090 GN=Vcl PE=1 SV=4	
CSK21_MOUSE	Casein kinase II subunit alpha OS=Mus musculus OX=10090 GN=Csnk2a1 PE=1 SV=2	
BLMH_MOUSE	Bleomycin hydrolase OS=Mus musculus OX=10090 GN=Blmh PE=1 SV=1	
ECHB_MOUSE	Trifunctional enzyme subunit beta, mitochondrial OS=Mus musculus OX=10090 GN=Hadhb PE=1 SV=1	
HNRDL_MOUSE	Heterogeneous nuclear ribonucleoprotein D-like OS=Mus musculus OX=10090 GN=Hnrnpdl PE=1 SV=1	
RL23A_MOUSE	60S ribosomal protein L23a OS=Mus musculus OX=10090 GN=Rpl23a PE=1 SV=1	
RS12_MOUSE	40S ribosomal protein S12 OS=Mus musculus OX=10090 GN=Rps12 PE=1 SV=2	
U2AF2_MOUSE	Splicing factor U2AF 65 kDa subunit OS=Mus musculus OX=10090 GN=U2af2 PE=1 SV=3	
EMC1_MOUSE	ER membrane protein complex subunit 1 OS=Mus musculus OX=10090 GN=Emc1 PE=1 SV=1	
H33_MOUSE	Histone H3.3 OS=Mus musculus OX=10090 GN=H3-3a PE=1 SV=2	
HNRPF_MOUSE	Heterogeneous nuclear ribonucleoprotein F OS=Mus musculus OX=10090 GN=Hnrnpf PE=1 SV=3	
CYBP_MOUSE	Calcyclin-binding protein OS=Mus musculus OX=10090 GN=Cacybp PE=1 SV=1	
ANXA1_MOUSE	Annexin A1 OS=Mus musculus OX=10090 GN=Anxa1 PE=1 SV=2	
COX2_LEGFO	Cytochrome c oxidase subunit 2 OS=Leggadina forresti OX=81935 GN=MT-CO2 PE=3 SV=1	
DNM3A_MOUSE	DNA (cytosine-5)-methyltransferase 3A OS=Mus musculus OX=10090 GN=Dnmt3a PE=1 SV=2	
F10A1_MOUSE	Hsc70-interacting protein OS=Mus musculus OX=10090 GN=St13 PE=1 SV=1	
FA49A_MOUSE	Protein FAM49A OS=Mus musculus OX=10090 GN=Fam49a PE=1 SV=1	
HINT1_MOUSE	Histidine triad nucleotide-binding protein 1 OS=Mus musculus OX=10090 GN=Hint1 PE=1 SV=3	
ITB1_MOUSE	Integrin beta-1 OS=Mus musculus OX=10090 GN=Itgb1 PE=1 SV=1	
JIP3_MOUSE	C-Jun-amino-terminal kinase-interacting protein 3 OS=Mus musculus OX=10090 GN=Mapk8ip3 PE=1 SV=1	
KCC2A_MOUSE	Calcium/calmodulin-dependent protein kinase type II subunit alpha OS=Mus musculus OX=10090 GN=Camk2a PE=1 SV=2	
MYL6_MOUSE	Myosin light polypeptide 6 OS=Mus musculus OX=10090 GN=Myl6 PE=1 SV=3	
NP1L4_MOUSE	Nucleosome assembly protein 1-like 4 OS=Mus musculus OX=10090 GN=Nap1l4 PE=1 SV=1	
ODO2_MOUSE	Dihydrolipoyllysine-residue succinyltransferase component of 2-oxoglutarate dehydrogenase complex, mitochondrial OS=Mus musculus OX=10090 GN=Dlst PE=1 SV=1	
RS5_MOUSE	40S ribosomal protein S5 OS=Mus musculus OX=10090 GN=Rps5 PE=1 SV=3	
RS6_MOUSE	40S ribosomal protein S6 OS=Mus musculus OX=10090 GN=Rps6 PE=1 SV=1	
STT3A_HUMAN	Dolichyl-diphosphooligosaccharide--protein glycosyltransferase subunit STT3A OS=Homo sapiens OX=9606 GN=STT3A PE=1 SV=2	
USO1_MOUSE	General vesicular transport factor p115 OS=Mus musculus OX=10090 GN=Uso1 PE=1 SV=2	
IDHG1_MOUSE	Isocitrate dehydrogenase [NAD] subunit gamma 1, mitochondrial OS=Mus musculus OX=10090 GN=Idh3g PE=1 SV=1	
H12_MOUSE	Histone H1.2 OS=Mus musculus OX=10090 GN=Hist1h1c PE=1 SV=2	
PPIB_MOUSE	Peptidyl-prolyl cis-trans isomerase B OS=Mus musculus OX=10090 GN=Ppib PE=1 SV=2	
PRDX5_MOUSE	Peroxiredoxin-5, mitochondrial OS=Mus musculus OX=10090 GN=Prdx5 PE=1 SV=2	
PSA5_MOUSE	Proteasome subunit alpha type-5 OS=Mus musculus OX=10090 GN=Psma5 PE=1 SV=1	
PUR9_MOUSE	Bifunctional purine biosynthesis protein PURH OS=Mus musculus OX=10090 GN=Atic PE=1 SV=2	
RU17_MOUSE	U1 small nuclear ribonucleoprotein 70 kDa OS=Mus musculus OX=10090 GN=Snrnp70 PE=1 SV=2	
STRAP_MOUSE	Serine-threonine kinase receptor-associated protein OS=Mus musculus OX=10090 GN=Strap PE=1 SV=2	
TEBP_MOUSE	Prostaglandin E synthase 3 OS=Mus musculus OX=10090 GN=Ptges3 PE=1 SV=1	
THIM_MOUSE	3-ketoacyl-CoA thiolase, mitochondrial OS=Mus musculus OX=10090 GN=Acaa2 PE=1 SV=3	
PIPNA_MOUSE	Phosphatidylinositol transfer protein alpha isoform OS=Mus musculus OX=10090 GN=Pitpna PE=1 SV=2	
IMDH2_MOUSE	Inosine-5'-monophosphate dehydrogenase 2 OS=Mus musculus OX=10090 GN=Impdh2 PE=1 SV=2	
NACAM_MOUSE	Nascent polypeptide-associated complex subunit alpha, muscle-specific form OS=Mus musculus OX=10090 GN=Naca PE=1 SV=2	
GSLG1_MOUSE	Golgi apparatus protein 1 OS=Mus musculus OX=10090 GN=Glg1 PE=1 SV=1	
ACDSB_MOUSE	Short/branched chain specific acyl-CoA dehydrogenase, mitochondrial OS=Mus musculus OX=10090 GN=Acadsb PE=1 SV=1	
SYK_MOUSE	Lysine--tRNA ligase OS=Mus musculus OX=10090 GN=Kars1 PE=1 SV=1	
SC6A1_MOUSE	Sodium- and chloride-dependent GABA transporter 1 OS=Mus musculus OX=10090 GN=Slc6a1 PE=1 SV=2	
UBR4_MOUSE	E3 ubiquitin-protein ligase UBR4 OS=Mus musculus OX=10090 GN=Ubr4 PE=1 SV=1	
AINX_MOUSE	Alpha-internexin OS=Mus musculus OX=10090 GN=Ina PE=1 SV=3	
CANB1_MOUSE	Calcineurin subunit B type 1 OS=Mus musculus OX=10090 GN=Ppp3r1 PE=1 SV=3	
GNAI1_MOUSE	Guanine nucleotide-binding protein G(i) subunit alpha-1 OS=Mus musculus OX=10090 GN=Gnai1 PE=1 SV=1	
MBB1A_MOUSE	Myb-binding protein 1A OS=Mus musculus OX=10090 GN=Mybbp1a PE=1 SV=2	
PSD3_MOUSE	PH and SEC7 domain-containing protein 3 OS=Mus musculus OX=10090 GN=Psd3 PE=1 SV=2	
RBBP4_MOUSE	Histone-binding protein RBBP4 OS=Mus musculus OX=10090 GN=Rbbp4 PE=1 SV=5	
SUCA_MOUSE	Succinate--CoA ligase [ADP/GDP-forming] subunit alpha, mitochondrial OS=Mus musculus OX=10090 GN=Suclg1 PE=1 SV=4	
H2AV_MOUSE	Histone H2A.V OS=Mus musculus OX=10090 GN=H2afv PE=1 SV=3	
CKAP5_MOUSE	Cytoskeleton-associated protein 5 OS=Mus musculus OX=10090 GN=Ckap5 PE=1 SV=1	
FHL1_MOUSE	Four and a half LIM domains protein 1 OS=Mus musculus OX=10090 GN=Fhl1 PE=1 SV=3	
LASP1_MOUSE	LIM and SH3 domain protein 1 OS=Mus musculus OX=10090 GN=Lasp1 PE=1 SV=1	
PSA3_MOUSE	Proteasome subunit alpha type-3 OS=Mus musculus OX=10090 GN=Psma3 PE=1 SV=3	
RAC1_MOUSE	Ras-related C3 botulinum toxin substrate 1 OS=Mus musculus OX=10090 GN=Rac1 PE=1 SV=1	
RL23_MOUSE	60S ribosomal protein L23 OS=Mus musculus OX=10090 GN=Rpl23 PE=1 SV=1	
RL8_MOUSE	60S ribosomal protein L8 OS=Mus musculus OX=10090 GN=Rpl8 PE=1 SV=2	
SGTA_MOUSE	Small glutamine-rich tetratricopeptide repeat-containing protein alpha OS=Mus musculus OX=10090 GN=Sgta PE=1 SV=2	
SNAG_MOUSE	Gamma-soluble NSF attachment protein OS=Mus musculus OX=10090 GN=Napg PE=1 SV=1	
TRA2B_MOUSE	Transformer-2 protein homolog beta OS=Mus musculus OX=10090 GN=Tra2b PE=1 SV=1	
VATD_MOUSE	V-type proton ATPase subunit D OS=Mus musculus OX=10090 GN=Atp6v1d PE=1 SV=1	
SYWC_MOUSE	Tryptophan--tRNA ligase, cytoplasmic OS=Mus musculus OX=10090 GN=Wars PE=1 SV=2	
RLA2_MOUSE	60S acidic ribosomal protein P2 OS=Mus musculus OX=10090 GN=Rplp2 PE=1 SV=3	
MIF_MOUSE	Macrophage migration inhibitory factor OS=Mus musculus OX=10090 GN=Mif PE=1 SV=2	
BZW1_MOUSE	Basic leucine zipper and W2 domain-containing protein 1 OS=Mus musculus OX=10090 GN=Bzw1 PE=1 SV=1	
SYFB_MOUSE	Phenylalanine--tRNA ligase beta subunit OS=Mus musculus OX=10090 GN=Farsb PE=1 SV=2	
ARPC3_MOUSE	Actin-related protein 2/3 complex subunit 3 OS=Mus musculus OX=10090 GN=Arpc3 PE=1 SV=3	
CAN2_MOUSE	Calpain-2 catalytic subunit OS=Mus musculus OX=10090 GN=Capn2 PE=1 SV=4	
DCE2_MOUSE	Glutamate decarboxylase 2 OS=Mus musculus OX=10090 GN=Gad2 PE=1 SV=1	
EIF3F_MOUSE	Eukaryotic translation initiation factor 3 subunit F OS=Mus musculus OX=10090 GN=Eif3f PE=1 SV=2	
G3BP2_MOUSE	Ras GTPase-activating protein-binding protein 2 OS=Mus musculus OX=10090 GN=G3bp2 PE=1 SV=2	
NSF1C_MOUSE	NSFL1 cofactor p47 OS=Mus musculus OX=10090 GN=Nsfl1c PE=1 SV=1	
PSA4_MOUSE	Proteasome subunit alpha type-4 OS=Mus musculus OX=10090 GN=Psma4 PE=1 SV=1	
PSB7_MOUSE	Proteasome subunit beta type-7 OS=Mus musculus OX=10090 GN=Psmb7 PE=1 SV=1	
PYRG1_MOUSE	CTP synthase 1 OS=Mus musculus OX=10090 GN=Ctps1 PE=1 SV=2	
TSN_MOUSE	Translin OS=Mus musculus OX=10090 GN=Tsn PE=1 SV=1	
SRC8_MOUSE	Src substrate cortactin OS=Mus musculus OX=10090 GN=Cttn PE=1 SV=2	
SYFA_MOUSE	Phenylalanine--tRNA ligase alpha subunit OS=Mus musculus OX=10090 GN=Farsa PE=1 SV=1	
SYIC_MOUSE	Isoleucine--tRNA ligase, cytoplasmic OS=Mus musculus OX=10090 GN=Iars PE=1 SV=2	
EHD1_MOUSE	EH domain-containing protein 1 OS=Mus musculus OX=10090 GN=Ehd1 PE=1 SV=1	
VIGLN_MOUSE	Vigilin OS=Mus musculus OX=10090 GN=Hdlbp PE=1 SV=1	
UBE2N_MOUSE	Ubiquitin-conjugating enzyme E2 N OS=Mus musculus OX=10090 GN=Ube2n PE=1 SV=1	
IF4A2_MOUSE	Eukaryotic initiation factor 4A-II OS=Mus musculus OX=10090 GN=Eif4a2 PE=1 SV=2	
CHD4_MOUSE	Chromodomain-helicase-DNA-binding protein 4 OS=Mus musculus OX=10090 GN=Chd4 PE=1 SV=1	
CALU_MOUSE	Calumenin OS=Mus musculus OX=10090 GN=Calu PE=1 SV=1	
CUL3_MOUSE	Cullin-3 OS=Mus musculus OX=10090 GN=Cul3 PE=1 SV=1	
EIF3L_MOUSE	Eukaryotic translation initiation factor 3 subunit L OS=Mus musculus OX=10090 GN=Eif3l PE=1 SV=1	
ELAV1_MOUSE	ELAV-like protein 1 OS=Mus musculus OX=10090 GN=Elavl1 PE=1 SV=2	
PAIRB_MOUSE	Plasminogen activator inhibitor 1 RNA-binding protein OS=Mus musculus OX=10090 GN=Serbp1 PE=1 SV=2	
RAP1B_MOUSE	Ras-related protein Rap-1b OS=Mus musculus OX=10090 GN=Rap1b PE=1 SV=2	
RL11_MOUSE	60S ribosomal protein L11 OS=Mus musculus OX=10090 GN=Rpl11 PE=1 SV=4	
RS16_MOUSE	40S ribosomal protein S16 OS=Mus musculus OX=10090 GN=Rps16 PE=1 SV=4	
SDHB_MOUSE	Succinate dehydrogenase [ubiquinone] iron-sulfur subunit, mitochondrial OS=Mus musculus OX=10090 GN=Sdhb PE=1 SV=1	
STX1A_MOUSE	Syntaxin-1A OS=Mus musculus OX=10090 GN=Stx1a PE=1 SV=3	
TALDO_MOUSE	Transaldolase OS=Mus musculus OX=10090 GN=Taldo1 PE=1 SV=2	
TXNL1_MOUSE	Thioredoxin-like protein 1 OS=Mus musculus OX=10090 GN=Txnl1 PE=1 SV=3	
PSPC1_MOUSE	Paraspeckle component 1 OS=Mus musculus OX=10090 GN=Pspc1 PE=1 SV=1	
FARP1_MOUSE	FERM, ARHGEF and pleckstrin domain-containing protein 1 OS=Mus musculus OX=10090 GN=Farp1 PE=1 SV=1	
S61A1_MOUSE	Protein transport protein Sec61 subunit alpha isoform 1 OS=Mus musculus OX=10090 GN=Sec61a1 PE=1 SV=2	
UGGG1_MOUSE	UDP-glucose:glycoprotein glucosyltransferase 1 OS=Mus musculus OX=10090 GN=Uggt1 PE=1 SV=4	
GLOD4_MOUSE	Glyoxalase domain-containing protein 4 OS=Mus musculus OX=10090 GN=Glod4 PE=1 SV=1	
DPP3_MOUSE	Dipeptidyl peptidase 3 OS=Mus musculus OX=10090 GN=Dpp3 PE=1 SV=2	
SKP1_HUMAN	S-phase kinase-associated protein 1 OS=Homo sapiens OX=9606 GN=SKP1 PE=1 SV=2	
KAD3_MOUSE	GTP:AMP phosphotransferase AK3, mitochondrial OS=Mus musculus OX=10090 GN=Ak3 PE=1 SV=3	
KPCA_MOUSE	Protein kinase C alpha type OS=Mus musculus OX=10090 GN=Prkca PE=1 SV=3	
ITAV_MOUSE	Integrin alpha-V OS=Mus musculus OX=10090 GN=Itgav PE=1 SV=2	
ADDB_MOUSE	Beta-adducin OS=Mus musculus OX=10090 GN=Add2 PE=1 SV=4	
CNDP2_MOUSE	Cytosolic non-specific dipeptidase OS=Mus musculus OX=10090 GN=Cndp2 PE=1 SV=1	
CON__P02533	CON__P02533	
H10_MOUSE	Histone H1.0 OS=Mus musculus OX=10090 GN=H1f0 PE=2 SV=4	
KCRU_MOUSE	Creatine kinase U-type, mitochondrial OS=Mus musculus OX=10090 GN=Ckmt1 PE=1 SV=1	
LAT1_MOUSE	Large neutral amino acids transporter small subunit 1 OS=Mus musculus OX=10090 GN=Slc7a5 PE=1 SV=2	
EF1B_MOUSE	Elongation factor 1-beta OS=Mus musculus OX=10090 GN=Eef1b PE=1 SV=5	
TCTP_MOUSE	Translationally-controlled tumor protein OS=Mus musculus OX=10090 GN=Tpt1 PE=1 SV=1	
TNPO2_MOUSE	Transportin-2 OS=Mus musculus OX=10090 GN=Tnpo2 PE=1 SV=1	
OGA_MOUSE	Protein O-GlcNAcase OS=Mus musculus OX=10090 GN=Oga PE=1 SV=2	
AK1A1_MOUSE	Aldo-keto reductase family 1 member A1 OS=Mus musculus OX=10090 GN=Akr1a1 PE=1 SV=3	
APEX1_MOUSE	DNA-(apurinic or apyrimidinic site) lyase OS=Mus musculus OX=10090 GN=Apex1 PE=1 SV=2	
ASNA_MOUSE	ATPase Asna1 OS=Mus musculus OX=10090 GN=Asna1 PE=1 SV=2	
LONM_MOUSE	Lon protease homolog, mitochondrial OS=Mus musculus OX=10090 GN=Lonp1 PE=1 SV=2	
LRC59_MOUSE	Leucine-rich repeat-containing protein 59 OS=Mus musculus OX=10090 GN=Lrrc59 PE=1 SV=1	
PLPR4_MOUSE	Phospholipid phosphatase-related protein type 4 OS=Mus musculus OX=10090 GN=Plppr4 PE=1 SV=2	
PSA2_MOUSE	Proteasome subunit alpha type-2 OS=Mus musculus OX=10090 GN=Psma2 PE=1 SV=3	
PTN11_MOUSE	Tyrosine-protein phosphatase non-receptor type 11 OS=Mus musculus OX=10090 GN=Ptpn11 PE=1 SV=2	
RS19_MOUSE	40S ribosomal protein S19 OS=Mus musculus OX=10090 GN=Rps19 PE=1 SV=3	
SNP47_MOUSE	Synaptosomal-associated protein 47 OS=Mus musculus OX=10090 GN=Snap47 PE=1 SV=1	
TR150_MOUSE	Thyroid hormone receptor-associated protein 3 OS=Mus musculus OX=10090 GN=Thrap3 PE=1 SV=1	
THIKA_MOUSE	3-ketoacyl-CoA thiolase A, peroxisomal OS=Mus musculus OX=10090 GN=Acaa1a PE=1 SV=1	
VP26B_MOUSE	Vacuolar protein sorting-associated protein 26B OS=Mus musculus OX=10090 GN=Vps26b PE=1 SV=1	
AP1G1_MOUSE	AP-1 complex subunit gamma-1 OS=Mus musculus OX=10090 GN=Ap1g1 PE=1 SV=3	
FUBP1_MOUSE	Far upstream element-binding protein 1 OS=Mus musculus OX=10090 GN=Fubp1 PE=1 SV=1	
APLP2_HUMAN	Amyloid-like protein 2 OS=Homo sapiens OX=9606 GN=APLP2 PE=1 SV=2	
PSME1_MOUSE	Proteasome activator complex subunit 1 OS=Mus musculus OX=10090 GN=Psme1 PE=1 SV=2	
MY18A_MOUSE	Unconventional myosin-XVIIIa OS=Mus musculus OX=10090 GN=Myo18a PE=1 SV=2	
HUWE1_MOUSE	E3 ubiquitin-protein ligase HUWE1 OS=Mus musculus OX=10090 GN=Huwe1 PE=1 SV=5	
PLXA4_MOUSE	Plexin-A4 OS=Mus musculus OX=10090 GN=Plxna4 PE=1 SV=3	
CH10_MOUSE	10 kDa heat shock protein, mitochondrial OS=Mus musculus OX=10090 GN=Hspe1 PE=1 SV=2	
HNRLL_MOUSE	Heterogeneous nuclear ribonucleoprotein L-like OS=Mus musculus OX=10090 GN=Hnrnpll PE=1 SV=3	
LEG1_MOUSE	Galectin-1 OS=Mus musculus OX=10090 GN=Lgals1 PE=1 SV=3	
MTPN_MOUSE	Myotrophin OS=Mus musculus OX=10090 GN=Mtpn PE=1 SV=2	
NPTN_MOUSE	Neuroplastin OS=Mus musculus OX=10090 GN=Nptn PE=1 SV=3	
QCR7_MOUSE	Cytochrome b-c1 complex subunit 7 OS=Mus musculus OX=10090 GN=Uqcrb PE=1 SV=3	
RANG_MOUSE	Ran-specific GTPase-activating protein OS=Mus musculus OX=10090 GN=Ranbp1 PE=1 SV=2	
RL18_MOUSE	60S ribosomal protein L18 OS=Mus musculus OX=10090 GN=Rpl18 PE=1 SV=3	
STML2_MOUSE	Stomatin-like protein 2, mitochondrial OS=Mus musculus OX=10090 GN=Stoml2 PE=1 SV=1	
EPMIP_MOUSE	EPM2A-interacting protein 1 OS=Mus musculus OX=10090 GN=Epm2aip1 PE=1 SV=1	
CSN1_MOUSE	COP9 signalosome complex subunit 1 OS=Mus musculus OX=10090 GN=Gps1 PE=1 SV=1	
ELAV3_MOUSE	ELAV-like protein 3 OS=Mus musculus OX=10090 GN=Elavl3 PE=1 SV=1	
PARK7_MOUSE	Protein/nucleic acid deglycase DJ-1 OS=Mus musculus OX=10090 GN=Park7 PE=1 SV=1	
COR1B_MOUSE	Coronin-1B OS=Mus musculus OX=10090 GN=Coro1b PE=1 SV=1	
G6PD1_MOUSE	Glucose-6-phosphate 1-dehydrogenase X OS=Mus musculus OX=10090 GN=G6pdx PE=1 SV=3	
LETM1_MOUSE	Mitochondrial proton/calcium exchanger protein OS=Mus musculus OX=10090 GN=Letm1 PE=1 SV=1	
CA2D1_MOUSE	Voltage-dependent calcium channel subunit alpha-2/delta-1 OS=Mus musculus OX=10090 GN=Cacna2d1 PE=1 SV=1	
PLEC_MOUSE	Plectin OS=Mus musculus OX=10090 GN=Plec PE=1 SV=3	
ADHX_MOUSE	Alcohol dehydrogenase class-3 OS=Mus musculus OX=10090 GN=Adh5 PE=1 SV=3	
AN32E_MOUSE	Acidic leucine-rich nuclear phosphoprotein 32 family member E OS=Mus musculus OX=10090 GN=Anp32e PE=1 SV=2	
COPD_MOUSE	Coatomer subunit delta OS=Mus musculus OX=10090 GN=Arcn1 PE=1 SV=2	
IF2B_MOUSE	Eukaryotic translation initiation factor 2 subunit 2 OS=Mus musculus OX=10090 GN=Eif2s2 PE=1 SV=1	
MP2K1_MOUSE	Dual specificity mitogen-activated protein kinase kinase 1 OS=Mus musculus OX=10090 GN=Map2k1 PE=1 SV=2	
PLMN_MOUSE	Plasminogen OS=Mus musculus OX=10090 GN=Plg PE=1 SV=3	
PROF2_MOUSE	Profilin-2 OS=Mus musculus OX=10090 GN=Pfn2 PE=1 SV=3	
RL10_MOUSE	60S ribosomal protein L10 OS=Mus musculus OX=10090 GN=Rpl10 PE=1 SV=3	
ROAA_MOUSE	Heterogeneous nuclear ribonucleoprotein A/B OS=Mus musculus OX=10090 GN=Hnrnpab PE=1 SV=1	
SNX3_MOUSE	Sorting nexin-3 OS=Mus musculus OX=10090 GN=Snx3 PE=1 SV=3	
TPM3_MOUSE	Tropomyosin alpha-3 chain OS=Mus musculus OX=10090 GN=Tpm3 PE=1 SV=3	
WDR7_MOUSE	WD repeat-containing protein 7 OS=Mus musculus OX=10090 GN=Wdr7 PE=1 SV=3	
IVD_MOUSE	Isovaleryl-CoA dehydrogenase, mitochondrial OS=Mus musculus OX=10090 GN=Ivd PE=1 SV=1	
RENT1_MOUSE	Regulator of nonsense transcripts 1 OS=Mus musculus OX=10090 GN=Upf1 PE=1 SV=2	
OLA1_MOUSE	Obg-like ATPase 1 OS=Mus musculus OX=10090 GN=Ola1 PE=1 SV=1	
SRSF3_MOUSE	Serine/arginine-rich splicing factor 3 OS=Mus musculus OX=10090 GN=Srsf3 PE=1 SV=1	
UBQL2_MOUSE	Ubiquilin-2 OS=Mus musculus OX=10090 GN=Ubqln2 PE=1 SV=2	
CSN4_MOUSE	COP9 signalosome complex subunit 4 OS=Mus musculus OX=10090 GN=Cops4 PE=1 SV=1	
RL21_MOUSE	60S ribosomal protein L21 OS=Mus musculus OX=10090 GN=Rpl21 PE=1 SV=3	
MLEC_MOUSE	Malectin OS=Mus musculus OX=10090 GN=Mlec PE=1 SV=2	
CSDE1_MOUSE	Cold shock domain-containing protein E1 OS=Mus musculus OX=10090 GN=Csde1 PE=1 SV=1	
2ABA_MOUSE	Serine/threonine-protein phosphatase 2A 55 kDa regulatory subunit B alpha isoform OS=Mus musculus OX=10090 GN=Ppp2r2a PE=1 SV=1	
HPRT_MOUSE	Hypoxanthine-guanine phosphoribosyltransferase OS=Mus musculus OX=10090 GN=Hprt1 PE=1 SV=3	
HVM51_MOUSE	Ig heavy chain V region AC38 205.12 OS=Mus musculus OX=10090 PE=1 SV=1	
NDUS3_MOUSE	NADH dehydrogenase [ubiquinone] iron-sulfur protein 3, mitochondrial OS=Mus musculus OX=10090 GN=Ndufs3 PE=1 SV=2	
NDUS7_MOUSE	NADH dehydrogenase [ubiquinone] iron-sulfur protein 7, mitochondrial OS=Mus musculus OX=10090 GN=Ndufs7 PE=1 SV=1	
RS13_MOUSE	40S ribosomal protein S13 OS=Mus musculus OX=10090 GN=Rps13 PE=1 SV=2	
NEB2_MOUSE	Neurabin-2 OS=Mus musculus OX=10090 GN=Ppp1r9b PE=1 SV=1	
RLA1_MOUSE	60S acidic ribosomal protein P1 OS=Mus musculus OX=10090 GN=Rplp1 PE=1 SV=1	
NEUL_MOUSE	Neurolysin, mitochondrial OS=Mus musculus OX=10090 GN=Nln PE=1 SV=1	
SEPT3_MOUSE	Neuronal-specific septin-3 OS=Mus musculus OX=10090 GN=Septin3 PE=1 SV=2	
SCAM1_MOUSE	Secretory carrier-associated membrane protein 1 OS=Mus musculus OX=10090 GN=Scamp1 PE=1 SV=1	
NCPR_MOUSE	NADPH--cytochrome P450 reductase OS=Mus musculus OX=10090 GN=Por PE=1 SV=2	
CO4B_MOUSE	Complement C4-B OS=Mus musculus OX=10090 GN=C4b PE=1 SV=3	
DNJA2_MOUSE	DnaJ homolog subfamily A member 2 OS=Mus musculus OX=10090 GN=Dnaja2 PE=1 SV=1	
ADK_MOUSE	Adenosine kinase OS=Mus musculus OX=10090 GN=Adk PE=1 SV=2	
TP53B_MOUSE	TP53-binding protein 1 OS=Mus musculus OX=10090 GN=Tp53bp1 PE=1 SV=3	
CLIP2_MOUSE	CAP-Gly domain-containing linker protein 2 OS=Mus musculus OX=10090 GN=Clip2 PE=1 SV=2	
AGK_MOUSE	Acylglycerol kinase, mitochondrial OS=Mus musculus OX=10090 GN=Agk PE=1 SV=1	
SYMC_MOUSE	Methionine--tRNA ligase, cytoplasmic OS=Mus musculus OX=10090 GN=Mars PE=1 SV=1	
SGPL1_MOUSE	Sphingosine-1-phosphate lyase 1 OS=Mus musculus OX=10090 GN=Sgpl1 PE=1 SV=1	
ANXA7_MOUSE	Annexin A7 OS=Mus musculus OX=10090 GN=Anxa7 PE=1 SV=2	
CNBP_MOUSE	Cellular nucleic acid-binding protein OS=Mus musculus OX=10090 GN=Cnbp PE=1 SV=2	
GSTM5_MOUSE	Glutathione S-transferase Mu 5 OS=Mus musculus OX=10090 GN=Gstm5 PE=1 SV=1	
RL27A_MOUSE	60S ribosomal protein L27a OS=Mus musculus OX=10090 GN=Rpl27a PE=1 SV=5	
SH3G2_MOUSE	Endophilin-A1 OS=Mus musculus OX=10090 GN=Sh3gl2 PE=1 SV=2	
NEUA_MOUSE	N-acylneuraminate cytidylyltransferase OS=Mus musculus OX=10090 GN=Cmas PE=1 SV=2	
AAK1_MOUSE	AP2-associated protein kinase 1 OS=Mus musculus OX=10090 GN=Aak1 PE=1 SV=2	
MGST3_MOUSE	Microsomal glutathione S-transferase 3 OS=Mus musculus OX=10090 GN=Mgst3 PE=1 SV=1	
SC31A_MOUSE	Protein transport protein Sec31A OS=Mus musculus OX=10090 GN=Sec31a PE=1 SV=2	
NDUBA_MOUSE	NADH dehydrogenase [ubiquinone] 1 beta subcomplex subunit 10 OS=Mus musculus OX=10090 GN=Ndufb10 PE=1 SV=3	
NEDD4_MOUSE	E3 ubiquitin-protein ligase NEDD4 OS=Mus musculus OX=10090 GN=Nedd4 PE=1 SV=3	
ASGL1_MOUSE	Isoaspartyl peptidase/L-asparaginase OS=Mus musculus OX=10090 GN=Asrgl1 PE=1 SV=1	
RTN3_MOUSE	Reticulon-3 OS=Mus musculus OX=10090 GN=Rtn3 PE=1 SV=2	
DHRS1_MOUSE	Dehydrogenase/reductase SDR family member 1 OS=Mus musculus OX=10090 GN=Dhrs1 PE=1 SV=1	
NP1L1_MOUSE	Nucleosome assembly protein 1-like 1 OS=Mus musculus OX=10090 GN=Nap1l1 PE=1 SV=2	
ABD12_MOUSE	Lysophosphatidylserine lipase ABHD12 OS=Mus musculus OX=10090 GN=Abhd12 PE=1 SV=2	
SMRC2_MOUSE	SWI/SNF complex subunit SMARCC2 OS=Mus musculus OX=10090 GN=Smarcc2 PE=1 SV=2	
ETFD_MOUSE	Electron transfer flavoprotein-ubiquinone oxidoreductase, mitochondrial OS=Mus musculus OX=10090 GN=Etfdh PE=1 SV=1	
PALM_MOUSE	Paralemmin-1 OS=Mus musculus OX=10090 GN=Palm PE=1 SV=1	
LANC2_MOUSE	LanC-like protein 2 OS=Mus musculus OX=10090 GN=Lancl2 PE=1 SV=1	
CLCB_MOUSE	Clathrin light chain B OS=Mus musculus OX=10090 GN=Cltb PE=1 SV=1	
COX5A_MOUSE	Cytochrome c oxidase subunit 5A, mitochondrial OS=Mus musculus OX=10090 GN=Cox5a PE=1 SV=2	
GLU2B_MOUSE	Glucosidase 2 subunit beta OS=Mus musculus OX=10090 GN=Prkcsh PE=1 SV=1	
LAP2B_MOUSE	Lamina-associated polypeptide 2, isoforms beta/delta/epsilon/gamma OS=Mus musculus OX=10090 GN=Tmpo PE=1 SV=4	
NIPS1_MOUSE	Protein NipSnap homolog 1 OS=Mus musculus OX=10090 GN=Nipsnap1 PE=1 SV=1	
PLPP_MOUSE	Pyridoxal phosphate phosphatase OS=Mus musculus OX=10090 GN=Pdxp PE=1 SV=1	
PSB4_MOUSE	Proteasome subunit beta type-4 OS=Mus musculus OX=10090 GN=Psmb4 PE=1 SV=1	
RAB18_MOUSE	Ras-related protein Rab-18 OS=Mus musculus OX=10090 GN=Rab18 PE=1 SV=2	
RL34_MOUSE	60S ribosomal protein L34 OS=Mus musculus OX=10090 GN=Rpl34 PE=1 SV=2	
RS9_MOUSE	40S ribosomal protein S9 OS=Mus musculus OX=10090 GN=Rps9 PE=1 SV=3	
SP16H_MOUSE	FACT complex subunit SPT16 OS=Mus musculus OX=10090 GN=Supt16h PE=1 SV=2	
LY6H_MOUSE	Lymphocyte antigen 6H OS=Mus musculus OX=10090 GN=Ly6h PE=1 SV=2	
SC22B_MOUSE	Vesicle-trafficking protein SEC22b OS=Mus musculus OX=10090 GN=Sec22b PE=1 SV=3	
AP1B1_MOUSE	AP-1 complex subunit beta-1 OS=Mus musculus OX=10090 GN=Ap1b1 PE=1 SV=2	
6PGL_MOUSE	6-phosphogluconolactonase OS=Mus musculus OX=10090 GN=Pgls PE=1 SV=1	
CENPV_MOUSE	Centromere protein V OS=Mus musculus OX=10090 GN=Cenpv PE=1 SV=2	
SNAA_MOUSE	Alpha-soluble NSF attachment protein OS=Mus musculus OX=10090 GN=Napa PE=1 SV=1	
AHSA1_MOUSE	Activator of 90 kDa heat shock protein ATPase homolog 1 OS=Mus musculus OX=10090 GN=Ahsa1 PE=1 SV=2	
GLNA_MOUSE	Glutamine synthetase OS=Mus musculus OX=10090 GN=Glul PE=1 SV=6	
TMED2_MOUSE	Transmembrane emp24 domain-containing protein 2 OS=Mus musculus OX=10090 GN=Tmed2 PE=1 SV=1	
GLRX3_MOUSE	Glutaredoxin-3 OS=Mus musculus OX=10090 GN=Glrx3 PE=1 SV=1	
PA1B2_MOUSE	Platelet-activating factor acetylhydrolase IB subunit beta OS=Mus musculus OX=10090 GN=Pafah1b2 PE=1 SV=2	
CLPT1_MOUSE	Cleft lip and palate transmembrane protein 1 homolog OS=Mus musculus OX=10090 GN=Clptm1 PE=1 SV=1	
E41L2_MOUSE	Band 4.1-like protein 2 OS=Mus musculus OX=10090 GN=Epb41l2 PE=1 SV=2	
HSDL1_MOUSE	Inactive hydroxysteroid dehydrogenase-like protein 1 OS=Mus musculus OX=10090 GN=Hsdl1 PE=1 SV=1	
NCLN_MOUSE	Nicalin OS=Mus musculus OX=10090 GN=Ncln PE=1 SV=2	
NRX1A_MOUSE	Neurexin-1 OS=Mus musculus OX=10090 GN=Nrxn1 PE=1 SV=3	
ROA0_MOUSE	Heterogeneous nuclear ribonucleoprotein A0 OS=Mus musculus OX=10090 GN=Hnrnpa0 PE=1 SV=1	
MPP2_MOUSE	MAGUK p55 subfamily member 2 OS=Mus musculus OX=10090 GN=Mpp2 PE=1 SV=1	
SRSF2_MOUSE	Serine/arginine-rich splicing factor 2 OS=Mus musculus OX=10090 GN=Srsf2 PE=1 SV=4	
NT5D3_MOUSE	5'-nucleotidase domain-containing protein 3 OS=Mus musculus OX=10090 GN=Nt5dc3 PE=1 SV=1	
PYR1_MOUSE	CAD protein OS=Mus musculus OX=10090 GN=Cad PE=1 SV=1	
A2ASS6-DECOY	A2ASS6	
BASI_MOUSE	Basigin OS=Mus musculus OX=10090 GN=Bsg PE=1 SV=2	
EIF3D_MOUSE	Eukaryotic translation initiation factor 3 subunit D OS=Mus musculus OX=10090 GN=Eif3d PE=1 SV=2	
FA49B_MOUSE	Protein FAM49B OS=Mus musculus OX=10090 GN=Fam49b PE=1 SV=1	
GCYB1_MOUSE	Guanylate cyclase soluble subunit beta-1 OS=Mus musculus OX=10090 GN=Gucy1b1 PE=1 SV=1	
PPR1B_MOUSE	Protein phosphatase 1 regulatory subunit 1B OS=Mus musculus OX=10090 GN=Ppp1r1b PE=1 SV=2	
PSMD7_MOUSE	26S proteasome non-ATPase regulatory subunit 7 OS=Mus musculus OX=10090 GN=Psmd7 PE=1 SV=2	
RL14_MOUSE	60S ribosomal protein L14 OS=Mus musculus OX=10090 GN=Rpl14 PE=1 SV=3	
SYUA_MOUSE	Alpha-synuclein OS=Mus musculus OX=10090 GN=Snca PE=1 SV=2	
THIO_MOUSE	Thioredoxin OS=Mus musculus OX=10090 GN=Txn PE=1 SV=3	
NUDC_MOUSE	Nuclear migration protein nudC OS=Mus musculus OX=10090 GN=Nudc PE=1 SV=1	
ABCD3_MOUSE	ATP-binding cassette sub-family D member 3 OS=Mus musculus OX=10090 GN=Abcd3 PE=1 SV=2	
PP1R7_MOUSE	Protein phosphatase 1 regulatory subunit 7 OS=Mus musculus OX=10090 GN=Ppp1r7 PE=1 SV=2	
SATT_MOUSE	Neutral amino acid transporter A OS=Mus musculus OX=10090 GN=Slc1a4 PE=1 SV=1	
UB2V1_MOUSE	Ubiquitin-conjugating enzyme E2 variant 1 OS=Mus musculus OX=10090 GN=Ube2v1 PE=1 SV=1	
PA1B3_MOUSE	Platelet-activating factor acetylhydrolase IB subunit gamma OS=Mus musculus OX=10090 GN=Pafah1b3 PE=1 SV=1	
SEPT5_MOUSE	Septin-5 OS=Mus musculus OX=10090 GN=Septin5 PE=1 SV=2	
ATAD1_MOUSE	ATPase family AAA domain-containing protein 1 OS=Mus musculus OX=10090 GN=Atad1 PE=1 SV=1	
NLTP_MOUSE	Non-specific lipid-transfer protein OS=Mus musculus OX=10090 GN=Scp2 PE=1 SV=3	
HS71A_MOUSE	Heat shock 70 kDa protein 1A OS=Mus musculus OX=10090 GN=Hspa1a PE=1 SV=2	
TRFE_MOUSE	Serotransferrin OS=Mus musculus OX=10090 GN=Tf PE=1 SV=1	
GEPH_MOUSE	Gephyrin OS=Mus musculus OX=10090 GN=Gphn PE=1 SV=2	
SAE2_MOUSE	SUMO-activating enzyme subunit 2 OS=Mus musculus OX=10090 GN=Uba2 PE=1 SV=1	
MECP2_MOUSE	Methyl-CpG-binding protein 2 OS=Mus musculus OX=10090 GN=Mecp2 PE=1 SV=1	
ACADL_MOUSE	Long-chain specific acyl-CoA dehydrogenase, mitochondrial OS=Mus musculus OX=10090 GN=Acadl PE=1 SV=2	
DHPR_MOUSE	Dihydropteridine reductase OS=Mus musculus OX=10090 GN=Qdpr PE=1 SV=2	
RL13_MOUSE	60S ribosomal protein L13 OS=Mus musculus OX=10090 GN=Rpl13 PE=1 SV=3	
RL17_MOUSE	60S ribosomal protein L17 OS=Mus musculus OX=10090 GN=Rpl17 PE=1 SV=3	
SRSF7_MOUSE	Serine/arginine-rich splicing factor 7 OS=Mus musculus OX=10090 GN=Srsf7 PE=1 SV=1	
TICN3_MOUSE	Testican-3 OS=Mus musculus OX=10090 GN=Spock3 PE=2 SV=2	
VAMP2_MOUSE	Vesicle-associated membrane protein 2 OS=Mus musculus OX=10090 GN=Vamp2 PE=1 SV=2	
CBX5_MOUSE	Chromobox protein homolog 5 OS=Mus musculus OX=10090 GN=Cbx5 PE=1 SV=1	
KV5AB_MOUSE	Ig kappa chain V-V region HP R16.7 OS=Mus musculus OX=10090 PE=1 SV=1	
SMCA5_MOUSE	SWI/SNF-related matrix-associated actin-dependent regulator of chromatin subfamily A member 5 OS=Mus musculus OX=10090 GN=Smarca5 PE=1 SV=1	
GSK3B_MOUSE	Glycogen synthase kinase-3 beta OS=Mus musculus OX=10090 GN=Gsk3b PE=1 SV=2	
RS17_MOUSE	40S ribosomal protein S17 OS=Mus musculus OX=10090 GN=Rps17 PE=1 SV=2	
ATP5L_MOUSE	ATP synthase subunit g, mitochondrial OS=Mus musculus OX=10090 GN=Atp5mg PE=1 SV=1	
CRIP2_MOUSE	Cysteine-rich protein 2 OS=Mus musculus OX=10090 GN=Crip2 PE=1 SV=1	
TPM1_MOUSE	Tropomyosin alpha-1 chain OS=Mus musculus OX=10090 GN=Tpm1 PE=1 SV=1	
IPO4_MOUSE	Importin-4 OS=Mus musculus OX=10090 GN=Ipo4 PE=1 SV=1	
ERP29_MOUSE	Endoplasmic reticulum resident protein 29 OS=Mus musculus OX=10090 GN=Erp29 PE=1 SV=2	
GTF2I_MOUSE	General transcription factor II-I OS=Mus musculus OX=10090 GN=Gtf2i PE=1 SV=3	
STX7_MOUSE	Syntaxin-7 OS=Mus musculus OX=10090 GN=Stx7 PE=1 SV=3	
SRC_MOUSE	Neuronal proto-oncogene tyrosine-protein kinase Src OS=Mus musculus OX=10090 GN=Src PE=1 SV=4	
AMPL_MOUSE	Cytosol aminopeptidase OS=Mus musculus OX=10090 GN=Lap3 PE=1 SV=3	
XPO5_MOUSE	Exportin-5 OS=Mus musculus OX=10090 GN=Xpo5 PE=1 SV=1	
2A5E_MOUSE	Serine/threonine-protein phosphatase 2A 56 kDa regulatory subunit epsilon isoform OS=Mus musculus OX=10090 GN=Ppp2r5e PE=1 SV=3	
NCAN_MOUSE	Neurocan core protein OS=Mus musculus OX=10090 GN=Ncan PE=1 SV=1	
TM1L2_MOUSE	TOM1-like protein 2 OS=Mus musculus OX=10090 GN=Tom1l2 PE=1 SV=1	
NAA15_MOUSE	N-alpha-acetyltransferase 15, NatA auxiliary subunit OS=Mus musculus OX=10090 GN=Naa15 PE=1 SV=1	
DMXL2_MOUSE	DmX-like protein 2 OS=Mus musculus OX=10090 GN=Dmxl2 PE=1 SV=3	
PLXB2_MOUSE	Plexin-B2 OS=Mus musculus OX=10090 GN=Plxnb2 PE=1 SV=1	
CUL2_MOUSE	Cullin-2 OS=Mus musculus OX=10090 GN=Cul2 PE=1 SV=2	
GBG2_MOUSE	Guanine nucleotide-binding protein G(I)/G(S)/G(O) subunit gamma-2 OS=Mus musculus OX=10090 GN=Gng2 PE=1 SV=2	
NDUA4_MOUSE	Cytochrome c oxidase subunit NDUFA4 OS=Mus musculus OX=10090 GN=Ndufa4 PE=1 SV=2	
RL13A_MOUSE	60S ribosomal protein L13a OS=Mus musculus OX=10090 GN=Rpl13a PE=1 SV=4	
TMEDA_MOUSE	Transmembrane emp24 domain-containing protein 10 OS=Mus musculus OX=10090 GN=Tmed10 PE=1 SV=1	
UBC12_MOUSE	NEDD8-conjugating enzyme Ubc12 OS=Mus musculus OX=10090 GN=Ube2m PE=1 SV=1	
CDK5_MOUSE	Cyclin-dependent-like kinase 5 OS=Mus musculus OX=10090 GN=Cdk5 PE=1 SV=1	
DNPEP_MOUSE	Aspartyl aminopeptidase OS=Mus musculus OX=10090 GN=Dnpep PE=1 SV=2	
ML12B_MOUSE	Myosin regulatory light chain 12B OS=Mus musculus OX=10090 GN=Myl12b PE=1 SV=2	
SAFB1_MOUSE	Scaffold attachment factor B1 OS=Mus musculus OX=10090 GN=Safb PE=1 SV=2	
CEND_MOUSE	Cell cycle exit and neuronal differentiation protein 1 OS=Mus musculus OX=10090 GN=Cend1 PE=1 SV=1	
RL38_MOUSE	60S ribosomal protein L38 OS=Mus musculus OX=10090 GN=Rpl38 PE=1 SV=3	
TAGL2_MOUSE	Transgelin-2 OS=Mus musculus OX=10090 GN=Tagln2 PE=1 SV=4	
RL18A_MOUSE	60S ribosomal protein L18a OS=Mus musculus OX=10090 GN=Rpl18a PE=1 SV=1	
AT1B2_MOUSE	Sodium/potassium-transporting ATPase subunit beta-2 OS=Mus musculus OX=10090 GN=Atp1b2 PE=1 SV=2	
NDUB9_MOUSE	NADH dehydrogenase [ubiquinone] 1 beta subcomplex subunit 9 OS=Mus musculus OX=10090 GN=Ndufb9 PE=1 SV=3	
RL24_MOUSE	60S ribosomal protein L24 OS=Mus musculus OX=10090 GN=Rpl24 PE=1 SV=2	
TMOD2_MOUSE	Tropomodulin-2 OS=Mus musculus OX=10090 GN=Tmod2 PE=1 SV=2	
ERGI1_MOUSE	Endoplasmic reticulum-Golgi intermediate compartment protein 1 OS=Mus musculus OX=10090 GN=Ergic1 PE=1 SV=1	
PSB3_MOUSE	Proteasome subunit beta type-3 OS=Mus musculus OX=10090 GN=Psmb3 PE=1 SV=1	
AACS_MOUSE	Acetoacetyl-CoA synthetase OS=Mus musculus OX=10090 GN=Aacs PE=1 SV=1	
PLAP_MOUSE	Phospholipase A-2-activating protein OS=Mus musculus OX=10090 GN=Plaa PE=1 SV=4	
NIPS2_MOUSE	Protein NipSnap homolog 2 OS=Mus musculus OX=10090 GN=Nipsnap2 PE=1 SV=1	
KIF1A_MOUSE	Kinesin-like protein KIF1A OS=Mus musculus OX=10090 GN=Kif1a PE=1 SV=2	
PHP14_MOUSE	14 kDa phosphohistidine phosphatase OS=Mus musculus OX=10090 GN=Phpt1 PE=1 SV=1	
ITSN1_MOUSE	Intersectin-1 OS=Mus musculus OX=10090 GN=Itsn1 PE=1 SV=2	
P5CR2_MOUSE	Pyrroline-5-carboxylate reductase 2 OS=Mus musculus OX=10090 GN=Pycr2 PE=1 SV=1	
SRSF6_MOUSE	Serine/arginine-rich splicing factor 6 OS=Mus musculus OX=10090 GN=Srsf6 PE=1 SV=1	
ASSY_MOUSE	Argininosuccinate synthase OS=Mus musculus OX=10090 GN=Ass1 PE=1 SV=1	
DNJC5_MOUSE	DnaJ homolog subfamily C member 5 OS=Mus musculus OX=10090 GN=Dnajc5 PE=1 SV=1	
UGDH_MOUSE	UDP-glucose 6-dehydrogenase OS=Mus musculus OX=10090 GN=Ugdh PE=1 SV=1	
RL31_MOUSE	60S ribosomal protein L31 OS=Mus musculus OX=10090 GN=Rpl31 PE=1 SV=1	
PTBP1_MOUSE	Polypyrimidine tract-binding protein 1 OS=Mus musculus OX=10090 GN=Ptbp1 PE=1 SV=2	
RRBP1_MOUSE	Ribosome-binding protein 1 OS=Mus musculus OX=10090 GN=Rrbp1 PE=1 SV=2	
A4_RAT	Amyloid-beta A4 protein OS=Rattus norvegicus OX=10116 GN=App PE=1 SV=2	
RS10_MOUSE	40S ribosomal protein S10 OS=Mus musculus OX=10090 GN=Rps10 PE=1 SV=1	
RS27_MOUSE	40S ribosomal protein S27 OS=Mus musculus OX=10090 GN=Rps27 PE=1 SV=3	
ELOB_MOUSE	Elongin-B OS=Mus musculus OX=10090 GN=Elob PE=1 SV=1	
HACD3_MOUSE	Very-long-chain (3R)-3-hydroxyacyl-CoA dehydratase 3 OS=Mus musculus OX=10090 GN=Hacd3 PE=1 SV=2	
TPM4_MOUSE	Tropomyosin alpha-4 chain OS=Mus musculus OX=10090 GN=Tpm4 PE=1 SV=3	
CATD_MOUSE	Cathepsin D OS=Mus musculus OX=10090 GN=Ctsd PE=1 SV=1	
NASP_MOUSE	Nuclear autoantigenic sperm protein OS=Mus musculus OX=10090 GN=Nasp PE=1 SV=2	
LIPA2_MOUSE	Liprin-alpha-2 OS=Mus musculus OX=10090 GN=Ppfia2 PE=1 SV=2	
LSAMP_RAT	Limbic system-associated membrane protein OS=Rattus norvegicus OX=10116 GN=Lsamp PE=1 SV=1	
CELF2_MOUSE	CUGBP Elav-like family member 2 OS=Mus musculus OX=10090 GN=Celf2 PE=1 SV=1	
HDAC2_MOUSE	Histone deacetylase 2 OS=Mus musculus OX=10090 GN=Hdac2 PE=1 SV=1	
RL19_MOUSE	60S ribosomal protein L19 OS=Mus musculus OX=10090 GN=Rpl19 PE=1 SV=1	
CYB5B_MOUSE	Cytochrome b5 type B OS=Mus musculus OX=10090 GN=Cyb5b PE=1 SV=1	
ANM5_MOUSE	Protein arginine N-methyltransferase 5 OS=Mus musculus OX=10090 GN=Prmt5 PE=1 SV=3	
EIF3I_MOUSE	Eukaryotic translation initiation factor 3 subunit I OS=Mus musculus OX=10090 GN=Eif3i PE=1 SV=1	
SAE1_MOUSE	SUMO-activating enzyme subunit 1 OS=Mus musculus OX=10090 GN=Sae1 PE=1 SV=1	
GRB2_MOUSE	Growth factor receptor-bound protein 2 OS=Mus musculus OX=10090 GN=Grb2 PE=1 SV=1	
ELP1_MOUSE	Elongator complex protein 1 OS=Mus musculus OX=10090 GN=Elp1 PE=1 SV=2	
ATRX_MOUSE	Transcriptional regulator ATRX OS=Mus musculus OX=10090 GN=Atrx PE=1 SV=3	
PP2BB_MOUSE	Serine/threonine-protein phosphatase 2B catalytic subunit beta isoform OS=Mus musculus OX=10090 GN=Ppp3cb PE=1 SV=2	
IF4G2_MOUSE	Eukaryotic translation initiation factor 4 gamma 2 OS=Mus musculus OX=10090 GN=Eif4g2 PE=1 SV=2	
SCG2_MOUSE	Secretogranin-2 OS=Mus musculus OX=10090 GN=Scg2 PE=1 SV=1	
TM9S2_MOUSE	Transmembrane 9 superfamily member 2 OS=Mus musculus OX=10090 GN=Tm9sf2 PE=1 SV=1	
EAA2_MOUSE	Excitatory amino acid transporter 2 OS=Mus musculus OX=10090 GN=Slc1a2 PE=1 SV=1	
LAC2_MOUSE	Ig lambda-2 chain C region OS=Mus musculus OX=10090 GN=Iglc2 PE=1 SV=1	
MARE2_MOUSE	Microtubule-associated protein RP/EB family member 2 OS=Mus musculus OX=10090 GN=Mapre2 PE=1 SV=1	
ACTB_BOVIN	Actin, cytoplasmic 1 OS=Bos taurus OX=9913 GN=ACTB PE=1 SV=1	
LRC47_MOUSE	Leucine-rich repeat-containing protein 47 OS=Mus musculus OX=10090 GN=Lrrc47 PE=1 SV=1	
RL28_MOUSE	60S ribosomal protein L28 OS=Mus musculus OX=10090 GN=Rpl28 PE=1 SV=2	
TWF1_MOUSE	Twinfilin-1 OS=Mus musculus OX=10090 GN=Twf1 PE=1 SV=2	
U2AF1_MOUSE	Splicing factor U2AF 35 kDa subunit OS=Mus musculus OX=10090 GN=U2af1 PE=1 SV=4	
NDUV2_MOUSE	NADH dehydrogenase [ubiquinone] flavoprotein 2, mitochondrial OS=Mus musculus OX=10090 GN=Ndufv2 PE=1 SV=2	
NCEH1_MOUSE	Neutral cholesterol ester hydrolase 1 OS=Mus musculus OX=10090 GN=Nceh1 PE=1 SV=1	
NDUS8_MOUSE	NADH dehydrogenase [ubiquinone] iron-sulfur protein 8, mitochondrial OS=Mus musculus OX=10090 GN=Ndufs8 PE=1 SV=1	
SGT1_MOUSE	Protein SGT1 homolog OS=Mus musculus OX=10090 GN=Sugt1 PE=1 SV=3	
AIMP2_MOUSE	Aminoacyl tRNA synthase complex-interacting multifunctional protein 2 OS=Mus musculus OX=10090 GN=Aimp2 PE=1 SV=2	
DECR_MOUSE	2,4-dienoyl-CoA reductase, mitochondrial OS=Mus musculus OX=10090 GN=Decr1 PE=1 SV=1	
RS15A_MOUSE	40S ribosomal protein S15a OS=Mus musculus OX=10090 GN=Rps15a PE=1 SV=2	
CPNS1_MOUSE	Calpain small subunit 1 OS=Mus musculus OX=10090 GN=Capns1 PE=1 SV=1	
SFXN1_MOUSE	Sideroflexin-1 OS=Mus musculus OX=10090 GN=Sfxn1 PE=1 SV=3	
CD47_MOUSE	Leukocyte surface antigen CD47 OS=Mus musculus OX=10090 GN=Cd47 PE=1 SV=2	
TAGL3_MOUSE	Transgelin-3 OS=Mus musculus OX=10090 GN=Tagln3 PE=1 SV=1	
PCCA_MOUSE	Propionyl-CoA carboxylase alpha chain, mitochondrial OS=Mus musculus OX=10090 GN=Pcca PE=1 SV=2	
AOFA_MOUSE	Amine oxidase [flavin-containing] A OS=Mus musculus OX=10090 GN=Maoa PE=1 SV=3	
CON__P00735	CON__P00735	
CXAR_MOUSE	Coxsackievirus and adenovirus receptor homolog OS=Mus musculus OX=10090 GN=Cxadr PE=1 SV=1	
GDN_MOUSE	Glia-derived nexin OS=Mus musculus OX=10090 GN=Serpine2 PE=1 SV=2	
CPSF6_MOUSE	Cleavage and polyadenylation specificity factor subunit 6 OS=Mus musculus OX=10090 GN=Cpsf6 PE=1 SV=1	
TMM65_MOUSE	Transmembrane protein 65 OS=Mus musculus OX=10090 GN=Tmem65 PE=1 SV=1	
KAP2_MOUSE	cAMP-dependent protein kinase type II-alpha regulatory subunit OS=Mus musculus OX=10090 GN=Prkar2a PE=1 SV=2	
MAOX_MOUSE	NADP-dependent malic enzyme OS=Mus musculus OX=10090 GN=Me1 PE=1 SV=2	
NRX3A_MOUSE	Neurexin-3 OS=Mus musculus OX=10090 GN=Nrxn3 PE=1 SV=2	
LMAN2_MOUSE	Vesicular integral-membrane protein VIP36 OS=Mus musculus OX=10090 GN=Lman2 PE=1 SV=2	
SORC2_MOUSE	VPS10 domain-containing receptor SorCS2 OS=Mus musculus OX=10090 GN=Sorcs2 PE=1 SV=2	
XPP1_MOUSE	Xaa-Pro aminopeptidase 1 OS=Mus musculus OX=10090 GN=Xpnpep1 PE=1 SV=1	
BCAL2_ARATH	Branched-chain-amino-acid aminotransferase-like protein 2 OS=Arabidopsis thaliana OX=3702 GN=At5g27410 PE=2 SV=1	
FIS1_MOUSE	Mitochondrial fission 1 protein OS=Mus musculus OX=10090 GN=Fis1 PE=1 SV=1	
FKB1A_MOUSE	Peptidyl-prolyl cis-trans isomerase FKBP1A OS=Mus musculus OX=10090 GN=Fkbp1a PE=1 SV=2	
NDUA5_MOUSE	NADH dehydrogenase [ubiquinone] 1 alpha subcomplex subunit 5 OS=Mus musculus OX=10090 GN=Ndufa5 PE=1 SV=3	
CON__P13647	CON__P13647	
THOC4_MOUSE	THO complex subunit 4 OS=Mus musculus OX=10090 GN=Alyref PE=1 SV=3	
SRP68_MOUSE	Signal recognition particle subunit SRP68 OS=Mus musculus OX=10090 GN=Srp68 PE=1 SV=2	
APT_MOUSE	Adenine phosphoribosyltransferase OS=Mus musculus OX=10090 GN=Aprt PE=1 SV=2	
CLIC4_MOUSE	Chloride intracellular channel protein 4 OS=Mus musculus OX=10090 GN=Clic4 PE=1 SV=3	
CYC_MOUSE	Cytochrome c, somatic OS=Mus musculus OX=10090 GN=Cycs PE=1 SV=2	
ACADM_MOUSE	Medium-chain specific acyl-CoA dehydrogenase, mitochondrial OS=Mus musculus OX=10090 GN=Acadm PE=1 SV=1	
sp|Q6ZPJ3|UBE2O_MOUSE	E2 ubiquitin-conjugating enzyme UBE2O OS=Mus musculus OX=10090 GN=Ube2o PE=1 SV=3	
BAF_MOUSE	Barrier-to-autointegration factor OS=Mus musculus OX=10090 GN=Banf1 PE=1 SV=1	
ERF1_HUMAN	Eukaryotic peptide chain release factor subunit 1 OS=Homo sapiens OX=9606 GN=ETF1 PE=1 SV=3	
RL10A_MOUSE	60S ribosomal protein L10a OS=Mus musculus OX=10090 GN=Rpl10a PE=1 SV=3	
NOVA1_MOUSE	RNA-binding protein Nova-1 OS=Mus musculus OX=10090 GN=Nova1 PE=1 SV=2	
TRXR1_MOUSE	Thioredoxin reductase 1, cytoplasmic OS=Mus musculus OX=10090 GN=Txnrd1 PE=1 SV=3	
TSP1_MOUSE	Thrombospondin-1 OS=Mus musculus OX=10090 GN=Thbs1 PE=1 SV=1	
HIP1_MOUSE	Huntingtin-interacting protein 1 OS=Mus musculus OX=10090 GN=Hip1 PE=1 SV=2	
PSDE_MOUSE	26S proteasome non-ATPase regulatory subunit 14 OS=Mus musculus OX=10090 GN=Psmd14 PE=1 SV=2	
PSMD4_MOUSE	26S proteasome non-ATPase regulatory subunit 4 OS=Mus musculus OX=10090 GN=Psmd4 PE=1 SV=1	
DYN2_MOUSE	Dynamin-2 OS=Mus musculus OX=10090 GN=Dnm2 PE=1 SV=2	
TMED9_MOUSE	Transmembrane emp24 domain-containing protein 9 OS=Mus musculus OX=10090 GN=Tmed9 PE=1 SV=2	
CSK2B_HUMAN	Casein kinase II subunit beta OS=Homo sapiens OX=9606 GN=CSNK2B PE=1 SV=1	
DC1L2_MOUSE	Cytoplasmic dynein 1 light intermediate chain 2 OS=Mus musculus OX=10090 GN=Dync1li2 PE=1 SV=2	
GD1L1_MOUSE	Ganglioside-induced differentiation-associated protein 1-like 1 OS=Mus musculus OX=10090 GN=Gdap1l1 PE=1 SV=1	
SEPT2_MOUSE	Septin-2 OS=Mus musculus OX=10090 GN=Septin2 PE=1 SV=2	
MINK1_HUMAN	Misshapen-like kinase 1 OS=Homo sapiens OX=9606 GN=MINK1 PE=1 SV=2	
APMAP_MOUSE	Adipocyte plasma membrane-associated protein OS=Mus musculus OX=10090 GN=Apmap PE=1 SV=1	
SMC3_MOUSE	Structural maintenance of chromosomes protein 3 OS=Mus musculus OX=10090 GN=Smc3 PE=1 SV=2	
KV3AA_MOUSE	Ig kappa chain V-III region ABPC 22/PC 9245 OS=Mus musculus OX=10090 PE=1 SV=1	
AL4A1_MOUSE	Delta-1-pyrroline-5-carboxylate dehydrogenase, mitochondrial OS=Mus musculus OX=10090 GN=Aldh4a1 PE=1 SV=3	
DCAKD_MOUSE	Dephospho-CoA kinase domain-containing protein OS=Mus musculus OX=10090 GN=Dcakd PE=1 SV=1	
CALB2_MOUSE	Calretinin OS=Mus musculus OX=10090 GN=Calb2 PE=1 SV=3	
DYN3_MOUSE	Dynamin-3 OS=Mus musculus OX=10090 GN=Dnm3 PE=1 SV=1	
MIRO1_MOUSE	Mitochondrial Rho GTPase 1 OS=Mus musculus OX=10090 GN=Rhot1 PE=1 SV=1	
FKBP3_MOUSE	Peptidyl-prolyl cis-trans isomerase FKBP3 OS=Mus musculus OX=10090 GN=Fkbp3 PE=1 SV=2	
HPCL4_MOUSE	Hippocalcin-like protein 4 OS=Mus musculus OX=10090 GN=Hpcal4 PE=1 SV=3	
IF4G3_MOUSE	Eukaryotic translation initiation factor 4 gamma 3 OS=Mus musculus OX=10090 GN=Eif4g3 PE=1 SV=2	
SEPT9_MOUSE	Septin-9 OS=Mus musculus OX=10090 GN=Septin9 PE=1 SV=1	
API5_MOUSE	Apoptosis inhibitor 5 OS=Mus musculus OX=10090 GN=Api5 PE=1 SV=2	
TBB2B_MOUSE	Tubulin beta-2B chain OS=Mus musculus OX=10090 GN=Tubb2b PE=1 SV=1	
PSMD5_MOUSE	26S proteasome non-ATPase regulatory subunit 5 OS=Mus musculus OX=10090 GN=Psmd5 PE=1 SV=4	
RS14_MOUSE	40S ribosomal protein S14 OS=Mus musculus OX=10090 GN=Rps14 PE=1 SV=3	
EFHD2_MOUSE	EF-hand domain-containing protein D2 OS=Mus musculus OX=10090 GN=Efhd2 PE=1 SV=1	
HBB1_MOUSE	Hemoglobin subunit beta-1 OS=Mus musculus OX=10090 GN=Hbb-b1 PE=1 SV=2	
ARL1_MOUSE	ADP-ribosylation factor-like protein 1 OS=Mus musculus OX=10090 GN=Arl1 PE=1 SV=1	
ANXA4_MOUSE	Annexin A4 OS=Mus musculus OX=10090 GN=Anxa4 PE=1 SV=4	
ATPD_MOUSE	ATP synthase subunit delta, mitochondrial OS=Mus musculus OX=10090 GN=Atp5f1d PE=1 SV=1	
ITPA_MOUSE	Inosine triphosphate pyrophosphatase OS=Mus musculus OX=10090 GN=Itpa PE=1 SV=2	
ARF4_MOUSE	ADP-ribosylation factor 4 OS=Mus musculus OX=10090 GN=Arf4 PE=1 SV=2	
DAZP1_MOUSE	DAZ-associated protein 1 OS=Mus musculus OX=10090 GN=Dazap1 PE=1 SV=2	
RAB6B_MOUSE	Ras-related protein Rab-6B OS=Mus musculus OX=10090 GN=Rab6b PE=1 SV=1	
VATF_MOUSE	V-type proton ATPase subunit F OS=Mus musculus OX=10090 GN=Atp6v1f PE=1 SV=2	
PSB6_MOUSE	Proteasome subunit beta type-6 OS=Mus musculus OX=10090 GN=Psmb6 PE=1 SV=3	
MIC19_MOUSE	MICOS complex subunit Mic19 OS=Mus musculus OX=10090 GN=Chchd3 PE=1 SV=1	
TECR_MOUSE	Very-long-chain enoyl-CoA reductase OS=Mus musculus OX=10090 GN=Tecr PE=1 SV=1	
CPNE6_MOUSE	Copine-6 OS=Mus musculus OX=10090 GN=Cpne6 PE=1 SV=1	
PCCB_MOUSE	Propionyl-CoA carboxylase beta chain, mitochondrial OS=Mus musculus OX=10090 GN=Pccb PE=1 SV=2	
KI21A_MOUSE	Kinesin-like protein KIF21A OS=Mus musculus OX=10090 GN=Kif21a PE=1 SV=2	
RCN2_MOUSE	Reticulocalbin-2 OS=Mus musculus OX=10090 GN=Rcn2 PE=1 SV=1	
SNX1_MOUSE	Sorting nexin-1 OS=Mus musculus OX=10090 GN=Snx1 PE=1 SV=1	
CLIC1_MOUSE	Chloride intracellular channel protein 1 OS=Mus musculus OX=10090 GN=Clic1 PE=1 SV=3	
CLCA_MOUSE	Clathrin light chain A OS=Mus musculus OX=10090 GN=Clta PE=1 SV=2	
PDC6I_MOUSE	Programmed cell death 6-interacting protein OS=Mus musculus OX=10090 GN=Pdcd6ip PE=1 SV=3	
SYHC_MOUSE	Histidine--tRNA ligase, cytoplasmic OS=Mus musculus OX=10090 GN=Hars PE=1 SV=2	
DCPS_MOUSE	m7GpppX diphosphatase OS=Mus musculus OX=10090 GN=Dcps PE=1 SV=1	
SAC1_MOUSE	Phosphatidylinositide phosphatase SAC1 OS=Mus musculus OX=10090 GN=Sacm1l PE=1 SV=1	
RBGP1_MOUSE	Rab GTPase-activating protein 1 OS=Mus musculus OX=10090 GN=Rabgap1 PE=1 SV=1	
GRIA2_MOUSE	Glutamate receptor 2 OS=Mus musculus OX=10090 GN=Gria2 PE=1 SV=3	
CAB39_MOUSE	Calcium-binding protein 39 OS=Mus musculus OX=10090 GN=Cab39 PE=1 SV=2	
PSB1_MOUSE	Proteasome subunit beta type-1 OS=Mus musculus OX=10090 GN=Psmb1 PE=1 SV=1	
KV3AJ_MOUSE	Ig kappa chain V-III region PC 7175 OS=Mus musculus OX=10090 PE=1 SV=1	
NLGN2_MOUSE	Neuroligin-2 OS=Mus musculus OX=10090 GN=Nlgn2 PE=1 SV=2	
IF4E_MOUSE	Eukaryotic translation initiation factor 4E OS=Mus musculus OX=10090 GN=Eif4e PE=1 SV=1	
SRCN1_MOUSE	SRC kinase signaling inhibitor 1 OS=Mus musculus OX=10090 GN=Srcin1 PE=1 SV=2	
ATP5J_MOUSE	ATP synthase-coupling factor 6, mitochondrial OS=Mus musculus OX=10090 GN=Atp5pf PE=1 SV=1	
CAZA1_MOUSE	F-actin-capping protein subunit alpha-1 OS=Mus musculus OX=10090 GN=Capza1 PE=1 SV=4	
KV5A3_MOUSE	Ig kappa chain V-V region K2 (Fragment) OS=Mus musculus OX=10090 PE=1 SV=1	
FA98B_MOUSE	Protein FAM98B OS=Mus musculus OX=10090 GN=Fam98b PE=1 SV=1	
PSMD8_MOUSE	26S proteasome non-ATPase regulatory subunit 8 OS=Mus musculus OX=10090 GN=Psmd8 PE=1 SV=2	
RAB1A_MOUSE	Ras-related protein Rab-1A OS=Mus musculus OX=10090 GN=Rab1A PE=1 SV=3	
CASP3_MOUSE	Caspase-3 OS=Mus musculus OX=10090 GN=Casp3 PE=1 SV=1	
RASN_MOUSE	GTPase NRas OS=Mus musculus OX=10090 GN=Nras PE=1 SV=1	
ARPC4_MOUSE	Actin-related protein 2/3 complex subunit 4 OS=Mus musculus OX=10090 GN=Arpc4 PE=1 SV=3	
STMN1_MOUSE	Stathmin OS=Mus musculus OX=10090 GN=Stmn1 PE=1 SV=2	
CDS2_MOUSE	Phosphatidate cytidylyltransferase 2 OS=Mus musculus OX=10090 GN=Cds2 PE=1 SV=1	
H11_MOUSE	Histone H1.1 OS=Mus musculus OX=10090 GN=H1-1 PE=1 SV=2	
PAK1_MOUSE	Serine/threonine-protein kinase PAK 1 OS=Mus musculus OX=10090 GN=Pak1 PE=1 SV=1	
DYL2_MOUSE	Dynein light chain 2, cytoplasmic OS=Mus musculus OX=10090 GN=Dynll2 PE=1 SV=1	
KTN1_MOUSE	Kinectin OS=Mus musculus OX=10090 GN=Ktn1 PE=1 SV=1	
SCRB2_MOUSE	Lysosome membrane protein 2 OS=Mus musculus OX=10090 GN=Scarb2 PE=1 SV=3	
CSN3_MOUSE	COP9 signalosome complex subunit 3 OS=Mus musculus OX=10090 GN=Cops3 PE=1 SV=3	
EWS_MOUSE	RNA-binding protein EWS OS=Mus musculus OX=10090 GN=Ewsr1 PE=1 SV=2	
TXND5_MOUSE	Thioredoxin domain-containing protein 5 OS=Mus musculus OX=10090 GN=Txndc5 PE=1 SV=2	
SAR1B_MOUSE	GTP-binding protein SAR1b OS=Mus musculus OX=10090 GN=Sar1b PE=1 SV=1	
EZRI_MOUSE	Ezrin OS=Mus musculus OX=10090 GN=Ezr PE=1 SV=3	
MARE3_MOUSE	Microtubule-associated protein RP/EB family member 3 OS=Mus musculus OX=10090 GN=Mapre3 PE=1 SV=1	
GBG4_MOUSE	Guanine nucleotide-binding protein G(I)/G(S)/G(O) subunit gamma-4 OS=Mus musculus OX=10090 GN=Gng4 PE=1 SV=1	
EP15R_MOUSE	Epidermal growth factor receptor substrate 15-like 1 OS=Mus musculus OX=10090 GN=Eps15l1 PE=1 SV=3	
IF4B_MOUSE	Eukaryotic translation initiation factor 4B OS=Mus musculus OX=10090 GN=Eif4b PE=1 SV=1	
PCYXL_MOUSE	Prenylcysteine oxidase-like OS=Mus musculus OX=10090 GN=Pcyox1l PE=1 SV=1	
CP51A_MOUSE	Lanosterol 14-alpha demethylase OS=Mus musculus OX=10090 GN=Cyp51a1 PE=1 SV=1	
MCCB_MOUSE	Methylcrotonoyl-CoA carboxylase beta chain, mitochondrial OS=Mus musculus OX=10090 GN=Mccc2 PE=1 SV=1	
NDKB_MOUSE	Nucleoside diphosphate kinase B OS=Mus musculus OX=10090 GN=Nme2 PE=1 SV=1	
ARLY_MOUSE	Argininosuccinate lyase OS=Mus musculus OX=10090 GN=Asl PE=1 SV=1	
RB6I2_MOUSE	ELKS/Rab6-interacting/CAST family member 1 OS=Mus musculus OX=10090 GN=Erc1 PE=1 SV=1	
RAB35_MOUSE	Ras-related protein Rab-35 OS=Mus musculus OX=10090 GN=Rab35 PE=1 SV=1	
ATAT_MOUSE	Alpha-tubulin N-acetyltransferase 1 OS=Mus musculus OX=10090 GN=Atat1 PE=1 SV=1	
STT3B_MOUSE	Dolichyl-diphosphooligosaccharide--protein glycosyltransferase subunit STT3B OS=Mus musculus OX=10090 GN=Stt3b PE=1 SV=2	
SYGP1_MOUSE	Ras/Rap GTPase-activating protein SynGAP OS=Mus musculus OX=10090 GN=Syngap1 PE=1 SV=2	
SMC1A_MOUSE	Structural maintenance of chromosomes protein 1A OS=Mus musculus OX=10090 GN=Smc1a PE=1 SV=4	
CLAP1_MOUSE	CLIP-associating protein 1 OS=Mus musculus OX=10090 GN=Clasp1 PE=1 SV=2	
SRGP2_MOUSE	SLIT-ROBO Rho GTPase-activating protein 2 OS=Mus musculus OX=10090 GN=Srgap2 PE=1 SV=2	
ZRAB2_MOUSE	Zinc finger Ran-binding domain-containing protein 2 OS=Mus musculus OX=10090 GN=Zranb2 PE=1 SV=2	
NDUA8_MOUSE	NADH dehydrogenase [ubiquinone] 1 alpha subcomplex subunit 8 OS=Mus musculus OX=10090 GN=Ndufa8 PE=1 SV=3	
GPD1L_MOUSE	Glycerol-3-phosphate dehydrogenase 1-like protein OS=Mus musculus OX=10090 GN=Gpd1l PE=1 SV=2	
CADM2_MOUSE	Cell adhesion molecule 2 OS=Mus musculus OX=10090 GN=Cadm2 PE=1 SV=2	
RS15_CHICK	40S ribosomal protein S15 OS=Gallus gallus OX=9031 GN=RPS15 PE=2 SV=2	
F120A_MOUSE	Constitutive coactivator of PPAR-gamma-like protein 1 OS=Mus musculus OX=10090 GN=FAM120A PE=1 SV=2	
TRIM2_MOUSE	Tripartite motif-containing protein 2 OS=Mus musculus OX=10090 GN=Trim2 PE=1 SV=1	
SSRP1_MOUSE	FACT complex subunit SSRP1 OS=Mus musculus OX=10090 GN=Ssrp1 PE=1 SV=2	
SYQ_MOUSE	Glutamine--tRNA ligase OS=Mus musculus OX=10090 GN=Qars PE=1 SV=1	
NDRG4_MOUSE	Protein NDRG4 OS=Mus musculus OX=10090 GN=Ndrg4 PE=1 SV=1	
Q3E960-DECOY	Q3E960	
RL22_MOUSE	60S ribosomal protein L22 OS=Mus musculus OX=10090 GN=Rpl22 PE=1 SV=2	
PP1A_MOUSE	Serine/threonine-protein phosphatase PP1-alpha catalytic subunit OS=Mus musculus OX=10090 GN=Ppp1ca PE=1 SV=1	
CISD1_MOUSE	CDGSH iron-sulfur domain-containing protein 1 OS=Mus musculus OX=10090 GN=Cisd1 PE=1 SV=1	
NRX2A_MOUSE	Neurexin-2 OS=Mus musculus OX=10090 GN=Nrxn2 PE=1 SV=1	
HVM32_MOUSE	Ig heavy chain V-III region J606 OS=Mus musculus OX=10090 PE=1 SV=1	
CX7A2_MOUSE	Cytochrome c oxidase subunit 7A2, mitochondrial OS=Mus musculus OX=10090 GN=Cox7a2 PE=1 SV=2	
GNA11_MOUSE	Guanine nucleotide-binding protein subunit alpha-11 OS=Mus musculus OX=10090 GN=Gna11 PE=1 SV=1	
CATB_MOUSE	Cathepsin B OS=Mus musculus OX=10090 GN=Ctsb PE=1 SV=2	
KCD12_MOUSE	BTB/POZ domain-containing protein KCTD12 OS=Mus musculus OX=10090 GN=Kctd12 PE=1 SV=1	
OX2G_MOUSE	OX-2 membrane glycoprotein OS=Mus musculus OX=10090 GN=Cd200 PE=1 SV=1	
BIN1_MOUSE	Myc box-dependent-interacting protein 1 OS=Mus musculus OX=10090 GN=Bin1 PE=1 SV=1	
PPME1_MOUSE	Protein phosphatase methylesterase 1 OS=Mus musculus OX=10090 GN=Ppme1 PE=1 SV=5	
PURA_MOUSE	Transcriptional activator protein Pur-alpha OS=Mus musculus OX=10090 GN=Pura PE=1 SV=1	
VPS29_MOUSE	Vacuolar protein sorting-associated protein 29 OS=Mus musculus OX=10090 GN=Vps29 PE=1 SV=1	
BUB3_MOUSE	Mitotic checkpoint protein BUB3 OS=Mus musculus OX=10090 GN=Bub3 PE=1 SV=2	
NECA2_MOUSE	N-terminal EF-hand calcium-binding protein 2 OS=Mus musculus OX=10090 GN=Necab2 PE=1 SV=1	
DC1I2_MOUSE	Cytoplasmic dynein 1 intermediate chain 2 OS=Mus musculus OX=10090 GN=Dync1i2 PE=1 SV=1	
HDGF_MOUSE	Hepatoma-derived growth factor OS=Mus musculus OX=10090 GN=Hdgf PE=1 SV=2	
CPSF5_MOUSE	Cleavage and polyadenylation specificity factor subunit 5 OS=Mus musculus OX=10090 GN=Nudt21 PE=1 SV=1	
CDC37_MOUSE	Hsp90 co-chaperone Cdc37 OS=Mus musculus OX=10090 GN=Cdc37 PE=1 SV=1	
DDAH2_MOUSE	N(G),N(G)-dimethylarginine dimethylaminohydrolase 2 OS=Mus musculus OX=10090 GN=Ddah2 PE=1 SV=1	
SRRT_MOUSE	Serrate RNA effector molecule homolog OS=Mus musculus OX=10090 GN=Srrt PE=1 SV=1	
CTND2_MOUSE	Catenin delta-2 OS=Mus musculus OX=10090 GN=Ctnnd2 PE=1 SV=1	
HVM36_MOUSE	Ig heavy chain V region 441 OS=Mus musculus OX=10090 PE=4 SV=1	
SYTC_MOUSE	Threonine--tRNA ligase 1, cytoplasmic OS=Mus musculus OX=10090 GN=Tars1 PE=1 SV=2	
SNG3_MOUSE	Synaptogyrin-3 OS=Mus musculus OX=10090 GN=Syngr3 PE=1 SV=1	
RL27_MOUSE	60S ribosomal protein L27 OS=Mus musculus OX=10090 GN=Rpl27 PE=1 SV=2	
TCAL5_MOUSE	Transcription elongation factor A protein-like 5 OS=Mus musculus OX=10090 GN=Tceal5 PE=1 SV=1	
PTMA_MOUSE	Prothymosin alpha OS=Mus musculus OX=10090 GN=Ptma PE=1 SV=2	
RS20_MOUSE	40S ribosomal protein S20 OS=Mus musculus OX=10090 GN=Rps20 PE=1 SV=1	
NOLC1_MOUSE	Nucleolar and coiled-body phosphoprotein 1 OS=Mus musculus OX=10090 GN=Nolc1 PE=1 SV=1	
GDPD1_MOUSE	Lysophospholipase D GDPD1 OS=Mus musculus OX=10090 GN=Gdpd1 PE=1 SV=1	
TBA4A_MOUSE	Tubulin alpha-4A chain OS=Mus musculus OX=10090 GN=Tuba4a PE=1 SV=1	
RHOB_MOUSE	Rho-related GTP-binding protein RhoB OS=Mus musculus OX=10090 GN=Rhob PE=1 SV=1	
SEPT6_MOUSE	Septin-6 OS=Mus musculus OX=10090 GN=Septin6 PE=1 SV=4	
NMT2_MOUSE	Glycylpeptide N-tetradecanoyltransferase 2 OS=Mus musculus OX=10090 GN=Nmt2 PE=1 SV=1	
SF3A1_MOUSE	Splicing factor 3A subunit 1 OS=Mus musculus OX=10090 GN=Sf3a1 PE=1 SV=1	
KIF3A_MOUSE	Kinesin-like protein KIF3A OS=Mus musculus OX=10090 GN=Kif3a PE=1 SV=2	
ATAD3_MOUSE	ATPase family AAA domain-containing protein 3 OS=Mus musculus OX=10090 GN=Atad3 PE=1 SV=1	
BT3L4_MOUSE	Transcription factor BTF3 homolog 4 OS=Mus musculus OX=10090 GN=Btf3l4 PE=1 SV=1	
NDUB6_MOUSE	NADH dehydrogenase [ubiquinone] 1 beta subcomplex subunit 6 OS=Mus musculus OX=10090 GN=Ndufb6 PE=1 SV=3	
TMM43_MOUSE	Transmembrane protein 43 OS=Mus musculus OX=10090 GN=Tmem43 PE=1 SV=1	
UBE3A_MOUSE	Ubiquitin-protein ligase E3A OS=Mus musculus OX=10090 GN=Ube3a PE=1 SV=2	
IF4H_MOUSE	Eukaryotic translation initiation factor 4H OS=Mus musculus OX=10090 GN=Eif4h PE=1 SV=3	
CSKP_MOUSE	Peripheral plasma membrane protein CASK OS=Mus musculus OX=10090 GN=Cask PE=1 SV=2	
RINI_MOUSE	Ribonuclease inhibitor OS=Mus musculus OX=10090 GN=Rnh1 PE=1 SV=1	
TOM40_MOUSE	Mitochondrial import receptor subunit TOM40 homolog OS=Mus musculus OX=10090 GN=Tomm40 PE=1 SV=3	
OGFR_MOUSE	Opioid growth factor receptor OS=Mus musculus OX=10090 GN=Ogfr PE=1 SV=1	
ANFY1_MOUSE	Rabankyrin-5 OS=Mus musculus OX=10090 GN=Ankfy1 PE=1 SV=2	
ABCE1_MOUSE	ATP-binding cassette sub-family E member 1 OS=Mus musculus OX=10090 GN=Abce1 PE=1 SV=1	
ATLA1_MOUSE	Atlastin-1 OS=Mus musculus OX=10090 GN=Atl1 PE=1 SV=1	
PPM1E_MOUSE	Protein phosphatase 1E OS=Mus musculus OX=10090 GN=Ppm1e PE=1 SV=2	
ERH_MOUSE	Enhancer of rudimentary homolog OS=Mus musculus OX=10090 GN=Erh PE=1 SV=1	
UBE2K_MOUSE	Ubiquitin-conjugating enzyme E2 K OS=Mus musculus OX=10090 GN=Ube2k PE=1 SV=3	
SYPH_MOUSE	Synaptophysin OS=Mus musculus OX=10090 GN=Syp PE=1 SV=2	
TCEA1_MOUSE	Transcription elongation factor A protein 1 OS=Mus musculus OX=10090 GN=Tcea1 PE=1 SV=2	
CC50A_MOUSE	Cell cycle control protein 50A OS=Mus musculus OX=10090 GN=Tmem30a PE=1 SV=1	
SMD1_MOUSE	Small nuclear ribonucleoprotein Sm D1 OS=Mus musculus OX=10090 GN=Snrpd1 PE=1 SV=1	
TMX2_MOUSE	Thioredoxin-related transmembrane protein 2 OS=Mus musculus OX=10090 GN=Tmx2 PE=1 SV=1	
RS23_MOUSE	40S ribosomal protein S23 OS=Mus musculus OX=10090 GN=Rps23 PE=1 SV=3	
LGUL_MOUSE	Lactoylglutathione lyase OS=Mus musculus OX=10090 GN=Glo1 PE=1 SV=3	
ACBG1_MOUSE	Long-chain-fatty-acid--CoA ligase ACSBG1 OS=Mus musculus OX=10090 GN=Acsbg1 PE=1 SV=1	
ZN512_MOUSE	Zinc finger protein 512 OS=Mus musculus OX=10090 GN=Znf512 PE=2 SV=2	
CNRP1_MOUSE	CB1 cannabinoid receptor-interacting protein 1 OS=Mus musculus OX=10090 GN=Cnrip1 PE=1 SV=1	
NCAM2_MOUSE	Neural cell adhesion molecule 2 OS=Mus musculus OX=10090 GN=Ncam2 PE=1 SV=1	
LC7L2_MOUSE	Putative RNA-binding protein Luc7-like 2 OS=Mus musculus OX=10090 GN=Luc7l2 PE=1 SV=1	
CSN6_MOUSE	COP9 signalosome complex subunit 6 OS=Mus musculus OX=10090 GN=Cops6 PE=1 SV=1	
NECP1_MOUSE	Adaptin ear-binding coat-associated protein 1 OS=Mus musculus OX=10090 GN=Necap1 PE=1 SV=2	
DLG3_MOUSE	Disks large homolog 3 OS=Mus musculus OX=10090 GN=Dlg3 PE=1 SV=1	
TOP2A_MOUSE	DNA topoisomerase 2-alpha OS=Mus musculus OX=10090 GN=Top2a PE=1 SV=2	
RBM14_MOUSE	RNA-binding protein 14 OS=Mus musculus OX=10090 GN=Rbm14 PE=1 SV=1	
TPR_MOUSE	Nucleoprotein TPR OS=Mus musculus OX=10090 GN=Tpr PE=1 SV=1	
OGT1_MOUSE	UDP-N-acetylglucosamine--peptide N-acetylglucosaminyltransferase 110 kDa subunit OS=Mus musculus OX=10090 GN=Ogt PE=1 SV=2	
GCYA1_MOUSE	Guanylate cyclase soluble subunit alpha-1 OS=Mus musculus OX=10090 GN=Gucy1a1 PE=1 SV=2	
AL1L2_MOUSE	Mitochondrial 10-formyltetrahydrofolate dehydrogenase OS=Mus musculus OX=10090 GN=Aldh1l2 PE=1 SV=2	
LYRIC_MOUSE	Protein LYRIC OS=Mus musculus OX=10090 GN=Mtdh PE=1 SV=1	
3HIDH_MOUSE	3-hydroxyisobutyrate dehydrogenase, mitochondrial OS=Mus musculus OX=10090 GN=Hibadh PE=1 SV=1	
AL7A1_MOUSE	Alpha-aminoadipic semialdehyde dehydrogenase OS=Mus musculus OX=10090 GN=Aldh7a1 PE=1 SV=4	
ACOT1_MOUSE	Acyl-coenzyme A thioesterase 1 OS=Mus musculus OX=10090 GN=Acot1 PE=1 SV=1	
NUCB1_MOUSE	Nucleobindin-1 OS=Mus musculus OX=10090 GN=Nucb1 PE=1 SV=2	
NUP93_MOUSE	Nuclear pore complex protein Nup93 OS=Mus musculus OX=10090 GN=Nup93 PE=1 SV=1	
SYT5_MOUSE	Synaptotagmin-5 OS=Mus musculus OX=10090 GN=Syt5 PE=1 SV=1	
TIM44_MOUSE	Mitochondrial import inner membrane translocase subunit TIM44 OS=Mus musculus OX=10090 GN=Timm44 PE=1 SV=2	
BZW2_MOUSE	Basic leucine zipper and W2 domain-containing protein 2 OS=Mus musculus OX=10090 GN=Bzw2 PE=1 SV=1	
CPLX2_MOUSE	Complexin-2 OS=Mus musculus OX=10090 GN=Cplx2 PE=1 SV=1	
SARM1_MOUSE	Sterile alpha and TIR motif-containing protein 1 OS=Mus musculus OX=10090 GN=Sarm1 PE=1 SV=1	
THY1_MOUSE	Thy-1 membrane glycoprotein OS=Mus musculus OX=10090 GN=Thy1 PE=1 SV=1	
MTX2_MOUSE	Metaxin-2 OS=Mus musculus OX=10090 GN=Mtx2 PE=1 SV=1	
CON__P81644	CON__P81644	
HVM57_MOUSE	Ig heavy chain V region 6.96 OS=Mus musculus OX=10090 PE=4 SV=1	
CSRP1_MOUSE	Cysteine and glycine-rich protein 1 OS=Mus musculus OX=10090 GN=Csrp1 PE=1 SV=3	
PDCD6_MOUSE	Programmed cell death protein 6 OS=Mus musculus OX=10090 GN=Pdcd6 PE=1 SV=2	
CSN2_MOUSE	COP9 signalosome complex subunit 2 OS=Mus musculus OX=10090 GN=Cops2 PE=1 SV=1	
WASF1_MOUSE	Wiskott-Aldrich syndrome protein family member 1 OS=Mus musculus OX=10090 GN=Wasf1 PE=1 SV=2	
PRAF3_MOUSE	PRA1 family protein 3 OS=Mus musculus OX=10090 GN=Arl6ip5 PE=1 SV=2	
RTRAF_MOUSE	RNA transcription, translation and transport factor protein OS=Mus musculus OX=10090 GN=RTRAF PE=1 SV=1	
CIRBP_MOUSE	Cold-inducible RNA-binding protein OS=Mus musculus OX=10090 GN=Cirbp PE=1 SV=1	
ACTY_MOUSE	Beta-centractin OS=Mus musculus OX=10090 GN=Actr1b PE=1 SV=1	
HCDH_MOUSE	Hydroxyacyl-coenzyme A dehydrogenase, mitochondrial OS=Mus musculus OX=10090 GN=Hadh PE=1 SV=2	
CHIP_MOUSE	STIP1 homology and U box-containing protein 1 OS=Mus musculus OX=10090 GN=Stub1 PE=1 SV=1	
LTOR1_MOUSE	Ragulator complex protein LAMTOR1 OS=Mus musculus OX=10090 GN=Lamtor1 PE=1 SV=1	
ARL3_MOUSE	ADP-ribosylation factor-like protein 3 OS=Mus musculus OX=10090 GN=Arl3 PE=1 SV=1	
MGN2_MOUSE	Protein mago nashi homolog 2 OS=Mus musculus OX=10090 GN=Magohb PE=2 SV=1	
SMD3_MOUSE	Small nuclear ribonucleoprotein Sm D3 OS=Mus musculus OX=10090 GN=Snrpd3 PE=1 SV=1	
DCLK2_MOUSE	Serine/threonine-protein kinase DCLK2 OS=Mus musculus OX=10090 GN=Dclk2 PE=1 SV=1	
LKHA4_MOUSE	Leukotriene A-4 hydrolase OS=Mus musculus OX=10090 GN=Lta4h PE=1 SV=4	
RALA_MOUSE	Ras-related protein Ral-A OS=Mus musculus OX=10090 GN=Rala PE=1 SV=1	
AIFM1_MOUSE	Apoptosis-inducing factor 1, mitochondrial OS=Mus musculus OX=10090 GN=Aifm1 PE=1 SV=1	
KV3AD_MOUSE	Ig kappa chain V-III region PC 7043 OS=Mus musculus OX=10090 PE=1 SV=1	
BAG6_MOUSE	Large proline-rich protein BAG6 OS=Mus musculus OX=10090 GN=Bag6 PE=1 SV=1	
SRRM2_MOUSE	Serine/arginine repetitive matrix protein 2 OS=Mus musculus OX=10090 GN=Srrm2 PE=1 SV=3	
VTNC_MOUSE	Vitronectin OS=Mus musculus OX=10090 GN=Vtn PE=1 SV=2	
PACS1_MOUSE	Phosphofurin acidic cluster sorting protein 1 OS=Mus musculus OX=10090 GN=Pacs1 PE=1 SV=2	
ACTBL_MOUSE	Beta-actin-like protein 2 OS=Mus musculus OX=10090 GN=Actbl2 PE=1 SV=1	
CYFP1_MOUSE	Cytoplasmic FMR1-interacting protein 1 OS=Mus musculus OX=10090 GN=Cyfip1 PE=1 SV=1	
H2A2A_HUMAN	Histone H2A type 2-A OS=Homo sapiens OX=9606 GN=HIST2H2AA3 PE=1 SV=3	
HNRL1_MOUSE	Heterogeneous nuclear ribonucleoprotein U-like protein 1 OS=Mus musculus OX=10090 GN=Hnrnpul1 PE=1 SV=1	
RHG01_MOUSE	Rho GTPase-activating protein 1 OS=Mus musculus OX=10090 GN=Arhgap1 PE=1 SV=1	
FKBP8_MOUSE	Peptidyl-prolyl cis-trans isomerase FKBP8 OS=Mus musculus OX=10090 GN=Fkbp8 PE=1 SV=2	
TRA2A_MOUSE	Transformer-2 protein homolog alpha OS=Mus musculus OX=10090 GN=Tra2a PE=1 SV=1	
CEMIP_MOUSE	Cell migration-inducing and hyaluronan-binding protein OS=Mus musculus OX=10090 GN=Cemip PE=1 SV=4	
PHOCN_MOUSE	MOB-like protein phocein OS=Mus musculus OX=10090 GN=Mob4 PE=1 SV=1	
ELOC_MOUSE	Elongin-C OS=Mus musculus OX=10090 GN=Eloc PE=1 SV=1	
PFKAL_MOUSE	ATP-dependent 6-phosphofructokinase, liver type OS=Mus musculus OX=10090 GN=Pfkl PE=1 SV=4	
RELCH_MOUSE	RAB11-binding protein RELCH OS=Mus musculus OX=10090 GN=Relch PE=1 SV=1	
ACOX1_MOUSE	Peroxisomal acyl-coenzyme A oxidase 1 OS=Mus musculus OX=10090 GN=Acox1 PE=1 SV=5	
HMGB3_MOUSE	High mobility group protein B3 OS=Mus musculus OX=10090 GN=Hmgb3 PE=1 SV=3	
LXN_MOUSE	Latexin OS=Mus musculus OX=10090 GN=Lxn PE=1 SV=2	
ARF5_MOUSE	ADP-ribosylation factor 5 OS=Mus musculus OX=10090 GN=Arf5 PE=1 SV=2	
GAL3A_MOUSE	Glutamine amidotransferase-like class 1 domain-containing protein 3A, mitochondrial OS=Mus musculus OX=10090 GN=Gatd3a PE=1 SV=1	
TICN1_MOUSE	Testican-1 OS=Mus musculus OX=10090 GN=Spock1 PE=2 SV=2	
PCBP3_MOUSE	Poly(rC)-binding protein 3 OS=Mus musculus OX=10090 GN=Pcbp3 PE=1 SV=3	
PGRC2_MOUSE	Membrane-associated progesterone receptor component 2 OS=Mus musculus OX=10090 GN=Pgrmc2 PE=1 SV=2	
AP2S1_MOUSE	AP-2 complex subunit sigma OS=Mus musculus OX=10090 GN=Ap2s1 PE=1 SV=1	
RAP2B_MOUSE	Ras-related protein Rap-2b OS=Mus musculus OX=10090 GN=Rap2b PE=1 SV=1	
MIC26_MOUSE	MICOS complex subunit Mic26 OS=Mus musculus OX=10090 GN=Apoo PE=1 SV=2	
BPNT1_MOUSE	3'(2'),5'-bisphosphate nucleotidase 1 OS=Mus musculus OX=10090 GN=Bpnt1 PE=1 SV=2	
RU2A_MOUSE	U2 small nuclear ribonucleoprotein A' OS=Mus musculus OX=10090 GN=Snrpa1 PE=1 SV=2	
PI42B_MOUSE	Phosphatidylinositol 5-phosphate 4-kinase type-2 beta OS=Mus musculus OX=10090 GN=Pip4k2b PE=1 SV=1	
SMD2_MOUSE	Small nuclear ribonucleoprotein Sm D2 OS=Mus musculus OX=10090 GN=Snrpd2 PE=1 SV=1	
RBM39_MOUSE	RNA-binding protein 39 OS=Mus musculus OX=10090 GN=Rbm39 PE=1 SV=2	
SORCN_MOUSE	Sorcin OS=Mus musculus OX=10090 GN=Sri PE=1 SV=1	
SNX2_MOUSE	Sorting nexin-2 OS=Mus musculus OX=10090 GN=Snx2 PE=1 SV=2	
AUHM_MOUSE	Methylglutaconyl-CoA hydratase, mitochondrial OS=Mus musculus OX=10090 GN=Auh PE=1 SV=1	
SNX27_MOUSE	Sorting nexin-27 OS=Mus musculus OX=10090 GN=Snx27 PE=1 SV=2	
MCAT_MOUSE	Mitochondrial carnitine/acylcarnitine carrier protein OS=Mus musculus OX=10090 GN=Slc25a20 PE=1 SV=1	
AL1B1_MOUSE	Aldehyde dehydrogenase X, mitochondrial OS=Mus musculus OX=10090 GN=Aldh1b1 PE=1 SV=1	
ACSL1_MOUSE	Long-chain-fatty-acid--CoA ligase 1 OS=Mus musculus OX=10090 GN=Acsl1 PE=1 SV=2	
RAGP1_MOUSE	Ran GTPase-activating protein 1 OS=Mus musculus OX=10090 GN=Rangap1 PE=1 SV=2	
MK03_MOUSE	Mitogen-activated protein kinase 3 OS=Mus musculus OX=10090 GN=Mapk3 PE=1 SV=5	
PPP5_MOUSE	Serine/threonine-protein phosphatase 5 OS=Mus musculus OX=10090 GN=Ppp5c PE=1 SV=3	
TLN1_MOUSE	Talin-1 OS=Mus musculus OX=10090 GN=Tln1 PE=1 SV=2	
AMRP_MOUSE	Alpha-2-macroglobulin receptor-associated protein OS=Mus musculus OX=10090 GN=Lrpap1 PE=1 SV=1	
NFIX_MOUSE	Nuclear factor 1 X-type OS=Mus musculus OX=10090 GN=Nfix PE=1 SV=2	
H32_HUMAN	Histone H3.2 OS=Homo sapiens OX=9606 GN=HIST2H3A PE=1 SV=3	
UBP7_MOUSE	Ubiquitin carboxyl-terminal hydrolase 7 OS=Mus musculus OX=10090 GN=Usp7 PE=1 SV=1	
PTBP2_MOUSE	Polypyrimidine tract-binding protein 2 OS=Mus musculus OX=10090 GN=Ptbp2 PE=1 SV=2	
G3BP1_MOUSE	Ras GTPase-activating protein-binding protein 1 OS=Mus musculus OX=10090 GN=G3bp1 PE=1 SV=1	
TMX4_MOUSE	Thioredoxin-related transmembrane protein 4 OS=Mus musculus OX=10090 GN=Tmx4 PE=1 SV=2	
PRIO_MOUSE	Major prion protein OS=Mus musculus OX=10090 GN=Prnp PE=1 SV=2	
CAD13_MOUSE	Cadherin-13 OS=Mus musculus OX=10090 GN=Cdh13 PE=1 SV=2	
UCHL3_MOUSE	Ubiquitin carboxyl-terminal hydrolase isozyme L3 OS=Mus musculus OX=10090 GN=Uchl3 PE=1 SV=2	
RP3A_MOUSE	Rabphilin-3A OS=Mus musculus OX=10090 GN=Rph3a PE=1 SV=2	
AGAP3_MOUSE	Arf-GAP with GTPase, ANK repeat and PH domain-containing protein 3 OS=Mus musculus OX=10090 GN=Agap3 PE=1 SV=1	
KV5A1_MOUSE	Ig kappa chain V19-17 OS=Mus musculus OX=10090 GN=Igk-V19-17 PE=1 SV=1	
SSRA_MOUSE	Translocon-associated protein subunit alpha OS=Mus musculus OX=10090 GN=Ssr1 PE=1 SV=1	
RAB3B_MOUSE	Ras-related protein Rab-3B OS=Mus musculus OX=10090 GN=Rab3b PE=1 SV=1	
KV5A6_MOUSE	Ig kappa chain V-V region L6 (Fragment) OS=Mus musculus OX=10090 PE=4 SV=1	
PTPA_MOUSE	Serine/threonine-protein phosphatase 2A activator OS=Mus musculus OX=10090 GN=Ptpa PE=1 SV=1	
HDGR3_MOUSE	Hepatoma-derived growth factor-related protein 3 OS=Mus musculus OX=10090 GN=Hdgfl3 PE=1 SV=2	
KAPCA_MOUSE	cAMP-dependent protein kinase catalytic subunit alpha OS=Mus musculus OX=10090 GN=Prkaca PE=1 SV=3	
SUGP2_MOUSE	SURP and G-patch domain-containing protein 2 OS=Mus musculus OX=10090 GN=Sugp2 PE=1 SV=2	
SPCS2_MOUSE	Signal peptidase complex subunit 2 OS=Mus musculus OX=10090 GN=Spcs2 PE=1 SV=1	
EMC2_MOUSE	ER membrane protein complex subunit 2 OS=Mus musculus OX=10090 GN=Emc2 PE=1 SV=1	
STX12_MOUSE	Syntaxin-12 OS=Mus musculus OX=10090 GN=Stx12 PE=1 SV=1	
BAP31_MOUSE	B-cell receptor-associated protein 31 OS=Mus musculus OX=10090 GN=Bcap31 PE=1 SV=4	
FBLL1_MOUSE	rRNA/tRNA 2'-O-methyltransferase fibrillarin-like protein 1 OS=Mus musculus OX=10090 GN=Fbll1 PE=1 SV=1	
DYH8_HUMAN	Dynein heavy chain 8, axonemal OS=Homo sapiens OX=9606 GN=DNAH8 PE=1 SV=2	
S6A11_MOUSE	Sodium- and chloride-dependent GABA transporter 3 OS=Mus musculus OX=10090 GN=Slc6a11 PE=1 SV=2	
TOLIP_MOUSE	Toll-interacting protein OS=Mus musculus OX=10090 GN=Tollip PE=1 SV=1	
ATP9A_MOUSE	Probable phospholipid-transporting ATPase IIA OS=Mus musculus OX=10090 GN=Atp9a PE=1 SV=3	
GBRB3_MOUSE	Gamma-aminobutyric acid receptor subunit beta-3 OS=Mus musculus OX=10090 GN=Gabrb3 PE=1 SV=1	
GELS_MOUSE	Gelsolin OS=Mus musculus OX=10090 GN=Gsn PE=1 SV=3	
K0513_MOUSE	Uncharacterized protein KIAA0513 OS=Mus musculus OX=10090 GN=Kiaa0513 PE=1 SV=1	
SYCC_MOUSE	Cysteine--tRNA ligase, cytoplasmic OS=Mus musculus OX=10090 GN=Cars PE=1 SV=2	
IF1AX_MOUSE	Eukaryotic translation initiation factor 1A, X-chromosomal OS=Mus musculus OX=10090 GN=Eif1ax PE=2 SV=3	
KAD2_MOUSE	Adenylate kinase 2, mitochondrial OS=Mus musculus OX=10090 GN=Ak2 PE=1 SV=5	
TOM20_MOUSE	Mitochondrial import receptor subunit TOM20 homolog OS=Mus musculus OX=10090 GN=Tomm20 PE=1 SV=1	
DNER_MOUSE	Delta and Notch-like epidermal growth factor-related receptor OS=Mus musculus OX=10090 GN=Dner PE=1 SV=1	
NDRG2_MOUSE	Protein NDRG2 OS=Mus musculus OX=10090 GN=Ndrg2 PE=1 SV=1	
AL9A1_MOUSE	4-trimethylaminobutyraldehyde dehydrogenase OS=Mus musculus OX=10090 GN=Aldh9a1 PE=1 SV=1	
GLO2_MOUSE	Hydroxyacylglutathione hydrolase, mitochondrial OS=Mus musculus OX=10090 GN=Hagh PE=1 SV=2	
WDR37_MOUSE	WD repeat-containing protein 37 OS=Mus musculus OX=10090 GN=Wdr37 PE=1 SV=1	
EIF3G_MOUSE	Eukaryotic translation initiation factor 3 subunit G OS=Mus musculus OX=10090 GN=Eif3g PE=1 SV=2	
GSTA4_MOUSE	Glutathione S-transferase A4 OS=Mus musculus OX=10090 GN=Gsta4 PE=1 SV=3	
NUMA1_MOUSE	Nuclear mitotic apparatus protein 1 OS=Mus musculus OX=10090 GN=Numa1 PE=1 SV=1	
CELF1_MOUSE	CUGBP Elav-like family member 1 OS=Mus musculus OX=10090 GN=Celf1 PE=1 SV=2	
NNRD_MOUSE	ATP-dependent (S)-NAD(P)H-hydrate dehydratase OS=Mus musculus OX=10090 GN=Naxd PE=1 SV=1	
HDAC6_MOUSE	Histone deacetylase 6 OS=Mus musculus OX=10090 GN=Hdac6 PE=1 SV=3	
SARNP_MOUSE	SAP domain-containing ribonucleoprotein OS=Mus musculus OX=10090 GN=Sarnp PE=1 SV=3	
GPM6A_MOUSE	Neuronal membrane glycoprotein M6-a OS=Mus musculus OX=10090 GN=Gpm6a PE=1 SV=1	
MPC2_MOUSE	Mitochondrial pyruvate carrier 2 OS=Mus musculus OX=10090 GN=Mpc2 PE=1 SV=1	
RADI_MOUSE	Radixin OS=Mus musculus OX=10090 GN=Rdx PE=1 SV=3	
QCR8_MOUSE	Cytochrome b-c1 complex subunit 8 OS=Mus musculus OX=10090 GN=Uqcrq PE=1 SV=3	
YKT6_MOUSE	Synaptobrevin homolog YKT6 OS=Mus musculus OX=10090 GN=Ykt6 PE=1 SV=1	
KI21B_MOUSE	Kinesin-like protein KIF21B OS=Mus musculus OX=10090 GN=Kif21b PE=1 SV=2	
HSP72_MOUSE	Heat shock-related 70 kDa protein 2 OS=Mus musculus OX=10090 GN=Hspa2 PE=1 SV=2	
PYGL_RAT	Glycogen phosphorylase, liver form OS=Rattus norvegicus OX=10116 GN=Pygl PE=1 SV=5	
SR140_MOUSE	U2 snRNP-associated SURP motif-containing protein OS=Mus musculus OX=10090 GN=U2surp PE=1 SV=3	
ERP44_MOUSE	Endoplasmic reticulum resident protein 44 OS=Mus musculus OX=10090 GN=Erp44 PE=1 SV=1	
SNX6_MOUSE	Sorting nexin-6 OS=Mus musculus OX=10090 GN=Snx6 PE=1 SV=2	
GGT7_MOUSE	Glutathione hydrolase 7 OS=Mus musculus OX=10090 GN=Ggt7 PE=1 SV=2	
SOGA3_MOUSE	Protein SOGA3 OS=Mus musculus OX=10090 GN=Soga3 PE=1 SV=2	
PIMT_MOUSE	Protein-L-isoaspartate(D-aspartate) O-methyltransferase OS=Mus musculus OX=10090 GN=Pcmt1 PE=1 SV=3	
TM35A_MOUSE	Transmembrane protein 35A OS=Mus musculus OX=10090 GN=Tmem35a PE=1 SV=1	
ATP5I_MOUSE	ATP synthase subunit e, mitochondrial OS=Mus musculus OX=10090 GN=Atp5me PE=1 SV=2	
RS21_MOUSE	40S ribosomal protein S21 OS=Mus musculus OX=10090 GN=Rps21 PE=1 SV=1	
RL36A_MOUSE	60S ribosomal protein L36a OS=Mus musculus OX=10090 GN=Rpl36a PE=3 SV=2	
RTCA_MOUSE	RNA 3'-terminal phosphate cyclase OS=Mus musculus OX=10090 GN=RtcA PE=1 SV=2	
DYLT1_MOUSE	Dynein light chain Tctex-type 1 OS=Mus musculus OX=10090 GN=Dynlt1 PE=1 SV=1	
PRKRA_MOUSE	Interferon-inducible double-stranded RNA-dependent protein kinase activator A OS=Mus musculus OX=10090 GN=Prkra PE=1 SV=1	
EIF3H_MOUSE	Eukaryotic translation initiation factor 3 subunit H OS=Mus musculus OX=10090 GN=Eif3h PE=1 SV=1	
DEK_MOUSE	Protein DEK OS=Mus musculus OX=10090 GN=Dek PE=1 SV=1	
RAB31_MOUSE	Ras-related protein Rab-31 OS=Mus musculus OX=10090 GN=Rab31 PE=1 SV=1	
ASPH_MOUSE	Aspartyl/asparaginyl beta-hydroxylase OS=Mus musculus OX=10090 GN=Asph PE=1 SV=1	
CYB5_MOUSE	Cytochrome b5 OS=Mus musculus OX=10090 GN=Cyb5a PE=1 SV=2	
GORS2_MOUSE	Golgi reassembly-stacking protein 2 OS=Mus musculus OX=10090 GN=Gorasp2 PE=1 SV=3	
S4A10_MOUSE	Sodium-driven chloride bicarbonate exchanger OS=Mus musculus OX=10090 GN=Slc4a10 PE=1 SV=2	
SYIM_MOUSE	Isoleucine--tRNA ligase, mitochondrial OS=Mus musculus OX=10090 GN=Iars2 PE=1 SV=1	
RL32_MOUSE	60S ribosomal protein L32 OS=Mus musculus OX=10090 GN=Rpl32 PE=1 SV=2	
AMPB_MOUSE	Aminopeptidase B OS=Mus musculus OX=10090 GN=Rnpep PE=1 SV=2	
KVM5_MOUSE	Ig kappa chain V region Mem5 (Fragment) OS=Mus musculus OX=10090 PE=1 SV=1	
SC11A_MOUSE	Signal peptidase complex catalytic subunit SEC11A OS=Mus musculus OX=10090 GN=Sec11a PE=1 SV=1	
TBA1C_MOUSE	Tubulin alpha-1C chain OS=Mus musculus OX=10090 GN=Tuba1c PE=1 SV=1	
IMPCT_MOUSE	Protein IMPACT OS=Mus musculus OX=10090 GN=Impact PE=1 SV=2	
TOM34_MOUSE	Mitochondrial import receptor subunit TOM34 OS=Mus musculus OX=10090 GN=Tomm34 PE=1 SV=1	
SFXN5_MOUSE	Sideroflexin-5 OS=Mus musculus OX=10090 GN=Sfxn5 PE=1 SV=2	
CLCN6_MOUSE	Chloride transport protein 6 OS=Mus musculus OX=10090 GN=Clcn6 PE=1 SV=1	
P19137-DECOY	P19137	
GATM_MOUSE	Glycine amidinotransferase, mitochondrial OS=Mus musculus OX=10090 GN=Gatm PE=1 SV=1	
XPO7_MOUSE	Exportin-7 OS=Mus musculus OX=10090 GN=Xpo7 PE=1 SV=3	
LMNB2_MOUSE	Lamin-B2 OS=Mus musculus OX=10090 GN=Lmnb2 PE=1 SV=2	
PDS5B_MOUSE	Sister chromatid cohesion protein PDS5 homolog B OS=Mus musculus OX=10090 GN=Pds5b PE=1 SV=1	
SHLB2_MOUSE	Endophilin-B2 OS=Mus musculus OX=10090 GN=Sh3glb2 PE=1 SV=2	
IDI1_MOUSE	Isopentenyl-diphosphate Delta-isomerase 1 OS=Mus musculus OX=10090 GN=Idi1 PE=1 SV=1	
TMX3_MOUSE	Protein disulfide-isomerase TMX3 OS=Mus musculus OX=10090 GN=Tmx3 PE=1 SV=2	
TLN2_MOUSE	Talin-2 OS=Mus musculus OX=10090 GN=Tln2 PE=1 SV=3	
CUL5_MOUSE	Cullin-5 OS=Mus musculus OX=10090 GN=Cul5 PE=1 SV=3	
TPD54_MOUSE	Tumor protein D54 OS=Mus musculus OX=10090 GN=Tpd52l2 PE=1 SV=1	
PCLO_MOUSE	Protein piccolo OS=Mus musculus OX=10090 GN=Pclo PE=1 SV=4	
ABR_MOUSE	Active breakpoint cluster region-related protein OS=Mus musculus OX=10090 GN=Abr PE=1 SV=1	
PLRKT_MOUSE	Plasminogen receptor (KT) OS=Mus musculus OX=10090 GN=Plgrkt PE=1 SV=1	
GNAZ_MOUSE	Guanine nucleotide-binding protein G(z) subunit alpha OS=Mus musculus OX=10090 GN=Gnaz PE=1 SV=4	
GNAI3_MOUSE	Guanine nucleotide-binding protein G(i) subunit alpha OS=Mus musculus OX=10090 GN=Gnai3 PE=1 SV=3	
DHCR7_MOUSE	7-dehydrocholesterol reductase OS=Mus musculus OX=10090 GN=Dhcr7 PE=1 SV=1	
NMT1_MOUSE	Glycylpeptide N-tetradecanoyltransferase 1 OS=Mus musculus OX=10090 GN=Nmt1 PE=1 SV=1	
COX5B_MOUSE	Cytochrome c oxidase subunit 5B, mitochondrial OS=Mus musculus OX=10090 GN=Cox5b PE=1 SV=1	
CON__P02672	CON__P02672	
TCP4_MOUSE	Activated RNA polymerase II transcriptional coactivator p15 OS=Mus musculus OX=10090 GN=Sub1 PE=1 SV=3	
SRS10_MOUSE	Serine/arginine-rich splicing factor 10 OS=Mus musculus OX=10090 GN=Srsf10 PE=1 SV=2	
ACSF2_MOUSE	Medium-chain acyl-CoA ligase ACSF2, mitochondrial OS=Mus musculus OX=10090 GN=Acsf2 PE=1 SV=1	
MARK3_HUMAN	MAP/microtubule affinity-regulating kinase 3 OS=Homo sapiens OX=9606 GN=MARK3 PE=1 SV=5	
HPLN1_MOUSE	Hyaluronan and proteoglycan link protein 1 OS=Mus musculus OX=10090 GN=Hapln1 PE=1 SV=1	
SCPDL_MOUSE	Saccharopine dehydrogenase-like oxidoreductase OS=Mus musculus OX=10090 GN=Sccpdh PE=1 SV=1	
CD166_MOUSE	CD166 antigen OS=Mus musculus OX=10090 GN=Alcam PE=1 SV=3	
NDUAC_MOUSE	NADH dehydrogenase [ubiquinone] 1 alpha subcomplex subunit 12 OS=Mus musculus OX=10090 GN=Ndufa12 PE=1 SV=2	
RNPS1_MOUSE	RNA-binding protein with serine-rich domain 1 OS=Mus musculus OX=10090 GN=Rnps1 PE=1 SV=1	
NRDC_MOUSE	Nardilysin OS=Mus musculus OX=10090 GN=Nrdc PE=1 SV=1	
ARPC5_MOUSE	Actin-related protein 2/3 complex subunit 5 OS=Mus musculus OX=10090 GN=Arpc5 PE=1 SV=3	
SF3A3_MOUSE	Splicing factor 3A subunit 3 OS=Mus musculus OX=10090 GN=Sf3a3 PE=1 SV=2	
KV3A1_MOUSE	Ig kappa chain V-III region PC 2880/PC 1229 OS=Mus musculus OX=10090 PE=1 SV=1	
RAB21_MOUSE	Ras-related protein Rab-21 OS=Mus musculus OX=10090 GN=Rab21 PE=1 SV=4	
GDAP1_MOUSE	Ganglioside-induced differentiation-associated protein 1 OS=Mus musculus OX=10090 GN=Gdap1 PE=1 SV=1	
RDH11_MOUSE	Retinol dehydrogenase 11 OS=Mus musculus OX=10090 GN=Rdh11 PE=1 SV=2	
GPX4_MOUSE	Phospholipid hydroperoxide glutathione peroxidase OS=Mus musculus OX=10090 GN=Gpx4 PE=1 SV=4	
GFPT1_MOUSE	Glutamine--fructose-6-phosphate aminotransferase [isomerizing] 1 OS=Mus musculus OX=10090 GN=Gfpt1 PE=1 SV=3	
ARK72_MOUSE	Aflatoxin B1 aldehyde reductase member 2 OS=Mus musculus OX=10090 GN=Akr7a2 PE=1 SV=3	
NU4M_MOUSE	NADH-ubiquinone oxidoreductase chain 4 OS=Mus musculus OX=10090 GN=Mtnd4 PE=1 SV=1	
H13_MOUSE	Histone H1.3 OS=Mus musculus OX=10090 GN=Hist1h1d PE=1 SV=2	
CD81_MOUSE	CD81 antigen OS=Mus musculus OX=10090 GN=Cd81 PE=1 SV=2	
RER1_MOUSE	Protein RER1 OS=Mus musculus OX=10090 GN=Rer1 PE=1 SV=1	
ARAF_MOUSE	Serine/threonine-protein kinase A-Raf OS=Mus musculus OX=10090 GN=Araf PE=1 SV=2	
RNF14_MOUSE	E3 ubiquitin-protein ligase RNF14 OS=Mus musculus OX=10090 GN=Rnf14 PE=1 SV=2	
TXD17_MOUSE	Thioredoxin domain-containing protein 17 OS=Mus musculus OX=10090 GN=Txndc17 PE=1 SV=1	
RL35A_MOUSE	60S ribosomal protein L35a OS=Mus musculus OX=10090 GN=Rpl35a PE=1 SV=2	
SPTN2_HUMAN	Spectrin beta chain, non-erythrocytic 2 OS=Homo sapiens OX=9606 GN=SPTBN2 PE=1 SV=3	
RL37A_MOUSE	60S ribosomal protein L37a OS=Mus musculus OX=10090 GN=Rpl37a PE=1 SV=2	
EIF1_MOUSE	Eukaryotic translation initiation factor 1 OS=Mus musculus OX=10090 GN=Eif1 PE=1 SV=2	
FSD1_MOUSE	Fibronectin type III and SPRY domain-containing protein 1 OS=Mus musculus OX=10090 GN=Fsd1 PE=1 SV=1	
BIEA_MOUSE	Biliverdin reductase A OS=Mus musculus OX=10090 GN=Blvra PE=1 SV=1	
MTA1_MOUSE	Metastasis-associated protein MTA1 OS=Mus musculus OX=10090 GN=Mta1 PE=1 SV=1	
RAB12_MOUSE	Ras-related protein Rab-12 OS=Mus musculus OX=10090 GN=Rab12 PE=1 SV=3	
RRAGC_MOUSE	Ras-related GTP-binding protein C OS=Mus musculus OX=10090 GN=Rragc PE=1 SV=1	
F120C_MOUSE	Constitutive coactivator of PPAR-gamma-like protein 2 OS=Mus musculus OX=10090 GN=Fam120c PE=1 SV=3	
DRG2_MOUSE	Developmentally-regulated GTP-binding protein 2 OS=Mus musculus OX=10090 GN=Drg2 PE=1 SV=1	
PPM1G_MOUSE	Protein phosphatase 1G OS=Mus musculus OX=10090 GN=Ppm1g PE=1 SV=3	
SAP18_MOUSE	Histone deacetylase complex subunit SAP18 OS=Mus musculus OX=10090 GN=Sap18 PE=1 SV=1	
CADH2_MOUSE	Cadherin-2 OS=Mus musculus OX=10090 GN=Cdh2 PE=1 SV=2	
CSK22_MOUSE	Casein kinase II subunit alpha' OS=Mus musculus OX=10090 GN=Csnk2a2 PE=1 SV=1	
MTX1_MOUSE	Metaxin-1 OS=Mus musculus OX=10090 GN=Mtx1 PE=1 SV=1	
PUF60_MOUSE	Poly(U)-binding-splicing factor PUF60 OS=Mus musculus OX=10090 GN=Puf60 PE=1 SV=2	
SH3L1_MOUSE	SH3 domain-binding glutamic acid-rich-like protein OS=Mus musculus OX=10090 GN=Sh3bgrl PE=1 SV=1	
ATPK_MOUSE	ATP synthase subunit f, mitochondrial OS=Mus musculus OX=10090 GN=Atp5mf PE=1 SV=3	
VATG1_MOUSE	V-type proton ATPase subunit G 1 OS=Mus musculus OX=10090 GN=Atp6v1g1 PE=1 SV=3	
RS25_MOUSE	40S ribosomal protein S25 OS=Mus musculus OX=10090 GN=Rps25 PE=1 SV=1	
SCAM5_MOUSE	Secretory carrier-associated membrane protein 5 OS=Mus musculus OX=10090 GN=Scamp5 PE=1 SV=1	
GPM6B_MOUSE	Neuronal membrane glycoprotein M6-b OS=Mus musculus OX=10090 GN=Gpm6b PE=1 SV=2	
ECI2_MOUSE	Enoyl-CoA delta isomerase 2, mitochondrial OS=Mus musculus OX=10090 GN=Eci2 PE=1 SV=2	
RAB5B_MOUSE	Ras-related protein Rab-5B OS=Mus musculus OX=10090 GN=Rab5b PE=1 SV=1	
METK2_MOUSE	S-adenosylmethionine synthase isoform type-2 OS=Mus musculus OX=10090 GN=Mat2a PE=1 SV=2	
SYNPR_MOUSE	Synaptoporin OS=Mus musculus OX=10090 GN=Synpr PE=1 SV=1	
NDUB5_MOUSE	NADH dehydrogenase [ubiquinone] 1 beta subcomplex subunit 5, mitochondrial OS=Mus musculus OX=10090 GN=Ndufb5 PE=1 SV=1	
MCU_MOUSE	Calcium uniporter protein, mitochondrial OS=Mus musculus OX=10090 GN=Mcu PE=1 SV=2	
RS24_MOUSE	40S ribosomal protein S24 OS=Mus musculus OX=10090 GN=Rps24 PE=1 SV=1	
RALY_MOUSE	RNA-binding protein Raly OS=Mus musculus OX=10090 GN=Raly PE=1 SV=3	
ARXS1_MOUSE	Adipocyte-related X-chromosome expressed sequence 1 OS=Mus musculus OX=10090 GN=Arxes1 PE=1 SV=1	
MLP3A_MOUSE	Microtubule-associated proteins 1A/1B light chain 3A OS=Mus musculus OX=10090 GN=Map1lc3a PE=1 SV=1	
EMD_MOUSE	Emerin OS=Mus musculus OX=10090 GN=Emd PE=1 SV=1	
GRPE1_MOUSE	GrpE protein homolog 1, mitochondrial OS=Mus musculus OX=10090 GN=Grpel1 PE=1 SV=1	
GSTO1_MOUSE	Glutathione S-transferase omega-1 OS=Mus musculus OX=10090 GN=Gsto1 PE=1 SV=2	
SDCB1_MOUSE	Syntenin-1 OS=Mus musculus OX=10090 GN=Sdcbp PE=1 SV=1	
TM9S3_MOUSE	Transmembrane 9 superfamily member 3 OS=Mus musculus OX=10090 GN=Tm9sf3 PE=1 SV=1	
ODB2_MOUSE	Lipoamide acyltransferase component of branched-chain alpha-keto acid dehydrogenase complex, mitochondrial OS=Mus musculus OX=10090 GN=Dbt PE=1 SV=2	
DIRA2_MOUSE	GTP-binding protein Di-Ras2 OS=Mus musculus OX=10090 GN=Diras2 PE=1 SV=1	
GMFB_MOUSE	Glia maturation factor beta OS=Mus musculus OX=10090 GN=Gmfb PE=1 SV=3	
SEC13_MOUSE	Protein SEC13 homolog OS=Mus musculus OX=10090 GN=Sec13 PE=1 SV=3	
IDE_MOUSE	Insulin-degrading enzyme OS=Mus musculus OX=10090 GN=Ide PE=1 SV=1	
HSDL2_MOUSE	Hydroxysteroid dehydrogenase-like protein 2 OS=Mus musculus OX=10090 GN=Hsdl2 PE=1 SV=1	
LC7L3_MOUSE	Luc7-like protein 3 OS=Mus musculus OX=10090 GN=Luc7l3 PE=1 SV=1	
SNG1_MOUSE	Synaptogyrin-1 OS=Mus musculus OX=10090 GN=Syngr1 PE=1 SV=2	
SNX4_MOUSE	Sorting nexin-4 OS=Mus musculus OX=10090 GN=Snx4 PE=1 SV=1	
FETUA_MOUSE	Alpha-2-HS-glycoprotein OS=Mus musculus OX=10090 GN=Ahsg PE=1 SV=1	
CX6B1_MOUSE	Cytochrome c oxidase subunit 6B1 OS=Mus musculus OX=10090 GN=Cox6b1 PE=1 SV=2	
H2AX_MOUSE	Histone H2AX OS=Mus musculus OX=10090 GN=H2afx PE=1 SV=2	
ATPBM_ARATH	ATP synthase subunit beta-1, mitochondrial OS=Arabidopsis thaliana OX=3702 GN=At5g08670 PE=1 SV=1	
PEX5_MOUSE	Peroxisomal targeting signal 1 receptor OS=Mus musculus OX=10090 GN=Pex5 PE=1 SV=2	
ADPRH_MOUSE	[Protein ADP-ribosylarginine] hydrolase OS=Mus musculus OX=10090 GN=Adprh PE=1 SV=1	
SNRPA_MOUSE	U1 small nuclear ribonucleoprotein A OS=Mus musculus OX=10090 GN=Snrpa PE=1 SV=3	
PRP6_MOUSE	Pre-mRNA-processing factor 6 OS=Mus musculus OX=10090 GN=Prpf6 PE=1 SV=1	
DIP2B_MOUSE	Disco-interacting protein 2 homolog B OS=Mus musculus OX=10090 GN=Dip2b PE=1 SV=1	
PDE1B_MOUSE	Calcium/calmodulin-dependent 3',5'-cyclic nucleotide phosphodiesterase 1B OS=Mus musculus OX=10090 GN=Pde1b PE=1 SV=2	
COF2_MOUSE	Cofilin-2 OS=Mus musculus OX=10090 GN=Cfl2 PE=1 SV=1	
NAC1_MOUSE	Sodium/calcium exchanger 1 OS=Mus musculus OX=10090 GN=Slc8a1 PE=1 SV=1	
CSN7B_MOUSE	COP9 signalosome complex subunit 7b OS=Mus musculus OX=10090 GN=Cops7b PE=1 SV=1	
UBP2L_MOUSE	Ubiquitin-associated protein 2-like OS=Mus musculus OX=10090 GN=Ubap2l PE=1 SV=1	
RBP2_MOUSE	E3 SUMO-protein ligase RanBP2 OS=Mus musculus OX=10090 GN=Ranbp2 PE=1 SV=2	
PSME2_MOUSE	Proteasome activator complex subunit 2 OS=Mus musculus OX=10090 GN=Psme2 PE=1 SV=4	
NTRK2_MOUSE	BDNF/NT-3 growth factors receptor OS=Mus musculus OX=10090 GN=Ntrk2 PE=1 SV=1	
ATCAY_MOUSE	Caytaxin OS=Mus musculus OX=10090 GN=Atcay PE=1 SV=1	
CCD47_MOUSE	Coiled-coil domain-containing protein 47 OS=Mus musculus OX=10090 GN=Ccdc47 PE=1 SV=2	
NU155_MOUSE	Nuclear pore complex protein Nup155 OS=Mus musculus OX=10090 GN=Nup155 PE=1 SV=1	
ZO1_MOUSE	Tight junction protein ZO-1 OS=Mus musculus OX=10090 GN=Tjp1 PE=1 SV=2	
DYL1_MOUSE	Dynein light chain 1, cytoplasmic OS=Mus musculus OX=10090 GN=Dynll1 PE=1 SV=1	
NCBP1_MOUSE	Nuclear cap-binding protein subunit 1 OS=Mus musculus OX=10090 GN=Ncbp1 PE=1 SV=2	
GPSM1_MOUSE	G-protein-signaling modulator 1 OS=Mus musculus OX=10090 GN=Gpsm1 PE=1 SV=3	
L1CAM_MOUSE	Neural cell adhesion molecule L1 OS=Mus musculus OX=10090 GN=L1cam PE=1 SV=1	
ACACA_MOUSE	Acetyl-CoA carboxylase 1 OS=Mus musculus OX=10090 GN=Acaca PE=1 SV=1	
ERF3A_MOUSE	Eukaryotic peptide chain release factor GTP-binding subunit ERF3A OS=Mus musculus OX=10090 GN=Gspt1 PE=1 SV=2	
NDUB8_MOUSE	NADH dehydrogenase [ubiquinone] 1 beta subcomplex subunit 8, mitochondrial OS=Mus musculus OX=10090 GN=Ndufb8 PE=1 SV=1	
DTD1_MOUSE	D-aminoacyl-tRNA deacylase 1 OS=Mus musculus OX=10090 GN=Dtd1 PE=1 SV=2	
VATG2_MOUSE	V-type proton ATPase subunit G 2 OS=Mus musculus OX=10090 GN=Atp6v1g2 PE=1 SV=1	
ARL8A_MOUSE	ADP-ribosylation factor-like protein 8A OS=Mus musculus OX=10090 GN=Arl8a PE=1 SV=1	
SF01_MOUSE	Splicing factor 1 OS=Mus musculus OX=10090 GN=Sf1 PE=1 SV=6	
UGPA_MOUSE	UTP--glucose-1-phosphate uridylyltransferase OS=Mus musculus OX=10090 GN=Ugp2 PE=1 SV=3	
COX6C_MOUSE	Cytochrome c oxidase subunit 6C OS=Mus musculus OX=10090 GN=Cox6c PE=1 SV=3	
VTI1B_MOUSE	Vesicle transport through interaction with t-SNAREs homolog 1B OS=Mus musculus OX=10090 GN=Vti1b PE=1 SV=1	
FLOT1_MOUSE	Flotillin-1 OS=Mus musculus OX=10090 GN=Flot1 PE=1 SV=1	
SUCB2_MOUSE	Succinate--CoA ligase [GDP-forming] subunit beta, mitochondrial OS=Mus musculus OX=10090 GN=Suclg2 PE=1 SV=3	
DCTN3_MOUSE	Dynactin subunit 3 OS=Mus musculus OX=10090 GN=Dctn3 PE=1 SV=2	
TBB6_MOUSE	Tubulin beta-6 chain OS=Mus musculus OX=10090 GN=Tubb6 PE=1 SV=1	
UB2L3_MOUSE	Ubiquitin-conjugating enzyme E2 L3 OS=Mus musculus OX=10090 GN=Ube2l3 PE=1 SV=1	
KC1A_MOUSE	Casein kinase I isoform alpha OS=Mus musculus OX=10090 GN=Csnk1a1 PE=1 SV=2	
KCC2G_MOUSE	Calcium/calmodulin-dependent protein kinase type II subunit gamma OS=Mus musculus OX=10090 GN=Camk2g PE=1 SV=1	
SRSF5_HUMAN	Serine/arginine-rich splicing factor 5 OS=Homo sapiens OX=9606 GN=SRSF5 PE=1 SV=1	
ELAV4_MOUSE	ELAV-like protein 4 OS=Mus musculus OX=10090 GN=Elavl4 PE=1 SV=1	
BCAT1_MOUSE	Branched-chain-amino-acid aminotransferase, cytosolic OS=Mus musculus OX=10090 GN=Bcat1 PE=1 SV=2	
HVM44_MOUSE	Ig heavy chain V region PJ14 OS=Mus musculus OX=10090 PE=1 SV=1	
LMAN1_MOUSE	Protein ERGIC-53 OS=Mus musculus OX=10090 GN=Lman1 PE=1 SV=1	
TTL12_MOUSE	Tubulin--tyrosine ligase-like protein 12 OS=Mus musculus OX=10090 GN=Ttll12 PE=1 SV=1	
GLYR1_MOUSE	Putative oxidoreductase GLYR1 OS=Mus musculus OX=10090 GN=Glyr1 PE=1 SV=1	
MTCH1_MOUSE	Mitochondrial carrier homolog 1 OS=Mus musculus OX=10090 GN=Mtch1 PE=1 SV=1	
PFD3_MOUSE	Prefoldin subunit 3 OS=Mus musculus OX=10090 GN=Vbp1 PE=1 SV=2	
ULA1_MOUSE	NEDD8-activating enzyme E1 regulatory subunit OS=Mus musculus OX=10090 GN=Nae1 PE=1 SV=1	
PCYOX_MOUSE	Prenylcysteine oxidase OS=Mus musculus OX=10090 GN=Pcyox1 PE=1 SV=1	
FXR1_MOUSE	Fragile X mental retardation syndrome-related protein 1 OS=Mus musculus OX=10090 GN=Fxr1 PE=1 SV=2	
PI4KA_MOUSE	Phosphatidylinositol 4-kinase alpha OS=Mus musculus OX=10090 GN=Pi4ka PE=1 SV=2	
NDUB4_MOUSE	NADH dehydrogenase [ubiquinone] 1 beta subcomplex subunit 4 OS=Mus musculus OX=10090 GN=Ndufb4 PE=1 SV=3	
TXTP_MOUSE	Tricarboxylate transport protein, mitochondrial OS=Mus musculus OX=10090 GN=Slc25a1 PE=1 SV=1	
OXR1_MOUSE	Oxidation resistance protein 1 OS=Mus musculus OX=10090 GN=Oxr1 PE=1 SV=3	
VPS51_MOUSE	Vacuolar protein sorting-associated protein 51 homolog OS=Mus musculus OX=10090 GN=Vps51 PE=1 SV=2	
NRCAM_MOUSE	Neuronal cell adhesion molecule OS=Mus musculus OX=10090 GN=Nrcam PE=1 SV=2	
DJB11_MOUSE	DnaJ homolog subfamily B member 11 OS=Mus musculus OX=10090 GN=Dnajb11 PE=1 SV=1	
TRNT1_MOUSE	CCA tRNA nucleotidyltransferase 1, mitochondrial OS=Mus musculus OX=10090 GN=Trnt1 PE=1 SV=1	
HPCA_HUMAN	Neuron-specific calcium-binding protein hippocalcin OS=Homo sapiens OX=9606 GN=HPCA PE=1 SV=2	
STMN3_MOUSE	Stathmin-3 OS=Mus musculus OX=10090 GN=Stmn3 PE=1 SV=1	
S4A4_MOUSE	Electrogenic sodium bicarbonate cotransporter 1 OS=Mus musculus OX=10090 GN=Slc4a4 PE=1 SV=2	
RL26_MOUSE	60S ribosomal protein L26 OS=Mus musculus OX=10090 GN=Rpl26 PE=1 SV=1	
AIMP1_MOUSE	Aminoacyl tRNA synthase complex-interacting multifunctional protein 1 OS=Mus musculus OX=10090 GN=Aimp1 PE=1 SV=2	
HIBCH_MOUSE	3-hydroxyisobutyryl-CoA hydrolase, mitochondrial OS=Mus musculus OX=10090 GN=Hibch PE=1 SV=1	
AT1B3_MOUSE	Sodium/potassium-transporting ATPase subunit beta-3 OS=Mus musculus OX=10090 GN=Atp1b3 PE=1 SV=1	
AFG32_MOUSE	AFG3-like protein 2 OS=Mus musculus OX=10090 GN=Afg3l2 PE=1 SV=1	
SRPK2_MOUSE	SRSF protein kinase 2 OS=Mus musculus OX=10090 GN=Srpk2 PE=1 SV=2	
ACINU_MOUSE	Apoptotic chromatin condensation inducer in the nucleus OS=Mus musculus OX=10090 GN=Acin1 PE=1 SV=3	
MCCA_MOUSE	Methylcrotonoyl-CoA carboxylase subunit alpha, mitochondrial OS=Mus musculus OX=10090 GN=Mccc1 PE=1 SV=2	
RHG35_MOUSE	Rho GTPase-activating protein 35 OS=Mus musculus OX=10090 GN=Arhgap35 PE=1 SV=3	
2A5G_MOUSE	Serine/threonine-protein phosphatase 2A 56 kDa regulatory subunit gamma isoform OS=Mus musculus OX=10090 GN=Ppp2r5c PE=1 SV=2	
SEM3C_MOUSE	Semaphorin-3C OS=Mus musculus OX=10090 GN=Sema3c PE=1 SV=2	
CNOT1_HUMAN	CCR4-NOT transcription complex subunit 1 OS=Homo sapiens OX=9606 GN=CNOT1 PE=1 SV=2	
NDUAD_MOUSE	NADH dehydrogenase [ubiquinone] 1 alpha subcomplex subunit 13 OS=Mus musculus OX=10090 GN=Ndufa13 PE=1 SV=3	
HA11_MOUSE	H-2 class I histocompatibility antigen, D-B alpha chain OS=Mus musculus OX=10090 GN=H2-D1 PE=1 SV=2	
LAMP1_MOUSE	Lysosome-associated membrane glycoprotein 1 OS=Mus musculus OX=10090 GN=Lamp1 PE=1 SV=2	
CON__P07477	CON__P07477	
UBC9_MOUSE	SUMO-conjugating enzyme UBC9 OS=Mus musculus OX=10090 GN=Ube2i PE=1 SV=1	
NNRE_MOUSE	NAD(P)H-hydrate epimerase OS=Mus musculus OX=10090 GN=Naxe PE=1 SV=1	
RM12_MOUSE	39S ribosomal protein L12, mitochondrial OS=Mus musculus OX=10090 GN=Mrpl12 PE=1 SV=2	
RU1C_MOUSE	U1 small nuclear ribonucleoprotein C OS=Mus musculus OX=10090 GN=Snrpc PE=1 SV=1	
HMGB2_MOUSE	High mobility group protein B2 OS=Mus musculus OX=10090 GN=Hmgb2 PE=1 SV=3	
PPR21_MOUSE	Protein phosphatase 1 regulatory subunit 21 OS=Mus musculus OX=10090 GN=Ppp1r21 PE=1 SV=2	
MSI2H_MOUSE	RNA-binding protein Musashi homolog 2 OS=Mus musculus OX=10090 GN=Msi2 PE=1 SV=1	
ACBP_MOUSE	Acyl-CoA-binding protein OS=Mus musculus OX=10090 GN=Dbi PE=1 SV=2	
UBXN1_MOUSE	UBX domain-containing protein 1 OS=Mus musculus OX=10090 GN=Ubxn1 PE=1 SV=1	
RSMB_MOUSE	Small nuclear ribonucleoprotein-associated protein B OS=Mus musculus OX=10090 GN=Snrpb PE=1 SV=1	
AT2B3_HUMAN	Plasma membrane calcium-transporting ATPase 3 OS=Homo sapiens OX=9606 GN=ATP2B3 PE=1 SV=3	
STMN2_MOUSE	Stathmin-2 OS=Mus musculus OX=10090 GN=Stmn2 PE=1 SV=1	
MYLK_MOUSE	Myosin light chain kinase, smooth muscle OS=Mus musculus OX=10090 GN=Mylk PE=1 SV=3	
BCLF1_MOUSE	Bcl-2-associated transcription factor 1 OS=Mus musculus OX=10090 GN=Bclaf1 PE=1 SV=2	
IMA4_MOUSE	Importin subunit alpha-4 OS=Mus musculus OX=10090 GN=Kpna3 PE=1 SV=1	
MT3_MOUSE	Metallothionein-3 OS=Mus musculus OX=10090 GN=Mt3 PE=1 SV=1	
LEGL_MOUSE	Galectin-related protein OS=Mus musculus OX=10090 GN=Lgalsl PE=1 SV=1	
RO60_MOUSE	60 kDa SS-A/Ro ribonucleoprotein OS=Mus musculus OX=10090 GN=RO60 PE=1 SV=1	
COPZ1_MOUSE	Coatomer subunit zeta-1 OS=Mus musculus OX=10090 GN=Copz1 PE=1 SV=1	
THTR_MOUSE	Thiosulfate sulfurtransferase OS=Mus musculus OX=10090 GN=Tst PE=1 SV=3	
GBG7_MOUSE	Guanine nucleotide-binding protein G(I)/G(S)/G(O) subunit gamma-7 OS=Mus musculus OX=10090 GN=Gng7 PE=1 SV=2	
SRRM1_MOUSE	Serine/arginine repetitive matrix protein 1 OS=Mus musculus OX=10090 GN=Srrm1 PE=1 SV=2	
DOCK7_MOUSE	Dedicator of cytokinesis protein 7 OS=Mus musculus OX=10090 GN=Dock7 PE=1 SV=3	
PPM1A_MOUSE	Protein phosphatase 1A OS=Mus musculus OX=10090 GN=Ppm1a PE=1 SV=1	
NMDZ1_MOUSE	Glutamate receptor ionotropic, NMDA 1 OS=Mus musculus OX=10090 GN=Grin1 PE=1 SV=1	
ATLA2_MOUSE	Atlastin-2 OS=Mus musculus OX=10090 GN=Atl2 PE=1 SV=1	
TM9S4_MOUSE	Transmembrane 9 superfamily member 4 OS=Mus musculus OX=10090 GN=Tm9sf4 PE=1 SV=1	
IMPA1_MOUSE	Inositol monophosphatase 1 OS=Mus musculus OX=10090 GN=Impa1 PE=1 SV=1	
RT23_MOUSE	28S ribosomal protein S23, mitochondrial OS=Mus musculus OX=10090 GN=Mrps23 PE=1 SV=1	
TTYH3_MOUSE	Protein tweety homolog 3 OS=Mus musculus OX=10090 GN=Ttyh3 PE=1 SV=1	
ZFR_MOUSE	Zinc finger RNA-binding protein OS=Mus musculus OX=10090 GN=Zfr PE=1 SV=2	
SRPRB_MOUSE	Signal recognition particle receptor subunit beta OS=Mus musculus OX=10090 GN=Srprb PE=1 SV=1	
MCES_MOUSE	mRNA cap guanine-N7 methyltransferase OS=Mus musculus OX=10090 GN=Rnmt PE=1 SV=1	
TIAR_MOUSE	Nucleolysin TIAR OS=Mus musculus OX=10090 GN=Tial1 PE=1 SV=1	
KAP0_MOUSE	cAMP-dependent protein kinase type I-alpha regulatory subunit OS=Mus musculus OX=10090 GN=Prkar1a PE=1 SV=3	
PIGS_MOUSE	GPI transamidase component PIG-S OS=Mus musculus OX=10090 GN=Pigs PE=1 SV=3	
ARP10_MOUSE	Actin-related protein 10 OS=Mus musculus OX=10090 GN=Actr10 PE=1 SV=2	
ANM8_MOUSE	Protein arginine N-methyltransferase 8 OS=Mus musculus OX=10090 GN=Prmt8 PE=1 SV=2	
BRK1_MOUSE	Protein BRICK1 OS=Mus musculus OX=10090 GN=Brk1 PE=1 SV=1	
RPGP1_MOUSE	Rap1 GTPase-activating protein 1 OS=Mus musculus OX=10090 GN=Rap1gap PE=1 SV=2	
CP131_MOUSE	Centrosomal protein of 131 kDa OS=Mus musculus OX=10090 GN=Cep131 PE=1 SV=2	
HCFC1_MOUSE	Host cell factor 1 OS=Mus musculus OX=10090 GN=Hcfc1 PE=1 SV=2	
ADRM1_MOUSE	Proteasomal ubiquitin receptor ADRM1 OS=Mus musculus OX=10090 GN=Adrm1 PE=1 SV=2	
STRN4_MOUSE	Striatin-4 OS=Mus musculus OX=10090 GN=Strn4 PE=1 SV=2	
CAP2_MOUSE	Adenylyl cyclase-associated protein 2 OS=Mus musculus OX=10090 GN=Cap2 PE=1 SV=1	
CADM4_MOUSE	Cell adhesion molecule 4 OS=Mus musculus OX=10090 GN=Cadm4 PE=1 SV=1	
RBX1_MOUSE	E3 ubiquitin-protein ligase RBX1 OS=Mus musculus OX=10090 GN=Rbx1 PE=1 SV=1	
DENR_MOUSE	Density-regulated protein OS=Mus musculus OX=10090 GN=Denr PE=1 SV=1	
NH2L1_MOUSE	NHP2-like protein 1 OS=Mus musculus OX=10090 GN=Snu13 PE=1 SV=4	
LYPA1_MOUSE	Acyl-protein thioesterase 1 OS=Mus musculus OX=10090 GN=Lypla1 PE=1 SV=1	
NDUA7_MOUSE	NADH dehydrogenase [ubiquinone] 1 alpha subcomplex subunit 7 OS=Mus musculus OX=10090 GN=Ndufa7 PE=1 SV=3	
TIM50_MOUSE	Mitochondrial import inner membrane translocase subunit TIM50 OS=Mus musculus OX=10090 GN=Timm50 PE=1 SV=1	
CDIPT_MOUSE	CDP-diacylglycerol--inositol 3-phosphatidyltransferase OS=Mus musculus OX=10090 GN=Cdipt PE=1 SV=1	
EVL_MOUSE	Ena/VASP-like protein OS=Mus musculus OX=10090 GN=Evl PE=1 SV=2	
NUDT3_MOUSE	Diphosphoinositol polyphosphate phosphohydrolase 1 OS=Mus musculus OX=10090 GN=Nudt3 PE=1 SV=1	
SODC_MOUSE	Superoxide dismutase [Cu-Zn] OS=Mus musculus OX=10090 GN=Sod1 PE=1 SV=2	
EFTS_MOUSE	Elongation factor Ts, mitochondrial OS=Mus musculus OX=10090 GN=Tsfm PE=1 SV=1	
SMU1_MOUSE	WD40 repeat-containing protein SMU1 OS=Mus musculus OX=10090 GN=Smu1 PE=2 SV=2	
RS26_MOUSE	40S ribosomal protein S26 OS=Mus musculus OX=10090 GN=Rps26 PE=1 SV=3	
RS51_ARATH	40S ribosomal protein S5-1 OS=Arabidopsis thaliana OX=3702 GN=RPS5A PE=1 SV=1	
IGHA_MOUSE	Ig alpha chain C region OS=Mus musculus OX=10090 PE=1 SV=1	
TYB4_RAT	Thymosin beta-4 OS=Rattus norvegicus OX=10116 GN=Tmsb4x PE=1 SV=2	
VPS36_MOUSE	Vacuolar protein-sorting-associated protein 36 OS=Mus musculus OX=10090 GN=Vps36 PE=1 SV=1	
BPHL_MOUSE	Valacyclovir hydrolase OS=Mus musculus OX=10090 GN=Bphl PE=1 SV=1	
P35969-DECOY	P35969	
ELP3_MOUSE	Elongator complex protein 3 OS=Mus musculus OX=10090 GN=Elp3 PE=1 SV=1	
CHRD1_MOUSE	Cysteine and histidine-rich domain-containing protein 1 OS=Mus musculus OX=10090 GN=Chordc1 PE=1 SV=1	
PUR8_MOUSE	Adenylosuccinate lyase OS=Mus musculus OX=10090 GN=Adsl PE=1 SV=2	
COPE_MOUSE	Coatomer subunit epsilon OS=Mus musculus OX=10090 GN=Cope PE=1 SV=3	
EI2BA_MOUSE	Translation initiation factor eIF-2B subunit alpha OS=Mus musculus OX=10090 GN=Eif2b1 PE=1 SV=1	
SAHH3_MOUSE	Putative adenosylhomocysteinase 3 OS=Mus musculus OX=10090 GN=Ahcyl2 PE=1 SV=1	
ABCF1_MOUSE	ATP-binding cassette sub-family F member 1 OS=Mus musculus OX=10090 GN=Abcf1 PE=1 SV=1	
MYH14_MOUSE	Myosin-14 OS=Mus musculus OX=10090 GN=Myh14 PE=1 SV=1	
Q61644-DECOY	Q61644	
RCC2_MOUSE	Protein RCC2 OS=Mus musculus OX=10090 GN=Rcc2 PE=1 SV=1	
GSHR_MOUSE	Glutathione reductase, mitochondrial OS=Mus musculus OX=10090 GN=Gsr PE=1 SV=3	
VPS28_MOUSE	Vacuolar protein sorting-associated protein 28 homolog OS=Mus musculus OX=10090 GN=Vps28 PE=1 SV=1	
MAOM_MOUSE	NAD-dependent malic enzyme, mitochondrial OS=Mus musculus OX=10090 GN=Me2 PE=1 SV=1	
SMCA4_MOUSE	Transcription activator BRG1 OS=Mus musculus OX=10090 GN=Smarca4 PE=1 SV=1	
VPS45_MOUSE	Vacuolar protein sorting-associated protein 45 OS=Mus musculus OX=10090 GN=Vps45 PE=1 SV=1	
FRIL1_MOUSE	Ferritin light chain 1 OS=Mus musculus OX=10090 GN=Ftl1 PE=1 SV=2	
SRGP3_MOUSE	SLIT-ROBO Rho GTPase-activating protein 3 OS=Mus musculus OX=10090 GN=Srgap3 PE=1 SV=1	
PGCA_MOUSE	Aggrecan core protein OS=Mus musculus OX=10090 GN=Acan PE=1 SV=2	
EI3JA_MOUSE	Eukaryotic translation initiation factor 3 subunit J-A OS=Mus musculus OX=10090 GN=Eif3j1 PE=2 SV=1	
PI51C_MOUSE	Phosphatidylinositol 4-phosphate 5-kinase type-1 gamma OS=Mus musculus OX=10090 GN=Pip5k1c PE=1 SV=2	
DAD1_MOUSE	Dolichyl-diphosphooligosaccharide--protein glycosyltransferase subunit DAD1 OS=Mus musculus OX=10090 GN=Dad1 PE=1 SV=3	
SRP54_MOUSE	Signal recognition particle 54 kDa protein OS=Mus musculus OX=10090 GN=Srp54 PE=1 SV=2	
DD19A_MOUSE	ATP-dependent RNA helicase DDX19A OS=Mus musculus OX=10090 GN=Ddx19a PE=1 SV=2	
TBCB_MOUSE	Tubulin-folding cofactor B OS=Mus musculus OX=10090 GN=Tbcb PE=1 SV=2	
PDLI5_MOUSE	PDZ and LIM domain protein 5 OS=Mus musculus OX=10090 GN=Pdlim5 PE=1 SV=4	
RBM25_MOUSE	RNA-binding protein 25 OS=Mus musculus OX=10090 GN=Rbm25 PE=1 SV=2	
IF5_MOUSE	Eukaryotic translation initiation factor 5 OS=Mus musculus OX=10090 GN=Eif5 PE=1 SV=1	
CON__P78385	CON__P78385	
VAS1_MOUSE	V-type proton ATPase subunit S1 OS=Mus musculus OX=10090 GN=Atp6ap1 PE=1 SV=1	
REEP5_MOUSE	Receptor expression-enhancing protein 5 OS=Mus musculus OX=10090 GN=Reep5 PE=1 SV=1	
NDUA2_MOUSE	NADH dehydrogenase [ubiquinone] 1 alpha subcomplex subunit 2 OS=Mus musculus OX=10090 GN=Ndufa2 PE=1 SV=3	
PPAC_MOUSE	Low molecular weight phosphotyrosine protein phosphatase OS=Mus musculus OX=10090 GN=Acp1 PE=1 SV=3	
RRAS2_MOUSE	Ras-related protein R-Ras2 OS=Mus musculus OX=10090 GN=Rras2 PE=1 SV=1	
RBBP7_MOUSE	Histone-binding protein RBBP7 OS=Mus musculus OX=10090 GN=Rbbp7 PE=1 SV=1	
SSRD_MOUSE	Translocon-associated protein subunit delta OS=Mus musculus OX=10090 GN=Ssr4 PE=1 SV=1	
TBCD_MOUSE	Tubulin-specific chaperone D OS=Mus musculus OX=10090 GN=Tbcd PE=1 SV=1	
RBM8A_MOUSE	RNA-binding protein 8A OS=Mus musculus OX=10090 GN=Rbm8a PE=1 SV=4	
CN37_MOUSE	2',3'-cyclic-nucleotide 3'-phosphodiesterase OS=Mus musculus OX=10090 GN=Cnp PE=1 SV=3	
PTMS_MOUSE	Parathymosin OS=Mus musculus OX=10090 GN=Ptms PE=1 SV=3	
ACPM_MOUSE	Acyl carrier protein, mitochondrial OS=Mus musculus OX=10090 GN=Ndufab1 PE=1 SV=1	
ITM2C_MOUSE	Integral membrane protein 2C OS=Mus musculus OX=10090 GN=Itm2c PE=1 SV=2	
MIC27_MOUSE	MICOS complex subunit Mic27 OS=Mus musculus OX=10090 GN=Apool PE=1 SV=1	
PLXA1_MOUSE	Plexin-A1 OS=Mus musculus OX=10090 GN=Plxna1 PE=1 SV=1	
H14_MOUSE	Histone H1.4 OS=Mus musculus OX=10090 GN=Hist1h1e PE=1 SV=2	
SC61B_MOUSE	Protein transport protein Sec61 subunit beta OS=Mus musculus OX=10090 GN=Sec61b PE=1 SV=3	
AP1M1_MOUSE	AP-1 complex subunit mu-1 OS=Mus musculus OX=10090 GN=Ap1m1 PE=1 SV=3	
PACN2_MOUSE	Protein kinase C and casein kinase substrate in neurons protein 2 OS=Mus musculus OX=10090 GN=Pacsin2 PE=1 SV=1	
XRN2_MOUSE	5'-3' exoribonuclease 2 OS=Mus musculus OX=10090 GN=Xrn2 PE=1 SV=1	
RS28_MOUSE	40S ribosomal protein S28 OS=Mus musculus OX=10090 GN=Rps28 PE=1 SV=1	
UB2D2_MOUSE	Ubiquitin-conjugating enzyme E2 D2 OS=Mus musculus OX=10090 GN=Ube2d2 PE=1 SV=1	
AN32B_MOUSE	Acidic leucine-rich nuclear phosphoprotein 32 family member B OS=Mus musculus OX=10090 GN=Anp32b PE=1 SV=1	
PABP2_MOUSE	Polyadenylate-binding protein 2 OS=Mus musculus OX=10090 GN=Pabpn1 PE=1 SV=3	
QCR9_MOUSE	Cytochrome b-c1 complex subunit 9 OS=Mus musculus OX=10090 GN=Uqcr10 PE=1 SV=1	
UBA6_MOUSE	Ubiquitin-like modifier-activating enzyme 6 OS=Mus musculus OX=10090 GN=Uba6 PE=1 SV=1	
WDR47_MOUSE	WD repeat-containing protein 47 OS=Mus musculus OX=10090 GN=Wdr47 PE=1 SV=2	
RM37_MOUSE	39S ribosomal protein L37, mitochondrial OS=Mus musculus OX=10090 GN=Mrpl37 PE=1 SV=1	
KLC2_MOUSE	Kinesin light chain 2 OS=Mus musculus OX=10090 GN=Klc2 PE=1 SV=1	
MESD_MOUSE	LRP chaperone MESD OS=Mus musculus OX=10090 GN=Mesd PE=1 SV=1	
FRIH_MOUSE	Ferritin heavy chain OS=Mus musculus OX=10090 GN=Fth1 PE=1 SV=2	
MPP6_MOUSE	MAGUK p55 subfamily member 6 OS=Mus musculus OX=10090 GN=Mpp6 PE=1 SV=1	
EIF3M_MOUSE	Eukaryotic translation initiation factor 3 subunit M OS=Mus musculus OX=10090 GN=Eif3m PE=1 SV=1	
MYCT_MOUSE	Proton myo-inositol cotransporter OS=Mus musculus OX=10090 GN=Slc2a13 PE=1 SV=2	
NFASC_MOUSE	Neurofascin OS=Mus musculus OX=10090 GN=Nfasc PE=1 SV=1	
PTPRS_MOUSE	Receptor-type tyrosine-protein phosphatase S OS=Mus musculus OX=10090 GN=Ptprs PE=1 SV=1	
NAKD2_MOUSE	NAD kinase 2, mitochondrial OS=Mus musculus OX=10090 GN=Nadk2 PE=1 SV=2	
ACADS_MOUSE	Short-chain specific acyl-CoA dehydrogenase, mitochondrial OS=Mus musculus OX=10090 GN=Acads PE=1 SV=2	
CON__P02070	CON__P02070	
HOOK3_MOUSE	Protein Hook homolog 3 OS=Mus musculus OX=10090 GN=Hook3 PE=1 SV=2	
F162A_MOUSE	Protein FAM162A OS=Mus musculus OX=10090 GN=Fam162a PE=1 SV=1	
NAV1_MOUSE	Neuron navigator 1 OS=Mus musculus OX=10090 GN=Nav1 PE=1 SV=2	
TIP_MOUSE	T-cell immunomodulatory protein OS=Mus musculus OX=10090 GN=Itfg1 PE=1 SV=2	
PP6R3_MOUSE	Serine/threonine-protein phosphatase 6 regulatory subunit 3 OS=Mus musculus OX=10090 GN=Ppp6r3 PE=1 SV=1	
GRM5_MOUSE	Metabotropic glutamate receptor 5 OS=Mus musculus OX=10090 GN=Grm5 PE=1 SV=2	
CON__ENSEMBL:ENSBTAP00000024146	CON__ENSEMBL:ENSBTAP00000024146	
PLST_MOUSE	Plastin-3 OS=Mus musculus OX=10090 GN=Pls3 PE=1 SV=3	
TS101_MOUSE	Tumor susceptibility gene 101 protein OS=Mus musculus OX=10090 GN=Tsg101 PE=1 SV=2	
MADD_HUMAN	MAP kinase-activating death domain protein OS=Homo sapiens OX=9606 GN=MADD PE=1 SV=2	
CPNE2_MOUSE	Copine-2 OS=Mus musculus OX=10090 GN=Cpne2 PE=1 SV=1	
PSMD9_MOUSE	26S proteasome non-ATPase regulatory subunit 9 OS=Mus musculus OX=10090 GN=Psmd9 PE=1 SV=1	
VAT1L_MOUSE	Synaptic vesicle membrane protein VAT-1 homolog-like OS=Mus musculus OX=10090 GN=Vat1l PE=1 SV=2	
EXOC4_MOUSE	Exocyst complex component 4 OS=Mus musculus OX=10090 GN=Exoc4 PE=1 SV=2	
PARP1_MOUSE	Poly [ADP-ribose] polymerase 1 OS=Mus musculus OX=10090 GN=Parp1 PE=1 SV=3	
TBC24_MOUSE	TBC1 domain family member 24 OS=Mus musculus OX=10090 GN=Tbc1d24 PE=1 SV=2	
CUL1_MOUSE	Cullin-1 OS=Mus musculus OX=10090 GN=Cul1 PE=1 SV=1	
AOXA_MOUSE	Aldehyde oxidase 1 OS=Mus musculus OX=10090 GN=Aox1 PE=1 SV=2	
KIF3B_MOUSE	Kinesin-like protein KIF3B OS=Mus musculus OX=10090 GN=Kif3b PE=1 SV=1	
PPCEL_MOUSE	Prolyl endopeptidase-like OS=Mus musculus OX=10090 GN=Prepl PE=1 SV=1	
DCTN4_MOUSE	Dynactin subunit 4 OS=Mus musculus OX=10090 GN=Dctn4 PE=1 SV=1	
APOB_MOUSE	Apolipoprotein B-100 OS=Mus musculus OX=10090 GN=Apob PE=1 SV=1	
DUS3_MOUSE	Dual specificity protein phosphatase 3 OS=Mus musculus OX=10090 GN=Dusp3 PE=1 SV=1	
RL35_MOUSE	60S ribosomal protein L35 OS=Mus musculus OX=10090 GN=Rpl35 PE=1 SV=1	
ZN207_MOUSE	BUB3-interacting and GLEBS motif-containing protein ZNF207 OS=Mus musculus OX=10090 GN=Znf207 PE=1 SV=1	
ATPMD_MOUSE	ATP synthase membrane subunit DAPIT, mitochondrial OS=Mus musculus OX=10090 GN=Atp5md PE=1 SV=1	
MIC25_MOUSE	MICOS complex subunit Mic25 OS=Mus musculus OX=10090 GN=Chchd6 PE=1 SV=2	
MAGD1_MOUSE	Melanoma-associated antigen D1 OS=Mus musculus OX=10090 GN=Maged1 PE=1 SV=1	
PRAF2_MOUSE	PRA1 family protein 2 OS=Mus musculus OX=10090 GN=Praf2 PE=1 SV=1	
AP3S1_MOUSE	AP-3 complex subunit sigma-1 OS=Mus musculus OX=10090 GN=Ap3s1 PE=1 SV=2	
HVM18_MOUSE	Ig heavy chain V regions TEPC 15/S107/HPCM1/HPCM2/HPCM3 OS=Mus musculus OX=10090 PE=1 SV=1	
MBOA7_MOUSE	Lysophospholipid acyltransferase 7 OS=Mus musculus OX=10090 GN=Mboat7 PE=1 SV=1	
STAM1_MOUSE	Signal transducing adapter molecule 1 OS=Mus musculus OX=10090 GN=Stam PE=1 SV=3	
CON__Q86YZ3	CON__Q86YZ3	
KPCB_MOUSE	Protein kinase C beta type OS=Mus musculus OX=10090 GN=Prkcb PE=1 SV=4	
KCAB2_MOUSE	Voltage-gated potassium channel subunit beta-2 OS=Mus musculus OX=10090 GN=Kcnab2 PE=1 SV=1	
SQSTM_MOUSE	Sequestosome-1 OS=Mus musculus OX=10090 GN=Sqstm1 PE=1 SV=1	
S38A3_MOUSE	Sodium-coupled neutral amino acid transporter 3 OS=Mus musculus OX=10090 GN=Slc38a3 PE=1 SV=1	
M4K4_MOUSE	Mitogen-activated protein kinase kinase kinase kinase 4 OS=Mus musculus OX=10090 GN=Map4k4 PE=1 SV=1	
ARF6_MOUSE	ADP-ribosylation factor 6 OS=Mus musculus OX=10090 GN=Arf6 PE=1 SV=2	
SLTM_MOUSE	SAFB-like transcription modulator OS=Mus musculus OX=10090 GN=Sltm PE=1 SV=1	
DPY30_MOUSE	Protein dpy-30 homolog OS=Mus musculus OX=10090 GN=Dpy30 PE=1 SV=1	
UMPS_MOUSE	Uridine 5'-monophosphate synthase OS=Mus musculus OX=10090 GN=Umps PE=1 SV=3	
MUTA_MOUSE	Methylmalonyl-CoA mutase, mitochondrial OS=Mus musculus OX=10090 GN=Mmut PE=1 SV=2	
RT29_MOUSE	28S ribosomal protein S29, mitochondrial OS=Mus musculus OX=10090 GN=Dap3 PE=1 SV=1	
MFN2_MOUSE	Mitofusin-2 OS=Mus musculus OX=10090 GN=Mfn2 PE=1 SV=3	
ITM2B_MOUSE	Integral membrane protein 2B OS=Mus musculus OX=10090 GN=Itm2b PE=1 SV=1	
EPN1_MOUSE	Epsin-1 OS=Mus musculus OX=10090 GN=Epn1 PE=1 SV=3	
DDX21_MOUSE	Nucleolar RNA helicase 2 OS=Mus musculus OX=10090 GN=Ddx21 PE=1 SV=3	
CSN5_MOUSE	COP9 signalosome complex subunit 5 OS=Mus musculus OX=10090 GN=Cops5 PE=1 SV=3	
GPX1_MOUSE	Glutathione peroxidase 1 OS=Mus musculus OX=10090 GN=Gpx1 PE=1 SV=2	
ENSA_MOUSE	Alpha-endosulfine OS=Mus musculus OX=10090 GN=Ensa PE=1 SV=1	
ACSL6_MOUSE	Long-chain-fatty-acid--CoA ligase 6 OS=Mus musculus OX=10090 GN=Acsl6 PE=1 SV=1	
THTM_MOUSE	3-mercaptopyruvate sulfurtransferase OS=Mus musculus OX=10090 GN=Mpst PE=1 SV=4	
RBM3_MOUSE	RNA-binding protein 3 OS=Mus musculus OX=10090 GN=Rbm3 PE=1 SV=1	
HMOX2_MOUSE	Heme oxygenase 2 OS=Mus musculus OX=10090 GN=Hmox2 PE=1 SV=1	
KGUA_MOUSE	Guanylate kinase OS=Mus musculus OX=10090 GN=Guk1 PE=1 SV=2	
MAGI1_MOUSE	Membrane-associated guanylate kinase, WW and PDZ domain-containing protein 1 OS=Mus musculus OX=10090 GN=Magi1 PE=1 SV=1	
S6A17_MOUSE	Sodium-dependent neutral amino acid transporter SLC6A17 OS=Mus musculus OX=10090 GN=Slc6a17 PE=1 SV=1	
ARL2_MOUSE	ADP-ribosylation factor-like protein 2 OS=Mus musculus OX=10090 GN=Arl2 PE=1 SV=1	
VAPB_MOUSE	Vesicle-associated membrane protein-associated protein B OS=Mus musculus OX=10090 GN=Vapb PE=1 SV=3	
SART3_MOUSE	Squamous cell carcinoma antigen recognized by T-cells 3 OS=Mus musculus OX=10090 GN=Sart3 PE=1 SV=1	
BHMT1_MOUSE	Betaine--homocysteine S-methyltransferase 1 OS=Mus musculus OX=10090 GN=Bhmt PE=1 SV=1	
KPRB_MOUSE	Phosphoribosyl pyrophosphate synthase-associated protein 2 OS=Mus musculus OX=10090 GN=Prpsap2 PE=1 SV=1	
PPM1H_MOUSE	Protein phosphatase 1H OS=Mus musculus OX=10090 GN=Ppm1h PE=1 SV=1	
BAIP2_MOUSE	Brain-specific angiogenesis inhibitor 1-associated protein 2 OS=Mus musculus OX=10090 GN=Baiap2 PE=1 SV=2	
ACOT9_MOUSE	Acyl-coenzyme A thioesterase 9, mitochondrial OS=Mus musculus OX=10090 GN=Acot9 PE=1 SV=1	
RAB5A_MOUSE	Ras-related protein Rab-5A OS=Mus musculus OX=10090 GN=Rab5a PE=1 SV=1	
MEST_MOUSE	Mesoderm-specific transcript protein OS=Mus musculus OX=10090 GN=Mest PE=2 SV=1	
RCC1_MOUSE	Regulator of chromosome condensation OS=Mus musculus OX=10090 GN=Rcc1 PE=1 SV=1	
GOGA2_MOUSE	Golgin subfamily A member 2 OS=Mus musculus OX=10090 GN=Golga2 PE=1 SV=3	
LAMA5_MOUSE	Laminin subunit alpha-5 OS=Mus musculus OX=10090 GN=Lama5 PE=1 SV=4	
FABPH_MOUSE	Fatty acid-binding protein, heart OS=Mus musculus OX=10090 GN=Fabp3 PE=1 SV=5	
PO210_MOUSE	Nuclear pore membrane glycoprotein 210 OS=Mus musculus OX=10090 GN=Nup210 PE=1 SV=2	
CLPP_MOUSE	ATP-dependent Clp protease proteolytic subunit, mitochondrial OS=Mus musculus OX=10090 GN=Clpp PE=1 SV=1	
BROX_MOUSE	BRO1 domain-containing protein BROX OS=Mus musculus OX=10090 GN=Brox PE=1 SV=1	
CHM4B_MOUSE	Charged multivesicular body protein 4b OS=Mus musculus OX=10090 GN=Chmp4b PE=1 SV=2	
P4HA1_MOUSE	Prolyl 4-hydroxylase subunit alpha-1 OS=Mus musculus OX=10090 GN=P4ha1 PE=1 SV=2	
MT2_MOUSE	Metallothionein-2 OS=Mus musculus OX=10090 GN=Mt2 PE=1 SV=2	
IQGA1_MOUSE	Ras GTPase-activating-like protein IQGAP1 OS=Mus musculus OX=10090 GN=Iqgap1 PE=1 SV=2	
WDR48_MOUSE	WD repeat-containing protein 48 OS=Mus musculus OX=10090 GN=Wdr48 PE=1 SV=1	
Q27IK6-DECOY	Q27IK6	
MCM6_MOUSE	DNA replication licensing factor MCM6 OS=Mus musculus OX=10090 GN=Mcm6 PE=1 SV=1	
SIAS_MOUSE	Sialic acid synthase OS=Mus musculus OX=10090 GN=Nans PE=1 SV=1	
CTND1_MOUSE	Catenin delta-1 OS=Mus musculus OX=10090 GN=Ctnnd1 PE=1 SV=2	
NPS3B_MOUSE	Protein NipSnap homolog 3B OS=Mus musculus OX=10090 GN=Nipsnap3b PE=1 SV=1	
CSK_MOUSE	Tyrosine-protein kinase CSK OS=Mus musculus OX=10090 GN=Csk PE=1 SV=2	
VMA5A_MOUSE	von Willebrand factor A domain-containing protein 5A OS=Mus musculus OX=10090 GN=Vwa5a PE=1 SV=2	
ISOC1_MOUSE	Isochorismatase domain-containing protein 1 OS=Mus musculus OX=10090 GN=Isoc1 PE=1 SV=1	
C1QT4_MOUSE	Complement C1q tumor necrosis factor-related protein 4 OS=Mus musculus OX=10090 GN=C1qtnf4 PE=1 SV=1	
RBM12_MOUSE	RNA-binding protein 12 OS=Mus musculus OX=10090 GN=Rbm12 PE=1 SV=3	
CDV3_MOUSE	Protein CDV3 OS=Mus musculus OX=10090 GN=Cdv3 PE=1 SV=2	
MPPA_MOUSE	Mitochondrial-processing peptidase subunit alpha OS=Mus musculus OX=10090 GN=Pmpca PE=1 SV=1	
CTTB2_MOUSE	Cortactin-binding protein 2 OS=Mus musculus OX=10090 GN=Cttnbp2 PE=1 SV=2	
PGP_MOUSE	Glycerol-3-phosphate phosphatase OS=Mus musculus OX=10090 GN=Pgp PE=1 SV=1	
GNL1_MOUSE	Guanine nucleotide-binding protein-like 1 OS=Mus musculus OX=10090 GN=Gnl1 PE=1 SV=4	
ACAP2_MOUSE	Arf-GAP with coiled-coil, ANK repeat and PH domain-containing protein 2 OS=Mus musculus OX=10090 GN=Acap2 PE=1 SV=2	
Q80VH0-DECOY	Q80VH0	
FLOT2_MOUSE	Flotillin-2 OS=Mus musculus OX=10090 GN=Flot2 PE=1 SV=2	
PAK2_MOUSE	Serine/threonine-protein kinase PAK 2 OS=Mus musculus OX=10090 GN=Pak2 PE=1 SV=1	
RN213_MOUSE	E3 ubiquitin-protein ligase RNF213 OS=Mus musculus OX=10090 GN=Rnf213 PE=1 SV=2	
F210B_MOUSE	Protein FAM210B, mitochondrial OS=Mus musculus OX=10090 GN=Fam210b PE=1 SV=3	
COQ9_MOUSE	Ubiquinone biosynthesis protein COQ9, mitochondrial OS=Mus musculus OX=10090 GN=Coq9 PE=1 SV=1	
RANB3_MOUSE	Ran-binding protein 3 OS=Mus musculus OX=10090 GN=Ranbp3 PE=1 SV=2	
NDUAB_MOUSE	NADH dehydrogenase [ubiquinone] 1 alpha subcomplex subunit 11 OS=Mus musculus OX=10090 GN=Ndufa11 PE=1 SV=2	
LIN7C_MOUSE	Protein lin-7 homolog C OS=Mus musculus OX=10090 GN=Lin7c PE=1 SV=2	
ACO13_MOUSE	Acyl-coenzyme A thioesterase 13 OS=Mus musculus OX=10090 GN=Acot13 PE=1 SV=1	
HAP28_MOUSE	28 kDa heat- and acid-stable phosphoprotein OS=Mus musculus OX=10090 GN=Pdap1 PE=1 SV=1	
KV2A7_MOUSE	Ig kappa chain V-II region 26-10 OS=Mus musculus OX=10090 PE=1 SV=1	
IMA3_MOUSE	Importin subunit alpha-3 OS=Mus musculus OX=10090 GN=Kpna4 PE=1 SV=1	
ATP8_MOUSE	ATP synthase protein 8 OS=Mus musculus OX=10090 GN=Mtatp8 PE=1 SV=1	
SUMO2_MOUSE	Small ubiquitin-related modifier 2 OS=Mus musculus OX=10090 GN=Sumo2 PE=1 SV=1	
MOT1_MOUSE	Monocarboxylate transporter 1 OS=Mus musculus OX=10090 GN=Slc16a1 PE=1 SV=1	
NAMPT_MOUSE	Nicotinamide phosphoribosyltransferase OS=Mus musculus OX=10090 GN=Nampt PE=1 SV=1	
MXRA7_MOUSE	Matrix-remodeling-associated protein 7 OS=Mus musculus OX=10090 GN=Mxra7 PE=1 SV=2	
GMPPA_MOUSE	Mannose-1-phosphate guanyltransferase alpha OS=Mus musculus OX=10090 GN=Gmppa PE=1 SV=1	
PCDG4_MOUSE	Protocadherin gamma-A4 OS=Mus musculus OX=10090 GN=Pcdhga4 PE=1 SV=1	
OCAD1_MOUSE	OCIA domain-containing protein 1 OS=Mus musculus OX=10090 GN=Ociad1 PE=1 SV=1	
FMR1_MOUSE	Synaptic functional regulator FMR1 OS=Mus musculus OX=10090 GN=Fmr1 PE=1 SV=1	
NHRF1_MOUSE	Na(+)/H(+) exchange regulatory cofactor NHE-RF1 OS=Mus musculus OX=10090 GN=Slc9a3r1 PE=1 SV=3	
SRSF4_MOUSE	Serine/arginine-rich splicing factor 4 OS=Mus musculus OX=10090 GN=Srsf4 PE=2 SV=1	
HSBP1_MOUSE	Heat shock factor-binding protein 1 OS=Mus musculus OX=10090 GN=Hsbp1 PE=1 SV=1	
TPPC3_MOUSE	Trafficking protein particle complex subunit 3 OS=Mus musculus OX=10090 GN=Trappc3 PE=1 SV=1	
ICAM1_MOUSE	Intercellular adhesion molecule 1 OS=Mus musculus OX=10090 GN=Icam1 PE=1 SV=1	
NET1B_ARATH	Protein NETWORKED 1B OS=Arabidopsis thaliana OX=3702 GN=NET1B PE=2 SV=1	
CASC4_MOUSE	Protein CASC4 OS=Mus musculus OX=10090 GN=Casc4 PE=1 SV=1	
THUM1_MOUSE	THUMP domain-containing protein 1 OS=Mus musculus OX=10090 GN=Thumpd1 PE=1 SV=1	
GMPR1_MOUSE	GMP reductase 1 OS=Mus musculus OX=10090 GN=Gmpr PE=1 SV=1	
COX3_MOUSE	Cytochrome c oxidase subunit 3 OS=Mus musculus OX=10090 GN=mt-Co3 PE=1 SV=2	
NU1M_MOUSE	NADH-ubiquinone oxidoreductase chain 1 OS=Mus musculus OX=10090 GN=Mtnd1 PE=1 SV=3	
NUD10_MOUSE	Diphosphoinositol polyphosphate phosphohydrolase 3-alpha OS=Mus musculus OX=10090 GN=Nudt10 PE=1 SV=1	
RHEB_MOUSE	GTP-binding protein Rheb OS=Mus musculus OX=10090 GN=Rheb PE=1 SV=1	
MPU1_MOUSE	Mannose-P-dolichol utilization defect 1 protein OS=Mus musculus OX=10090 GN=Mpdu1 PE=1 SV=1	
NCKX4_MOUSE	Sodium/potassium/calcium exchanger 4 OS=Mus musculus OX=10090 GN=Slc24a4 PE=1 SV=2	
ENDD1_MOUSE	Endonuclease domain-containing 1 protein OS=Mus musculus OX=10090 GN=Endod1 PE=1 SV=2	
STRN3_MOUSE	Striatin-3 OS=Mus musculus OX=10090 GN=Strn3 PE=1 SV=1	
DHX30_MOUSE	ATP-dependent RNA helicase DHX30 OS=Mus musculus OX=10090 GN=Dhx30 PE=1 SV=1	
KISHA_MOUSE	Protein kish-A OS=Mus musculus OX=10090 GN=Tmem167a PE=1 SV=1	
PBX1_MOUSE	Pre-B-cell leukemia transcription factor 1 OS=Mus musculus OX=10090 GN=Pbx1 PE=1 SV=2	
HGS_MOUSE	Hepatocyte growth factor-regulated tyrosine kinase substrate OS=Mus musculus OX=10090 GN=Hgs PE=1 SV=2	
KV5A2_MOUSE	Ig kappa chain V-V region MOPC 21 OS=Mus musculus OX=10090 PE=1 SV=1	
CA2D3_MOUSE	Voltage-dependent calcium channel subunit alpha-2/delta-3 OS=Mus musculus OX=10090 GN=Cacna2d3 PE=1 SV=1	
Q8VHE6-DECOY	Q8VHE6	
CFAH_MOUSE	Complement factor H OS=Mus musculus OX=10090 GN=Cfh PE=1 SV=2	
APLP2_MOUSE	Amyloid-like protein 2 OS=Mus musculus OX=10090 GN=Aplp2 PE=1 SV=4	
MAT2B_MOUSE	Methionine adenosyltransferase 2 subunit beta OS=Mus musculus OX=10090 GN=Mat2b PE=1 SV=1	
CTBP2_MOUSE	C-terminal-binding protein 2 OS=Mus musculus OX=10090 GN=Ctbp2 PE=1 SV=2	
GBRA5_MOUSE	Gamma-aminobutyric acid receptor subunit alpha-5 OS=Mus musculus OX=10090 GN=Gabra5 PE=1 SV=1	
ICAM5_MOUSE	Intercellular adhesion molecule 5 OS=Mus musculus OX=10090 GN=Icam5 PE=1 SV=2	
NEGR1_MOUSE	Neuronal growth regulator 1 OS=Mus musculus OX=10090 GN=Negr1 PE=1 SV=1	
RT05_MOUSE	28S ribosomal protein S5, mitochondrial OS=Mus musculus OX=10090 GN=Mrps5 PE=1 SV=1	
DDX46_MOUSE	Probable ATP-dependent RNA helicase DDX46 OS=Mus musculus OX=10090 GN=Ddx46 PE=1 SV=2	
RGS7_MOUSE	Regulator of G-protein signaling 7 OS=Mus musculus OX=10090 GN=Rgs7 PE=1 SV=2	
IMA7_MOUSE	Importin subunit alpha-7 OS=Mus musculus OX=10090 GN=Kpna6 PE=1 SV=2	
FXR2_MOUSE	Fragile X mental retardation syndrome-related protein 2 OS=Mus musculus OX=10090 GN=Fxr2 PE=1 SV=1	
MTA2_MOUSE	Metastasis-associated protein MTA2 OS=Mus musculus OX=10090 GN=Mta2 PE=1 SV=1	
CPNE8_MOUSE	Copine-8 OS=Mus musculus OX=10090 GN=Cpne8 PE=2 SV=3	
CRNL1_MOUSE	Crooked neck-like protein 1 OS=Mus musculus OX=10090 GN=Crnkl1 PE=1 SV=1	
EFGM_MOUSE	Elongation factor G, mitochondrial OS=Mus musculus OX=10090 GN=Gfm1 PE=1 SV=1	
LYAG_MOUSE	Lysosomal alpha-glucosidase OS=Mus musculus OX=10090 GN=Gaa PE=1 SV=2	
SAP_MOUSE	Prosaposin OS=Mus musculus OX=10090 GN=Psap PE=1 SV=2	
STK39_MOUSE	STE20/SPS1-related proline-alanine-rich protein kinase OS=Mus musculus OX=10090 GN=Stk39 PE=1 SV=1	
ARMC6_MOUSE	Armadillo repeat-containing protein 6 OS=Mus musculus OX=10090 GN=Armc6 PE=1 SV=1	
NCOA5_MOUSE	Nuclear receptor coactivator 5 OS=Mus musculus OX=10090 GN=Ncoa5 PE=1 SV=1	
ECM29_MOUSE	Proteasome adapter and scaffold protein ECM29 OS=Mus musculus OX=10090 GN=Ecpas PE=1 SV=3	
MCTS1_MOUSE	Malignant T-cell-amplified sequence 1 OS=Mus musculus OX=10090 GN=Mcts1 PE=1 SV=1	
ITA6_MOUSE	Integrin alpha-6 OS=Mus musculus OX=10090 GN=Itga6 PE=1 SV=3	
SEPT8_MOUSE	Septin-8 OS=Mus musculus OX=10090 GN=Septin8 PE=1 SV=4	
HIP1R_MOUSE	Huntingtin-interacting protein 1-related protein OS=Mus musculus OX=10090 GN=Hip1r PE=1 SV=2	
PUM2_MOUSE	Pumilio homolog 2 OS=Mus musculus OX=10090 GN=Pum2 PE=1 SV=2	
CE170_MOUSE	Centrosomal protein of 170 kDa OS=Mus musculus OX=10090 GN=Cep170 PE=1 SV=2	
AT2B4_MOUSE	Plasma membrane calcium-transporting ATPase 4 OS=Mus musculus OX=10090 GN=Atp2b4 PE=1 SV=1	
BSN_MOUSE	Protein bassoon OS=Mus musculus OX=10090 GN=Bsn PE=1 SV=4	
SYTC2_MOUSE	Threonine--tRNA ligase 2, cytoplasmic OS=Mus musculus OX=10090 GN=Tarsl2 PE=1 SV=1	
CUL4A_MOUSE	Cullin-4A OS=Mus musculus OX=10090 GN=Cul4a PE=1 SV=1	
UBP47_MOUSE	Ubiquitin carboxyl-terminal hydrolase 47 OS=Mus musculus OX=10090 GN=Usp47 PE=1 SV=2	
EHD3_MOUSE	EH domain-containing protein 3 OS=Mus musculus OX=10090 GN=Ehd3 PE=1 SV=2	
DCAF7_MOUSE	DDB1- and CUL4-associated factor 7 OS=Mus musculus OX=10090 GN=Dcaf7 PE=1 SV=1	
S27A4_MOUSE	Long-chain fatty acid transport protein 4 OS=Mus musculus OX=10090 GN=Slc27a4 PE=1 SV=1	
CLIP1_MOUSE	CAP-Gly domain-containing linker protein 1 OS=Mus musculus OX=10090 GN=Clip1 PE=1 SV=1	
RAB23_MOUSE	Ras-related protein Rab-23 OS=Mus musculus OX=10090 GN=Rab23 PE=1 SV=2	
MRCKB_MOUSE	Serine/threonine-protein kinase MRCK beta OS=Mus musculus OX=10090 GN=Cdc42bpb PE=1 SV=2	
SH3G1_MOUSE	Endophilin-A2 OS=Mus musculus OX=10090 GN=Sh3gl1 PE=1 SV=1	
NF1_MOUSE	Neurofibromin OS=Mus musculus OX=10090 GN=Nf1 PE=1 SV=1	
PGAM5_MOUSE	Serine/threonine-protein phosphatase PGAM5, mitochondrial OS=Mus musculus OX=10090 GN=Pgam5 PE=1 SV=1	
RUXF_MOUSE	Small nuclear ribonucleoprotein F OS=Mus musculus OX=10090 GN=Snrpf PE=1 SV=1	
PHF24_MOUSE	PHD finger protein 24 OS=Mus musculus OX=10090 GN=Phf24 PE=1 SV=2	
SRR_MOUSE	Serine racemase OS=Mus musculus OX=10090 GN=Srr PE=1 SV=1	
NDUS4_MOUSE	NADH dehydrogenase [ubiquinone] iron-sulfur protein 4, mitochondrial OS=Mus musculus OX=10090 GN=Ndufs4 PE=1 SV=3	
TMX1_MOUSE	Thioredoxin-related transmembrane protein 1 OS=Mus musculus OX=10090 GN=Tmx1 PE=1 SV=1	
GAS7_MOUSE	Growth arrest-specific protein 7 OS=Mus musculus OX=10090 GN=Gas7 PE=1 SV=1	
KV5A7_MOUSE	Ig kappa chain V-V region MOPC 41 OS=Mus musculus OX=10090 GN=Gm5571 PE=1 SV=1	
ECI1_MOUSE	Enoyl-CoA delta isomerase 1, mitochondrial OS=Mus musculus OX=10090 GN=Eci1 PE=1 SV=2	
NUDC2_MOUSE	NudC domain-containing protein 2 OS=Mus musculus OX=10090 GN=Nudcd2 PE=1 SV=1	
SPRE_MOUSE	Sepiapterin reductase OS=Mus musculus OX=10090 GN=Spr PE=1 SV=1	
AL1A7_MOUSE	Aldehyde dehydrogenase, cytosolic 1 OS=Mus musculus OX=10090 GN=Aldh1a7 PE=1 SV=1	
PLPR2_MOUSE	Phospholipid phosphatase-related protein type 2 OS=Mus musculus OX=10090 GN=Plppr2 PE=1 SV=1	
S39A6_MOUSE	Zinc transporter ZIP6 OS=Mus musculus OX=10090 GN=Slc39a6 PE=1 SV=1	
KV3AM_MOUSE	Ig kappa chain V-III region PC 2154 OS=Mus musculus OX=10090 PE=1 SV=1	
PC4L1_MOUSE	Purkinje cell protein 4-like protein 1 OS=Mus musculus OX=10090 GN=Pcp4l1 PE=1 SV=1	
GBG3_MOUSE	Guanine nucleotide-binding protein G(I)/G(S)/G(O) subunit gamma-3 OS=Mus musculus OX=10090 GN=Gng3 PE=1 SV=1	
TBA3_ARATH	Tubulin alpha-3 chain OS=Arabidopsis thaliana OX=3702 GN=TUBA3 PE=1 SV=2	
CON__P12763	CON__P12763	
RUXE_MOUSE	Small nuclear ribonucleoprotein E OS=Mus musculus OX=10090 GN=Snrpe PE=1 SV=1	
O82190-DECOY	O82190	
PFD2_MOUSE	Prefoldin subunit 2 OS=Mus musculus OX=10090 GN=Pfdn2 PE=1 SV=2	
DHB8_MOUSE	Estradiol 17-beta-dehydrogenase 8 OS=Mus musculus OX=10090 GN=Hsd17b8 PE=1 SV=2	
GHC2_MOUSE	Mitochondrial glutamate carrier 2 OS=Mus musculus OX=10090 GN=Slc25a18 PE=1 SV=4	
ABRAL_MOUSE	Costars family protein ABRACL OS=Mus musculus OX=10090 GN=Abracl PE=1 SV=1	
PK1IP_MOUSE	p21-activated protein kinase-interacting protein 1 OS=Mus musculus OX=10090 GN=Pak1ip1 PE=1 SV=2	
RT07_MOUSE	28S ribosomal protein S7, mitochondrial OS=Mus musculus OX=10090 GN=Mrps7 PE=1 SV=1	
NSMA2_MOUSE	Sphingomyelin phosphodiesterase 3 OS=Mus musculus OX=10090 GN=Smpd3 PE=1 SV=1	
HBA_MOUSE	Hemoglobin subunit alpha OS=Mus musculus OX=10090 GN=Hba PE=1 SV=2	
CTNA1_MOUSE	Catenin alpha-1 OS=Mus musculus OX=10090 GN=Ctnna1 PE=1 SV=1	
CCG8_MOUSE	Voltage-dependent calcium channel gamma-8 subunit OS=Mus musculus OX=10090 GN=Cacng8 PE=1 SV=1	
LANC1_MOUSE	Glutathione S-transferase LANCL1 OS=Mus musculus OX=10090 GN=Lancl1 PE=1 SV=1	
FADS2_MOUSE	Acyl-CoA 6-desaturase OS=Mus musculus OX=10090 GN=Fads2 PE=1 SV=1	
PCP4_HUMAN	Calmodulin regulator protein PCP4 OS=Homo sapiens OX=9606 GN=PCP4 PE=1 SV=3	
GSK3A_MOUSE	Glycogen synthase kinase-3 alpha OS=Mus musculus OX=10090 GN=Gsk3a PE=1 SV=2	
TNPO1_MOUSE	Transportin-1 OS=Mus musculus OX=10090 GN=Tnpo1 PE=1 SV=2	
PDLI1_MOUSE	PDZ and LIM domain protein 1 OS=Mus musculus OX=10090 GN=Pdlim1 PE=1 SV=4	
PR38B_MOUSE	Pre-mRNA-splicing factor 38B OS=Mus musculus OX=10090 GN=Prpf38b PE=1 SV=1	
SRP14_MOUSE	Signal recognition particle 14 kDa protein OS=Mus musculus OX=10090 GN=Srp14 PE=1 SV=1	
ACSL3_MOUSE	Long-chain-fatty-acid--CoA ligase 3 OS=Mus musculus OX=10090 GN=Acsl3 PE=1 SV=2	
KV6A6_MOUSE	Ig kappa chain V-VI region NQ2-17.4.1 OS=Mus musculus OX=10090 PE=2 SV=1	
PITM1_MOUSE	Membrane-associated phosphatidylinositol transfer protein 1 OS=Mus musculus OX=10090 GN=Pitpnm1 PE=1 SV=1	
HDGR2_MOUSE	Hepatoma-derived growth factor-related protein 2 OS=Mus musculus OX=10090 GN=Hdgfl2 PE=1 SV=1	
AP4A_MOUSE	Bis(5'-nucleosyl)-tetraphosphatase [asymmetrical] OS=Mus musculus OX=10090 GN=Nudt2 PE=1 SV=3	
LOXL2_HUMAN	Lysyl oxidase homolog 2 OS=Homo sapiens OX=9606 GN=LOXL2 PE=1 SV=1	
VPS18_MOUSE	Vacuolar protein sorting-associated protein 18 homolog OS=Mus musculus OX=10090 GN=Vps18 PE=1 SV=2	
TFAM_MOUSE	Transcription factor A, mitochondrial OS=Mus musculus OX=10090 GN=Tfam PE=1 SV=2	
S10A6_MOUSE	Protein S100-A6 OS=Mus musculus OX=10090 GN=S100a6 PE=1 SV=3	
PICAL_MOUSE	Phosphatidylinositol-binding clathrin assembly protein OS=Mus musculus OX=10090 GN=Picalm PE=1 SV=1	
SF3B4_MOUSE	Splicing factor 3B subunit 4 OS=Mus musculus OX=10090 GN=Sf3b4 PE=1 SV=1	
BAX_MOUSE	Apoptosis regulator BAX OS=Mus musculus OX=10090 GN=Bax PE=1 SV=1	
INP4A_MOUSE	Inositol polyphosphate-4-phosphatase type I A OS=Mus musculus OX=10090 GN=Inpp4a PE=1 SV=1	
CS1A_MOUSE	Complement C1s-A subcomponent OS=Mus musculus OX=10090 GN=C1sa PE=2 SV=2	
ISLR2_MOUSE	Immunoglobulin superfamily containing leucine-rich repeat protein 2 OS=Mus musculus OX=10090 GN=Islr2 PE=1 SV=1	
GPC2_MOUSE	Glypican-2 OS=Mus musculus OX=10090 GN=Gpc2 PE=2 SV=1	
HECAM_MOUSE	Hepatocyte cell adhesion molecule OS=Mus musculus OX=10090 GN=Hepacam PE=1 SV=2	
DNAK_MYCTU	Chaperone protein DnaK OS=Mycobacterium tuberculosis (strain ATCC 25618 / H37Rv) OX=83332 GN=dnaK PE=1 SV=1	
TTC3_MOUSE	E3 ubiquitin-protein ligase TTC3 OS=Mus musculus OX=10090 GN=Ttc3 PE=1 SV=2	
AGO2_MOUSE	Protein argonaute-2 OS=Mus musculus OX=10090 GN=Ago2 PE=1 SV=3	
KIFA3_MOUSE	Kinesin-associated protein 3 OS=Mus musculus OX=10090 GN=Kifap3 PE=1 SV=1	
CPLX1_MOUSE	Complexin-1 OS=Mus musculus OX=10090 GN=Cplx1 PE=1 SV=1	
GIT1_MOUSE	ARF GTPase-activating protein GIT1 OS=Mus musculus OX=10090 GN=Git1 PE=1 SV=1	
WDR6_MOUSE	WD repeat-containing protein 6 OS=Mus musculus OX=10090 GN=Wdr6 PE=1 SV=1	
UBFD1_MOUSE	Ubiquitin domain-containing protein UBFD1 OS=Mus musculus OX=10090 GN=Ubfd1 PE=1 SV=2	
IGSF8_MOUSE	Immunoglobulin superfamily member 8 OS=Mus musculus OX=10090 GN=Igsf8 PE=1 SV=2	
SMAP1_MOUSE	Stromal membrane-associated protein 1 OS=Mus musculus OX=10090 GN=Smap1 PE=1 SV=1	
PININ_MOUSE	Pinin OS=Mus musculus OX=10090 GN=Pnn PE=1 SV=4	
VTA1_MOUSE	Vacuolar protein sorting-associated protein VTA1 homolog OS=Mus musculus OX=10090 GN=Vta1 PE=1 SV=1	
MEP50_MOUSE	Methylosome protein 50 OS=Mus musculus OX=10090 GN=Wdr77 PE=1 SV=1	
LRRK1_MOUSE	Leucine-rich repeat serine/threonine-protein kinase 1 OS=Mus musculus OX=10090 GN=Lrrk1 PE=1 SV=1	
AL1A1_MOUSE	Retinal dehydrogenase 1 OS=Mus musculus OX=10090 GN=Aldh1a1 PE=1 SV=5	
ILK_MOUSE	Integrin-linked protein kinase OS=Mus musculus OX=10090 GN=Ilk PE=1 SV=2	
DLG2_MOUSE	Disks large homolog 2 OS=Mus musculus OX=10090 GN=Dlg2 PE=1 SV=2	
CYTB_MOUSE	Cystatin-B OS=Mus musculus OX=10090 GN=Cstb PE=1 SV=1	
BRCA2_MOUSE	Breast cancer type 2 susceptibility protein homolog OS=Mus musculus OX=10090 GN=Brca2 PE=1 SV=2	
IPYR2_MOUSE	Inorganic pyrophosphatase 2, mitochondrial OS=Mus musculus OX=10090 GN=Ppa2 PE=1 SV=1	
CPNE3_MOUSE	Copine-3 OS=Mus musculus OX=10090 GN=Cpne3 PE=1 SV=2	
HMGN2_MOUSE	Non-histone chromosomal protein HMG-17 OS=Mus musculus OX=10090 GN=Hmgn2 PE=1 SV=2	
TBAL3_MOUSE	Tubulin alpha chain-like 3 OS=Mus musculus OX=10090 GN=Tubal3 PE=2 SV=2	
S23IP_MOUSE	SEC23-interacting protein OS=Mus musculus OX=10090 GN=Sec23ip PE=1 SV=2	
KV2A6_MOUSE	Ig kappa chain V-II region 7S34.1 OS=Mus musculus OX=10090 PE=1 SV=1	
NDUB3_MOUSE	NADH dehydrogenase [ubiquinone] 1 beta subcomplex subunit 3 OS=Mus musculus OX=10090 GN=Ndufb3 PE=1 SV=1	
TCAL3_MOUSE	Transcription elongation factor A protein-like 3 OS=Mus musculus OX=10090 GN=Tceal3 PE=1 SV=2	
RAA1E_ARATH	Ras-related protein RABA1e OS=Arabidopsis thaliana OX=3702 GN=RABA1E PE=2 SV=1	
TIM14_MOUSE	Mitochondrial import inner membrane translocase subunit TIM14 OS=Mus musculus OX=10090 GN=Dnajc19 PE=1 SV=3	
VKORL_MOUSE	Vitamin K epoxide reductase complex subunit 1-like protein 1 OS=Mus musculus OX=10090 GN=Vkorc1l1 PE=1 SV=1	
CSN7A_MOUSE	COP9 signalosome complex subunit 7a OS=Mus musculus OX=10090 GN=Cops7a PE=1 SV=2	
VATB1_ARATH	V-type proton ATPase subunit B1 OS=Arabidopsis thaliana OX=3702 GN=VHA-B1 PE=2 SV=2	
B2L13_MOUSE	Bcl-2-like protein 13 OS=Mus musculus OX=10090 GN=Bcl2l13 PE=1 SV=2	
NCS1_MOUSE	Neuronal calcium sensor 1 OS=Mus musculus OX=10090 GN=Ncs1 PE=1 SV=3	
ICLN_MOUSE	Methylosome subunit pICln OS=Mus musculus OX=10090 GN=Clns1a PE=1 SV=1	
MK09_MOUSE	Mitogen-activated protein kinase 9 OS=Mus musculus OX=10090 GN=Mapk9 PE=1 SV=2	
CHMP5_MOUSE	Charged multivesicular body protein 5 OS=Mus musculus OX=10090 GN=Chmp5 PE=1 SV=1	
CON__Q3SX28	CON__Q3SX28	
MT1_MOUSE	Metallothionein-1 OS=Mus musculus OX=10090 GN=Mt1 PE=1 SV=1	
TC132_ARATH	Translocase of chloroplast 132, chloroplastic OS=Arabidopsis thaliana OX=3702 GN=TOC132 PE=1 SV=1	
CACP_MOUSE	Carnitine O-acetyltransferase OS=Mus musculus OX=10090 GN=Crat PE=1 SV=3	
2ABD_MOUSE	Serine/threonine-protein phosphatase 2A 55 kDa regulatory subunit B delta isoform OS=Mus musculus OX=10090 GN=Ppp2r2d PE=1 SV=1	
1433S_MOUSE	14-3-3 protein sigma OS=Mus musculus OX=10090 GN=Sfn PE=1 SV=2	
H31_HUMAN	Histone H3.1 OS=Homo sapiens OX=9606 GN=H3C1 PE=1 SV=2	
HPCL1_MOUSE	Hippocalcin-like protein 1 OS=Mus musculus OX=10090 GN=Hpcal1 PE=1 SV=2	
CPSF7_MOUSE	Cleavage and polyadenylation specificity factor subunit 7 OS=Mus musculus OX=10090 GN=Cpsf7 PE=1 SV=2	
GAK_MOUSE	Cyclin-G-associated kinase OS=Mus musculus OX=10090 GN=Gak PE=1 SV=2	
RBMX_MOUSE	RNA-binding motif protein, X chromosome OS=Mus musculus OX=10090 GN=Rbmx PE=1 SV=1	
GCDH_MOUSE	Glutaryl-CoA dehydrogenase, mitochondrial OS=Mus musculus OX=10090 GN=Gcdh PE=1 SV=2	
TCAF1_MOUSE	TRPM8 channel-associated factor 1 OS=Mus musculus OX=10090 GN=Tcaf1 PE=2 SV=1	
LEA29_ARATH	Late embryogenesis abundant protein 29 OS=Arabidopsis thaliana OX=3702 GN=LEA29 PE=2 SV=1	
MCA3_MOUSE	Eukaryotic translation elongation factor 1 epsilon-1 OS=Mus musculus OX=10090 GN=Eef1e1 PE=1 SV=1	
CROCC_MOUSE	Rootletin OS=Mus musculus OX=10090 GN=Crocc PE=1 SV=2	
PNPH_MOUSE	Purine nucleoside phosphorylase OS=Mus musculus OX=10090 GN=Pnp PE=1 SV=2	
RM04_MOUSE	39S ribosomal protein L4, mitochondrial OS=Mus musculus OX=10090 GN=Mrpl4 PE=1 SV=1	
PK3CD_MOUSE	Phosphatidylinositol 4,5-bisphosphate 3-kinase catalytic subunit delta isoform OS=Mus musculus OX=10090 GN=Pik3cd PE=1 SV=2	
FKBP5_MOUSE	Peptidyl-prolyl cis-trans isomerase FKBP5 OS=Mus musculus OX=10090 GN=Fkbp5 PE=1 SV=1	
RAB8B_MOUSE	Ras-related protein Rab-8B OS=Mus musculus OX=10090 GN=Rab8b PE=1 SV=1	
GDE1_MOUSE	Glycerophosphodiester phosphodiesterase 1 OS=Mus musculus OX=10090 GN=Gde1 PE=1 SV=1	
ECSIT_MOUSE	Evolutionarily conserved signaling intermediate in Toll pathway, mitochondrial OS=Mus musculus OX=10090 GN=Ecsit PE=1 SV=2	
LBR_MOUSE	Delta(14)-sterol reductase LBR OS=Mus musculus OX=10090 GN=Lbr PE=1 SV=2	
CUL4B_MOUSE	Cullin-4B OS=Mus musculus OX=10090 GN=Cul4b PE=1 SV=1	
NIT2_MOUSE	Omega-amidase NIT2 OS=Mus musculus OX=10090 GN=Nit2 PE=1 SV=1	
TEFF2_MOUSE	Tomoregulin-2 OS=Mus musculus OX=10090 GN=Tmeff2 PE=2 SV=1	
GPI8_MOUSE	GPI-anchor transamidase OS=Mus musculus OX=10090 GN=Pigk PE=1 SV=2	
AFAD_MOUSE	Afadin OS=Mus musculus OX=10090 GN=Afdn PE=1 SV=3	
GPC1_MOUSE	Glypican-1 OS=Mus musculus OX=10090 GN=Gpc1 PE=1 SV=1	
NUDC3_MOUSE	NudC domain-containing protein 3 OS=Mus musculus OX=10090 GN=Nudcd3 PE=1 SV=3	
PCNA_MOUSE	Proliferating cell nuclear antigen OS=Mus musculus OX=10090 GN=Pcna PE=1 SV=2	
PFD6_MOUSE	Prefoldin subunit 6 OS=Mus musculus OX=10090 GN=Pfdn6 PE=1 SV=1	
MCM7_MOUSE	DNA replication licensing factor MCM7 OS=Mus musculus OX=10090 GN=Mcm7 PE=1 SV=1	
PFD5_MOUSE	Prefoldin subunit 5 OS=Mus musculus OX=10090 GN=Pfdn5 PE=1 SV=1	
RNZ2_MOUSE	Zinc phosphodiesterase ELAC protein 2 OS=Mus musculus OX=10090 GN=Elac2 PE=1 SV=1	
NAGK_MOUSE	N-acetyl-D-glucosamine kinase OS=Mus musculus OX=10090 GN=Nagk PE=1 SV=3	
CBARP_MOUSE	Voltage-dependent calcium channel beta subunit-associated regulatory protein OS=Mus musculus OX=10090 GN=Cbarp PE=1 SV=4	
TBCA_MOUSE	Tubulin-specific chaperone A OS=Mus musculus OX=10090 GN=Tbca PE=1 SV=3	
PRUN1_MOUSE	Exopolyphosphatase PRUNE1 OS=Mus musculus OX=10090 GN=Prune1 PE=1 SV=1	
BTF3_MOUSE	Transcription factor BTF3 OS=Mus musculus OX=10090 GN=Btf3 PE=1 SV=3	
KPCE_MOUSE	Protein kinase C epsilon type OS=Mus musculus OX=10090 GN=Prkce PE=1 SV=1	
IPO11_MOUSE	Importin-11 OS=Mus musculus OX=10090 GN=Ipo11 PE=1 SV=1	
PAPS1_MOUSE	Bifunctional 3'-phosphoadenosine 5'-phosphosulfate synthase 1 OS=Mus musculus OX=10090 GN=Papss1 PE=1 SV=1	
FAF2_MOUSE	FAS-associated factor 2 OS=Mus musculus OX=10090 GN=Faf2 PE=1 SV=2	
EMC8_MOUSE	ER membrane protein complex subunit 8 OS=Mus musculus OX=10090 GN=Emc8 PE=1 SV=1	
NT5C_MOUSE	5'(3')-deoxyribonucleotidase, cytosolic type OS=Mus musculus OX=10090 GN=Nt5c PE=1 SV=1	
CTBL1_MOUSE	Beta-catenin-like protein 1 OS=Mus musculus OX=10090 GN=Ctnnbl1 PE=1 SV=1	
Q94FN2-DECOY	Q94FN2	
FLII_MOUSE	Protein flightless-1 homolog OS=Mus musculus OX=10090 GN=Flii PE=1 SV=1	
CON__Q32PI4	CON__Q32PI4	
PSMG2_MOUSE	Proteasome assembly chaperone 2 OS=Mus musculus OX=10090 GN=Psmg2 PE=1 SV=1	
ABI2_MOUSE	Abl interactor 2 OS=Mus musculus OX=10090 GN=Abi2 PE=1 SV=1	
AGRL3_MOUSE	Adhesion G protein-coupled receptor L3 OS=Mus musculus OX=10090 GN=Adgrl3 PE=1 SV=3	
MAP1S_MOUSE	Microtubule-associated protein 1S OS=Mus musculus OX=10090 GN=Map1s PE=1 SV=2	
NMRL1_MOUSE	NmrA-like family domain-containing protein 1 OS=Mus musculus OX=10090 GN=Nmral1 PE=1 SV=1	
HM13_MOUSE	Minor histocompatibility antigen H13 OS=Mus musculus OX=10090 GN=Hm13 PE=1 SV=1	
DYH5_HUMAN	Dynein heavy chain 5, axonemal OS=Homo sapiens OX=9606 GN=DNAH5 PE=1 SV=3	
MICU1_MOUSE	Calcium uptake protein 1, mitochondrial OS=Mus musculus OX=10090 GN=Micu1 PE=1 SV=1	
P5CR3_MOUSE	Pyrroline-5-carboxylate reductase 3 OS=Mus musculus OX=10090 GN=Pycr3 PE=1 SV=2	
TDRKH_MOUSE	Tudor and KH domain-containing protein OS=Mus musculus OX=10090 GN=Tdrkh PE=1 SV=1	
RBGPR_MOUSE	Rab3 GTPase-activating protein non-catalytic subunit OS=Mus musculus OX=10090 GN=Rab3gap2 PE=1 SV=2	
DJC11_MOUSE	DnaJ homolog subfamily C member 11 OS=Mus musculus OX=10090 GN=Dnajc11 PE=1 SV=2	
OXSR1_MOUSE	Serine/threonine-protein kinase OSR1 OS=Mus musculus OX=10090 GN=Oxsr1 PE=1 SV=1	
S4A7_MOUSE	Sodium bicarbonate cotransporter 3 OS=Mus musculus OX=10090 GN=Slc4a7 PE=1 SV=2	
PP4P2_MOUSE	Type 2 phosphatidylinositol 4,5-bisphosphate 4-phosphatase OS=Mus musculus OX=10090 GN=Pip4p2 PE=1 SV=1	
MCM3_MOUSE	DNA replication licensing factor MCM3 OS=Mus musculus OX=10090 GN=Mcm3 PE=1 SV=2	
P85A_MOUSE	Phosphatidylinositol 3-kinase regulatory subunit alpha OS=Mus musculus OX=10090 GN=Pik3r1 PE=1 SV=2	
KIF15_MOUSE	Kinesin-like protein KIF15 OS=Mus musculus OX=10090 GN=Kif15 PE=1 SV=1	
BUD31_MOUSE	Protein BUD31 homolog OS=Mus musculus OX=10090 GN=Bud31 PE=1 SV=2	
C1QBP_MOUSE	Complement component 1 Q subcomponent-binding protein, mitochondrial OS=Mus musculus OX=10090 GN=C1qbp PE=1 SV=1	
HVM17_MOUSE	Ig heavy chain V region MOPC 47A OS=Mus musculus OX=10090 PE=1 SV=1	
HVM35_MOUSE	Ig heavy chain V-III region HPC76 (Fragment) OS=Mus musculus OX=10090 PE=4 SV=1	
HVM54_MOUSE	Ig heavy chain V region 5-84 OS=Mus musculus OX=10090 PE=1 SV=1	
HVM55_MOUSE	Ig heavy chain V region 345 OS=Mus musculus OX=10090 PE=1 SV=1	
KV4A1_MOUSE	Ig kappa chain V-IV region S107B OS=Mus musculus OX=10090 PE=4 SV=1	
KV5A4_MOUSE	Ig kappa chain V-V region MOPC 149 OS=Mus musculus OX=10090 PE=1 SV=1	
KV5A9_MOUSE	Ig kappa chain V-V region L7 (Fragment) OS=Mus musculus OX=10090 GN=Gm10881 PE=1 SV=1	
KV6AB_MOUSE	Ig kappa chain V-VI region NQ2-6.1 OS=Mus musculus OX=10090 PE=2 SV=1	
MGST1_MOUSE	Microsomal glutathione S-transferase 1 OS=Mus musculus OX=10090 GN=Mgst1 PE=1 SV=3	
NXF1_MOUSE	Nuclear RNA export factor 1 OS=Mus musculus OX=10090 GN=Nxf1 PE=1 SV=3	
RL36_MOUSE	60S ribosomal protein L36 OS=Mus musculus OX=10090 GN=Rpl36 PE=1 SV=2	
SUMO1_MOUSE	Small ubiquitin-related modifier 1 OS=Mus musculus OX=10090 GN=Sumo1 PE=1 SV=1	
RS30_MOUSE	40S ribosomal protein S30 OS=Mus musculus OX=10090 GN=Fau PE=1 SV=1	
TOM7_MOUSE	Mitochondrial import receptor subunit TOM7 homolog OS=Mus musculus OX=10090 GN=Tomm7 PE=3 SV=1	
KV2A5_MOUSE	Ig kappa chain V-II region 17S29.1 OS=Mus musculus OX=10090 PE=1 SV=1	
MYB73_ARATH	Transcription factor MYB73 OS=Arabidopsis thaliana OX=3702 GN=MYB73 PE=1 SV=1	
INV3_ARATH	Beta-fructofuranosidase, insoluble isoenzyme CWINV3 OS=Arabidopsis thaliana OX=3702 GN=CWINV3 PE=1 SV=2	
ARI1_HUMAN	E3 ubiquitin-protein ligase ARIH1 OS=Homo sapiens OX=9606 GN=ARIH1 PE=1 SV=2	
CON__Q7Z794	CON__Q7Z794	
SP8_MOUSE	Transcription factor Sp8 OS=Mus musculus OX=10090 GN=Sp8 PE=2 SV=1	
INPP_MOUSE	Inositol polyphosphate 1-phosphatase OS=Mus musculus OX=10090 GN=Inpp1 PE=1 SV=2	
GALD1_MOUSE	Glutamine amidotransferase-like class 1 domain-containing protein 1 OS=Mus musculus OX=10090 GN=Gatd1 PE=1 SV=1	
ATE1_MOUSE	Arginyl-tRNA--protein transferase 1 OS=Mus musculus OX=10090 GN=Ate1 PE=1 SV=2	
SENP8_MOUSE	Sentrin-specific protease 8 OS=Mus musculus OX=10090 GN=Senp8 PE=1 SV=2	
ADDG_MOUSE	Gamma-adducin OS=Mus musculus OX=10090 GN=Add3 PE=1 SV=2	
Q9JMH9-DECOY	Q9JMH9	
SUN2_MOUSE	SUN domain-containing protein 2 OS=Mus musculus OX=10090 GN=Sun2 PE=1 SV=3	
ENAH_MOUSE	Protein enabled homolog OS=Mus musculus OX=10090 GN=Enah PE=1 SV=2	
NR4A2_MOUSE	Nuclear receptor subfamily 4 group A member 2 OS=Mus musculus OX=10090 GN=Nr4a2 PE=1 SV=1	
GNB5_MOUSE	Guanine nucleotide-binding protein subunit beta-5 OS=Mus musculus OX=10090 GN=Gnb5 PE=1 SV=1	
MK08_MOUSE	Mitogen-activated protein kinase 8 OS=Mus musculus OX=10090 GN=Mapk8 PE=1 SV=1	
CERS6_MOUSE	Ceramide synthase 6 OS=Mus musculus OX=10090 GN=Cers6 PE=1 SV=1	
IF6_MOUSE	Eukaryotic translation initiation factor 6 OS=Mus musculus OX=10090 GN=Eif6 PE=1 SV=2	
ARP5L_MOUSE	Actin-related protein 2/3 complex subunit 5-like protein OS=Mus musculus OX=10090 GN=Arpc5l PE=1 SV=1	
CBX1_MOUSE	Chromobox protein homolog 1 OS=Mus musculus OX=10090 GN=Cbx1 PE=1 SV=1	
WDR13_MOUSE	WD repeat-containing protein 13 OS=Mus musculus OX=10090 GN=Wdr13 PE=1 SV=1	
RUXG_MOUSE	Small nuclear ribonucleoprotein G OS=Mus musculus OX=10090 GN=Snrpg PE=1 SV=1	
TSYL4_MOUSE	Testis-specific Y-encoded-like protein 4 OS=Mus musculus OX=10090 GN=Tspyl4 PE=1 SV=1	
SNR40_MOUSE	U5 small nuclear ribonucleoprotein 40 kDa protein OS=Mus musculus OX=10090 GN=Snrnp40 PE=1 SV=1	
SMRD3_MOUSE	SWI/SNF-related matrix-associated actin-dependent regulator of chromatin subfamily D member 3 OS=Mus musculus OX=10090 GN=Smarcd3 PE=1 SV=2	
STAT3_MOUSE	Signal transducer and activator of transcription 3 OS=Mus musculus OX=10090 GN=Stat3 PE=1 SV=2	
ADAS_MOUSE	Alkyldihydroxyacetonephosphate synthase, peroxisomal OS=Mus musculus OX=10090 GN=Agps PE=1 SV=1	
P4HA3_MOUSE	Prolyl 4-hydroxylase subunit alpha-3 OS=Mus musculus OX=10090 GN=P4ha3 PE=2 SV=1	
X3CL1_MOUSE	Fractalkine OS=Mus musculus OX=10090 GN=Cx3cl1 PE=1 SV=3	
ATX2L_MOUSE	Ataxin-2-like protein OS=Mus musculus OX=10090 GN=Atxn2l PE=1 SV=1	
SHOT1_MOUSE	Shootin-1 OS=Mus musculus OX=10090 GN=Shtn1 PE=1 SV=1	
RM22_MOUSE	39S ribosomal protein L22, mitochondrial OS=Mus musculus OX=10090 GN=Mrpl22 PE=1 SV=1	
CHERP_MOUSE	Calcium homeostasis endoplasmic reticulum protein OS=Mus musculus OX=10090 GN=Cherp PE=1 SV=1	
MEMO1_MOUSE	Protein MEMO1 OS=Mus musculus OX=10090 GN=Memo1 PE=1 SV=1	
MLF2_MOUSE	Myeloid leukemia factor 2 OS=Mus musculus OX=10090 GN=Mlf2 PE=1 SV=1	
I2BPL_MOUSE	Probable E3 ubiquitin-protein ligase IRF2BPL OS=Mus musculus OX=10090 GN=Irf2bpl PE=1 SV=1	
DKC1_MOUSE	H/ACA ribonucleoprotein complex subunit DKC1 OS=Mus musculus OX=10090 GN=Dkc1 PE=1 SV=4	
DNJC7_MOUSE	DnaJ homolog subfamily C member 7 OS=Mus musculus OX=10090 GN=Dnajc7 PE=1 SV=2	
ZN326_MOUSE	DBIRD complex subunit ZNF326 OS=Mus musculus OX=10090 GN=Znf326 PE=1 SV=1	
SHPS1_MOUSE	Tyrosine-protein phosphatase non-receptor type substrate 1 OS=Mus musculus OX=10090 GN=Sirpa PE=1 SV=2	
MBD3_MOUSE	Methyl-CpG-binding domain protein 3 OS=Mus musculus OX=10090 GN=Mbd3 PE=1 SV=1	
VPS52_MOUSE	Vacuolar protein sorting-associated protein 52 homolog OS=Mus musculus OX=10090 GN=Vps52 PE=1 SV=1	
SIR2_MOUSE	NAD-dependent protein deacetylase sirtuin-2 OS=Mus musculus OX=10090 GN=Sirt2 PE=1 SV=2	
NSDHL_MOUSE	Sterol-4-alpha-carboxylate 3-dehydrogenase, decarboxylating OS=Mus musculus OX=10090 GN=Nsdhl PE=1 SV=1	
T126A_MOUSE	Transmembrane protein 126A OS=Mus musculus OX=10090 GN=Tmem126a PE=1 SV=1	
NPL4_MOUSE	Nuclear protein localization protein 4 homolog OS=Mus musculus OX=10090 GN=Nploc4 PE=1 SV=3	
CPIN1_MOUSE	Anamorsin OS=Mus musculus OX=10090 GN=Ciapin1 PE=1 SV=1	
DC1I1_HUMAN	Cytoplasmic dynein 1 intermediate chain 1 OS=Homo sapiens OX=9606 GN=DYNC1I1 PE=1 SV=2	
BRSK1_MOUSE	Serine/threonine-protein kinase BRSK1 OS=Mus musculus OX=10090 GN=Brsk1 PE=1 SV=1	
DLG4_MOUSE	Disks large homolog 4 OS=Mus musculus OX=10090 GN=Dlg4 PE=1 SV=1	
UBP11_MOUSE	Ubiquitin carboxyl-terminal hydrolase 11 OS=Mus musculus OX=10090 GN=Usp11 PE=1 SV=4	
QRIC1_MOUSE	Glutamine-rich protein 1 OS=Mus musculus OX=10090 GN=Qrich1 PE=1 SV=1	
VPS4A_MOUSE	Vacuolar protein sorting-associated protein 4A OS=Mus musculus OX=10090 GN=Vps4a PE=1 SV=1	
FNTA_MOUSE	Protein farnesyltransferase/geranylgeranyltransferase type-1 subunit alpha OS=Mus musculus OX=10090 GN=Fnta PE=1 SV=1	
TUSC3_MOUSE	Tumor suppressor candidate 3 OS=Mus musculus OX=10090 GN=Tusc3 PE=1 SV=1	
ARRB1_MOUSE	Beta-arrestin-1 OS=Mus musculus OX=10090 GN=Arrb1 PE=1 SV=1	
HMGCL_MOUSE	Hydroxymethylglutaryl-CoA lyase, mitochondrial OS=Mus musculus OX=10090 GN=Hmgcl PE=1 SV=2	
CC181_MOUSE	Coiled-coil domain-containing protein 181 OS=Mus musculus OX=10090 GN=Ccdc181 PE=1 SV=1	
WDR82_MOUSE	WD repeat-containing protein 82 OS=Mus musculus OX=10090 GN=Wdr82 PE=1 SV=1	
STAU2_MOUSE	Double-stranded RNA-binding protein Staufen homolog 2 OS=Mus musculus OX=10090 GN=Stau2 PE=1 SV=1	
DNJB1_MOUSE	DnaJ homolog subfamily B member 1 OS=Mus musculus OX=10090 GN=Dnajb1 PE=1 SV=3	
RUN3A_MOUSE	RUN domain-containing protein 3A OS=Mus musculus OX=10090 GN=Rundc3a PE=1 SV=1	
ELMO2_MOUSE	Engulfment and cell motility protein 2 OS=Mus musculus OX=10090 GN=Elmo2 PE=1 SV=1	
BIG3_MOUSE	Brefeldin A-inhibited guanine nucleotide-exchange protein 3 OS=Mus musculus OX=10090 GN=Arfgef3 PE=1 SV=1	
PREP_MOUSE	Presequence protease, mitochondrial OS=Mus musculus OX=10090 GN=Pitrm1 PE=1 SV=1	
GCC2_MOUSE	GRIP and coiled-coil domain-containing protein 2 OS=Mus musculus OX=10090 GN=Gcc2 PE=1 SV=2	
CPEB3_MOUSE	Cytoplasmic polyadenylation element-binding protein 3 OS=Mus musculus OX=10090 GN=Cpeb3 PE=1 SV=1	
PDE2A_MOUSE	cGMP-dependent 3',5'-cyclic phosphodiesterase OS=Mus musculus OX=10090 GN=Pde2a PE=1 SV=4	
PIN1_MOUSE	Peptidyl-prolyl cis-trans isomerase NIMA-interacting 1 OS=Mus musculus OX=10090 GN=Pin1 PE=1 SV=1	
RRFM_MOUSE	Ribosome-recycling factor, mitochondrial OS=Mus musculus OX=10090 GN=Mrrf PE=1 SV=1	
VP37B_MOUSE	Vacuolar protein sorting-associated protein 37B OS=Mus musculus OX=10090 GN=Vps37b PE=1 SV=1	
TTYH1_MOUSE	Protein tweety homolog 1 OS=Mus musculus OX=10090 GN=Ttyh1 PE=1 SV=1	
RWDD1_MOUSE	RWD domain-containing protein 1 OS=Mus musculus OX=10090 GN=Rwdd1 PE=1 SV=1	
ACTN2_MOUSE	Alpha-actinin-2 OS=Mus musculus OX=10090 GN=Actn2 PE=1 SV=2	
TENA_MOUSE	Tenascin OS=Mus musculus OX=10090 GN=Tnc PE=1 SV=1	
FTO_MOUSE	Alpha-ketoglutarate-dependent dioxygenase FTO OS=Mus musculus OX=10090 GN=Fto PE=1 SV=1	
CK5P1_MOUSE	CDK5 regulatory subunit-associated protein 1 OS=Mus musculus OX=10090 GN=Cdk5rap1 PE=2 SV=2	
ACYP2_MOUSE	Acylphosphatase-2 OS=Mus musculus OX=10090 GN=Acyp2 PE=1 SV=2	
RPAB3_MOUSE	DNA-directed RNA polymerases I, II, and III subunit RPABC3 OS=Mus musculus OX=10090 GN=Polr2h PE=1 SV=3	
QCR10_MOUSE	Cytochrome b-c1 complex subunit 10 OS=Mus musculus OX=10090 GN=Uqcr11 PE=3 SV=1	
MYADM_MOUSE	Myeloid-associated differentiation marker OS=Mus musculus OX=10090 GN=Myadm PE=1 SV=2	
PP1G_MOUSE	Serine/threonine-protein phosphatase PP1-gamma catalytic subunit OS=Mus musculus OX=10090 GN=Ppp1cc PE=1 SV=1	
RRP1_MOUSE	Ribosomal RNA processing protein 1 homolog A OS=Mus musculus OX=10090 GN=Rrp1 PE=1 SV=2	
NEDD8_MOUSE	NEDD8 OS=Mus musculus OX=10090 GN=Nedd8 PE=1 SV=2	
HBB_MYOVE	Hemoglobin subunit beta OS=Myotis velifer OX=9435 GN=HBB PE=1 SV=1	
ZN428_MOUSE	Zinc finger protein 428 OS=Mus musculus OX=10090 GN=Znf428 PE=1 SV=1	
WASF3_MOUSE	Wiskott-Aldrich syndrome protein family member 3 OS=Mus musculus OX=10090 GN=Wasf3 PE=1 SV=1	
SESD1_MOUSE	SEC14 domain and spectrin repeat-containing protein 1 OS=Mus musculus OX=10090 GN=Sestd1 PE=1 SV=1	
Q9SJL2-DECOY	Q9SJL2	
HIG1A_MOUSE	HIG1 domain family member 1A, mitochondrial OS=Mus musculus OX=10090 GN=Higd1a PE=1 SV=1	
SC61G_MOUSE	Protein transport protein Sec61 subunit gamma OS=Mus musculus OX=10090 GN=Sec61g PE=3 SV=1	
2AAB_MOUSE	Serine/threonine-protein phosphatase 2A 65 kDa regulatory subunit A beta isoform OS=Mus musculus OX=10090 GN=Ppp2r1b PE=1 SV=2	
NU3M_MOUSE	NADH-ubiquinone oxidoreductase chain 3 OS=Mus musculus OX=10090 GN=Mtnd3 PE=1 SV=3	
PTH2_MOUSE	Peptidyl-tRNA hydrolase 2, mitochondrial OS=Mus musculus OX=10090 GN=Ptrh2 PE=1 SV=1	
LCLT1_MOUSE	Lysocardiolipin acyltransferase 1 OS=Mus musculus OX=10090 GN=Lclat1 PE=1 SV=2	
CDN1B_MOUSE	Cyclin-dependent kinase inhibitor 1B OS=Mus musculus OX=10090 GN=Cdkn1b PE=1 SV=2	
7B2_MOUSE	Neuroendocrine protein 7B2 OS=Mus musculus OX=10090 GN=Scg5 PE=1 SV=1	
MYPT2_MOUSE	Protein phosphatase 1 regulatory subunit 12B OS=Mus musculus OX=10090 GN=Ppp1r12b PE=1 SV=2	
FAHD2_MOUSE	Fumarylacetoacetate hydrolase domain-containing protein 2A OS=Mus musculus OX=10090 GN=Fahd2 PE=1 SV=1	
C1RA_MOUSE	Complement C1r-A subcomponent OS=Mus musculus OX=10090 GN=C1ra PE=1 SV=1	
GLRX5_MOUSE	Glutaredoxin-related protein 5, mitochondrial OS=Mus musculus OX=10090 GN=Glrx5 PE=1 SV=2	
EMC4_MOUSE	ER membrane protein complex subunit 4 OS=Mus musculus OX=10090 GN=Emc4 PE=1 SV=1	
ARMC1_MOUSE	Armadillo repeat-containing protein 1 OS=Mus musculus OX=10090 GN=Armc1 PE=1 SV=1	
LIPL_MOUSE	Lipoprotein lipase OS=Mus musculus OX=10090 GN=Lpl PE=1 SV=3	
RL29_MOUSE	60S ribosomal protein L29 OS=Mus musculus OX=10090 GN=Rpl29 PE=1 SV=2	
SDC4_MOUSE	Syndecan-4 OS=Mus musculus OX=10090 GN=Sdc4 PE=1 SV=1	
NET1_MOUSE	Netrin-1 OS=Mus musculus OX=10090 GN=Ntn1 PE=1 SV=3	
RM14_MOUSE	39S ribosomal protein L14, mitochondrial OS=Mus musculus OX=10090 GN=Mrpl14 PE=1 SV=1	
KCD19_MOUSE	BTB/POZ domain-containing protein KCTD19 OS=Mus musculus OX=10090 GN=Kctd19 PE=1 SV=2	
SDC3_MOUSE	Syndecan-3 OS=Mus musculus OX=10090 GN=Sdc3 PE=1 SV=2	
DJB12_MOUSE	DnaJ homolog subfamily B member 12 OS=Mus musculus OX=10090 GN=Dnajb12 PE=1 SV=2	
CAND2_MOUSE	Cullin-associated NEDD8-dissociated protein 2 OS=Mus musculus OX=10090 GN=Cand2 PE=1 SV=2	
CPSF3_MOUSE	Cleavage and polyadenylation specificity factor subunit 3 OS=Mus musculus OX=10090 GN=Cpsf3 PE=1 SV=2	
PXL2A_MOUSE	Peroxiredoxin-like 2A OS=Mus musculus OX=10090 GN=Prxl2a PE=1 SV=2	
DHX29_MOUSE	ATP-dependent RNA helicase DHX29 OS=Mus musculus OX=10090 GN=Dhx29 PE=1 SV=1	
MAST4_MOUSE	Microtubule-associated serine/threonine-protein kinase 4 OS=Mus musculus OX=10090 GN=Mast4 PE=1 SV=3	
P4K2A_MOUSE	Phosphatidylinositol 4-kinase type 2-alpha OS=Mus musculus OX=10090 GN=Pi4k2a PE=1 SV=1	
BGLR_MOUSE	Beta-glucuronidase OS=Mus musculus OX=10090 GN=Gusb PE=1 SV=2	
ARFP2_MOUSE	Arfaptin-2 OS=Mus musculus OX=10090 GN=Arfip2 PE=1 SV=2	
RT02_MOUSE	28S ribosomal protein S2, mitochondrial OS=Mus musculus OX=10090 GN=Mrps2 PE=1 SV=1	
HOME1_MOUSE	Homer protein homolog 1 OS=Mus musculus OX=10090 GN=Homer1 PE=1 SV=2	
MOGS_MOUSE	Mannosyl-oligosaccharide glucosidase OS=Mus musculus OX=10090 GN=Mogs PE=1 SV=1	
ERGI3_MOUSE	Endoplasmic reticulum-Golgi intermediate compartment protein 3 OS=Mus musculus OX=10090 GN=Ergic3 PE=1 SV=1	
MTAP_MOUSE	S-methyl-5'-thioadenosine phosphorylase OS=Mus musculus OX=10090 GN=Mtap PE=1 SV=1	
SMCE1_MOUSE	SWI/SNF-related matrix-associated actin-dependent regulator of chromatin subfamily E member 1 OS=Mus musculus OX=10090 GN=Smarce1 PE=1 SV=1	
WFS1_MOUSE	Wolframin OS=Mus musculus OX=10090 GN=Wfs1 PE=1 SV=1	
PLCA_MOUSE	1-acyl-sn-glycerol-3-phosphate acyltransferase alpha OS=Mus musculus OX=10090 GN=Agpat1 PE=1 SV=1	
NDEL1_MOUSE	Nuclear distribution protein nudE-like 1 OS=Mus musculus OX=10090 GN=Ndel1 PE=1 SV=2	
VAC14_MOUSE	Protein VAC14 homolog OS=Mus musculus OX=10090 GN=Vac14 PE=1 SV=1	
MYO5A_HUMAN	Unconventional myosin-Va OS=Homo sapiens OX=9606 GN=MYO5A PE=1 SV=2	
SACS_MOUSE	Sacsin OS=Mus musculus OX=10090 GN=Sacs PE=1 SV=2	
5NTC_MOUSE	Cytosolic purine 5'-nucleotidase OS=Mus musculus OX=10090 GN=Nt5c2 PE=1 SV=2	
CCHL_MOUSE	Cytochrome c-type heme lyase OS=Mus musculus OX=10090 GN=Hccs PE=1 SV=2	
FPRP_MOUSE	Prostaglandin F2 receptor negative regulator OS=Mus musculus OX=10090 GN=Ptgfrn PE=1 SV=2	
MS5L2_ARATH	Protein POLLENLESS 3-LIKE 2 OS=Arabidopsis thaliana OX=3702 GN=At3g51280 PE=2 SV=1	
CLVS2_MOUSE	Clavesin-2 OS=Mus musculus OX=10090 GN=Clvs2 PE=1 SV=1	
DOCK3_MOUSE	Dedicator of cytokinesis protein 3 OS=Mus musculus OX=10090 GN=Dock3 PE=1 SV=1	
CATZ_MOUSE	Cathepsin Z OS=Mus musculus OX=10090 GN=Ctsz PE=1 SV=1	
T132A_MOUSE	Transmembrane protein 132A OS=Mus musculus OX=10090 GN=Tmem132a PE=1 SV=2	
NSG2_MOUSE	Neuronal vesicle trafficking-associated protein 2 OS=Mus musculus OX=10090 GN=Nsg2 PE=1 SV=1	
AL3A2_MOUSE	Aldehyde dehydrogenase family 3 member A2 OS=Mus musculus OX=10090 GN=Aldh3a2 PE=1 SV=2	
A8R7K9-DECOY	A8R7K9	
TCRG1_MOUSE	Transcription elongation regulator 1 OS=Mus musculus OX=10090 GN=Tcerg1 PE=1 SV=2	
ABCF2_MOUSE	ATP-binding cassette sub-family F member 2 OS=Mus musculus OX=10090 GN=Abcf2 PE=1 SV=1	
TENR_MOUSE	Tenascin-R OS=Mus musculus OX=10090 GN=Tnr PE=1 SV=2	
PEX19_MOUSE	Peroxisomal biogenesis factor 19 OS=Mus musculus OX=10090 GN=Pex19 PE=1 SV=1	
ERI3_MOUSE	ERI1 exoribonuclease 3 OS=Mus musculus OX=10090 GN=Eri3 PE=1 SV=1	
TMCO1_MOUSE	Calcium load-activated calcium channel OS=Mus musculus OX=10090 GN=Tmco1 PE=1 SV=1	
UBA3_MOUSE	NEDD8-activating enzyme E1 catalytic subunit OS=Mus musculus OX=10090 GN=Uba3 PE=1 SV=2	
IF2P_MOUSE	Eukaryotic translation initiation factor 5B OS=Mus musculus OX=10090 GN=Eif5b PE=1 SV=2	
GTR3_MOUSE	Solute carrier family 2, facilitated glucose transporter member 3 OS=Mus musculus OX=10090 GN=Slc2a3 PE=1 SV=1	
ACL6B_MOUSE	Actin-like protein 6B OS=Mus musculus OX=10090 GN=Actl6b PE=1 SV=1	
ARM10_MOUSE	Armadillo repeat-containing protein 10 OS=Mus musculus OX=10090 GN=Armc10 PE=1 SV=1	
ACAD8_MOUSE	Isobutyryl-CoA dehydrogenase, mitochondrial OS=Mus musculus OX=10090 GN=Acad8 PE=1 SV=2	
PGTB2_MOUSE	Geranylgeranyl transferase type-2 subunit beta OS=Mus musculus OX=10090 GN=Rabggtb PE=1 SV=2	
ATIF1_MOUSE	ATPase inhibitor, mitochondrial OS=Mus musculus OX=10090 GN=ATP5IF1 PE=1 SV=2	
LARP7_MOUSE	La-related protein 7 OS=Mus musculus OX=10090 GN=Larp7 PE=1 SV=2	
AFAP1_MOUSE	Actin filament-associated protein 1 OS=Mus musculus OX=10090 GN=Afap1 PE=1 SV=1	
KALRN_MOUSE	Kalirin OS=Mus musculus OX=10090 GN=Kalrn PE=1 SV=1	
CON__P08779	CON__P08779	
UBP24_MOUSE	Ubiquitin carboxyl-terminal hydrolase 24 OS=Mus musculus OX=10090 GN=Usp24 PE=1 SV=1	
MYO1C_MOUSE	Unconventional myosin-Ic OS=Mus musculus OX=10090 GN=Myo1c PE=1 SV=2	
COA3_MOUSE	Cytochrome c oxidase assembly factor 3 homolog, mitochondrial OS=Mus musculus OX=10090 GN=Coa3 PE=1 SV=1	
SYUB_MOUSE	Beta-synuclein OS=Mus musculus OX=10090 GN=Sncb PE=1 SV=1	
LUC7L_MOUSE	Putative RNA-binding protein Luc7-like 1 OS=Mus musculus OX=10090 GN=Luc7l PE=1 SV=2	
PCSK1_MOUSE	ProSAAS OS=Mus musculus OX=10090 GN=Pcsk1n PE=1 SV=2	
CP46A_MOUSE	Cholesterol 24-hydroxylase OS=Mus musculus OX=10090 GN=Cyp46a1 PE=1 SV=1	
GBRA3_MOUSE	Gamma-aminobutyric acid receptor subunit alpha-3 OS=Mus musculus OX=10090 GN=Gabra3 PE=1 SV=1	
TIM8B_MOUSE	Mitochondrial import inner membrane translocase subunit Tim8 B OS=Mus musculus OX=10090 GN=Timm8b PE=1 SV=1	
MPPB_MOUSE	Mitochondrial-processing peptidase subunit beta OS=Mus musculus OX=10090 GN=Pmpcb PE=1 SV=1	
PP4R2_MOUSE	Serine/threonine-protein phosphatase 4 regulatory subunit 2 OS=Mus musculus OX=10090 GN=Ppp4r2 PE=1 SV=1	
GBG12_MOUSE	Guanine nucleotide-binding protein G(I)/G(S)/G(O) subunit gamma-12 OS=Mus musculus OX=10090 GN=Gng12 PE=1 SV=3	
COASY_MOUSE	Bifunctional coenzyme A synthase OS=Mus musculus OX=10090 GN=Coasy PE=1 SV=2	
NIT1_MOUSE	Deaminated glutathione amidase OS=Mus musculus OX=10090 GN=Nit1 PE=1 SV=2	
TMM33_MOUSE	Transmembrane protein 33 OS=Mus musculus OX=10090 GN=Tmem33 PE=1 SV=1	
ARF2_MOUSE	ADP-ribosylation factor 2 OS=Mus musculus OX=10090 GN=Arf2 PE=1 SV=2	
TF3C5_MOUSE	General transcription factor 3C polypeptide 5 OS=Mus musculus OX=10090 GN=Gtf3c5 PE=2 SV=2	
PEX14_MOUSE	Peroxisomal membrane protein PEX14 OS=Mus musculus OX=10090 GN=Pex14 PE=1 SV=1	
TM163_MOUSE	Transmembrane protein 163 OS=Mus musculus OX=10090 GN=Tmem163 PE=1 SV=1	
S10AB_MOUSE	Protein S100-A11 OS=Mus musculus OX=10090 GN=S100a11 PE=1 SV=1	
QOR_MOUSE	Quinone oxidoreductase OS=Mus musculus OX=10090 GN=Cryz PE=1 SV=1	
ACOX1_ARATH	Peroxisomal acyl-coenzyme A oxidase 1 OS=Arabidopsis thaliana OX=3702 GN=ACX1 PE=1 SV=1	
OTC_MOUSE	Ornithine carbamoyltransferase, mitochondrial OS=Mus musculus OX=10090 GN=Otc PE=1 SV=1	
LACTB_MOUSE	Serine beta-lactamase-like protein LACTB, mitochondrial OS=Mus musculus OX=10090 GN=Lactb PE=1 SV=1	
ZYX_MOUSE	Zyxin OS=Mus musculus OX=10090 GN=Zyx PE=1 SV=2	
NACAD_MOUSE	NAC-alpha domain-containing protein 1 OS=Mus musculus OX=10090 GN=Nacad PE=1 SV=1	
HAT1_MOUSE	Histone acetyltransferase type B catalytic subunit OS=Mus musculus OX=10090 GN=Hat1 PE=1 SV=1	
TPC6B_MOUSE	Trafficking protein particle complex subunit 6B OS=Mus musculus OX=10090 GN=Trappc6b PE=1 SV=1	
F169A_MOUSE	Soluble lamin-associated protein of 75 kDa OS=Mus musculus OX=10090 GN=Fam169a PE=1 SV=3	
CPSF1_MOUSE	Cleavage and polyadenylation specificity factor subunit 1 OS=Mus musculus OX=10090 GN=Cpsf1 PE=1 SV=1	
RN214_MOUSE	RING finger protein 214 OS=Mus musculus OX=10090 GN=Rnf214 PE=1 SV=1	
EVI5_MOUSE	Ecotropic viral integration site 5 protein OS=Mus musculus OX=10090 GN=Evi5 PE=1 SV=2	
MMGT1_MOUSE	Membrane magnesium transporter 1 OS=Mus musculus OX=10090 GN=Mmgt1 PE=1 SV=1	
TIAM1_MOUSE	T-lymphoma invasion and metastasis-inducing protein 1 OS=Mus musculus OX=10090 GN=Tiam1 PE=1 SV=1	
BABA2_CHLAE	BRISC and BRCA1-A complex member 2 OS=Chlorocebus aethiops OX=9534 GN=BABAM2 PE=2 SV=1	
Q9WUH2-DECOY	Q9WUH2	
FACE1_MOUSE	CAAX prenyl protease 1 homolog OS=Mus musculus OX=10090 GN=Zmpste24 PE=1 SV=2	
UBE3C_MOUSE	Ubiquitin-protein ligase E3C OS=Mus musculus OX=10090 GN=Ube3c PE=1 SV=2	
VAMP4_MOUSE	Vesicle-associated membrane protein 4 OS=Mus musculus OX=10090 GN=Vamp4 PE=1 SV=1	
MPI_MOUSE	Mannose-6-phosphate isomerase OS=Mus musculus OX=10090 GN=Mpi PE=1 SV=1	
TSN7_MOUSE	Tetraspanin-7 OS=Mus musculus OX=10090 GN=Tspan7 PE=1 SV=2	
SCG1_MOUSE	Secretogranin-1 OS=Mus musculus OX=10090 GN=Chgb PE=1 SV=2	
P66B_MOUSE	Transcriptional repressor p66-beta OS=Mus musculus OX=10090 GN=Gatad2b PE=1 SV=1	
Q1PEY6-DECOY	Q1PEY6	
SMRD1_MOUSE	SWI/SNF-related matrix-associated actin-dependent regulator of chromatin subfamily D member 1 OS=Mus musculus OX=10090 GN=Smarcd1 PE=1 SV=3	
PELP1_MOUSE	Proline-, glutamic acid- and leucine-rich protein 1 OS=Mus musculus OX=10090 GN=Pelp1 PE=1 SV=2	
HSP13_MOUSE	Heat shock 70 kDa protein 13 OS=Mus musculus OX=10090 GN=Hspa13 PE=1 SV=1	
C560_MOUSE	Succinate dehydrogenase cytochrome b560 subunit, mitochondrial OS=Mus musculus OX=10090 GN=Sdhc PE=1 SV=1	
HDAC1_MOUSE	Histone deacetylase 1 OS=Mus musculus OX=10090 GN=Hdac1 PE=1 SV=1	
CCAR1_MOUSE	Cell division cycle and apoptosis regulator protein 1 OS=Mus musculus OX=10090 GN=Ccar1 PE=1 SV=1	
CPNE1_MOUSE	Copine-1 OS=Mus musculus OX=10090 GN=Cpne1 PE=1 SV=1	
TMM11_MOUSE	Transmembrane protein 11, mitochondrial OS=Mus musculus OX=10090 GN=Tmem11 PE=1 SV=1	
SFRP1_MOUSE	Secreted frizzled-related protein 1 OS=Mus musculus OX=10090 GN=Sfrp1 PE=1 SV=3	
MYPT1_MOUSE	Protein phosphatase 1 regulatory subunit 12A OS=Mus musculus OX=10090 GN=Ppp1r12a PE=1 SV=2	
GBB4_MOUSE	Guanine nucleotide-binding protein subunit beta-4 OS=Mus musculus OX=10090 GN=Gnb4 PE=1 SV=4	
CAN5_MOUSE	Calpain-5 OS=Mus musculus OX=10090 GN=Capn5 PE=1 SV=1	
SPRY4_MOUSE	SPRY domain-containing protein 4 OS=Mus musculus OX=10090 GN=Spryd4 PE=1 SV=1	
APEH_MOUSE	Acylamino-acid-releasing enzyme OS=Mus musculus OX=10090 GN=Apeh PE=1 SV=3	
GSH0_MOUSE	Glutamate--cysteine ligase regulatory subunit OS=Mus musculus OX=10090 GN=Gclm PE=1 SV=1	
SAM50_MOUSE	Sorting and assembly machinery component 50 homolog OS=Mus musculus OX=10090 GN=Samm50 PE=1 SV=1	
DEN10_MOUSE	DENN domain-containing protein 10 OS=Mus musculus OX=10090 GN=Dennd10 PE=1 SV=2	
Y1285_ARATH	Putative UPF0725 protein At1g28500 OS=Arabidopsis thaliana OX=3702 GN=At1g28500 PE=3 SV=1	
PMVK_MOUSE	Phosphomevalonate kinase OS=Mus musculus OX=10090 GN=Pmvk PE=1 SV=3	
GAPD1_MOUSE	GTPase-activating protein and VPS9 domain-containing protein 1 OS=Mus musculus OX=10090 GN=Gapvd1 PE=1 SV=2	
A2AGT5-DECOY	A2AGT5	
IBP3_MOUSE	Insulin-like growth factor-binding protein 3 OS=Mus musculus OX=10090 GN=Igfbp3 PE=2 SV=2	
TGRM1_MOUSE	TOG array regulator of axonemal microtubules protein 1 OS=Mus musculus OX=10090 GN=Togaram1 PE=1 SV=3	
TACO1_MOUSE	Translational activator of cytochrome c oxidase 1 OS=Mus musculus OX=10090 GN=Taco1 PE=1 SV=1	
ACBD6_MOUSE	Acyl-CoA-binding domain-containing protein 6 OS=Mus musculus OX=10090 GN=Acbd6 PE=1 SV=2	
TNPO3_MOUSE	Transportin-3 OS=Mus musculus OX=10090 GN=Tnpo3 PE=1 SV=1	
PCD10_HUMAN	Protocadherin-10 OS=Homo sapiens OX=9606 GN=PCDH10 PE=2 SV=2	
CHTOP_MOUSE	Chromatin target of PRMT1 protein OS=Mus musculus OX=10090 GN=Chtop PE=1 SV=2	
ARHL2_MOUSE	ADP-ribose glycohydrolase ARH3 OS=Mus musculus OX=10090 GN=Adprhl2 PE=1 SV=1	
QCR6_MOUSE	Cytochrome b-c1 complex subunit 6, mitochondrial OS=Mus musculus OX=10090 GN=Uqcrh PE=1 SV=2	
FXYD6_MOUSE	FXYD domain-containing ion transport regulator 6 OS=Mus musculus OX=10090 GN=Fxyd6 PE=1 SV=2	
RFOX1_MOUSE	RNA binding protein fox-1 homolog 1 OS=Mus musculus OX=10090 GN=Rbfox1 PE=1 SV=3	
DPYS_MOUSE	Dihydropyrimidinase OS=Mus musculus OX=10090 GN=Dpys PE=1 SV=2	
S2539_MOUSE	Solute carrier family 25 member 39 OS=Mus musculus OX=10090 GN=Slc25a39 PE=2 SV=1	
YES_MOUSE	Tyrosine-protein kinase Yes OS=Mus musculus OX=10090 GN=Yes1 PE=1 SV=3	
UBQL1_MOUSE	Ubiquilin-1 OS=Mus musculus OX=10090 GN=Ubqln1 PE=1 SV=1	
MYL9_MOUSE	Myosin regulatory light polypeptide 9 OS=Mus musculus OX=10090 GN=Myl9 PE=1 SV=3	
CF97D_MOUSE	Uncharacterized protein CFAP97D1 OS=Mus musculus OX=10090 GN=Cfap97d1 PE=2 SV=1	
CC115_MOUSE	Coiled-coil domain-containing protein 115 OS=Mus musculus OX=10090 GN=Ccdc115 PE=1 SV=1	
WRB_MOUSE	Tail-anchored protein insertion receptor WRB OS=Mus musculus OX=10090 GN=Wrb PE=1 SV=1	
SCAI_MOUSE	Protein SCAI OS=Mus musculus OX=10090 GN=Scai PE=1 SV=2	
IHH_MOUSE	Indian hedgehog protein OS=Mus musculus OX=10090 GN=Ihh PE=1 SV=2	
RAB2B_MOUSE	Ras-related protein Rab-2B OS=Mus musculus OX=10090 GN=Rab2b PE=1 SV=1	
PP4C_MOUSE	Serine/threonine-protein phosphatase 4 catalytic subunit OS=Mus musculus OX=10090 GN=Ppp4c PE=1 SV=2	
SPZ1_ARATH	Serpin-Z1 OS=Arabidopsis thaliana OX=3702 GN=At1g64030 PE=2 SV=2	
KAD5_MOUSE	Adenylate kinase isoenzyme 5 OS=Mus musculus OX=10090 GN=Ak5 PE=1 SV=2	
RM46_MOUSE	39S ribosomal protein L46, mitochondrial OS=Mus musculus OX=10090 GN=Mrpl46 PE=1 SV=1	
RT10_MOUSE	28S ribosomal protein S10, mitochondrial OS=Mus musculus OX=10090 GN=Mrps10 PE=1 SV=1	
LCAP_MOUSE	Leucyl-cystinyl aminopeptidase OS=Mus musculus OX=10090 GN=Lnpep PE=1 SV=1	
TSNAX_MOUSE	Translin-associated protein X OS=Mus musculus OX=10090 GN=Tsnax PE=1 SV=1	
ABCB7_MOUSE	ATP-binding cassette sub-family B member 7, mitochondrial OS=Mus musculus OX=10090 GN=Abcb7 PE=1 SV=3	
PARVA_MOUSE	Alpha-parvin OS=Mus musculus OX=10090 GN=Parva PE=1 SV=1	
ACM1_MOUSE	Muscarinic acetylcholine receptor M1 OS=Mus musculus OX=10090 GN=Chrm1 PE=1 SV=2	
RCAN2_MOUSE	Calcipressin-2 OS=Mus musculus OX=10090 GN=Rcan2 PE=1 SV=1	
SPF27_MOUSE	Pre-mRNA-splicing factor SPF27 OS=Mus musculus OX=10090 GN=Bcas2 PE=1 SV=1	
CBX6_MOUSE	Chromobox protein homolog 6 OS=Mus musculus OX=10090 GN=Cbx6 PE=1 SV=2	
RCN3_MOUSE	Reticulocalbin-3 OS=Mus musculus OX=10090 GN=Rcn3 PE=1 SV=1	
KV3A8_MOUSE	Ig kappa chain V-III region PC 3741/TEPC 111 OS=Mus musculus OX=10090 PE=1 SV=1	
HMGN1_MOUSE	Non-histone chromosomal protein HMG-14 OS=Mus musculus OX=10090 GN=Hmgn1 PE=1 SV=2	
CNOT9_MOUSE	CCR4-NOT transcription complex subunit 9 OS=Mus musculus OX=10090 GN=Cnot9 PE=1 SV=1	
SBP1_RAT	Methanethiol oxidase OS=Rattus norvegicus OX=10116 GN=Selenbp1 PE=1 SV=1	
MAON_MOUSE	NADP-dependent malic enzyme, mitochondrial OS=Mus musculus OX=10090 GN=Me3 PE=1 SV=2	
TIM10_MOUSE	Mitochondrial import inner membrane translocase subunit Tim10 OS=Mus musculus OX=10090 GN=Timm10 PE=1 SV=1	
ARHG7_MOUSE	Rho guanine nucleotide exchange factor 7 OS=Mus musculus OX=10090 GN=Arhgef7 PE=1 SV=2	
GCP60_MOUSE	Golgi resident protein GCP60 OS=Mus musculus OX=10090 GN=Acbd3 PE=1 SV=3	
MK_MOUSE	Midkine OS=Mus musculus OX=10090 GN=Mdk PE=1 SV=2	
CSN8_MOUSE	COP9 signalosome complex subunit 8 OS=Mus musculus OX=10090 GN=Cops8 PE=1 SV=1	
CON__Q15323	CON__Q15323	
CCD22_MOUSE	Coiled-coil domain-containing protein 22 OS=Mus musculus OX=10090 GN=Ccdc22 PE=1 SV=1	
KT3K_MOUSE	Ketosamine-3-kinase OS=Mus musculus OX=10090 GN=Fn3krp PE=1 SV=2	
UBR3_MOUSE	E3 ubiquitin-protein ligase UBR3 OS=Mus musculus OX=10090 GN=Ubr3 PE=1 SV=3	
MIRO2_MOUSE	Mitochondrial Rho GTPase 2 OS=Mus musculus OX=10090 GN=Rhot2 PE=1 SV=1	
MTSS2_MOUSE	Protein MTSS 2 OS=Mus musculus OX=10090 GN=Mtss2 PE=1 SV=1	
COCA1_HUMAN	Collagen alpha-1(XII) chain OS=Homo sapiens OX=9606 GN=COL12A1 PE=1 SV=2	
T22D1_MOUSE	TSC22 domain family protein 1 OS=Mus musculus OX=10090 GN=Tsc22d1 PE=1 SV=2	
ZC3HF_HUMAN	Zinc finger CCCH domain-containing protein 15 OS=Homo sapiens OX=9606 GN=ZC3H15 PE=1 SV=1	
EXOC8_MOUSE	Exocyst complex component 8 OS=Mus musculus OX=10090 GN=Exoc8 PE=1 SV=1	
SNX30_MOUSE	Sorting nexin-30 OS=Mus musculus OX=10090 GN=Snx30 PE=1 SV=1	
MON2_MOUSE	Protein MON2 homolog OS=Mus musculus OX=10090 GN=Mon2 PE=1 SV=2	
AKP8L_MOUSE	A-kinase anchor protein 8-like OS=Mus musculus OX=10090 GN=Akap8l PE=1 SV=1	
KI26B_MOUSE	Kinesin-like protein KIF26B OS=Mus musculus OX=10090 GN=Kif26b PE=1 SV=3	
TBCE_MOUSE	Tubulin-specific chaperone E OS=Mus musculus OX=10090 GN=Tbce PE=1 SV=1	
NU5M_MOUSE	NADH-ubiquinone oxidoreductase chain 5 OS=Mus musculus OX=10090 GN=Mtnd5 PE=1 SV=3	
PPM1L_MOUSE	Protein phosphatase 1L OS=Mus musculus OX=10090 GN=Ppm1l PE=1 SV=1	
CON__Q3ZBS7	CON__Q3ZBS7	
PESC_MOUSE	Pescadillo homolog OS=Mus musculus OX=10090 GN=Pes1 PE=1 SV=1	
NRP1_MOUSE	Neuropilin-1 OS=Mus musculus OX=10090 GN=Nrp1 PE=1 SV=2	
LYPA2_MOUSE	Acyl-protein thioesterase 2 OS=Mus musculus OX=10090 GN=Lypla2 PE=1 SV=1	
DCMC_MOUSE	Malonyl-CoA decarboxylase, mitochondrial OS=Mus musculus OX=10090 GN=Mlycd PE=1 SV=1	
KN7L_ARATH	Kinesin-like protein KIN-7L, chloroplastic OS=Arabidopsis thaliana OX=3702 GN=KIN7L PE=3 SV=2	
MA7D2_MOUSE	MAP7 domain-containing protein 2 OS=Mus musculus OX=10090 GN=Map7d2 PE=1 SV=1	
A16L1_HUMAN	Autophagy-related protein 16-1 OS=Homo sapiens OX=9606 GN=ATG16L1 PE=1 SV=2	
CDC23_ARATH	Anaphase-promoting complex subunit 8 OS=Arabidopsis thaliana OX=3702 GN=APC8 PE=1 SV=1	
IMA1_MOUSE	Importin subunit alpha-1 OS=Mus musculus OX=10090 GN=Kpna2 PE=1 SV=2	
O48671-DECOY	O48671	
ODPAT_MOUSE	Pyruvate dehydrogenase E1 component subunit alpha, testis-specific form, mitochondrial OS=Mus musculus OX=10090 GN=Pdha2 PE=1 SV=1	
PGPI_MOUSE	Pyroglutamyl-peptidase 1 OS=Mus musculus OX=10090 GN=Pgpep1 PE=1 SV=1	
PRPS2_MOUSE	Ribose-phosphate pyrophosphokinase 2 OS=Mus musculus OX=10090 GN=Prps2 PE=1 SV=4	
SFR1_MOUSE	Swi5-dependent recombination DNA repair protein 1 homolog OS=Mus musculus OX=10090 GN=Sfr1 PE=1 SV=2	
TXD12_MOUSE	Thioredoxin domain-containing protein 12 OS=Mus musculus OX=10090 GN=Txndc12 PE=1 SV=1	
UQCC1_MOUSE	Ubiquinol-cytochrome-c reductase complex assembly factor 1 OS=Mus musculus OX=10090 GN=Uqcc1 PE=1 SV=1	
ZN235_HUMAN	Zinc finger protein 235 OS=Homo sapiens OX=9606 GN=ZNF235 PE=2 SV=3	
