# Supplementary material for: CPEB3 low-complexity motif regulates local protein synthesis via protein–protein interactions in neuronal ribonucleoprotein granules
Source: Proc Natl Acad Sci U S A. 2023 Jan 30;120(6):e2114747120. doi: 10.1073/pnas.2114747120 (PMC9964033; doi:10.1073/pnas.2114747120)
Supplement: Supplementary file 4 — Dataset S03 (RTF) [file pnas.2114747120.sd03.rtf]

Supplementary Data 3. CPEB3 and S240-242A interactome analysis using Fischer's t-test.


Significant(x)	Significant(y)	Accession	Nonsignificant(x)	Nonsignificant(y)	Accession	Significant Outlier(x)	Significant Outlier(y)	Accession	Nonsignificant Outlier(x)	Nonsignificant Outlier(y)	Accession	Significance Threshold(x)	Significance Threshold(y)	Currently Selected(x)	Currently Selected(y)	Accession	
-4.83906	1.361239	Q8BIE6  Frmd4a - FERM domain-containing protein 4A	-4.40979	1.271818	O70305	-10	1.440976	E9PYH6 Histone-lysine N-methyltransferase SETD1A setd1a	-10	0.657688	A2A6Q5	-11	1.30103	-3.05852	2.478424	P56183	
-4.32527	1.366161	Q9DBG5 Perilipin-3 Plin3	-4.36531	0.896096	Q62261	-10	1.360291	O88736 3-keto-steroid reductase hsd17b7	-10	0.657688	A2AN08-3-DECOY	11	1.30103	-3.05852	2.478424	Q5SWD9	
-4.22609	4.87748	Q99J09 Methylosome protein 50 Wdr77	-4.13528	1.187085	Q6ZQ88	-10	1.360291	O89090 Transcription factor Sp1	-10	0.657688	A2AAE1			-3.05852	2.478424	Q9CX11	
-4.20285	1.328629	Q9WV80 Sorting nexin-1 Snx1	-4.13528	1.187085	Q8CHP8	-10	1.440976	O08529	-10	0.657688	D3YZP9						
-4.16946	1.336501	Q8CAY6 Acetyl-CoA acetyltransferase, cytosolic Acat2	-4.13528	1.187085	Q91XI1	-10	1.440976	O35609	-10	0.657688	A2AG50						
-4.03239	1.337855	Q9CSN1 SNW domain-containing protein 1 Snw1	-4.06906	1.246592	Q8K3Y3	-10	1.360291	P31324	-10	0.657688	A2APB8						
-4.03239	1.337855	Q9D662 Protein transport protein Sec23B Sec23b	-4.06906	1.246592	Q9CR00	-10	1.440976	O54916	-10	0.657688	O35465						
-3.95864	1.322625	Q60864 Stress-induced-phosphoprotein 1 Stip1	-4.02759	0.604373	Q9ER72-2	-10	1.440976	O89112	-10	0.657688	A2ARZ3-DECOY					
-3.72785	1.71656	Q9CWK8 Sorting nexin-2 Snx2	-3.83906	1.275149	Q91W39	-10	1.440976	P15105	-10	0.907805	Q9D554						
-3.61772	1.312171	Q9Z1R2 Large proline-rich protein BAG6 Bag6	-3.79182	1.172485	P28738	-10	1.440976	P20357	-10	1.175008	E9QAT4						
-3.58913	3.33316	Q60749 KH domain-containing, RNA-binding, signal transduction-associated protein 1 Khdrbs1	-3.71166	1.181016	Q9D8S3	-10	1.360291	P56960	-10	0.657688	E9Q4N7						
-3.58668	3.075978	Q923D4 Splicing factor 3B subunit 5 Sf3b5	-3.68827	1.26594	Q6P5D8	-10	1.440976	P23249	-10	0.657688	O08586						
-3.43358	1.643777	P61087 Ubiquitin-conjugating enzyme E2 K Ube2k	-3.6645	1.285921	Q9CQJ4	-10	1.440976	P29595	-10	0.657688	O70439						
-3.3762	3.086814	Q9CYG7 Mitochondrial import receptor subunit TOM34 Tomm34	-3.65684	1.272249	A2BH40	-10	1.360291	P97496	-10	0.657688	O70480						
-3.31644	2.128936	Q9Z2D0 Myotubularin-related protein 9 Mtmr9	-3.63973	1.255524	Q6P1F6	-10	1.440976	P35922	-10	0.99214	O54833						
-3.20213	1.787205	Q5SVQ0 Histone acetyltransferase KAT7 kat7	-3.61931	1.081505	P97742	-10	1.360291	P99028	-10	0.657688	P11531						
-3.05852	2.478424	P56183 Ribosomal RNA processing protein 1 homolog A rrp1	-3.61788	1.254844	Q810A7	-10	1.440976	P45481	-10	0.657688	O08791						
-3.05852	2.478424	Q5SWD9 Pre-rRNA-processing protein TSR1 homolog tsr1	-3.61575	1.102473	Q61749	-10	1.440976	P54276	-10	0.657688	P16546						
-3.05852	2.478424	Q9CX11 rRNA-processing protein UTP23 homolog utp23	-3.56861	1.268681	O88291	-10	5.152007	Q9CR26	-10	0.657688	O09110						
-3.02546	1.757339	Q8CHY6 Transcriptional repressor p66 alpha gatad2a	-3.51987	0.998478	Q3TIX9	-10	1.360291	Q3V1H1	-10	0.657688	O35218						
-2.9351	1.737663	Q9ES00 Ubiquitin conjugation factor E4 B ube4b	-3.51987	0.998478	Q8BGS2	-10	1.440976	P58389	-10	0.657688	P17665						
-2.74422	1.915478	P08043 Zinc finger protein 2 zfp2	-3.49367	1.015301	Q8R059	-10	1.440976	P59017	-10	0.657688	P25976						
-2.74422	1.915478	Q3UFY7 7-methylguanosine phosphate-specific 5'-nucleotidase nt5c3b	-3.46588	1.228349	P28271	-10	1.440976	P70336	-10	1.150707	B9EJ86						
-2.74422	1.915478	Q91YP0 L-2-hydroxyglutarate dehydrogenase, mitochondrial l2hgdh	-3.46588	1.228349	Q07813	-10	1.440976	P83741	-10	0.657688	O55047						
-2.74422	1.915478	Q9R099 Transducin beta-like protein 2 tbl2	-3.46588	1.228349	Q80YV2	-10	3.386609	Q9QYJ3	-10	0.657688	P49586						
-2.69772	1.598434	O70492 Sorting nexin-3 snx3	-3.46588	1.228349	Q8R404	-10	1.440976	P97315	-10	1.283603	O55023						
-2.51646	1.929825	P13864 DNA (cytosine-5)-methyltransferase 1 dnmt1	-3.46588	1.228349	Q9CYR6	-10	1.440976	P97452	-10	0.657688	O70126						
-2.50704	1.658882	Q61584-7 Fragile X mental retardation syndrome-related protein 1 fxr1	-3.46588	1.228349	Q9EQQ9	-10	1.360291	Q3V4B5	-10	1.053744	Q5SW19						
-2.50441	1.343044	Q9D3B1 Very-long-chain (3R)-3-hydroxyacyl-CoA dehydratase 2 hacd2	-3.40979	1.15335	P61202	-10	1.351823	Q8VHR5	-10	0.907805	Q9WVE8						
-2.49472	1.637083	Q9D892 Inosine triphosphate pyrophosphatase itpa	-3.31347	1.056386	Q80X50	-10	1.440976	Q148V7	-10	0.657688	O89032						
-2.4568	1.808664	P33174 Chromosome-associated kinesin KIF4 kif4	-3.26302	0.77931	Q8R326	-10	1.440976	Q3TCJ1	-10	0.657688	P14576						
-2.42763	2.36952	Q7TSC1 Protein PRRC2A prrc2a	-3.23548	1.060872	O70252	-10	1.440976	Q3U821	-10	0.657688	P97314						
-2.42763	2.36952	Q8R001 Microtubule-associated protein RP/EB family member 2 mapre2	-3.23548	1.060872	Q8VDP3	-10	1.440976	Q3UGS4	-10	0.657688	Q31125						
-2.4257	1.345189	P54729 NEDD8 ultimate buster 1 nub1	-3.23548	1.060872	Q9DBR7	-10	1.440976	Q3UHJ0	-10	1.2929	B1AZI6						
-2.33785	1.712222	Q8BML9 Glutamine--tRNA ligase qars1	-3.22046	1.116482	Q3TLH4	-10	1.469782	P61290	-10	1.150707	O70551						
-2.31644	1.310596	Q9R1P4 Proteasome subunit alpha type-1 psma1	-3.21069	1.167696	Q78ZA7	-10	1.440976	Q3UQ28	-10	0.657688	P26040-DECOY					
-2.24438	1.478516	Q9WUM4 Coronin-1C coro1c	-3.16946	1.174336	Q3UGR5	-10	1.360291	Q7TMF2	-10	0.657688	Q3THG9						
-2.22923	1.317589	Q2NL51 Glycogen synthase kinase-3 alpha gsk3a	-3.16946	1.174336	Q5FWK3	-10	1.360291	Q8BFY6	-10	0.657688	P30282						
-2.2085	1.645405	Q9D1M0 Protein SEC13 homolog sec13	-3.16946	1.174336	Q8C7R4	-10	1.469134	Q91W89	-10	0.657688	P42128						
-2.20015	2.066483	P16254 Signal recognition particle 14 kDa protein srp14	-3.16946	1.174336	Q9QY36	-10	1.440976	Q4JIM5	-10	0.657688	P43024						
-2.12953	1.583444	Q9CY58 Plasminogen activator inhibitor 1 RNA-binding protein serbp1	-3.10663	1.149785	Q8CBY8	-10	1.440976	Q4PJX1	-10	0.657688	P48725-DECOY					
-2.12805	1.92732	O55131 Septin-7 septin7	-3.10427	1.038009	P11103	-10	1.440976	Q4QQM4	-10	0.657688	Q3TIV5						
-2.12543	1.408271	Q00PI9 Heterogeneous nuclear ribonucleoprotein U-like protein 2 hnrnpul2	-3.10027	0.860962	P30285	-10	1.440976	Q5DW34	-10	0.657688	Q3TPE9						
-2.09142	1.76002	Q9JIW9 Ras-related protein Ral-B ralb	-3.10027	0.860962	Q61210	-10	1.360291	Q8BG51	-10	0.657688	Q3U5F4						
-1.99067	1.43112	Q09143 High affinity cationic amino acid transporter 1 slc7a1	-3.04916	0.793437	P81122	-10	1.453241	P35951	-10	0.657688	P61327						
-1.97807	2.52797	Q8CH25 SAFB-like transcription modulator sltm	-3.04916	0.793437	Q9WV92	-10	1.440976	Q5SWP3	-10	0.657688	P62484						
-1.97065	1.446993	Q8BGA9 Mitochondrial inner membrane protein OXA1L oxa1l	-3.03842	1.205567	P46664	-10	1.440976	Q60876	-10	1.053744	Q8C0J2						
-1.97065	1.446993	Q9D8C2 Tetraspanin-13 tspan13	-3.03239	1.151367	Q9R060	-10	1.453241	Q3TX08	-10	1.053744	Q8K268						
-1.95033	1.326992	Q9JMA1 Tetraspanin-13 tspan13	-3.03239	1.151367	Q9WVG6	-10	1.440976	Q61103	-10	1.150707	O70591						
-1.9483	1.715903	P40240 CD9 antigen cd9	-3.02759	0.544411	O88448	-10	1.440976	Q61164	-10	0.657688	Q3UFM5						
-1.94349	1.392411	Q99J36 THUMP domain-containing protein 1 thumpd1	-2.96319	1.130279	Q9CQE8	-10	1.440976	Q61687	-10	1.283603	Q80UW8						
-1.943	2.461405	Q61205 Platelet-activating factor acetylhydrolase IB subunit gamma pafah1b3	-2.96116	0.792341	P47809	-10	1.360291	Q8BHL8	-10	1.150707	P42208						
-1.89947	1.638761	O09005 Sphingolipid delta(4)-desaturase DES1 degs1	-2.96116	0.792341	P57784	-10	1.440976	Q62193	-10	0.657688	P63154						
-1.89947	1.638761	Q9DAA6 Exosome complex component CSL4 exosc1	-2.96116	0.792341	P70677	-10	1.453241	Q6Q477	-10	0.657688	Q3UHU5						
-1.7581	1.394729	Q3TWW8 Serine/arginine-rich splicing factor 6 srsf6	-2.96116	0.792341	P70700	-10	1.401402	P45591	-10	0.657688	P70268						
-1.75566	1.437731	Q61029 Lamina-associated polypeptide 2, isoforms beta/delta/epsilon/gamma tmpo	-2.96116	0.792341	Q6NZC7	-10	1.453241	Q9CRB6	-10	0.657688	Q3UJU9						
-1.64619	1.785819	O55222 Integrin-linked protein kinase ilk	-2.96116	0.792341	Q8BLN5	-10	1.364624	P27546	-10	0.657688	Q3UY96-DECOY					
-1.62844	1.592304	Q80WC7 Arf-GAP domain and FG repeat-containing protein 2 agfg2	-2.96116	0.792341	Q922U1	-10	1.440976	Q62523	-10	0.657688	P70318						
-1.59969	2.703324	Q05D44 Eukaryotic translation initiation factor 5B eif5b	-2.96116	0.792341	Q9QYF9	-10	1.440976	Q64521	-10	0.657688	P70335						
-1.41961	1.615383	Q93092 Transaldolase taldo1	-2.93833	0.793545	Q8BG87-4-DECOY	-10	1.440976	Q69ZS6	-10	1.150707	P55821						
-1.4037	1.314884	Q62432 Mothers against decapentaplegic homolog 2 smad2	-2.88092	1.087083	P54227	-10	1.360291	Q8BJU0	-10	0.657688	P70697						
-1.4005	1.622363	P70288 Histone deacetylase 2 hdac2	-2.88092	1.087083	Q3TYX3	-10	1.360291	Q8C181	-10	1.283603	Q8K212						
-1.34249	1.882526	Q5SSI6 U3 small nucleolar RNA-associated protein 18 homolog utp18	-2.88092	1.087083	Q6P2K6	-10	1.440976	Q6IR34	-10	0.657688	Q3V0Q1-DECOY					
-1.29708	1.342347	Q64737 Trifunctional purine biosynthetic protein adenosine-3 gart	-2.88092	1.087083	Q6P2L6	-10	1.360291	Q8C6M1	-10	0.657688	Q60680						
-1.24987	2.514415	P32921-2 Tryptophan--tRNA ligase, cytoplasmic wars1	-2.88092	1.087083	Q6WKZ8	-10	1.440976	Q6NXJ0	-10	0.657688	P83510						
-1.20637	1.740248	P34884 Macrophage migration inhibitory factor mif	-2.88092	1.087083	Q8BNU0	-10	1.440976	Q6NZN0	-10	0.657688	P97355						
-1.18946	2.81039	Q7TN99 cpeb3	-2.88092	1.087083	Q8K021	-10	1.426794	P39447	-10	0.657688	Q60698						
-1.17018	1.45043	P47955 60S acidic ribosomal protein P1 rplp1	-2.88092	1.087083	Q8R1A4	-10	1.364624	Q3UI43	-10	0.657688	Q61334						
-1.15665	2.284322	P53996-2 Cellular nucleic acid-binding protein cnbp	-2.88092	1.087083	Q921M7	-10	1.360291	Q8CHC4	-10	0.657688	P97789						
-1.15523	1.536235	Q61937 Nucleophosmin npm1	-2.86717	1.090839	P42669	-10	1.453241	Q9JJV2	-10	0.975439	O54988						
-1.11248	1.744083	Q569Z5 Probable ATP-dependent RNA helicase DDX46 ddx46	-2.83906	1.065129	Q64727	-10	1.440976	Q6P8X1	-10	0.657688	Q14BV6						
-1.08425	1.970413	Q9CXW3 Calcyclin-binding protein cacybp	-2.83906	1.065129	Q9D2M8	-10	1.360291	Q8CHW4	-10	0.657688	Q6GU68						
-1.04994	2.613432	P56382 ATP synthase subunit epsilon, mitochondrial atp5f1e	-2.79595	0.888533	O70475	-10	1.440976	Q6PDG5	-10	0.657688	Q2KN98-DECOY					
-1.01238	2.447743	O70251 Elongation factor 1-beta eef1b	-2.79595	0.888533	P46467	-10	1.440976	Q7TPM1	-10	0.657688	Q6P1G0						
-0.89033	1.961397	P61027 Ras-related protein Rab-10 rab10	-2.79595	0.888533	P70429	-10	1.440976	Q80TJ7	-10	0.657688	Q6PGA0						
-0.85557	2.834618	P47962 60S ribosomal protein L5 rpl5	-2.79595	0.888533	Q99K28	-10	5.152007	Q9EST4	-10	1.175008	P63085						
-0.84854	1.542699	O09167 60S ribosomal protein L21 rpl21	-2.77992	1.16085	P70460	-10	1.440976	Q80X82	-10	0.657688	Q6PIP5						
-0.84495	1.791266	Q9Z2W0 Aspartyl aminopeptidase dnpep	-2.71166	0.969716	Q9ESN9-3	-10	1.440976	Q8BTZ7	-10	0.657688	Q3TDD9						
-0.84061	1.528329	P62823 Ras-related protein Rab-3C rab3c	-2.70838	1.03142	Q6PDL0	-10	1.440976	Q8C796	-10	0.657688	Q3TWF6						
-0.83145	1.343816	Q9CX86 Heterogeneous nuclear ribonucleoprotein A0 hnrnpa0	-2.67835	0.893526	Q6A065	-10	1.360291	Q8K202	-10	0.657688	Q6VGS5-DECOY					
-0.82213	2.400152	Q99020 Heterogeneous nuclear ribonucleoprotein A/B hnrnpab	-2.65888	0.737122	P48428	-10	1.440976	Q8CCS6	-10	0.657688	Q6ZQ93-3-DECOY					
-0.81062	2.420725	P57722 Poly(rC)-binding protein 3 pcbp3	-2.65888	0.737122	P56959	-10	1.440976	Q8CE90	-10	0.657688	Q7TNV0						
-0.80929	2.074658	P63276 40S ribosomal protein S17 rps17	-2.65888	0.737122	Q8K1N4	-10	1.471085	P54103	-10	1.283603	Q8K298						
-0.76389	1.708318	Q61553 Fascin fscn1	-2.65888	0.737122	Q8VE47	-10	1.467534	P62627	-10	0.657688	Q3UFY0						
-0.7561	1.611771	Q61990 Poly(rC)-binding protein 2 pcbp2	-2.65053	1.026371	Q5SQM0	-10	1.440976	Q8CJ67	-10	0.657688	Q7TT50						
-0.7489	1.629806	Q921F2 TAR DNA-binding protein 43 tardbp	-2.62214	0.503525	P35546	-10	1.440976	Q8JZM7	-10	0.657688	Q80T79						
-0.74113	1.328329	Q9Z1Q9 Valine--tRNA ligase vars1	-2.62214	0.503525	Q8CDA1	-10	1.440976	Q8K1R7	-10	1.053744	Q8K394						
-0.71935	2.660883	O70194 Eukaryotic translation initiation factor 3 subunit D eif3d	-2.62214	0.503525	Q9Z1M8	-10	1.360291	Q8K2T8	-10	0.657688	Q3UMC0						
-0.71664	2.562617	Q62188 Dihydropyrimidinase-related protein 3 dpysl3	-2.61575	0.893935	Q08093	-10	1.440976	Q8K3I9	-10	0.657688	Q80U87						
-0.61911	1.438205	Q9D6Z1 Nucleolar protein 56 nop56	-2.61454	1.102879	Q8CG76	-10	3.521881	Q80TL7	-10	0.657688	Q3UN04						
-0.5911	2.780818	P97427 Dihydropyrimidinase-related protein 1 crmp1	-2.5932	0.500112	P61028	-10	1.440976	Q8R3V5	-10	0.99214	Q569Z6						
-0.59076	1.372145	Q9CZX8 40S ribosomal protein S19 rps19	-2.57927	1.125089	O35130	-10	1.440976	Q8R3Y5	-10	1.23431	P39749						
-0.58155	1.437082	P61514 60S ribosomal protein L37a rpl37a	-2.57864	0.98501	Q8WTY4	-10	1.360291	Q8R3C6	-10	0.657688	Q3URU2						
-0.56537	2.702759	P52480 Pyruvate kinase PKM pkm	-2.51987	0.802115	O35864	-10	1.440976	Q8VBT9	-10	0.657688	Q80Y44						
-0.5359	1.723274	P68254 14-3-3 protein theta ywhaq	-2.51987	0.802115	Q2TBE6	-10	1.440976	Q8VC30	-10	0.657688	Q3V300						
-0.508	2.090429	P57776-3 Elongation factor 1-delta eef1d	-2.51987	0.802115	Q3TIU4	-10	1.360291	Q8VCE2	-10	0.657688	Q4VBE8						
-0.4532	1.589952	P62631 Elongation factor 1-alpha 2 eef1a2	-2.51987	0.802115	Q5PSV9	-10	1.471085	Q61191	-10	0.657688	Q52KR3						
-0.44435	2.413146	P10126 Elongation factor 1-alpha 1 eef1a1	-2.51987	0.802115	Q8BG15	-10	1.440976	Q8VEE4	-10	0.657688	Q5S003-DECOY					
-0.43725	1.954918	P11983 T-complex protein 1 subunit alpha tcp1	-2.51987	0.802115	Q8CHI8	-10	1.453241	Q9QZQ1	-10	0.657688	Q5SRX1						
-0.41995	1.455216	P11499 Heat shock protein HSP 90-beta hsp90ab1	-2.51987	0.802115	Q99PM9	-10	1.440976	Q91VR8	-10	0.657688	Q61129						
-0.41379	1.66927	P25444 40S ribosomal protein S2 rps2	-2.51987	0.802115	Q9ERF3	-10	1.440976	Q91W50	-10	0.657688	Q80YR5						
-0.38438	1.347922	P47963 60S ribosomal protein L13 rpl13	-2.51987	0.802115	Q9R0L7	-10	1.440976	Q921Y2	-10	0.657688	Q810D6						
-0.37913	1.367838	P16858 Glyceraldehyde-3-phosphate dehydrogenase gapdh	-2.50942	1.125657	Q6ZQ58	-10	1.360291	Q9CQ71	-10	0.99214	Q8BUV3						
-0.33963	1.817836	P63017 Heat shock cognate 71 kDa protein hspa8	-2.50546	1.091068	Q6ZQL4	-10	1.440976	Q922B1	-10	0.657688	Q61180						
-0.28848	1.711523	Q9CWF2 Tubulin beta-2B chain tubb2b	-2.46588	0.94154	Q5F2E7	-10	1.440976	Q922X9	-10	0.657688	Q810J8						
-0.26581	1.650301	P68372 Tubulin beta-4B chain tubb4b	-2.46588	0.94154	Q9D024	-10	1.440976	Q99JW2	-10	0.657688	Q61474						
-0.25148	1.565157	P99024 Tubulin beta-5 chain tubb5	-2.44743	0.949002	Q8CIN4	-10	1.440976	Q99L28	-10	0.657688	Q61792						
0.320503	2.411837	P62082 40S ribosomal protein S7 rps7	-2.41115	1.025371	Q8BWW4	-10	1.360291	Q9CWZ7	-10	1.283603	Q99LI8						
0.434014	1.378531	Q8VEM8 Phosphate carrier protein, mitochondrial slc25a3	-2.40979	0.879352	A2AWA9	-10	1.440976	Q99LB6-2	-10	1.175008	Q7TSV4						
0.478449	2.809075	P20029 Endoplasmic reticulum chaperone BiP hspa5	-2.40979	0.879352	O08915	-10	1.360291	Q9CZH3	-10	0.657688	Q8BH43						
0.496083	1.817496	Q7TPV4 Myb-binding protein 1A mybbp1a	-2.40979	0.879352	O35326	-10	1.440976	Q99NH2	-10	0.657688	Q61884-DECOY					
0.637031	1.335361	Q91VR2 ATP synthase subunit gamma, mitochondrial atp5f1c	-2.40979	0.879352	O35344	-10	1.440976	Q9CQN3	-10	0.657688	Q62441						
0.654575	1.342067	P38647 Stress-70 protein, mitochondrial hspa9	-2.40979	0.879352	Q0VBL3	-10	1.440976	Q9CRA8	-10	0.657688	Q64701						
0.657347	1.378475	O70503 Very-long-chain 3-oxoacyl-CoA reductase hsd17b12	-2.40979	0.879352	Q80W00	-10	1.360291	Q9CZT6	-10	0.975439	P21619						
0.703081	1.96898	P14115 60S ribosomal protein L27a rpl27a	-2.40979	0.879352	Q8R3Y8-2	-10	1.440976	Q9DBL7	-10	0.657688	Q8BMP6						
0.73857	2.588502	Q9Z2I8 Succinate--CoA ligase [GDP-forming] subunit beta, mitochondrial suclg2	-2.40979	0.879352	Q9CQT1	-10	1.426794	Q3B7Z2	-10	0.657688	Q6IE82						
0.845481	2.010051	P63242 Eukaryotic translation initiation factor 5A-1 eif5a	-2.40979	0.879352	Q9DAN9	-10	1.453241	Q9WV55	-10	0.657688	Q6NZF1						
0.851401	1.620293	Q99JI6 Ras-related protein Rap-1b rap1b	-2.40979	0.879352	Q9ERG0	-10	2.852341	Q8C5L7	-10	0.657688	Q6NZJ6-2-DECOY					
0.864108	2.108893	B2RQC6 CAD protein cad	-2.40979	0.879352	Q9WUB4	-10	1.440976	Q9ER73	-10	0.99214	Q9D0L8						
0.901948	2.26283	P54071 Isocitrate dehydrogenase [NADP], mitochondrial idh2	-2.40979	0.879352	Q9WUQ2	-10	1.440976	Q9JK48	-10	0.657688	Q6P8I4						
0.911026	1.461262	Q9R0Q7 Prostaglandin E synthase 3 ptges3	-2.40625	0.685169	Q8BWZ3	-10	1.360291	Q9D071	-10	0.657688	Q8BQM9-DECOY					
0.951675	1.954913	Q9Z110 Delta-1-pyrroline-5-carboxylate synthase aldh18a1	-2.37615	0.975104	Q9JK23	-10	1.440976	Q9R0A5	-10	0.657688	Q6P9P6						
0.994107	2.299287	Q8CIE6 Coatomer subunit alpha copa	-2.36447	0.76805	Q9WU62	-10	1.440976	Q9R0I7	-10	0.657688	Q8BV13						
1.007497	1.339071	Q8QZY1 Eukaryotic translation initiation factor 3 subunit L eif3l	-2.35144	0.657948	Q9CQ48	-10	1.360291	Q9EQ28	-10	0.827353	Q9Z315						
1.021314	1.325934	P00405 Cytochrome c oxidase subunit 2 mtco2	-2.34181	0.867672	Q60972	-10	1.360291	Q9EST3	-10	0.657688	Q6PB93						
1.027769	1.44862	Q99LC3 NADH dehydrogenase [ubiquinone] 1 alpha subcomplex subunit 10, mitochondrial ndufa10	-2.33636	0.78514	O88532	-10	1.440976	Q9R1T4	-10	1.150707	Q0P678						
1.031154	1.343552	Q99KX1 Myeloid leukemia factor 2 mlf2	-2.33315	1.014916	P26516	-10	1.440976	Q9WTU6	-10	0.657688	Q8C156						
1.035767	1.396205	Q6PB66 Leucine-rich PPR motif-containing protein, mitochondrial lrpprc	-2.33141	0.977429	Q3U0V1	-10	3.521881	Q8C166	-10	0.657688	Q6PCP5						
1.121947	2.236055	Q99LC5 Electron transfer flavoprotein subunit alpha, mitochondrial etfa	-2.32243	1.044649	P48722	-10	1.360291	Q9WUL7	-10	0.657688	Q6PHN1-DECOY					
1.164281	1.55028	O35286 Pre-mRNA-splicing factor ATP-dependent RNA helicase DHX15 dhx15	-2.31985	0.464197	Q3UE37	-10	1.426794	Q60676	-10	0.657688	Q6VN19						
1.165221	1.543718	Q921I2 Kelch domain-containing protein 4 klhdc4	-2.29992	1.001692	O08810	-10	1.440976	Q9WU56	-10	0.657688	Q8C1A5						
1.165299	1.370539	P29758 Ornithine aminotransferase, mitochondrial oat	-2.29062	0.459946	P58871	-10	1.453241	Q9Z2Q6	-10	0.657688	Q8C6G8						
1.263772	1.452483	O08756 3-hydroxyacyl-CoA dehydrogenase type-2 hsd17b10	-2.29062	0.459946	Q9ERI5	-10	1.440976	Q9Z268	-10	1.283603	sp|Q6ZPJ3|UBE2O_MOUSE				
1.309325	2.253965	Q9D824 Pre-mRNA 3'-end-processing factor FIP1 fip1l1	-2.25075	0.938663	Q9WU40	-10	1.364624	Q60865	-10	0.657688	Q8CDG3						
1.332393	1.407706	P14685 26S proteasome non-ATPase regulatory subunit 3 psmd3	-2.23548	0.776819	Q8BU03	10	1.303491	Q07456	-10	0.657688	Q791T5						
1.41689	2.843633	P50247 Adenosylhomocysteinase ahcy	-2.23548	0.776819	Q91VK1	10	1.302649	Q9JM99	-10	0.657688	Q7TQ95						
1.508982	1.755431	P62320 Small nuclear ribonucleoprotein Sm D3 snrpd3	-2.23548	0.776819	Q99J47	10	1.478391	P06327	-10	0.657688	Q8JZV7						
1.574285	1.357195	Q8K4L0 ATP-dependent RNA helicase DDX54 ddx54	-2.22394	0.986449	P31230	10	1.512518	Q91WM2	-10	0.657688	Q80UU2						
1.783919	1.32062	P01642 Ig kappa chain V-V region L7	-2.21759	0.70967	Q8BTW3	10	1.401493	Q8CDM1	-10	0.657688	Q80VC9						
1.817186	1.404375	Q8K009 Mitochondrial 10-formyltetrahydrofolate dehydrogenase aldh1l2	-2.21663	0.77366	Q6ZQH8	10	4.25697	Q9CY57-2	-10	0.657688	Q80XQ2						
1.876102	1.579231	Q8VE22 28S ribosomal protein S23, mitochondrial mrps23	-2.21663	0.77366	Q91WG4	10	1.491048	Q8BK62	-10	0.657688	Q8K019						
1.917621	1.786736	Q8R2Q8 Bone marrow stromal antigen 2 bst2	-2.19239	0.679038	Q8VBV7	10	1.593888	Q61425	-10	0.657688	Q8R0S1						
1.948897	2.648042	P47738 Aldehyde dehydrogenase, mitochondrial aldh2	-2.18119	1.263443	Q9CQ65	10	1.740207	Q3TTY5	-10	0.657688	Q80ZV0						
2.012278	2.059945	Q922J9 Fatty acyl-CoA reductase 1 far1	-2.16946	0.819188	P08775	10	2.12	Q9D023	-10	0.657688	Q811D2-DECOY					
2.037903	1.330709	Q8BGH2 Sorting and assembly machinery component 50 homolog samm50	-2.16946	0.819188	Q8BRG8-2	10	1.318236	Q61263	-10	0.657688	Q8BHB4						
2.041012	1.371305	Q8K1Z0 Ubiquinone biosynthesis protein COQ9, mitochondrial coq9	-2.16946	0.819188	Q9Z108	10	1.366815	P12246	-10	0.657688	Q8BLJ3						
2.088182	1.680319	Q9D3D9 ATP synthase subunit delta, mitochondrial atp5f1d	-2.15962	0.986096	O70318	10	1.460692	Q9CQ06	-10	1.150707	Q62241						
2.128532	2.881115	D3Z7P3-2 Glutaminase kidney isoform, mitochondrial gls	-2.14912	0.798969	Q60598	10	1.528792	P46412	-10	0.657688	Q8VE11-2						
2.162762	1.345908	P26262 Plasma kallikrein klkb1	-2.14855	0.968868	Q9CR51	10	1.735839	P11370	-10	0.657688	Q8BLY2						
2.208915	1.780655	Q8K1J6 CCA tRNA nucleotidyltransferase 1, mitochondrial trnt1	-2.11673	0.951782	Q9WTL7	10	2.015143	O08573	-10	0.657688	Q8BMZ5						
2.258358	1.385421	Q80UM7 Mannosyl-oligosaccharide glucosidase mpgs	-2.10751	0.756197	Q3TJZ6	10	1.464862	P10605	-10	0.657688	Q91V61						
2.269414	2.625857	Q9DC61 Mitochondrial-processing peptidase subunit alpha pmpca	-2.10751	0.756197	Q60809	10	1.323987	Q3UMB9	-10	0.657688	Q8BX70						
2.347921	1.474386	O88455 7-dehydrocholesterol reductase dhcr7	-2.10271	1.297838	Q99KP6	10	1.369828	Q8BTV1	-10	0.657688	Q8BXR1						
2.376825	3.112848	Q91VM5 RNA binding motif protein, X-linked-like-1 rbmxl1	-2.10027	0.627513	Q9JJ28	10	1.46259	P01630	-10	0.657688	Q8C0E3						
2.380317	1.355953	Q00623 Apolipoprotein A-I apoa1	-2.09942	0.985458	Q6P1B1	10	1.492282	Q9QZ88	-10	0.657688	Q8C1S0						
2.409746	2.090768	P53994 Ras-related protein Rab-2A rab2a	-2.09464	0.677793	Q91VU7	10	2.429447	Q9WTI7	-10	0.657688	Q8C3F2						
2.541216	2.599621	Q61733 28S ribosomal protein S31, mitochondrial mrps31	-2.07954	0.933668	Q8VDP4	10	2.410984	Q07646	-10	0.657688	Q8C4J0						
2.630876	1.856962	Q8BKZ9 Pyruvate dehydrogenase protein X component, mitochondrial pdhx	-2.07954	0.782576	Q8BW10	10	1.92526	Q8VED5	-10	0.657688	Q91VJ4						
2.72983	2.001336	Q80Y14 Glutaredoxin-related protein 5, mitochondrial glrx5	-2.07644	0.834367	Q3UVL4	10	1.324271	Q80X85	-10	0.657688	Q8C9J3-4						
2.74485	3.500361	D3Z7P3  gls	-2.04765	0.845705	P14733	10	3.481273	Q9DC70	-10	0.657688	Q8CA72						
3.058878	2.097434	Q9D666 SUN domain-containing protein 1 sun1	-2.03717	0.419606	P68037	10	1.450426	Q3TUH1	-10	0.657688	Q8CBE3						
3.133154	1.420567	Q91WS0 CDGSH iron-sulfur domain-containing protein 1 cisd1	-2.03717	0.419606	Q8BTT6	10	1.757471	P14211	-10	0.657688	Q8CCB4						
3.196554	1.460651	A6X935 Inter alpha-trypsin inhibitor, heavy chain 4 itih4	-2.03717	0.419606	Q9DC48	10	1.405651	Q9CXY9	-10	0.657688	Q91VZ6						
3.257466	2.303661	P09103 Protein disulfide-isomerase p4hb	-2.03407	1.222739	O55201	10	1.512713	Q8C8U0	-10	1.175008	Q8R3G1						
3.507645	1.695404	Q91WD5 NADH dehydrogenase [ubiquinone] iron-sulfur protein 2, mitochondrial ndufs2	-2.03079	0.686539	Q8R3N1	10	1.460692	Q9JKC8	-10	0.657688	Q8CCJ3						
3.547543	2.378748	P99027 60S acidic ribosomal protein P2 rplp2	-2.0274	0.891503	Q8BY87	10	1.682358	P42225	-10	0.657688	Q8CFC2						
3.710334	1.337972	P42227 Signal transducer and activator of transcription 3 stat3	-2.02166	0.651072	Q99JY4	10	1.464862	P63158	-10	0.657688	Q8CI75-2						
			-2.01753	0.6056	Q64105	10	1.464862	Q9QZH6	-10	0.657688	Q8K1K9						
			-2.01041	0.902286	Q8VHK9				-10	0.657688	Q91YL2						
			-1.99476	0.70646	Q8R4U7				-10	0.657688	Q8K2L8						
			-1.98834	0.411109	Q8BJ05				-10	0.657688	Q8QZV7						
			-1.98829	1.213444	Q7TMY8				-10	0.657688	Q920F6						
			-1.97623	0.803504	Q08943				-10	0.657688	Q8R080						
			-1.97159	0.884257	B2RX14				-10	0.99214	Q9D1G2						
			-1.96348	1.111526	Q8VDJ3				-10	0.657688	Q925H1						
			-1.96116	0.558197	P36916				-10	1.150707	Q76KJ5						
			-1.96116	0.558197	Q3UGC7				-10	0.657688	Q8R0A0-DECOY					
			-1.96116	0.558197	Q8R5H1-2			-10	0.657688	Q99JF5						
			-1.94979	1.035277	P62984				-10	0.657688	Q8R0F6						
			-1.94181	0.96081	Q9QUR7				-10	0.657688	Q8R2M2						
			-1.93833	0.553855	Q9CYL5				-10	0.657688	Q8R4X3						
			-1.92996	0.840039	Q9Z0X1				-10	0.657688	Q8VDM6						
			-1.91811	0.853706	Q9CPQ3				-10	0.657688	Q91VH6						
			-1.91435	0.649306	Q8BIG7				-10	0.657688	Q99LS3						
			-1.89189	0.393647	Q3TFK5				-10	0.657688	Q91W43						
			-1.88092	0.677366	B2RXC1				-10	1.150707	Q8C079						
			-1.88092	0.677366	O55126				-10	1.175008	Q9JHK4						
			-1.88092	0.677366	O88746				-10	0.657688	Q91W59						
			-1.88092	0.677366	P57759				-10	0.657688	Q9CR16						
			-1.88092	0.677366	P59764				-10	0.657688	Q91WE2						
			-1.88092	0.677366	P97433-2-DECOY			-10	1.053744	Q91WJ8						
			-1.88092	0.677366	Q3UKJ7				-10	0.657688	Q9CWL8						
			-1.88092	0.677366	Q60575-3				-10	0.657688	Q9D1E6						
			-1.88092	0.677366	Q60722				-10	0.657688	Q91WU5						
			-1.88092	0.677366	Q6A4J8				-10	1.2929	Q3UUY6						
			-1.88092	0.677366	Q80TP3				-10	1.150707	Q8K1E6						
			-1.88092	0.677366	Q80TV8				-10	1.150707	Q8K4Z5						
			-1.88092	0.677366	Q8BSZ2				-10	0.657688	Q91YD9						
			-1.88092	0.677366	Q8CIM8				-10	1.150707	Q9CXR1						
			-1.88092	0.677366	Q8K1E0				-10	0.657688	Q9D8S9						
			-1.88092	0.677366	Q8K2V1				-10	0.657688	Q91ZU6						
			-1.88092	0.677366	Q8R3C0				-10	0.657688	Q91ZV0						
			-1.88092	0.677366	Q91ZJ5				-10	0.657688	Q99JR8						
			-1.88092	0.677366	Q99J10				-10	0.657688	Q99JX7						
			-1.88092	0.677366	Q99MK8				-10	0.99214	Q9QY76						
			-1.88092	0.677366	Q9CQQ4				-10	0.657688	Q9DAW6						
			-1.88092	0.677366	Q9CXK9				-10	0.657688	Q9DB05						
			-1.88092	0.677366	Q9D787				-10	0.657688	Q9DB43						
			-1.88092	0.677366	Q9JJT0				-10	0.657688	Q9CPT5						
			-1.88092	0.677366	Q9QYR9				-10	0.657688	Q9ESU6						
			-1.88092	0.677366	Q9Z0G0				-10	0.657688	Q9ESZ8						
			-1.87245	1.098052	P37913				-10	0.657688	Q9QWY8						
			-1.86111	0.920184	P52479				-10	0.657688	Q9CQA9						
			-1.8593	0.779673	Q9DCC4				-10	0.657688	Q9CXD6						
			-1.83988	0.698766	Q8R1Q8				-10	0.657688	Q9D6T0						
			-1.83468	1.16153	Q6PDQ2				-10	1.2929	Q80VL1						
			-1.82483	0.628926	Q6PA06				-10	0.657688	Q9DBC3						
			-1.82467	0.815984	Q61081				-10	0.657688	Q9DBT5						
			-1.80796	0.923425	Q9DCL9				-10	0.657688	Q9QXN0						
			-1.80228	0.800937	O08759				-10	0.657688	Q9DC29						
			-1.78307	0.540852	P52651				-10	1.2929	Q9D706						
			-1.78179	0.743529	Q01405				-10	0.657688	Q9QXZ0						
			-1.77884	0.985812	O35691				-10	0.657688	Q9QYE6						
			-1.7738	0.767858	P56399				-10	0.975439	P53569						
			-1.77297	0.780736	Q80U78				-10	0.657688	Q9EQF6						
			-1.76269	1.240194	Q9CYN2				-10	0.657688	Q9R0A0						
			-1.76237	0.544107	Q8VBW6				-10	0.856742	P56873						
			-1.75738	0.70413	Q80U93				-10	0.657688	Q9ERD6						
			-1.75011	0.923382	Q6P542				-10	0.657688	Q8BP71						
			-1.74422	0.835924	P58501				-10	0.657688	Q9JI99						
			-1.74422	0.835924	Q8BHC1				-10	1.150707	Q9D1C1						
			-1.74313	0.962961	Q99K85				-10	0.657688	Q9WTX5						
			-1.73489	0.363348	Q62426				-10	0.657688	Q9JJF3						
			-1.73317	1.028239	Q9CU62				-10	0.657688	Q9WTX8						
			-1.72344	1.039862	Q61074				-10	0.657688	Q9JJK2						
			-1.72187	0.766855	Q3THK3				-10	0.657688	Q9QWV9						
			-1.72187	0.766855	Q80VJ3				-10	0.657688	Q9QYB2						
			-1.72187	0.766855	Q8BQM4				-10	0.657688	Q9WV30						
			-1.72187	0.766855	Q99LP6				-10	0.657688	Q9WUM3						
			-1.71424	0.62526	Q3UJB9				-10	0.657688	Q9WVQ0						
			-1.70848	0.57485	Q9D1A2				-10	0.657688	Q9Z1K5						
			-1.70566	0.357463	P42125				-10	0.657688	Q9Z1Z2						
			-1.70566	0.357463	P43247				10	1.169892	Q91X78						
			-1.70566	0.357463	P85094				10	0.559385	Q62178						
			-1.70566	0.357463	Q64520				10	0.317297	A2AAE1-6-DECOY					
			-1.70566	0.357463	Q6ZWR6				10	0.537503	Q8R035						
			-1.70566	0.357463	Q8K3X4				10	0.317297	A2AIL4						
			-1.70566	0.357463	Q99J62				10	0.317297	A2AVR2						
			-1.70566	0.357463	Q9DBL1				10	0.888525	Q8JZK9						
			-1.69517	0.845302	Q8C1B7				10	0.317297	A3KGS3-2-DECOY					
			-1.68889	1.17305	P62858				10	0.317297	B1AQJ2						
			-1.68656	1.289886	Q80UG5				10	0.773735	Q8R3F5						
			-1.68505	0.848569	P07742				10	0.317297	B1AR13						
			-1.67236	0.573154	O88487				10	0.317297	A2AIV2-DECOY					
			-1.67236	0.573154	Q61464				10	0.317297	E9Q4Z2						
			-1.6715	0.722463	P50396				10	1.154292	P49025-5						
			-1.6645	0.576244	Q99K01-2				10	0.56793	O08848						
			-1.65919	1.269342	P19157				10	0.317297	B2RRF6						
			-1.65053	0.568941	Q9WVR4				10	0.317297	E9Q5K9						
			-1.64995	0.971936	Q61768				10	0.568074	O09172						
			-1.64116	0.686821	Q6Y7W8				10	0.317297	O35654						
			-1.6369	0.620126	A2ADY9				10	0.317297	O55091						
			-1.6369	0.620126	Q80SW1				10	0.317297	O88700						
			-1.62004	1.19816	P84096				10	0.56793	O35454						
			-1.61765	1.2758	Q3TKT4-2				10	0.573817	C0HKD1						
			-1.60057	0.899228	Q9R0L6				10	0.543679	O55022						
			-1.6	0.713204	Q8CIG8				10	0.317297	O88845						
			-1.59806	0.98144	O70378				10	0.317297	P06728						
			-1.59729	0.619137	P26039				10	0.317297	P0C5E4-DECOY					
			-1.59481	1.291853	Q9Z0H1				10	0.770305	P99029						
			-1.5932	0.334162	P11214				10	0.435792	P13020						
			-1.5932	0.334162	Q8BNJ2				10	0.317297	P12804						
			-1.58634	0.504878	Q91VX2				10	0.317297	P23506						
			-1.58518	1.143248	O35231				10	0.317297	P26041-DECOY					
			-1.58518	1.143248	Q80Y81				10	0.317297	P26350						
			-1.58518	1.143248	Q9QYI4				10	0.317297	P28867-2						
			-1.58449	0.533621	Q8K354				10	0.317297	P40630-2						
			-1.58449	0.533621	Q9Z2M7				10	0.926273	P61982						
			-1.58279	0.670036	O54984				10	0.317297	P50096						
			-1.58279	0.670036	P13439				10	0.317297	P52332-DECOY					
			-1.58279	0.670036	P35235				10	0.317297	P58064						
			-1.58036	0.606153	Q99LD4				10	0.317297	P59016						
			-1.57864	0.528667	P97760				10	0.799369	Q6P2B1						
			-1.57864	0.528667	Q8C4Y3-3				10	0.317297	P70255						
			-1.57864	0.528667	Q9CQ80				10	0.317297	Q0PMG2						
			-1.57853	0.638632	Q9Z1X4				10	0.317297	P06684						
			-1.57154	0.98813	Q9Z1F9				10	0.574169	E9PZQ0-DECOY					
			-1.55591	1.130557	Q9CQM9				10	0.718511	Q01339						
			-1.54918	0.76942	Q01853				10	0.685653	Q9CY73						
			-1.54276	0.533894	Q9D1P4				10	0.550924	Q640N1						
			-1.54224	0.925126	O88342				10	0.317297	Q3TLH4-5-DECOY					
			-1.53781	0.693261	Q91VI7				10	0.543397	A6BLY7						
			-1.53518	0.584743	Q9JKP5				10	0.73824	Q8VI94						
			-1.52799	0.798169	Q11011				10	0.711944	Q60766-2						
			-1.52799	0.798169	Q61655				10	0.568074	Q7TQK1						
			-1.52025	1.198305	Q8VDF2				10	0.612793	P24547						
			-1.51987	0.448811	P20664				10	0.543679	Q3UW53						
			-1.51987	0.448811	Q8R2N2				10	0.317297	Q3TUU5						
			-1.51987	0.448811	Q8VE99				10	0.317297	Q3TXS7						
			-1.51987	0.448811	Q9CZ91				10	0.317297	Q3U1G5						
			-1.51987	0.448811	Q9D0R4				10	0.48982	P21278						
			-1.51948	0.740716	Q91VR5				10	0.317297	Q3UH68-DECOY					
			-1.51783	1.153799	Q62189				10	0.573817	Q01768						
			-1.51532	0.473774	Q8R0K4				10	0.56793	P10833						
			-1.5153	0.426451	P27612				10	0.543679	Q810S1						
			-1.5153	0.426451	Q8CCF0				10	0.317297	Q3ULD5						
			-1.50554	0.751346	O88844				10	0.574169	P01821						
			-1.50065	0.789706	Q8BH58				10	0.317297	P39039						
			-1.49938	0.639446	Q07417				10	0.317297	A2AG58						
			-1.49622	0.626215	P22892				10	0.782806	P01845						
			-1.49563	0.9605	Q9DCT8				10	0.317297	Q3UTQ8						
			-1.4945	0.955409	Q9WTM5				10	0.317297	Q3UV70						
			-1.49368	0.745329	O35134				10	0.317297	Q3UYI5						
			-1.48806	0.63945	Q64213				10	0.559385	Q6ZPZ3						
			-1.48651	0.9975	Q4FZF3				10	0.317297	Q3V0F0						
			-1.47998	0.801512	Q9QYH6				10	0.317297	Q505D7						
			-1.47654	0.637257	Q99NH0				10	0.317297	Q56A06						
			-1.46172	1.171472	O08709				10	1.107063	P07309						
			-1.4549	0.592364	Q80X90				10	0.317297	Q59J78						
			-1.45301	0.619215	Q9R1J0				10	0.317297	Q5DTT1						
			-1.44839	0.575787	Q8BGA5				10	0.317297	Q5HZG4-DECOY					
			-1.43678	0.876746	Q3UA37				10	0.543679	Q8R3Q0						
			-1.43057	1.069865	Q62318				10	0.56793	Q3UFY8						
			-1.4275	0.48246	Q9QXS6				10	0.317297	Q5M8N4						
			-1.42087	0.754342	Q64378				10	0.317297	Q5SYL3-DECOY					
			-1.40979	0.437181	Q5XFZ0				10	0.56037	P59708						
			-1.40979	0.437181	Q99JW4				10	0.47441	Q3TBW2						
			-1.40979	0.437181	Q99LD8				10	0.886954	P61620						
			-1.40735	0.481325	Q8BRF7				10	0.895492	Q923G2						
			-1.40338	0.292757	O35685				10	0.559385	Q8BX10						
			-1.40338	0.292757	Q80U58				10	0.935503	O88477						
			-1.40338	0.292757	Q8BWM0				10	0.888553	Q91VM9						
			-1.40309	0.518757	Q9D4J7				10	0.554795	Q8BMD8						
			-1.39086	0.52384	Q61686				10	0.317297	Q62209						
			-1.38265	0.727997	Q8VCM8				10	0.874229	Q9WUR9						
			-1.37611	0.556961	Q69ZS7				10	0.317297	Q61493						
			-1.37561	0.792557	Q8CG47				10	0.317297	Q61595-5-DECOY					
			-1.37248	1.266609	Q8BI72				10	1.228517	Q3U186						
			-1.36628	1.145815	Q64332				10	0.91238	Q70KF4						
			-1.36215	0.395524	Q80TM9				10	0.317297	Q62018						
			-1.36123	1.207146	Q5SUR0				10	0.56793	Q64282						
			-1.36082	0.501326	Q9WVM3				10	0.51621	Q91VA6						
			-1.35757	0.719509	Q8C854				10	0.875235	Q99N94						
			-1.35164	0.537638	Q9QWT9				10	0.317297	Q62036						
			-1.35136	0.84527	Q9R0U0-3			10	0.56037	Q8R5K4						
			-1.34145	1.10844	P46660				10	0.317297	Q62187						
			-1.33315	0.566554	Q3UHD6				10	0.317297	Q64285						
			-1.33123	1.275083	P62137				10	0.317297	Q62504						
			-1.32993	0.622683	Q7TQH0				10	0.317297	Q640L5-DECOY					
			-1.32362	0.842407	Q9CZ13				10	0.82058	P49962						
			-1.32341	0.560994	P10639				10	0.317297	Q64152-2						
			-1.32341	0.560994	P59325				10	0.543397	O08688						
			-1.31979	0.316272	Q4V9W2				10	0.7544	Q8C4Q6						
			-1.3182	1.014261	P62814				10	0.317297	Q6A028						
			-1.3112	1.160874	P17918				10	0.317297	Q6DFV1						
			-1.31109	0.561312	Q8BP48				10	0.317297	Q6P4S8						
			-1.3064	0.955969	Q6NS46				10	0.46305	P19221						
			-1.29937	0.622519	Q9CW03				10	0.563453	P48771						
			-1.2926	0.856501	Q5XJY4				10	0.56793	Q78JN3						
			-1.29092	0.26728	E9Q634-DECOY			10	0.317297	Q6PD26						
			-1.29092	0.26728	Q5HZI9-DECOY			10	1.194775	Q61704						
			-1.29062	0.267212	P28659				10	0.317297	Q6Q899						
			-1.29062	0.267212	P37889				10	0.887886	P19536						
			-1.29062	0.267212	Q6PDM2				10	0.47441	Q9JJN5						
			-1.29062	0.267212	Q8BU14				10	0.550924	Q8VHZ7						
			-1.29062	0.267212	Q8CCP0				10	0.317297	O70325						
			-1.27967	0.264704	Q80XI3-2-DECOY			10	0.317297	Q6ZWQ0-DECOY					
			-1.2779	0.544783	P63011				10	0.875182	A2A6A1						
			-1.27355	0.50154	G5E870				10	0.317297	Q70FJ1						
			-1.27293	0.86069	Q3TEA8				10	0.317297	Q80SY4						
			-1.27132	0.746178	Q99N92				10	0.480974	Q3U7R1						
			-1.26994	0.955998	Q9R0P3				10	0.317297	Q80U72						
			-1.26798	0.584977	Q63844				10	0.317297	Q80UZ2						
			-1.26288	0.260849	Q8CGY8				10	0.317297	Q80VR2-DECOY					
			-1.25075	0.50809	P35585				10	0.56793	Q8BHY2						
			-1.25075	0.50809	Q8BGS0				10	0.79585	Q9CPP6						
			-1.25075	0.50809	Q9CWS0				10	0.454654	Q9DCT5						
			-1.24585	0.724649	Q9CQS8				10	0.317297	Q8BGC4						
			-1.24211	0.655793	Q9D1G1				10	0.90903	Q9CQR4						
			-1.23034	0.60397	Q9DBR0				10	0.911252	Q9D338						
			-1.23	0.855748	Q9CQ60				10	0.559385	Q8R5J9						
			-1.21759	0.331611	Q8C570				10	0.317297	Q8BI84						
			-1.21663	0.346475	Q91Z49				10	0.677338	Q8BU88						
			-1.20657	0.93255	Q6NVF9				10	1.21765	O89079						
			-1.2005	0.573965	Q80TB8				10	0.317297	Q8BKX6						
			-1.1999	0.482393	Q9EPU0-2			10	0.317297	Q8BNA6						
			-1.19375	0.630842	Q61171				10	0.491627	P46935						
			-1.19045	1.164248	P06837				10	0.538887	P54276-DECOY					
			-1.188	0.482774	Q8C878				10	0.317297	Q64GA5-DECOY					
			-1.188	0.482774	Q9ERA6				10	0.876881	Q9CQW2						
			-1.18357	0.422942	Q8CEL2-DECOY			10	0.317297	Q80WQ2						
			-1.17961	0.343749	Q8VCT3				10	0.543397	O35638						
			-1.17961	0.343749	Q9CT10				10	0.842032	Q9EQU5						
			-1.17961	0.343749	Q9Z0W3				10	0.54674	Q5U3K5						
			-1.17292	0.906034	Q6PE01				10	0.543679	Q99K23						
			-1.17218	0.455052	A2AGT5				10	0.317297	Q8BRH0						
			-1.16102	1.170284	E9PVX6				10	0.317297	Q8BUV6-DECOY					
			-1.15926	0.39627	Q9JKF7				10	0.570666	Q9DCM0						
			-1.15201	0.443667	Q99KK2				10	0.317297	Q8BWF0						
			-1.14976	0.696149	P50431				10	0.543397	Q8BXA1						
			-1.14751	0.484657	Q3UPF5				10	0.317297	Q8BYC6						
			-1.14728	0.653372	Q9Z0S1				10	0.563453	Q9CQJ8						
			-1.14467	1.005936	Q02053				10	0.317297	Q8BZ20						
			-1.14288	0.634983	O55029				10	0.317297	Q8BZN6-2					
			-1.14232	0.547446	O55128				10	0.574169	Q8CE72-DECOY					
			-1.13996	0.599884	Q9JK92				10	1.208015	Q9ET01						
			-1.13695	0.691526	Q9D172				10	0.568074	Q99LB7						
			-1.13315	0.901229	Q99JR1				10	0.872297	P55096						
			-1.13202	0.445785	Q07076				10	0.799369	Q8C7X2						
			-1.13158	0.273516	Q9JM52-3			10	0.317297	Q8C3I8						
			-1.12701	0.396319	P24527				10	0.543679	Q9JIH2						
			-1.12595	0.453796	O35621				10	0.317297	Q8C3Q9						
			-1.12342	0.319942	P97801				10	0.317297	Q8CB62-DECOY					
			-1.12342	0.319942	Q8VEB1				10	0.317297	Q8CFW7						
			-1.11742	0.946298	Q8VE97				10	0.555899	Q8C2E7						
			-1.11279	0.780036	Q8BJW6				10	0.51621	Q9WUU7						
			-1.10751	0.307915	Q61206				10	0.729428	O35709						
			-1.10751	0.307915	Q9JLN9				10	0.317297	Q8CG46						
			-1.10275	0.449777	Q6PAC3				10	0.607735	P70670						
			-1.10027	0.278938	Q2YDW2				10	0.317297	Q8CIG0						
			-1.097	0.92301	Q64514-2				10	0.811203	O88665						
			-1.09196	0.380193	Q06185				10	0.563453	Q9Z1P8-DECOY					
			-1.08999	0.418387	Q80XI3				10	0.538887	Q62388						
			-1.08949	0.508953	Q91YK2				10	0.317297	Q7TN58						
			-1.08787	0.300076	Q5U4C1				10	0.317297	Q8CJF7						
			-1.08632	0.935325	O35295				10	0.555899	Q8R1S0						
			-1.08632	0.935325	O35343				10	0.538887	Q7TN37-DECOY					
			-1.07906	0.922776	Q9D1N9				10	0.317297	Q8JZM0						
			-1.07816	0.495426	Q60875				10	0.48982	Q9CXI0						
			-1.06933	0.379397	Q9D2R0				10	0.799369	Q99KK9						
			-1.06818	0.594353	Q8BVQ5				10	0.317297	Q8JZY4						
			-1.06811	0.992951	Q9DCX2				10	0.491627	Q99PT1						
			-1.0651	0.72689	Q922S8				10	0.317297	Q8VDT9						
			-1.06473	0.466574	Q9JLV1				10	0.317297	Q8K012						
			-1.05759	0.400517	Q62383				10	0.573968	Q8BLY1						
			-1.04795	0.753403	O08749				10	0.317297	Q8K0V4						
			-1.04665	0.928102	P68433				10	0.533028	Q922P9						
			-1.04161	0.505404	Q99KK7				10	0.56793	Q8CFD4						
			-1.04153	0.631627	Q9DAW9				10	0.317297	Q8K2V6						
			-1.0403	0.404265	Q8R574				10	0.317297	Q8R151-DECOY					
			-1.03717	0.208511	O70146-DECOY			10	0.317297	Q8R4V4						
			-1.03717	0.208511	P17809				10	0.317297	Q8R5F7						
			-1.03717	0.208511	P21107-2				10	0.935503	Q9JIY5						
			-1.03717	0.208511	P43883				10	0.543397	Q9CR88						
			-1.03717	0.208511	P97298				10	0.317297	Q8VD46						
			-1.03717	0.208511	P97329				10	0.538887	Q7TT37						
			-1.03717	0.208511	Q60520				10	0.317297	Q91VE0						
			-1.03717	0.208511	Q61187				10	0.758785	Q99KE1						
			-1.03717	0.208511	Q68FF6				10	0.317297	Q91YM4						
			-1.03717	0.208511	Q6A0A2				10	0.317297	Q91YP2						
			-1.03717	0.208511	Q80UY2				10	0.317297	Q91YW3						
			-1.03717	0.208511	Q8R2Q4				10	0.543397	Q9CY64						
			-1.03717	0.208511	Q8R3L2-4				10	0.79585	Q9CR47						
			-1.03717	0.208511	Q8R418				10	0.800312	Q9D1P0						
			-1.03717	0.208511	Q9CSU0-2			10	0.317297	Q91ZA3						
			-1.03717	0.208511	Q9CU65				10	0.317297	Q91ZD6-DECOY					
			-1.03717	0.208511	Q9EPV8				10	1.123877	P62317						
			-1.03717	0.208511	Q9EPZ6-DECOY			10	0.51105	Q8R0F5						
			-1.03717	0.208511	Q9EQW7				10	0.887886	Q6PIX9-2						
			-1.03178	0.719529	Q60668				10	0.317297	Q920A5						
			-1.0295	0.283005	Q9EPE9				10	0.317297	Q921H8						
			-1.02232	0.830623	Q00612				10	0.317297	Q921I0						
			-1.02154	0.613185	Q9Z130				10	0.317297	Q7TSQ8						
			-1.01694	0.383049	O89017				10	0.317297	Q921M4-2-DECOY					
			-1.01274	0.24263	P47753				10	0.317297	Q8JZZ7-DECOY					
			-1.01274	0.24263	Q810B6				10	0.317297	Q922Q1						
			-1.01025	0.866845	Q6KCD5				10	0.785325	P05366						
			-1.00909	0.342146	Q9ES97				10	1.189784	Q6GQT9						
			-1.00845	0.990731	Q9Z127				10	0.90584	Q9Z1J3						
			-1.0044	0.473841	Q8C052				10	0.475911	Q61818-2-DECOY					
			-0.99594	0.491518	Q80UM3				10	0.317297	Q924W5						
			-0.99415	0.904927	Q7TMM9				10	0.317297	Q99J25						
			-0.99326	0.874187	Q8K2K6				10	0.317297	Q99MR8						
			-0.98483	0.438243	Q7M6Y3-5			10	0.56037	Q9D1H8						
			-0.98187	0.381755	Q99P72				10	0.317297	Q99M04						
			-0.9817	0.358025	P63024				10	0.317297	Q99N93						
			-0.97984	0.997083	P50516				10	0.317297	Q99N95						
			-0.9768	0.412637	O88544				10	0.317297	Q99PG2						
			-0.96461	0.67544	Q8R010				10	0.317297	Q9CPY1						
			-0.95987	1.257308	Q921N6				10	0.317297	Q9CPN8						
			-0.95664	0.395906	O54825				10	0.317297	Q9CQF0						
			-0.9538	0.584014	P53395				10	0.317297	Q9CWW6						
			-0.94862	0.569938	Q9Z103				10	0.543679	Q9QZ23						
			-0.94853	0.418687	P60122				10	0.538887	Q9Z2E2-5-DECOY					
			-0.94082	0.94391	P28656				10	0.550924	Q9D8K8						
			-0.93997	0.999286	Q8K310				10	0.752627	Q08879						
			-0.93602	0.674605	Q8K3J1				10	0.779404	Q8BWY9						
			-0.93589	0.581254	Q91YI0				10	0.317297	Q9D0M5						
			-0.92361	1.092455	P17225				10	0.543397	Q9D937						
			-0.91211	0.317127	Q99L48				10	1.004584	P32020						
			-0.90872	0.431685	Q9Z1P6				10	0.543679	Q9WTP7						
			-0.90496	1.149305	Q9CZY3				10	0.317297	Q99K43						
			-0.90085	0.934298	Q9DBG7				10	0.884341	Q9D6J6						
			-0.90025	0.49658	P46061				10	0.317297	Q9D0S9						
			-0.89901	0.321405	Q571I9				10	0.317297	Q9D1E8						
			-0.89718	0.429525	Q80ZS3				10	0.505801	Q924L1						
			-0.89668	0.544522	Q3UDE2				10	0.729428	P61804						
			-0.89487	0.439156	Q61033				10	1.023151	P57787						
			-0.88956	0.609154	Q6PGB6				10	0.559385	Q9CQF3						
			-0.88504	0.549382	P62192				10	0.317297	Q9D2G5-6					
			-0.88096	0.361079	Q99MR6				10	0.317297	Q9D7N6						
			-0.88092	0.22555	P38060				10	0.317297	Q9D906						
			-0.88092	0.22555	Q8BNV1				10	0.317297	Q9D952-DECOY					
			-0.88092	0.22555	Q8BYK6				10	0.573817	Q5NC83-DECOY					
			-0.88092	0.22555	Q8VCY6				10	0.317297	Q9DB25						
			-0.88092	0.22555	Q9QXV1				10	0.317297	Q9DCB1						
			-0.88092	0.22555	Q9Z2V5				10	0.317297	Q9WVP6-DECOY					
			-0.87813	0.211979	Q61735				10	0.317297	Q9WTP6						
			-0.87756	1.091255	Q8BK67				10	0.317297	Q9ESW4						
			-0.87526	1.08732	Q62418-2				10	0.317297	Q9JHI5						
			-0.86973	0.414798	P52432				10	0.317297	Q9JJG9						
			-0.86763	0.387674	Q8QZS1				10	0.799369	Q9CQN7						
			-0.86482	1.098969	P09411				10	1.036453	Q6URW6						
			-0.86405	0.754534	P84244				10	0.317297	Q9QXN3						
			-0.86272	0.205184	Q9JII5				10	0.872297	Q80SY5						
			-0.85229	0.310155	O88796				10	0.875182	O70166						
			-0.84753	0.214068	Q9DCB8				10	0.317297	Q9R049-2						
			-0.84559	0.696443	Q3THE2				10	0.766972	Q921S7						
			-0.83868	0.388886	P05202				10	0.317297	Q9WU42						
			-0.83788	0.434339	Q3THK7				10	1.24284	Q99L13						
			-0.82491	0.219136	Q91VT4				10	0.317297	Q9WVF7						
			-0.82483	0.203204	Q8K2Q7				10	0.56793	Q8K2Z4						
			-0.82359	0.78735	Q8VIJ6												
			-0.8218	1.122227	Q80WJ7												
			-0.82056	0.67296	P59999												
			-0.81891	0.158279	Q6ZWQ0												
			-0.81891	0.158279	Q9Z1R3												
			-0.81581	1.184768	Q99LF4												
			-0.81256	0.445299	Q68FL6												
			-0.81018	1.271579	P35282												
			-0.803	0.359192	Q6A0A9												
			-0.79595	0.186962	Q80VD1												
			-0.78785	0.634155	O08553												
			-0.78565	0.324509	Q8BHD7-2											
			-0.77926	0.633417	P84228												
			-0.77626	0.202565	L0N7N1												
			-0.77544	0.663484	Q6NZJ6												
			-0.771	1.044543	Q9CR57												
			-0.77062	1.106298	P70349												
			-0.76965	0.712073	Q5XJY5												
			-0.76476	0.518084	Q99MT2												
			-0.7638	0.492021	Q921I9												
			-0.76232	0.203549	Q08288												
			-0.76002	0.542667	Q8BGQ7												
			-0.75546	0.438795	Q9D823												
			-0.7505	0.44714	A2AN08												
			-0.7481	0.400384	G5E829												
			-0.74788	0.45777	Q6P5E4												
			-0.74448	0.265843	Q91YT7												
			-0.74422	0.196972	Q91XD7												
			-0.73954	0.37787	P54775												
			-0.73775	0.232747	Q8CFI7												
			-0.73625	0.262455	Q9D5T0												
			-0.73489	0.139438	P18572												
			-0.73489	0.139438	P30681												
			-0.73489	0.139438	P60487												
			-0.73422	0.34111	Q6PGH1												
			-0.72607	0.264031	P56379												
			-0.72187	0.25907	P31938												
			-0.72187	0.25907	Q63850												
			-0.72187	0.25907	Q8BJL0												
			-0.72187	0.25907	Q91V01												
			-0.72187	0.25907	Q9D0D5												
			-0.72187	0.25907	Q9D2Z4												
			-0.72187	0.25907	Q9D7X3												
			-0.71978	0.261576	Q8C5L3												
			-0.71815	0.401835	Q8BGT5												
			-0.71716	0.735432	Q9WTX6												
			-0.71688	0.244439	P35601												
			-0.71688	0.244439	Q8C4J7												
			-0.71688	0.244439	Q99P31												
			-0.71538	0.229242	Q9CWX9												
			-0.71174	0.746123	Q8R1B4												
			-0.70566	0.132978	A2APV2												
			-0.70566	0.132978	O35387												
			-0.70566	0.132978	O88967												
			-0.70566	0.132978	P35123												
			-0.70566	0.132978	P53995												
			-0.70566	0.132978	P56383												
			-0.70566	0.132978	P70303												
			-0.70566	0.132978	P70335-2-DECOY											
			-0.70566	0.132978	P97494												
			-0.70566	0.132978	P97929												
			-0.70566	0.132978	Q0VGY8												
			-0.70566	0.132978	Q3TIR3												
			-0.70566	0.132978	Q64127												
			-0.70566	0.132978	Q6PG16												
			-0.70566	0.132978	Q80U16-DECOY											
			-0.70566	0.132978	Q8BJW5												
			-0.70566	0.132978	Q8K3A9												
			-0.70566	0.132978	Q8VCG3												
			-0.70566	0.132978	Q8VD75-DECOY											
			-0.70566	0.132978	Q91XD6												
			-0.70566	0.132978	Q99MN9												
			-0.70566	0.132978	Q99N96												
			-0.70566	0.132978	Q9CQT2												
			-0.70566	0.132978	Q9D7B6												
			-0.70566	0.132978	Q9DB96												
			-0.70566	0.132978	Q9JMH9												
			-0.70566	0.132978	Q9Z2H5												
			-0.70566	0.132978	Q8CGC6												
			-0.70009	0.294996	Q9Z1D1												
			-0.6985	0.861066	P35980												
			-0.68543	1.093459	P30416												
			-0.68415	0.852353	Q9D8N0												
			-0.6839	0.689434	Q99MN1												
			-0.68223	1.024135	P47915												
			-0.68211	0.291994	Q8BHN3-2											
			-0.68063	0.793029	P60766												
			-0.6805	0.634225	Q8C2Q3												
			-0.67974	0.368539	P32233												
			-0.67957	0.476674	Q9R0Y5												
			-0.67902	0.438318	Q99N87												
			-0.67422	1.136137	P18155												
			-0.67388	0.178813	P57746												
			-0.67154	0.813972	P17751												
			-0.66945	0.519784	P14824												
			-0.6674	0.236321	Q9JJZ2												
			-0.66714	0.384728	Q8VDN2												
			-0.66261	0.555804	O35593												
			-0.66074	0.48076	P05132												
			-0.65913	0.219317	Q9D2G2												
			-0.65798	0.389692	Q99LH1												
			-0.6573	0.586839	P49442												
			-0.65679	0.220139	Q9JLJ2												
			-0.65093	0.439218	Q6P4T2												
			-0.64726	0.7037	Q9CXT8												
			-0.64716	1.081842	Q3THS6												
			-0.64623	0.651864	Q9CWJ9												
			-0.64286	0.610943	Q9R1T2												
			-0.64173	1.188454	P60335												
			-0.63779	0.476956	Q1HFZ0												
			-0.63772	0.573031	Q91WQ3												
			-0.63744	0.285468	Q8R2U4												
			-0.6369	0.203976	Q9CRC8												
			-0.63603	0.202256	Q9CYZ2												
			-0.63553	0.557517	Q9CRB2												
			-0.63435	0.396268	Q9JIX8												
			-0.63351	0.371859	Q9CQR2												
			-0.63129	0.319649	P19783												
			-0.62954	0.770419	O55125												
			-0.62896	0.219643	Q61035												
			-0.62692	1.175336	P67984												
			-0.6248	1.20063	Q922D8												
			-0.62372	0.515949	Q8VBT0												
			-0.62256	0.787186	P62911												
			-0.62178	0.415232	Q505F5												
			-0.61826	0.232358	Q6PB44												
			-0.6181	0.747762	Q9WUM5												
			-0.61525	0.512108	P05064												
			-0.61261	0.505621	Q9R0P9												
			-0.61261	1.142261	P53026												
			-0.61213	0.208841	Q91YU3												
			-0.6118	0.833207	Q60605												
			-0.60927	0.141631	P35761												
			-0.60713	0.311082	Q91YT0												
			-0.60346	0.262656	Q99KN9												
			-0.60336	0.479975	Q9CQI6												
			-0.60244	0.133818	Q921F4												
			-0.59816	0.247606	Q8K3W0												
			-0.59764	0.480112	P46460												
			-0.59553	0.162789	Q8BG81												
			-0.59085	0.312464	Q9DBE9												
			-0.59026	0.897203	P02798												
			-0.59026	0.897203	P60060												
			-0.59004	0.553176	O88685												
			-0.58515	1.017386	Q8R081												
			-0.58509	1.036655	P84099												
			-0.58103	0.309455	Q91YR1												
			-0.58099	0.591304	E9PYK3-DECOY											
			-0.57446	0.293495	O08547												
			-0.57352	0.523221	Q8K363												
			-0.57254	0.822492	P61255												
			-0.57187	0.270882	Q9CZU3												
			-0.56896	0.613069	P61161												
			-0.56758	0.194828	Q9WTQ5												
			-0.56458	1.22659	Q8VEK3												
			-0.56434	0.249396	P50518												
			-0.56193	0.364097	Q9WU78												
			-0.55863	0.338723	Q0VGU4												
			-0.55721	0.501258	P32067												
			-0.55719	0.313079	Q9EPL8												
			-0.5507	0.488024	Q99J99												
			-0.5492	1.027074	P62827												
			-0.54662	0.128326	Q3TCH7												
			-0.54662	0.128326	Q8BGX2												
			-0.54563	1.027304	Q9CZD3												
			-0.54533	0.861123	P42932												
			-0.54138	0.217613	Q9DBY8												
			-0.54062	0.347781	P28740-2												
			-0.53663	0.93133	Q9Z0N1												
			-0.53264	0.216535	Q9D4H8												
			-0.53152	0.239918	Q8BG05												
			-0.53023	0.734746	Q01320												
			-0.52818	0.188249	Q99PV0												
			-0.52595	0.17102	O88543												
			-0.52372	0.162349	Q9CXJ1												
			-0.52285	0.396219	Q920B9												
			-0.5206	0.586227	Q8VDD5												
			-0.51987	0.107904	Q8CFE2												
			-0.51527	0.197929	P47758												
			-0.51432	0.674196	Q8VE37												
			-0.51383	0.830494	P97461												
			-0.51312	0.275148	Q8BXZ1												
			-0.51016	0.41369	P62196												
			-0.50708	0.15136	Q99M31												
			-0.50613	0.608246	Q8CGC7												
			-0.50195	0.137647	Q9D1B9												
			-0.49914	0.232695	Q99MJ9												
			-0.49609	0.442726	Q6PGG6												
			-0.4952	0.248294	Q62465												
			-0.49325	0.137798	Q3UJD6-2												
			-0.49273	0.26536	P63168												
			-0.49043	0.18717	A2A432												
			-0.48555	1.11647	P62821												
			-0.48298	0.190495	Q8CJG0												
			-0.47563	0.629958	Q9WVA4												
			-0.47028	0.173896	Q9Z1Z0												
			-0.4678	0.980631	Q9Z1Q5												
			-0.46779	0.937929	P84104												
			-0.46723	0.250538	O88696												
			-0.467	0.185668	Q8VHE0												
			-0.46533	1.126469	P54822												
			-0.46441	0.601982	Q8BP67												
			-0.46393	0.642964	Q61699												
			-0.45931	0.148749	Q9D2Y4-2												
			-0.45301	0.139776	Q8N9S3												
			-0.45221	0.079637	Q6PGF7												
			-0.45056	0.210979	Q9D8M4												
			-0.44841	0.780955	Q9CXW4												
			-0.44806	0.664426	Q9R0N0												
			-0.44767	0.160372	Q9DBZ5												
			-0.44755	0.298057	Q3UPL0												
			-0.44133	0.980107	Q91V12-2												
			-0.44038	0.994617	Q6ZWV3												
			-0.4376	0.175789	Q9D0N7												
			-0.43498	0.245414	Q91WK2												
			-0.43416	0.100236	Q3UQ84												
			-0.4316	0.310781	Q9QUR6												
			-0.4315	0.776764	P62900												
			-0.42816	0.963124	P14873												
			-0.42644	0.354081	P62918												
			-0.42384	0.152383	Q8K4B0												
			-0.42172	0.364882	P83882												
			-0.42042	0.322357	P63037												
			-0.41974	0.275139	D3YXK2												
			-0.41538	0.291862	Q9D1D4												
			-0.41375	0.723252	Q9D0I9												
			-0.4108	0.525685	P35700												
			-0.40828	0.104457	P52431												
			-0.40828	0.104457	Q9Z0V7												
			-0.40574	0.762103	P35279												
			-0.40558	0.273922	O35972												
			-0.40338	0.070007	P24369												
			-0.40338	0.070007	Q7TPR4												
			-0.40338	0.070007	Q8BHE8												
			-0.40338	0.070007	Q8BZA9												
			-0.40338	0.070007	Q8K2J0												
			-0.40338	0.070007	Q91YN9												
			-0.39147	0.469498	P41105												
			-0.38794	1.151855	Q78PY7												
			-0.38513	0.540028	Q9CZ30												
			-0.38421	0.690828	P62874												
			-0.38285	1.165852	P18760												
			-0.38246	0.280733	Q923T9												
			-0.38219	0.543175	P70168												
			-0.37902	0.398265	Q9D1R9												
			-0.37776	0.180995	Q8R191												
			-0.37653	0.555389	P17710-3												
			-0.36591	0.169561	Q9D0T1												
			-0.36344	0.080818	O09106												
			-0.35926	0.260997	Q9JM76												
			-0.35842	0.36007	Q8BHC4												
			-0.35522	0.29055	Q91VE6												
			-0.35277	0.575376	P62880												
			-0.35039	0.371933	Q99L45												
			-0.34988	0.347369	P68510												
			-0.34956	0.497448	P97310												
			-0.34749	0.102609	O88712												
			-0.34564	0.93001	Q9JIK5												
			-0.34411	0.71371	P07901												
			-0.34281	0.268464	P34022												
			-0.34173	0.346773	Q8C0C7												
			-0.34169	0.23648	Q9D0R2												
			-0.33945	1.111491	Q91V92												
			-0.33742	0.860447	P61222												
			-0.33686	0.333165	Q8BG05-2											
			-0.33586	0.057082	Q5SSW2												
			-0.33586	0.057082	Q8BG79												
			-0.33586	0.057082	Q9EPU4												
			-0.33315	0.09967	Q8BG51-2-DECOY											
			-0.33288	0.553301	P27773												
			-0.33283	0.112984	Q6P3A8												
			-0.32996	0.102523	P24788												
			-0.32939	0.063714	Q8BKS9												
			-0.32808	0.088306	Q5EG47												
			-0.32768	0.31653	O88569-3												
			-0.32004	0.779667	O35737												
			-0.31549	0.318075	P62717												
			-0.31248	0.403686	O08807												
			-0.31106	0.170498	Q60715-2												
			-0.30949	0.198502	Q9JKY0												
			-0.30614	0.430131	P47964												
			-0.30513	0.090627	Q61701												
			-0.30472	0.745933	P97351												
			-0.30346	0.142771	P97478												
			-0.30264	0.191442	Q9CZW5												
			-0.30183	0.128497	Q99JX4												
			-0.30042	0.445499	P60867												
			-0.30023	0.498001	P62852												
			-0.2984	0.237429	Q99JB2												
			-0.29596	0.057018	Q6PAR5												
			-0.29381	0.311837	P26638												
			-0.29332	0.111258	Q9CPW4												
			-0.29256	0.117078	P70698												
			-0.2904	0.135704	Q9ER88												
			-0.28219	0.595874	P80313												
			-0.27967	0.046683	P23116-DECOY											
			-0.27967	0.046683	Q8VE73												
			-0.27345	0.203975	Q9CZ42												
			-0.27287	0.175943	Q9CQ88												
			-0.27198	0.543254	Q9Z1N5												
			-0.27196	0.272655	P62334												
			-0.27191	0.290771	Q99PL5												
			-0.27018	0.449912	Q6DFW4												
			-0.26983	0.31995	Q9DCD0												
			-0.26914	0.435237	Q9QYB1												
			-0.26857	0.233276	P46471												
			-0.26755	0.152001	Q922W5												
			-0.26718	0.05841	Q8R2Y8												
			-0.26718	0.05841	Q8VCX5												
			-0.26669	0.199934	P62869												
			-0.26488	0.069897	P32037												
			-0.26478	0.201809	O54774												
			-0.26436	0.701021	P80314												
			-0.26354	0.069338	P60879												
			-0.25817	0.487003	Q9CQ69												
			-0.25497	0.095676	P09055-2												
			-0.25006	0.183655	Q78IK4												
			-0.25006	0.183655	Q9CQV7												
			-0.24896	0.071136	Q5SWU9												
			-0.24761	0.411894	Q922K7												
			-0.24694	0.433309	Q922F4												
			-0.24589	0.425435	P62270												
			-0.24576	0.121563	P62305												
			-0.24372	0.082944	P97372												
			-0.23919	0.083257	O35382												
			-0.23874	0.362285	P63038												
			-0.23859	0.713447	Q9Z204-3												
			-0.23782	0.862164	P03975												
			-0.23716	0.329322	P62849												
			-0.23674	0.282902	Q6ZWN5												
			-0.23518	0.31199	Q9D0E1												
			-0.23491	0.229931	Q64674												
			-0.23441	0.070891	Q8BNI4												
			-0.2324	0.506829	Q99K48												
			-0.22963	0.090466	Q6P9R1												
			-0.22806	0.546257	P62889												
			-0.22738	0.369353	P63260												
			-0.22733	0.513835	P54116												
			-0.22673	0.077256	Q9EP69												
			-0.22664	0.244497	P70333												
			-0.22153	0.217195	Q924T2												
			-0.21994	0.287399	Q05920												
			-0.21916	0.152902	Q9D0Q7												
			-0.21039	0.064263	Q9Z1G3												
			-0.20979	0.429608	Q61881												
			-0.20823	0.363868	P62855												
			-0.20717	0.206854	P63005												
			-0.20589	0.120682	Q9D0I8												
			-0.2053	0.097078	Q80YD1												
			-0.20322	0.060772	A2AR02												
			-0.20007	0.665967	Q8BVE3												
			-0.20003	0.053155	Q8R0A0												
			-0.20003	0.053155	Q9WTX2												
			-0.19968	0.15158	P17742												
			-0.19909	0.120082	Q8BMA6												
			-0.19888	0.05497	Q99JF8												
			-0.19868	0.953205	Q61656												
			-0.19855	0.373046	Q921J2												
			-0.19816	0.173868	P63330												
			-0.19767	0.330671	Q922R8												
			-0.19456	0.536459	P80318												
			-0.19355	0.078735	Q7TMK9												
			-0.19355	0.078735	Q8BMS4												
			-0.19298	0.250675	P19253												
			-0.19101	0.655121	P58252												
			-0.18944	0.126232	Q9DBP5												
			-0.18822	1.142386	P62983												
			-0.18749	0.267703	P67778												
			-0.18545	0.657012	P68369												
			-0.18459	0.395204	P47857												
			-0.18293	0.166729	P63325												
			-0.18247	0.324603	P62267												
			-0.181	0.752908	Q9ERD7												
			-0.17961	0.03266	Q4VA53												
			-0.1769	0.301767	O55143												
			-0.17378	0.060073	Q80X41												
			-0.1717	0.171718	Q9R190												
			-0.16948	0.052281	Q3UL36												
			-0.1691	0.250672	P62281												
			-0.16838	0.127717	Q6ZWX6												
			-0.16697	0.153166	Q8BTM8												
			-0.1669	0.093074	Q8CG48												
			-0.16641	0.214343	Q9WVA3												
			-0.16541	0.475702	P17182												
			-0.1653	0.035308	Q8BSY0												
			-0.16287	0.25589	P80315												
			-0.16021	0.20972	Q8K224												
			-0.15962	0.046346	Q8BL97												
			-0.15573	0.29478	Q61753												
			-0.15472	0.032923	Q8C5Q4												
			-0.15472	0.032923	Q9DCJ7												
			-0.15312	0.061145	Q8R1F6												
			-0.15281	0.104688	P32883												
			-0.14984	0.081635	P62141												
			-0.14979	0.064258	Q62203												
			-0.14818	0.19688	Q91VC3												
			-0.1451	0.0441	P97762												
			-0.14463	0.045424	P97820												
			-0.14417	0.11501	P48678												
			-0.14146	0.048424	Q9D394-2												
			-0.13941	0.137417	P47757-2												
			-0.13588	0.080297	Q9ERK4												
			-0.13502	0.147631	Q9CPR4												
			-0.13375	0.131404	P61079												
			-0.13263	0.044002	P14152												
			-0.1324	0.049207	Q9QXK3												
			-0.12959	0.072101	Q9DBD5												
			-0.12955	0.046994	Q8BY71												
			-0.12678	0.053908	Q9CQ22												
			-0.1233	0.112063	Q8BG32												
			-0.12255	0.147883	Q61335												
			-0.12218	0.044612	Q91YR7												
			-0.11989	0.108025	Q9WV60												
			-0.11965	0.366159	P80317												
			-0.11908	0.356104	P05213												
			-0.11425	0.285004	P62806												
			-0.11384	0.161768	P10854												
			-0.11358	0.204456	P08030												
			-0.1121	0.040325	Q05CL8												
			-0.11173	0.109082	P63001												
			-0.11007	0.038746	Q9CR68												
			-0.10709	0.07732	Q9WVJ2												
			-0.10448	0.055375	Q62351												
			-0.10399	0.183968	Q61598												
			-0.10018	0.045776	P84089												
			-0.10016	0.034203	Q80SZ7												
			-0.0989	0.122895	Q9WUA2												
			-0.0978	0.191398	P62908												
			-0.0972	0.042309	P45952												
			-0.0954	0.127862	P51881												
			-0.09209	0.206039	P48962												
			-0.09011	0.067598	Q9D0M1												
			-0.08775	0.138939	P62754												
			-0.08757	0.021066	Q8BYH7-DECOY											
			-0.08593	0.074132	P52293												
			-0.08463	0.017003	Q9CR98												
			-0.08243	0.031229	Q9D6M3												
			-0.07882	0.052878	P62492												
			-0.07578	0.094585	Q8CGP0												
			-0.07262	0.087227	P35979												
			-0.07171	0.045838	P62274												
			-0.07119	0.050041	Q9QYI3												
			-0.06892	0.028112	Q9D0R8												
			-0.06052	0.078829	P51410												
			-0.05773	0.068653	Q9CZU6												
			-0.05772	0.014445	Q9Z2U1												
			-0.05675	0.108953	P60843												
			-0.05261	0.041549	Q99JY9												
			-0.05082	0.013038	P22315												
			-0.04726	0.050465	Q6ZWU9												
			-0.0467	0.099609	Q8QZT1												
			-0.04474	0.037308	P48758												
			-0.04198	0.022674	Q7TQI3												
			-0.04186	0.027545	Q64012												
			-0.04055	0.010187	Q91WK5												
			-0.03717	0.00571	A6H611												
			-0.03717	0.00571	P35441												
			-0.03717	0.00571	Q8BXR9												
			-0.03717	0.00571	Q91W67												
			-0.03418	0.033279	Q9WUA3												
			-0.03185	0.006748	O35643												
			-0.03123	0.014579	Q76MZ3												
			-0.02553	0.022857	P62242												
			-0.02396	0.015361	Q9WV32												
			-0.02255	0.005816	Q9JJY3												
			-0.02213	0.004848	O54692												
			-0.02213	0.004848	Q60596												
			-0.02095	0.012337	Q8R4R6												
			-0.02041	0.005966	Q8BX17												
			-0.01944	0.031281	P14869												
			-0.01755	0.015901	Q922Q4												
			-0.01597	0.006316	P50136												
			-0.01326	0.003324	P18526												
			-0.01274	0.002623	Q60960												
			-0.01238	0.021037	Q9D358												
			-0.01055	0.002355	Q9DCF9												
			-0.0099	0.008416	Q8BVY0												
			-0.00928	0.00418	Q80X73												
			-0.00824	0.001252	P70388												
			-0.00824	0.001252	Q99N85												
			-0.00797	0.008295	Q9D7G0												
			-0.00437	0.002003	Q9DCG9												
			-0.0026	5.81E-04	P39053												
			-8.25E-04	0.002547	Q9CZN7												
			2.30E-04	1.87E-04	P47911												
			3.88E-04	1.84E-04	P19246												
			0.001478	0.001422	P62301												
			0.002567	0.0018	Q8BMJ2												
			0.003508	0.005158	P19096												
			0.00425	0.005542	P47754												
			0.007765	0.003882	Q7TMB8												
			0.0081	0.006716	P53986												
			0.010903	0.004055	Q05915												
			0.013003	0.010114	Q9CQV8												
			0.016468	0.006603	P12787												
			0.019454	0.004643	Q6ZQ73												
			0.020014	0.019281	E9Q5C9												
			0.02591	0.131074	P61979												
			0.030177	0.009581	Q8C0L0												
			0.030177	0.009581	Q99KF1												
			0.03136	0.025673	P63101												
			0.031908	0.029731	P35278												
			0.032799	0.019511	Q6ZWY3												
			0.042389	0.010532	Q5RJG1												
			0.043437	0.043193	Q922B2												
			0.043855	0.071842	P56480												
			0.044539	0.008747	Q9QXK7												
			0.044988	0.024576	P70404												
			0.045075	0.09216	Q80XN0												
			0.045351	0.061497	P20152												
			0.045389	0.053551	P23116												
			0.047524	0.103944	Q9CQQ7												
			0.049428	0.013009	Q9CXF4												
			0.049997	0.021619	Q8BYA0												
			0.05184	0.00785	P23475												
			0.05184	0.00785	Q8BW94												
			0.056657	0.084304	O70133-2												
			0.057534	0.027612	Q9CQA3												
			0.058488	0.021356	Q8VI75												
			0.066244	0.053319	Q9R0P5												
			0.067337	0.023124	P59328												
			0.068633	0.010389	Q640N3												
			0.068633	0.010389	Q9R0Q4												
			0.069606	0.095918	P35550												
			0.07104	0.063595	Q9Z2X1												
			0.072199	0.040317	P55258												
			0.072701	0.018495	P40142												
			0.075975	0.092711	P62830												
			0.081318	0.071464	Q9JKC6												
			0.084335	0.122521	Q99JI4												
			0.085021	0.030577	Q9DCT2												
			0.085024	0.16409	O35129												
			0.085948	0.17655	P14206												
			0.087088	0.028365	Q9JJY4												
			0.08769	0.017281	Q9DCD2												
			0.087971	0.102686	Q91ZW3												
			0.088432	0.046442	Q8BK64												
			0.089905	0.056733	P70372												
			0.090457	0.029727	Q8R180												
			0.091279	0.031301	Q922H4												
			0.094659	0.171229	P08113												
			0.096669	0.023681	Q8BK63												
			0.09743	0.070674	P61089												
			0.110753	0.067614	P49312												
			0.113129	0.082365	Q922Q8												
			0.113835	0.118936	P25206												
			0.115746	0.165177	Q9CYH6												
			0.116146	0.314324	Q8BMS1												
			0.117852	0.212186	P12382												
			0.119082	0.019553	O88986												
			0.120567	0.031023	Q9CQD1												
			0.120718	0.061696	Q62186												
			0.12187	0.026081	P30999-3												
			0.12187	0.026081	Q63918												
			0.12187	0.026081	Q9DCS3												
			0.12219	0.089137	Q8BU30												
			0.129422	0.04445	Q61578												
			0.131145	0.228022	Q8BP47												
			0.131201	0.165941	P14148												
			0.135718	0.06278	Q9D8V0												
			0.13606	0.112789	Q8R050												
			0.139781	0.169618	P80316												
			0.141625	0.207195	P18872-2												
			0.143463	0.179722	Q6PHZ2												
			0.145061	0.031326	Q9CVI2												
			0.145446	0.054166	O08788												
			0.146733	0.158917	Q8R127												
			0.153914	0.209686	P63323												
			0.154691	0.108267	Q9ESV0												
			0.158057	0.106164	P26443												
			0.16121	0.291647	P15331												
			0.162962	0.050307	O54908												
			0.163008	0.048787	Q9QXS1												
			0.164298	0.02478	A2ASS6												
			0.165221	0.116128	Q9DBR1												
			0.1679	0.164145	P61082												
			0.170149	0.200931	Q9R0E1												
			0.173299	0.11493	Q9D819												
			0.174291	0.062473	Q9ESX5												
			0.175239	0.110206	Q9DB77												
			0.177082	0.200781	Q60932-2												
			0.177575	0.184937	Q9DBC7												
			0.180658	0.05823	P01636												
			0.181092	0.02729	Q8BK35												
			0.181585	0.027364	Q9CQC7												
			0.182007	0.067939	O35841												
			0.182476	0.049541	Q9CZW4												
			0.183191	0.195033	P35564												
			0.183381	0.113852	P61924												
			0.184255	0.06801	Q99M28												
			0.188103	0.178943	Q91UZ5												
			0.188357	0.385696	Q9JKV1												
			0.188649	0.099266	Q6ZWV7												
			0.197162	0.069164	O35345												
			0.197959	0.223824	Q60931												
			0.199803	0.03829	Q8VCB1												
			0.200148	0.039899	Q9D0F6												
			0.201873	0.467821	Q91YU8												
			0.202082	0.347605	Q8VDW0												
			0.2042	0.189277	P62264												
			0.205502	0.079781	Q9DC28												
			0.207675	0.41343	Q9D6R2												
			0.211569	0.39514	Q9JKR6												
			0.211631	0.069135	Q64337												
			0.212374	0.075018	Q3TKR3												
			0.213965	0.250554	P17427												
			0.21833	0.13678	Q9CPQ1												
			0.219572	0.244283	Q8BKC5												
			0.22051	0.358078	P62843												
			0.227312	0.143877	P07356												
			0.228158	0.488908	Q9CXS4												
			0.236	0.610055	Q61024												
			0.236511	0.064226	P10630												
			0.23783	0.087117	P28667												
			0.239356	0.136577	P61164												
			0.2397	0.066935	P52430												
			0.240214	0.154513	Q91VD9												
			0.241133	0.197722	Q8JZQ9												
			0.248947	0.119108	Q8BH59												
			0.250027	0.150435	Q6PGC1												
			0.250726	0.192362	Q8VDM4												
			0.252634	0.375976	P68040												
			0.253643	0.101517	P70122												
			0.255668	0.076738	Q3U308												
			0.258797	0.463123	Q8K297												
			0.259618	0.085968	Q91YE6												
			0.260896	0.123031	Q9CQR6												
			0.26517	0.057945	Q9DC71												
			0.26676	0.274324	P29341												
			0.266765	0.099723	Q9JM93												
			0.267084	0.092326	P01631												
			0.267499	0.139263	Q8C0I1												
			0.268077	0.298709	P12970												
			0.268966	0.64025	P49717												
			0.272978	0.083476	P18654												
			0.273736	0.126846	Q9QUM9												
			0.278126	0.07134	Q99LR1												
			0.285117	0.282795	Q9CZS1												
			0.287303	0.774953	Q03265												
			0.290321	0.280915	Q9D0K2												
			0.290339	0.173006	Q99L47												
			0.292229	0.800209	Q501J6												
			0.293125	0.313922	Q9CZM2												
			0.293878	0.271495	P08752												
			0.29434	0.044044	P41216												
			0.29434	0.044044	Q9DCZ4-3											
			0.29434	0.044044	Q9JME5												
			0.298123	0.512004	P49718												
			0.298498	0.795532	Q62167												
			0.299523	0.638747	P62702												
			0.300099	0.674678	P61358												
			0.304573	0.257927	Q8BWY3												
			0.305725	0.123158	P51863												
			0.30596	0.285643	Q6P5B0												
			0.306821	0.270469	P16045												
			0.306921	1.032983	Q3V3R1												
			0.30794	0.3809	Q8CGP7												
			0.311173	0.269034	Q8JZU2												
			0.311223	0.179962	Q91VY9												
			0.316334	0.087895	Q9JLV5												
			0.320101	0.691603	Q9D8E6												
			0.330092	0.224045	Q922Y1												
			0.334993	0.998825	P06151												
			0.342067	0.44865	P83870												
			0.343687	0.19971	Q9CX34												
			0.349217	0.274449	Q91V41												
			0.354917	0.08657	Q9CQ75												
			0.355308	0.115114	Q9JIG8												
			0.357868	0.12418	Q61595												
			0.358178	0.331345	P51150												
			0.358532	0.390771	P63094												
			0.358713	0.156799	Q9DBG3												
			0.363696	0.300258	P23198												
			0.364029	0.084268	Q9D0F3												
			0.364609	0.237241	Q9JIF7												
			0.365542	0.685142	Q61879												
			0.36757	0.583995	Q9QZE5												
			0.371044	0.316239	Q9QUJ7												
			0.371119	0.117035	Q62191												
			0.37122	0.387337	P27659												
			0.371603	0.636923	Q8BH04												
			0.373316	0.891087	P97807												
			0.378996	0.105875	Q9CR59												
			0.390043	0.532087	Q9DC69												
			0.394963	0.141401	Q9R0Q6												
			0.399763	0.144869	Q9CY66												
			0.399763	0.144869	Q9WTQ8												
			0.400101	0.45011	Q9JJI8												
			0.40143	0.366094	Q61316												
			0.403136	0.250338	Q99L04												
			0.403402	0.359027	Q99JY0												
			0.403831	0.205734	O08599												
			0.406799	0.060301	Q9D773												
			0.410816	0.115712	Q9Z2U0												
			0.418811	0.152101	Q7TMF3												
			0.420273	0.346706	Q9D883												
			0.423396	0.150697	Q9CY27												
			0.425565	0.207964	P23591												
			0.434534	0.368928	P15864												
			0.435511	0.145545	Q60973												
			0.435852	0.489537	Q8BJY1												
			0.437334	0.595463	Q9DCW4												
			0.44108	0.207789	P50171												
			0.444156	0.919107	Q9CQN1												
			0.449177	0.331939	P43274												
			0.450258	0.416948	Q9Z2I9												
			0.452249	0.532126	Q791V5												
			0.453384	0.100886	Q8CDN6												
			0.453384	0.100886	Q8K1R3												
			0.453384	0.100886	Q9WUP7												
			0.455701	0.275103	Q8BMK4												
			0.458085	0.30881	P61957												
			0.45814	0.517174	Q99KI0												
			0.45943	0.537158	Q8BJ71												
			0.460416	0.659526	P62862												
			0.460548	0.272847	Q9ERU9												
			0.46361	0.134172	Q5XKN4												
			0.465885	0.295952	O08528												
			0.466341	0.359723	P43277												
			0.468439	0.669694	P35293												
			0.471678	0.597532	Q91YQ5												
			0.476215	0.303474	P10922												
			0.478551	0.279996	Q5SUF2-2												
			0.479792	0.227075	P51912												
			0.48009	0.140436	P97855												
			0.486707	0.47807	Q9JJ80												
			0.48688	0.180074	Q9WUK4												
			0.487896	1.290224	Q9DCN2												
			0.488511	0.099002	Q9CY16												
			0.490607	0.808245	P62751												
			0.493252	0.32306	P08003												
			0.494927	0.095011	Q9CWZ3												
			0.495445	0.278038	Q9CPY7												
			0.499126	0.367775	Q9Z2I0												
			0.50682	0.502954	P01942												
			0.51089	0.404066	Q9CR62												
			0.514106	0.383349	Q8K411												
			0.518156	0.454259	P49183												
			0.518206	0.301423	O35857												
			0.520332	0.115104	Q8C1E7												
			0.520926	0.929449	Q9D051												
			0.521162	0.504035	Q9JHU4												
			0.523537	0.846734	Q99LE6												
			0.524668	0.353172	Q8R5C5												
			0.529569	0.19991	Q91YR5												
			0.533137	0.20166	Q99LX5												
			0.53493	0.135857	Q921M3												
			0.540655	0.150396	P46735												
			0.5446	0.157906	Q61398												
			0.54665	0.592507	Q9DB20												
			0.546843	0.315494	Q99N84												
			0.547495	0.330539	P45376												
			0.547789	0.080001	Q921G7												
			0.547789	0.080001	Q9Z1T1												
			0.548408	1.277281	Q8BFR5												
			0.549052	0.250203	Q99KQ4												
			0.549324	0.126115	O35226												
			0.553727	1.243893	Q62095												
			0.553911	1.080302	Q8CGK3												
			0.553983	0.743957	Q924C1												
			0.555857	0.669046	Q9QZD9												
			0.558006	0.524256	P62878												
			0.559771	0.658193	Q925I1												
			0.56486	0.317007	O55142												
			0.568345	0.593337	Q6ZQ38												
			0.576978	0.731679	Q62159												
			0.577204	0.578171	P27661												
			0.578537	1.258209	Q60930												
			0.581475	0.598961	Q9QZQ8												
			0.58486	0.255048	Q8R480												
			0.588759	0.946112	P62245												
			0.591474	0.322763	Q9D7H3												
			0.593269	0.29627	Q9CRB9												
			0.597941	0.819173	Q9QUI0												
			0.598812	0.179843	Q7SIG6												
			0.600336	0.149449	P18608												
			0.605309	0.993647	Q8R0X7												
			0.606965	0.564562	Q61941												
			0.607355	0.607897	Q9R0E2												
			0.608772	1.2819	P14131												
			0.60897	0.219056	E9PV24												
			0.610398	0.369317	P27601												
			0.612577	0.614306	Q8K2B3												
			0.613468	0.426761	P43276												
			0.613862	0.735531	P35486												
			0.617805	1.102896	O54734												
			0.6228	0.343702	P62835												
			0.624508	0.381443	Q9EQH3												
			0.632246	1.293702	P04223												
			0.632827	0.320988	Q8VH51												
			0.632884	0.289909	P63073												
			0.633804	0.213914	Q9DB42												
			0.634235	0.648192	Q9DBG6												
			0.635887	0.7475	Q8BH95												
			0.637568	0.638476	Q99LX0												
			0.638319	0.601268	Q8R323												
			0.639724	0.147779	P70699												
			0.639744	0.39774	Q99NB9												
			0.639898	0.891105	Q68FD5												
			0.643207	0.250166	P29788												
			0.658312	0.218264	P18528												
			0.660892	0.515913	Q6PDI5												
			0.665091	0.181214	Q9EST5												
			0.6681	0.234692	Q6A026												
			0.674874	0.303825	Q61136												
			0.676434	0.58519	P26041												
			0.685569	0.224883	Q8BMC4												
			0.686069	0.279662	Q9Z0R9												
			0.688118	0.746589	Q6GSS7												
			0.690157	0.210456	Q9CPS7												
			0.691029	0.689652	P11440												
			0.691141	0.487701	Q3TDN2												
			0.699013	0.589344	Q9QYJ0												
			0.707603	0.285852	Q9D0F4												
			0.710033	0.472483	P62962												
			0.712045	0.150561	O88947												
			0.71314	0.489188	Q9QXB9												
			0.713953	0.338141	Q8BM55												
			0.716012	0.216073	P01812												
			0.718449	0.210563	Q99LG2												
			0.722246	0.15612	Q99KD5												
			0.724596	0.736013	P68033												
			0.733204	0.588943	Q8BYL4												
			0.733386	0.441192	Q60597-3												
			0.733505	0.919425	P97311												
			0.740713	0.274712	O70311												
			0.74125	0.275665	Q8K0C4												
			0.743622	1.254984	Q9EQ61												
			0.745213	0.639884	P01900												
			0.747507	0.421868	P84091												
			0.747517	1.102365	P50580												
			0.748704	0.540771	Q9CQW9												
			0.749366	0.273652	P70279												
			0.750281	0.238507	P48024												
			0.75269	0.544403	Q8BTI8												
			0.756176	0.152025	Q8R570												
			0.761054	0.666505	P56135												
			0.765241	0.451607	Q8C7U1												
			0.767637	0.661498	Q9WV70												
			0.772226	0.859762	Q9D8W5												
			0.775986	0.450184	Q9D1I6												
			0.780207	0.222935	Q61387												
			0.783333	1.109317	P51660												
			0.78347	0.510709	Q04750												
			0.784577	0.406946	O08784												
			0.789025	0.462054	Q9D0D4												
			0.789695	0.738958	P43275												
			0.801559	0.420745	P29699												
			0.804296	0.306452	Q8CGZ0												
			0.80608	0.36775	Q8CG16												
			0.809887	1.12385	Q9DCH4												
			0.817286	0.257143	Q9DCU6												
			0.823366	0.391143	P40336												
			0.824778	0.622261	Q9CZR8												
			0.82569	0.395353	O08692												
			0.826859	0.355378	P06330												
			0.828754	0.911384	Q6P5F9												
			0.829507	0.876323	Q9JLJ5												
			0.829885	0.372137	P97868												
			0.840095	0.283005	Q9CPR5												
			0.842112	0.186862	P13705												
			0.842112	0.186862	P63280												
			0.842112	0.186862	Q6A068												
			0.847285	0.509657	P50544												
			0.848962	0.493702	P57724												
			0.848962	0.493702	Q9QYA2												
			0.851062	1.202169	Q8BFY9												
			0.851846	0.188707	P63166												
			0.852259	0.339598	P01634												
			0.854286	0.330131	P97430												
			0.859301	0.254994	Q9D945												
			0.864181	0.711585	Q8K0D5												
			0.866159	0.402167	P08249												
			0.867146	0.273329	P01897												
			0.869633	0.512827	Q6IFZ6												
			0.875884	0.332545	Q8JZX4												
			0.877406	0.818052	P0C0S6												
			0.879191	0.365712	Q61001												
			0.879191	0.365712	Q9D1C9												
			0.879303	0.122615	O55135												
			0.879303	0.122615	Q8K3Z9												
			0.879303	0.122615	Q9R0M6												
			0.879931	0.402691	P20918												
			0.881928	0.800595	P70398												
			0.884327	0.367881	Q99N89												
			0.888512	0.594782	Q80UU9												
			0.889147	0.534265	P10852												
			0.890206	0.271341	Q99M87												
			0.892678	0.742414	P10649												
			0.897483	0.39309	E9Q7G0												
			0.898782	0.46639	Q9D0M3												
			0.900959	0.375272	Q62136												
			0.900959	0.375272	Q8BH79												
			0.911081	0.28143	P83887												
			0.916866	0.390986	Q91X72												
			0.922439	0.568235	Q9QX47												
			0.923687	0.345638	Q99J95												
			0.927066	0.359291	O08677-2												
			0.92812	0.297924	Q9EQI8												
			0.929492	0.460829	P24270												
			0.930209	1.059226	P60229												
			0.933758	0.294379	Q6PFR5												
			0.942283	0.289106	Q8BHF7												
			0.942283	0.289106	Q9D903												
			0.945905	0.725615	Q9D880												
			0.94853	0.59596	P70245												
			0.952245	0.99562	Q9JKX6												
			0.955321	0.174204	Q6PFD9												
			0.962826	0.132438	Q8BRT1												
			0.96802	0.605576	Q99KV1												
			0.974616	0.841609	Q920E5												
			0.982134	0.587585	P70399												
			0.984704	0.738161	P02088												
			0.987256	0.212345	Q62093												
			0.990551	0.549645	P07724												
			0.995691	0.216313	O88508												
			0.995718	0.405172	P97376												
			1.007818	0.340225	Q9ESB3												
			1.016748	0.501717	Q9ESX4												
			1.018281	0.222113	O35114												
			1.018281	0.222113	Q5U458												
			1.021184	1.257584	Q8BGD9												
			1.025134	0.223855	O35658												
			1.028517	0.489534	Q61140												
			1.030244	1.17111	P58059												
			1.052958	0.475859	Q3UQU0												
			1.06001	1.159863	Q8CAQ8-2											
			1.061149	0.682292	P18527												
			1.061526	0.925464	P51174												
			1.068633	0.144328	P55264												
			1.071519	0.672955	B2RY56												
			1.077996	0.500173	P28798												
			1.083993	0.352009	Q60737												
			1.08704	0.276958	Q9DCA5												
			1.08769	0.21282	Q8VE33												
			1.088215	0.563676	P01863												
			1.092559	0.507289	P01639												
			1.094795	1.203758	Q60716												
			1.108776	1.100056	Q99ME9												
			1.120567	0.344681	P39054												
			1.12187	0.247387	Q571H0												
			1.128373	1.02668	Q61703												
			1.129976	0.514215	Q91XV3												
			1.131022	0.587303	Q8BMF4												
			1.135608	0.36206	Q920A7												
			1.137038	0.467962	P54923												
			1.140943	0.429545	E9Q5G3												
			1.146272	0.742512	Q8C3X8												
			1.150572	0.370285	Q9CXW2												
			1.153754	0.287338	Q62448												
			1.158458	0.796331	P62960												
			1.168544	0.991903	Q8VEH3												
			1.178179	0.259317	Q9EPK7												
			1.178179	0.259317	Q9Z210												
			1.178347	1.039433	P20108												
			1.180702	0.945015	P17426												
			1.181585	0.156339	Q8R0G9												
			1.185896	0.261028	Q9D6K9												
			1.186499	0.497934	Q99LC8												
			1.187967	0.518432	E9Q555												
			1.199899	0.546032	P97770-2												
			1.204928	0.802918	P09405												
			1.205151	0.711738	Q02105												
			1.208367	0.558807	P01820												
			1.209676	1.168508	Q9D0J4												
			1.226314	0.411822	Q9DBM1												
			1.22678	0.499831	Q9Z277												
			1.235441	0.71854	Q6PHN9												
			1.239849	0.56145	P07759												
			1.249099	0.163182	Q3UEB3												
			1.25608	0.554057	O70310												
			1.266538	0.3935	Q60710												
			1.266765	0.894684	O35682												
			1.267227	0.763571	Q8K1M6												
			1.308636	0.995348	Q9QYF1												
			1.308846	1.245964	Q8CI94												
			1.312834	0.656176	Q9WVM1												
			1.31617	0.927809	Q8BMJ3												
			1.337557	0.868266	E9PVA8												
			1.338892	0.428488	Q8JZN5												
			1.3428	0.510084	P26369												
			1.349692	0.637716	P08226												
			1.353866	1.179362	Q9Z1G4												
			1.35515	0.903083	Q9JKF1												
			1.376053	0.260171	Q9D8P4												
			1.380628	0.938077	Q9WVK4												
			1.382469	0.243608	Q9D2R8												
			1.40959	0.859715	Q8JZQ2												
			1.410275	0.642991	Q9JJL8												
			1.413821	0.59902	Q6NV83												
			1.417581	1.168364	Q7TSJ2												
			1.422578	0.935874	Q8BJZ4												
			1.434792	0.409533	P16460												
			1.440355	0.667609	Q8K2Y0												
			1.44816	0.713363	P01801												
			1.449621	0.59512	Q99104												
			1.450109	0.801589	Q8BK72												
			1.453384	0.308799	Q9DBH0												
			1.456202	0.738193	Q7TNC4												
			1.457778	0.787152	P01592												
			1.458873	0.762786	Q9D7N3												
			1.463731	0.775969	P01807												
			1.468983	0.457887	Q9CQ62												
			1.473554	0.84404	P61750												
			1.480399	0.951865	O08583												
			1.491977	0.856333	P03987												
			1.493713	1.165058	Q9D8X2												
			1.506599	0.478719	Q8BWQ4												
			1.510485	0.821653	P01843												
			1.514466	0.88884	P01662												
			1.523274	0.46817	P62315												
			1.52818	0.928582	P04104												
			1.529259	1.064549	Q64511												
			1.54103	0.598478	Q91Y47												
			1.550693	0.782972	Q9JKB3-2												
			1.562476	0.48099	Q9D8T7												
			1.57196	0.82744	P01680												
			1.575022	0.488219	Q9CSH3												
			1.582309	0.975223	P98086												
			1.590552	0.370095	Q8BWT1												
			1.594069	0.493691	Q9D0D3												
			1.599857	0.495264	Q9CZU4												
			1.605353	0.310368	Q8BQZ5												
			1.608985	0.79262	P32261												
			1.612614	1.001277	P01746												
			1.626563	1.081444	P01868												
			1.630238	0.941806	Q9CVB6												
			1.641478	0.849442	P01029												
			1.641715	0.730431	Q91VN4												
			1.649003	1.006213	P18525												
			1.660801	0.465752	Q9JHS4												
			1.682005	0.281733	Q8BKF1												
			1.682731	0.290915	P14069												
			1.683502	1.097854	O08582												
			1.686332	1.067534	Q9QWK4												
			1.689423	0.966169	P01786												
			1.70696	0.688191	Q6ZQ08												
			1.724691	0.74277	Q8C5N3												
			1.726975	1.122872	P01675												
			1.744875	0.355207	P58281												
			1.749289	0.970023	Q06890												
			1.758464	0.758132	Q8BJS4												
			1.764265	0.95116	P28665												
			1.765599	0.690234	P61922												
			1.775225	0.439048	P47856												
			1.778263	1.196016	Q3U9G9												
			1.783555	0.883093	Q8CG14												
			1.788501	0.834216	Q9CWN7												
			1.7947	0.36504	Q9CPQ8												
			1.7947	0.36504	Q9CRY7												
			1.802066	0.618565	Q60963												
			1.80347	0.553035	Q61207												
			1.822615	1.052271	Q61838												
			1.826829	1.150107	P01872												
			1.829483	0.772595	Q8BH35												
			1.83102	0.552847	Q9CZX0												
			1.835052	0.529246	P11680												
			1.846468	1.015366	P01027												
			1.846846	0.935847	P28658												
			1.846987	0.559407	Q9CXY6												
			1.850529	0.472068	Q8C0E2												
			1.860827	0.497363	Q9DBJ1												
			1.861279	0.456051	Q03963												
			1.866329	1.208341	P01635												
			1.872336	0.854155	Q61147												
			1.88072	0.464834	Q8BIJ6												
			1.880772	1.121642	Q52KI8												
			1.902554	0.484796	Q9CXL3												
			1.903373	0.797383	P55065												
			1.917708	1.175979	Q9R1C7												
			1.921139	0.584769	P49615												
			1.925539	0.494716	Q9CQC9												
			1.927759	1.262907	P01665												
			1.929492	1.195864	O35381												
			1.940499	0.957786	Q9CX56												
			1.957988	0.547711	Q3TZX3												
			1.96038	0.67837	Q99PU8-2											
			1.973076	1.101458	P01867												
			1.97584	0.352167	Q8K182												
			1.989729	0.744441	Q62425												
			1.999804	0.896353	P09602												
			2.001481	1.263861	P01638												
			2.002072	1.022956	P01644												
			2.005754	0.961818	Q91XU3												
			2.00892	1.295697	Q9R0X4												
			2.015968	1.110735	Q922U2												
			2.0202	0.786229	P37040												
			2.032671	1.133511	P01654												
			2.06041	0.870032	Q80ZW2												
			2.073706	0.723682	P84084												
			2.090302	0.59235	O54781												
			2.101951	0.472761	Q9DAJ4												
			2.137629	0.610374	Q9CPX7												
			2.137629	0.610374	Q9CQL5												
			2.151116	1.176505	Q8CI11												
			2.156051	0.62007	Q3TDQ1												
			2.190221	0.76774	Q9JIK9												
			2.190554	0.862957	P18531												
			2.20058	1.119835	Q6NXH9												
			2.210747	0.87233	P61021												
			2.221338	1.260264	P62996												
			2.237695	0.958686	P21614												
			2.240316	0.999594	Q3UQ44												
			2.253928	1.296919	P14106												
			2.283412	1.186132	P62307												
			2.289775	1.24551	Q8BFZ9												
			2.304172	0.451617	Q4FK66												
			2.309745	1.040887	Q61646												
			2.311906	1.115788	Q9QY81												
			2.335775	0.303372	Q5XG71												
			2.336119	1.106013	P04186												
			2.347834	0.914495	O70362												
			2.371801	0.915197	P06909												
			2.383987	0.840408	Q8K2Y7												
			2.408302	1.248335	Q62376												
			2.432257	1.033524	Q9DBE8												
			2.470857	0.643391	P01759												
			2.480316	1.263241	P01837												
			2.492145	0.924386	Q14CH7												
			2.516125	1.09237	P00375												
			2.527245	0.674141	Q9CQY5												
			2.533803	0.982665	P01671												
			2.535183	1.015257	P18242												
			2.541566	0.929242	Q61805												
			2.573273	0.834182	P28660												
			2.59805	0.581318	P01750												
			2.62144	1.157979	Q9QWL7												
			2.626127	1.097888	Q14C51												
			2.631424	1.079363	Q3UV17												
			2.667594	0.907195	Q9JIF0												
			2.675171	0.973981	Q8BH74												
			2.690148	1.196775	P61205												
			2.720063	1.019917	Q9DC51												
			2.728584	1.117948	Q8K0Z7												
			2.768969	0.975894	Q8K426												
			2.805181	1.237689	P02535												
			2.847382	0.925021	P98064-2												
			2.857244	1.266368	P01633												
			2.878148	1.29731	Q9D0G0												
			2.920365	0.830472	P54823												
			2.938217	0.975352	O88441												
			2.959641	1.096729	O88783												
			2.961611	1.172191	P01844												
			3.092814	0.873731	Q921I1												
			3.196369	0.903708	P04940												
			3.236237	1.015973	P01787												
			3.302979	1.072164	P01878												
			3.361444	1.044525	P63028												
			3.395583	0.895323	Q8VCM7												
			3.483714	1.139664	P46978												
			3.651191	0.909801	Q99P88												
			3.899881	1.037476	Q00896												
			4.34245	1.100567	Q8K0E8												
			4.699533	1.21813	P11276												
